# One‐Pot Amidation/C─H Halogenation by an Efficient Electrochemical Cascade

**DOI:** 10.1002/anie.9028210

**Published:** 2026-03-24

**Authors:** Sudipta Ponra, Ruzal Sitdikov, Hasil Aman, Alyssio Calis, Gergely Laczkó, Virgile Rouffeteau, Maxime R. Vitale, Imre Pápai, Oscar Verho

**Affiliations:** ^1^ Department of Medicinal Chemistry, Uppsala Biomedical Centre Uppsala University Uppsala Sweden; ^2^ Institute of Organic Chemistry HUN‐REN Research Centre For Natural Sciences Budapest Hungary; ^3^ Hevesy György PhD School of Chemistry Eötvös Loránd University Budapest Hungary; ^4^ Chimie Physique et Chimie du Vivant (CPCV), Département de Chimie, École Normale Supérieure, PSL University, Sorbonne Université CNRS Paris France

**Keywords:** amidation, cascade reactions, electrochemical reactions, halogenation, undivided cell

## Abstract

The advancement of sustainable synthetic methodologies is a central goal of modern chemistry, given the societal importance of green chemical practices. Amide groups and halogen atoms are prevalent in chemical and biological systems, with major relevance to both organic and medicinal chemistry. Consequently, there is strong demand for efficient methods that enable amide bond formation and selective halogenation under mild, resource‐efficient conditions. Conventional approaches typically require separate steps, activating reagents, catalysts, or harsh reaction conditions, which limit scalability and sustainability. To address these challenges, we developed a novel electrochemical cascade methodology that unites amide bond formation and electro‐induced C─H halogenation in a single, atom‐economical, and environmentally benign process. This strategy provides streamlined access to halogenated *N*‐aryl amides, carbamates, and ureas without additives or co‐reagents. The method's generality and robustness are demonstrated across more than 145 examples, encompassing complex, functional group‐dense scaffolds and pharmaceutically relevant compounds, including successful scale‐up reactions.

## Graphical abstract

A sustainable electrochemical cascade enables the one‐pot synthesis of regioselectively halogenated *N*‐aryl amides from readily available amines and acyl halides under mild conditions. The method merges amidation and C─H halogenation in a single operational step, delivering broad substrate scope, high selectivity, and scalability, and provides an efficient platform for late‐stage halogenation of complex molecular scaffolds.

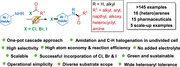

## 1 Introduction

The cascade chemistry approach represents a powerful synthetic strategy within organic synthesis [1, 2, 3], wherein multiple bond‐forming events occur sequentially under a single set of reaction conditions, without the need for isolation of intermediates. Cascade chemistry offers many advantages, including increased atom economy, reduced reaction times and purification steps, as well as reduced solvent usage, which all contribute to operational simplicity and improved sustainability. The inherent efficiency of cascade protocols makes them particularly attractive for the rapid construction of complex molecular architectures from simple starting materials. As such, they have become increasingly valuable in the synthesis of bioactive compounds, pharmaceuticals, functional materials, and various fine chemical products [1, 2, 3]. In recent years, electrochemical methods have emerged as particularly attractive platforms for cascade reactions, as they enable precise control of redox events using electrons as traceless reagents. Such approaches allow multiple transformations to be integrated into a single operation, often under mild and environmentally benign conditions.

**FIGURE 1:**
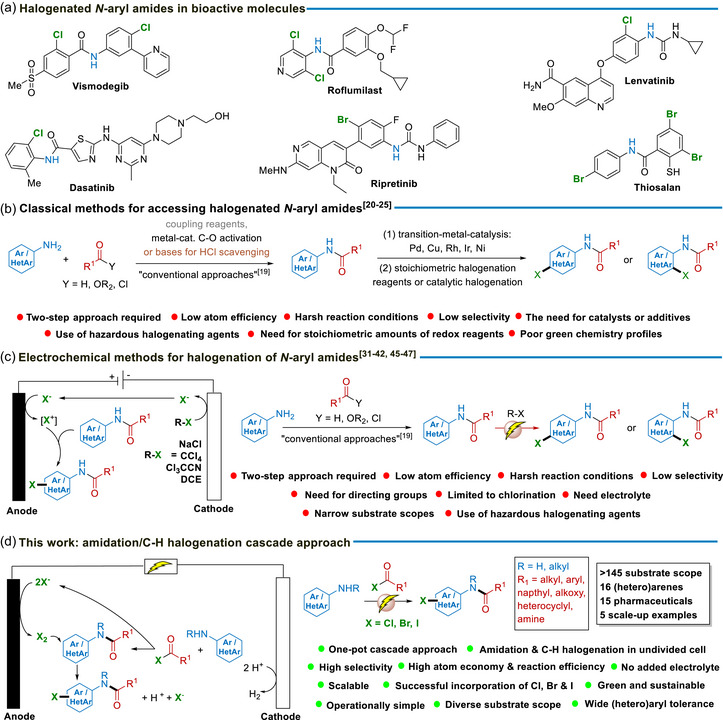
Background and reaction design. (a) Examples of bioactive *N*‐aryl amide derivatives bearing halogen groups. (b) Classical methods for accessing halogenated of *N*‐aryl amides. (c) Electrochemical methods for the halogenation of *N*‐aryl amides. (d) This work: A novel amidation/C─H halogenation cascade approach.

Despite the central importance of organohalides [4, 5, 6, 7, 8] and amides [9, 10, 11, 12, 13, 14, 15, 16, 17, 18] in organic chemistry and their widespread presence in bioactive molecules (Figure 1a), many established methods for their synthesis remain relatively inefficient and are often associated with the generation of substantial hazardous waste. As a result, there is a need within the chemistry community for new, broadly applicable strategies that enable efficient amidation and halogenation from simple starting materials under sustainable conditions. Traditionally, the synthesis of such compounds has relied on a two‐step approach in which amidation [19] and chlorination [20, 21, 22, 23, 24, 25] are performed separately using atom‐uneconomical coupling and halogenating reagents (Figure 1b). More recently, catalytic halogenation methodologies have emerged as efficient alternatives to stoichiometric and unselective chlorinating agents for the selective halogenation of *N*‐aryl amides and other organic compounds (Figure 1c) [26, 27, 28, 29, 30]. In this regard, the direct electrochemical halogenation of C─H bonds constitutes perhaps the most appealing strategy, since it enables electrons to be used as green and highly modular redox reagents for generating electrophilic species (e.g., X^+^ or X_2_) from the anodic oxidation of halide anions (X^−^), without the need of any additional redox reagents. Here, a variety of halogenating reagents have been utilized including HX‐ and X_2_‐type reagents [31, 32, 33, 34, 35, 36, 37, 38], as well as various metal halide salts [39, 40, 41, 42, 43, 44] and organic chlorine sources, such as 1,2‐dichloroethane (DCE) [45], CCl_4_ [46], Cl_3_CCN [47] and ethyl chloroformate [48]. From a conceptual standpoint, electrochemical halogenation, which involves the in situ generation of electrophilic species under mild reaction conditions, is especially well suited for achieving selective aromatic substitution reactions.

However, to the best of our knowledge, no electrochemically induced C─H halogenation method has yet been integrated into a cascade protocol involving amide bond formation, allowing the direct and convenient synthesis of halogenated amides, which are structural motifs frequently found in bioactive and pharmaceutical compounds. Herein, we describe an electrochemical, one‐pot amidation/C─H halogenation cascade protocol in an undivided cell set‐up, which can be used to access a wide range of halogenated *N*‐aryl amide, carbamate and urea compounds in high efficiency (Figure 1d). In this method, an amine is first reacted with an acyl halide in dimethylacetamide (DMA) to form the corresponding amide, which is subsequently C─H halogenated at the more electron‐rich aromatic amine ring by a reactive halogen species that is electrochemically generated from the liberated X^−^ in the first step. Remarkably, the electrochemically induced C–H halogenation step proceeds with high efficiency at room temperature (rt), accommodating a variety of acyl halides and haloformate reagents without the need for additional catalysts or additives. Furthermore, no supporting electrolyte is required beyond anilinium halide salts that are transiently formed over the course of the reactions, highlighting the operational simplicity and high sustainability of this method. Another advantage of this electrochemistry‐enabled cascade protocol is that it can be performed efficiently with near‐equimolar ratios of aromatic amine and acyl halide/haloformate reagents, making the overall transformation highly atom‐economical. The synergy of these features, combined with excellent chemo‐ and regioselectivity, affords exceptionally clean reactions with minimal byproduct formation, positioning this approach as a transformative advance in green electrosynthetic methodology.

## 2 Results and Discussion

The optimization of this electrochemical cascade protocol was conducted with aniline (**1**) and cyclohexanecarbonyl chloride (**2**) as the model substrates, and selected entries from these trials are shown in Table 1 (for the complete optimization table, see Supporting Information; Table S1). All experiments were carried out in an undivided cell without any additional additives or catalysts besides the inserted anode and cathode. As expected, no C─H chlorination was observed in the absence of current (Entry 1). On the other hand, when a carbon cloth (CC) anode and a platinum (Pt) cathode were inserted into the reaction media and a current of 5 mA was applied, a highly encouraging conversion of 93% toward the mono‐chlorinated product **3** was observed when the reaction was performed in DMA at rt for 3 h using a 1:1 ratio of the two reaction partners (Entry 2). By slightly increasing the amounts of acyl chloride **2** to 1.05 equivalent, it proved possible to achieve quantitative conversion toward product **3** within the same time frame and with a Faradaic efficiency of 71% and a total charge of 2.80 Fmol^−1^ (Entry 3).

**TABLE 1:** Optimization study of the electrochemical cascade approach.

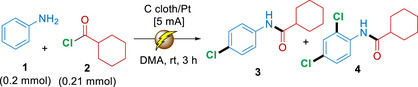		
Entry	Modification	Conv. (%) (3:4) [Total Charge, FE (%)]
1	Without electricity	0 (0:0)
2	0.2 mmol of **2** instead of 0.21 mmol of **2**	93 (93:0) [2.80, 67]
3	None	>99 (99:0) [2 80, 71]
4	2 mA for 6.5 h instead of 5 mA for 3 h	98 (98:0) [2.42, 81]
5	10 mA for 1.5 h instead of 5 mA for 3 h	>99 (93:>6) [2.80, 71]
6	0.42 mmol of **2** for 15 h	98 (55:43) [14.0, 14]
7	0.42 mmol of **2** for 15 h using 10 mA	>99 (13:79) [28.0, 7.1]

*Reagents and conditions*: substrates **1** (0.2 mmol) and **2** (0.21 mmol) dissolved in DMA (4 mL) in an undivided cell at rt. Conversions were determined by ^1^H‐NMR against 1,3,5‐trimethoxybenzene as the internal standard and LC‐MS (following work‐up); selectivity ratios given in parenthesis. Total charge in Fmol^−1^, FE (%) = Faradaic efficiency. 0% conversion means no halogenated product; only amide was formed.

Notably, this cascade protocol also works comparably well with other combinations of electrode, such as CC(+)/CC(‐), Pt(+)/Pt(‐), Pt(+)/CC(‐) and CC(+)/Ni(‐). The choice of solvent was found to have a profound impact on the reaction performance, and despite extensive screening (Table S1), DMA proved especially effective, indicating that this solvent is uniquely capable of promoting this transformation. Lowering the current to 2 mA was found to result in a slower reaction, and in this case an extended time of 6.5 h was needed in order to obtain 98% conversion to **3** (Entry 4). However, it is worth noting that this slower reaction proceeded with a higher Faradaic efficiency of 81% and a lower total charge of 2.42 Fmol^−1^. When the current was increased to 10 mA, the reaction proceeded substantially faster; however, significant formation of the dichlorinated product **4** was already observed after 1.5 h (Entry 5). Interestingly, if the acyl chloride equivalents were doubled, it proved possible to increase the conversion toward dichlorinated product **4** to 43% (Entry 6). Following further optimization of the dichlorination process (Table S1), we managed to obtain the product **4** in 79% yield by combining 10 mA current with 2 equivalents of acyl chloride **2** and performing the reaction with a CC(+)/Pt(‐) electrode pair, in DMA at rt for 15 h (Entry 7).

Encouraged by the successful chlorination cascade, we became interested in investigating if the analogous acylation/bromination approach would be possible as well. Substituting the acyl chloride with an acyl bromide under the standard conditions (5 mA current for 3 h) afforded 30% conversion toward the brominated product *N*‐(4‐bromophenyl)acetamide (Table S1). To our delight, increasing the reaction time to 4 h and using a 15 mA current resulted in a significant improvement of the reaction performance with 92% conversion toward *N*‐(4‐bromophenyl)acetamide. On the basis of the results from the optimization study with aniline, it was chosen to use a CC(+)/Pt(‐) electrode set‐up and the following reaction conditions for further substrate scope studies: 1.05 equivalents of acyl chloride and 5 mA current for mono‐chlorination; 1.05 equivalents of acyl bromide and 15 mA current for mono bromination; 2.1 equivalents of acyl chloride and 10 mA current for di‐chlorination. However, as will be shown in later large‐scale reaction examples, CC(+)/CC(‐) can serve as a more cost‐friendly replacement for CC(+)/Pt(‐) without any significant decrease in reaction performance.

**SCHEME 1:**
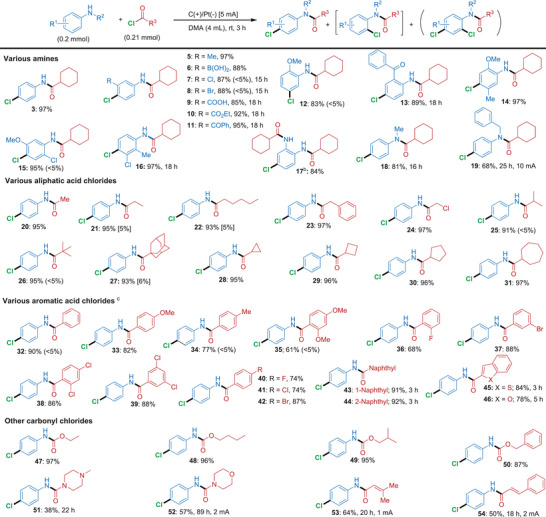
Electrochemical cascade amidation/*para*‐chlorination of various amines and acyl halides. *Reagents and conditions*: ^a^ standard experiment unless otherwise noted: aniline (0.2 mmol) and acyl chloride (0.21 mmol) in DMA (4 mL), in an undivided cell with carbon cloth (anode) and platinum (cathode). ^b^ cyclohexanecarbonyl chloride (0.42 mmol), 4 h; ^c^ 3.5 h All yields refer to isolated yields; mono‐*ortho* chlorinated and di‐chlorinated product in parenthesis. In the Supporting Information, the total charge for each reaction as well as the Faradaic efficiencies for selected entries have been provided.

**SCHEME 2:**
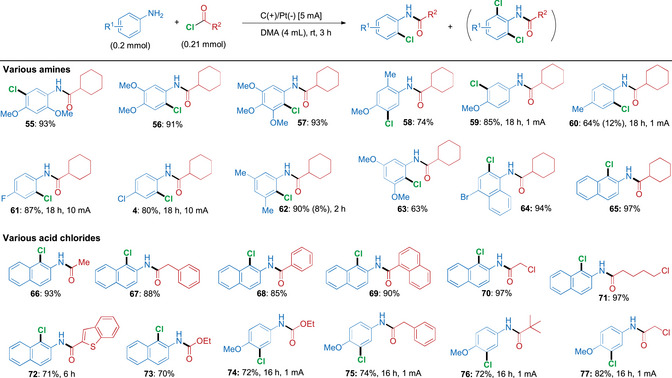
Electrochemical cascade amidation/*ortho*‐chlorination of various amines and acyl chlorides. *Reagents and conditions*: aromatic amine (0.2 mmol) and acyl chloride (0.21 mmol) in DMA (4 mL), in an undivided cell with carbon cloth (anode) and platinum (cathode). All yields refer to isolated yields; mono‐*ortho* chlorinated and di‐chlorinated product in parenthesis. In the Supporting Information, the total charge for each reaction as well as the Faradaic efficiencies for selected entries have been provided.

For the investigation of the mono‐chlorination cascade, a wide range of aromatic amines and acyl chlorides with either electron‐donating or electron‐withdrawing substituents were surveyed in order to provide a detailed picture of the functional group tolerance and limitations of this cascade reaction (Schemes 1 and 2). First, aniline derivatives possessing a free *para*‐position were investigated with a range of acyl chlorides to establish the regioselectivity of this cascade (Scheme 1). Within this substrate class, aniline derivatives bearing electron‐donating or electron‐withdrawing substituents in the *meta*‐ and *ortho*‐positions were initially examined using cyclohexanecarbonyl chloride (**2**) as a representative acyl chloride. As shown by these reactions, this electrochemically induced cascade protocol displays a broad functional group tolerance by allowing the use of several synthetically useful moieties including boronic acid, halogen, carboxylic acid, ester and keto groups, while favoring almost exclusively the formation of the *para*‐substituted products **4–13** in high yields ranging between 83% and 95%. Here, we witnessed an electronic trend, where simple aniline (**1**) or anilines having electron‐donating functionalities reacted smoothly under standard conditions, while electron‐deficient anilines reacted less efficiently and required extended reaction time of 15–18 h and higher total charges (14.0–16.8 Fmol^−1^). In the case of the reaction forming product **12**, it was demonstrated that a methoxy‐substituent could exert a stronger *para*‐directing influence than the amide group.

Disubstituted anilines of various patterns and substituents were also well tolerated by this electrochemically induced cascade protocol, as shown by the reactions furnishing the *para*‐substituted products **14–16** in 95%–97% yield. When *ortho*‐phenylenediamine was used together with 2 equiv of acyl chloride **2**, product **17** arising from diacylation followed by mono‐chlorination, was isolated as the major product in 84% yield after 4 h and a total charge of 3.73 Fmol^−1^. *N*‐alkylated anilines were also reactive under the optimized conditions, enabling access to chlorinated amide products **18** (Me substituent, 81% yield) and **19** (Bn substituent). Interestingly, however, prolonged reaction times and higher current (10 mA) were required, suggesting that secondary anilines lacking a free N─H moiety react more slowly in this cascade protocol. Acyl chlorides carrying different aliphatic, cycloaliphatic, and benzylic substituents gave the corresponding 4‐Cl *N*‐aryl amide products **20–31** in 91%–97% yield within 3 h and a total charge of 2.8 Fmol^−1^, with no or only minor formation of di‐chlorinated byproducts being observed.

Aromatic acyl chlorides with various substituents were also well‐tolerated and afforded *para*‐substituted products **32–42** in 61%–90% yield, when slightly extended reaction times were used (3.5 h, corresponding to total charges of 3.26 Fmol^−1^). Also, naphthoyl chlorides were compatible with this electrochemically induced cascade, which allowed access to the products **43** and **44** in 91% and 92% yield, respectively. Similarly, heteroaromatic acyl chlorides provide exclusively *para*‐chlorinated products. Benzo[*b*]thiophene‐2‐carbonyl chloride under standard conditions afforded product **45** in 84% yield, while the reaction employing benzofuran‐2‐carbonyl chloride required a longer reaction of 5 h to give **46** in 78% yield. Interestingly, a wide range of chloroformate reagents were also compatible with this cascade approach, allowing the synthesis of the *para*‐chlorinated carbamate products **47–50** in 87%–97% yield within 3 h and using a total charge of 2.8 Fmol^−1^. We also investigated the versatility of our methodology for piperazine and morpholine carbonyl chlorides, which with extended reaction times afforded the *para*‐chlorinated urea derivatives **51** and **52** in 38% and 57% yield, respectively. This method could also be applied to α,β‐unsaturated acyl chlorides such as 3‐methylbut‐2‐enoyl chloride and cinnamoyl chloride. By applying lower currents (1–2 mA) and extended reaction times (18–20 h), the *para*‐chlorinated amide products **53** and **54** were obtained in synthetically useful yields (64% and 50%). Looking at the results presented herein, it can be concluded that the present method is highly general and can be applied to a broad range of substrates of varying structural complexity to furnish various *para*‐chlorinated *N*‐aryl amide and carbamate derivatives in good to excellent yields.

In the case of aromatic amines bearing blocked *para*‐positions (Scheme 2), C─H chlorination instead proceeded with high selectivity at the *ortho*‐position relative to the amide substituent. An exception was observed for the formation of products **55** and **58**, where the strongly electron‐donating methoxy group overrode the directing influence of the amide substituent. Notably, di‐ and tri‐substituted anilines with electron‐donating groups displayed higher reactivity with our cascade protocol, which enabled access to products **55–58** in 74–93% yield under the standard conditions. In contrast, the use of 4‐methoxyaniline as a substrate required a prolonged reaction time of 18 h and a lower current of 1 mA to afford product **59** in 85% yield. Here, the starting material exhibits a relatively low oxidation potential, which likely explains the need for such low current conditions. Operating under more gentle conditions enhances chemoselectivity during the oxidation process.

A similar need for a lower current was also observed for the reaction between 4‐methylaniline and acyl chloride **2**, which yielded product **60** in 64% yield, when performed with 1 mA current over 18 h. In the case of electron‐deficient 4‐fluoro and 4‐chloroaniline reacting with acyl chloride **2**, a higher current of 10 mA and extended reaction times were required in order to give 87% and 80% yield of **61** and **4**. It is worth noting that, in the case of 3,5‐dimethylaniline and 3,5‐dimethoxyaniline with an unsubstituted *para* position, C─H chlorination proceeded with high *ortho* selectivity to afford **62** in 90% (2 h) and **63** in 63% yield, respectively. Naphthyl amines proved to be excellent substrates for this cascade as well, as demonstrated by 4‐bromo‐1‐naphthyl amine providing **64** in 94% yield with only a total charge of 2.8 Fmol^−1^. For 2‐naphthyl amines, chlorination was found to occur exclusively at the more nucleophilic 1‐position of the naphthyl ring with a broad scope of acyl chlorides and chloroformates carrying both aliphatic and aromatic substituents. These reactions furnished products **65–73** in high to excellent yield (70%–97%) under the standard conditions and using a total charge of 2.8 Fmol^−1^.

Different acyl chlorides and chloroformates were similarly reacted with 4‐methoxyaniline over extended times and using modified reaction conditions. When a lower current of 2 mA was used, the products **74–77** were obtained in 64%–82% yields, corresponding to a total charge of 3.0 Fmol^−1^.

**SCHEME 3:**
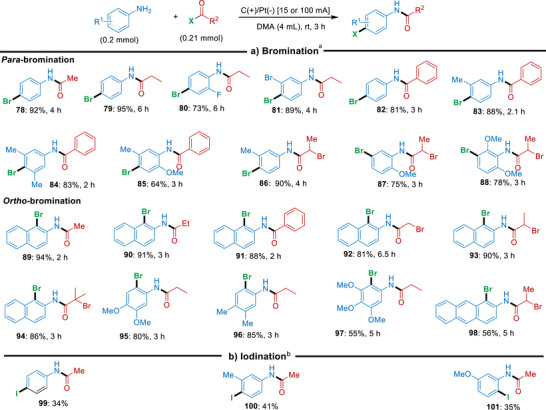
Electrochemical cascade amidation/bromination or iodination of various amines and acyl halides. *Reagents and conditions*: ^a^ bromination: aromatic amine (0.2 mmol) and acyl bromide (0.21 mmol) in DMA (4 mL), 15 mA, in an undivided cell with carbon cloth (anode) and platinum (cathode), yields refer to isolated yields. ^b^ iodination: aniline (0.2 mmol) and acyl iodide (0.21 mmol) in DMA (4 mL), in an undivided cell with carbon cloth (anode) and platinum (cathode), 100 mA, 45 h, yields determined by NMR. In the Supporting Information, the total charge for each reaction as well as the Faradaic efficiencies for selected entries have been provided.

After having studied the C─H chlorination cascade, we explored the generality of the amidation/bromination approach with various amines and acyl bromides using 15 mA current (Scheme 3a). Here, we observed similar trends as for the chlorination cascades, where amines with unoccupied *para*‐position were found to react with various acyl bromides to provide the corresponding 4‐Br products **78–88** in 64%–95% yield (with total charges ranging between 5.60 and 16.8 Fmol^−1^). Comparable regioselectivity to that observed in the corresponding C─H chlorination reactions was also found for aromatic amine partners lacking accessible *para*‐positions; in these cases, the positions directly adjacent to the amide group were selectively brominated. Different kinds of substituted amines were found to react in high efficiency with a variety of acyl bromides to give the 2‐brominated products **89–98** in 55%–94% yield with total charges ranging between 5.60 and 18.2 Fmol^−1^. However, it should be noted that the bromination cascade exhibited lower Faradaic efficiencies than the corresponding chlorination cascade. This can be attributed to the slower overall reaction profile of the bromination cascade, which necessitates the application of higher currents and longer electrolysis times. Under these conditions, a larger fraction of the passed charge is likely consumed by non‐productive background processes, such as solvent oxidation.

This cascade approach could also be extended to the corresponding amidation/iodination sequence (Scheme 3b), although this transformation proved significantly less effective. When using acetyl iodide as the acyl halide source and increasing the applied current to 100 mA, the 4‐iodo‐substituted products **99–101** could be afforded in 34%–41% yield (as determined by NMR). This corresponds to a total charge of approximately 839 Fmol^−1^, indicating that, while feasible in principle, further optimization will be required to enhance the practical synthetic utility of this cascade approach for the preparation of iodinated amide derivatives.

**SCHEME 4:**
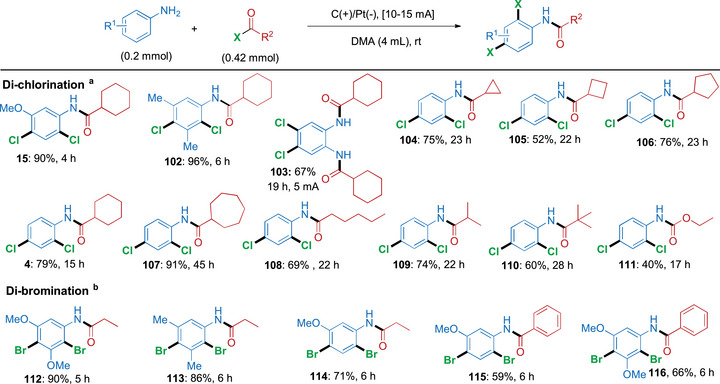
Electrochemical cascade amidation/di‐halogenation. *Reagents and conditions*: ^a^ Di‐chlorination: aniline (0.2 mmol) and acyl chloride (0.42 mmol) in DMA (4 mL), in an undivided cell with carbon cloth (anode) and platinum (cathode); 10 mA current. ^b^ Di‐bromination: amine (0.2 mmol) and acyl bromide (0.42 mmol) in DMA (4 mL), in an undivided cell with carbon cloth (anode) and platinum (cathode), 15 mA current. All yields refer to isolated yields. In the Supporting Information, the total charge for each reaction as well as the Faradaic efficiencies for selected entries have been provided.

After having extensively surveyed the mono‐halogenation cascade, we then turned our attention toward the scope of the di‐chlorination variant (Scheme 4). Anilines containing either 3‐methoxy or 3,5‐dimethyl groups reacted faster than unsubstituted aniline with acyl chloride **2** to provide the di‐chlorinated products **15** in 90% yield after 4 h and **102** in 96% yield after 6 h (corresponding to total charges of 7.46 and 11.2 Fmol^−1^, respectively). In the case of *ortho*‐phenylenediamine, we were able to achieve diacylation in parallel to di‐chlorination **103** by carrying out the reaction at a lower current (5 mA) for 6 h. This cascade amidation/di‐chlorination strategy also displays a broad tolerance toward different cyclic and acyclic acyl chlorides, as shown by the reactions furnishing 2,4‐di‐chlorinated products **4** and **104–110** in yields ranging between 52% and 91%. Using ethyl chloroformate as the reaction partner resulted in the formation of the 2,4‐di‐chlorinated carbamate product **111** in 40% yield after 17 h and a total charge of 31.7 Fmol^−1^. Similarly, by increasing to 2 equivalents of acyl bromide and extending the reaction time to 5–6 h, it proved possible to direct this electrochemical cascade protocol toward the di‐brominated products as well, as demonstrated by the reactions giving products **112–116** in 59%–90% yield with total charges ranging between 14.0 and 16.8 Fmol^−1^.

**SCHEME 5:**
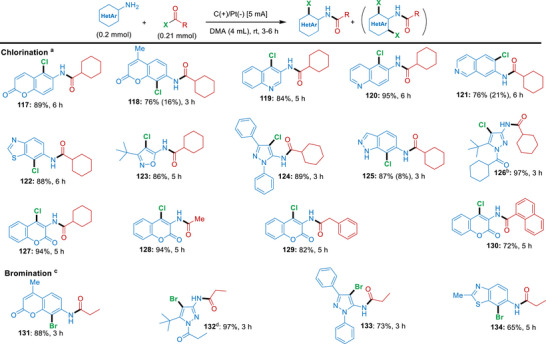
Electrochemical cascade amidation/halogenation of various heteroaromatic compounds. *Reagents and conditions*: ^a^ standard chlorination experiment unless otherwise noted: heteroaromatic amine (0.2 mmol) and acyl chloride (0.21 mmol), in DMA (4 mL), in an undivided cell with carbon cloth (anode) and platinum (cathode) using 5 mA current. ^b^ Chlorination with modified conditions: acyl chloride (0.42 mmol); ^c^ standard bromination experiment unless otherwise noted: heteroaromatic amine (0.2 mmol) and acyl bromide (0.21 mmol), in DMA (4 mL), in an undivided cell with carbon cloth (anode) and platinum (cathode) using 15 mA current; ^d^ Bromination with modified conditions: propionyl bromide (0.42 mmol). All yields refer to isolated yields; di‐chlorinated product in parenthesis. In the Supporting Information, the total charge for each reaction as well as the Faradaic efficiencies for selected entries have been provided.

Gratifyingly, this electrochemical cascade protocol can also be applied in high efficiency to a wide range of heteroaromatic amines, as shown by the results summarized in Scheme 5. Here, heteroaromatic amines comprising scaffolds such as coumarin, quinoline, benzothiazole, isoxazole, pyrimidine, pyrazole, and indazole, were all well‐tolerated and gave the products **117–127** in 76%–97% yield after 3–6 h. Only a few heteroaromatic amine substrates (giving products **118**, **121** and **125**) showed minor formation of the di‐chlorination byproducts. Also, these reactions were highly flexible in terms of acyl chloride partners, as acyl chlorides carrying, methyl, benzyl and 1‐naphthyl substituents all reacted smoothly with 3‐aminocoumarin to afford products **128–130** in 72%–94% yield after 6 h (with 4.66 Fmol^−1^ total charge). It is worth noting that heterocyclic amines also reacted efficiently with propionyl bromide to produce the brominated products **131–134** in 65%–97% yield (total charge 8.39–14.0 Fmol^−1^).

**SCHEME 6:**
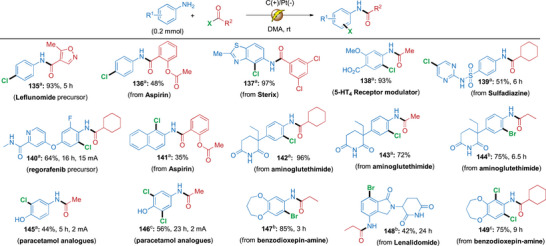
Cascades involving pharmaceutically‐relevant molecules. *Reagents and conditions*: ^a^ aromatic or heteroaromatic amine (0.2 mmol) and acyl chloride (0.21 mmol) in DMA (4 mL), 5 mA, 3 h; ^b^ aromatic or heteroaromatic amine (0.2 mmol) and acyl bromide (0.21 mmol) in DMA (4 mL), 15 mA; ^c^ aromatic or heteroaromatic amine (0.2 mmol) and acyl chloride (0.42 mmol) in DMA (4 mL), 10 mA. All yields refer to isolated yields. All reactions were carried out in an undivided cell with carbon cloth (anode) and platinum (cathode) unless otherwise mentioned. In the Supporting Information, the total charge for each reaction as well as the Faradaic efficiencies for selected entries have been provided.

Furthermore, the developed cascade acylation/C─H halogenation is applicable for late‐stage modification of pharmaceuticals and other bioactive compounds (Scheme 6). To our delight, this electrochemical cascade protocol could be successfully used to access several medicinally important chlorinated compounds (Method A), as well as chlorinated analogues or intermediates of compounds such as Leflunomide (**135**, 93%), Aspirin (**136** and **141**, 48% and 35%), Sterix^48^ (**137**, 97%), a 5‐HT_4_ receptor modulating compound, (**138**, 93%), Sulfadiazine (**139**, 51%), a Regorafenib precursor (**140**, 64%), Aminoglutethimide (**142**, 96% and **143**, 72%), and Paracetamol (**145**, 44%). Similarly, the corresponding cascade bromination using acyl bromides (Method B) were also compatible for the pharmaceutical molecules: Aminoglutethimide (**144**, 75%), Benzodioxepin‐amine (**147**, 85%) and Lenalidomide (**148**, 42%). By doubling the amount of the acyl chloride reagent (Method C) we were able to prepare the di‐chlorinated analogue of Paracetamol **146** in 56% yield (2 mA current for 23 h), while benzodioxopin‐amine provided **149** in 75% yield (10 mA current for 9 h). Overall, the results summarized in Scheme 6, clearly showcase the high synthetic utility of the developed acylation/C─H halogenation cascade protocols for accessing both known pharmaceutical compounds, as well as completely new analogues whose biological activity could be interesting to study further.

**SCHEME 7:**
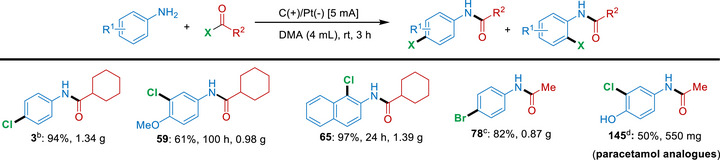
Scale up examples of electrochemical cascade amidation/halogenation. *Reagents and conditions*: ^a^ Standard experiment unless otherwise noted: amine (5 mmol), cyclohexanecarbonyl chloride (5.25 mmol), and DMA (30 mL), 20 mA current in an undivided cell with carbon cloth (anode and cathode); ^b^ Aniline (6 mmol), cyclohexanecarbonyl chloride (6.3 mmol), 28 h. ^c^ Acetyl bromide (5.25 mmol), 100 mA current, 23 h. ^d^ Acetyl chloride (5.25 mmol), 2 mA, 65 h. All yields refer to isolated yields.

Notably, the electrochemical cascade protocol exhibited good scalability. Chlorinated compounds **3**, **59**, and **65** could be synthesized on a ≥1 g scale using carbon cloth as both the anode and cathode with Faradaic efficiencies of 54%, 8.2%, and 54%, respectively. Similarly, 870 mg of brominated compound **78** was obtained within 23 h (5 mmol scale, 100 mA) using an analogous scale‐up setup, corresponding to an isolated yield of 82%. The scalability of the method was further demonstrated by the synthesis of the pharmaceutically relevant compound **145** on a >0.5 g scale in 50% yield. Although gram‐scale syntheses were achieved for multiple substrate classes, certain transformations required prolonged reaction times and elevated current densities. In particular, amines bearing electron‐withdrawing substituents exhibited reduced reactivity, leading to diminished yields. Overall, the results summarized in Scheme 7 underscore the broad synthetic utility and practical potential of this cascade methodology for large‐scale applications.

The efficient performance of this cascade in the absence of any added supporting electrolyte can be attributed primarily to two factors. First, the solvent DMA plays a crucial general role in facilitating electrochemical transformations owing to its high dielectric constant and relatively low resistivity. Second, in situ formation of anilinium halide salts provides a transient electrolyte during the initial stages of the reaction, which is enabled by the HX that is released as a consequence of the first acylation step. These anilinium species are then gradually consumed toward the end of the process following cathodic reduction of protons to molecular hydrogen, which enables regeneration of free aniline and ensures that the cascade can go to completion. This progressive depletion of the in situ generated anilinium halide electrolyte toward the end of the reaction could be observed indirectly from the cell voltage that was found to significantly increase toward the end of the reaction.

To gain insight into the underlying mechanism of our cascade, a series of control and monitoring experiments were performed. NMR and LC–MS analyses of mixtures containing amine and acyl halide in DMA prior to electrolysis confirmed that amide formation occurs in the absence of an applied current (Table S1, entry 2). With an applied current, LC–MS monitoring revealed continuous formation of *N*‐aryl halogenated products, accompanied by a corresponding decrease in *N*‐aryl amide concentration (Table S2a). This observation supports a sequential pathway involving initial amide formation followed by electrochemically induced C–H halogenation mediated by anodically generated electrophilic halogen species [31, 32, 33, 34, 35, 36, 37, 38, 39, 40, 41, 42, 43, 44, 45, 46].

**FIGURE 2:**
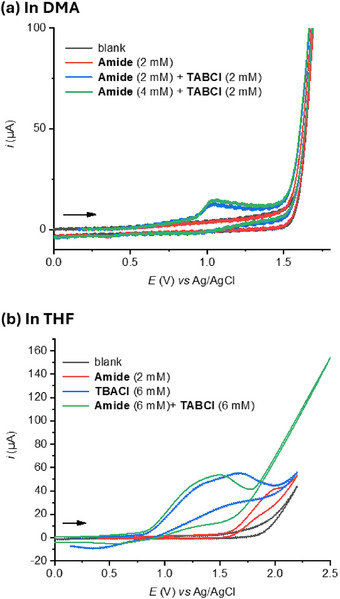
Cyclic voltammetry studies. Measurements were carried out using n‑Bu_4_NBF_4_ (0.1 M) as the supporting electrolyte in anhydrous, degassed solvent at 20 °C, employing a 3 mm glassy carbon working electrode, a platinum wire counter electrode, and an Ag/AgCl (3 M NaCl) reference electrode, with a scan rate of 100 mV s^−^^1^. “Amide” refers to *N*‑phenylcyclohexanecarboxamide.

To conclusively rule out the alternative reaction mechanism in which the halogenation step would originate from an initial anodic oxidation of the transient amide's aromatic core, we performed cyclic voltammetry (CV) experiments (Figure 2). Specifically, we investigated the electrochemical behavior of *N*‐phenylcyclohexanecarboxamide in the presence and absence of chloride anions. While the oxidation peak potential of *N*‐phenylcyclohexanecarboxamide could not be determined in DMA due to the solvent oxidation limit (“solvent wall”, Figure 2a), it was observable in tetrahydrofuran (THF) at approximately 1.8 V vs Ag/AgCl (Figure 2b). Upon addition of tetra‐*n‐*butylammonium chloride (TBACl) as a chloride source, a distinct oxidation peak appeared in both solvents at approximately 1.0 V vs Ag/AgCl, corresponding to the oxidation of Cl^−^. Hence, the halogenation step certainly stems from the anodic oxidation of the halogen anion released upon amide bond formation, producing electrophilic halogen cation species [39, 45, 46, 48], or halogen gas [47].

**FIGURE 3:**
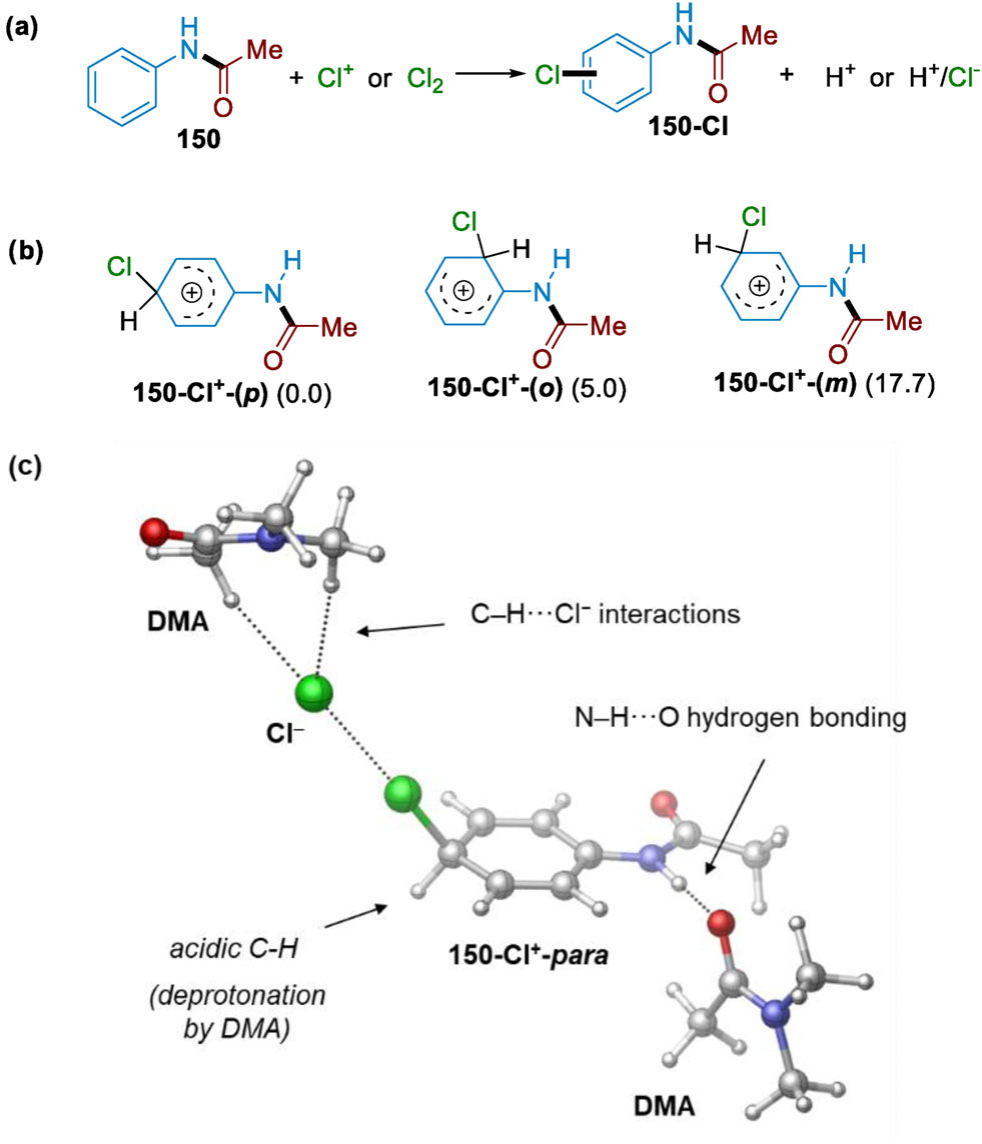
Results of computational analysis. (a) Examined reactions. (b) Computed relative stabilities (in kcal mol^−1^) of the isomeric forms of **150**‐Cl^+^ arenium intermediate (*para*, *ortho* and *meta* isomers). (c) Computed structure of σ‐complex intermediate formed upon the electrophilic attack of Cl_2_ at the aromatic carbon in the *para* (*p*) position of **150**. The role of solvent DMA molecules is highlighted by arrows.

To gain further insight into the C─H halogenation mechanism and the specific role of DMA, we carried out density functional theory (DFT) calculations on the C─H chlorination of amide **150**, derived from aniline and acetyl chloride (Figure 3a; see Supporting Information for further details). We considered electrophilic chlorination pathways via Cl^+^ and Cl_2_ as possible electrophilic species; however, only the latter pathway could account for the observed selectivity toward the *para*‐chlorinated products (see the Supporting Information, sections C2 and C3). This pathway is consistent with the generally accepted stepwise mechanism of electrophilic aromatic substitution (EAS) [49, 50, 51] that involves the formation of a transient arenium ion species (σ‐complexes) as a key reaction intermediate, followed by facile deprotonation (for relevant computational studies, see Refs. [52, 53, 54, 55]). Further support for an EAS‐type mechanism was obtained from kinetic isotope labeling experiments conducted on both the full cascade and the isolated C–H halogenation step (Table S2c). In these studies, insignificant kinetic isotope effects (KIE = 1.02–1.04) were observed, consistent with the rate‐determining step of electrophilic aromatic substitution not involving C–H bond cleavage.

The *N*‐amide group is a classic *ortho*/*para* director in EAS reactions, which is clearly borne out by the computed relative stabilities of the three isomeric forms of the σ‐complex intermediate (Figure 3b). The *meta* isomer is highly unfavored and inaccessible, and the *para* form is favored over the *ortho* by 5 kcal • mol^−1^. This implies that the regioselectivity can be associated with the thermodynamic preference of the *para*‐form of the transient arenium intermediates (Figure S14).

DFT calculations reveal that the heterolytic cleavage of Cl_2_ is facilitated by solvent molecules (Figure 3c). DMA thus acts as a catalyst by stabilizing the cationic chlorinated intermediate through enhanced N–H···O hydrogen bonding, as well as the forming chloride anion via noncovalent C–H···Cl^−^ interactions [56, 57]. The reaction proceeds via facile and highly exothermic deprotonation of the σ‐complex, with DMA acting as a base. The specific interactions with solvent molecules are in line with the experimental observation that the choice of solvent has a profound effect on the performance. The importance of the N–H···O hydrogen bond is further supported by the significantly slower reactions of *N*‐alkylated amides (**18** and **19** in Scheme 1).

**SCHEME 8:**
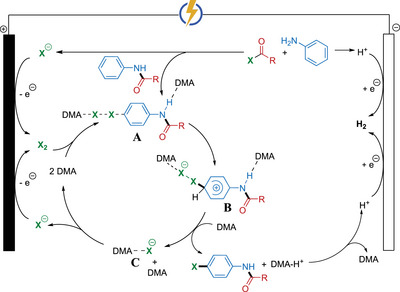
Plausible reaction pathway of electrochemical cascade *para*‐halogenation.

Based on our mechanistic investigations, we propose a cascade halogenation process as outlined in Scheme 8. The process begins with formation of the *N*‐aryl amide via acylation of the amine with the acyl chloride, releasing a halide anion in the process. Anodic oxidation of this halide anion generates molecular halogen (X_2_), and interacts with DMA and the aromatic ring of the amide in the solution phase (**A**). Next, DMA‐assisted heterolytic cleavage of X_2_ affords a transient σ‐complex intermediate, preferentially in the *para*‐configuration (**B**), which undergoes deprotonation by DMA. Finally, the released, DMA‐stabilized X^−^ (**C**) is re‐oxidized at the anode to regenerate X_2_, which can enter the next reaction cycle. This mechanism highlights the multiple role of DMA in the reaction, enabling highly efficient electrochemical halogenation of complex molecular scaffolds.

## 3 Conclusion

In summary, we have developed a mild, additive‐free electrochemical cascade protocol that enables the efficient synthesis of halogenated *N*‐aryl amides from readily available amines and acyl halides. The method combines amidation and C─H halogenation in a single operational step under environmentally benign conditions, providing broad substrate scope, high regioselectivity, and demonstrated scalability. The protocol is applicable to complex and pharmaceutically relevant molecules, highlighting its practical utility. Ongoing studies are focused on expanding this strategy to other substrate classes and related electrochemical cascade transformations.

## Conflicts of Interest

The authors declare no conflict of interest.

## Supporting information




**Supporting File 1**: anie71887‐sup‐0001‐SuppMat.pdf.

## Data Availability

The data that support the findings of this study are available from the corresponding author upon reasonable request.
