# Supplementary material for: One‐Pot Amidation/C─H Halogenation by an Efficient Electrochemical Cascade
Source: Angew Chem Int Ed Engl. 2026 Mar 24;65(19):e9028210. doi: 10.1002/anie.9028210 (PMC13134629; doi:10.1002/anie.9028210)

One-Pot Amidation/C-H Halogenation by an Efficient Electrochemical Cascade

Sudipta Ponra,*† Ruzal Sitdikov,† Hasil Aman,† Alyssio Calis,† Gergely Laczkó,§,¥ Virgile Rouffeteau,‡ Maxime R. Vitale,*‡ Imre Pápai,*§ Oscar Verho*†

† Uppsala Biomedical Centre, Department of Medicinal Chemistry, Uppsala University, SE-75123, Uppsala, Sweden

§ HUN-REN Research Centre for Natural Sciences, Institute of Organic Chemistry, Magyar Tudósok Körútja 2, H-1117 Budapest, Hungary

¥ Hevesy György PhD School of Chemistry, Eötvös Loránd University, P.O. Box 32, Budapest H-1518, Hungary

‡ Chimie Physique et Chimie du Vivant (CPCV), Département de Chimie, École Normale Supérieure, PSL University, Sorbonne Université, CNRS, 75005 Paris, France.

E-mail: oscar.verho@ilk.uu.se, sudiptaponra@gmail.com, maxime.vitale@ens.psl.eu, papai.imre@ttk.hu

Table of contents

Experimental section..S2

General chemistry information S2

Table 1: Optimization study of the electrochemical cascade approach …..………………...S3

Table 2a. Formation and engagement of amides S4

Table 2b. Halogenation of amides by different halogenating reagents S4

Table 2c. Kinetic isotope effect starting from amide with different brominating reagent S5

Table 2d. Effect of bromine concentration on bromination reaction....................................S6

General procedure A: Electrochemical cascade amidation/halogenation of various amines and acid halides S6

Electrochemical small scale reaction setup images S7

General procedure B: Electrochemical cascade amidation/dihalogenation S40

General procedure C: Electrochemical acylation/halogenation of various heteroaromatics S46

Cascades involving pharmaceutically-relevant molecules S53

General procedure D: Scale-up S58

Electrochemical large scale reaction setup images S59

List of reported compounds S61

Computational studies..S61

Computational approach S61

Computational results..S62

C1 – Stability of σ-complexes..S63

C2 – Cl^+ as a chlorinating agent...S63

C3 – Cl_2 as a chlorinating agent...S67

C4 – Computed energy components of reported structures S73

References..S74

Cartesian Coordinates of the Calculated Structures……………………………….……..S79

NMR spectra S98

NMR studies on DMA.Br_2 complex S251

General information. All substrates, reagents, and solvents were commercially available and used without further purification. Heating was carried out using a 17.4 mm DrySyn reaction vial insert compatible with 10 mL microwave vials. Purifications were performed on an automated Biotage Isolera Flash Chromatography System using 25 or 10 g prepacked Biotage SNAP KP-SIL columns. Accurate mass values were determined on a mass spectrometer equipped with an electrospray ion source and TOF detector. NMR spectra were recorded on a Bruker Avance III HD at 25 °C and 400 MHz for ^{1}H,

101 MHz for ^{13}C, and ^{19}F at 376.5 MHz using a SmartProbe BB/^{1}H probe or on a Varian Mercury plus at 25 °C and 400 MHz for ^{1}H, 101 MHz for ^{13}C, and ^{19}F at 376.5 MHz. Chemical shifts (δ) are reported in ppm, indirectly referenced to tetramethylsilane (TMS) via the residual solvent signal (^{1}H: $CHCl_3$ δ 7.26, CD_3OD δ 3.31, ^{13}C: $CDCl_3$ δ 77.2, CD_3OD δ 49.0). Electrocatalytic reactions were performed with platinum (Pt) electrodes (25 mm × 10 mm × 0.125 mm, 99.9%; obtained from redox.me, Sweden) and carbon cloth (C) electrodes (15 mm × 10 mm × 410 µm) obtained from Fuel Cell Store, USA) connected by stainless steel adapters. Electrocatalytic reactions were conducted using an AX-3005PQ programmable power supply from AXIOMET.

Table S1. Optimization study of the electrochemical cascade approach [a]

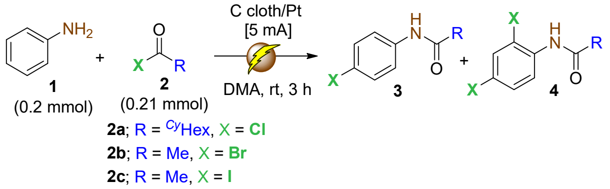

Entry	RCOX	Modification	Conversion (%) (mono:di) [Total Charge]
1	**2a**	None	>99 (99:0) [2.80]
2	**2a**	Without electricity	0 (0:0)
3	**2a**	0.2 mmol of **2a**	93 (93:0)
4	**2a**	graphite rod and graphite felt/C cloth instead of C cloth/Pt	97 (97:0), 97 (97:0)
5	**2a**	C cloth/C cloth instead of C cloth/Pt	97 (97:0)
6	**2a**	Pt/Pt instead of C cloth/Pt	99 (99:0)
7	**2a**	Pt/C cloth instead of C cloth/Pt	98 (98:0)
8	**2a**	SS, Cu, Ni, Zn or Pb as anode instead of Pt	>99 (88:12), >99 (97:3), 76 (75:1), 86 (86:0), 22 (22:0)
9	**2a**	DMF, MeCN, DCE instead of DMA	41 (41:0), 0 (0:0), 0 (0:0)
10	**2a**	NMP for 6 h instead of DMA for 3 h	>99 (98:2) [5.60]
11	**2a**	GVL or propylene carbonate instead of DMA	68 (66:2), 44 (44:0),
12	**2a**	2 mA for 6.5 h instead of 5 mA for 3 h	98 (98:0) [2.42]
13	**2a**	10 mA for 1 h instead of 5 mA for 3 h	95 (95:0) [1.86]
14	**2a**	10 mA for 1.5 h instead of 5 mA for 3 h	>99 (93:>6) [2.80]
15	**2a**	0.42 mmol of **2a** for 15 h	98 (55:43) [13.99]
16	**2a**	0.42 mmol of **2a** for 40 h	99 (43:56) [37.31]
17	**2a**	0.42 mmol of **2a** for 15 h using 10 mA	>99 (13:79) [27.99]
18	**2a**	0.42 mmol of **2a** for 23 h using 10 mA	>99 (9:70) [42.90]
19	**2b**	Without electricity	0 (0:0)
20	**2b**	None	30 (30:0)
21	**2b**	C cloth/C cloth instead of C cloth/Pt	29 (29:0)
22	**2b**	SS, Cu, Ni or Zn as anode instead of Pt	30 (30:0), 0 (0:0), 12 (12:0), 5 (5:0)
23	**2b**	10 mA instead of 5 mA	43 (43:0) [5.60]
24	**2b**	15 mA instead of 5 mA	76 (76:0) [8.39]
25	**2b**	15 mA for 4 h instead of 5 mA for 3 h	92 (92:0) [11.19]
26	**2c**	None	0 (0:0)
27	**2c**	100 mA for 45 h instead of 5 mA for 3 h	34 (34:0) [839.38]

[a] *Reagents and conditions*: Substrates **1** (0.2 mmol) and **2** (0.21 mmol) dissolved in DMA (4 mL) in an undivided cell at rt. Conversions were determined by ^{1}H-NMR against 1,3,5-trimethoxybenzene as the internal standard and LC-MS following work-up; selectivity ratio in parentheses. Total Change in $F.mol^{-1}$. 0 % conversion means no halogenated product; only amide is formed.

Table S2a. Formation and engagement of amides [a]

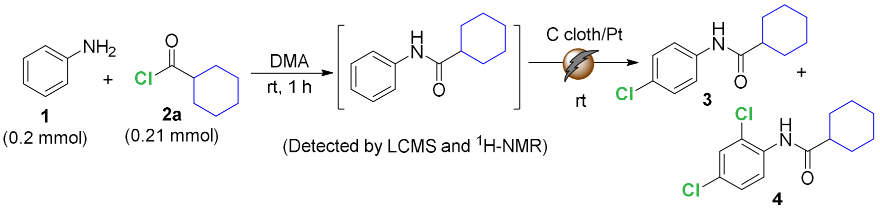

Entry	Current (mA)	Time (h)	Amide left (%)	3:4 (%) [Total Charge]
1	2	3	37	63:0 [1.12]
2	2	5	16	84:0 [1.86]
3	2	6.5	2	98:0 [2.42]
4	5	2	25	75:0 [1.86]
5	5	3	<1	99:0 [2.80]
6	5	3.5	0	91:9 [3.26]
7	10	1	5	95:0 [1.86]
8	10	1,5	0	>93:>6 [2.80]

[a] *Reagents and conditions*: Substrates **1** (0.2 mmol) and **2a** (0.21 mmol) dissolved in DMA (4 mL) at rt. Conversions were determined by ^{1}H-NMR against 1,3,5-trimethoxybenzene as the internal standard and LC-MS following work-up. Total Change in F.mol^{-1}.

Table S2b. Halogenation of amides by different halogenating reagents [a]

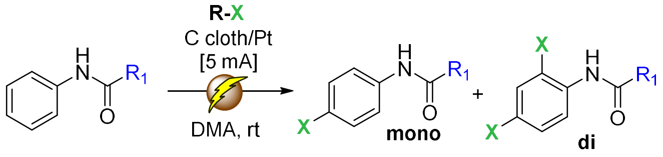

Entry	R_1	R-X (equiv.)	Time (h)	Conversion (%)	mono:di (%) [Total Charge]
1	Ph	NaCl (2)	20	59	10:0 [18.65]
2	Ph	NaBr (2)	20	55	14:0 [18.65]
3	Ph	$ClCO_2Et$ (2)	3	52	49:0 [2.80]
4	Ph	$ClCO_2Et$ (2)	7	93	86:0 [6.53]
5	^{Cy}Hex	NaCl (2)	3	31	24:0 [2.80]
6	^{Cy}Hex	NaCl (2)	6	52	38:0 [5.60]
7	^{Cy}Hex	$ClCO_2Et$ (2)	3	71	71:0 [2.80]
8	^{Cy}Hex	$ClCO_2Et$ (2)	6	>99	>89:>10 [5.60]
9[b]	^{Cy}Hex	Br_2 (1)	2	99	91:0 [5.60]
10[c]	^{Cy}Hex	I_2 (1)	4	5	5:0 [37.30]

[a] *Reagents and conditions*: Amide (0.2 mmol) dissolved in DMA (4 mL) in an undivided cell at rt. [b] 15 mA current. [c] 50 mA current. Conversions were determined by ^{1}H-NMR against 1,3,5-trimethoxybenzene as the internal standard and LC-MS following work-up; selectivity ratio in parentheses. Total Change in F.mol^{-1}.

Table S2c. Kinetic isotope effect [a]

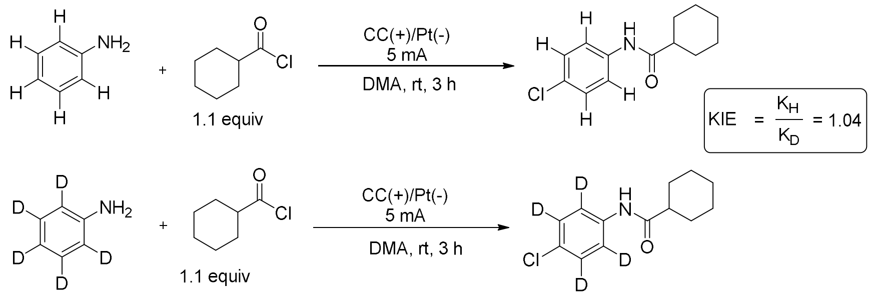

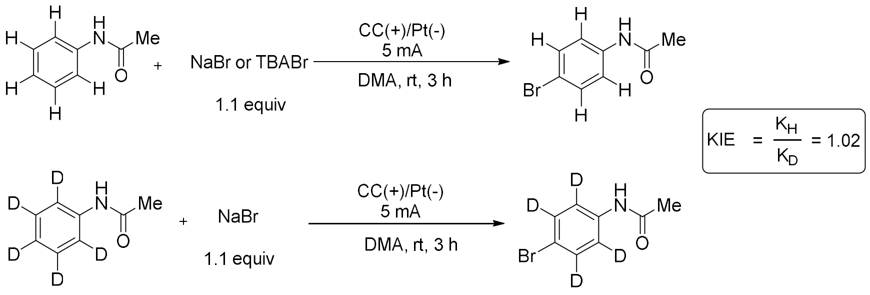

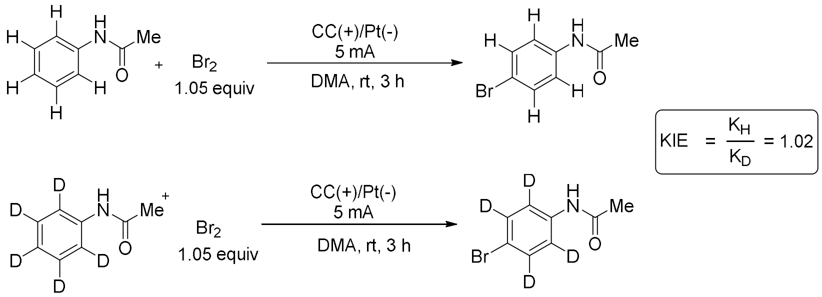

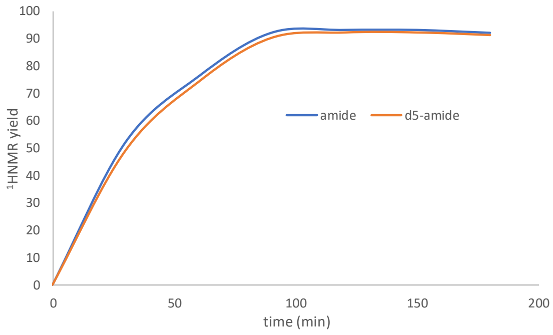

[a] *Reagents and conditions*: Amide (0.2 mmol) and brominating reagent (0.22 mmol) in DMA (4 mL) at rt using constant current of 5 mA for 3h. Reaction mixture were taken in every 30 minutes and analysed by LC-MS.

Table S2d. Effect of Bromine concentration on Bromination reaction [a]

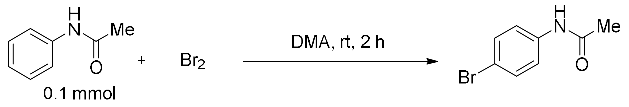

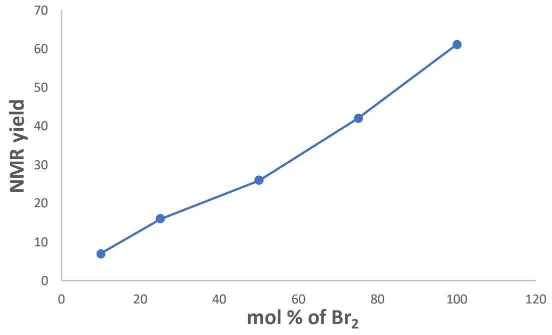

Reagents and conditions: 6 parallel reactions of amide with different concentrations of bromine (from 10, 25, 50, 75 and 100 mol%).

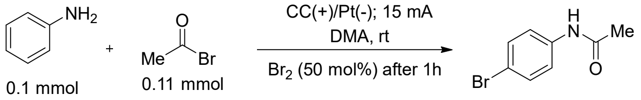

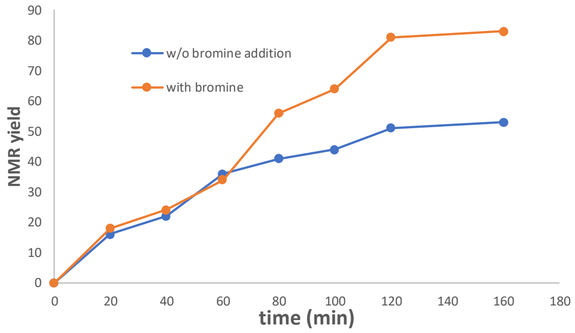

Reagents and conditions: (a) Bromine was added to one of the parallel reactions (after 1 hour) to evaluate the potential involvement of an in situ generated halogen species; (b) The reaction rate increased upon addition of 50 mol% Br_2.

General procedure A: Electrochemical cascade amidation/halogenation of various amines and acid halides [a]

The electrolysis was carried out in 10 mL microwave reaction vial in an undivided cell setup under air. A carbon cloth anode (15 mm × 10 mm × 410 µm) and a platinum (Pt) cathode (25 mm × 10 mm × 0.125 mm) with electrode holders made of stainless steel were used and the distance between two electrodes was 10 mm. The cell was charged with the amine (0.20 mmol, 1.0 equiv.) and a teflon-coated magnetic stirring bar (10 × 3 mm). 4 mL of DMA was added and the reaction mixture was stirred at 25 °C for 5 min to obtain a homogeneous solution, then acid chloride (0.21 mmol, 1.05 equiv.) was added. Subsequently, the electrolysis was performed at room temperature with a constant current of 5.0-15.0 mA maintained for 3 h (unless otherwise stated) with a stirring rate of 600 rpm. After

completion of the reaction, the reaction mixture was diluted with 2 mL ethyl acetate and transferred to a round bottom flask. The electrodes (carbon cloth and platinum) were washed in the reaction flask with ethyl acetate (3 × 5 mL) in an ultrasonic cleaner (3 × 3 min) and the washes were combined in the round bottom flask. The reaction mixture was then transferred to a separating funnel and washed with brine (15 mL). The aqueous layer was washed with ethyl acetate (3 x 15 mL) and the combined organic layer was washed with brine (5 x 30 mL). The organic phase was dried with sodium sulfate and the solvent was removed under vacuum to obtain crude product. The crude products were purified by flash column chromatography using isohexane and ethyl acetate mixture (20:1 to 3:1) to afford the title compounds.

Experimental Setup (Small scale):

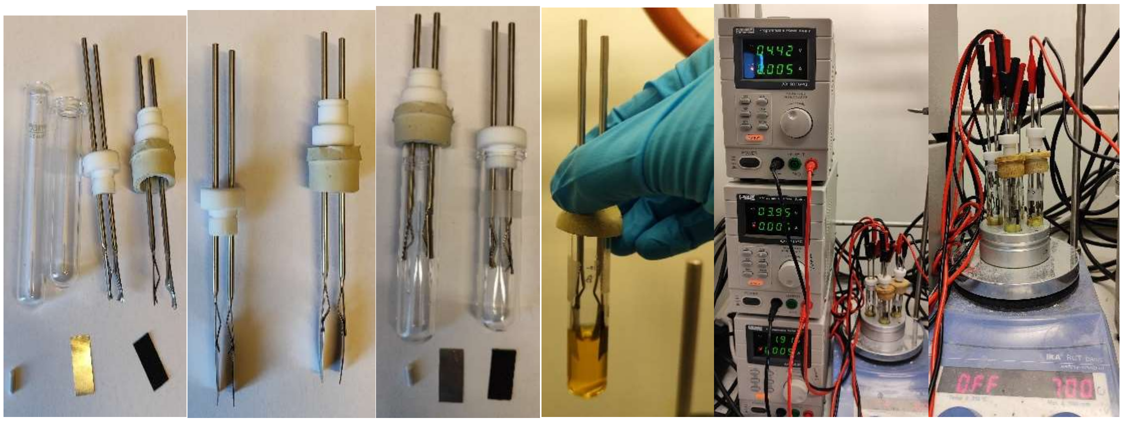

Figure S1. Images of the equipment and instrumentation used in this study, including vials, electrodes, electrode holders, and power supplies.

Scheme S1. Electrochemical cascade amidation/*para*-chlorination of various amines and acid halides [a]

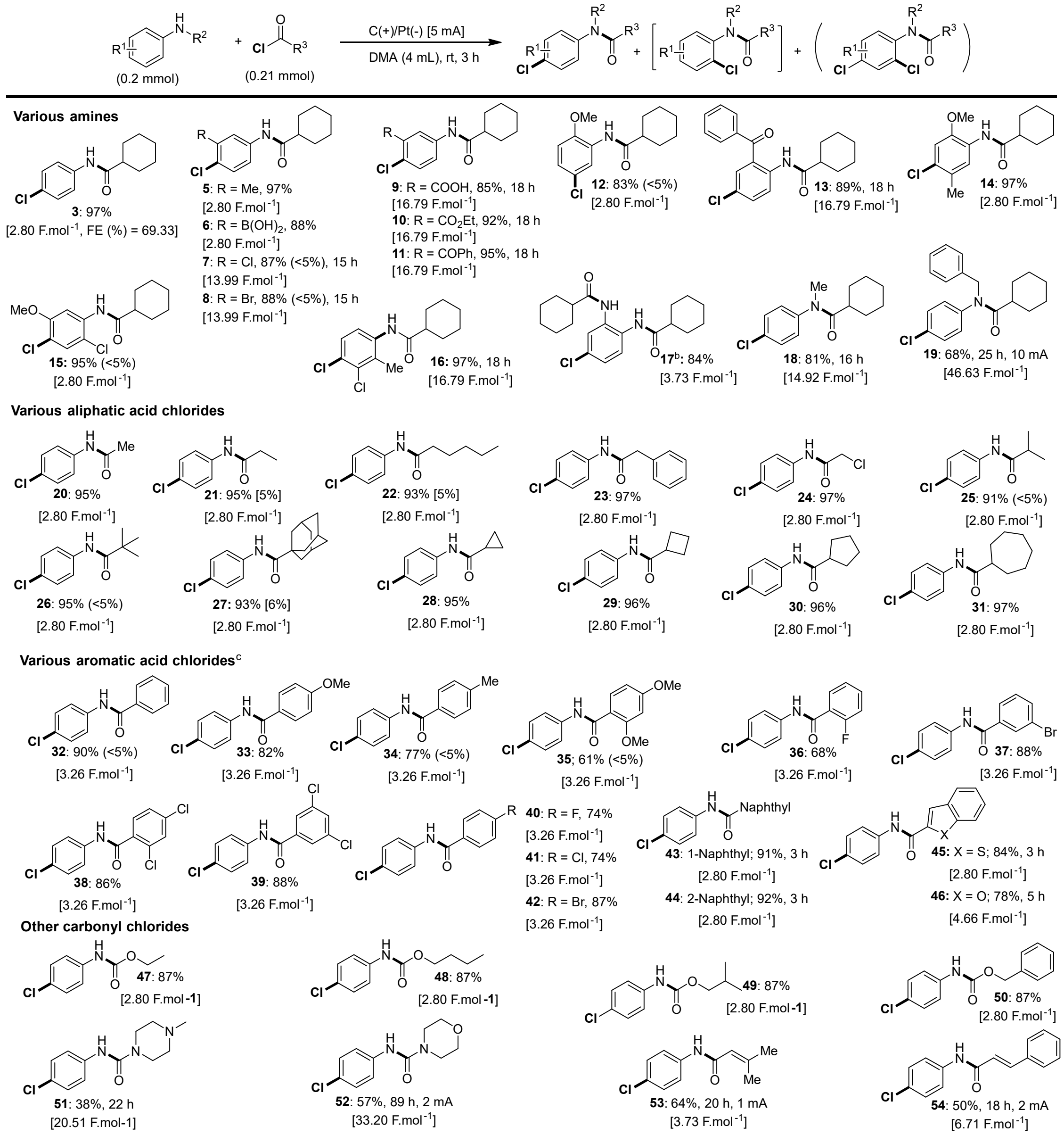

Reagents and conditions: [a] Standard experiment unless otherwise noted: Amine (0.2 mmol) and acid chloride (0.21 mmol) in DMA (4 mL), in an undivided cell with carbon cloth (anode) and platinum (cathode). [b] cyclohexanecarbonyl chloride (0.42 mmol), 4 h; [c] 3.5 h; All yields refer to isolated yields; mono-*ortho* chlorinated and di-chlorinated product in parenthesis.

Scheme S2. Electrochemical cascade amidation/*ortho*-chlorination of various amines and acid halides [a]

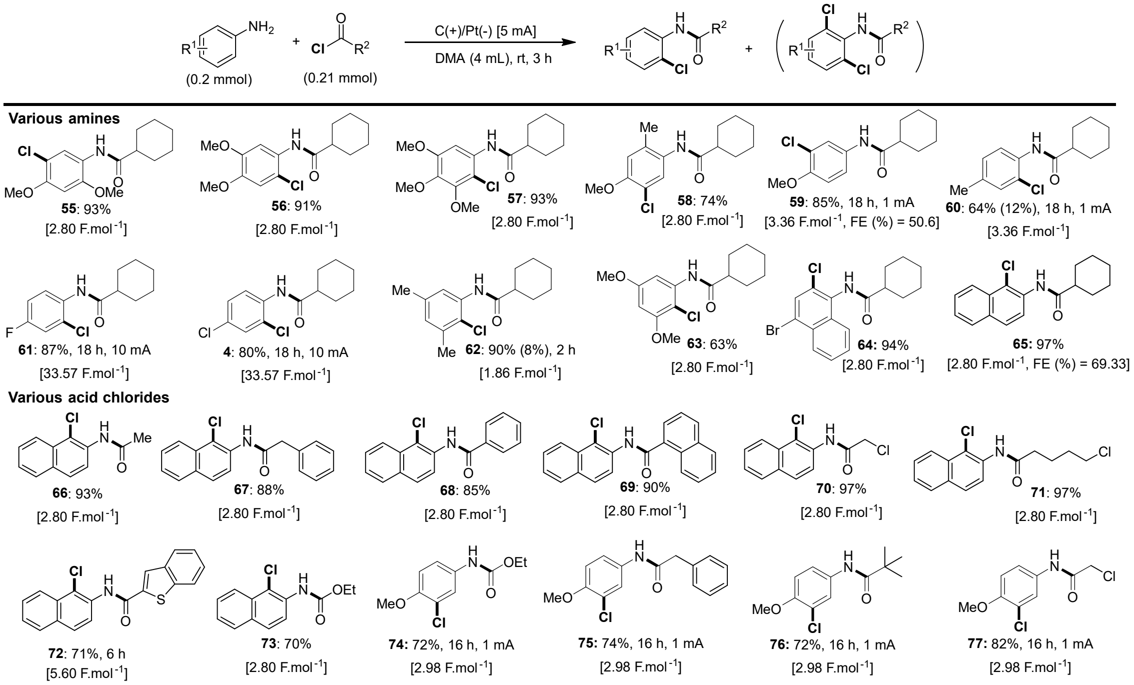

Reagents and conditions: [a] Standard experiment unless otherwise noted: Amine (0.2 mmol) and acid chloride (0.21 mmol) in DMA (4 mL), in an undivided cell with carbon cloth (anode) and platinum (cathode). All yields refer to isolated yields; mono-*ortho* chlorinated and di-chlorinated product in parenthesis.

Scheme S3. Electrochemical cascade amidation/bromination or iodination of various amines

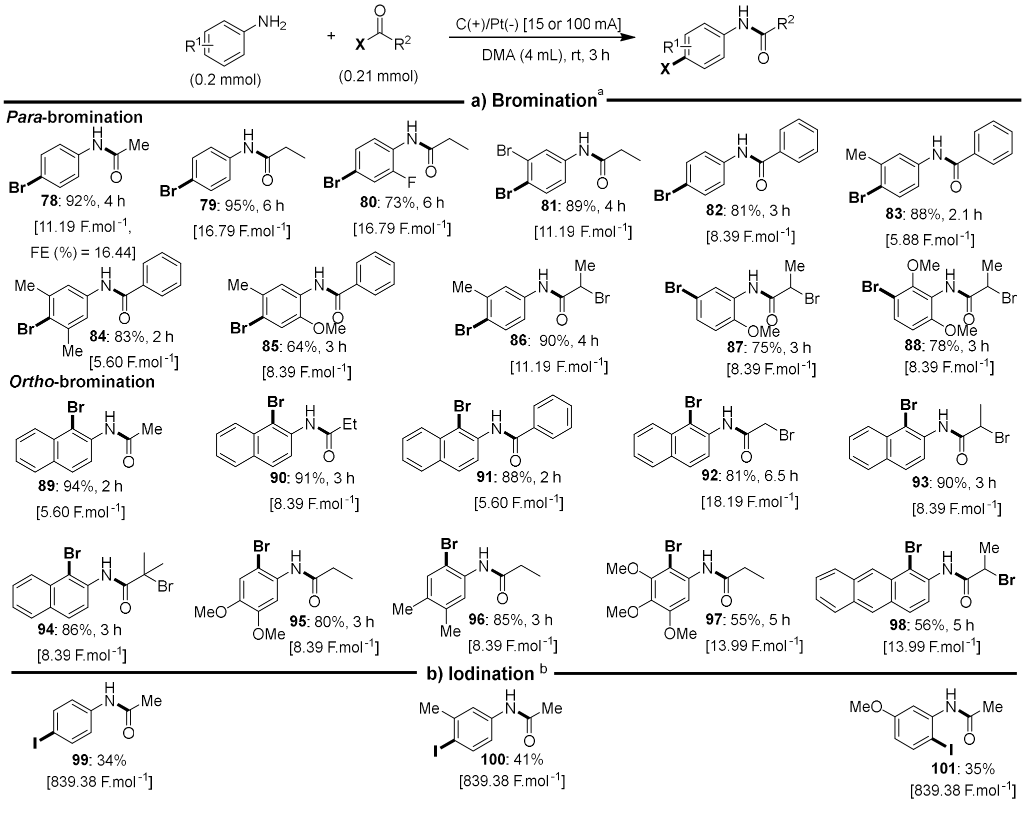

Reagents and conditions: [a] Amine (0.2 mmol) and acid halide (0.21 mmol) in DMA (4 mL), 15 mA, in an undivided cell with carbon cloth (anode) and platinum (cathode), yields refer to isolated yields. [b] Amine (0.2 mmol) and acid halide (0.21 mmol) in DMA (4 mL), in an undivided cell with carbon cloth (anode) and platinum (cathode), 100 mA, 45 h, yields determined by NMR.

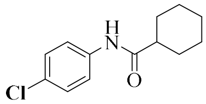

***N*-(4-chlorophenyl)cyclohexanecarboxamide (3)** was synthesized using 18.6 mg of aniline, 28 μL of cyclohexanecarbonyl chloride and following general procedure A using 5 mA constant current for 3 h. Compound **3** was isolated as a white solid (46 mg, 97% yield) following work-up without the need for any further purification. ^{1}H NMR (400 MHz, DMSO-d$_6$) δ 9.92 (s, 1H), 7.70 – 7.50 (m, 2H), 7.37 – 7.23 (m, 2H), 2.30 (tt, *J* = 11.8, 3.3 Hz, 1H), 1.77 (tt, *J* = 15.0, 2.2 Hz, 4H), 1.67 – 1.61 (m, 1H), 1.39 (qd, *J* = 13.5, 13.1, 3.5 Hz, 2H), 1.32 – 1.14 (m, 3H). ^{13}C NMR (101 MHz, DMSO-d$_6$) δ 174.4, 138.4, 128.5, 126.3, 120.5, 44.8, 29.1, 25.4, 25.2. HRMS (ESI): m/z [M+H]$^+$ calcd for $C_{13}H_{17}NOCl$: 238.0999; found: 238.0988.

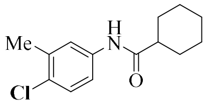

***N*-(4-chloro-3-methylphenyl)cyclohexanecarboxamide (5)** was synthesized using 21.4 mg of *m*-toluidine, 28.0 μL of cyclohexanecarbonyl chloride and following general procedure A using 5 mA constant current for 3 h. Compound **5** was isolated as a white solid (48.5 mg, 97% yield) following work-up without the need for any further purification. ^{1}H NMR (400 MHz, DMSO-d$_6$) δ 9.83 (s, 1H), 7.60 (d, *J* = 2.5 Hz, 1H), 7.44 (dd, *J* = 8.7, 2.6 Hz, 1H), 7.28 (d, *J* = 8.7 Hz, 1H), 2.31 (dt, *J* = 11.6, 3.2 Hz, 1H), 2.27 (s, 3H), 1.82 – 1.70 (m, 4H), 1.66 – 1.60 (m, 1H), 1.39 (qd, *J* = 13.7, 13.1, 3.5 Hz, 2H), 1.31 – 1.17 (m, 3H). ^{13}C NMR (101 MHz, DMSO-d$_6$) δ 174.3, 138.4, 135.4, 128.8, 126.6, 121.4, 118.2, 44.8, 29.1, 25.4, 25.2, 19.8. HRMS (ESI): m/z [M+H]$^+$ calcd for $C_{14}H_{19}NOCl$: 252.1155; found: 252.1147.

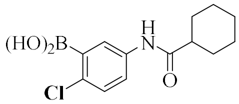

(2-Chloro-5-(cyclohexanecarboxamido)phenyl)boronic acid (6) was synthesized using 27.4 mg of (3-aminophenyl)boronic acid, 28.0 μL of cyclohexanecarbonyl chloride and following general procedure A using 5 mA constant current for 3 h. Compound **6** was isolated as a white solid (49.4 mg, 88% yield) following work-up and recrystallisation in ethyl acetate without the need for any further purification. ^{1}H NMR (400 MHz, DMSO-d$_6$) δ 9.83 (s, 1H), 8.27 (s, 2H), 7.63 (dd, *J* = 8.6, 2.7 Hz, 1H), 7.58 (d, *J* = 2.6 Hz, 1H), 7.24 (d, *J* = 8.7 Hz, 1H), 2.31 (ddt, *J* = 15.0, 11.7, 3.5 Hz, 1H), 1.80 – 1.73 (m, 4H), 1.64 (d, *J* = 11.0 Hz, 1H), 1.45 – 1.37 (m, 2H), 1.29 – 1.20 (m, 3H). ^{13}C NMR

(101 MHz, DMSO-d_6) δ 174.3, 137.6, 129.3, 128.4 (2C), 123.9, 120.6, 44.8, 29.1, 25.4, 25.2. HRMS (ESI): m/z $[M+H]^+$ calcd for $C_{13}H_{18}BNO_3Cl$: 282.1068; found: 282.1070.

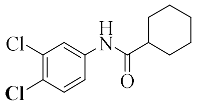

***N*-(3,4-dichlorophenyl)cyclohexanecarboxamide (7)** was synthesized using 25.4 mg of 3-chloroaniline, 28.0 μL of cyclohexanecarbonyl chloride and following general procedure A using 5 mA constant current for 15 h. Compound **7** was isolated using automated flash chromatography (silica gel, gradient elution 5-30% EtOAc in isohexane) as a white solid (47 mg, 87% yield). ^1H NMR (400 MHz, $CDCl_3$) δ 8.38 (d, *J* = 8.9 Hz, 1H), 7.64 (s, 1H), 7.37 (d, *J* = 2.4 Hz, 1H), 7.24 (dd, *J* = 8.9, 2.4 Hz, 1H), 2.30 (tt, *J* = 11.7, 3.6 Hz, 1H), 2.03 – 1.97 (m, 2H), 1.85 (dt, *J* = 13.1, 3.6 Hz, 2H), 1.72 (dddt, *J* = 9.5, 4.8, 3.0, 1.5 Hz, 1H), 1.58 – 1.48 (m, 2H), 1.37 – 1.24 (m, 3H). ^{13}C NMR (101 MHz, $CDCl_3$) δ 174.4, 133.6, 128.9, 128.7, 128.0, 123.2, 122.4, 46.7, 29.8, 25.8, 25.7.

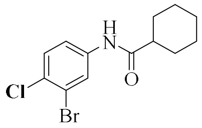

***N*-(3-bromo-4-chlorophenyl)cyclohexanecarboxamide (8)** was synthesized using 34.0 mg of 3-bromoaniline, 28.0 μL of cyclohexanecarbonyl chloride and following general procedure A using 5 mA constant current for 15 h. **8** was isolated using automated flash chromatography (silica gel, gradient elution 5-30% EtOAc in isohexane) as a white solid (55.5 mg, 88% yield). ^1H NMR (400 MHz, $CDCl_3$) δ 7.92 (d, *J* = 2.4 Hz, 1H), 7.41 (dd, *J* = 8.7, 2.4 Hz, 1H), 7.35 (d, *J* = 8.7 Hz, 1H), 7.20 (s, 1H), 2.21 (tt, *J* = 11.7, 3.5 Hz, 1H), 1.97 – 1.90 (m, 2H), 1.87 – 1.81 (m, 2H). 1.74 – 1.67 (m, 1H), 1.54 (td, *J* = 13.7, 12.4, 5.0 Hz, 2H), 1.30 (ddt, *J* = 16.8, 8.6, 2.9 Hz, 3H). ^{13}C NMR (101 MHz, $CDCl_3$) δ 174.6, 137.6, 130.4, 129.3, 124.7, 122.6, 119.8, 46.6, 29.7, 25.7, 25.7. HRMS (ESI): m/z $[M+H]^+$ calcd for $C_{13}H_{16}NOClBr$: 316.0104; found: 316.0090.

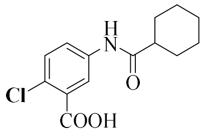

2-Chloro-5-(cyclohexanecarboxamido)benzoic acid (9) was synthesized using 27.4 mg of 3-aminobenzoic acid, 28.0 μL of cyclohexanecarbonyl chloride and following general procedure A using 5 mA constant current for 18 h. **9** was isolated as a white solid (47.5 mg, 85% yield) by recrystallisation in ethyl acetate without the need for any further purification.. ^1H NMR (400 MHz, DMSO-d_6) δ 13.35 (s, 1H), 10.07 (s, 1H), 8.11 (d, *J* = 2.6 Hz, 1H), 7.73 (dd, *J* = 8.7, 2.7 Hz, 1H), 7.44 (d, *J* = 8.7 Hz, 1H), 2.31 (tt, *J* = 11.6, 3.4 Hz, 1H), 1.83 – 1.71 (m, 4H), 1.64 (d, *J* = 10.8 Hz, 1H), 1.45 – 1.34 (m, 2H), 1.31-1.18 (m, 3H). ^{13}C NMR (101 MHz, DMSO-d_6) δ 174.7, 166.5, 138.4, 131.2, 130.9, 124.9, 122.7, 121.0, 44.9, 29.0, 25.3, 25.2.

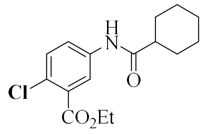

Ethyl 2-chloro-5-(cyclohexanecarboxamido)benzoate (10) was synthesized using 33.0 mg of ethyl 3-aminobenzoate, 28.0 μL of cyclohexanecarbonyl chloride and following general procedure A using 5 mA constant current for 18 h. **10** was isolated using automated flash chromatography (silica gel, gradient elution 5-30% EtOAc in isohexane) as a white solid (56.5 mg, 92% yield). ^{1}H NMR (400 MHz, $CDCl_3$) δ 7.93 (d, *J* = 2.7 Hz, 1H), 7.71 (dd, *J* = 8.7, 2.7 Hz, 1H), 7.65 (s, 1H), 7.34 (d, *J* = 8.7 Hz, 1H), 4.36 (q, *J* = 7.1 Hz, 2H), 2.22 (tt, *J* = 11.7, 3.5 Hz, 1H), 1.94 – 1.87 (m, 2H), 1.84 – 1.77 (m, 2H), 1.70 – 1.64 (m, 1H), 1.53 (td, *J* = 12.1, 3.5 Hz, 2H), 1.37 (t, *J* = 7.1 Hz, 3H), 1.31 – 1.21 (m, 3H). ^{13}C NMR (101 MHz, $CDCl_3$) δ 174.9, 165.6, 137.0, 131.5, 130.7, 128.1, 123.8, 122.2, 61.8, 46.5, 29.7, 25.7, 25.7, 14.3. HRMS (ESI): m/z $[M+H]^+$ calcd for $C_{16}H_{21}NO_3Cl$: 310.1210; found: 310.1212.

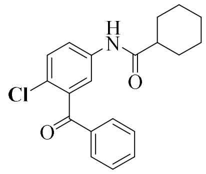

***N*-(3-benzoyl-4-chlorophenyl)cyclohexanecarboxamide (11)** was synthesized using 39.4 mg of (3-aminophenyl)(phenyl)methanone, 28.0 μL of cyclohexanecarbonyl chloride and following general procedure A using 5 mA constant current for 18 h. **11** was isolated using automated flash chromatography (silica gel, gradient elution 5-30% EtOAc in isohexane) as a colorless sticky solid (64.5 mg, 95% yield). ^{1}H NMR (400 MHz, $CDCl_3$) δ 7.84 – 7.80 (m, 2H), 7.72 (dd, *J* = 8.7, 2.6 Hz, 1H), 7.63 – 7.58 (m, 1H), 7.50 – 7.44 (m, 3H), 7.39 (d, *J* = 8.8 Hz, 1H), 7.25 (s, 1H), 2.22 (tt, *J* = 11.7, 3.5 Hz, 1H), 1.94 (dd, *J* = 13.0, 3.5 Hz, 2H), 1.84 (dq, *J* = 9.6, 3.2 Hz, 2H), 1.71 (d, *J* = 3.1 Hz, 1H), 1.57 – 1.50 (m, 2H), 1.35 – 1.27 (m, 3H). ^{13}C NMR (101 MHz, $CDCl_3$) δ 195.0, 174.6, 139.1, 137.0, 136.4, 134.0, 130.7, 130.3, 128.8, 125.9, 122.4, 120.1, 46.6, 29.7, 25.7, 25.7. HRMS (ESI): m/z $[M+H]^+$ calcd for $C_{20}H_{21}NO_2Cl$: 342.1261; found: 342.1265.

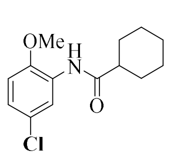

***N*-(5-chloro-2-methoxyphenyl)cyclohexanecarboxamide (12)** was synthesized using 24.6 mg of 2-methoxyaniline, 28.0 μL of cyclohexanecarbonyl chloride and following general procedure A using 5 mA constant current for 3 h. **12** was isolated using automated flash chromatography (silica gel, gradient elution 5-30% EtOAc in isohexane) as a white solid (44.0 mg, 83% yield). ^{1}H NMR (400 MHz, $CDCl_3$) δ 8.35 (d, *J* = 8.7 Hz, 1H), 7.72 (s, 1H), 6.93 (dd, *J* = 8.7, 2.2 Hz, 1H), 6.85 (d, *J* = 2.2 Hz, 1H), 3.89 (s, 3H), 2.26 (tt, *J* = 11.7, 3.5 Hz, 1H), 1.99 – 1.93 (m, 2H), 1.87 – 1.81 (m, 2H), 1.74 – 1.68 (m, 1H), 1.58

– 1.47 (m, 2H), 1.35 – 1.25 (m, 3H). ^{13}C NMR (101 MHz, $CDCl_3$) δ 174.3, 148.4, 128.3, 126.7, 121.1, 120.5, 110.7, 56.1, 46.9, 29.8, 25.8, 25.8. HRMS (ESI): m/z $[M+H]^+$ calcd for $C_{14}H_{19}NO_2Cl$: 268.1104; found: 268.1093.

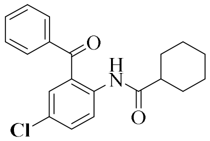

***N*-(2-benzoyl-4-chlorophenyl)cyclohexanecarboxamide (13)** was synthesized using 39.4 mg of (2-aminophenyl)(phenyl)methanone, 28.0 µL of cyclohexanecarbonyl chloride and following general procedure A using 5 mA constant current for 18 h. **13** was isolated using automated flash chromatography (silica gel, gradient elution 5-30% EtOAc in isohexane) as a white solid (60.5 mg, 89% yield). ^{1}H NMR (400 MHz, $CDCl_3$) δ 10.77 (s, 1H), 8.68 (d, *J* = 9.7 Hz, 1H), 7.72 – 7.68 (m, 2H), 7.65 – 7.60 (m, 1H), 7.52 (ddd, *J* = 7.2, 4.6, 2.6 Hz, 4H), 2.31 (tt, *J* = 11.7, 3.5 Hz, 1H), 2.06 – 1.98 (m, 2H), 1.83 (dt, *J* = 11.7, 3.0 Hz, 2H), 1.73 – 1.66 (m, 1H), 1.53 (qd, *J* = 12.1, 3.3 Hz, 2H), 1.38 – 1.22 (m, 3H). ^{13}C NMR (101 MHz, $CDCl_3$) δ 198.8, 175.5, 139.5, 138.2, 134.2, 133.0, 132.9, 130.0, 128.7, 127.1, 124.5, 123.2, 47.1, 29.7, 25.8 (2C). HRMS (ESI): m/z $[M+H]^+$ calcd for $C_{20}H_{21}NO_2Cl$: 342.1261; found: 342.1265.

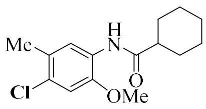

***N*-(4-chloro-2-methoxy-5-methylphenyl)cyclohexanecarboxamide (14)** was synthesized using 27.4 mg of 2-methoxy-5-methylaniline, 28.0 µL of cyclohexanecarbonyl chloride and following general procedure A using 5 mA constant current for 3 h. Compound **14** was isolated as a white solid (54.5 mg, 97% yield) following work-up without the need for any further purification. ^{1}H NMR (400 MHz, $CDCl_3$) δ 8.33 (s, 1H), 7.69 (s, 1H), 6.84 (s, 1H), 3.86 (s, 3H), 2.29 (d, *J* = 0.6 Hz, 3H), 2.24 (dt, *J* = 11.7, 3.5 Hz, 1H), 1.99 – 1.93 (m, 2H), 1.84 (dt, *J* = 12.6, 3.4 Hz, 2H), 1.71 (ddd, *J* = 12.0, 3.7, 1.9 Hz, 1H), 1.59 – 1.47 (m, 3H), 1.36 – 1.28 (m, 2H). ^{13}C NMR (101 MHz, $CDCl_3$) δ 174.3, 146.6, 128.3, 127.9, 126.4, 121.7, 110.9, 56.1, 46.8, 29.8, 25.8, 25.8, 19.5. HRMS (ESI): m/z $[M+H]^+$ calcd for $C_{15}H_{21}NO_2Cl$: 282.1261; found: 282.1247.

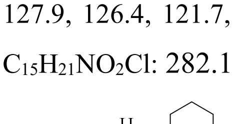

***N*-(2,4-dichloro-5-methoxyphenyl)cyclohexanecarboxamide (15)** was synthesized using 31.4 mg of 2-chloro-5-methoxyaniline, 28.0 µL of cyclohexanecarbonyl chloride and following general procedure A using 5 mA constant current for 3 h. Compound **15** was isolated as a white solid (57.0 mg, 95% yield) following work-up without the need for any further purification. ^{1}H NMR (400 MHz, $CDCl_3$) δ 8.30 (d, *J* = 1.5 Hz, 1H), 7.74 – 7.62 (m, 1H), 7.35 (s, 1H), 3.91 (s, 3H), 2.31 (tt, *J* = 11.6, 3.5 Hz, 1H), 2.06

– 1.96 (m, 2H), 1.91 – 1.81 (m, 2H), 1.73 (dtd, J = 10.5, 3.2, 1.5 Hz, 1H), 1.64 – 1.45 (m, 2H), 1.41 – 1.20 (m, 3H). ^{13}C NMR (101 MHz, $CDCl_3$) δ 174.6, 154.4, 134.4, 129.3, 117.0, 113.3, 105.0, 56.5, 46.81, 29.7, 25.7, 25.7. HRMS (ESI): m/z $[M+H]^+$ calcd for $C_{14}H_{18}NO_2Cl_2$: 302.0715; found: 302.0719.

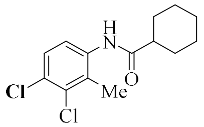

***N*-(3,4-dichloro-2-methylphenyl)cyclohexanecarboxamide (16)** was synthesized using 28.2 mg of 3-chloro-2-methylaniline, 28.0 μL of cyclohexanecarbonyl chloride and following general procedure A using 5 mA constant current for 18 h. Compound **16** was isolated as a white solid (55.3 mg, 97% yield) following work-up without the need for any further purification. ^{1}H NMR (400 MHz, DMSO-d_6) δ 9.50 (s, 1H), 7.45 (d, J = 8.6 Hz, 1H), 7.31 (d, J = 8.6 Hz, 1H), 2.40 (tt, J = 11.6, 3.5 Hz, 1H), 2.25 (s, 3H), 1.86 – 1.80 (m, 2H), 1.79 – 1.73 (m, 2H), 1.68 – 1.62 (m, 1H), 1.42 (qd, J = 12.2, 3.0 Hz, 2H), 1.34 – 1.17 (m, 3H). ^{13}C NMR (101 MHz, DMSO-d_6) δ 174.4, 136.6, 133.1, 131.5, 128.0, 127.1, 125.6, 44.1, 29.2, 25.4, 25.2, 16.3. HRMS (ESI): m/z $[M+H]^+$ calcd for $C_{14}H_{18}NOCl_2$: 286.0765; found: 286.0755.

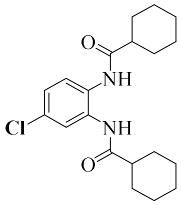

***N,N'*-(4-chloro-1,2-phenylene)dicyclohexanecarboxamide (17)** was synthesized using 21.6 mg of benzene-1,2-diamine, 56.0 μL of cyclohexanecarbonyl chloride and following general procedure A using 5 mA constant current for 4 h. Compound **17** was isolated using automated flash chromatography (silica gel, gradient elution 5-30% EtOAc in isohexane) as a pale grey solid (60.5 mg, 84% yield). ^{1}H NMR (400 MHz, $CDCl_3$) δ 8.46 (s, 1H), 8.32 (s, 1H), 7.23 (d, J = 2.3 Hz, 1H), 7.17 (d, J = 8.6 Hz, 1H), 7.07 (dd, J = 8.6, 2.3 Hz, 1H), 2.19 (qd, J = 10.1, 8.6, 6.5 Hz, 2H), 1.90 – 1.80 (m, 8H), 1.74 – 1.68 (m, 2H), 1.44 (tdt, J = 9.1, 6.0, 3.8 Hz, 4H), 1.36 – 1.21 (m, 6H). ^{13}C NMR (101 MHz, $CDCl_3$) δ 176.4, 176.4, 132.2, 131.2, 129.6, 127.2, 126.0, 125.6, 45.8, 45.8, 29.7, 29.7, 25.8, 25.8, 25.7 (2C). HRMS (ESI): m/z $[M+H]^+$ calcd for $C_{20}H_{28}N_2O_2Cl$: 363.1839; found: 363.1841.

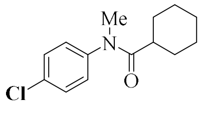

***N*-(4-chlorophenyl)-*N*-methylcyclohexanecarboxamide (18)** was synthesized using 21.4 mg of *N*-methylaniline, 28.0 μL of cyclohexanecarbonyl chloride and following general procedure A using 5 mA constant current for 16 h. Compound **18** was isolated using automated flash chromatography (silica gel, gradient elution 5-30% EtOAc in isohexane) as a colorless sticky solid (40.5 mg, 81% yield). ^{1}H NMR (400 MHz, $CDCl_3$) δ 7.44 – 7.35 (m, 2H), 7.16 – 7.04 (m, 2H), 3.21 (s, 3H), 2.26 – 2.08 (m, 1H),

1.70 – 1.45 (m, 7H), 1.26 – 1.09 (m, 1H), 1.07 – 0.89 (m, 2H). ^{13}C NMR (101 MHz, $CDCl_3$) δ 176.4, 143.0, 130.1 (2C), 128.8, 41.5, 37.6, 29.6, 25.7, 25.6. HRMS (ESI): m/z $[M+H]^+$ calcd for $C_{14}H_{19}NOCl$: 252.1155; found: 252.1156.

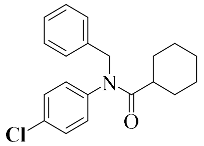

***N*-benzyl-*N*-(4-chlorophenyl)cyclohexanecarboxamide (19)** was synthesized using 36.6 mg of *N*-benzylaniline, 28.0 μL of cyclohexanecarbonyl chloride and following general procedure A using 10 mA constant current for 25 h. Compound **19** was isolated using automated flash chromatography (silica gel, gradient elution 5-30% EtOAc in isohexane) as a white solid (44.5 mg, 68% yield). ^{1}H NMR (400 MHz, $CDCl_3$) δ 7.30 – 7.24 (m, 5H), 7.14 (dd, *J* = 7.4, 2.1 Hz, 2H), 6.88 (d, *J* = 8.2 Hz, 2H), 4.83 (s, 2H), 2.10 (td, *J* = 11.0, 5.6 Hz, 1H), 1.63 (dddd, *J* = 31.5, 15.5, 7.9, 3.4 Hz, 6H), 1.27 – 1.17 (m, 2H), 0.99 (tt, *J* = 12.9, 3.7 Hz, 2H). ^{13}C NMR (101 MHz, $CDCl_3$) δ 176.2, 141.1, 137.6, 133.8, 129.8, 129.8, 128.9, 128.6, 127.5, 52.9, 41.8, 29.6, 25.7, 25.6. HRMS (ESI): m/z $[M+H]^+$ calcd for $C_{20}H_{23}NOCl$: 328.1468; found: 328.1465.

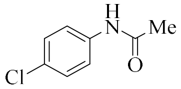

***N*-(4-chlorophenyl)acetamide (20)** was synthesized using 18.6 mg of aniline, 15 μL of acetyl chloride and following general procedure A using 5 mA constant current for 3 h. Compound **20** was obtained as a pale brown solid (32 mg, 95% yield) following work-up without the need for any further purification. ^{1}H NMR (400 MHz, $CDCl_3$) δ 7.54 – 7.35 (m, 2H), 7.34 – 7.17 (m, 3H), 2.17 (s, 3H). ^{13}C NMR (101 MHz, $CDCl_3$) δ 168.4, 136.6, 129.4, 129.2, 121.2, 24.7.

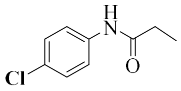

***N*-(4-chlorophenyl)propionamide (21)** was synthesized using 18.6 mg of aniline, 18.5 μL of propionyl chloride and following general procedure A using 5 mA constant current for 3 h. Compound **21** was obtained as a white solid (34.5 mg, 95% yield) following work-up without the need for any further purification. ^{1}H NMR (400 MHz, $CDCl_3$) δ 7.42 – 7.38 (m, 2H), 7.23 – 7.18 (m, 3H), 2.31 (q, *J* = 7.6 Hz, 2H), 1.17 (t, *J* = 7.5 Hz, 3H).^{13}C NMR (101 MHz, $CDCl_3$) δ 172.2, 136.7, 129.2, 129.1, 121.1, 30.8, 9.7.

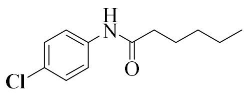

***N*-(4-chlorophenyl)hexanamide (22)** was synthesized using 18.6 mg of aniline, 29.5 μL of hexanoyl chloride and following general procedure A using 5 mA constant current for 3 h. Compound **22** was isolated using automated flash chromatography (silica gel, gradient elution 5-30% EtOAc in isohexane) as a white solid (41.5 mg, 93% yield). ^{1}H NMR (400 MHz, $CDCl_3$) δ 7.46 (d, *J* = 8.8 Hz, 2H), 7.34 (s, 1H), 7.26 (dt, *J* = 6.6, 2.3 Hz, 2H), 2.34 (t, *J* = 7.6 Hz, 2H), 1.76 – 1.66 (m, 2H), 1.34 (td, *J* = 7.2, 6.2, 3.6 Hz, 4H), 0.93 – 0.86 (m, 3H). ^{13}C NMR (101 MHz, $CDCl_3$) δ 171.6, 136.7, 129.2, 129.1, 121.2, 37.9, 31.5, 25.4, 22.5, 14.1. HRMS (ESI): m/z $[M+H]^+$ calcd for $C_{12}H_{17}NOCl$: 226.0999; found: 226.0992.

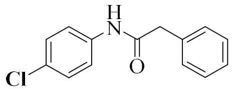

***N*-(4-chlorophenyl)-2-phenylacetamide (23)** was synthesized using 18.6 mg of aniline, 28.0 μL of phenylacetyl chloride and following general procedure A using 5 mA constant current for 3 h. Compound **23** was isolated using automated flash chromatography (silica gel, gradient elution 5-30% EtOAc in isohexane) as a white solid (47.5 mg, 97% yield). ^{1}H NMR (400 MHz, $CDCl_3$) δ 7.44 – 7.31 (m, 7H), 7.26 – 7.21 (m, 2H), 7.01 (s, 1H), 3.74 (s, 2H). ^{13}C NMR (101 MHz, $CDCl_3$) δ 169.1, 136.3, 134.3, 129.7, 129.5, 129.1, 128.0, 121.1, 119.9, 45.0.

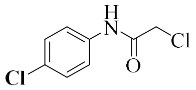

2-chloro-*N*-(4-chlorophenyl)acetamide (24) was synthesized using 18.6 mg of aniline, 17 μL of 2-chloroacetyl chloride and following general procedure A using 5 mA constant current for 3 h. Compound **24** was obtained as a white solid (39.5 mg, 97% yield) following work-up without the need for any further purification. ^{1}H NMR (400 MHz, $CDCl_3$) δ 8.22 (s, 1H), 7.51 (d, *J* = 8.9 Hz, 2H), 7.33 (d, *J* = 8.8 Hz, 2H), 4.19 (s, 2H). ^{13}C NMR (101 MHz, $CDCl_3$) δ 163.9, 135.4, 130.5, 129.3, 121.5, 43.0.

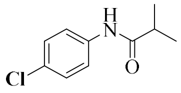

***N*-(4-chlorophenyl)isobutyramide (25)** was synthesized using 18.6 mg of aniline, 22 μL of isobutyryl chloride and following general procedure A using 5 mA constant current for 3 h. Compound **25** was isolated using automated flash chromatography (silica gel, gradient elution 10-30% EtOAc in isohexane) as a white solid (36 mg, 91% yield). ^{1}H NMR (400 MHz, $CDCl_3$) δ 7.50 – 7.45 (m, 2H), 7.32 (s, 1H), 7.28 – 7.23 (m, 2H), 2.50 (p, *J* = 6.9 Hz, 1H), 1.24 (d, *J* = 6.9 Hz, 6H). ^{13}C NMR (101

MHz, $CDCl_3$) δ 175.5, 136.7, 129.2, 129.1, 121.2, 36.8, 19.7. HRMS (ESI): m/z $[M+H]^+$ calcd. for $C_{10}H_{13}NOCl$: 198.0686; found: 198.0676.

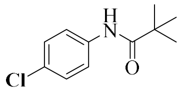

***N*-(4-chlorophenyl)pivalamide (26)** was synthesized using 18.6 mg of aniline, 22 μL of pivaloyl chloride and following general procedure A using 5 mA constant current for 3 h. Compound **26** was isolated using automated flash chromatography (silica gel, gradient elution 10-30% EtOAc in isohexane) as a white solid (40 mg, 95% yield). ^1H NMR (400 MHz, $CDCl_3$) δ 7.48 (d, *J* = 8.9 Hz, 2H), 7.28 (d, *J* = 8.9 Hz, 2H), 7.26 (s, 1H), 1.31 (s, 9H). ^{13}C NMR (101 MHz, $CDCl_3$) δ 176.7, 136.7, 129.3, 129.1, 121.3, 39.8, 27.7. HRMS (ESI): m/z $[M+H]^+$ calcd for $C_{11}H_{15}NOCl$: 212.0842; found: 212.0842.

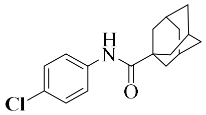

***N*-(4-chlorophenyl)adamantane-1-carboxamide (27)** was synthesized using 18.6 mg of aniline, 42 mg of (1*S*,3*R*,5*S*)-adamantane-1-carbonyl chloride and following general procedure A using 5 mA constant current for 3 h. Compound **27** was isolated using automated flash chromatography (silica gel, gradient elution 10-30% EtOAc in isohexane) as a white solid (53.5 mg, 93% yield). ^1H NMR (400 MHz, $CDCl_3$) δ 7.59 – 7.42 (m, 2H), 7.30 (td, *J* = 7.0, 6.6, 3.3 Hz, 3H), 2.14 – 2.10 (m, 3H), 2.01 (d, *J* = 3.0 Hz, 1H), 1.98 (d, *J* = 2.9 Hz, 4H), 1.84 – 1.72 (m, 7H). ^{13}C NMR (101 MHz, $CDCl_3$) δ 176.2, 136.8, 129.1, 129.0, 121.3, 41.7, 39.4, 39.1, 36.5, 36.2, 28.2, 28.0. HRMS (ESI): m/z $[M+H]^+$ calcd for $C_{17}H_{21}NOCl$: 290.1312; found: 290.1305.

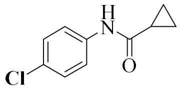

***N*-(4-chlorophenyl)cyclopropanecarboxamide (28)** was synthesized using 18.6 mg of aniline, 17 μL of cyclopropanecarbonyl chloride and following general procedure A using 5 mA constant current for 3 h. Compound **28** was isolated as a white solid (37.0 mg, 95% yield) following work-up without the need for any further purification. ^1H NMR (400 MHz, DMSO-d_6) δ 10.33 (s, 1H), 7.61 (d, *J* = 8.9 Hz, 2H), 7.33 (d, *J* = 8.9 Hz, 2H), 1.76 (tt, *J* = 7.0, 5.5 Hz, 1H), 0.85 – 0.64 (m, 4H). ^{13}C NMR (101 MHz, DMSO-d_6) δ 171.8, 138.3, 128.6, 126.4, 120.5, 14.5, 7.3. HRMS (ESI): m/z $[M+H]^+$ calcd for $C_{10}H_{11}NOCl$: 196.0529; found: 196.0523.

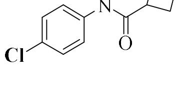

***N*-(4-chlorophenyl)cyclobutanecarboxamide (29)** was synthesized using 18.6 mg of aniline, 24 μL of cyclobutanecarbonyl chloride and following general procedure A using 5 mA constant current for 3h. Compound **29** was isolated as a white solid (40.0 mg, 96% yield) following work-up without the

need for any further purification. ^{1}H NMR (400 MHz, $CDCl_3$) δ 7.52 – 7.42 (m, 2H), 7.31 – 7.23 (m, 2H), 7.17 (s, 1H), 3.14 (pd, *J* = 8.5, 1.0 Hz, 1H), 2.43 – 2.32 (m, 2H), 2.27 – 2.16 (m, 2H), 2.06 – 1.87 (m, 2H). ^{13}C NMR (101 MHz, $CDCl_3$) δ 173.4, 136.7, 129.1, 129.1, 121.0, 40.9, 25.4, 18.2. HRMS (ESI): m/z $[M+H]^+$ calcd for $C_{11}H_{13}NOCl$: 210.0686; found: 210.0688.

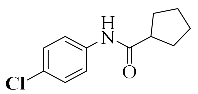

***N*-(4-chlorophenyl)cyclopentanecarboxamide (30)** was synthesized using 18.6 mg of aniline, 25.5 μL of cyclopentanecarbonyl chloride and following general procedure A using 5 mA constant current for 3 h. Compound **30** was isolated as a white solid (43.0 mg, 96% yield) following work-up without the need for any further purification. ^{1}H NMR (400 MHz, $CDCl_3$) δ 7.50 – 7.46 (m, 2H), 7.28 (d, *J* = 2.2 Hz, 2H), 7.13 (s, 1H), 2.66 (p, *J* = 8.0 Hz, 1H), 1.96 – 1.86 (m, 4H), 1.82 – 1.76 (m, 2H), 1.62 (dp, *J* = 6.9, 3.5, 3.1 Hz, 2H). ^{13}C NMR (101 MHz, $CDCl_3$) δ 174.9, 136.9, 129.1, 129.0, 121.2, 46.9, 30.6, 26.1. HRMS (ESI): m/z $[M-H]^-$ calcd for $C_{12}H_{13}NOCl$: 222.0691; found: 222.0689.

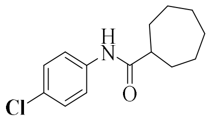

***N*-(4-chlorophenyl)cycloheptanecarboxamide (31)** was synthesized using 18.6 mg of aniline, 31.0 μL of cycloheptanecarbonyl chloride and following general procedure A using 5 mA constant current for 3 h. Compound **31** was isolated as an off-white solid (48.5 mg, 97% yield) following work-up without the need for any further purification. ^{1}H NMR (400 MHz, $CDCl_3$) δ 7.49 – 7.44 (m, 2H), 7.29 – 7.23 (m, 3H), 2.36 (tt, *J* = 9.7, 4.0 Hz, 1H), 1.96 (ddd, *J* = 13.9, 6.8, 3.8 Hz, 2H), 1.83 – 1.69 (m, 4H), 1.62 – 1.43 (m, 6H). ^{13}C NMR (101 MHz, $CDCl_3$) δ 175.7, 136.9, 129.1, 129.1, 121.2, 48.5, 31.7, 28.3, 26.6. HRMS (ESI): m/z $[M+H]^+$ calcd for $C_{14}H_{19}NOCl$: 252.1155; found: 252.1149.

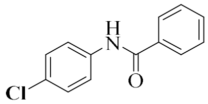

***N*-(4-chlorophenyl)benzamide (32)** was synthesized using 18.6 mg of aniline, 24.5 μL of benzoyl chloride and following general procedure A using 5 mA constant current for 3.5 h. Compound **32** was isolated using automated flash chromatography (silica gel, gradient elution 5-30% EtOAc in isohexane) as a white solid (41.5 mg, 90% yield). ^{1}H NMR (400 MHz, DMSO-d_6) δ 10.37 (s, 1H), 7.96 – 7.93 (m, 2H), 7.84 – 7.80 (m, 2H), 7.62 – 7.58 (m, 1H), 7.56 – 7.51 (m, 2H), 7.43 – 7.39 (m, 2H). ^{13}C NMR (101 MHz, DMSO-d_6) δ 165.6, 138.1, 134.7, 131.7, 128.5, 128.4, 127.7, 127.2, 121.8. HRMS (ESI): m/z $[M+H]^+$ calcd for $C_{13}H_{11}NOCl$: 232.0529; found: 232.0522.

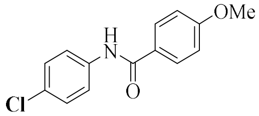

***N*-(4-chlorophenyl)-4-methoxybenzamide (33)** was synthesized using 18.6 mg of aniline, 35 mg of 4-methoxybenzoyl chloride and following general procedure A using 5 mA constant current for 3.5 h. Compound **33** was isolated using automated flash chromatography (silica gel, gradient elution 5-30% EtOAc in isohexane) as an off-white solid (42.5 mg, 82% yield). ^{1}H NMR (400 MHz, DMSO-d_6) δ 10.22 (s, 1H), 8.02 – 7.90 (m, 2H), 7.89 – 7.77 (m, 2H), 7.39 (d, J = 8.9 Hz, 2H), 7.06 (d, J = 8.8 Hz, 2H), 3.84 (s, 3H). ^{13}C NMR (101 MHz, DMSO-d_6) δ 165.0, 162.0, 138.4, 129.7, 128.4, 127.0, 126.7, 121.8, 113.6, 55.4.

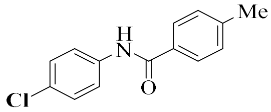

***N*-(4-chlorophenyl)-4-methylbenzamide (34)** was synthesized using 18.6 mg of aniline, 28 μL of 4-methlbenzoyl chloride and following general procedure A using 5 mA constant current for 3.5 h. Compound **34** was isolated using automated flash chromatography (silica gel, gradient elution 5-10% EtOAc in isohexane) as an off-white solid (37.5 mg, 77% yield). ^{1}H NMR (400 MHz, DMSO-d_6) δ 10.29 (s, 1H), 7.87 (d, J = 8.2 Hz, 2H), 7.82 (d, J = 8.9 Hz, 2H), 7.40 (d, J = 8.9 Hz, 2H), 7.34 (d, J = 8.0 Hz, 2H), 2.38 (s, 3H). ^{13}C NMR (101 MHz, DMSO-d_6) δ 165.4, 141.7, 138.2, 131.8, 128.9, 128.5, 127.7, 127.1, 121.8, 21.0.

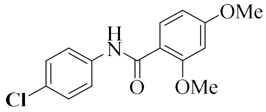

***N*-(4-chlorophenyl)-2,4-dimethoxybenzamide (35)** was synthesized using 18.6 mg of aniline, 37 μL of 2,4-dimethoxybenzoyl chloride and following general procedure A using 5 mA constant current for 3.5 h. Compound **35** was isolated using automated flash chromatography (silica gel, gradient elution 5-10% EtOAc in isohexane) as a pale brown solid (35.5 mg, 61% yield). ^{1}H NMR (400 MHz, $CDCl_3$) δ 9.59 (s, 1H), 8.29 (s, 1H), 7.67 – 7.60 (m, 2H), 7.35 (dd, J = 8.5, 7.3 Hz, 2H), 7.17 – 7.07 (m, 1H), 6.54 (s, 1H), 4.07 (s, 3H), 3.97 (s, 3H). ^{13}C NMR (101 MHz, $CDCl_3$) δ 162.0, 158.6, 157.2, 138.4, 133.7, 129.1, 124.3, 120.5, 115.7, 115.1, 96.3, 56.8, 56.5. HRMS (ESI): m/z $[M+H]^+$ calcd for $C_{15}H_{15}NO_3Cl$: 292.0740; found: 292.0746.

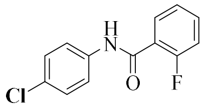

***N*-(4-chlorophenyl)-2-fluorobenzamide (36)** was synthesized using 18.6 mg of aniline, 25 μL of 2-fluorobenzoyl chloride and following general procedure A using 5 mA constant current for 3.5 h. Compound **36** was isolated using automated flash chromatography (silica gel, gradient elution 10-30%

EtOAc in isohexane) as a white solid (33.5 mg, 68% yield). ^{1}H NMR (400 MHz, $CDCl_3$) δ 8.47 (d, *J* = 15.4 Hz, 1H), 8.15 (tdd, *J* = 7.9, 5.8, 1.9 Hz, 1H), 7.68 – 7.59 (m, 2H), 7.56 – 7.48 (m, 1H), 7.39 – 7.28 (m, 3H), 7.21 – 7.13 (m, 1H). ^{19}F NMR (376 MHz, $CDCl_3$) δ -113.17 (m).

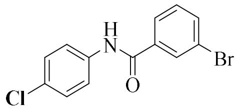

3-bromo-*N*-(4-chlorophenyl)benzamide (37) was synthesized using 18.6 mg of aniline, 28 μL of 3-bromobenzoyl chloride and following general procedure A using 5 mA constant current for 3.5 h. Compound **37** was isolated using automated flash chromatography (silica gel, gradient elution 10-30% EtOAc in isohexane) as a white solid (54.5 mg, 88% yield). ^{1}H NMR (400 MHz, $CDCl_3$) δ 8.27 (s, 1H), 7.94 (t, *J* = 1.8 Hz, 1H), 7.73 (ddd, *J* = 7.8, 1.8, 1.1 Hz, 1H), 7.64 (ddd, *J* = 8.0, 2.0, 1.0 Hz, 1H), 7.59 – 7.54 (m, 2H), 7.32 – 7.27 (m, 3H). ^{13}C NMR (101 MHz, $CDCl_3$) δ 164.8, 136.6, 136.2, 135.0, 130.4, 130.1, 129.2 (2C), 125.8, 123.0, 122.0.

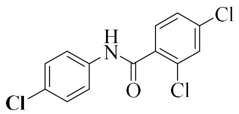

2,4-dichloro-N-(4-chlorophenyl)benzamide (38) was synthesized using 18.6 mg of aniline, 29.5 μL of 2,4-dichlorobenzoyl chloride and following general procedure A using 5 mA constant current for 3.5 h. Compound **38** was isolated using automated flash chromatography (silica gel, gradient elution 10-30% EtOAc in isohexane) as a white solid (51.4 mg, 86% yield). ^{1}H NMR (400 MHz, DMSO-d_6) δ 10.67 (s, 1H), 7.77 (d, *J* = 2.0 Hz, 1H), 7.74 – 7.71 (m, 2H), 7.64 (d, *J* = 8.2 Hz, 1H), 7.56 (dd, *J* = 8.2, 2.0 Hz, 1H), 7.44 – 7.40 (m, 2H). ^{13}C NMR (101 MHz, DMSO-d_6) δ 164.1, 137.7, 135.5, 135.0, 131.2, 130.4, 129.2, 128.8, 127.6, 127.5, 121.1.

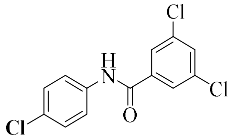

3,5-Dichloro-*N*-(4-chlorophenyl)benzamide (39) was synthesized using 18.6 mg of aniline, 44 mg of 3,5-dichlorobenzoyl chloride and following general procedure A using 5 mA constant current for 3.5 h. Compound **39** was isolated using automated flash chromatography (silica gel, gradient elution 10-30% EtOAc in isohexane) as a white solid (52.5 mg, 88% yield). ^{1}H NMR (400 MHz, $CDCl_3$) δ 7.84 (s, 1H), 7.71 (dd, *J* = 3.3, 1.8 Hz, 2H), 7.59 – 7.51 (m, 3H), 7.36 – 7.32 (m, 2H). ^{13}C NMR (101 MHz, $CDCl_3$) δ 163.3, 137.6, 135.9, 135.9, 132.0, 130.4, 129.4, 125.8, 121.8.

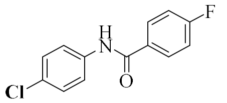

***N*-(4-chlorophenyl)-4-fluorobenzamide (40)** was synthesized using 18.6 mg of aniline, 24.8 μL of 4-fluorobenzoyl chloride and following general procedure A using 5 mA constant current for 3.5 h. Compound **40** was isolated using automated flash chromatography (silica gel, gradient elution 10-30% EtOAc in isohexane) as a white solid (37 mg, 74% yield). ^{1}H NMR (400 MHz, DMSO-d_6) δ 10.40 (s, 1H), 8.12 – 7.94 (m, 2H), 7.87 – 7.77 (m, 2H), 7.48 – 7.31 (m, 4H). ^{13}C NMR (101 MHz, DMSO-d_6) δ 165.0, 164.6 (d, J = 249.2 Hz), 138.5, 130.9 (d, J = 9.1 Hz), 129.6, 129.0, 127.8, 122.3, 115.8 (d, J = 21.9 Hz).^{19}F NMR (376 MHz, DMSO-d_6) δ -108.57.

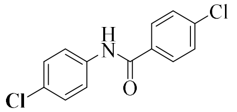

4-Chloro-*N*-(4-chlorophenyl)benzamide (41) was synthesized using 18.6 mg of aniline, 27 μL of 4-chlorobenzoyl chloride and following general procedure A using 5 mA constant current for 3.5 h. Compound **41** was isolated using automated flash chromatography (silica gel, gradient elution 10-30% EtOAc in isohexane) as a white solid (39 mg, 74% yield). ^{1}H NMR (400 MHz, DMSO-d_6) δ 10.44 (s, 1H), 8.06 - 7.91 (m, 2H), 7.89 – 7.76 (m, 2H), 7.70 – 7.54 (m, 2H), 7.48 – 7.35 (m, 2H). ^{13}C NMR (101 MHz, DMSO-d_6) δ 164.5, 138.0, 136.5, 133.4, 129.7, 128.5, 128.5, 127.4, 121.9.

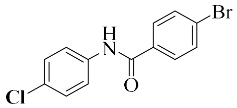

4-Bromo-N-(4-chlorophenyl)benzamide (42) was synthesized using 18.6 mg of aniline, 46 mg of 4-bromobenzoyl chloride and following general procedure A using 5 mA constant current for 3.5 h. Compound **42** was isolated using automated flash chromatography (silica gel, gradient elution 10-30% EtOAc in isohexane) as a white solid (53.5 mg, 87% yield). ^{1}H NMR (400 MHz, $CDCl_3$) δ 7.77 (s, 1H), 7.75 – 7.70 (m, 2H), 7.65 – 7.55 (m, 4H), 7.36 – 7.31 (m, 2H). ^{13}C NMR (101 MHz, $CDCl_3$) δ 164.8, 136.3, 133.6, 132.3, 130.0, 129.3, 128.7, 127.0, 121.6.

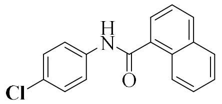

***N*-(4-chlorophenyl)-1-naphthamide (43)** was synthesized using 18.6 mg of aniline, 31.5 μL of 1-naphthoyl chloride and following general procedure A using 5 mA constant current for 3 h. Compound **43** was isolated using automated flash chromatography (silica gel, gradient elution 10-30% EtOAc in isohexane) as a white solid (51 mg, 91% yield). ^{1}H NMR (400 MHz, DMSO-d_6) δ 10.71 (s, 1H), 8.23 – 8.15 (m, 1H), 8.09 (dt, J = 8.4, 1.1 Hz, 1H), 8.06 – 8.00 (m, 1H), 7.86 (dd, J = 9.2, 2.6 Hz, 2H), 7.77

(dd, J = 7.0, 1.3 Hz, 1H), 7.65 – 7.56 (m, 3H), 7.47 – 7.40 (m, 2H). ^{13}C NMR (101 MHz, DMSO-d$_6$) δ 167.4, 138.3, 134.5, 133.2, 130.3, 129.6, 128.7, 128.4, 127.3, 127.1, 126.4, 125.6, 125.1, 125.0, 121.3.

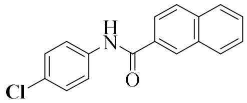

***N*-(4-chlorophenyl)-2-naphthamide (44)** was synthesized using 18.6 mg of aniline, 40 mg of 2-naphthoyl chloride and following general procedure A using 5 mA constant current for 3 h. Compound **44** was isolated using automated flash chromatography (silica gel, gradient elution 10-30% EtOAc in isohexane) as a white solid (51.5 mg, 92% yield). ^{1}H NMR (400 MHz, CDCl$_3$) δ 8.38 (t, J = 1.2 Hz, 1H), 7.99 – 7.89 (m, 5H), 7.69 – 7.64 (m, 2H), 7.63 – 7.56 (m, 2H), 7.39 – 7.34 (m, 2H). ^{13}C NMR (101 MHz, CDCl$_3$) δ 165.8, 136.7, 135.1, 132.8, 132.0, 129.7, 129.3, 129.1, 129.0, 128.2, 128.0, 127.7, 127.2, 123.6, 121.6.

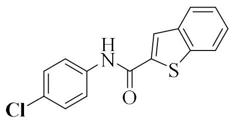

***N*-(4-chlorophenyl)benzo[*b*]thiophene-2-carboxamide (45)** was synthesized using 18.6 mg of aniline, 41.3 mg of benzo[*b*]thiophene-2-carbonyl chloride and following general procedure A using 5 mA constant current for 3 h. Compound **45** was isolated using automated flash chromatography (silica gel, gradient elution 5-30% EtOAc in isohexane) as a pale yellow solid (48 mg, 84% yield). ^{1}H NMR (400 MHz, DMSO-d_6) δ 8.42 (d, J = 0.8 Hz, 1H), 8.08 – 8.05 (m, 1H), 8.03 – 8.00 (m, 1H), 7.86 – 7.82 (m, 2H), 7.53 – 7.41 (m, 5H). ^{13}C NMR (101 MHz, DMSO-d_6) δ 160.4, 140.5, 139.7, 139.1, 137.6, 128.7, 127.6, 126.6, 126.2, 125.5, 125.1, 122.9, 121.8.

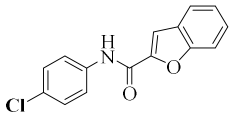

***N*-(4-chlorophenyl)benzofuran-2-carboxamide (46)** was synthesized using 18.6 mg of aniline, 41.3 mg of benzofuran-2-carbonyl chloride and following general procedure A using 5 mA constant current for 5 h. Compound **46** was isolated using automated flash chromatography (silica gel, gradient elution 5-30% EtOAc in isohexane) as a white solid (42 mg, 78% yield). ^{1}H NMR (400 MHz, DMSO-d_6) δ 10.67 (s, 1H), 7.89 – 7.85 (m, 2H), 7.83 (dt, J = 7.8, 1.0 Hz, 1H), 7.79 (d, J = 1.0 Hz, 1H), 7.72 (dd, J = 8.4, 1.0 Hz, 1H), 7.51 (ddd, J = 8.5, 7.2, 1.3 Hz, 1H), 7.45 – 7.41 (m, 2H), 7.37 (td, J = 7.5, 7.1, 0.9 Hz, 1H). ^{13}C NMR (101 MHz, DMSO-d_6) δ 156.7, 154.5, 148.5, 137.4, 128.6, 127.7, 127.3, 127.1, 123.9, 123.0, 122.0, 112.0, 111.0.

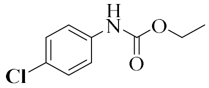

Ethyl (4-chlorophenyl)carbamate (47) was synthesized using 18.6 mg of aniline, 27 μL of ethyl chloroformate and following general procedure A using 5 mA constant current for 3 h. Compound **47** was isolated using automated flash chromatography (silica gel, gradient elution 5-30% EtOAc in isohexane) as a white crystalline solid (38.5 mg, 97% yield) following work-up without the need for any further purification. ^{1}H NMR (400 MHz, $CDCl_3$) δ 7.38 – 7.32 (m, 2H), 7.30 – 7.25 (m, 2H), 6.73 (s, 1H), 4.24 (q, J = 7.1 Hz, 2H), 1.32 (t, J = 7.1 Hz, 3H). ^{13}C NMR (101 MHz, $CDCl_3$) δ 153.6, 136.7, 129.1, 128.4, 120.0, 61.5, 14.6.

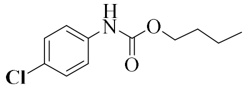

Butyl (4-chlorophenyl)carbamate (48) was synthesized using 18.6 mg of aniline, 20 μL of butyl chloroformate and following general procedure A using 5 mA constant current for 3 h. Compound **48** was isolated using automated flash chromatography (silica gel, gradient elution 5-30% EtOAc in isohexane) as a white solid (43.5 mg, 96% yield). ^{1}H NMR (400 MHz, $CDCl_3$) δ 7.32 (d, J = 8.5 Hz, 2H), 7.27 – 7.23 (m, 2H), 6.60 (s, 1H), 4.16 (t, J = 6.7 Hz, 2H), 1.64 (dq, J = 8.6, 6.8 Hz, 2H), 1.46 – 1.35 (m, 2H), 0.94 (t, J = 7.4 Hz, 3H). ^{13}C NMR (101 MHz, $CDCl_3$) δ 153.7, 136.7, 129.2, 128.4, 119.9, 65.5, 31.1, 19.2, 13.8. HRMS (ESI): m/z $[M-H]^-$ calcd for $C_{11}H_{13}NO_2Cl$: 226.0640; found: 226.0638.

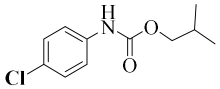

Isobutyl (4-chlorophenyl)carbamate (49) was synthesized using 18.6 mg of aniline, 27.5 μL of isobutyl chloroformate and following general procedure A using 5 mA constant current for 3 h. Compound **49** was isolated using automated flash chromatography (silica gel, gradient elution 5-30% EtOAc in isohexane) as a white solid (43.0 mg, 95% yield). ^{1}H NMR (400 MHz, $CDCl_3$) δ 7.34 (d, J = 8.5 Hz, 2H), 7.26 (dt, J = 6.6, 2.5 Hz, 2H), 6.61 (s, 1H), 3.95 (d, J = 6.7 Hz, 2H), 1.97 (dt, J = 13.4, 6.7 Hz, 1H), 0.96 (d, J = 6.7 Hz, 6H). ^{13}C NMR (101 MHz, $CDCl_3$) δ 153.7, 136.7, 129.2, 128.5, 120.0, 71.7, 28.1, 19.2. HRMS (ESI): m/z $[M-H]^-$ calcd for $C_{11}H_{13}NO_2Cl$: 226.0640; found: 226.0637.

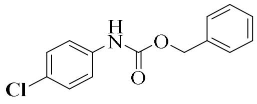

Benzyl (4-chlorophenyl)carbamate (50) was synthesized using 18.6 mg of aniline, 30 μL of isobutyl chloroformate and following general procedure A using 5 mA constant current for 3 h. Compound **50** was isolated using automated flash chromatography (silica gel, gradient elution 5-30% EtOAc in isohexane) as a white solid (45.4 mg, 87% yield). ^{1}H NMR (400 MHz, $CDCl_3$) δ 7.41 – 7.30 (m, 7H),

7.28 – 7.25 (m, 2H), 6.64 (s, 1H), 5.20 (s, 2H). ^{13}C NMR (101 MHz, $CDCl_3$) δ 153.3, 136.5, 136.0, 129.2, 128.8, 128.6, 128.5, 123.7, 120.0, 67.3.

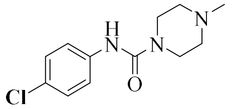

***N*-(4-chlorophenyl)-4-methylpiperazine-1-carboxamide (51)** was synthesized using 18.6 mg of aniline, 41.8 mg of 4-Methyl-1-piperazinecarbonyl chloride hydrochloride and following general procedure A using 5 mA constant current for 22 h. Compound **51** was isolated using automated flash chromatography (silica gel, gradient elution 70% EtOAc in isohexane) as a white solid (19.0 mg, 38% yield). ^{1}H NMR (400 MHz, DMSO-d_6) 9.02 (d, J = 6.6 Hz, 1H), 7.53 (dd, J = 8.9, 2.0 Hz, 2H), 7.34 – 7.24 (m, 2H), 3.19 (s, 4H), 2.75 (s, 3H), 2.50 (m, 4H). ^{13}C NMR (101 MHz, DMSO-d_6) δ 154.4, 139.2, 128.2, 125.6, 121.1, 52.1, 42.1, 41.1.

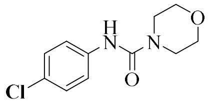

***N*-(4-chlorophenyl)morpholine-4-carboxamide (52)** was synthesized using 18.6 mg of aniline, 24.5 µL of 4-Morpholinecarbonyl chloride and following general procedure A using 2 mA constant current for 89 h. Compound **52** was isolated using automated flash chromatography (silica gel, gradient elution 70% EtOAc in isohexane) as a white solid (27.2 mg, 57% yield). ^{1}H NMR (400 MHz, DMSO-d_6) δ 8.64 (s, 1H), 7.57 – 7.42 (m, 2H), 7.31 – 7.20 (m, 2H), 3.60 (dd, J = 5.6, 4.0 Hz, 4H), 3.48 – 3.38 (m, 4H). ^{13}C NMR (101 MHz, DMSO-d_6) δ 154.9, 139.4, 128.2, 125.3, 121.0, 66.0, 44.1.

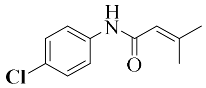

***N*-(4-chlorophenyl)-3-methylbut-2-enamide (53)** was synthesized using 18.6 mg of aniline, 23.5 µL of 3,3-Dimethylacryloyl chloride and following general procedure A using 1 mA constant current for 20 h. Compound **53** was isolated using automated flash chromatography (silica gel, gradient elution 5-30% EtOAc in isohexane) as a white solid (26.7 mg, 64% yield). ^{1}H NMR (400 MHz, $CDCl_3$) δ 7.41 (d, J = 8.5 Hz, 2H), 7.23 (s, 1H), 7.20 – 7.15 (m, 2H), 5.62 (p, J = 1.4 Hz, 1H), 2.14 (d, J = 1.3 Hz, 3H), 1.81 (d, J = 1.4 Hz, 3H). ^{13}C NMR (101 MHz, $CDCl_3$) δ 165.2, 154.3, 136.9, 129.0 (2C), 121.1, 118.5, 27.6, 20.1.

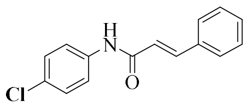

***N*-(4-chlorophenyl)cinnamamide (54)** was synthesized using 18.6 mg of aniline, 30 µL of cinnamoyl chloride and following general procedure A using 2 mA constant current for 18 h. Compound **54** was

isolated using automated flash chromatography (silica gel, gradient elution 5-30% EtOAc in isohexane) as a white solid (25.5 mg, 50% yield). ^{1}H NMR (400 MHz, $CDCl_3$) δ 7.76 (d, *J* = 15.5 Hz, 1H), 7.58 (d, *J* = 8.3 Hz, 2H), 7.55 – 7.50 (m, 2H), 7.42 (s, 1H), 7.41 – 7.36 (m, 3H), 7.33 – 7.28 (m, 2H), 6.54 (d, *J* = 15.5 Hz, 1H). ^{13}C NMR (101 MHz, $CDCl_3$) δ 164.1, 143.0, 136.7, 134.6, 130.3, 129.3, 129.1 (2C), 128.1, 121.3, 120.5.

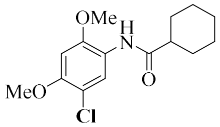

***N*-(5-chloro-2,4-dimethoxyphenyl)cyclohexanecarboxamide (55)** was synthesized using 30.6 mg of 2,4-dimethoxyaniline, 28.0 μL of cyclohexanecarbonyl chloride and following general procedure A using 5 mA constant current for 3 h. Compound **55** was isolated using automated flash chromatography (silica gel, gradient elution 5-30% EtOAc in isohexane) as an off-white solid (55.0 mg, 93% yield). ^{1}H NMR (400 MHz, $CDCl_3$) δ 8.45 (s, 1H), 7.57 (s, 1H), 6.49 (s, 1H), 3.89 (s, 3H), 3.86 (s, 3H), 2.23 (tt, *J* = 11.7, 3.5 Hz, 1H), 1.98 – 1.90 (m, 2H), 1.86 – 1.79 (m, 2H), 1.72 – 1.66 (m, 1H), 1.51 (qd, *J* = 12.1, 3.4 Hz, 2H), 1.37 – 1.24 (m, 3H). ^{13}C NMR (101 MHz, $CDCl_3$) δ 174.0, 151.1, 147.6, 121.8, 121.5, 113.8, 96.7, 56.8, 56.2, 46.7, 29.8, 25.8, 25.8. HRMS (ESI): m/z $[M+H]^+$ calcd for $C_{15}H_{21}NO_3Cl$: 298.1210; found: 298.1197.

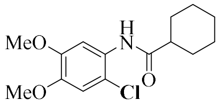

***N*-(2-chloro-4,5-dimethoxyphenyl)cyclohexanecarboxamide (56)** was synthesized using 30.6 mg of 3,4-dimethoxyaniline, 28.0 μL of cyclohexanecarbonyl chloride and following general procedure A using 5 mA constant current for 3 h. Compound **56** was isolated using automated flash chromatography (silica gel, gradient elution 5-30% EtOAc in isohexane) as an off-white solid (54.0 mg, 91% yield). ^{1}H NMR (400 MHz, $CDCl_3$) δ 8.12 (d, *J* = 3.2 Hz, 1H), 7.52 (d, *J* = 3.8 Hz, 1H), 6.84 (s, 1H), 3.89 (s, 3H), 3.85 (s, 3H), 2.33 – 2.25 (m, 1H), 2.01 (d, *J* = 12.8 Hz, 2H), 1.89 – 1.83 (m, 2H), 1.73 (d, *J* = 11.6 Hz, 1H), 1.53 (td, *J* = 12.3, 3.4 Hz, 2H), 1.38 – 1.27 (m, 3H). ^{13}C NMR (101 MHz, $CDCl_3$) δ 174.3, 148.1, 145.5, 128.3, 113.3, 111.7, 105.5, 56.4, 56.2, 46.6, 29.8, 25.8, 25.7. HRMS (ESI): m/z $[M+H]^+$ calcd for $C_{15}H_{21}NO_3Cl$: 298.1210; found: 298.1196.

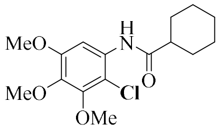

***N*-(2-chloro-3,4,5-trimethoxyphenyl)cyclohexanecarboxamide (57)** was synthesized using 36.6 mg of 3,4,5-trimethoxyaniline, 28.0 μL of cyclohexanecarbonyl chloride and following general procedure A using 5 mA constant current for 3 h. Compound **57** was isolated using automated flash chromatography (silica gel, gradient elution 5-30% EtOAc in isohexane) as a white solid (60.5 mg, 93%

yield). ^{1}H NMR (400 MHz, $CDCl_3$) δ 7.99 (d, *J* = 2.2 Hz, 1H), 7.67 (s, 1H), 3.91 (s, 3H), 3.88 (s, 3H), 3.85 (s, 3H), 2.30 (tt, *J* = 11.7, 3.5 Hz, 1H), 2.03 – 1.97 (m, 2H), 1.89 – 1.81 (m, 2H), 1.75 – 1.69 (m, 2H), 1.60 – 1.48 (m, 2H), 1.41 – 1.24 (m, 2H). ^{13}C NMR (101 MHz, $CDCl_3$) δ 174.6, 152.4, 149.7, 139.2, 131.1, 108.6, 100.8, 61.3 (2C), 56.2, 46.8, 29.8, 25.8, 25.7. HRMS (ESI): m/z $[M+H]^+$ calcd for $C_{16}H_{23}NO_4Cl$: 328.1316; found: 328.1302.

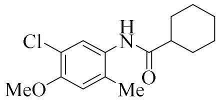

***N*-(5-chloro-4-methoxy-2-methylphenyl)cyclohexanecarboxamide (58)** was synthesized using 27.4 mg of 4-methoxy-2-methylaniline, 28.0 μL of cyclohexanecarbonyl chloride and following general procedure A using 5 mA constant current for 3 h. Compound **58** was isolated using automated flash chromatography (silica gel, gradient elution 5-30% EtOAc in isohexane) as a white solid (41.5 mg, 74% yield). ^{1}H NMR (400 MHz, $CDCl_3$) δ 7.70 (s, 1H), 6.82 (s, 1H), 6.74 (s, 1H), 3.87 (s, 3H), 2.26 (ddd, *J* = 11.6, 8.1, 3.5 Hz, 1H), 2.22 (s, 3H), 2.01 – 1.95 (m, 2H), 1.88 – 1.82 (m, 2H), 1.72 (d, *J* = 10.7 Hz, 1H), 1.60 – 1.48 (m, 2H), 1.37 – 1.23 (m, 3H). ^{13}C NMR (101 MHz, $CDCl_3$) δ 174.5, 152.6, 130.5, 129.0, 126.3, 120.0, 114.0, 56.5, 46.2, 30.0, 25.8 (2C), 18.1. HRMS (ESI): m/z $[M+H]^+$ calcd for $C_{15}H_{21}NO_2Cl$: 282.1261; found: 282.1274.

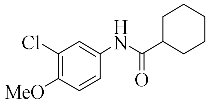

***N*-(3-chloro-4-methoxyphenyl)cyclohexanecarboxamide (59)** was synthesized using 24.6 mg of 4-methoxyaniline, 28.0 μL of cyclohexanecarbonyl chloride and following general procedure A using 1 mA constant current for 18 h. Compound **59** was isolated using automated flash chromatography (silica gel, gradient elution 5-30% EtOAc in isohexane) as a white solid (45 mg, 85% yield). ^{1}H NMR (400 MHz, $CDCl_3$) δ 8.20 (d, *J* = 9.1 Hz, 1H), 7.48 (s, 1H), 6.92 (d, *J* = 2.8 Hz, 1H), 6.81 (dd, *J* = 9.1, 2.8 Hz, 1H), 3.77 (s, 3H), 2.28 (tt, *J* = 11.7, 3.5 Hz, 1H), 2.04 – 1.96 (m, 2H), 1.84 (dt, *J* = 13.0, 3.5 Hz, 2H), 1.74 – 1.67 (m, 1H), 1.53 (qd, *J* = 12.1, 3.2 Hz, 2H), 1.31 (dqd, *J* = 15.1, 12.3, 9.1 Hz, 3H). ^{13}C NMR (101 MHz, $CDCl_3$) δ 174.2, 156.2, 128.2, 124.1, 123.2, 114.6, 113.3, 55.8, 46.6, 29.8, 25.8, 25.8. HRMS (ESI): m/z $[M+H]^+$ calcd for $C_{14}H_{19}NO_2Cl$: 268.1104; found: 268.1100.

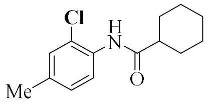

***N*-(2-chloro-4-methylphenyl)cyclohexanecarboxamide (60)** was synthesized using 21.4 mg of *p*-toluidine, 28.0 μL of cyclohexanecarbonyl chloride and following general procedure A using 1 mA constant current for 18 h. Compound **60** was isolated using automated flash chromatography (silica gel, gradient elution 5-30% EtOAc in isohexane) as a white solid (32 mg, 64% yield). ^{1}H NMR (400 MHz, $CDCl_3$) δ 8.25 (d, *J* = 8.3 Hz, 1H), 7.61 (s, 1H), 7.19 – 7.15 (m, 1H), 7.06 (dd, *J* = 8.4, 2.0 Hz, 1H),

2.29 (s, 3H), 2.34 – 2.25 (m, 1H), 2.01 (d, *J* = 12.7 Hz, 2H), 1.88 – 1.82 (m, 2H), 1.72 (d, *J* = 11.4 Hz, 1H), 1.59 – 1.48 (m, 2H), 1.38 – 1.22 (m, 3H). ^{13}C NMR (101 MHz, $CDCl_3$) δ 174.3, 134.6, 132.3, 129.3, 128.5, 122.6, 121.6, 46.8, 29.8, 25.8, 25.8, 20.8. HRMS (ESI): m/z $[M+H]^+$ calcd for $C_{14}H_{19}NOCl$: 252.1155; found: 252.1154.

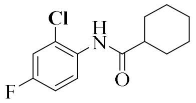

***N*-(2-chloro-4-fluorophenyl)cyclohexanecarboxamide (61)** was synthesized using 22.2 mg of 4-fluoroaniline, 28.0 μL of cyclohexanecarbonyl chloride and following general procedure A using 10 mA constant current for 16 h. Compound **61** was isolated using automated flash chromatography (silica gel, gradient elution 5-30% EtOAc in isohexane) as a white solid (44.0 mg, 87% yield). ^{1}H NMR (400 MHz, $CDCl_3$) δ 8.34 (dd, *J* = 9.2, 5.7 Hz, 1H), 7.57 (s, 1H), 7.12 (dd, *J* = 8.1, 2.9 Hz, 1H), 6.99 (ddd, *J* = 9.2, 7.9, 2.9 Hz, 1H), 2.30 (tt, *J* = 11.7, 3.5 Hz, 1H), 2.04 – 1.97 (m, 2H), 1.85 (dt, *J* = 11.8, 3.0 Hz, 2H), 1.72 (dtt, *J* = 10.7, 3.4, 1.5 Hz, 1H), 1.54 (qd, *J* = 12.1, 3.2 Hz, 2H), 1.40 – 1.23 (m, 3H). ^{13}C NMR (101 MHz, $CDCl_3$) δ 174.3, 159.6, 157.1, 131.3 (d, *J* = 3.4 Hz), 123.0 (d, *J* = 8.2 Hz), 116.3 (d, *J* = 26.0 Hz), 114.7 (d, *J* = 21.5 Hz), 46.6, 29.8, 25.8, 25.7. ^{19}F NMR (376 MHz, $CDCl_3$) δ -116.71 (q, *J* = 7.6 Hz). HRMS (ESI): m/z $[M+H]^+$ calcd for $C_{13}H_{16}NOClF$: 256.0904; found: 256.0903.

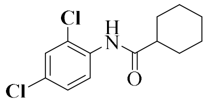

***N*-(2,4-dichlorophenyl)cyclohexanecarboxamide (4)** was synthesized using 25.4 mg of 4-chloroaniline, 28.0 μL of cyclohexanecarbonyl chloride and following general procedure A using 10 mA constant current for 18 h. Compound **4** was isolated using automated flash chromatography (silica gel, gradient elution 5-30% EtOAc in isohexane) as a white solid (43.2 mg, 80% yield). ^{1}H NMR (400 MHz, $CDCl_3$) δ 8.38 (d, *J* = 8.9 Hz, 1H), 7.64 (s, 1H), 7.37 (d, *J* = 2.4 Hz, 1H), 7.24 (dd, *J* = 8.9, 2.4 Hz, 1H), 2.30 (tt, *J* = 11.7, 3.5 Hz, 1H), 2.03 – 1.97 (m, 2H), 1.85 (dt, *J* = 13.1, 3.6 Hz, 2H), 1.72 (dddd, *J* = 11.2, 4.9, 2.9, 1.5 Hz, 1H), 1.52 (td, *J* = 12.3, 3.2 Hz, 2H), 1.37 – 1.25 (m, 3H). ^{13}C NMR (101 MHz, CDCl3) δ 174.4, 133.6, 128.9, 128.7, 128.0, 123.2, 122.3, 46.7, 29.8, 25.8, 25.7.

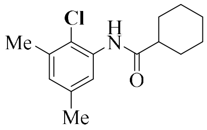

***N*-(2-chloro-3,5-dimethylphenyl)cyclohexanecarboxamide (62)** was synthesized using 24.2 mg of 3,5-dimethylaniline, 28.0 μL of cyclohexanecarbonyl chloride and following general procedure A using 5 mA constant current for 2 h. Compound **62** was isolated using automated flash chromatography (silica gel, gradient elution 5-30% EtOAc in isohexane) as a white solid (47.5 mg, 90% yield). ^{1}H NMR (400 MHz, $CDCl_3$) δ 8.17 – 8.03 (m, 1H), 7.73 (s, 1H), 6.80 (dt, *J* = 2.2, 0.8 Hz, 1H), 2.34 (s, 3H), 2.29 (s,

3H), 2.32 – 2.26 (m, 1H), 2.04 – 1.97 (m, 2H), 1.89 – 1.82 (m, 2H), 1.72 (dtt, *J* = 10.8, 3.5, 1.5 Hz, 1H), 1.54 (qd, *J* = 12.1, 3.1 Hz, 2H), 1.41 – 1.24 (m, 3H). ^{13}C NMR (101 MHz, $CDCl_3$) δ 174.4, 137.0, 135.9, 134.4, 126.7, 120.1, 119.6, 46.9, 29.8, 25.8, 25.8, 21.3, 20.7. HRMS (ESI): m/z $[M+H]^+$ calcd for $C_{15}H_{21}NOCl$: 266.1312; found: 266.1312.

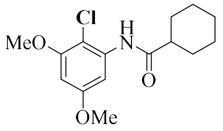

***N*-(2-chloro-3,5-dimethoxyphenyl)cyclohexanecarboxamide (63)** was synthesized using 30.6 mg of 3,5-dimethoxyaniline, 28.0 μL of cyclohexanecarbonyl chloride and following general procedure A using 5 mA constant current for 3 h. Compound **63** was isolated using automated flash chromatography (silica gel, gradient elution 5-30% EtOAc in isohexane) as a white solid (37.4 mg, 63% yield). ^{1}H NMR (400 MHz, $CDCl_3$) δ 7.87 – 7.76 (m, 2H), 6.29 (d, *J* = 2.8 Hz, 1H), 3.86 (s, 3H), 3.81 (s, 3H), 2.31 (tt, *J* = 11.6, 3.5 Hz, 1H), 2.04 – 1.98 (m, 2H), 1.88 – 1.82 (m, 2H), 1.72 (dtd, *J* = 11.0, 3.2, 1.6 Hz, 1H), 1.54 (qd, *J* = 12.1, 3.3 Hz, 2H), 1.38 – 1.23 (m, 3H). ^{13}C NMR (101 MHz, $CDCl_3$) δ 174.7, 159.4, 155.5, 136.3, 102.8, 97.4, 95.8, 56.3, 55.8, 47.0, 29.8, 25.8, 25.5. HRMS (ESI): m/z $[M+H]^+$ calcd for $C_{15}H_{21}NO_3Cl$: 298.1210; found: 298.1197.

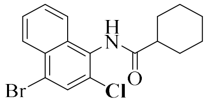

***N*-(4-bromo-2-chloronaphthalen-1-yl)cyclohexanecarboxamide (64)** was synthesized using 44.0 mg of 4-bromonaphthalen-1-amine, 28.0 μL of cyclohexanecarbonyl chloride and following general procedure A using 5 mA constant current for 3 h. Compound **64** was isolated using automated flash chromatography (silica gel, gradient elution 5-30% EtOAc in isohexane) as a white solid (68.5 mg, 94% yield). ^{1}H NMR (400 MHz, DMSO-d_6) δ 9.88 (s, 1H), 8.19 – 8.12 (m, 1H), 8.07 (s, 1H), 7.95 – 7.78 (m, 1H), 7.80 – 7.64 (m, 2H), 2.55 (ddd, *J* = 11.9, 8.2, 3.7 Hz, 1H), 1.98 (dd, *J* = 12.7, 4.0 Hz, 2H), 1.80 (dt, *J* = 12.5, 3.3 Hz, 2H), 1.68 (d, *J* = 12.2 Hz, 1H), 1.50 (qd, *J* = 12.4, 3.2 Hz, 2H), 1.40 – 1.19 (m, 3H). ^{13}C NMR (101 MHz, DMSO-d_6) δ 174.7, 132.3, 131.7, 130.4, 130.0, 129.3, 128.4, 128.3, 126.8, 124.2, 120.8, 44.1, 29.3, 25.5, 25.2. HRMS (ESI): m/z $[M+H]^+$ calcd for $C_{17}H_{18}NOClBr$: 366.0260; found: 366.0273.

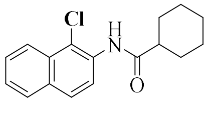

***N*-(1-chloronaphthalen-2-yl)cyclohexanecarboxamide (65)** was synthesized using 28.6 mg of naphthalen-2-amine, 28.0 μL of cyclohexanecarbonyl chloride and following general procedure A using 5 mA constant current for 3 h. Compound **65** was isolated as a pale brownish solid (55.5 mg, 97% yield)

following work-up without the need for any further purification. ^{1}H NMR (400 MHz, $CDCl_3$) δ 8.56 (dd, J = 9.0, 4.0 Hz, 1H), 8.17 (dt, J = 8.6, 0.9 Hz, 1H), 7.99 – 7.90 (m, 1H), 7.87 – 7.72 (m, 2H), 7.58 (ddd, J = 8.4, 6.8, 1.3 Hz, 1H), 7.46 (ddd, J = 8.1, 6.8, 1.2 Hz, 1H), 2.39 (tt, J = 11.7, 3.5 Hz, 1H), 2.13 – 2.01 (m, 2H), 1.88 (dt, J = 12.4, 3.3 Hz, 2H), 1.74 (dtd, J = 10.8, 3.1, 1.5 Hz, 1H), 1.66 – 1.55 (m, 2H), 1.35 (dddd, J = 23.6, 15.3, 12.3, 9.0 Hz, 3H). ^{13}C NMR (101 MHz, $CDCl_3$) δ 174.6, 133.0, 131.2, 130.7, 128.2, 127.6, 127.5, 125.5, 123.9, 120.7, 118.2, 46.8, 29.8, 25.8, 25.8. HRMS (ESI): m/z $[M+H]^+$ calcd for $C_{17}H_{19}NOCl$: 288.1155; found: 288.1145.

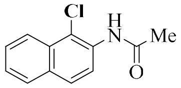

***N*-(1-chloronaphthalen-2-yl)acetamide (66)** was synthesized using 28.6 mg of naphthalen-2-amine, 15.0 μL of acetyl chloride and following general procedure A using 5 mA constant current for 3 h. Compound **66** was isolated using automated flash chromatography (silica gel, gradient elution 5-30% EtOAc in isohexane) as a white solid (40.5 mg, 93% yield). ^{1}H NMR (400 MHz, $CDCl_3$) 8.50 (d, J = 9.0 Hz, 1H), 8.18 (d, J = 8.5 Hz, 1H), 7.80 (dd, J = 16.4, 8.6 Hz, 3H), 7.58 (ddd, J = 8.4, 6.8, 1.4 Hz, 1H), 7.47 (ddd, J = 8.2, 6.9, 1.3 Hz, 1H), 2.31 (s, 3H). ^{13}C NMR (101 MHz, $CDCl_3$) δ 168.6, 132.9, 131.3, 130.7, 128.3, 127.7, 127.6, 125.7, 124.0, 120.6, 117.9, 25.1.

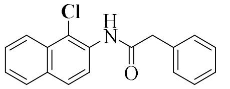

***N*-(1-chloronaphthalen-2-yl)-2-phenylacetamide (67)** was synthesized using 28.6 mg of naphthalen-2-amine, 28.0 μL of phenylacetyl chloride and following general procedure A using 5 mA constant current for 3 h. Compound **67** was isolated using automated flash chromatography (silica gel, gradient elution 5-30% EtOAc in isohexane) as a white solid (51.8 mg, 88% yield). ^{1}H NMR (400 MHz, DMSO-d_6) δ 9.99 (s, 1H), 8.17 (dd, J = 8.5, 1.2 Hz, 1H), 7.99 – 7.95 (m, 1H), 7.90 (d, J = 8.9 Hz, 1H), 7.83 (d, J = 8.9 Hz, 1H), 7.67 (ddd, J = 8.4, 6.9, 1.3 Hz, 1H), 7.57 (ddd, J = 8.1, 6.8, 1.2 Hz, 1H), 7.43 – 7.39 (m, 2H), 7.35 (dd, J = 8.4, 6.8 Hz, 2H), 7.30 – 7.24 (m, 1H), 3.82 (s, 2H). ^{13}C NMR (101 MHz, DMSO-d_6) δ 169.7, 135.8, 133.1, 131.5, 130.2, 129.2, 128.4, 128.2, 127.8, 127.06, 126.6, 126.2, 124.4, 123.5, 122.0, 42.6. HRMS (ESI): m/z $[M+H]^+$ calcd for $C_{18}H_{15}NOCl$: 296.0842; found: 296.0845.

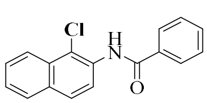

***N*-(1-chloronaphthalen-2-yl)benzamide (68)** was synthesized using 28.6 mg of naphthalen-2-amine, 24.5 μL of benzoyl chloride and following general procedure A using 5 mA constant current for 3 h. Compound **68** was isolated using automated flash chromatography (silica gel, gradient elution 5-30% EtOAc in isohexane) as a white solid (47.5 mg, 85% yield). ^{1}H NMR (400 MHz, DMSO-d_6) δ 10.32 (s, 1H), 8.24 (dd, J = 8.4, 1.1 Hz, 1H), 8.06 (ddd, J = 9.4, 8.1, 1.2 Hz, 3H), 7.99 (d, J = 8.7 Hz, 1H), 7.77 – 7.69 (m, 2H), 7.66 – 7.61 (m, 2H), 7.59 – 7.55 (m, 2H). ^{13}C NMR (101 MHz, DMSO-d_6) δ 165.5,

133.9, 133.2, 132.1, 131.9, 130.4, 128.5, 128.3, 127.8, 127.8, 127.2, 126.5, 126.2, 125.4, 123.7. HRMS (ESI): m/z $[M+H]^+$ calcd for $C_{17}H_{13}NOCl$: 282.0686; found: 282.0680.

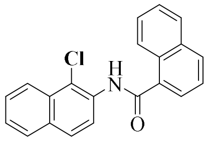

***N*-(1-chloronaphthalen-2-yl)-1-naphthamide (69)** was synthesized using 28.6 mg of naphthalen-2-amine, 31.5 μL of 1-naphthoyl chloride and following general procedure A using 5 mA constant current for 3 h. Compound **69** was isolated using automated flash chromatography (silica gel, gradient elution 5-30% EtOAc in isohexane) as a white solid (59.5 mg, 90% yield). ^1H NMR (400 MHz, $CDCl_3$) δ 8.79 (d, *J* = 9.0 Hz, 1H), 8.54 – 8.50 (m, 1H), 8.48 (s, 1H), 8.21 (dd, *J* = 8.5, 1.1 Hz, 1H), 8.03 (dd, *J* = 8.3, 1.2 Hz, 1H), 7.96 – 7.92 (m, 1H), 7.89 (ddd, *J* = 8.2, 5.1, 1.3 Hz, 3H), 7.65 – 7.55 (m, 4H), 7.51 (ddd, *J* = 8.1, 6.9, 1.2 Hz, 1H). ^{13}C NMR (101 MHz, $CDCl_3$) δ 167.7, 134.1, 134.0, 133.1, 131.7, 131.6, 130.8, 130.4, 128.6, 128.3, 127.8, 127.7, 127.7, 126.9, 125.8, 125.7, 125.5, 125.0, 124.0, 120.7, 118.9. HRMS (ESI): m/z $[M+H]^+$ calcd for $C_{21}H_{15}NOCl$: 332.0842; found: 332.0831.

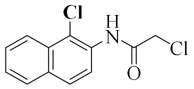

2-chloro-*N*-(1-chloronaphthalen-2-yl)acetamide (70) was synthesized using 28.6 mg of naphthalen-2-amine, 17.0 μL of choloacetyl chloride and following general procedure A using 5 mA constant current for 3 h. Compound **70** was isolated as a pale brownish solid (49.0 mg, 97% yield) following work-up without the need for any further purification. ^1H NMR (400 MHz, $CDCl_3$) δ 9.15 (s, 1H), 8.50 (d, *J* = 9.0 Hz, 1H), 8.20 (dt, *J* = 8.5, 1.0 Hz, 1H), 7.86 – 7.78 (m, 2H), 7.61 (ddd, *J* = 8.4, 6.9, 1.3 Hz, 1H), 7.50 (ddd, *J* = 8.1, 6.9, 1.2 Hz, 1H), 4.29 (s, 2H). ^{13}C NMR (101 MHz, $CDCl_3$) δ 164.2, 131.8, 131.7, 130.7, 128.3, 127.8, 127.8, 126.1, 124.1, 119.9, 119.4, 43.4.

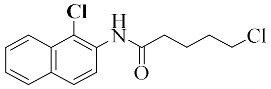

5-chloro-*N*-(1-chloronaphthalen-2-yl)pentanamide (71) was synthesized using 28.6 mg of naphthalen-2-amine, 27.0 μL of 5-chlorovaleryl chloride and following general procedure A using 5 mA constant current for 3 h. Compound **71** was isolated using automated flash chromatography (silica gel, gradient elution 5-30% EtOAc in isohexane) as a white solid (57.0 mg, 97% yield). ^1H NMR (400 MHz, $CDCl_3$) δ 8.49 (d, *J* = 8.9 Hz, 1H), 8.16 (d, *J* = 8.5 Hz, 1H), 7.86 (s, 1H), 7.83 – 7.75 (m, 2H), 7.58 (ddd, *J* = 8.4, 6.8, 1.3 Hz, 1H), 7.47 (ddd, *J* = 8.1, 6.8, 1.2 Hz, 1H), 3.60 (t, *J* = 6.0 Hz, 2H), 2.54 (t, *J* = 6.8 Hz, 2H), 2.02 – 1.86 (m, 4H). ^{13}C NMR (101 MHz, $CDCl_3$) δ 170.9, 132.7, 131.3, 130.7, 128.2, 127.6, 127.6, 125.7, 123.9, 120.7, 118.3, 44.6, 37.0, 32.0, 22.9. HRMS (ESI): m/z $[M+H]^+$ calcd for $C_{15}H_{16}NOCl_2$: 296.0609; found: 296.0615.

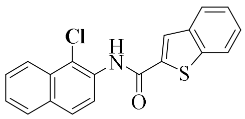

***N*-(1-chloronaphthalen-2-yl)benzo[*b*]thiophene-2-carboxamide (72)** was synthesized using 28.6 mg of naphthalen-2-amine, 41.3 mg of benzo[*b*]thiophene-2-carbonyl chloride and following general procedure A using 5 mA constant current for 6 h. Compound **72** was isolated using automated flash chromatography (silica gel, gradient elution 5-30% EtOAc in isohexane) as a white solid (47.5 mg, 71% yield). ^{1}H NMR (400 MHz, $CDCl_3$) δ 8.67 (d, *J* = 9.2 Hz, 2H), 8.21 (dt, *J* = 8.6, 1.0 Hz, 1H), 7.99 (s, 1H), 7.94 – 7.89 (m, 2H), 7.85 (dd, *J* = 8.7, 3.9 Hz, 2H), 7.61 (ddd, *J* = 8.4, 6.8, 1.3 Hz, 1H), 7.52 – 7.43 (m, 3H). ^{13}C NMR (101 MHz, $CDCl_3$) δ 160.4, 141.5, 139.2, 138.7, 132.6, 131.5, 130.7, 128.4, 127.9, 127.8, 127.0, 126.2, 125.9, 125.5, 125.4, 124.0, 123.0, 120.3, 118.6. HRMS (ESI): m/z $[M+H]^+$ calcd for $C_{19}H_{13}NOSCl$: 338.0406; found: 338.0417.

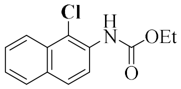

Ethyl (1-chloronaphthalen-2-yl)carbamate (73) was synthesized using 28.6 mg of naphthalen-2-amine, 20.5 µL of ethyl chloroformate and following general procedure A using 5 mA constant current for 3 h. Compound **73** was isolated using automated flash chromatography (silica gel, gradient elution 5-30% EtOAc in isohexane) as a white solid (34.8 mg, 70% yield). ^{1}H NMR (400 MHz, $CDCl_3$) δ 8.39 (d, *J* = 9.0 Hz, 1H), 8.16 (dq, *J* = 8.5, 0.9 Hz, 1H), 7.83 – 7.75 (m, 2H), 7.58 (ddd, *J* = 8.4, 6.9, 1.3 Hz, 1H), 7.45 (ddd, *J* = 8.1, 6.8, 1.2 Hz, 1H), 7.40 – 7.37 (m, 1H), 4.29 (q, *J* = 7.1 Hz, 2H), 1.37 (t, *J* = 7.1 Hz, 3H). ^{13}C NMR (101 MHz, $CDCl_3$) δ 153.5, 133.0, 130.8, 130.8, 128.2, 127.7, 127.6, 125.2, 123.7, 119.2, 117.1, 61.8, 14.6. HRMS (ESI): m/z $[M+H]^+$ calcd for $C_{13}H_{13}NO_2Cl$: 250.0635; found: 250.0627.

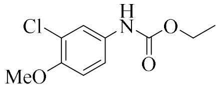

Ethyl (3-chloro-4-methoxyphenyl)carbamate (74) was synthesized using 24.6 mg of 4-methoxyaniline, 20.5 µL of ethyl chloroformate and following general procedure A using 2 mA constant current for 16 h. Compound **74** was isolated using automated flash chromatography (silica gel, gradient elution 5-30% EtOAc in isohexane) as a white solid (33 mg, 72% yield). ^{1}H NMR (400 MHz, $CDCl_3$) δ 7.97 (d, *J* = 8.3 Hz, 1H), 6.91 (d, *J* = 2.9 Hz, 1H), 6.86 – 6.80 (m, 2H), 4.23 (q, *J* = 7.1 Hz, 2H), 3.77 (s, 3H), 1.32 (t, *J* = 7.1 Hz, 3H). ^{13}C NMR (101 MHz, $CDCl_3$) δ 155.8, 153.7, 128.2, 117.0, 114.6, 114.4, 113.5, 61.6, 55.8, 14.7. LC-MS: m/z $[M+H]^+$ calcd for $C_{10}H_{13}NO_3Cl$: 230.1; found: 230.1.

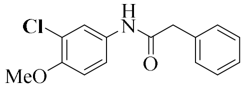

***N*-(3-chloro-4-methoxyphenyl)-2-phenylacetamide (75)** was synthesized using 24.6 mg of 4-methoxyaniline, 28.0 µL of phenylacetyl chloride and following general procedure A using 2 mA

constant current for 1 6h. Compound **75** was isolated using automated flash chromatography (silica gel, gradient elution 5-30% EtOAc in isohexane) as a white solid (40.5 mg, 74% yield). ^{1}H NMR (400 MHz, $CDCl_3$) δ 8.18 (d, *J* = 9.1 Hz, 1H), 7.45 – 7.39 (m, 3H), 7.38 – 7.34 (m, 3H), 6.85 (d, *J* = 2.8 Hz, 1H), 6.79 (dd, *J* = 9.0, 2.9 Hz, 1H), 3.78 (s, 2H), 3.75 (s, 3H). ^{13}C NMR (101 MHz, $CDCl_3$) δ 169.0, 156.4, 134.3, 129.8, 129.4, 127.9, 127.9, 124.2, 122.9, 114.6, 113.2, 55.8, 45.1. HRMS (ESI): m/z $[M+H]^+$ calcd for $C_{15}H_{15}NO_2Cl$: 276.0791; found: 276.0796.

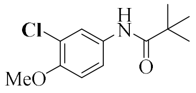

***N*-(3-chloro-4-methoxyphenyl)pivalamide (76)** was synthesized using 24.6 mg of 4-methoxyaniline, 26.0 μL of pivaloyl chloride and following general procedure A using 2 mA constant current for 16 h. Compound **76** was isolated using automated flash chromatography (silica gel, gradient elution 5-30% EtOAc in isohexane) as a white solid (34.5 mg, 72% yield). ^{1}H NMR (400 MHz, $CDCl_3$) δ 8.20 (d, *J* = 9.1 Hz, 1H), 7.76 (s, 1H), 6.91 (d, *J* = 2.9 Hz, 1H), 6.81 (dd, *J* = 9.1, 2.8 Hz, 1H), 3.76 (s, 3H), 1.32 (s, 9H). ^{13}C NMR (101 MHz, $CDCl_3$) δ 176.5, 156.2, 128.2, 124.3, 123.0, 114.5, 113.2, 55.8, 40.0, 27.7. HRMS (ESI): m/z $[M+H]^+$ calcd for $C_{12}H_{17}NO_2Cl$: 242.0948; found: 242.0942.

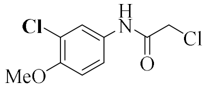

2-chloro-*N*-(3-chloro-4-methoxyphenyl)acetamide (77) was synthesized using 24.6 mg of 4-methoxyaniline, 17.0 μL of choloacetyl chloride and following general procedure A using 2 mA constant current for 16 h. Compound **77** was isolated using automated flash chromatography (silica gel, gradient elution 5-30% EtOAc in isohexane) as a white solid (38 mg, 82% yield). ^{1}H NMR (400 MHz, $CDCl_3$) δ 8.14 (s, 1H), 7.60 (d, *J* = 2.6 Hz, 1H), 7.41 (dd, *J* = 8.9, 2.7 Hz, 1H), 6.90 (d, *J* = 8.9 Hz, 1H), 4.18 (s, 2H), 3.89 (s, 3H). ^{13}C NMR (101 MHz, $CDCl_3$) δ 163.9, 152.7, 130.2, 122.9, 122.8, 120.1, 112.3, 56.5, 42.9.

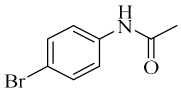

***N*-(4-bromophenyl)acetamide (78)** was synthesized using 18.6 mg of aniline, 15.5 μL of acetyl bromide and following general procedure A using 15 mA constant current for 4 h. Compound **78** was isolated using automated flash chromatography (silica gel, gradient elution 30-70% EtOAc in isohexane) as a white solid (39 mg, 92% yield). ^{1}H NMR (400 MHz, $CDCl_3$) δ 7.46 – 7.37 (m, 4H), 7.34 (s, 1H), 2.17 (s, 3H). ^{13}C NMR (101 MHz, $CDCl_3$) δ 168.4, 137.1, 132.1, 121.5, 117.0, 24.7.

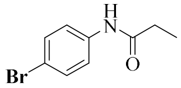

***N*-(4-bromophenyl)propionamide (79)** was synthesized using 18.6 mg of aniline, 19.0 μL of propionyl bromide and following general procedure A using 15 mA constant current for 6 h. Compound **79** was isolated using automated flash chromatography (silica gel, gradient elution 20-40% EtOAc in isohexane) as a white solid (43 mg, 95% yield). ^{1}H NMR (400 MHz, $CDCl_3$) δ 7.46 (s, 1H), 7.40 (s, 4H), 2.37 (q, J = 7.5 Hz, 2H), 1.22 (t, J = 7.6 Hz, 3H). ^{13}C NMR (101 MHz, $CDCl_3$) δ 172.3, 137.2, 132.0, 121.5, 116.8, 30.8, 9.7.

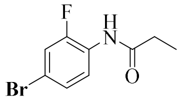

***N*-(4-bromo-2-fluorophenyl)propionamide (80)** was synthesized using 22.2 mg of 2-fluoroaniline, 19.0 μL of propionyl bromide and following general procedure A using 15 mA constant current for 6 h. Compound **80** was isolated using automated flash chromatography (silica gel, gradient elution 20-40% EtOAc in isohexane) as a white solid (35.5 mg, 73% yield). ^{1}H NMR (400 MHz, $CDCl_3$) δ 8.27 (t, J = 8.5 Hz, 1H), 7.30 – 7.22 (m, 3H), 2.44 (q, J = 7.5 Hz, 2H), 1.26 (t, J = 7.5 Hz, 3H). ^{13}C NMR (101 MHz, $CDCl_3$) δ 172.0, 152.0 (d, J = 246.0 Hz), 127.9 (d, J = 3.7 Hz), 125.9 (d, J = 9.4 Hz), 122.7, 118.5 (d, J = 22.3 Hz), 115.6, 31.0, 9.6. ^{19}F NMR (376 MHz, $CDCl_3$) δ -129.09 (q, J = 6.1 Hz). HRMS (ESI): m/z $[M+H+ACN]^+$ calcd for $C_{11}H_{13}N_2OFBr$: 287.0189; found: 287.0195.

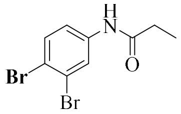

***N*-(3,4-dibromophenyl)propionamide (81)** was synthesized using 34 mg of 3-bromoaniline, 19.0 μL of propionyl bromide and following general procedure A using 15 mA constant current for 4 h. Compound **81** was isolated using automated flash chromatography (silica gel, gradient elution 20-40% EtOAc in isohexane) as an off-white solid (54 mg, 89% yield). ^{1}H NMR (400 MHz, $CDCl_3$) δ 7.89 (d, J = 2.5 Hz, 1H), 7.58 (s, 1H), 7.49 (d, J = 8.7 Hz, 1H), 7.32 (dd, J = 8.7, 2.5 Hz, 1H), 2.38 (q, J = 7.5 Hz, 2H), 1.22 (t, J = 7.5 Hz, 3H). ^{13}C NMR (101 MHz, $CDCl_3$) δ 172.5, 138.2, 133.7, 125.0, 124.8, 120.1, 119.1, 30.8, 9.6. HRMS (ESI): m/z $[M+H+ACN]^+$ calcd for $C_{11}H_{13}N_2OBr_2$: 346.9395; found: 346.9410.

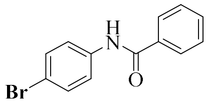

***N*-(4-bromophenyl)benzamide (82)** was synthesized using 18.6 mg of aniline, 25.0 μL of benzoyl bromide and following general procedure A using 15 mA constant current for 3.0 h. Compound **82** was

isolated using automated flash chromatography (silica gel, gradient elution 20-40% EtOAc in isohexane) as a white solid (44.5 mg, 81% yield). ^{1}H NMR (400 MHz, DMSO-d$_6$) δ 10.37 (s, 1H), 7.95 (dt, *J* = 7.0, 1.4 Hz, 2H), 7.80 – 7.75 (m, 2H), 7.63 – 7.58 (m, 1H), 7.57 – 7.50 (m, 4H). ^{13}C NMR (101 MHz, DMSO-d$_6$) δ 165.7, 138.6, 134.7, 131.7, 131.4, 128.4, 127.7, 122.2, 115.3.

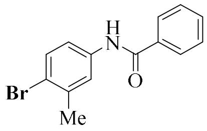

***N*-(4-bromo-3-methylphenyl)benzamide (83)** was synthesized using 21.4 mg of *m*-toluidine, 25.0 μL of benzoyl bromide and following general procedure A using 15 mA constant current for 2.1 h. Compound **83** was isolated using automated flash chromatography (silica gel, gradient elution 20-40% EtOAc in isohexane) as a white solid (51 mg, 88% yield). ^{1}H NMR (400 MHz, $CDCl_3$) δ 8.17 (s, 1H), 7.85 – 7.80 (m, 2H), 7.56 – 7.49 (m, 2H), 7.45 – 7.38 (m, 3H), 7.32 (dd, *J* = 8.6, 2.5 Hz, 1H), 2.33 (s, 3H). ^{13}C NMR (101 MHz, $CDCl_3$) δ 166.1, 138.6, 137.2, 134.7, 132.7, 132.0, 128.8, 127.2, 122.8, 119.9, 119.5, 23.1.

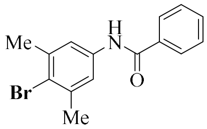

***N*-(4-bromo-3,5-dimethylphenyl)benzamide (84)** was synthesized using 24.2 mg of 3,5-dimethylaniline, 25.0 μL of benzoyl bromide and following general procedure A using 15 mA constant current for 2 h. Compound **84** was isolated using automated flash chromatography (silica gel, gradient elution 20-40% EtOAc in isohexane) as a white solid (50 mg, 83% yield). ^{1}H NMR (400 MHz, $CDCl_3$) δ 7.87 – 7.82 (m, 2H), 7.78 (s, 1H), 7.58 – 7.52 (m, 1H), 7.50 – 7.45 (m, 2H), 7.40 (s, 2H), 2.41 (s, 6H). ^{13}C NMR (101 MHz, $CDCl_3$) δ 165.8, 139.1, 136.5, 134.9, 132.1, 128.9, 127.1, 122.7, 120.0, 24.1. HRMS (ESI): m/z $[M+H]^+$ calcd for $C_{15}H_{15}NOBr$: 304.0337; found: 304.0344.

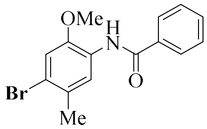

***N*-(4-bromo-2-methoxy-5-methylphenyl)benzamide (85)** was synthesized using 27.4 mg of 2-methoxy-5-methylaniline, 25.0 μL of benzoyl bromide and following general procedure A using 15 mA constant current for 3 h. Compound **85** was isolated using automated flash chromatography (silica gel, gradient elution 20-40% EtOAc in isohexane) as a light brown solid (41 mg, 64% yield). ^{1}H NMR (400 MHz, $CDCl_3$) δ 8.47 (s, 1H), 8.43 (s, 1H), 7.90 – 7.86 (m, 2H), 7.56 – 7.47 (m, 3H), 7.06 (s, 1H), 3.90 (s, 3H), 2.38 (s, 3H). ^{13}C NMR (101 MHz, $CDCl_3$) δ 165.3, 146.9, 135.1, 132.0, 130.4, 128.9, 127.1,

127.0, 121.6, 118.2, 114.1, 56.3, 22.5. HRMS (ESI): m/z $[M+H]^+$ calcd for $C_{15}H_{15}NO_2Br$: 320.0286; found: 320.0296.

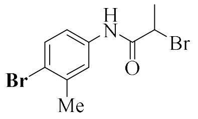

2-Bromo-*N*-(4-bromo-3-methylphenyl)propenamide (86) was synthesized using 21.4 mg of *m*-toluidine, 22.0 µL of 2-bromopropanoyl bromide and following general procedure A using 15 mA constant current for 4 h. Compound **86** was isolated using automated flash chromatography (silica gel, gradient elution 10-40% EtOAc in isohexane) as a white solid (57.4 mg, 90% yield). ^{1}H NMR (400 MHz, DMSO-d$_6$) δ 10.38 (s, 1H), 7.60 (d, *J* = 2.5 Hz, 1H), 7.51 (d, *J* = 8.7 Hz, 1H), 7.38 (dd, *J* = 8.6, 2.6 Hz, 1H), 4.67 (q, *J* = 6.7 Hz, 1H), 2.32 (s, 3H), 1.74 (d, *J* = 6.7 Hz, 3H). ^{13}C NMR (101 MHz, DMSO-d$_6$) δ 167.6, 138.1, 137.6, 132.3, 121.6, 118.8, 118.0, 44.3, 22.6, 21.3. HRMS (ESI): m/z $[M+H+ACN]^+$ calcd for $C_{12}H_{15}N_2OBr_2$: 360.9551; found: 360.9541.

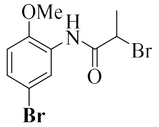

2-Bromo-*N*-(5-bromo-2-methoxyphenyl)propanamide (87) was synthesized using 24.6 mg of 2-methoxyaniline, 22.0 µL of 2-bromopropanoyl bromide and following general procedure A using 15 mA constant current for 3 h. Compound **87** was isolated using automated flash chromatography (silica gel, gradient elution 10-40% EtOAc in isohexane) as a white solid (50 mg, 75% yield). ^{1}H NMR (400 MHz, $CDCl_3$) δ 8.70 – 8.59 (m, 1H), 8.53 (d, *J* = 2.4 Hz, 1H), 7.18 (dd, *J* = 8.7, 2.4 Hz, 1H), 6.75 (d, *J* = 8.7 Hz, 1H), 4.54 (q, *J* = 7.0 Hz, 1H), 3.90 (s, 3H), 1.95 (d, *J* = 7.0 Hz, 3H). ^{13}C NMR (101 MHz, $CDCl_3$) δ 167.2, 147.4, 128.3, 127.0, 122.4, 113.5, 111.5, 56.3, 45.3, 23.1. HRMS (ESI): m/z $[M+H]^+$ calcd for $C_{10}H_{12}NO_2Br_2$: 337.9215; found: 337.9302.

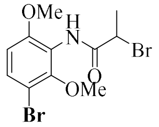

2-Bromo-N-(3-bromo-2,6-dimethoxyphenyl)propanamide (88) was synthesized using 30.6 mg of 2,6-dimethoxyaniline, 22.0 µL of 2-bromopropanoyl bromide and following general procedure A using 15 mA constant current for 3 h. Compound **88** was isolated using automated flash chromatography (silica gel, gradient elution 10-40% EtOAc in isohexane) as a white solid (57 mg, 78% yield). ^{1}H NMR (400 MHz, $CDCl_3$) δ 8.51 (s, 1H), 8.44 (s, 1H), 6.49 (s, 1H), 4.54 (q, *J* = 7.0 Hz, 1H), 3.91 (s, 3H), 3.87 (s, 3H), 1.95 (d, *J* = 7.0 Hz, 3H). ^{13}C NMR (101 MHz, $CDCl_3$) δ 166.8, 152.9, 148.9, 124.1, 121.2, 102.0, 96.4, 56.8, 56.3, 45.5, 23.1. HRMS (ESI): m/z $[M+H]^+$ calcd for $C_{11}H_{14}NO_3Br_2$: 367.9320; found: 367.9325.

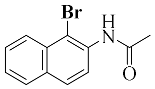

***N*-(1-bromonaphthalen-2-yl)acetamide (89)** was synthesized using 28.6 mg of naphthalen-2-amine, 15.5 μL of acetyl bromide and following general procedure A using 15 mA constant current for 2 h. Compound **89** was isolated using automated flash chromatography (silica gel, gradient elution 10-40% EtOAc in isohexane) as a white solid (49.4 mg, 94% yield). ^{1}H NMR (400 MHz, $CDCl_3$) δ 8.43 (d, *J* = 8.9 Hz, 1H), 8.15 (d, *J* = 8.5 Hz, 1H), 7.89 (s, 1H), 7.79 (d, *J* = 8.6 Hz, 2H), 7.57 (ddd, *J* = 8.4, 6.8, 1.3 Hz, 1H), 7.45 (ddd, *J* = 8.1, 6.8, 1.2 Hz, 1H), 2.29 (s, 3H). ^{13}C NMR (101 MHz, $CDCl_3$) δ 168.6, 134.4, 132.0, 131.7, 128.4, 128.3, 127.8, 126.7, 125.7, 121.1, 111.7, 25.0.

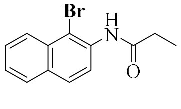

***N*-(1-bromonaphthalen-2-yl)propionamide (90)** was synthesized using 28.6 mg of 2-naphthylamine, 19.0 μL of propionyl bromide and following general procedure A using 15 mA constant current for 3 h. Compound **90** was isolated using automated flash chromatography (silica gel, gradient elution 10-40% EtOAc in isohexane) as a white solid (50.4 mg, 91% yield). ^{1}H NMR (400 MHz, $CDCl_3$) δ 8.50 (d, *J* = 9.0 Hz, 1H), 8.16 (dd, *J* = 8.5, 1.0 Hz, 1H), 7.92 (s, 1H), 7.85 – 7.74 (m, 2H), 7.57 (ddd, *J* = 8.4, 6.9, 1.3 Hz, 1H), 7.46 (ddd, *J* = 8.1, 6.9, 1.2 Hz, 1H), 2.54 (q, *J* = 7.6 Hz, 2H), 1.33 (t, *J* = 7.6 Hz, 3H). ^{13}C NMR (101 MHz, $CDCl_3$) δ 172.3, 134.5, 132.1, 131.6, 128.5, 128.3, 127.8, 126.7, 125.6, 121.0, 111.5, 31.3, 9.8.

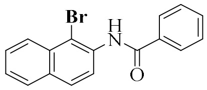

***N*-(1-bromonaphthalen-2-yl)benzamide (91)** was synthesized using 28.6 mg of 2-naphthylamine, 25.0 μL of benzoyl bromide and following general procedure A using 15 mA constant current for 2 h. Compound **91** was isolated using automated flash chromatography (silica gel, gradient elution 10-40% EtOAc in isohexane) as a white solid (57 mg, 88% yield). ^{1}H NMR (400 MHz, $CDCl_3$) δ 8.76 (s, 1H), 8.69 (d, *J* = 9.0 Hz, 1H), 8.19 (dd, *J* = 8.5, 1.0 Hz, 1H), 8.03 – 7.99 (m, 2H), 7.90 – 7.82 (m, 2H), 7.62 – 7.53 (m, 4H), 7.48 (ddd, J = 8.1, 6.8, 1.1 Hz, 1H). ^{13}C NMR (101 MHz, $CDCl_3$) δ 165.6, 134.8, 134.6, 132.4, 132.1, 131.8, 129.1, 128.6, 128.4, 128.0, 127.4, 126.7, 125.7, 120.8, 112.1.

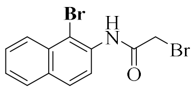

2-bromo-*N*-(1-bromonaphthalen-2-yl)acetamide (92) was synthesized using 28.6 mg of 2-naphthylamine, 18.3 μL of 2-bromoacetyl bromide and following general procedure A using 15 mA constant current for 6.5 h. Compound **92** was isolated using automated flash chromatography (silica

gel, gradient elution 10-40% EtOAc in isohexane) as a white solid (55.2 mg, 81% yield). ^{1}H NMR (400 MHz, $CDCl_3$) δ 9.07 (s, 1H), 8.44 (d, *J* = 9.0 Hz, 1H), 8.20 (dd, *J* = 8.5, 1.1 Hz, 1H), 7.87 – 7.77 (m, 2H), 7.60 (ddd, *J* = 8.4, 6.9, 1.3 Hz, 1H), 7.50 (ddd, *J* = 8.1, 6.9, 1.2 Hz, 1H), 4.13 (s, 2H). ^{13}C NMR (101 MHz, $CDCl_3$) δ 163.8, 133.7, 132.1, 132.0, 128.6, 128.3, 128.1, 126.9, 126.1, 120.2, 112.7, 29.9. HRMS (ESI): m/z $[M+H]^+$ calcd for $C_{12}H_{10}NOBr_2$: 343.9109; found: 343.9100.

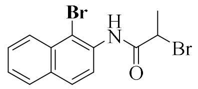

2-bromo-*N*-(1-bromonaphthalen-2-yl)propanamide (93) was synthesized using 28.6 mg of 2-naphthylamine, 22.0 μL of 2-bromopropanoyl bromide and following general procedure A using 15 mA constant current for 3.0 h. Compound **93** was isolated using automated flash chromatography (silica gel, gradient elution 10-40% EtOAc in isohexane) as a white solid (63.9 mg, 90% yield). ^{1}H NMR (400 MHz, $CDCl_3$) δ 8.95 (s, 1H), 8.43 (d, *J* = 9.0 Hz, 1H), 8.21 – 8.17 (m, 1H), 7.87 – 7.78 (m, 2H), 7.60 (ddd, *J* = 8.4, 6.8, 1.3 Hz, 1H), 7.49 (ddd, *J* = 8.1, 6.9, 1.2 Hz, 1H), 4.66 (q, *J* = 7.1 Hz, 1H), 2.04 (d, *J* = 7.0 Hz, 3H). ^{13}C NMR (101 MHz, $CDCl_3$) δ 167.8, 133.8, 132.1, 132.0, 128.6, 128.3, 128.0, 126.9, 126.0, 120.4, 112.8, 45.7, 23.3. LC-MS: m/z $[M+H]^+$ calcd for $C_{13}H_{12}NOBr_2$: 357.9; found: 357.9.

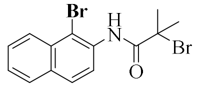

2-bromo-*N*-(1-bromonaphthalen-2-yl)-2-methylpropanamide (94) was synthesized using 28.6 mg of 2-naphthylamine, 26.0 μL of α-bromoisobutyryl bromide and following general procedure A using 15 mA constant current for 3.0 h. Compound **94** was isolated using automated flash chromatography (silica gel, gradient elution 10-40% EtOAc in isohexane) as a white solid (63.5 mg, 86% yield). ^{1}H NMR (400 MHz, $CDCl_3$) δ 9.30 (s, 1H), 8.43 (d, *J* = 9.0 Hz, 1H), 8.20 (dq, *J* = 8.6, 0.9 Hz, 1H), 7.86 – 7.80 (m, 2H), 7.60 (ddd, *J* = 8.4, 6.9, 1.3 Hz, 1H), 7.52 – 7.47 (m, 1H), 2.12 (s, 6H).^{13}C NMR (101 MHz, $CDCl_3$) δ 170.6, 134.2, 132.1, 131.9, 128.5, 128.3, 128.0, 126.9, 125.9, 120.4, 112.9, 62.7, 32.7. HRMS (ESI): m/z $[M+H]^+$ calcd for $C_{14}H_{14}NOBr_2$: 369.9442; found: 369.9444.

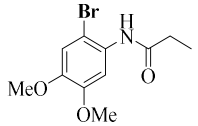

***N*-(2-bromo-4,5-dimethoxyphenyl)propionamide (95)** was synthesized using 30.6 mg of 3,4-dimethoxyaniline, 19.0 μL of propionyl bromide and following general procedure A using 15 mA constant current for 3 h. Compound **95** was isolated using automated flash chromatography (silica gel, gradient elution 10-40% EtOAc in isohexane) as an off-white solid (50 mg, 80% yield). ^{1}H NMR (400 MHz, $CDCl_3$) δ 8.06 (s, 1H), 7.45 (s, 1H), 6.97 (s, 1H), 3.88 (s, 3H), 3.84 (s, 3H), 2.45 (q, *J* = 7.5 Hz, 2H), 1.27 (t, *J* = 7.5 Hz, 3H). ^{13}C NMR (101 MHz, $CDCl_3$) δ 172.0, 148.7, 145.9, 129.6, 114.6, 105.8,

102.7, 56.4, 56.2, 31.0, 9.7. HRMS (ESI): m/z $[M+H]^+$ calcd for $C_{11}H_{15}NO_3Br$: 288.0235; found: 288.0229.

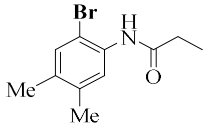

***N*-(2-bromo-4,5-dimethylphenyl)propionamide (96)** was synthesized using 24.2 mg of 3,4-dimethylaniline, 19.0 μL of propionyl bromide and following general procedure A using 15 mA constant current for 3 h. Compound **96** was isolated using automated flash chromatography (silica gel, gradient elution 10-40% EtOAc in isohexane) as a white solid (43.3 mg, 85% yield). ^{1}H NMR (400 MHz, $CDCl_3$) δ 8.13 (s, 1H), 7.48 (s, 1H), 7.28 (s, 1H), 2.45 (q, *J* = 7.5 Hz, 2H), 2.22 (s, 3H), 2.20 (s, 3H), 1.27 (t, *J* = 7.5 Hz, 3H). ^{13}C NMR (101 MHz, $CDCl_3$) δ 171.9, 137.2, 134.1, 133.3, 132.6, 123.1, 110.1, 31.1, 19.8, 19.2, 9.8. HRMS (ESI): m/z $[M+H]^+$ calcd for $C_{11}H_{15}NOBr$: 256.0337; found: 256.0335.

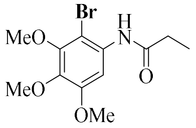

***N*-(2-bromo-3,4,5-trimethoxyphenyl)propionamide (97)** was synthesized using 36.6 mg of 3,4,5-trimethoxyaniline, 19.0 μL of propionyl bromide and following general procedure A using 15 mA constant current for 5 h. Compound **97** was isolated using automated flash chromatography (silica gel, gradient elution 10-40% EtOAc in isohexane) as an off-white solid (35 mg, 55% yield). ^{1}H NMR (400 MHz, $CDCl_3$) δ 7.97 (s, 1H), 7.63 (s, 1H), 3.89 (s, 3H), 3.86 (s, 3H), 3.83 (s, 3H), 2.45 (q, *J* = 7.6 Hz, 2H), 1.26 (t, *J* = 7.5 Hz, 3H). ^{13}C NMR (101 MHz, $CDCl_3$) δ 172.2, 153.1, 150.6, 139.3, 132.2, 101.2, 99.5, 61.3, 61.2, 56.2, 31.2, 9.6. HRMS (ESI): m/z $[M+H]^+$ calcd for $C_{12}H_{17}NO_4Br$: 318.0341; found: 318.0347.

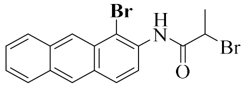

2-Bromo-*N*-(1-bromoanthracen-2-yl)propenamide (98) was synthesized using 38.6 mg of anthracen-2-amine, 22.0 μL of 2-bromopropanoyl bromide and following general procedure A using 15 mA constant current for 5 h. Compound **98** was isolated using automated flash chromatography (silica gel, gradient elution 10-40% EtOAc in isohexane) as a yellow solid (45.4 mg, 56% yield). ^{1}H NMR (400 MHz, DMSO-d_6) δ 10.79 (s, 1H), 8.98 (d, *J* = 2.0 Hz, 1H), 8.67 (s, 1H), 8.37 (dd, *J* = 8.8, 1.1 Hz, 1H), 8.15 (dd, *J* = 9.0, 7.3 Hz, 2H), 7.77 – 7.66 (m, 2H), 7.57 (ddd, *J* = 8.1, 6.6, 1.1 Hz, 1H), 4.81 (q, *J* = 6.6 Hz, 1H), 1.83 (d, *J* = 6.6 Hz, 3H). ^{13}C NMR (101 MHz, DMSO-d_6) δ 168.1, 137.8,

131.0, 130.5, 130.3, 130.0, 129.1, 129.0, 128.2, 127.3, 126.4, 125.5, 120.8, 119.9, 113.3, 44.4, 21.3. HRMS (ESI): m/z $[M+H]^+$ calcd for $C_{17}H_{14}NOBr_2$: 405.9442; found: 405.9422.

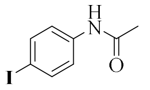

***N*-(4-iodophenyl)acetamide (99)** was synthesized using 18.6 mg of aniline, 20.0 μL of acetyl iodide and following general procedure A using 100 mA constant current for 45 h. Compound **99** was isolated using automated flash chromatography (silica gel, gradient elution 40-70% EtOAc in isohexane) as a white solid (17.5 mg, 34% yield). ^{1}H NMR (400 MHz, $CDCl_3$) δ 7.61 (d, *J* = 8.6 Hz, 2H), 7.29-7.25 (m, 3H), 2.16 (s, 3H). ^{13}C NMR (101 MHz, $CDCl_3$) δ 168.4, 138.1, 137.8, 121.8, 87.6, 24.8.

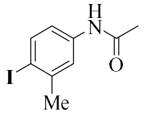

***N*-(4-iodo-3-methylphenyl)acetamide (100)** was synthesized using 21.4 mg of *m*-toluidine, 20.0 μL of acetyl iodide and following general procedure A using 100 mA constant current for 45 h. Compound **100** was isolated using automated flash chromatography (silica gel, gradient elution 40-70% EtOAc in isohexane) as a pale brown solid (22.5 mg, 41% yield). ^{1}H NMR (400 MHz, $CDCl_3$) δ 7.45 (d, *J* = 2.7 Hz, 1H), 7.34 (d, *J* = 2.0 Hz, 1H), 7.18 (t, *J* = 7.8 Hz, 1H), 6.91 (d, *J* = 7.5 Hz, 1H), 2.31 (s, 3H), 2.15 (s, 3H).^{13}C NMR (101 MHz, $CDCl_3$) δ 168.6, 139.2, 139.0, 128.9, 125.3, 120.8, 117.2, 28.3, 24.7.

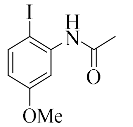

***N*-(2-iodo-5-methoxyphenyl)acetamide (101)** was synthesized using 24.6 mg of 3-methoxyaniline, 20.0 μL of acetyl iodide and following general procedure A using 100 mA constant current for 45 h. Compound **101** was isolated using automated flash chromatography (silica gel, gradient elution 40-70% EtOAc in isohexane) as a pale brown solid (20.0 mg, 35% yield). ^{1}H NMR (400 MHz, $CDCl_3$) δ 7.57 (d, *J* = 8.4 Hz, 1H), 7.39 (d, *J* = 2.3 Hz, 1H), 7.09 (s, 1H), 6.56 (dd, *J* = 8.4, 2.3 Hz, 1H), 3.81 (s, 3H), 2.11 (s, 3H). ^{13}C NMR (101 MHz, $CDCl_3$) δ 168.4, 158.7, 139.7, 139.2, 120.7, 113.2, 103.3, 56.5, 25.0.

General procedure B: Electrochemical cascade amidation/dihalogenation

Scheme S4. Electrochemical cascade amidation/di-halogenation

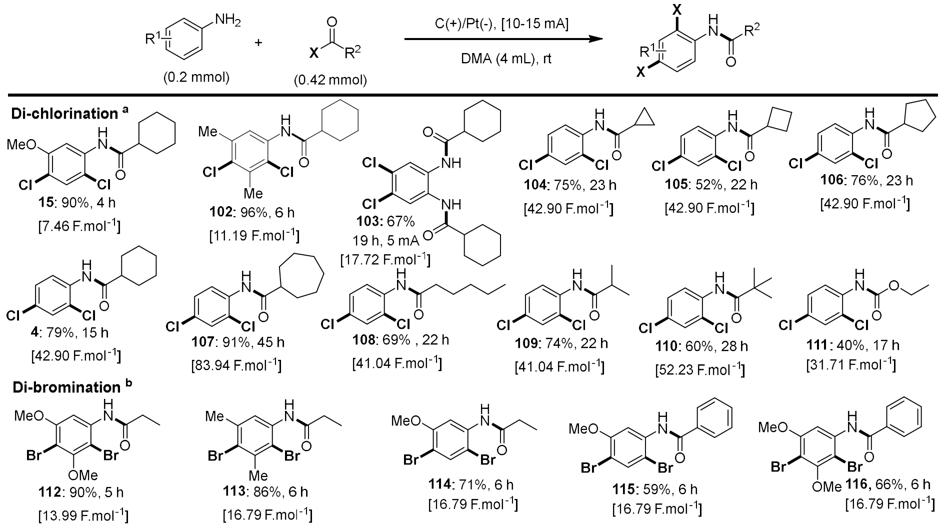

Reagents and conditions: [a] Amine (0.2 mmol) and acid chloride (0.42 mmol) in DMA (4 mL), in an undivided cell with carbon cloth (anode) and platinum (cathode); 10 mA current; [b] Amine (0.2 mmol) and acid bromide (0.42 mmol) in DMA (4 mL), in an undivided cell with carbon cloth (anode) and platinum (cathode), 15 mA current. All yields refer to isolated yields.

The electrolysis was carried out in 10 mL microwave reaction vial in an undivided cell setup under air. A carbon cloth anode (15 mm × 10 mm × 410 μm) and a platinum (Pt) cathode (25 mm × 10 mm × 0.125 mm) with electrode holders made of stainless steel were used and the distance between two electrodes was 10 mm. The cell was charged with the amine (0.20 mmol, 1.0 equiv.) and a teflon-coated magnetic stirring bar (15 × 6 mm). 4 mL of DMA was added and the reaction mixture was stirred at 25 °C for 5 min to obtain a homogeneous solution, then acid halide (0.42 mmol, 2.1 equiv.) was added. Subsequently, the electrolysis was performed at room temperature with a constant current of 10.0-15.0 mA maintained for 18 h (unless otherwise stated) with a stirring rate of 600 rpm. After completion of the reaction, the reaction mixture was diluted with 2 mL ethyl acetate and transferred to a round bottom flask. The electrodes (carbon cloth and platinum) were washed in the reaction flask with ethyl acetate (3 × 5 mL) in an ultrasonic cleaner (3 × 3 min) and the washes were combined in the round bottom flask. The reaction mixture was then transferred to a separating funnel and washed with brine (15 mL). The aqueous layer was washed with ethyl acetate (3 x 15 mL) and the combined organic layer was washed with brine (5 x 30 mL). The organic phase was dried with sodium sulfate and the solvent was removed under vacuum to obtain crude product. The crude products were purified by flash column chromatography using isohexane and ethyl acetate mixture (20:1 to 3:1) to afford the title compounds.

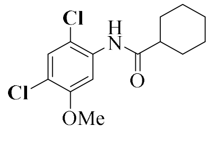

***N*-(2,4-dichloro-5-methoxyphenyl)cyclohexanecarboxamide (15)** was synthesized using 24.6 mg of 3-methoxyaniline, 56.0 µL of cyclohexanecarbonyl chloride and following general procedure B using 10 mA constant current for 4 h. Compound **15** was isolated using automated flash chromatography (silica gel, gradient elution 5-30% EtOAc in isohexane) as a white solid (54 mg, 90% yield). ^{1}H NMR (400 MHz, $CDCl_3$) δ 8.30 (d, *J* = 1.5 Hz, 1H), 7.73 – 7.64 (m, 1H), 7.35 (s, 1H), 3.91 (s, 3H), 2.31 (tt, *J* = 11.6, 3.5 Hz, 1H), 2.05 – 1.97 (m, 2H), 1.86 (dt, *J* = 12.6, 3.3 Hz, 2H), 1.73 (dtd, *J* = 10.5, 3.2, 1.5 Hz, 1H), 1.60 – 1.48 (m, 3H), 1.39 – 1.26 (m, 2H). ^{13}C NMR (101 MHz, $CDCl_3$) δ 174.6, 154.4, 134.4, 129.3, 117.0, 113.3, 105.0, 56.5, 46.8, 29.7, 25.7, 25.7. HRMS (ESI): m/z $[M+H]^+$ calcd for $C_{14}H_{18}NO_2Cl_2$: 302.0715; found: 302.0701.

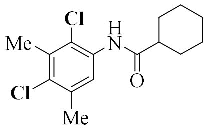

***N*-(2,4-dichloro-3,5-dimethylphenyl)cyclohexanecarboxamide (102)** was synthesized using 24.2 mg of 3,5-dimethylaniline, 56.0 µL of cyclohexanecarbonyl chloride and following general procedure B using 10 mA constant current for 6 h. Compound **102** was isolated using automated flash chromatography (silica gel, gradient elution 5-30% EtOAc in isohexane) as a white solid (57 mg, 96% yield). ^{1}H NMR (400 MHz, $CDCl_3$) δ 8.22 (s, 1H), 7.69 (s, 1H), 2.48 (s, 3H), 2.35 (s, 3H), 2.33 – 2.26 (m, 1H), 2.03 – 1.97 (m, 2H), 1.85 (dt, *J* = 12.1, 3.1 Hz, 2H), 1.74 – 1.69 (m, 1H), 1.54 (qd, *J* = 12.2, 3.3 Hz, 2H), 1.39 – 1.24 (m, 3H). ^{13}C NMR (101 MHz, $CDCl_3$) δ 174.4, 135.6, 134.1, 132.9, 130.1, 121.3, 120.7, 46.8, 29.8, 25.8, 25.8, 21.2, 18.7. HRMS (ESI): m/z $[M+H]^+$ calcd for $C_{15}H_{20}NOCl_2$: 300.0922; found: 300.0923.

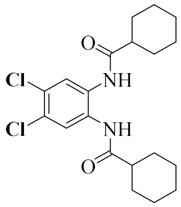

***N,N′*-(4,5-dichloro-1,2-phenylene)dicyclohexanecarboxamide (103)** was synthesized using 21.6 mg of benzene-1,2-diamine, 56.0 µL of cyclohexanecarbonyl chloride and following general procedure B using 5 mA constant current for 19 h. Compound **103** was isolated using automated flash chromatography (silica gel, gradient elution 5-30% EtOAc in isohexane) as a pale brownish solid (53 mg, 67% yield). ^{1}H NMR (400 MHz, DMSO-*d*$_6$) δ 9.36 (s, 2H), 7.81 (s, 2H), 2.37 (tt, *J* = 11.4, 3.5 Hz, 2H), 1.84 (dd, *J* = 12.2, 3.5 Hz, 4H), 1.75 (dt, *J* = 11.5, 3.3 Hz, 4H), 1.68 – 1.62 (m, 2H), 1.40 (qd, *J* = 12.1, 2.8 Hz, 4H), 1.26 (dddd, *J* = 28.5, 15.2, 7.4, 3.0 Hz, 6H). ^{13}C NMR (101 MHz, DMSO-*d*$_6$) δ 174.7, 130.5, 126.0, 125.6, 44.4, 29.0, 25.4, 25.1. HRMS (ESI): m/z $[M+H]^+$ calcd for $C_{20}H_{27}N_2O_2Cl_2$: 397.1450; found: 397.1434.

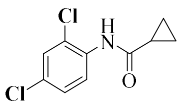

***N*-(2,4-dichlorophenyl)cyclopropanecarboxamide (104)** was synthesized using 18.6 mg of aniline, 34.0 μL of cyclopropanecarbonyl chloride and following general procedure B using 10 mA constant current for 23 h. Compound **104** was isolated using automated flash chromatography (silica gel, gradient elution 5-30% EtOAc in isohexane) as a white solid (34.2 mg, 75% yield). ^{1}H NMR (400 MHz, DMSO-d_6) δ 9.83 (s, 1H), 7.75 (d, *J* = 8.8 Hz, 1H), 7.65 (d, *J* = 2.4 Hz, 1H), 7.39 (dd, *J* = 8.7, 2.4 Hz, 1H), 2.01 (p, *J* = 6.2 Hz, 1H), 0.89 – 0.72 (m, 4H). ^{13}C NMR (101 MHz, DMSO-d_6) δ 172.2, 134.2, 129.0, 128.8, 127.4, 127.1, 13.9, 7.5. HRMS (ESI): m/z $[M+H]^+$ calcd for $C_{10}H_{10}NOCl_2$: 230.0139; found: 230.0147.

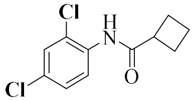

***N*-(2,4-dichlorophenyl)cyclobutanecarboxamide (105)** was synthesized using 18.6 mg of aniline, 48.0 μL of cyclobutanecarbonyl chloride and following general procedure B using 10 mA constant current for 22 h. Compound **105** was isolated using automated flash chromatography (silica gel, gradient elution 5-30% EtOAc in isohexane) as a white solid (25.0 mg, 52% yield). ^{1}H NMR (400 MHz, $CDCl_3$) δ 8.38 (d, *J* = 8.9 Hz, 1H), 7.50 (s, 1H), 7.35 (d, *J* = 2.4 Hz, 1H), 7.23 (dd, *J* = 8.9, 2.4 Hz, 1H), 3.22 (pd, *J* = 8.5, 1.0 Hz, 1H), 2.45 – 2.32 (m, 2H), 2.31 – 2.21 (m, 2H), 2.09 – 1.88 (m, 2H). ^{13}C NMR (101 MHz, $CDCl_3$) δ 173.3, 133.5, 128.9, 128.7, 128.0, 123.1, 122.2, 41.1, 25.4, 18.1. HRMS (ESI): m/z $[M+H]^+$ calcd for $C_{11}H_{12}NOCl_2$: 244.0296; found: 244.0296.

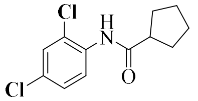

***N*-(2,4-dichlorophenyl)cyclopentanecarboxamide (106)** was synthesized using 18.6 mg of aniline, 51.0 μL of cyclopentanecarbonyl chloride and following general procedure B using 10 mA constant current for 23 h. Compound **106** was isolated using automated flash chromatography (silica gel, gradient elution 5-30% EtOAc in isohexane) as a white solid (39.0 mg, 76% yield). ^{1}H NMR (400 MHz, $CDCl_3$) δ 8.36 (d, *J* = 8.9 Hz, 1H), 7.62 (s, 1H), 7.35 (d, *J* = 2.4 Hz, 1H), 7.22 (dd, *J* = 8.9, 2.4 Hz, 1H), 2.76 (p, *J* = 8.1 Hz, 1H), 2.02 – 1.93 (m, 2H), 1.89 (ddt, *J* = 14.0, 7.2, 4.5 Hz, 2H), 1.82 – 1.70 (m, 2H), 1.69 – 1.59 (m, 2H). ^{13}C NMR (101 MHz, $CDCl_3$) δ 174.6, 133.7, 128.8, 128.7, 128.0, 123.1, 122.3, 47.2, 30.5, 26.0. HRMS (ESI): m/z $[M+H]^+$ calcd for $C_{12}H_{14}NOCl_2$: 258.0452; found: 258.0460.

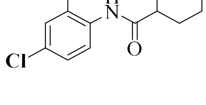

***N*-(2,4-dichlorophenyl)cyclohexanecarboxamide (4)** was synthesized using 18.6 mg of aniline, 56.0 μL of cyclohexanecarbonyl chloride and following general procedure B using 10 mA constant current for 15 h. Compound **4** was isolated using automated flash chromatography (silica gel, gradient elution 5-30% EtOAc in isohexane) as a white solid (42.5 mg, 79% yield). ^{1}H NMR (400 MHz, $CDCl_3$) δ 8.38 (d, *J* = 8.9 Hz, 1H), 7.64 (s, 1H), 7.37 (d, *J* = 2.4 Hz, 1H), 7.24 (dd, *J* = 8.9, 2.4 Hz, 1H), 2.30 (tt, *J* = 11.7, 3.5 Hz, 1H), 2.03 – 1.97 (m, 2H), 1.85 (dt, *J* = 13.1, 3.6 Hz, 2H), 1.72 (dddd, *J* = 11.2, 4.9, 2.9, 1.5 Hz, 1H), 1.52 (td, *J* = 12.3, 3.2 Hz, 2H), 1.37 – 1.25 (m, 3H). ^{13}C NMR (101 MHz, CDCl3) δ 174.4, 133.6, 128.9, 128.7, 128.0, 123.2, 122.3, 46.7, 29.8, 25.8, 25.7.

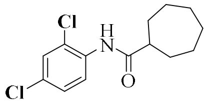

***N*-(2,4-dichlorophenyl)cycloheptanecarboxamide (107)** was synthesized using 18.6 mg of aniline, 62.0 μL of cycloheptanecarbonyl chloride and following general procedure B using 10 mA constant current for 45 h. Compound **107** was isolated using automated flash chromatography (silica gel, gradient elution 5-30% EtOAc in isohexane) as a white solid (51.8 mg, 91% yield). ^{1}H NMR (400 MHz, $CDCl_3$) δ 8.33 (d, *J* = 8.9 Hz, 1H), 7.58 (s, 1H), 7.35 (d, *J* = 2.4 Hz, 1H), 7.22 (dd, *J* = 8.9, 2.4 Hz, 1H), 2.50 – 2.42 (m, 1H), 2.05 – 1.96 (m, 2H), 1.84 – 1.72 (m, 4H), 1.62 – 1.46 (m, 6H). ^{13}C NMR (101 MHz, $CDCl_3$) δ 175.5, 133.7, 128.9, 128.7, 127.9, 123.3, 122.4, 48.6, 31.7, 28.3, 26.5. HRMS (ESI): m/z $[M+H]^+$ calcd for $C_{14}H_{18}NOCl_2$: 286.0765; found: 286.0764.

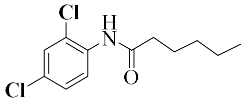

***N*-(2,4-dichlorophenyl)hexanamide (108)** was synthesized using 18.6 mg of aniline, 59.0 μL of hexanoyl chloride and following general procedure B using 10 mA constant current for 22 h. Compound **108** was isolated using automated flash chromatography (silica gel, gradient elution 5-30% EtOAc in isohexane) as a white solid (35.5 mg, 69% yield). ^{1}H NMR (400 MHz, $CDCl_3$) δ 8.34 (d, *J* = 8.9 Hz, 1H), 7.58 (s, 1H), 7.35 (d, *J* = 2.4 Hz, 1H), 7.22 (dd, *J* = 8.9, 2.4 Hz, 1H), 2.41 (t, *J* = 7.6 Hz, 2H), 1.77 – 1.68 (m, 2H), 1.36 (tt, *J* = 6.7, 3.1 Hz, 4H), 0.94 – 0.87 (m, 3H). ^{13}C NMR (101 MHz, $CDCl_3$) δ 171.5, 133.5, 129.0, 128.7, 128.0, 123.1, 122.4, 38.0, 31.4, 25.2, 22.5, 14.0. HRMS (ESI): m/z $[M+H]^+$ calcd for $C_{12}H_{16}NOCl_2$: 260.0609; found: 260.0613.

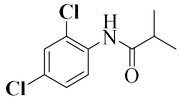

***N*-(2,4-dichlorophenyl)isobutyramide (109)** was synthesized using 18.6 mg of aniline, 44.0 μL of isobutyryl chloride and following general procedure B using 10 mA constant current for 22 h. Compound **109** was isolated using automated flash chromatography (silica gel, gradient elution 5-30% EtOAc in isohexane) as a white solid (34.0 mg, 74% yield). ^{1}H NMR (400 MHz, DMSO-d_6) δ 9.49 (s,

1H), 7.74 – 7.45 (m, 2H), 7.40 (ddd, *J* = 8.7, 2.4, 0.9 Hz, 1H), 2.73 (p, *J* = 6.8 Hz, 1H), 1.11 (d, *J* = 6.8 Hz, 6H). ^{13}C NMR (101 MHz, DMSO-d$_6$) δ 175.5, 134.2, 129.4, 128.8, 128.0, 127.7, 127.4, 34.2, 19.4.

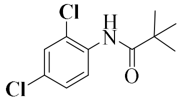

***N*-(2,4-dichlorophenyl)pivalamide (110)** was synthesized using 18.6 mg of aniline, 52.0 μL of pivaloyl chloride and following general procedure B using 10 mA constant current for 22 h. Compound **110** was isolated using automated flash chromatography (silica gel, gradient elution 5-30% EtOAc in isohexane) as an off-white solid (29.4 mg, 60% yield). ^{1}H NMR (400 MHz, $CDCl_3$) δ 8.37 (d, *J* = 8.9 Hz, 1H), 7.94 (s, 1H), 7.36 (d, *J* = 2.4 Hz, 1H), 7.25 – 7.22 (m, 1H), 1.33 (s, 9H). ^{13}C NMR (101 MHz, $CDCl_3$) δ 176.7, 133.7, 128.9, 128.7, 128.0, 123.5, 122.2, 40.3, 27.6.

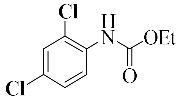

Ethyl (2,4-dichlorophenyl)carbamate (111) was synthesized using 18.6 mg of aniline, 41.0 μL of ethyl chloroformate and following general procedure B using 10 mA constant current for 17 h. Compound **111** was isolated using automated flash chromatography (silica gel, gradient elution 5-30% EtOAc in isohexane) as a white solid (18.6 mg, 40% yield). ^{1}H NMR (400 MHz, $CDCl_3$) δ 8.04 (d, *J* = 8.9 Hz, 1H), 7.26 (d, *J* = 2.4 Hz, 1H), 7.19 – 7.09 (m, 1H), 6.97 (s, 1H), 4.16 (q, *J* = 7.1 Hz, 2H), 1.24 (t, *J* = 7.1 Hz, 3H). ^{13}C NMR (101 MHz, $CDCl_3$) δ 153.2, 133.8, 128.8, 128.2, 128.0, 122.6, 120.7, 61.9, 14.6.

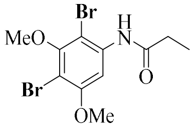

***N*-(2,4-dibromo-3,5-dimethoxyphenyl)propionamide (112)** was synthesized using 30.6 mg of 3,5-dimethoxyaniline, 38.0 μL of propionyl bromide and following general procedure B using 15 mA constant current for 5 h. Compound **112** was isolated using automated flash chromatography (silica gel, gradient elution 10-40% EtOAc in isohexane) as a white solid (65.7 mg, 90% yield). ^{1}H NMR (400 MHz, $CDCl_3$) δ 8.11 (s, 1H), 7.76 (s, 1H), 3.91 (s, 3H), 3.86 (s, 3H), 2.48 (q, *J* = 7.5 Hz, 2H), 1.28 (t, *J* = 7.5 Hz, 3H). ^{13}C NMR (101 MHz, $CDCl_3$) δ 172.3, 156.7, 154.8, 136.4, 101.9, 100.8, 99.9, 60.7, 56.8, 31.3, 9.5. HRMS (ESI): m/z $[M+H]^+$ calcd for $C_{11}H_{14}NO_3Br_2$: 367.9320; found: 367.9317.

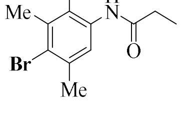

***N*-(2,4-dibromo-3,5-dimethylphenyl)propionamide (113)** was synthesized using 24.2 mg of 3,5-dimethylaniline, 38.0 μL of propionyl bromide and following general procedure B using 15 mA constant current for 6 h. Compound **113** was isolated using automated flash chromatography (silica gel, gradient elution 10-40% EtOAc in isohexane) as a white solid (57.2 mg, 86% yield). ^{1}H NMR (400 MHz, $CDCl_3$) δ 8.18 (s, 1H), 7.68 (s, 1H), 2.63 (s, 3H), 2.46 (q, *J* = 7.5 Hz, 2H), 2.39 (d, *J* = 0.7 Hz, 3H), 1.27 (t, *J* = 7.6 Hz, 3H). ^{13}C NMR (101 MHz, $CDCl_3$) δ 172.0, 138.4, 137.5, 134.6, 122.3, 121.0, 113.5, 31.2, 25.4, 24.5, 9.7. LC-MS: m/z $[M+H]^+$ calcd for $C_{11}H_{14}NOBr_2$: 335.9; found: 336.0.

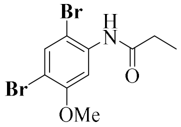

***N*-(2,4-dibromo-5-methoxyphenyl)propionamide (114)** was synthesized using 24.6 mg of 3-methoxyaniline, 38.0 μL of propionyl bromide and following general procedure B using 15 mA constant current for 6 h. Compound **114** was isolated using automated flash chromatography (silica gel, gradient elution 10-40% EtOAc in isohexane) as a white solid (47.6 mg, 71% yield). ^{1}H NMR (400 MHz, $CDCl_3$) δ 8.24 (s, 1H), 7.65 (s, 1H), 7.62 (s, 1H), 3.90 (s, 3H), 2.48 (q, *J* = 7.5 Hz, 2H), 1.27 (t, *J* = 7.5 Hz, 3H). ^{13}C NMR (101 MHz, $CDCl_3$) δ 172.3, 155.9, 136.1, 135.0, 105.9, 105.0, 102.9, 56.6, 31.2, 9.5. HRMS (ESI): m/z $[M+H+ACN]^+$ calcd for $C_{12}H_{15}N_2O_2Br_2$: 376.9500; found: 376.9499.

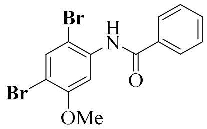

***N*-(2,4-dibromo-5-methoxyphenyl)benzamide (115)** was synthesized using 24.6 mg of 3-methoxyaniline, 36.0 μL of benzoyl bromide and following general procedure B using 15 mA constant current for 4 h. Compound **115** was isolated using automated flash chromatography (silica gel, gradient elution 10-40% EtOAc in isohexane) as a white solid (45.5 mg, 59% yield). ^{1}H NMR (400 MHz, $CDCl_3$) δ 8.47 (s, 1H), 8.42 (s, 1H), 7.94 – 7.89 (m, 2H), 7.70 (s, 1H), 7.63 – 7.58 (m, 1H), 7.55 – 7.51 (m, 2H), 3.96 (s, 3H). ^{13}C NMR (101 MHz, $CDCl_3$) δ 165.4, 156.0, 136.2, 135.0, 134.3, 132.6, 129.2, 127.2, 106.3, 105.0, 103.6, 56.7. HRMS (ESI): m/z $[M+H]^+$ calcd for $C_{14}H_{12}NO_2Br_2$: 385.9214; found: 385.9222.

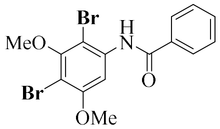

***N*-(2,4-dibromo-3,5-dimethoxyphenyl)benzamide (116)** was synthesized using 30.6 mg of 3,5-dimethoxyaniline, 36.0 μL of benzoyl bromide and following general procedure B using 15 mA constant current for 6 h. Compound **116** was isolated using automated flash chromatography (silica gel, gradient elution 10-40% EtOAc in isohexane) as a white solid (50 mg, 61% yield). ^{1}H NMR (400 MHz,

$CDCl_3$) δ 8.60 (s, 1H), 8.27 (s, 1H), 7.94 – 7.90 (m, 2H), 7.62 – 7.50 (m, 3H), 3.96 (s, 3H), 3.89 (s, 3H). ^{13}C NMR (101 MHz, $CDCl_3$) δ 165.5, 156.8, 154.9, 136.4, 134.3, 132.6, 129.2, 127.1, 102.3, 100.8, 100.5, 60.7, 56.8. HRMS (ESI): m/z $[M+H]^+$ calcd for $C_{15}H_{14}NO_3Br_2$: 415.9320; found: 415.9330.

General procedure C: Electrochemical acylation/halogenation of various heteroaromatics

Scheme S5. Electrochemical cascade amidation/halogenation of various heteroaromatic compounds

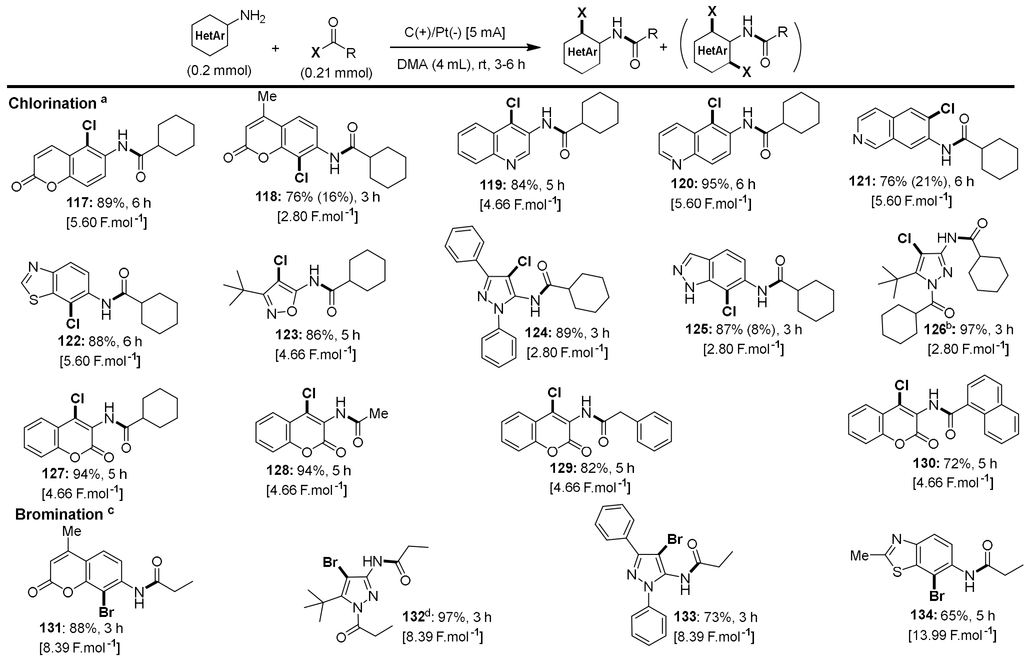

Reagents and conditions: [a] Amine (0.2 mmol) and acid chloride (0.21 mmol), in DMA (4 mL), in an undivided cell with carbon cloth (anode) and platinum (cathode) using 5 mA current. [b] Acid chloride (0.42 mmol); [c] Amine (0.2 mmol) and acid bromide (0.21 mmol), in DMA (4 mL), in an undivided cell with carbon cloth (anode) and platinum (cathode) using 15 mA current. [d] propionyl bromide (0.42 mmol); All yields refer to isolated yields; di-chlorinated product in parenthesis.

The electrolysis was carried out in 10 mL microwave reaction vial in an undivided cell setup under air. A carbon cloth anode (15 mm × 10 mm × 410 µm) and a platinum (Pt) cathode (25 mm × 10 mm × 0.125 mm) with electrode holders made of stainless steel were used and the distance between two electrodes was 10 mm. The cell was charged with the amine (0.20 mmol, 1.0 equiv) and a teflon-coated magnetic stirring bar (10 × 3 mm). 4 mL of DMA was added and the reaction mixture was stirred at 25 °C for 5 min to obtain a homogeneous solution, then acid halide (0.21 mmol, 1.05 equiv.) was added. Subsequently, the electrolysis was performed at room temperature with a constant current of 5.0 mA maintained for 3 h (unless otherwise stated) with a stirring rate of 600 rpm. After completion of the reaction, the reaction mixture was diluted with 2 mL ethyl acetate and transferred to a round bottom flask. The electrodes (carbon cloth and platinum) were washed in the reaction flask with ethyl acetate (3 × 5 mL) in an ultrasonic cleaner (3 × 3 min) and the washes were combined in the round bottom flask. The reaction mixture was then transferred to a separating funnel and washed with brine (15 mL). The aqueous layer was washed with ethyl acetate (3 x 15 mL) and the combined organic layer was

washed with brine (5 x 30 mL). The organic phase was dried with sodium sulfate and the solvent was removed under vacuum to obtain crude product. The crude products were purified by flash column chromatography using isohexane and ethyl acetate mixture (20:1 to 3:1) to afford the title compounds.

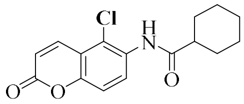

***N*-(5-chloro-2-oxo-2*H*-chromen-6-yl)cyclohexanecarboxamide (117)** was synthesized using 32.2 mg of 6-amino-2*H*-chromen-2-one, 28 μL of cyclohexanecarbonyl chloride and following general procedure C using 5 mA constant current for 6 h. Compound **117** was isolated using automated flash chromatography (silica gel, gradient elution 20-40% EtOAc in isohexane) as a white solid (54.0 mg, 89% yield). ^{1}H NMR (400 MHz, $CDCl_3$) δ 8.54 (d, J = 9.2 Hz, 1H), 8.06 (d, J = 9.8 Hz, 1H), 7.65 (s, 1H), 7.26 (d, J = 9.2 Hz, 1H), 6.51 (d, J = 9.8 Hz, 1H), 2.34 (tt, J = 11.7, 3.6 Hz, 1H), 2.06 – 1.99 (m, 2H), 1.86 (dt, J = 11.6, 3.0 Hz, 2H), 1.77 – 1.67 (m, 1H), 1.56 (qd, J = 12.1, 3.2 Hz, 2H), 1.33 (tdt, J = 24.8, 12.2, 3.2 Hz, 3H). ^{13}C NMR (101 MHz, $CDCl_3$) δ 174.5, 159.8, 150.8, 139.6, 131.9, 125.4, 120.2, 118.1, 117.1, 116.0, 46.6, 29.8, 25.7, 25.7. HRMS (ESI): m/z $[M+H]^+$ calcd for $C_{16}H_{17}NO_3Cl$: 306.0897; found: 306.0886.

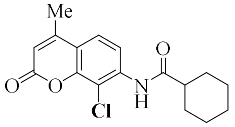

***N*-(8-chloro-4-methyl-2-oxo-2*H*-chromen-7-yl)cyclohexanecarboxamide (118)** was synthesized using 35.0 mg of 7-amino-4-methyl-2*H*-chromen-2-one, 28.0 μL of cyclohexanecarbonyl chloride and following general procedure C using 5 mA constant current for 6 h. Compound **118** was isolated using automated flash chromatography (silica gel, gradient elution 20-40% EtOAc in isohexane) as a white solid (48.5 mg, 76% yield). ^{1}H NMR (400 MHz, DMSO-d_6) δ 10.30 (s, 1H), 7.86 – 7.72 (m, 2H), 7.52 (dd, J = 8.8, 2.1 Hz, 1H), 2.52 (s, 3H), 2.36 (tt, J = 11.5, 3.5 Hz, 1H), 1.86 – 1.73 (m, 4H), 1.69 – 1.62 (m, 1H), 1.41 (qd, J = 12.2, 2.8 Hz, 2H), 1.33 – 1.17 (m, 3H). ^{13}C NMR (101 MHz, DMSO-d_6) δ 175.1, 156.4, 151.6, 148.6, 142.8, 126.4, 117.1, 115.6, 114.3, 105.2, 45.0, 29.0, 25.3, 25.1, 15.9. HRMS (ESI): m/z $[M+H]^+$ calcd for $C_{17}H_{19}NO_3Cl$: 320.1053; found: 320.1068.

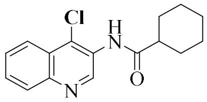

***N*-(4-chloroquinolin-3-yl)cyclohexanecarboxamide (119)** was synthesized using 28.8 mg of quinolin-3-amine, 28.0 μL of cyclohexanecarbonyl chloride and following general procedure C using 5 mA constant current for 5 h. Compound **119** was isolated using automated flash chromatography (silica gel, gradient elution 20-40% EtOAc in isohexane) as a white solid (48.0 mg, 84% yield). ^{1}H NMR (400 MHz, $CDCl_3$) δ 9.78 (s, 1H), 8.09 (ddd, J = 8.2, 2.8, 1.4 Hz, 2H), 7.73 (s, 1H), 7.68 (ddd, J

= 8.4, 6.9, 1.5 Hz, 1H), 7.61 (ddd, *J* = 8.2, 6.9, 1.3 Hz, 1H), 2.42 (tt, *J* = 11.7, 3.5 Hz, 1H), 2.10 – 2.03 (m, 2H), 1.88 (dt, *J* = 12.1, 3.0 Hz, 2H), 1.79 – 1.70 (m, 1H), 1.60 (qd, *J* = 12.1, 3.3 Hz, 2H), 1.43 – 1.26 (m, 3H). ^{13}C NMR (101 MHz, $CDCl_3$) δ 174.6, 145.6, 145.5, 129.9, 129.3, 128.9, 128.2, 128.1, 125.8, 123.6, 46.4, 29.8, 25.7, 25.7. HRMS (ESI): m/z $[M+H]^+$ calcd for $C_{16}H_{18}N_2OCl$: 289.1108; found: 289.1096.

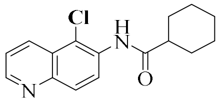

***N*-(5-chloroquinolin-6-yl)cyclohexanecarboxamide (120)** was synthesized using 28.8 mg of isoquinolin-6-amine, 28.0 μl of cyclohexanecarbonyl chloride and following general procedure C using 5 mA constant current for 6 h. Compound **120** was isolated using automated flash chromatography (silica gel, gradient elution 10-40% EtOAc in isohexane) as a white solid (54.5 mg, 95% yield). ^{1}H NMR (400 MHz, $CDCl_3$) δ 8.85 (dd, *J* = 4.2, 1.6 Hz, 1H), 8.76 (d, *J* = 9.3 Hz, 1H), 8.44 (dt, *J* = 8.5, 1.3 Hz, 1H), 8.02 (d, *J* = 9.4 Hz, 1H), 7.92 (s, 1H), 7.46 (dd, *J* = 8.6, 4.2 Hz, 1H), 2.38 (tt, *J* = 11.7, 3.5 Hz, 1H), 2.05 (ddt, *J* = 16.7, 5.0, 2.4 Hz, 2H), 1.87 (dt, *J* = 11.9, 3.0 Hz, 2H), 1.73 (dtd, *J* = 11.5, 3.4, 1.6 Hz, 1H), 1.59 (qd, *J* = 12.1, 3.2 Hz, 2H), 1.42 – 1.24 (m, 3H). ^{13}C NMR (101 MHz, $CDCl_3$) δ 174.6, 149.7, 145.7, 133.6, 132.2, 129.1, 126.2, 124.1, 122.2, 117.1, 46.8, 29.8, 25.8, 25.7. HRMS (ESI): m/z $[M+H]^+$ calcd for $C_{16}H_{18}N_2OCl$: 289.1108; found: 289.1102.

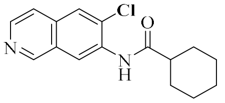

***N*-(6-chloroisoquinolin-7-yl)cyclohexanecarboxamide (121)** was synthesized using 28.8 mg of isoquinolin-7-amine, 28.0 μl of cyclohexanecarbonyl chloride and following general procedure C using 5 mA constant current for 6 h. Compound **121** was isolated using automated flash chromatography (silica gel, gradient elution 10-40% EtOAc in isohexane) as a white solid (43.8 mg, 76% yield). ^{1}H NMR (400 MHz, $CDCl_3$) δ 9.41 (d, *J* = 0.9 Hz, 1H), 8.86 (d, *J* = 9.3 Hz, 1H), 8.52 (s, 1H), 8.07 (d, *J* = 9.3 Hz, 1H), 7.90 (s, 1H), 7.19 (s, 1H), 2.34 (tt, *J* = 11.6, 3.5 Hz, 1H), 2.04 – 1.97 (m, 2H), 1.87 – 1.78 (m, 2H), 1.72 – 1.65 (m, 1H), 1.59 – 1.47 (m, 2H), 1.38-1.20 (m, 3H). ^{13}C NMR (101 MHz, $CDCl_3$) δ 174.8, 147.4, 141.7, 135.1, 131.3, 128.3, 126.6, 125.9, 123.2, 117.8, 46.8, 29.8, 25.8, 25.7. LC-MS: m/z $[M+H]^+$ calcd for $C_{16}H_{18}N_2OCl$: 289.1; found: 289.2.

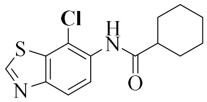

***N*-(7-chlorobenzo[*d*]thiazol-6-yl)cyclohexanecarboxamide (122)** was synthesized using 30.0 mg of benzo[*d*]thiazol-6-amine, 28.0 μL of cyclohexanecarbonyl chloride and following general procedure C using 5 mA constant current for 5 h. Compound **122** was isolated using automated flash chromatography

(silica gel, gradient elution 20-40% EtOAc in isohexane) as a white solid (51.5 mg, 88% yield). ^{1}H NMR (400 MHz, $CDCl_3$) δ 8.93 (s, 1H), 8.55 (d, *J* = 8.9 Hz, 1H), 8.02 (d, *J* = 9.0 Hz, 1H), 7.69 (s, 1H), 2.37 (tt, *J* = 11.7, 3.5 Hz, 1H), 2.07 – 2.01 (m, 2H), 1.87 (dt, *J* = 11.2, 2.9 Hz, 2H), 1.73 (dtd, *J* = 10.3, 3.2, 1.5 Hz, 1H), 1.58 (qd, *J* = 12.2, 3.3 Hz, 2H), 1.42 – 1.25 (m, 3H). ^{13}C NMR (101 MHz, $CDCl_3$) δ 174.5, 153.7, 149.7, 134.6, 132.9, 122.4, 121.0, 115.3, 46.8, 29.8, 25.8, 25.7. HRMS (ESI): m/z $[M+H]^+$ calcd for $C_{14}H_{16}N_2OSCl$: 295.0672; found: 295.0671.

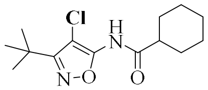

***N*-(4-chloro-2-oxo-2*H*-chromen-3-yl)acetamide (123)** was synthesized using 32.2 mg of 3-amino-2*H*-chromen-2-one, 15.0 μL of acetyl chloride and following general procedure C using 5 mA constant current for 6 h. Compound **123** was isolated using automated flash chromatography (silica gel, gradient elution 20-40% EtOAc in isohexane) as a white solid (44.5 mg, 94% yield). ^{1}H NMR (400 MHz, $CDCl_3$) δ 7.60 – 7.44 (m, 1H), 2.37 – 2.28 (m, 1H), 1.91 – 1.84 (m, 2H), 1.76 (dt, *J* = 9.7, 3.3 Hz, 2H), 1.65 – 1.59 (m, 1H), 1.52 – 1.38 (m, 2H), 1.33 (s, 9H), 1.27 – 1.18 (m, 3H). ^{13}C NMR (101 MHz, $CDCl_3$) δ 181.3, 168.0, 157.1, 42.9, 33.5, 29.4, 28.9, 27.6, 25.7, 25.5. HRMS (ESI): m/z $[M+H]^+$ calcd for $C_{14}H_{22}N_2O_2Cl$: 285.1370; found: 285.1369.

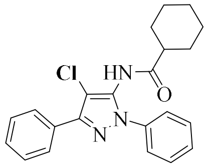

***N*-(4-chloro-1,3-diphenyl-1*H*-pyrazol-5-yl)cyclohexanecarboxamide (124)** was synthesized using 47.2 mg of 1,3-diphenyl-1*H*-pyrazol-5-amine, 28.0 μl of cyclohexanecarbonyl chloride and following general procedure C using 5 mA constant current for 3 h. Compound **124** was isolated using automated flash chromatography (silica gel, gradient elution 10-40% EtOAc in isohexane) as a white solid (67.5 mg, 89% yield). ^{1}H NMR (400 MHz, $CDCl_3$) δ 7.99 – 7.94 (m, 2H), 7.54 – 7.49 (m, 2H), 7.44 (t, *J* = 7.4 Hz, 4H), 7.41 – 7.36 (m, 2H), 6.87 (s, 1H), 2.27 (tt, *J* = 11.7, 3.5 Hz, 1H), 1.89 (d, *J* = 13.0 Hz, 2H), 1.79 (t, *J* = 6.6 Hz, 2H), 1.68 (s, 1H), 1.54 – 1.39 (m, 2H), 1.33 – 1.19 (m, 3H). ^{13}C NMR (101 MHz, $CDCl_3$) δ 175.5, 147.2, 138.7, 132.9, 131.7, 129.3, 128.6, 128.6, 128.5, 127.4, 124.2, 106.9, 45.3, 29.5, 25.7, 25.6. HRMS (ESI): m/z $[M+H]^+$ calcd for $C_{22}H_{23}N_3OCl$: 380.1530; found: 380.1532.

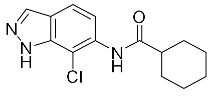

***N*-(7-chloro-1H-indazol-6-yl)cyclohexanecarboxamide (125)** was synthesized using 26.6 mg of 1*H*-indazol-6-amine, 28.0 μl of cyclohexanecarbonyl chloride and following general procedure C using 5 mA constant current for 3 h. Compound **125** was isolated using automated flash chromatography (silica gel, gradient elution 10-40% EtOAc in isohexane) as a white solid (48.0 mg, 87% yield). ^{1}H NMR (400

MHz, DMSO-d_6) δ 13.47 (s, 1H), 9.55 (s, 1H), 8.14 (s, 1H), 7.66 (d, *J* = 8.6 Hz, 1H), 7.28 (d, *J* = 8.5 Hz, 1H), 2.46 (dd, *J* = 11.5, 3.3 Hz, 1H), 1.90 – 1.82 (m, 2H), 1.80 – 1.73 (m, 2H), 1.66 (d, *J* = 11.6 Hz, 1H), 1.44 (qd, *J* = 12.3, 3.0 Hz, 2H), 1.35 – 1.19 (m, 3H). ^{13}C NMR (101 MHz, DMSO-d_6) δ 174.6, 138.2, 134.6, 133.0, 121.8, 120.4, 118.8, 109.0, 44.1, 29.2, 25.4, 25.2. HRMS (ESI): m/z $[M+H]^+$ calcd for $C_{14}H_{17}N_3OCl$: 278.1060; found: 278.1066.

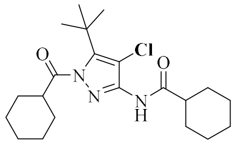

***N*-(5-(*tert*-butyl)-4-chloro-1-(cyclohexanecarbonyl)-1*H*-pyrazol-3-yl)cyclohexanecarboxamide (126)** was synthesized using 27.8 mg of 5-(*tert*-butyl)-1*H*-pyrazol-3-amine, 56.0 μl of cyclohexanecarbonyl chloride and following general procedure C using 5 mA constant current for 3 h. Compound **126** was isolated using automated flash chromatography (silica gel, gradient elution 10-40% EtOAc in isohexane) as a white solid (76.0 mg, 97% yield). ^{1}H NMR (400 MHz, $CDCl_3$) δ 8.65 (s, 1H), 3.55 (tt, *J* = 11.5, 3.4 Hz, 1H), 2.31 (tt, *J* = 11.6, 3.5 Hz, 1H), 2.03 – 1.91 (m, 4H), 1.86 – 1.77 (m, 4H), 1.70 (dddd, *J* = 15.3, 11.5, 5.0, 2.7 Hz, 2H), 1.54 – 1.41 (m, 4H), 1.38 (s, 9H), 1.36 – 1.18 (m, 6H). ^{13}C NMR (101 MHz, $CDCl_3$) δ 178.5, 172.6, 158.9, 136.7, 107.0, 45.6, 42.3, 33.7, 29.4, 29.1, 28.1, 25.9, 25.8, 25.7, 25.6. HRMS (ESI): m/z $[M+H]^+$ calcd for $C_{21}H_{33}N_3O_2Cl$: 394.2261; found: 394.2253.

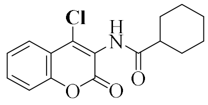

***N*-(4-chloro-2-oxo-2H-chromen-3-yl)cyclohexanecarboxamide (127)** was synthesized using 32.2 mg of 3-amino-2*H*-chromen-2-one, 28 μL of cyclohexanecarbonyl chloride and following general procedure C using 5 mA constant current for 5 h. Compound **127** was isolated using automated flash chromatography (silica gel, gradient elution 20-40% EtOAc in isohexane) as a white solid (58.0 mg, 94% yield). ^{1}H NMR (400 MHz, $CDCl_3$) δ 7.91 (dd, *J* = 8.0, 1.5 Hz, 1H), 7.57 (ddd, *J* = 8.6, 7.4, 1.6 Hz, 1H), 7.42 – 7.34 (m, 2H), 7.20 (s, 1H), 2.39 (tt, *J* = 11.6, 3.6 Hz, 1H), 2.06 – 1.99 (m, 2H), 1.85 (dt, *J* = 11.7, 3.3 Hz, 2H), 1.73 – 1.68 (m, 1H), 1.64 – 1.52 (m, 2H), 1.40 – 1.24 (m, 3H). ^{13}C NMR (101 MHz, $CDCl_3$) δ 173.8, 158.7, 150.5, 141.1, 132.2, 125.9, 125.3, 121.5, 118.9, 116.8, 45.8, 29.6, 25.8, 25.7. LC-MS: m/z $[M+H]^+$ calcd for $C_{16}H_{17}NO_3Cl$: 306.1; found: 306.1.

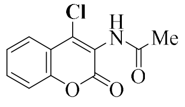

***N*-(4-chloro-2-oxo-2*H*-chromen-3-yl)acetamide (128)** was synthesized using 32.2 mg of 3-amino-2*H*-chromen-2-one, 15.0 μl of acetyl chloride and following general procedure C using 5 mA constant current for 5 h. Compound **128** was isolated using automated flash chromatography (silica gel, gradient elution 20-40% EtOAc in isohexane) as a white solid (44.5 mg, 94% yield). ^{1}H NMR (400 MHz, $CDCl_3$) δ 7.93 (dd, *J* = 8.0, 1.6 Hz, 1H), 7.60 (ddd, *J* = 8.2, 7.4, 1.6 Hz, 1H), 7.43 – 7.35 (m, 2H), 7.17 (s, 1H),

2.25 (s, 3H). ^{13}C NMR (101 MHz, $CDCl_3$) δ 168.0, 158.6, 150.7, 142.0, 132.5, 126.0, 125.4, 121.4, 118.7, 116.8, 23.6. HRMS (ESI): m/z $[M+H]^+$ calcd for $C_{11}H_9NO_3Cl$: 238.0271; found: 238.0269.

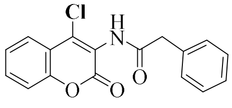

***N*-(4-chloro-2-oxo-2H-chromen-3-yl)-2-phenylacetamide (129)** was synthesized using 32.2 mg of 3-amino-2*H*-chromen-2-one, 31.5 μL of phenylacetyl chloride and following general procedure C using 5 mA constant current for 5 h. Compound **129** was isolated using automated flash chromatography (silica gel, gradient elution 20-40% EtOAc in isohexane) as a white solid (53.0 mg, 82% yield). ^{1}H NMR (400 MHz, $CDCl_3$) δ 7.89 (dd, *J* = 8.0, 1.6 Hz, 1H), 7.57 (ddd, *J* = 8.3, 7.4, 1.6 Hz, 1H), 7.44 – 7.31 (m, 7H), 7.11 (s, 1H), 3.82 (s, 2H). ^{13}C NMR (101 MHz, $CDCl_3$) δ 168.9, 158.2, 150.7, 142.0, 134.0, 132.5, 129.7, 129.4, 127.9, 125.9, 125.3, 121.4, 118.6, 116.8, 44.0. HRMS (ESI): m/z $[M+H]^+$ calcd for $C_{17}H_{13}NO_3Cl$: 314.0584; found: 314.0570.

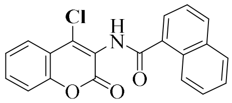

***N*-(4-chloro-2-oxo-2*H*-chromen-3-yl)-1-naphthamide (130)** was synthesized using 32.2 mg of 3-amino-2*H*-chromen-2-one, 31.5 μL of 1-naphthoyl chloride and following general procedure C using 5 mA constant current for 5 h. Compound **130** was isolated using automated flash chromatography (silica gel, gradient elution 10-40% EtOAc in isohexane) as a white solid (50.0 mg, 72% yield). ^{1}H NMR (400 MHz, DMSO-d_6) δ 10.58 (s, 1H), 8.42 – 8.36 (m, 1H), 8.14 – 8.09 (m, 1H), 8.06 – 8.01 (m, 1H), 7.98 (dd, *J* = 8.0, 1.5 Hz, 1H), 7.84 – 7.75 (m, 2H), 7.67 – 7.52 (m, 5H). ^{13}C NMR (101 MHz, DMSO-d_6) δ 167.7, 156.9, 150.7, 143.6, 133.2, 133.2, 133.1, 130.7, 129.8, 128.3, 127.1, 126.5, 126.0, 125.6, 125.5, 125.2, 124.9, 122.6, 117.6, 116.7. HRMS (ESI): m/z $[M+H]^+$ calcd for $C_{20}H_{13}NO_3Cl$: 350.0584; found: 350.0573.

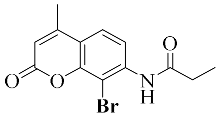

***N*-(8-bromo-4-methyl-2-oxo-2*H*-chromen-7-yl)propionamide (131)** was synthesized using 35.0 mg of 7-amino-4-methyl-2*H*-chromen-2-one, 19.0 μL of propionyl bromide and following general procedure C using 15 mA constant current for 3 h. Compound **131** was isolated using automated flash chromatography (silica gel, gradient elution 30-70% EtOAc in isohexane) as a pale yellow solid (54.4 mg, 88% yield). ^{1}H NMR (400 MHz, DMSO-d_6) δ 10.36 (s, 1H), 7.94 – 7.71 (m, 2H), 7.49 (dd, *J* = 8.8, 2.2 Hz, 1H), 2.57 (s, 3H), 2.38 (q, *J* = 7.5 Hz, 2H), 1.09 (t, *J* = 7.5 Hz, 3H). ^{13}C NMR (101 MHz, DMSO-d_6) δ 172.8, 156.4, 152.1, 151.6, 142.9, 126.6, 115.5, 114.4, 109.5, 105.1, 29.6, 19.2, 9.3. HRMS (ESI): m/z $[M+H]^+$ calcd for $C_{13}H_{13}NO_3Br$: 310.0079; found: 310.0084.

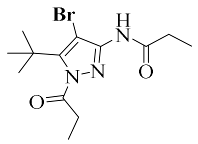

***N*-(4-bromo-5-(*tert*-butyl)-1-propionyl-1*H*-pyrazol-3-yl)propionamide (132)** was synthesized using 27.8 mg of 5-(*tert*-butyl)-1H-pyrazol-3-amine, 38.0 μL of propionyl bromide and following general procedure C using 15 mA constant current for 3 h. Compound **132** was isolated using automated flash chromatography (silica gel, gradient elution 20-40% EtOAc in isohexane) as a white solid (63.5 mg, 97% yield). ^{1}H NMR (400 MHz, $CDCl_3$) δ 8.27 (s, 1H), 3.08 (q, *J* = 7.4 Hz, 2H), 2.44 (q, *J* = 7.5 Hz, 2H), 1.40 (s, 9H), 1.23 (dt, *J* = 11.0, 7.5 Hz, 6H). ^{13}C NMR (101 MHz, $CDCl_3$) δ 175.8, 170.7, 159.4, 138.1, 94.0, 34.0, 30.1, 30.0, 28.3, 9.3, 8.3. HRMS (ESI): m/z $[M+H]^+$ calcd for $C_{13}H_{21}N_3O_2Br$: 330.0817; found: 330.0825.

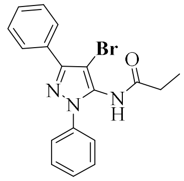

***N*-(4-bromo-1,3-diphenyl-1*H*-pyrazol-5-yl)propionamide (133)** was synthesized using 47.2 mg of 1,3-diphenyl-1*H*-pyrazol-5-amine, 19.0 μL of propionyl bromide and following general procedure C using 15 mA constant current for 3 h. Compound **133** was isolated using automated flash chromatography (silica gel, gradient elution 20-40% EtOAc in isohexane) as a white solid (57.8 mg, 73% yield). ^{1}H NMR (400 MHz, $CDCl_3$) δ 7.93 (d, *J* = 7.5 Hz, 2H), 7.42 (ddd, *J* = 19.7, 14.1, 7.2 Hz, 8H), 7.25 (s, 1H), 2.25 (q, *J* = 7.6 Hz, 2H), 1.11 (t, *J* = 7.6 Hz, 3H). ^{13}C NMR (101 MHz, $CDCl_3$) δ 173.6, 148.8, 138.6, 134.7, 131.9, 129.3, 128.6, 128.5, 128.4, 127.7, 124.1, 93.2, 29.5, 9.6. HRMS (ESI): m/z $[M+H]^+$ calcd for $C_{18}H_{17}N_3OBr$: 370.0555; found: 370.0552.

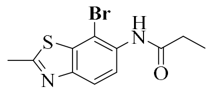

***N*-(7-bromo-2-methylbenzo[*d*]thiazol-6-yl)propionamide (134)** was synthesized using 32.8 mg of 2-methylbenzo[*d*]thiazol-6-amine, 19.0 μL of propionyl bromide and following general procedure C using 15 mA constant current for 5 h. Compound **134** was isolated using automated flash chromatography (silica gel, gradient elution 20-40% EtOAc in isohexane) as an off-white solid (38.7 mg, 65% yield). ^{1}H NMR (400 MHz, $CDCl_3$) δ 8.42 (d, *J* = 8.6 Hz, 1H), 7.80 (s, 1H), 7.72 (d, *J* = 8.8 Hz, 1H), 2.87 (s, 3H), 2.52 (q, *J* = 7.5 Hz, 2H), 1.31 (t, *J* = 7.6 Hz, 3H). ^{13}C NMR (101 MHz, $CDCl_3$) δ 172.2, 169.4, 151.8, 134.5, 131.1, 120.6, 119.1, 106.5, 31.2, 20.5, 9.7. HRMS (ESI): m/z $[M+H]^+$ calcd for $C_{11}H_{12}N_2OSBr$: 298.9854; found: 298.9843.

Cascades involving pharmaceutically-relevant molecules [a]

Scheme S6. Cascades involving pharmaceutically-relevant molecules

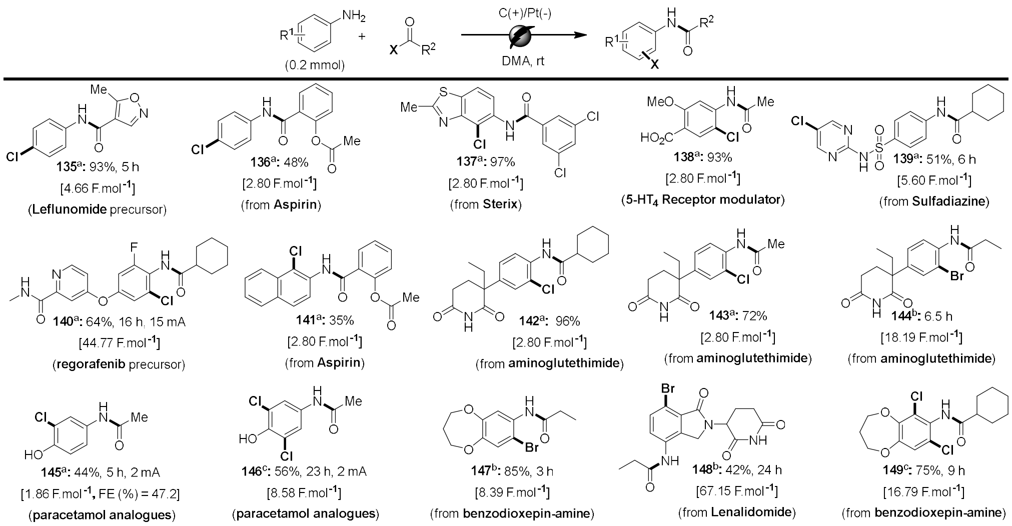

Reagents and conditions: [a] Amine (0.2 mmol) and acid chloride (0.21 mmol) in DMA (4 mL), 5 mA, 3 h; [b] Amine (0.2 mmol) and acid bromide (0.21 mmol) in DMA (4 mL), 15 mA; [c] Amine (0.2 mmol) and acid chloride (0.42 mmol) in DMA (4 mL), 10 mA; All yields refer to isolated yields. All reactions were carried out in an undivided cell with carbon cloth (anode) and platinum (cathode) unless otherwise mentioned.

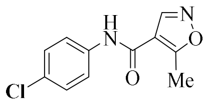

***N*-(4-chlorophenyl)-5-methylisoxazole-4-carboxamide (135)** was synthesized using 18.6 mg of aniline, 30.5 mg of 5-methylisoxazole-4-carbonyl chloride and following general procedure A using 5 mA constant current for 3 h. Compound **135** was isolated using automated flash chromatography (silica gel, gradient elution 5-30% EtOAc in isohexane) as a white solid (44 mg, 93% yield). ^{1}H NMR (400 MHz, $CDCl_3$) δ 8.36 (s, 1H), 7.59 (s, 1H), 7.40 – 7.36 (m, 2H), 7.22 – 7.18 (m, 2H), 2.64 (d, *J* = 0.7 Hz, 3H). ^{13}C NMR (101 MHz, $CDCl_3$) δ 173.9, 159.5, 147.7, 135.7, 130.3, 129.3, 122.0, 112.0, 12.8. HRMS (ESI): m/z $[M+H]^+$ calcd for $C_{11}H_{10}N_2O_2Cl$: 237.0431; found: 237.0437.

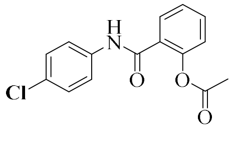

2-((4-chlorophenyl)carbamoyl)phenyl acetate (136) was synthesized using 18.6 mg of aniline, 15.7 µL of *O*-acetylsalicyloyl chloride and following general procedure A using 5 mA constant current for 3 h. Compound **136** was isolated using automated flash chromatography (silica gel, gradient elution 5-30% EtOAc in isohexane) as an off-white solid (27.5 mg, 48% yield). ^{1}H NMR (400 MHz, $CDCl_3$) δ 8.07 (s, 1H), 7.81 (dd, *J* = 7.8, 1.7 Hz, 1H), 7.57 – 7.49 (m, 3H), 7.38 – 7.29 (m, 3H), 7.15 (dd, *J* = 8.1,

1.2 Hz, 1H), 2.32 (s, 3H). ^{13}C NMR (101 MHz, $CDCl_3$) δ 169.4, 163.8, 147.9, 136.5, 132.5, 130.0, 129.8, 129.3, 128.6, 126.7, 123.5, 121.2, 21.2.

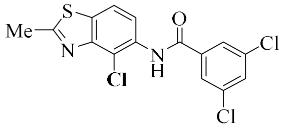

3,5-dichloro-*N*-(4-chloro-2-methylbenzo[*d*]thiazol-5-yl)benzamide (137) was synthesized using 32.8 mg of 2-methylbenzo[*d*]thiazol-5-amine, 44.0 mg of 3,5-dichlorobenzoyl chloride and following general procedure A using 5 mA constant current for 3 h. Compound **137** was isolated using automated flash chromatography (silica gel, gradient elution 5-30% EtOAc in isohexane) as a white solid (71.5 mg, 97% yield). ^{1}H NMR (400 MHz, $CDCl_3$) δ 8.50 (d, *J* = 8.8 Hz, 1H), 8.41 (s, 1H), 7.81 (d, *J* = 1.9 Hz, 2H), 7.77 (d, *J* = 8.8 Hz, 1H), 7.58 (t, *J* = 1.9 Hz, 1H), 2.90 (s, 3H). ^{13}C NMR (101 MHz, $CDCl_3$) δ 170.1, 163.0, 150.5, 137.5, 136.1, 132.6, 132.5, 132.3, 125.9, 120.2, 118.9, 116.3, 20.6.

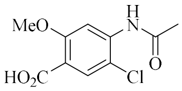

4-acetamido-5-chloro-2-methoxybenzoic acid (138) was synthesized using 33.4 mg of 4-amino-2-methoxybenzoic acid, 15.0 μL of acetyl chloride and following general procedure A using 5 mA constant current for 3 h. Compound **138** was isolated following work-up and recrystallisation from ethyl acetate as a white crystalline solid (45.2 mg, 93% yield). ^{1}H NMR (400 MHz, DMSO-d_6) δ 12.74 (s, 1H), 9.55 (s, 1H), 7.76 (s, 1H), 7.71 (s, 1H), 3.77 (s, 3H), 2.15 (s, 3H). ^{13}C NMR (101 MHz, DMSO-d_6) δ 169.2, 165.4, 157.6, 139.2, 131.3, 117.4, 115.1, 108.2, 56.0, 23.8.

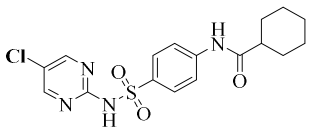

***N*-(4-(N-(5-chloropyrimidin-2-yl)sulfamoyl)phenyl)cyclohexanecarboxamide (139)** was synthesized using 50.0 mg of 4-amino-*N*-(pyrimidin-2-yl)benzenesulfonamide, 28.0 μL of cyclohexanecarbonyl chloride and following general procedure A using 5 mA constant current for 6 h. Compound **139** was isolated following work-up and recrystallisation from ethyl acetate as a white crystalline solid (40.0 mg, 51% yield). ^{1}H NMR (400 MHz, DMSO-d_6) δ 11.89 (s, 1H), 10.23 (d, *J* = 5.8 Hz, 1H), 8.61 (s, 2H), 7.99 – 7.82 (m, 2H), 7.82 – 7.72 (m, 2H), 2.39 – 2.29 (m, 1H), 1.82 – 1.70 (m, 4H), 1.64 (d, *J* = 10.9 Hz, 1H), 1.43 – 1.33 (m, 2H), 1.31 – 1.14 (m, 3H). ^{13}C NMR (101 MHz, DMSO-d_6) δ 175.0, 156.7, 155.3, 143.6, 133.1, 128.9, 124.1, 118.3, 44.9, 29.0, 25.3, 25.1. HRMS (ESI): m/z $[M+H]^+$ calcd for $C_{17}H_{20}N_4O_3SCl$: 395.0945; found: 395.0956.

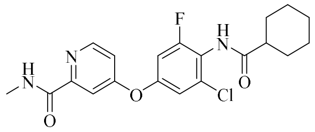

4-(3-chloro-4-(cyclohexanecarboxamido)-5-fluorophenoxy)-N-methylpicolinamide (140) was synthesized using 52.0 mg of 4-(4-amino-3-fluorophenoxy)-N-methylpicolinamide, 28.0 μL of cyclohexanecarbonyl chloride and following general procedure A using 15 mA constant current for 16 h. Compound **140** was isolated following work-up and recrystallisation from ethyl acetate as a white crystalline solid (51.5 mg, 64% yield). ^{1}H NMR (400 MHz, $CDCl_3$) δ 8.44 (d, *J* = 5.5 Hz, 1H), 8.00 (d, *J* = 6.6 Hz, 1H), 7.76 (d, *J* = 2.5 Hz, 1H), 7.04 – 6.97 (m, 2H), 6.88 – 6.79 (m, 2H), 3.02 (d, *J* = 5.1 Hz, 3H), 2.46 – 2.32 (m, 1H), 2.04 (d, *J* = 12.9 Hz, 2H), 1.92 – 1.80 (m, 2H), 1.74 – 1.69 (m, 1H), 1.65 – 1.51 (m, 2H), 1.44 – 1.25 (m, 3H). ^{13}C NMR (101 MHz, $CDCl_3$) δ 164.9, 164.2, 160.1, 157.6, 152.7, 150.1, 120.8, 120.6, 117.2 (d, *J* = 3.6 Hz), 114.7, 111.0, 108.2, 107.9, 45.3, 29.6, 26.2, 25.7, 25.6. ^{19}F NMR (376 MHz, $CDCl_3$) δ -110.53 (d, *J* = 9.6 Hz). HRMS (ESI): m/z $[M+H]^+$ calcd for $C_{20}H_{22}N_3O_3FCl$: 406.1334; found: 406.1343.

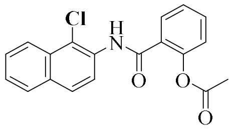

2-((1-Chloronaphthalen-2-yl)carbamoyl)phenyl acetate (141) was synthesized using 28.6 mg of naphthalen-2-amine, 15.7 μL of *O*-acetylsalicyloyl chloride and following general procedure A using 5 mA constant current for 3 h. Compound **141** was isolated using automated flash chromatography (silica gel, gradient elution 5-30% EtOAc in isohexane) as a pale brown solid (23.5 mg, 35% yield). ^{1}H NMR (400 MHz, $CDCl_3$) δ 8.96 (s, 1H), 8.70 (d, *J* = 9.1 Hz, 1H), 8.20 (dt, *J* = 8.5, 1.0 Hz, 1H), 8.03 (dd, *J* = 7.8, 1.7 Hz, 1H), 7.87 – 7.82 (m, 2H), 7.63 – 7.55 (m, 2H), 7.50 (ddd, *J* = 8.1, 6.9, 1.2 Hz, 1H), 7.42 (td, *J* = 7.6, 1.2 Hz, 1H), 7.22 (dd, *J* = 8.1, 1.1 Hz, 1H), 2.39 (s, 3H). ^{13}C NMR (101 MHz, $CDCl_3$) δ 169.1, 163.7, 148.3, 133.1, 132.8, 131.5, 130.8, 130.7, 128.3, 128.0, 127.9, 127.7, 126.7, 125.8, 124.0, 123.7, 120.7, 118.4, 21.5. HRMS (ESI): m/z $[M+H]^+$ calcd for $C_{19}H_{15}NO_3Cl$: 340.0740; found: 340.0740.

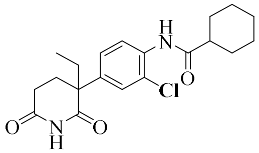

***N*-(2-chloro-4-(3-ethyl-2,6-dioxopiperidin-3-yl)phenyl)cyclohexanecarboxamide (142)** was synthesized using 46.4 mg of 3-(4-aminophenyl)-3-ethylpiperidine-2,6-dione, 28.0 μL of cyclohexanecarbonyl chloride and following general procedure A using 5 mA constant current for 3 h. Compound **142** was isolated using automated flash chromatography (silica gel, gradient elution 5-30%

EtOAc in isohexane) as a white solid (65.5 mg, 96% yield). ^{1}H NMR (400 MHz, $CDCl_3$) δ 8.43 (d, J = 8.7 Hz, 1H), 7.92 (d, J = 4.4 Hz, 1H), 7.69 (s, 1H), 7.29 (d, J = 2.3 Hz, 1H), 7.18 (dd, J = 8.8, 2.3 Hz, 1H), 2.66 – 2.57 (m, 1H), 2.46 – 2.20 (m, 4H), 2.06 – 1.97 (m, 3H), 1.92 – 1.82 (m, 3H), 1.74 – 1.68 (m, 1H), 1.60 – 1.47 (m, 2H), 1.40 – 1.23 (m, 3H), 0.86 (t, J = 7.4 Hz, 3H). ^{13}C NMR (101 MHz, $CDCl_3$) δ 174.7, 174.5, 172.2, 135.1, 134.3, 127.1, 125.5, 123.3, 121.8, 50.6, 46.7, 33.0, 29.7, 29.3, 26.9, 25.7, 25.7, 9.0. HRMS (ESI): m/z $[M+H]^+$ calcd for $C_{20}H_{26}N_2O_3Cl$: 377.1632; found: 377.1613.

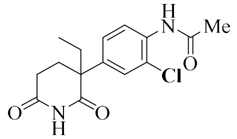

***N*-(2-chloro-4-(3-ethyl-2,6-dioxopiperidin-3-yl)phenyl)acetamide (143)** was synthesized using 46.4 mg of 3-(4-aminophenyl)-3-ethylpiperidine-2,6-dione, 15.0 µL of acetyl chloride and following general procedure A using 5 mA constant current for 3 h. Compound **143** was isolated using automated flash chromatography (silica gel, gradient elution 5-30% EtOAc in isohexane) as a colorless sticky gum (39.5 mg, 72% yield). ^{1}H NMR (400 MHz, $CDCl_3$) δ 8.39 (d, J = 8.7 Hz, 1H), 7.88 (s, 1H), 7.59 (s, 1H), 7.29 (d, J = 2.3 Hz, 1H), 7.19 (dd, J = 8.7, 2.3 Hz, 1H), 2.67 – 2.58 (m, 1H), 2.46 – 2.30 (m, 2H), 2.28 – 2.20 (m, 4H), 2.03 (dq, J = 14.8, 7.4 Hz, 1H), 1.90 (dq, J = 14.6, 7.4 Hz, 1H), 0.87 (t, J = 7.4 Hz, 3H). ^{13}C NMR (101 MHz, $CDCl_3$) δ 174.7, 172.1, 168.5, 135.4, 134.2, 127.1, 125.6, 123.2, 121.9, 50.6, 33.0, 29.3, 26.9, 24.9, 9.1.

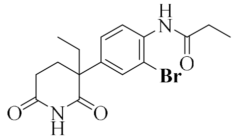

***N*-(2-bromo-4-(3-ethyl-2,6-dioxopiperidin-3-yl)phenyl)propionamide (144)** was synthesized using 46.4 mg of 3-(4-aminophenyl)-3-ethylpiperidine-2,6-dione, 19.0 µL of propionyl bromide and following general procedure A using 15 mA constant current for 6.5 h. Compound **144** was isolated using automated flash chromatography (silica gel, gradient elution 5-30% EtOAc in isohexane) as a white solid (54.8 mg, 75% yield). ^{1}H NMR (400 MHz, DMSO-d_6) δ 10.92 (s, 1H), 9.35 (s, 1H), 7.61 (d, J = 8.5 Hz, 1H), 7.51 (d, J = 2.2 Hz, 1H), 7.29 (dd, J = 8.5, 2.2 Hz, 1H), 2.48 (d, J = 7.1 Hz, 1H), 2.41 – 2.32 (m, 3H), 2.18 – 2.11 (m, 2H), 1.84 (dh, J = 21.1, 7.1 Hz, 2H), 1.08 (t, J = 7.5 Hz, 3H), 0.76 (t, J = 7.3 Hz, 3H). ^{13}C NMR (101 MHz, DMSO-d_6) δ 175.3, 172.6, 172.2, 138.5, 135.4, 130.3, 127.0, 126.0, 118.0, 49.8, 31.9, 29.0, 28.9, 25.9, 9.7, 8.9. HRMS (ESI): m/z $[M+H]^+$ calcd for $C_{17}H_{20}N_2O_3Br$: 379.0657; found: 379.0641.

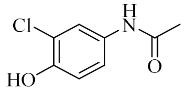

***N*-(3-chloro-4-hydroxyphenyl)acetamide (145)** was synthesized using 21.8 mg of 4-aminophenol, 15.0 µL of acetyl chloride and following general procedure A using 2 mA constant current for 5 h.

Compound **145** was isolated using automated flash chromatography (silica gel, gradient elution 100% EtOAc) as a pale white solid (16.3 mg, 44% yield). ^{1}H NMR (400 MHz, DMSO-d$_6$) δ 9.84 (s, 1H), 9.81 (s, 1H), 7.68 (d, *J* = 2.6 Hz, 1H), 7.22 (dd, *J* = 8.8, 2.5 Hz, 1H), 6.88 (d, *J* = 8.7 Hz, 1H), 1.99 (s, 3H).^{13}C NMR (101 MHz, DMSO-d$_6$) δ 167.8, 148.7, 131.8, 120.6, 119.1, 119.0, 116.4, 23.8.

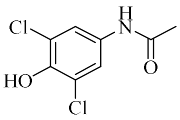

***N*-(3,5-dichloro-4-hydroxyphenyl)acetamide(146)** was synthesized using 21.8 mg of 4-aminophenol, 30.0 μL of acetyl chloride and following general procedure B using 2 mA constant current for 23 h. Compound **146** was isolated using automated flash chromatography (silica gel, gradient elution 100% EtOAc) as a pale white solid (24.5 mg, 56% yield). ^{1}H NMR (400 MHz, DMSO-d$_6$) δ 9.96 (s, 1H), 9.81 (s, 1H), 7.58 (s, 2H), 2.01 (s, 3H). ^{13}C NMR (101 MHz, DMSO-d$_6$) δ 168.3, 144.6, 132.5, 122.2, 118.9, 23.9. HRMS (ESI): m/z [M-H]$^-$ calcd for $C_8H_6l_2NO_2$: 217.9781; found: 217.9777.

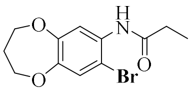

***N*-(8-bromo-3,4-dihydro-2*H*-benzo[*b*][1,4]dioxepin-7-yl)propionamide (147)** was synthesized using 33.0 mg of 3,4-dihydro-2*H*-benzo[*b*][1,4]dioxepin-7-amine, 19.0 μL of propionyl bromide and following general procedure A using 15 mA constant current for 3 h. Compound **147** was isolated using automated flash chromatography (silica gel, gradient elution 5-20% EtOAc in isohexane) as a white solid (50.8 mg, 85% yield). ^{1}H NMR (400 MHz, CDCl$_3$) δ 8.02 (s, 1H), 7.51 – 7.35 (m, 1H), 7.16 (s, 1H), 4.18 (dt, *J* = 11.1, 5.6 Hz, 4H), 2.43 (q, *J* = 7.6 Hz, 2H), 2.17 (p, *J* = 5.6 Hz, 2H), 1.26 (t, *J* = 7.6 Hz, 3H). ^{13}C NMR (101 MHz, CDCl$_3$) δ 171.7, 151.1, 148.1, 131.1, 124.5, 115.2, 105.8, 71.1, 70.8, 31.9, 31.1, 9.8. HRMS (ESI): m/z [M+H]$^+$ calcd for $C_{12}H_{15}NO_3Br$: 300.0235; found: 300.0242.

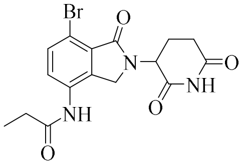

***N*-(7-bromo-2-(2,6-dioxopiperidin-3-yl)-1-oxoisoindolin-4-yl)propionamide (148)** was synthesized using 51.8 mg of 3-(4-amino-1-oxoisoindolin-2-yl)piperidine-2,6-dione, 19.0 μL of propionyl bromide and following general procedure A using 15 mA constant current for 24 h. Compound **148** was isolated using automated flash chromatography (silica gel, gradient elution 5-10% MeOH in DCM) as a pale yellow solid (33.0 mg, 42% yield). ^{1}H NMR (400 MHz, DMSO-d$_6$) δ 11.02 (s, 1H), 9.76 (s, 1H), 7.83 – 7.80 (m, 1H), 7.51 – 7.48 (m, 1H), 5.19 – 5.12 (m, 1H), 4.36 (t, *J* = 9.8 Hz, 2H), 2.92 (ddd, *J* = 17.3, 13.6, 5.4 Hz, 1H), 2.66 – 2.57 (m, 1H), 2.38 (q, *J* = 7.6 Hz, 3H), 2.08 – 1.99 (m, 1H), 1.13 – 1.08 (m, 3H). ^{13}C NMR (101 MHz, DMSO-d$_6$) δ 172.9, 172.1, 171.1, 167.8, 133.8, 133.4, 132.7, 128.6, 125.2,

118.9, 51.5, 46.4, 31.2, 29.0, 22.6, 9.7. HRMS (ESI): m/z $[M+H]^+$ calcd for $C_{16}H_{17}N_3O_4Br$: 394.0402; found: 394.0412.

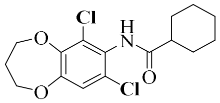

***N*-(6,8-dichloro-3,4-dihydro-2*H*-benzo[*b*][1,4]dioxepin-7-yl)cyclohexanecarboxamide (149)** was synthesized using 33.0 mg of 3,4-dihydro-2*H*-benzo[*b*][1,4]dioxepin-7-amine, 56.0 µL of cyclohexanecarbonyl chloride and following general procedure B using 10 mA constant current for 9 h. Compound **149** was isolated using automated flash chromatography (silica gel, gradient elution 5-20% EtOAc in isohexane) as a pale white solid (51.4 mg, 75% yield). ^{1}H NMR (400 MHz, $CDCl_3$) δ 7.01 (s, 1H), 6.80 (s, 1H), 4.30 (t, *J* = 5.7 Hz, 2H), 4.24 (t, *J* = 5.7 Hz, 2H), 2.36 (ddt, *J* = 11.7, 9.0, 3.5 Hz, 1H), 2.24 (p, *J* = 5.7 Hz, 2H), 2.07 – 2.02 (m, 2H), 1.89 – 1.82 (m, 2H), 1.71 (d, *J* = 10.6 Hz, 1H), 1.63 – 1.53 (m, 2H), 1.39 – 1.25 (m, 3H). ^{13}C NMR (101 MHz, $CDCl_3$) δ 174.4, 151.2, 147.2, 127.3, 126.7, 126.3, 120.4, 71.1, 70.9, 45.7, 31.3, 29.8, 25.9, 25.8. HRMS (ESI): m/z $[M+H]^+$ calcd for $C_{16}H_{20}NO_3Cl_2$: 344.0820; found: 344.0810.

General procedure D: Scale-up

Scheme S7. Scale up examples of electrochemical cascade amidation/halogenation [a]

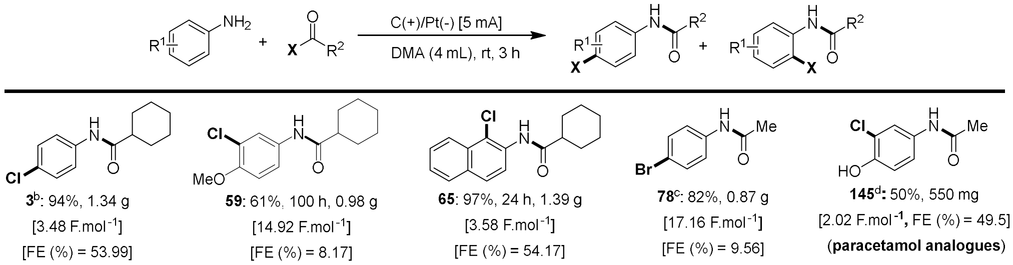

Reagents and conditions: [a] amine (5 mmol), cyclohexanecarbonyl chloride (5.25 mmol), and DMA (30 mL), 20 mA current in an undivided cell with carbon cloth (anode and cathode); [b] Aniline (6 mmol), cyclohexanecarbonyl chloride (6.3 mmol), 28 h. [c] Acetyl bromide (5.25 mmol), 100 mA current, 23 h. [d] Acetyl chloride (5.25 mmol), 2 mA, 65 h; All yields refer to isolated yields.

The electrolysis was carried out in 50 mL round bottom flask in an undivided cell setup under air. A carbon cloth anode (40 mm × 30 mm × 410 µm) and a carbon cloth cathode (40 mm × 30 mm × 410 µm) with electrode holders made of stainless steel were used and the distance between two electrodes was 20 mm. The cell was charged with the amine (1.0 equiv.) and a teflon-coated magnetic stirring bar (15 × 6 mm). 30 mL of DMA was added and the reaction mixture was stirred at 25 °C for 5 min to obtain a homogeneous solution, then acid chloride (1.05 equiv.) was added. Subsequently, the electrolysis was performed at room temperature with a constant current of 20.0 mA with a stirring rate of 600 rpm. After completion of the reaction, the reaction mixture was diluted with 20 mL ethyl acetate

and transferred to a round bottom flask. The electrodes (carbon cloth and platinum) were washed in the reaction flask with ethyl acetate (3 × 10 mL) in an ultrasonic cleaner (3 × 3 min) and the washes were combined in the round bottom flask. The reaction mixture was then transferred to a separating funnel and washed with brine (50 mL). The aqueous layer was washed with ethyl acetate (3 x 50 mL) and the combined organic layer was washed with brine (5 x 100 mL). The organic phase was dried with sodium sulfate and the solvent was removed under vacuum to obtain crude product. The crude products were purified by flash column chromatography using isohexane and ethyl acetate mixture (20:1 to 3:1) to afford the title compounds.

Experimental Setup (Gram scale):

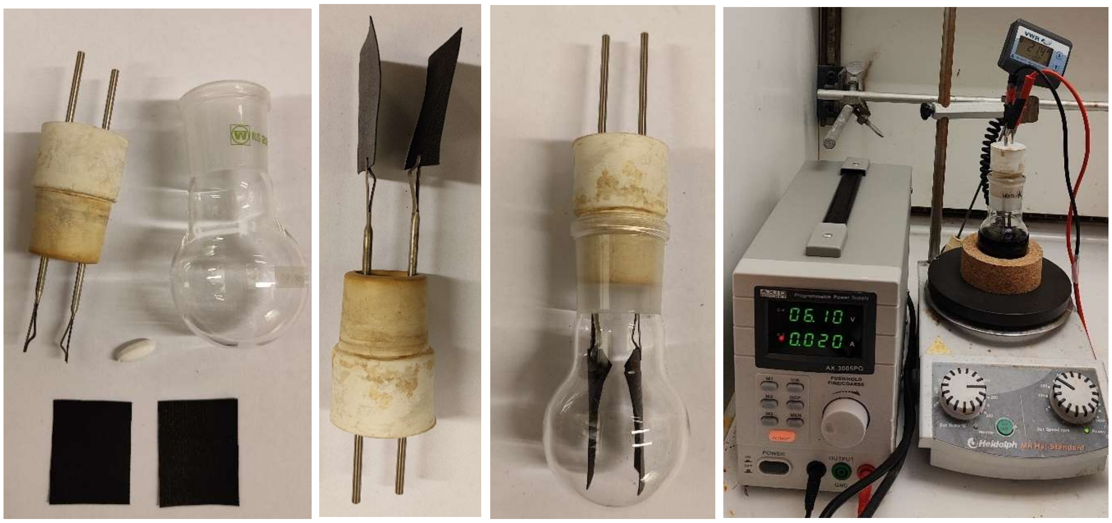

Figure S2. Images of the equipment and instrumentation used in this scale up experiment, including reaction flask, electrodes, electrode holders, and power supplies.

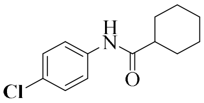

***N*-(4-chlorophenyl)cyclohexanecarboxamide (3)** was synthesized using aniline (556 mg, 6 mmol, 1.0 equiv.), cyclohexanecarbonyl chloride (840 µL, 6.3 mmol, 1.05 equiv) and following general procedure E-A using 20.0 mA constant current for 28 h. Compound **3** was obtained as a white solid (1.34 g, 94% yield) following work-up without the need for any further purification. ^{1}H NMR (400 MHz, DMSO-d_6) δ 9.92 (s, 1H), 7.70 – 7.50 (m, 2H), 7.37 – 7.23 (m, 2H), 2.30 (tt, *J* = 11.8, 3.3 Hz, 1H), 1.77 (tt, *J* = 15.0, 2.2 Hz, 4H), 1.67 – 1.61 (m, 1H), 1.39 (qd, *J* = 13.5, 13.1, 3.5 Hz, 2H), 1.32 – 1.14 (m, 3H). ^{13}C NMR (101 MHz, DMSO-d_6) δ 174.4, 138.4, 128.5, 126.3, 120.5, 44.8, 29.1, 25.4, 25.2. HRMS (ESI): m/z $[M+H]^+$ calcd for $C_{13}H_{17}NOCl$: 238.0999; found: 238.0988.

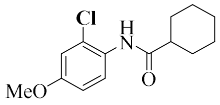

***N*-(2-chloro-4-methoxyphenyl)cyclohexanecarboxamide (59)** was synthesized using 4-methoxyaniline (738 mg, 6 mmol, 1.0 equiv.), cyclohexanecarbonyl chloride (840 µL, 6.3 mmol, 1.05 equiv.) and following general procedure E-A using 20.0 mA constant current for 100 h. Compound **59** was purified using flash chromatography (silica gel, gradient elution 5-10% EtOAc in isohexane) as a white solid (970 mg, 61% yield). ^{1}H NMR (400 MHz, $CDCl_3$) δ 8.17 (d, *J* = 9.1 Hz, 1H), 7.50 (s, 1H), 6.90 (d, *J* = 2.9 Hz, 1H), 6.79 (dd, *J* = 9.1, 2.9 Hz, 1H), 3.75 (s, 3H), 2.28 (tt, *J* = 11.7, 3.5 Hz, 1H), 2.02 – 1.95 (m, 2H), 1.83 (dt, *J* = 13.0, 3.5 Hz, 2H), 1.70 (dtd, *J* = 10.5, 3.6, 1.7 Hz, 1H), 1.52 (qd, *J* = 12.1, 3.3 Hz, 2H), 1.38 – 1.21 (m, 3H). ^{13}C NMR (101 MHz, $CDCl_3$) δ 174.2, 156.2, 128.1, 124.2, 123.3, 114.5, 113.2, 55.7, 46.4, 29.8, 25.8, 25.7.

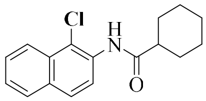

***N*-(1-chloronaphthalen-2-yl)cyclohexanecarboxamide (65)** was synthesized using naphthalen-2-amine (716 mg, 5 mmol, 1.0 equiv.), cyclohexanecarbonyl chloride (700 µL, 5.25 mmol, 1.05 equiv.) and following general procedure E-A using 20.0 mA constant current for 24 h. Compound **65** was isolated as a pale brownish solid (1.39 g, 97% yield) following work-up without the need for any further purification. ^{1}H NMR (400 MHz, $CDCl_3$) δ 8.55 (d, *J* = 9.0 Hz, 1H), 8.16 (dd, *J* = 8.5, 1.1 Hz, 1H), 7.94 (s, 1H), 7.85 – 7.73 (m, 2H), 7.57 (ddd, *J* = 8.4, 6.9, 1.3 Hz, 1H), 7.46 (ddd, *J* = 8.1, 6.8, 1.2 Hz, 1H), 2.39 (tt, *J* = 11.7, 3.5 Hz, 1H), 2.16 – 1.99 (m, 2H), 1.88 (dt, *J* = 12.0, 3.1 Hz, 2H), 1.74 (dtd, *J* = 11.3, 3.3, 1.6 Hz, 1H), 1.60 (qd, *J* = 12.1, 3.2 Hz, 2H), 1.35 (dddd, *J* = 23.6, 15.3, 12.3, 9.0 Hz, 3H). ^{13}C NMR (101 MHz, $CDCl_3$) δ 174.6, 133.1, 131.2, 130.7, 128.2, 127.6, 127.5, 125.5, 123.9, 120.7, 118.1, 46.9, 29.9, 25.8, 25.8.

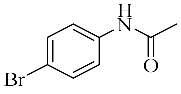

***N*-(4-bromophenyl)acetamide (78)** was synthesized using aniline (466 mg, 5 mmol, 1.0 equiv.), acetyl bromide (388 µL, 5.25 mmol, 1.05 equiv.) and following general procedure E-A using 100.0 mA constant current for 23 h. Compound **78** was purified using flash chromatography (silica gel, gradient elution 5-30% EtOAc in isohexane) as a white solid (870 mg, 82% yield). ^{1}H NMR (400 MHz, $CDCl_3$) δ 7.46 – 7.37 (m, 4H), 7.34 (s, 1H), 2.17 (s, 3H). ^{13}C NMR (101 MHz, $CDCl_3$) δ 168.4, 137.1, 132.1, 121.5, 117.0, 24.7.

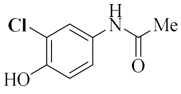

***N*-(3-chloro-4-hydroxyphenyl)acetamide (153)** was synthesized using 4-aminophenol (654 mg, 6 mmol, 1.0 equiv.), acetyl chloride (450 μL, 6.3 mmol, 1.05 equiv.) and following general procedure H-A using 5 mA constant current for 65 h. Compound **153** was isolated using automated flash chromatography (silica gel, gradient elution 100% EtOAc) as a pale white solid (550.0 mg, 50% yield). ^{1}H NMR (400 MHz, DMSO-d$_6$) δ 9.84 (s, 1H), 9.81 (s, 1H), 7.68 (d, *J* = 2.6 Hz, 1H), 7.22 (dd, *J* = 8.8, 2.5 Hz, 1H), 6.88 (d, *J* = 8.7 Hz, 1H), 1.99 (s, 3H).^{13}C NMR (101 MHz, DMSO-d$_6$) δ 167.8, 148.7, 131.8, 120.6, 119.1, 119.0, 116.4, 23.8.

List of reported compounds:

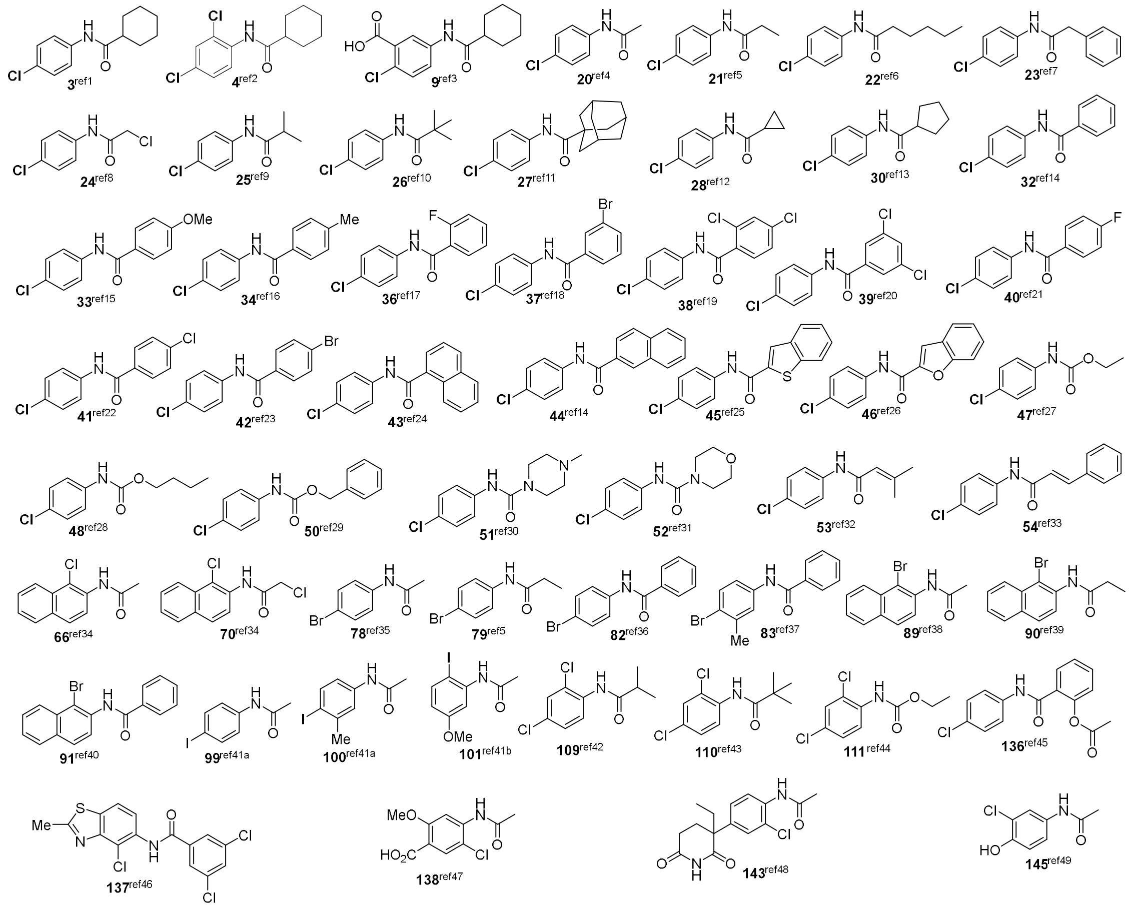

Computational Studies

Computational approach

All computations were carried out with the Gaussian 16[50] software and the ωB97X-D functional.[51] For geometry optimizations, we applied the Def2-SVP basis set[52] and the implicit SMD solvation model[53] to account for the *N*,*N*-dimethylacetamide (DMA; ε = 37.78) media. The located structures were characterized as energy minima or transition states (TSs) based on the number of imaginary vibrational frequencies (zero or one). From the TSs, we followed the

intrinsic reaction coordinate (IRC) pathways in both forward and reverse directions, using a Hessian-based predictor-corrector algorithm,[54] and we identified the related intermediates accordingly. The thermal and entropic corrections were also estimated at this level of theory (i.e., using ωB97X-D/def2-SVP in the solution phase) and within Grimme's quasi rigid rotor harmonic oscillator approximation (q-RRHO).[55] The Gibbs free energies were calculated at room temperature (298.15 K) and were corrected to the standard 1 mol/dm^3 concentration. The concentration of DMA in the bulk solvent was estimated to be 10.8 mol/dm^3 and the related free energy correction was computed accordingly.

In order to estimate the electronic energy more accurately, we calculated the gas-phase electronic energy, applying the Def2-TZVPP basis set, and added the solvation free energy and the thermal and entropic corrections to this electronic energy. The sum of these corrections was estimated by subtracting the gas phase electronic energy calculated at the level of optimization (ωB97X-D/Def2-SVP) from the sum of solution-phase electronic energy and thermal and entropic corrections. The total Gibbs free energy of a structure was calculated according to equation 1, where $E'^{\,\mathrm{gas}}_{\circ}$ is the ωB97X-D/Def2-TZVPP electronic energy, $G^{\mathrm{solv}}_{\circ}$ denotes solution-phase Gibbs free energies obtained from *ω*B97X-D/def2-SVP/SMD(DMA) calculations, $E'^{\,\mathrm{gas}}_{\circ}$ is the ωB97X-D/Def2-SVP electronic energy, and ΔG_{conc} is the concentration correction (1.89 and 3.30 kcal/mol for 1.0 and 10.8 mol/dm^3 concentrations).

$$G = E'^{\,\mathrm{gas}}_{\circ} + \left(G^{\mathrm{solv}}_{\circ} - E^{\mathrm{gas}}_{\circ}\right) + \Delta G_{\mathrm{conc}} \qquad (1)$$

Computational results

Mechanistic pathways relevant to the chlorination phase of the reported reaction were examined computationally via DFT calculations. We thus infere that the amide formation between the amine and acid chloride reactants is completed before the electrochemical process, and the chlorination of the amide takes place in a consecutive step after the oxidation of chloride ions. In constructing the molecular model, we assumed that the chlorination proceeds in the bulk solvent (dimethylacetamide, DMA), therefore, the electrode was excluded from consideration. Given the sensitivity of the reaction to the solvent choice, a few DMA molecules were explicitly included in our models as described below. Cl^+ and Cl_2 were considered as possible active chlorinating agents. The main goal of our computational analysis was to provide a reasonable rationale for the *para* selectivity of the reported electrochemical amidation/C-H halogenation cascade reactions. Towards this end, we examined a model reaction that involves the amide (**am**) derived from aniline and acetyl chloride, Cl^+ or Cl_2 as the electrophile, resulting in various isomeric forms of the chlorinated product (**amCl**) (Scheme S8).

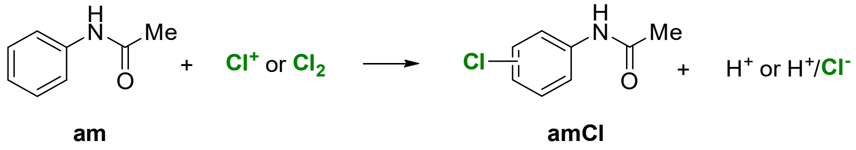

Scheme S8: Chlorination reaction examined computationally.

C1 – Stability of σ-complexes

Arenium ions, the so-called σ-complexes, are generally considered as key intermediates in electrophilic aromatic substitution (EAS) reactions.[56] Their formation is often rate-determining, thus affecting the regioselectivity of these reactions.[57] DFT calculations carried out for the three isomeric forms of the σ-complex intermediate of the investigated reaction predict the *para* isomer to be clearly favored thermodynamically over the other forms (Figure S3). Although these results are consistent with the observed *para* selectivity, further insight into the mechanism of the formation of these intermediates is required.

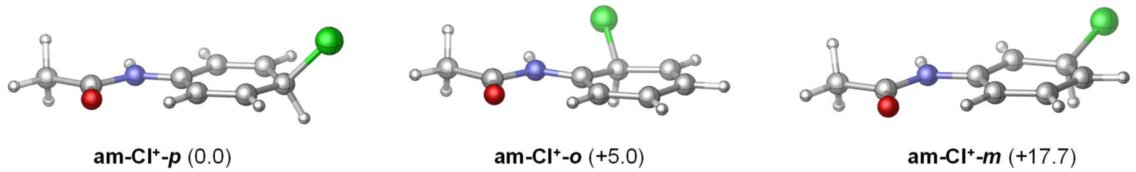

Figure S3: Computed structures and relative stabilities (in kcal/mol) of the isomeric forms of **am-Cl**$^+$ arenium intermediate (*para*, *ortho* and *meta* isomers, respectively).

In our present work, we focus on the reaction pathways leading to the *para* and *ortho* product isomers. The amide substituent on the aromatic ring is a typical *ortho*/*para* directing group, which is borne out by the predicted stability trend (highly unfavored *meta* isomer).

C2 – Cl$^+$ as a chlorinating species

Our electrochemical experiments provide no clear evidence for the nature of the electrophilic chlorine species, however, the formation of Cl^+ via $2e^-$ oxidation might be possible. If so, the Cl^+ likely interacts with the solvent molecules prior to the electrophilic attack event. Our DFT calculations indicate that Cl^+ interacts preferentially with the N atom of the DMA molecule, forming an N-Cl covalent bond (Figure S4). The carbonyl O atom of DMA is an alternative binding site for Cl^+, but this species is predicted to be 8.7 kcal/mol less stable. The electrophilicity of the Cl center in **dma-Cl**$^+$ is significantly reduced, therefore interaction with an additional DMA molecule gives only a weakly bound complex (**dma-Cl**$^+$**···dma** in Figure S2), which is predicted to be 4.7 kcal/mol above the **dma-Cl**$^+$ + **dma** state in Gibbs free energy.

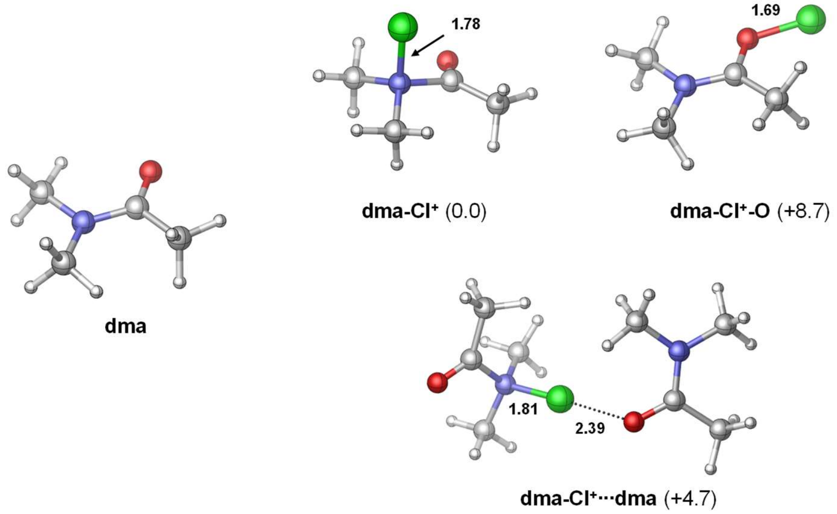

Figure S4: Computed structures and relative stabilities (in kcal/mol) of species derived from the interaction of Cl^{+} and solvent DMA molecules. The stability of the **dma-Cl⁺···dma** species is given with respect to the **dma-Cl⁺ + dma** dissociated state. Selected bond distances are given in Å.

Chlorenium transfer from **dma-Cl⁺** to amide **am** was examined next computationally. In these calculations, an additional solvent DMA molecule was explicitly included in the model to take into account H-bonding interactions between the amide and the solvent molecules. Indeed, a relatively strong N-H···O hydrogen bond is revealed by DFT calculations as illustrated in Figure S5. The formation of the H-bonded **am-dma** complex is predicted to be only slightly endergonic with respect to the dissociation limit, so this species is likely present in DMA solution.

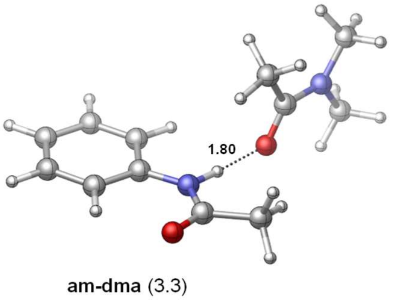

Figure S5: Computed structure of the H-bonded **am-dma** dimer. The relative stability is given in kcal/mol with respect to the **am + dma** dissociated state. The selected H···O bond distance is in Å.

Weakly bound complexes between **am-dma** and the electrophilic **dma-Cl⁺** species could be identified computationally. In Figure S6, complexes corresponding to the electrophilic attack of **dma-Cl⁺** at the aromatic carbon atoms in the *para* (*p*) and the two *ortho* (o_1 and o_2) positions of the **am** amide are depicted. These transient species, also referred to as π-complexes, can be

considered as the reactant states of the Cl^+ transfer elementary step, and they are predicted to be about 8 kcal/mol above the **dma-Cl⁺** + **am-dma** state.

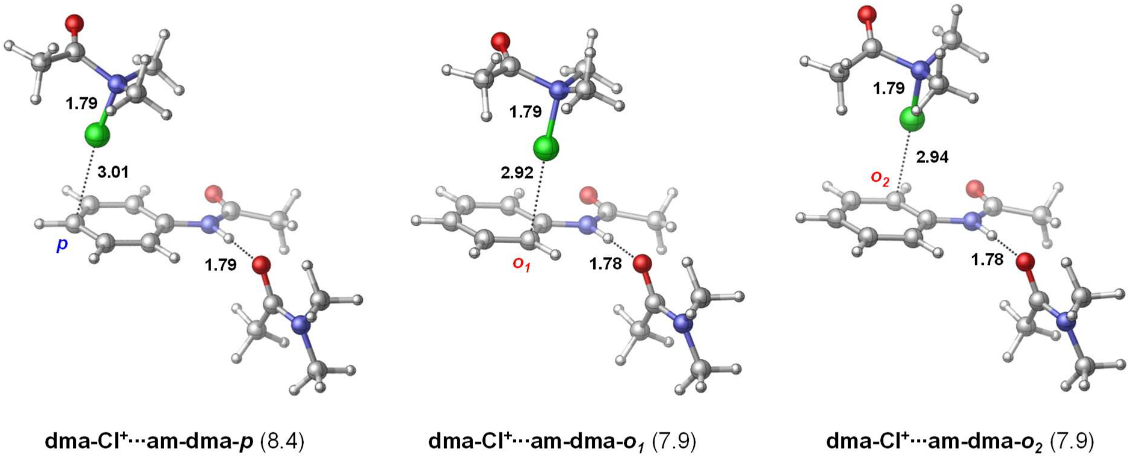

Figure S6: Computed structures of weakly bound **dma-Cl⁺···am-dma** species corresponding to the electrophilic attacks at the aromatic carbon atoms in the *para* (*p*) and the two *ortho* (o_1 and o_2) positions of amide **am**. Relative stabilities are given in kcal/mol with respect to the **dma-Cl⁺** + **am-dma** dissociated state. Selected bond distances are in Å.

Potential energy scan calculations were carried out to map the energetics of the Cl^+ transfer along the *para* and *ortho* pathways, and the results pointed to an early TS region and a substantial energy gain along the pathways towards the σ-complex intermediates (Figure S7).

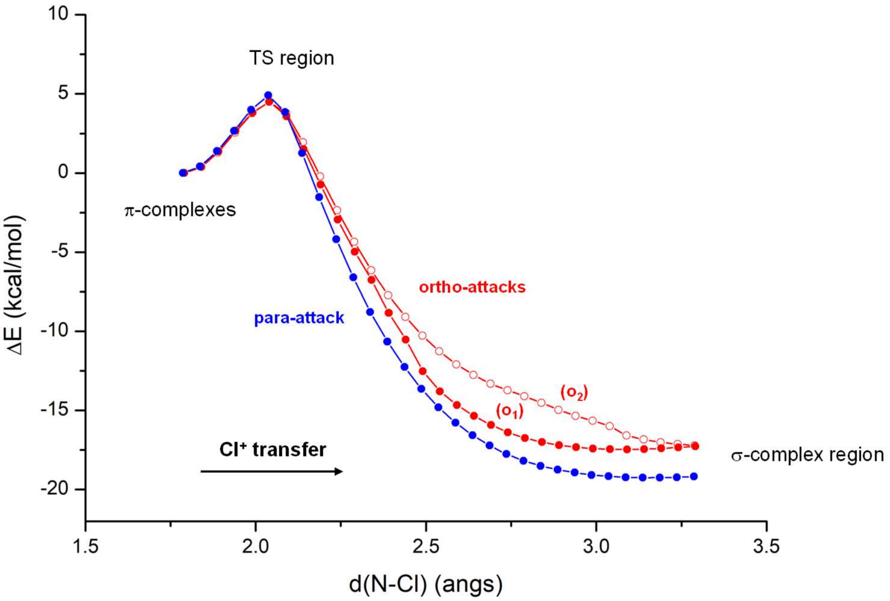

Figure S7: Energies computed along the *para* and *ortho* Cl^+ transfer pathways via potential energy scan calculations. π-complexes shown in Figure S4 were used as initial structures, and the energies were computed by systematic variation of the N-Cl bond of **dma-Cl⁺** (lengthening gradually by 0.05 Å), while optimizing all other structural parameters.

The early nature of the transition states is apparent from their optimized structures as well, which are depicted in Figure S8. For instance, the N-Cl bond of the **dma-Cl⁺** reactant is lengthened only by 0.25 Å in these TSs, whereas the forming C-Cl bonds are still fairly long (around 2.3 Å). In the corresponding product states (σ-complex intermediates depicted in Figure S9), the latter bonds are about 1.8 Å long, and the DMA molecules that provided the chloreniums are shifted away from the Cl atoms and interact weakly with the chlorinated species.

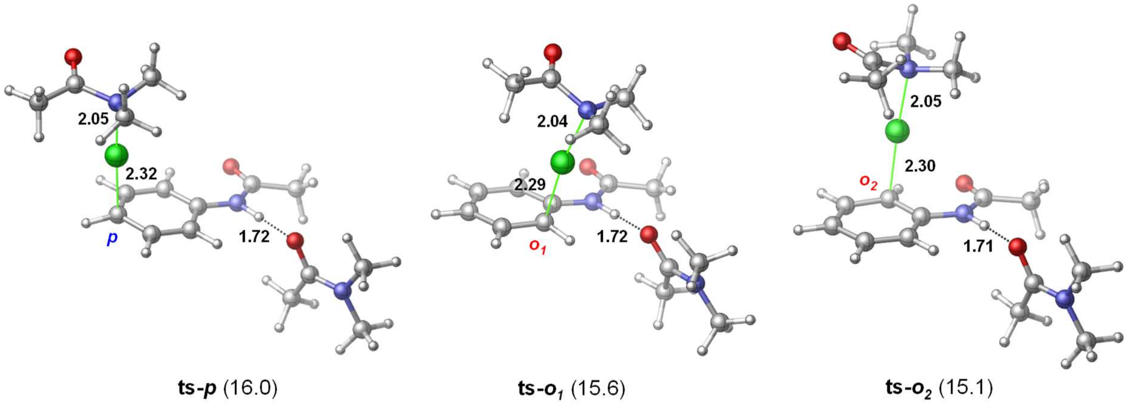

Figure S8: Transition states identified computationally along the *para* and *ortho* Cl^+ transfer pathways in the model reaction between **dma-Cl⁺** and **am-dma**. Relative stabilities are given in kcal/mol with respect to the **dma-Cl⁺** + **am-dma** dissociated state. Selected bond distances are in Å.

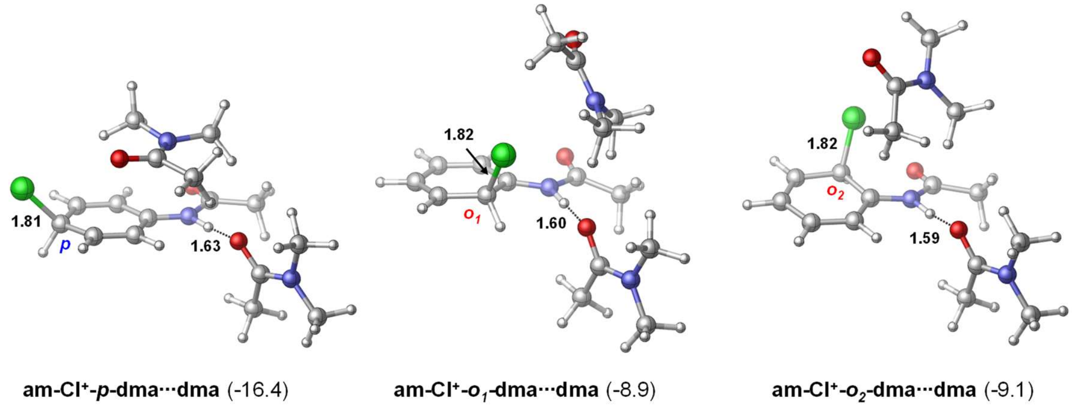

Figure S9: Computed structures of the product states (σ-complex intermediates) formed upon the *para* and *ortho* Cl^+ transfer pathways in the model reaction between **dma-Cl⁺** and **am-dma**. Relative stabilities are given in kcal/mol with respect to the **dma-Cl⁺** + **am-dma** dissociated state. Selected bond distances are in Å.

In line with the early nature of the Cl^+ transfer transition states, no kinetic preference for the formation of the experimentally observed *para*-chlorinated product is found within the framework of the present model. This is revealed by the TS region of the computed potential energy curves shown in Figure S6, and also by the relative stabilities of the identified transition

states, which are all within 1 kcal/mol, showing even slight preference for the *ortho* pathways (Figure S8).

C3 – Cl_2 as a chlorinating species

Chloride oxidation may also give rise to Cl_2, which can act as an electrophile in the reaction with amide **am**. Molecular models involving the H-bonded **am-dma** complex, a Cl_2 molecule and an additional DMA molecule were constructed to explore the energetics of electrophilic attacks at the *para* and *ortho* positions of the **am** amide. The π-complexes formed between **am-dma** and the Cl_2 molecule were considered as the reactant states in this model, which could be identified computationally (see Figure S10). In these structures, the Cl_2 molecules reside in the on-top positions of the actual aromatic carbon atoms and they also interact weakly with the nitrogen of the DMA molecule. The relative stabilities of the three species are very similar. We note here that in the solution, several additional DMA molecules are present in the vicinity of the reacting Cl_2 molecules, so the choice for the position of a single DMA molecule that interacts with Cl_2 seems arbitrary. However, in our present approach, we intended to avoid any contact between this DMA and the amide substrate to exclude the energy bias arising from these contacts, which would alter the energetics of various reaction pathways. For this reason, our molecular model is expected to give only a qualitative mechanistic picture about the chlorination event because the interaction between Cl_2 and the solvent environment is simplified. Nevertheless, we think the model is able to accentuate the role of the solvent molecules in the investigated electrophilic aromatic substitution reaction.

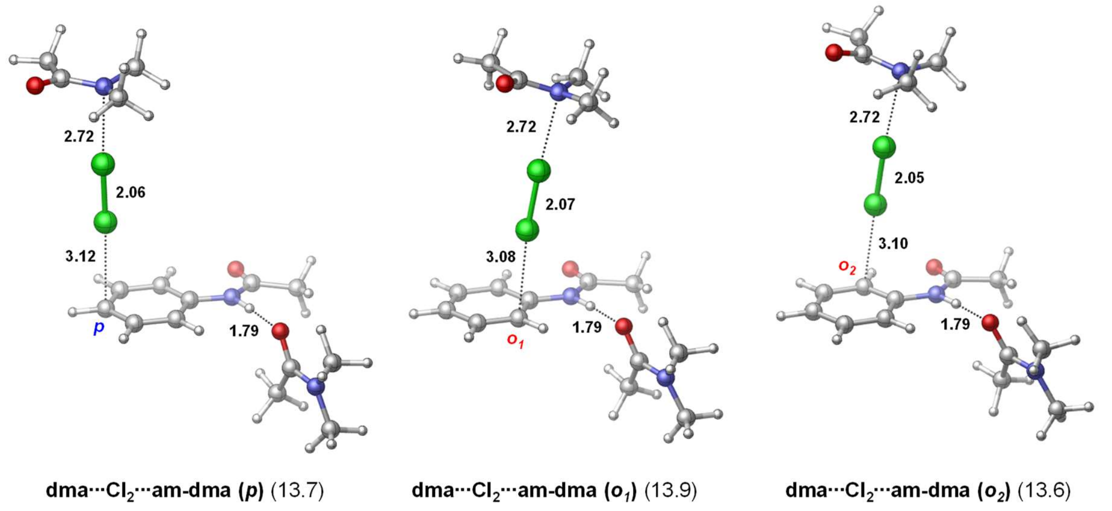

Figure S10: Computed structures of weakly bound **dma···Cl_2···am-dma** species considered as reactant states for the electrophilic attack of Cl_2 at the aromatic carbon atoms in the *para* (*p*) and the two *ortho* (o_1 and o_2) positions of amide **am**. Relative stabilities are given in kcal/mol with respect to the **dma** + **Cl_2** + **am-dma** dissociated state. Selected bond distances are in Å.

The potential energy surfaces of the electrophilic attack along the three pathways (p, o_1 and o_2) were mapped via constrained geometry optimizations, wherein the distance between the attacking Cl and the aromatic C atoms was used as an approximate reaction coordinate (Figure S11).

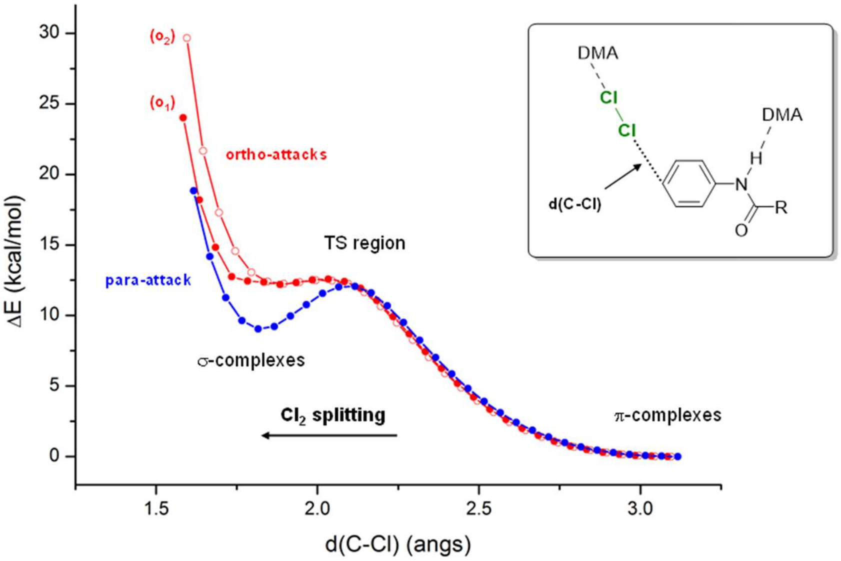

Figure S11: Energies computed along the *para* and *ortho* electrophilic Cl_2 attack pathways via potential energy scan calculations. π-complexes shown in Figure S9 were used as initial structures, and the energies were computed by systematic variation of the C-Cl bond (shortening gradually by 0.05 Å), while optimizing all other structural parameters.

The computed potential energy curves imply that the formation of the heterolytic splitting of Cl_2 leading to the σ-complex intermediates is endothermic and a clear difference in the shape of the curves computed for the *para* and *ortho* attacks is apparent. Namely, the σ-complex formed along the *para* pathway is predicted to be in a well-defined energy minimum, whereas this region of the potential energy surface, as well as the TS region, is found to be rather flat for the two *ortho* pathways. The σ-complex intermediates could be identified computationally for all three pathways (see Figure S12), and the obtained structures uncover a few important features highlighting the role of the solvent molecules in this mechanistic scenario.

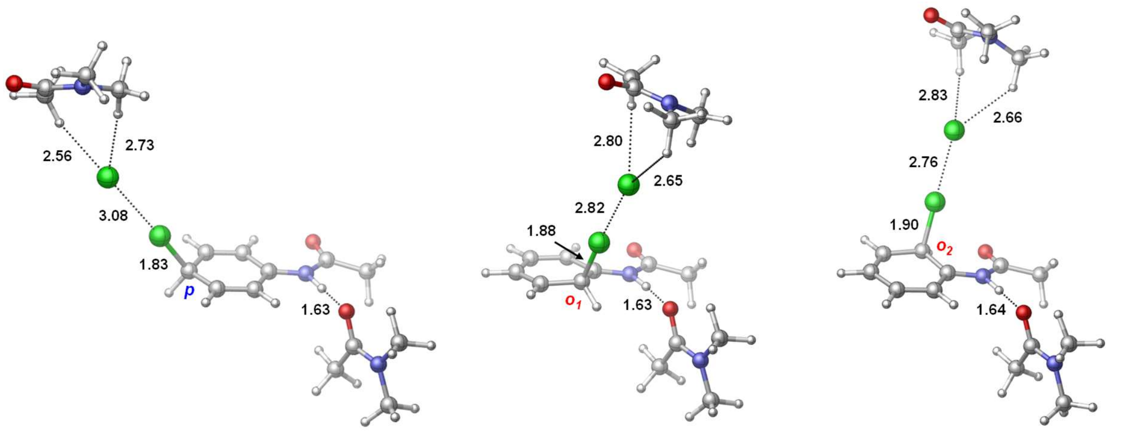

dma(Cl⁻)···am-Cl⁺-*p*-dma (18.3) **dma(Cl⁻)···am-Cl⁺-*o*$_1$-dma** (25.1) **dma(Cl⁻)···am-Cl⁺-*o*$_2$-dma** (26.4)

Figure S12: Computed structures of σ-complex intermediates formed upon the electrophilic attack of Cl_2 at the aromatic carbon atoms in the *para* (*p*) and the two *ortho* (o_1 and o_2) positions of amide **am**. Relative stabilities are given in kcal/mol with respect to the **dma** + **Cl_2** + **am-dma** state. Selected bond distances are in Å.

First, the Cl^- anion generated upon the heterolytic Cl_2 cleavage is stabilized by the adjacent DMA molecule via C-H···Cl^- interactions. The stabilization is likely enhanced by additional DMA molecules in the solution. Furthermore, the N-H···O hydrogen bond between **am** and DMA is notably strengthened upon the chlorination step due to the cationic nature of the arenium intermediate. This is apparent from the variation of the H···O bond distances (1.79 Å versus 1.63 Å in π- and σ-complexes, respectively; see Figures S10 and S12). As expected from the shape of the computed potential energy curves (Figure S11), the transient σ-complex species formed along the *para* attack pathway (**dma(Cl⁻)···am-Cl⁺-*p*-dma** in Figure S12) is significantly more favored than those identified on the *ortho* pathways. This is in line with the trend found in the relative stabilities of the **am-Cl⁺-*p*** and **am-Cl⁺-*o*** species (Figure S3).

It is important to note that both C-H···Cl^- and N-H···O stabilizing interactions are already significant in the transition state (TS) region of the electrophilic attack, as illustrated in Figure S13. Herein, the C-H···Cl and N-H···O bond distances are depicted with respect to d(Cl-C) (blue curves), along with the variation of the Cl···Cl bond distance (green curve). In the TS region, the heterolytic Cl_2 cleavage is in an advanced state, and the evolving charged species (Cl^- and cationic σ-complex) are stabilized by increased C-H···Cl^- and N-H···O interactions, which is reflected in significantly decreased C-H···Cl and N-H···O bond distances. These stabilizing effects highlight the catalytic role of solvent DMA molecules.

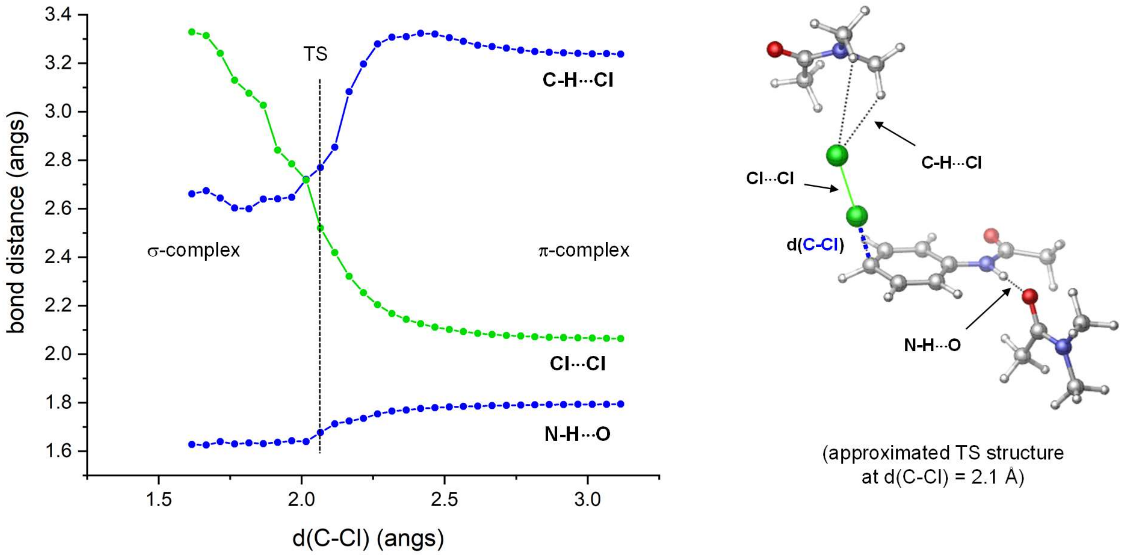

Figure S13: Variation of selected bond distances along the d(C-Cl) parameter used in potential energy scan calculations (see Figure S12). C-H···Cl and N-H···O bond distances are depicted in blue, Cl···Cl in green. The transition state region (corresponding to the energy maximum) is highlighted by a vertical dashed line. The structure corresponding to the energy maximum (transition state region) is shown on the right with highlighted bond distances.

Our attempts to fully optimize the geometry of the transition states on the potential energy surface that connect the identified π-complex and σ-complex species along the *para* and *ortho* pathways were unsuccessful. In all these TS search calculations, the DMA molecule interacting with the Cl^- anion drifted toward the **am** molecule, forming contacts with the amide, and optimization converged to either π- or σ-complexes. These results indicate that other structural arrangements of π- and σ-complex intermediates, involving various DMA···**am** contacts, exist as local minima on the potential energy surface, but such structures introduce energy biases in our present analysis.

Nevertheless, the shape of the potential energy curves shown in Figure S11 imply no or very small difference in the energies of different pathways in the transition state region, so one expects similar barriers for the formation *para* and *ortho* σ-complex intermediates. However, the *ortho* σ-complexes lie in extremely shallow energy minima, so they can very easily transform back to the reactant states (i.e., the π-complex intermediates). The energy barrier of back-transformation on the *para* pathway is estimated to be about 4-5 kcal/mol from the computed potential energy curve, which is not high either, but could be comparable, or even higher than that of the deprotonation steps that generate the final chlorinated product. The solvent DMA molecule can act as a base in the deprotonation process. Indeed, our calculations predict an extremely low energy barrier and high exothermicity for this step (see Figures S14

and S15), implying that this step is likely diffusion-controlled. The Gibbs free energy barrier of diffusion-controlled reactions is estimated to be roughly 4 kcal/mol.[58]

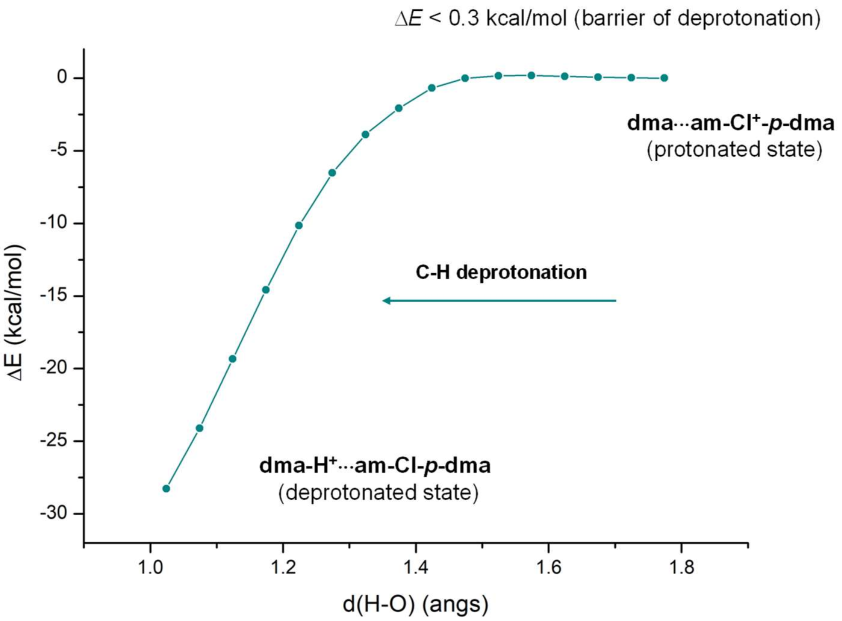

Figure S14: Energies computed for the deprotonation of **am-Cl$^+$-*p*-dma** with DMA as a base via potential energy scan calculations. The optimized structure of the protonated state (**dma···am-Cl$^+$-*p*-dma** complex shown in Figure S14, left) was used as the initial structure, and the energies were computed by systematic shortening of the O-H bond relevant to deprotonation. All other structural parameters were optimized.

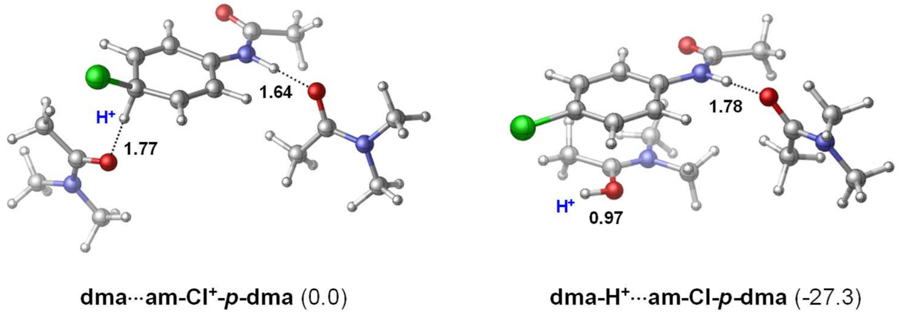

Figure S15: Computed structures of the protonated and deprotonated states of the chlorinated amide **am**. Relative stabilities are given in kcal/mol. Selected bond distances are in Å.

These results thus suggest that the regioselectivity of the present chlorination reactions may be controlled kinetically as illustrated qualitatively in Figure S16. Assuming Cl_2 as the chlorinating agent, the chlorination can take place via the formation of σ-complex intermediates, which are subsequently deprotonated by the solvent DMA molecules. The regioselectivity essentially arises from the interplay of the transient nature of these intermediates, the thermodynamic preference of the *para* form, and the facile deprotonation

step leading to the overall kinetic regioselectivity control. We note that such interplay cannot be expected if **dma-Cl**$^+$ is the chlorinating agent, as in this case, the formation of the σ-complex is already irreversible (Figure S9). We also note that this qualitative mechanistic picture has emerged using transition state theory (TST), which has its limitations, so we think that more advanced modeling approaches, most likely molecular dynamics simulations on extended solvent phase systems are required to account for the regioselectivity quantitatively.[59]

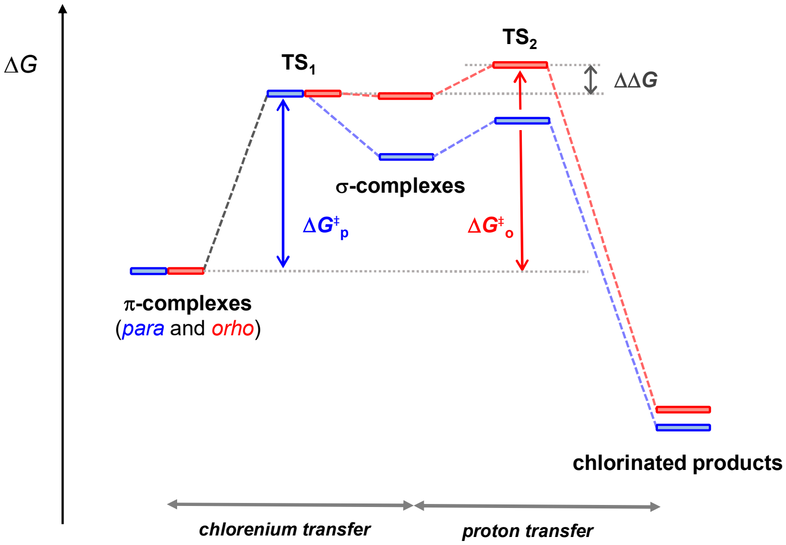

Figure S16: Schematic view of the energetics of regioselective chlorination of amides assuming Cl_2 as chlorinating agent. TS_1 and TS_2 represent transition states of the chlorenium and proton transfer steps. $\Delta G^{\ddagger}_{p}$ and $\Delta G^{\ddagger}_{o}$ denote rate determining barriers along the *para* and *ortho* reaction pathways, and $\Delta\Delta G = \Delta G^{\ddagger}_{p} - \Delta G^{\ddagger}_{o}$ is the measure of selectivity.

C4 – Computed energy components of the reported structures

Table S3: Energy data (in a.u.) computed for ωB97X-D/def2-SVP/SMD(DMA) optimized structures.[a]

	$G_{\circ}^{\mathrm{solv}}$	$E_{\circ}^{\mathrm{gas}}$	$E'^{\,gas}_{\circ}$	G
	σ-complex intermediates			
**am-Cl⁺-*p***	-899.5511	-899.5734	-900.2129	-900.1876
**am-Cl⁺-*o***	-899.5449	-899.5708	-900.2085	-900.1795
**am-Cl⁺-*m***	-899.5231	-899.5403	-900.1795	-900.1593
	Cl⁺ as electrophile			
Cl⁺-DMA interactions				
**dma**	-287.4438	-287.5326	-287.8544	-287.7603
**dma-Cl⁺**	-747.2625	-747.2656	-747.7473	-747.7412
**dma-Cl⁺-O**	-747.2477	-747.2592	-747.7418	-747.7273
**dma-Cl⁺···dma**	-1034.6972	-1034.8290	-1035.6287	-1035.4939
H-bonded am-dma				
**am-dma**	-727.1404	-727.3613	-728.1573	-727.9333
**am**	-439.7023	-439.8069	-440.2859	-440.1783
Cl⁺-transfer				
**dma-Cl⁺···am-dma-*p***	-1474.3883	-1474.6545	-1475.9304	-1475.6611
**ts-*p***	-1474.3802	-1474.6628	-1475.9346	-1475.6490
**am-Cl⁺-*p*-dma···dma**	-1474.4309	-1474.7074	-1475.9803	-1475.7007
**dma-Cl⁺···am-dma-*o1***	-1474.3893	-1474.6556	-1475.9312	-1475.6620
**ts-*o1***	-1474.3812	-1474.6633	-1475.9347	-1475.6496
**am-Cl⁺-*o1*-dma···dma**	-1474.4167	-1474.6867	-1475.9612	-1475.6881
**dma-Cl⁺···am-dma-*o2***	-1474.3895	-1474.6586	-1475.9341	-1475.6620
**ts-*o2***	-1474.3819	-1474.6662	-1475.9377	-1475.6504
**am-Cl⁺-*o2*-dma···dma**	-1474.4183	-1474.6884	-1475.9622	-1475.6890
**dma-Cl⁺···am-dma-*p***	-1474.3883	-1474.6545	-1475.9304	-1475.6611
**ts-*p***	-1474.3802	-1474.6628	-1475.9346	-1475.6490
**am-Cl⁺-*p*-dma···dma**	-1474.4309	-1474.7074	-1475.9803	-1475.7007
	Cl_2 as electrophile			
π-complexes				
**Cl_2**	-920.0845	-920.0609	-920.3861	-920.4066
**dma···Cl_2···am-dma (*p*)**	-1934.6435	-1934.9680	-1936.4060	-1936.0785
**dma···Cl_2···am-dma (*o1*)**	-1934.6434	-1934.9685	-1936.4063	-1936.0782
**dma···Cl_2···am-dma (*o2*)**	-1934.6438	-1934.9682	-1936.4060	-1936.0787
σ-complexes				
**dma(Cl⁻)···am-Cl⁺-*p*-dma**	-1934.629	-1934.8972	-1936.3424	-1936.0712
**dma(Cl⁻)···am-Cl⁺-*o1*-dma**	-1934.6232	-1934.912	-1936.3521	-1936.0603
**dma(Cl⁻)···am-Cl⁺-*o2*-dma**	-1934.6228	-1934.9129	-1936.3513	-1936.0582
dissociated σ-complexes				
**am-Cl⁺-*p*-dma**	-1186.9942	-1187.1534	-1188.109	-1187.9468
**am-Cl⁺-*o1*-dma**	-1186.9874	-1187.1465	-1188.1011	-1187.9390

**am-Cl⁺-*o2*-dma**	-1186.9874	-1187.1479	-1188.1023	-1187.9387
deprotonation				
**dma···am-Cl⁺-*p*-dma**	-1474.4287	-1474.7136	-1475.9864	-1475.6984
**dma-H⁺···am-Cl-*p*-dma**	-1474.4723	-1474.7501	-1476.0227	-1475.7419

[a] Notation: E_o ^{gas} and E_o' ^{gas} refer to gas-phase electronic energies computed at ωB97X-D/def2-SVP and ωB97X-D/def2-TVPP levels of DFT; G_o ^{solv} denotes solution-phase Gibbs free energies obtained from ωB97X-D/def2-SVP calculations (T = 298.15 K). The last column is obtained as $G = E_{\circ}'^{\,gas} + \left(G_{\circ}^{\text{solv}} - E_{\circ}^{\text{gas}}\right) + \Delta G_{\text{conc}}$ where the last term is the concentration correction (see Computational approach section above). The relative stabilities discussed in the manuscript and in the SI are obtained from *G* values compiled herein.

References

1. Lu, L.; Qiu, F.; Alhumade, H.; Zhang, H.; Lei, A. Tuning the Oxidative Mono- or Double-Carbonylation of Alkanes with CO by Choosing a Co or Cu Catalyst. *ACS Catal.* **2022**, *12*, 9664-9669.

2. Yan, Q.; Yuan, Q.-J.; Shatskiy, A.; Alvey, G. R.; Stepanova, E. V.; Liu, J.-Q.; Kärkäs, M. D.; Xiang-Shan Wang, X.-S. General Approach to Amides through Decarboxylative Radical Cross-Coupling of Carboxylic Acids and Isocyanides. *Org. Lett.* **2024**, *26*, 3380-3385.

3. Preparation of 2-amino-5-substituted pyrimidine kinase inhibitors and use in therapy Assignee: Targegen, Inc. World Intellectual Property Organization, WO2008008234 A1 2008-01-17.

4. Misal, B.; Palav, A.; Ganwir, P.; Chaturbhuj, G. Activator free, expeditious and eco-friendly chlorination of activated arenes by *N*-chloro-*N* (phenylsulfonyl)benzene sulfonamide (NCBSI). *Tetrahedron Lett.* **2021**, *63*, 152689.

5. Luo, Q.-L.; Lv, L.; Li, Y.; Tan, J.-P.; Nan, W.; Hui, Q. An Efficient Protocol for the Amidation of Carboxylic Acids Promoted by Trimethyl Phosphite and Iodine. *Eur. J. Org. Chem.* **2011**, ~~*2011*,~~ 6916-6922.

6. Zuo, D.; Wang, Q.; Liu, L.; Huang, T.; Szostak, M.; Chen, T. Highly Chemoselective Transamidation of Unactivated Tertiary Amides by Electrophilic N-C(O) Activation by Amide-to-Acyl Iodide Re-routing. *Angew. Chem. Int. Ed.* **2022**, *61*, e202202794.

7. (a) Basavaprabhu, Muniyappa, K.; Panguluri, N. R.; Veladi, P.; Sureshbabu, V. V. A simple and greener approach for the amide bond formation employing $FeCl_3$ as a catalyst. *New J. Chem.* **2015**, *39*, 7746-7749. (b) Lal, S.; Snape, T. J. Towards a sustainable synthesis of aniline-derived amides using an indirect chemoenzymatic process: challenges and successes. *RSC Adv.* **2014**, *4*, 1609-1615.

8. Schweda, S. I.; Alder, A.; Gilberger, T.; Kunick, C. 4-Arylthieno[2,3-*b*]pyridine-2-carboxamides are a new class of antiplasmodial agents. *Molecules* **2020**, *25*, 3187.

9. Mei, C; Lu, W. Palladium(II)-Catalyzed Oxidative Homo- and Cross-Coupling of Aryl ortho-sp^2 C-H Bonds of Anilides at Room Temperature. *J. Org. Chem.* **2018**, *83*, 4812-4823.

10. Nguyen, T. T.; Hull, K. L. Rhodium-Catalyzed Oxidative Amidation of Sterically Hindered Aldehydes and Alcohols. *ACS Catalysis* **2016**, *6*, 8214-8218.

11. Leiva, R.; Barniol-X., M.; Codony, S.; Ginex, T.; Vanderlinden, E.; Montes, M.; Caffrey, M.; Luque, F. J.; Naesens, L.; Vázquez, S. Aniline-Based Inhibitors of Influenza H1N1 Virus Acting on Hemagglutinin-Mediated Fusion. *J. Med. Chem.* **2018**, *61*, 98-118.

12. Li, Q.; Dai, P.; Tang, H.; Zhang, M.; Wu, J. Photomediated reductive coupling of nitroarenes with aldehydes for amide synthesis. *Chem. Sci.* **2022**, *13*, 9361-9365.

13. Wang, S.-P.; Cheung, C. W.; Ma, J.-A. Direct Amidation of Carboxylic Acids with Nitroarenes. *J. Org. Chem.* **2019**, *84*, 13922-13934.

14. Štrukil, V.; Bartolec, B.; Portada, T.; Đilović, I.; Halasz, I.; Margetić, D. One-pot mechanosynthesis of aromatic amides and dipeptides from carboxylic acids and amines. *Chem. Commun.* **2012**, *48*, 12100-12102.

15. Zhao, Z.; Ikawa, S.; Mori, S.; Sumii, Y.; Adachi, H.; Kagawa, T.; Shibata, N. Mechanochemical Deoxyfluorination of Carboxylic Acids to Acyl Fluorides and Successive Mechanochemical Amide Bond Formation. *ACS Sustain. Chem. Eng.* **2024**, *12*, 3565-3574.

16. (a) Mart, M.; Tylus, W.; Trzeciak, A. M. Pd/DNA as a highly active and recyclable catalyst for aminocarbonylation and hydroxycarbonylation in water: The effect of $Mo(CO)_6$ on the reaction course. *Mol. Catal.* **2019**, *462*, 28-36. (b) Teo, Y.-C.; Yong, F.-F.; Ithnin, I. K.; Yio, S.-H. T.; Lin, Z. Efficient Manganese/Copper Bimetallic Catalyst for N-Arylation of Amides and Sulfonamides Under Mild Conditions in Water. *Eur. J. Org. Chem.* **2013**, *2013*, 515-524.

17. Deng, M.; Liu, K.; Ma, Z.; Luo, G.; Dian, L. Photo-Induced $FeCl_3$-catalysed direct denitrative chlorination of (hetero)nitroarenes at room temperature. *Green Chem.* **2024**, *26*, 11556-11562.

18. Wood, D. J.; Lopez-Fernandez J. D.; Knight, L. E.; Al-Khawaldeh, I.; Gai, C.; Lin, S.; Martin, M. P.; Miller, D. C.; Cano, C.; Endicott, J. A.; Hardcastle, I. R.; Noble, M. E. M.; Waring, M. J. FragLites-Minimal, Halogenated Fragments Displaying Pharmacophore Doublets. An Efficient Approach to Druggability Assessment and Hit Generation. *J. Med. Chem.* **2019**, *62*, 3741-3752.

19. Fanga, Y.; Tranmer, G. K. Continuous flow photochemistry as an enabling synthetic technology: synthesis of substituted-6(5H)-phenanthridinones for use as poly(ADP-ribose) polymerase inhibitors. *MedChemComm* **2016**, *7*, 720-724.

20. Zhang, J.; Li, Y.; Wang, B.; Jia, A.-Q.; Zhang, Q.-F. Synthesis of Dichlorobenzamide Derivatives: Crystal Structures of 3,5-Dichloro-*N*-(2-chlorophenyl)benzamide and 3,5-Dichloro-*N*-(4-chlorophenyl)benzamide. *J. Chem. Crystallogr.* **2021**, *51*, 108-115.

21. (a) Cheng, C.; Sun, G.; Wan, J.; Sun, C. Facile CuI-catalyzed arylation of azoles and amides using simple enaminones as efficient ligands. *Synlett* **2009**, *16*, 2663-2668. (b) Wang, X.; Liu, J.; An, G. Green synthetic method of N-arylamides using recyclable cheap metal catalyst. *Tetrahedron Lett.* **2020**, *61*, 152327.

22. Van den Nieuwendijk, A. M. C. H.; Pietra, D.; Heitman, L.; Göblyös, A.; IJzerman, A. P. Synthesis and biological evaluation of 2,3,5-substituted [1,2,4]thiadiazoles as allosteric modulators of adenosine receptors. *J. Med. Chem.* **2004**, *47*, 663-672.

23. Zhu, R.; Li, Y.; Shen, Y.; Pan, M.; Dong, W.; Li, W. Visible-light induced $FeCl_3$-catalyzed reductive transamidation of *N*-acyl benzotriazoles with nitro compounds. *Org. Chem. Front.* **2024**, *11*, 2095-2101.

24. Pan, C.; Wang, L.; Han, J. Palladium-Catalyzed Annulation of Arylbenzamides with Diaryliodonium Salts. *Adv. Synth. Catal.* **2022**, *364*, 268-273.

25. Mahajan, P. S.; Nikam, M. D.; Nawale, L. U.; Khedkar, V. M. Sarkar, D.; Gill, C. H. Synthesis and Antitubercular Activity of New Benzo[*b*]thiophenes. *ACS Med. Chem. Lett.* **2016**, *7*, 751-756.

26. Choi, M.; Jo, H.; Park, H.-J.; Kumar, A. S.; Lee, J.; Yun, J.; Kim, Y.; Han, S.-B.; Jung, J.-K.; Cho, J.; Lee, K.; Kwak, J.-H.; Lee, H. Design, synthesis, and biological evaluation of benzofuran- and 2,3-dihydrobenzofuran-2-carboxylic acid N-(substituted)phenylamide derivatives as anticancer agents and inhibitors of NF-κB. *Bioorg. Med. Chem. Lett.* **2015**, *25*, 2545-2549.

27. Chilin, A.; Marzaro, G.; Zanatta, S.; Barbieri, V.; Pastorini, G.; Manzini, P.; Guiotto, A. A new access to quinazolines from simple anilines. *Tetrahedron* **2006**, *62*, 12351-12356.

28. Khatun, R.; Biswas, S.; Islam, S.; Biswas, I. H.; Riyajuddin, SK.; Ghosh, K.; Islam, SK. M. Modified Graphene Oxide Based Zinc Composite: an Efficient Catalyst for N-formylation and Carbamate Formation Reactions Through CO2 Fixation. *ChemCatChem* **2019**, *11*, 1303-1312.

29. Yang, B.; Shi, L.; Wu, J.; Fang, X.; Yang, X.; Wu, F. Microwave-assisted expeditious synthesis of 5-fluoroalkyl-3-(aryl/alkyl)-oxazolidin-2-ones. *Tetrahedron* **2013**, *69*, 3331-3337.

30. Latacz, G.; Kechagioglou, P.; Papi, R.; Dorota Łażewska, D.; Więzcek, M.; Kamińska, K.; Wencel, P.; Karcz, T.; Schwed, J. S.; Stark, H.; Kyriakidis, D. A.; Kieć-Kononowicz, K. The Synthesis of 1,3,5-triazine Derivatives and JNJ7777120 Analogues with Histamine H4 Receptor Affinity and Their Interaction with PTEN Promoter. *Chem. Biol. Drug Des.* **2016**, *88*, 254-263.

31. Pujaria, V. K. Vinnakota, S.; Kakarla, R. K.; Maroju, S. Antimicrobial activity and microwave assisted synthesis of 4-chlorophenyl urea derivatives by using Dabal-Me_3. *Heterocycl. Lett.* **2018**, *8*, 487-491.

32. Shen, S.; Hadley, M.; Ustinova, K.; Pavlicek, J.; Knox, T.; Noonepalle, S.; Tavares, M. T.; Chad A. Zimprich, C. A.; Zhang, G.; Robers, M. B.; Bařinka, C.; Kozikowski, A. P.; Villagra, A. Discovery of a New Isoxazole-3-hydroxamate-Based Histone Deacetylase 6 Inhibitor SS-208 with Antitumor Activity in Syngeneic Melanoma Mouse Models. *J. Med. Chem.* **2019**, *62*, 8557-8577.

33. Rhazri, K.; Izikki, M.; Bouali, J.; S. Hamri, S.; Jabrane, J.; Hajbi, Y.; Hafid, A.; Eddahibi, S.; Khouili, M. Design, synthesis and evaluation of *N*-aryl carboxamide derivatives as potential anti-proliferative effect on the pulmonary artery smooth muscle cells. *J. Pharm. Chem. Biol. Sci.* **2015**, *3*, 122-131.

34. Zhang, D.; Huang, Z.; Lei, A. Oxidation-induced ortho-selective C-H bond functionalization of 2-naphthylamine derivative. *Sci. China Chem.* **2018**, *61*, 1274-1277.

35. Guo, R.; Zhu, C.; Sheng, Z.; Li, Y.; Yin, W.; Chu, C. Silica sulfuric acid mediated acylation of amines with 1,3-diketones via C-C bond cleavage under solvent-free conditions. *Tetrahedron Lett.* **2015**, *56*, 6223-6226.

36. John, J. M.; Loorthuraja, R.; Antoniuk, E.; Bergens, S. H. Catalytic hydrogenation of functionalized amides under basic and neutral conditions. *Catal. Sci. Technol.* **2015**, *5*, 1181-1186.

37. Zhang, R.; Yao, W.-Z.; Qian, L.; Sang, W.; Yuan, Y.; Min-Chen Du, M.-C.; Cheng, H.; Chen, C.; Qin, X. A practical and sustainable protocol for direct amidation of unactivated esters under transition-metal-free and solvent-free conditions. *Green Chem.* **2021**, *23*, 3972-3982.

38. Du, Y.; Xi, Z.; Guo, L.; Lu, H.; Feng, L.; Gao, H. Practical bromination of arylhydroxylamines with $SOBr_2$ towards *ortho*-bromo-anilides. *Tetrahedron Lett.* **2021**, *72*, 153074

39. Preparation of organic bromine-containing compound Assignee: Zhengzhou University China, CN118724712 A 2024-10-01.

40. Du, Y.; Xi, Z.; Guo, L.; Lu, H.; Feng, L.; Gao, H. Practical bromination of arylhydroxylamines with $SOBr_2$ towards ortho-bromo-anilides. *Tetrahedron Lett.* **2021**, *72*, 153074.

41. (a) Shi, C.; Miao, Q.; Ma, L.; Lu, T.; Yang, D.; Chen, J.; Li, Z. Room-Temperature C-H Bromination and Iodination with Sodium Bromide and Sodium Iodide Using *N*-Fluorobenzenesulfonimide as an Oxidant. *ChemistrySelect* **2019**, *4*, 6043 –6047. (b) Michelet, B.; Deldaele, C.; Kajouj, S.; Moucheron, C.; Evano, G. A General Copper Catalyst for Photoredox Transformations of Organic Halides. *Org. Lett.* **2017**, *19*, 3576-3579.

42. Singh, H.; Sen, C.; Sahoo, T.; Ghosh, S. C. A Visible Light-Mediated Regioselective Halogenation of Anilides and Quinolines by Using a Heterogeneous Cu-MnO Catalyst. *Eur. J. Org. Chem.* **2018**, *2018*, 4748-4753.

43. Ichikawa, M.; Yokomizo, A.; Itoh, M.; Haginoya, N.; Sugita, K.; Usui, H.; Terayama, K.; Kanda, A. Discovery of atrop fixed alkoxy-aminobenzhydrol derivatives: Novel, highly potent and orally efficacious squalene synthase inhibitors. *Bioorg. Med. Chem.* **2011**, *19*, 5207-5224.

44. Kianmehr, E.; Afaridoun, H. Nickel(II)- and Silver(I)-Catalyzed C-H Bond Halogenation of Anilides and Carbamates. *Synthesis* **2021**, *53*, 1513-1523.

45. Dahlgren, M. K.; Kauppi, A. M.; Olsson, I.-M.; Linusson, A.; Elofsson, M. Design, Synthesis, and Multivariate Quantitative Structure-Activity Relationship of Salicylanilides-Potent Inhibitors of Type III Secretion in Yersinia. *J. Med. Chem.* **2007**, *50*, 6177-6188.

46. Preparation of phenyl carboxamide and sulfonamide derivatives for use in inhibition of 11-beta-hydroxysteroid dehydrogenase Assignee: Sterix Limited World Intellectual Property Organization, WO2005042513 A1 2005-05-12.

47. Drug evolution: drug design at hot spots Assignee: National Research Council of Canada World Intellectual Property Organization, WO2002095393 A2 2002-11-28.

48. Liang, Y.; Lin, F.; Adeli, Y.; Jin, R.; Jiao, N. Efficient Electrocatalysis for the Preparation of (Hetero)aryl Chlorides and Vinyl Chloride with 1,2-Dichloroethane. *Angew. Chem. Int. Ed.* **2019**, *58*, 4566-4570.

49. Youse, M. S.; Abutaleb, N, S.; Nocentini, A.; Abdelsattar, A. S.; Ali, F.; Supuran, C. T.; Seleem, M. N.; Flaherty, D. P. Optimization of Ethoxzolamide Analogs with Improved Pharmacokinetic Properties for In Vivo Efficacy against *Neisseria gonorrhoeae*. *J. Med. Chem.* **2024**, *67*, 15537-15556.

50. *Gaussian 16*, Revision A.03, Frisch, M. J.; Trucks, G. W.; Schlegel, H. B.; Scuseria, G. E.; Robb, M. A.; Cheeseman, J. R.; Scalmani, G.; Barone, V.; Petersson, G. A.; Nakatsuji, H.; Li, X.; Caricato, M.; Marenich, A. V.; Bloino, J.; Janesko, B. G.; Gomperts, R.; Mennucci, B.; Hratchian, H. P.; Ortiz, J. V.; Izmaylov, A. F.; Sonnenberg, J. L.; Williams-Young, D.; Ding, F.; Lipparini, F.; Egidi, F.; Goings, J.; Peng, B.; Petrone, A.; Henderson, T.; Ranasinghe, D.; Zakrzewski, V. G.; Gao, J.; Rega, N.; Zheng, G.; Liang, W.; Hada, M.; Ehara, M.; Toyota, K.; Fukuda, R.; Hasegawa, J.; Ishida, M.; Nakajima, T.; Honda, Y.; Kitao, O.; Nakai, H.; Vreven, T.; Throssell, K.; Montgomery, J. A., Jr.; Peralta, J. E.; Ogliaro, F.; Bearpark, M. J.; Heyd, J. J.; Brothers, E. N.; Kudin, K. N.; Staroverov, V. N.; Keith, T. A.; Kobayashi, R.; Normand, J.; Raghavachari, K.; Rendell, A. P.; Burant, J. C.; Iyengar, S. S.; Tomasi, J.; Cossi, M.; Millam, J. M.; Klene, M.; Adamo, C.; Cammi, R.; Ochterski, J. W.; Martin, R. L.; Morokuma, K.; Farkas, O.; Foresman, J. B.; Fox, D. J. Gaussian, Inc., Wallingford CT, 2016.

51. Chai, J. D.; and Head-Gordon, M. *Phys. Chem. Chem. Phys.*, **2008**, *10*, 6615-6620.

52. Weigend, F; and Ahlrichs, R. *Phys. Chem. Chem. Phys.*, **2005**, *7*, 3297-3305.

53. Marenich, A. V.; Cramer, C. J.; Truhlar, D. G. *J. Phys. Chem. B*, **2009**, *113*, 6378-6396.

54. Gonzalez, C; Schlegel, H. B.; *J. Phys. Chem.*, **1990**, *94*, 5523-5527.

55. Grimme, S. *Chem. Eur. J.*, **2012**, *18*, 9955-9964.

56. For relevant reviews, see: a) Klumpp, D. A. in Arene Chemistry: Reaction Mechanisms and Methods for Aromatic Compounds, Wiley, 2016, Ed: Mortier, J., Chapter 1: Electrophilic Aromatic Substitution: Mechanism, pages: 1-31; b) Galabov, B.; Nalbantova, D.; Schleyer, P. v. R.; Schaefer, H. F. Electrophilic Aromatic Substitution: New Insights into an Old Class of Reactions. *Acc. Chem. Res.* **2016**, *49*, 1191-1199; c) Stamenković, N.; Ulrih, N. P.; Cerkovnik, J. An analysis of electrophilic aromatic substitution: a “complex approach”. *Phys. Chem. Chem. Phys.*, **2021**, *23*, 5051-5068.

57. For relevant computational mechanistic studies, see: a) Liljenberg, M.; Stenlid, J. H.; Brinck, T. Theoretical Investigation into Rate-Determining Factors in Electrophilic Aromatic Halogenation. *J. Phys. Chem. A* **2018**, *122*, 3270-3279; b) Qu, Z.-W.; Zhu, H.; Grimme, S. Mechanistic Insights for Iodane Mediated Aromatic Halogenation Reactions. *ChemCatChem* **2020**, *12*, 6186-6190; c) Galabov, B.; Koleva, G.; Simova, S.; Hadjieva, B.; Schaefer, H. F.; Schleyer, P. v. R. Arenium ions are not obligatory intermediates in electrophilic aromatic substitution. *PNAS* **2014**, *111*, 10067-10072.

58. For conceptual and methodological issues regarding barrierless reactions in homogeneous catalysis, see: Harvey, J. N.; Himo, F.; Maseras, F.; Perrin, L. *ACS Catal*. **2019**, *9*, 6803-6813.

59. For an insightful computational study along this direction, see: Nieves-Quinones, Y.; Singleton, D. A. Dynamics and the Regiochemistry of Nitration of Toluene. *J. Am. Chem. Soc.* **2016**, *138*, 15167-15176.

Cartesian Coordinates of the Calculated Structures

Cartesian coordinates of structures optimized at the ωB97X-D/def2-SVP/SMD(DMA) level of DFT are given below in standard XYZ format (units are in Å). First line indicates total number of atoms, second line is molecule name (as defined above, see also Table S3).

```
20
am-Cl+-p
C    0.832677    1.041904   -0.642042
C   -0.011471    0.004980    0.000930
C    0.106669   -0.080622    1.476763
C    0.826307    0.797897    2.203251
C    1.570448    1.834093    1.527773
C    1.549138    1.918549    0.083414
Cl   0.304573   -1.612260   -0.742389
N    2.309038    2.736353    2.164468
C    2.538756    2.914254    3.569082
O    2.038676    2.191914    4.386385
C    3.443714    4.067495    3.858196
H    0.865353    0.730071    3.286254
H    2.138819    2.700580   -0.399331
H    2.788497    3.412169    1.566597
H   -0.451183   -0.876061    1.976570
H    0.824666    1.097048   -1.732958
H   -1.067897    0.242998   -0.232685
H    4.417726    3.907330    3.370117
H    3.577760    4.161208    4.941144
H    3.011512    4.993733    3.449312

20
am-Cl+-o
C    0.847674    0.811595   -0.654026
C    0.092009   -0.078954    0.015646
C   -0.076228    0.067326    1.433186
C    0.516787    1.057479    2.178183
C    1.352968    2.000743    1.541912
C    1.432022    1.989501    0.036785
N    2.036312    2.954029    2.169708
C    2.050723    3.286706    3.560752
O    1.412744    2.663632    4.364663
Cl   3.081703    2.353715   -0.571893
C    2.921331    4.464641    3.856829
H    0.387922    1.094066    3.255469
H    0.793126    2.848422   -0.265850
H    2.636496    3.540597    1.586725
H   -0.693615   -0.666662    1.957244
H    1.002691    0.748339   -1.733015
H   -0.384916   -0.913387   -0.498796
H    3.950103    4.267692    3.518197
H    2.911258    4.661968    4.934118
H    2.550328    5.345596    3.310213

20
am-Cl+-m
O    2.233507    1.283651   -0.185338
N    1.176892   -0.726110    0.061789
C   -0.158559   -0.352383    0.073484
C   -0.588012    0.999249   -0.012760
C   -1.128096   -1.326056    0.205807
C   -1.940446    1.373641    0.011923
C   -2.922180    0.427170    0.132623
C    2.295042    0.076250   -0.067537
C    3.587372   -0.694555   -0.051404
H    0.177390    1.771082   -0.094992
H   -0.856771   -2.381312    0.296285
```

H	1.349801	-1.724061	0.147575
H	-2.196921	2.431917	-0.044990
H	-3.982257	0.688438	0.157831
H	3.683958	-1.257902	0.889096
H	4.425329	0.004358	-0.150656
H	3.608590	-1.417835	-0.880809
C	-2.545050	-0.984205	0.108221
H	-2.705857	-1.166894	-0.998170
Cl	-3.644060	-2.086446	0.967316

15

dma

N	-0.520231	-2.677108	-0.497334
C	-0.124061	-3.891285	-0.979083
O	1.044783	-4.131225	-1.254916
C	-1.195472	-4.945301	-1.168863
H	-1.715909	-5.170697	-0.225935
H	-1.950719	-4.625367	-1.902710
H	-0.708619	-5.856481	-1.535770
C	0.468715	-1.656857	-0.225286
H	0.508590	-1.427059	0.853540
H	1.451486	-2.014026	-0.550493
H	0.224568	-0.726498	-0.764157
C	-1.869954	-2.357905	-0.084200
H	-2.605142	-3.027519	-0.542612
H	-1.982328	-2.413738	1.013251
H	-2.115678	-1.330974	-0.396619

16

dma-Cl$^+$

N	-0.458377	-2.814932	0.016830
C	-0.127529	-3.928562	-1.083904
O	0.952808	-3.869743	-1.548077
C	-1.233653	-4.873295	-1.362900
H	-1.658566	-5.292315	-0.440669
H	-2.032005	-4.344793	-1.906081
H	-0.834684	-5.675659	-1.993615
C	0.465050	-1.656357	-0.129711
H	0.276833	-0.966379	0.699534
H	1.499118	-2.011866	-0.119833
H	0.229066	-1.176777	-1.088196
C	-1.881200	-2.371243	0.008824
H	-2.541694	-3.216299	0.224875
H	-1.998766	-1.590318	0.768198
H	-2.079995	-1.963563	-0.991031
Cl	-0.106594	-3.622341	1.558788

16

dma-Cl$^+$-O

N	-0.546206	-2.686817	-0.424531
C	-0.273273	-3.839877	-0.952454
O	0.975369	-3.941974	-1.407202
C	-1.230811	-4.959160	-1.069708
H	-2.161181	-4.751233	-0.536290
H	-1.452905	-5.136954	-2.133932
H	-0.777594	-5.869892	-0.652713
C	0.455973	-1.626290	-0.290212
H	0.328298	-1.166389	0.696992
H	1.468746	-2.028689	-0.375774
H	0.286009	-0.872242	-1.071564
C	-1.898427	-2.362351	0.048514
H	-2.644242	-2.616525	-0.713046
H	-2.107941	-2.895573	0.985531
H	-1.935013	-1.283913	0.230768
Cl	1.485229	-5.406513	-2.089767

31

dma-Cl^{+}···dma

N	-0.468430	-2.542875	-0.149147
C	-0.274449	-3.841058	-0.980972
O	0.231921	-3.717551	-2.043343
C	-0.762769	-5.087308	-0.334227
H	-0.544220	-5.120088	0.740420
H	-1.854072	-5.147200	-0.471776
H	-0.295697	-5.937386	-0.844514
C	-0.204181	-1.332895	-0.969178
H	-0.265317	-0.461341	-0.308407
H	0.788408	-1.407945	-1.422534
H	-0.977402	-1.275872	-1.745573
C	-1.808816	-2.470916	0.493545
H	-1.942373	-3.312141	1.180205
H	-1.873367	-1.523745	1.040767
H	-2.560232	-2.498686	-0.306949
Cl	0.805161	-2.648559	1.135373
N	1.731912	-4.109087	4.415552
C	2.354415	-2.981606	4.016947
O	2.499737	-2.689857	2.820569
C	2.903566	-2.058592	5.077309
H	2.083952	-1.574619	5.630942
H	3.534139	-2.592584	5.802443
H	3.494890	-1.281984	4.578974
C	1.322081	-5.108812	3.447145
H	0.235182	-5.070881	3.266200
H	1.850425	-4.952496	2.500374
H	1.573559	-6.108312	3.832099
C	1.486560	-4.448560	5.805520
H	1.469391	-3.557205	6.441200
H	0.503307	-4.933970	5.886719
H	2.249859	-5.146440	6.188501

34

am-dma

O	-2.458893	0.885567	1.024714
N	-1.318927	-0.707265	-0.162283
C	0.018923	-0.385859	0.099069
C	0.429379	0.724433	0.854044
C	0.997056	-1.244767	-0.435179
C	1.790120	0.956447	1.061578
C	2.348884	-0.999246	-0.220691
C	2.757530	0.104476	0.531654
C	-2.450065	-0.105268	0.312095
C	-3.731750	-0.797073	-0.096820
H	-0.321693	1.391090	1.271219
H	0.679490	-2.108971	-1.024054
H	-1.458276	-1.537542	-0.758450
H	2.093166	1.825385	1.651714
H	3.090582	-1.680152	-0.645853
H	3.819328	0.297069	0.701043
H	-4.134028	-1.327296	0.780755
H	-4.469255	-0.038365	-0.392576
H	-3.592478	-1.521177	-0.909934
N	-1.942469	-5.275960	-1.835566
C	-1.787371	-4.127448	-1.143543
O	-1.671390	-3.037910	-1.722006
C	-1.754220	-4.209621	0.363611
H	-0.863995	-4.759645	0.704940
H	-2.641598	-4.724584	0.759381
H	-1.716824	-3.194753	0.775738
C	-2.063393	-5.246399	-3.279987
H	-1.269449	-5.852714	-3.744322
H	-1.979147	-4.212802	-3.629917
H	-3.038717	-5.656068	-3.589452
C	-2.124364	-6.573542	-1.212579
H	-1.609844	-6.635569	-0.247194

H	-1.695029	-7.344581	-1.867684
H	-3.192787	-6.804646	-1.063158

19

am

O	2.293392	1.296254	0.002138
N	1.159506	-0.688725	-0.005769
C	-0.184054	-0.290208	-0.003047
C	-0.597145	1.051220	-0.005540
C	-1.158150	-1.303750	0.002165
C	-1.959136	1.355426	-0.002818
C	-2.511816	-0.984233	0.004664
C	-2.924173	0.349928	0.002244
C	2.296383	0.078530	-0.001285
C	3.569514	-0.736547	0.000810
H	0.151008	1.840254	-0.009306
H	-0.842628	-2.350861	0.004536
H	1.307052	-1.692987	-0.007641
H	-2.264710	2.404924	-0.004778
H	-3.251295	-1.788890	0.008721
H	-3.987200	0.600851	0.004288
H	3.614886	-1.375495	0.896090
H	4.431084	-0.059028	-0.007929
H	3.609383	-1.392762	-0.882043

50

dma-Cl^{+}···am-dma-*p*

C	2.727882	3.220350	3.430767
C	2.523064	2.914029	2.083722
C	1.659391	1.888832	1.698196
C	0.978258	1.147082	2.676952
C	1.175170	1.464819	4.034931
C	2.037743	2.488364	4.404207
N	0.102196	0.095007	2.395385
C	-0.326571	-0.357085	1.177275
C	-1.368322	-1.449962	1.261593
O	0.055555	0.082995	0.105691
O	-1.010279	-1.132709	4.677209
C	-2.149956	-0.811695	5.043336
C	-2.901948	0.293631	4.341484
N	-2.753980	-1.434835	6.077044
C	-4.102414	-1.145269	6.525558
C	-2.104893	-2.550815	6.736359
Cl	4.750991	0.998682	3.350298
N	5.793785	-0.451880	3.363932
C	5.662071	-1.138878	4.679591
C	7.249636	0.102319	3.115211
C	7.868027	0.778219	4.282251
C	5.365364	-1.342851	2.252321
O	7.696060	-0.088142	2.039769
H	1.507843	1.651721	0.648060
H	0.641464	0.889935	4.795675
H	-0.294726	-0.374508	3.224691
H	3.050131	3.480099	1.311456
H	2.179066	2.715612	5.463642
H	3.400893	4.030786	3.719761
H	-1.371228	-1.968555	2.229144
H	-1.199951	-2.169205	0.449122
H	-2.360513	-0.996489	1.107567
H	7.160374	1.415870	4.826417
H	8.245465	0.005702	4.971029
H	8.714267	1.371241	3.916954
H	4.322949	-1.625524	2.434496
H	5.468373	-0.815666	1.299724
H	6.011259	-2.229517	2.275141
H	5.872763	-0.436881	5.491679
H	4.639151	-1.522856	4.760303

H	6.384706	-1.966292	4.680889
H	-3.870457	-0.060312	3.959200
H	-3.094385	1.134691	5.024546
H	-2.298446	0.657886	3.502315
H	-2.720863	-3.459843	6.641069
H	-1.127794	-2.727461	6.276360
H	-1.968642	-2.335922	7.808294
H	-4.407361	-0.126310	6.265490
H	-4.828488	-1.854825	6.093779
H	-4.143209	-1.237146	7.620623

50

ts-*p*

C	0.035612	-0.050528	-0.131817
C	-0.006584	-0.027908	1.287857
C	1.079810	0.373159	2.035506
C	2.255649	0.812808	1.379040
C	2.285470	0.862153	-0.041412
C	1.197754	0.459857	-0.775657
N	3.395580	1.208538	2.030313
C	3.630221	1.304757	3.392923
C	4.997332	1.848915	3.720026
O	2.815062	1.005416	4.240844
O	5.486126	1.948273	0.380552
C	5.744228	3.143806	0.172121
C	4.801189	4.227467	0.635247
N	6.869035	3.511354	-0.473144
C	7.256404	4.889578	-0.707449
C	7.842559	2.513147	-0.871542
Cl	0.608265	-2.296998	-0.300365
N	1.179560	-4.254002	-0.474206
C	1.766415	-4.392799	-1.818911
C	-0.093237	-4.975692	-0.243625
C	-1.016784	-5.081306	-1.411932
C	2.166825	-4.464987	0.595854
O	-0.303111	-5.410930	0.849613
H	1.049207	0.356113	3.121320
H	3.189252	1.224293	-0.535479
H	4.186389	1.484645	1.414455
H	-0.913031	-0.363940	1.796589
H	1.229898	0.504555	-1.866384
H	-0.887522	-0.181487	-0.700480
H	5.778265	1.369994	3.112948
H	5.200676	1.696255	4.786393
H	5.020320	2.928845	3.502946
H	-1.143116	-4.116760	-1.921818
H	-0.612716	-5.803381	-2.137977
H	-1.985184	-5.443610	-1.049411
H	3.000741	-3.772292	0.435987
H	1.703740	-4.284094	1.570232
H	2.530202	-5.501802	0.544346
H	1.055278	-4.073114	-2.587205
H	2.662982	-3.764222	-1.869516
H	2.044822	-5.445220	-1.977344
H	5.307670	4.940217	1.302434
H	4.400314	4.792249	-0.219749
H	3.963370	3.768169	1.172498
H	8.807495	2.704243	-0.374604
H	7.482162	1.519096	-0.589509
H	8.000883	2.547204	-1.961101
H	6.387973	5.555944	-0.736422
H	7.953450	5.246429	0.069422
H	7.762978	4.957590	-1.680840

50

am-Cl$^+$-*p*-dma···dma

C	-0.838947	-0.159035	0.010515

C	-0.772071	-0.218345	1.491265
C	0.359307	0.026578	2.179256
C	1.585996	0.301855	1.464181
C	1.581829	0.330993	0.017133
C	0.459213	0.064526	-0.672235
N	2.752577	0.515146	2.056481
C	3.073679	0.506117	3.445350
C	4.523184	0.783268	3.696734
O	2.252916	0.293329	4.298556
O	4.841596	0.837630	0.406450
C	5.471242	1.891283	0.183189
C	4.912971	3.219188	0.624079
N	6.650991	1.847266	-0.448833
C	7.427117	3.013202	-0.828870
C	7.193302	0.562583	-0.858037
Cl	-1.665206	-1.632634	-0.632147
N	2.948442	-2.525607	0.618551
C	4.132006	-2.421311	1.450647
C	3.001040	-2.443240	-0.746287
C	4.300789	-1.964703	-1.351181
C	1.760365	-3.087220	1.227824
O	2.035853	-2.723876	-1.444602
H	0.371765	0.002106	3.264629
H	2.522476	0.534351	-0.496757
H	3.570917	0.687629	1.412614
H	-1.700159	-0.440007	2.023506
H	0.466439	0.051874	-1.763707
H	-1.502670	0.686530	-0.254786
H	5.143874	0.031950	3.186032
H	4.714928	0.756631	4.774951
H	4.793814	1.771152	3.293587
H	4.580891	-0.976165	-0.957546
H	5.124338	-2.659878	-1.125607
H	4.170742	-1.906510	-2.438345
H	1.630239	-2.667806	2.236041
H	0.883151	-2.840548	0.617349
H	1.823125	-4.187010	1.316289
H	4.774498	-1.591609	1.129166
H	3.824246	-2.224841	2.487085
H	4.727070	-3.351855	1.448614
H	5.614523	3.751395	1.282387
H	4.705585	3.862330	-0.244213
H	3.972541	3.050273	1.161973
H	8.289185	0.629071	-0.876960
H	6.893407	-0.219324	-0.150748
H	6.840014	0.283518	-1.864297
H	6.893619	3.941572	-0.605656
H	8.389618	3.022724	-0.293899
H	7.630155	2.985457	-1.910419

50

dma-Cl^{+}···am-dma-*o1*

C	2.503928	-0.998667	-0.081226
C	2.871994	0.136970	0.639579
C	1.875660	1.002460	1.091809
C	0.527170	0.756176	0.835356
C	0.153854	-0.384891	0.106739
C	1.160857	-1.260213	-0.350268
N	-1.163320	-0.712778	-0.222011
C	-2.315207	-0.049822	0.102786
O	-2.355678	0.989237	0.740346
C	-3.574354	-0.739121	-0.372713
O	-1.402366	-3.091099	-1.704658
C	-1.746613	-4.137174	-1.135454
N	-1.902275	-5.287398	-1.823729
C	-1.724373	-5.306954	-3.262235
C	-2.005326	-4.154410	0.352127

C	-2.349306	-6.536172	-1.238238
Cl	1.193899	0.374402	-2.774251
N	1.107517	1.409109	-4.233118
C	1.140320	0.554305	-5.452836
C	-0.149899	2.201082	-4.163362
C	2.369860	2.346929	-4.121305
C	3.666354	1.689471	-4.418589
O	2.153261	3.468952	-3.827043
H	-0.243271	1.437745	1.187490
H	0.872850	-2.157655	-0.904059
H	-1.270116	-1.572827	-0.783435
H	2.148913	1.896709	1.657994
H	3.265468	-1.692590	-0.445239
H	3.923725	0.345136	0.847114
H	-4.040738	-1.243985	0.488019
H	-4.281010	0.018922	-0.736535
H	-3.389020	-1.485579	-1.155936
H	-1.317140	-4.841367	0.866807
H	-3.033695	-4.475381	0.574129
H	-1.853257	-3.144958	0.750795
H	-0.923875	-6.011480	-3.539399
H	-1.459614	-4.303920	-3.610609
H	-2.655377	-5.629353	-3.756087
H	-2.199856	-6.552978	-0.154233
H	-1.770572	-7.365797	-1.670805
H	-3.416893	-6.713826	-1.451512
H	-0.185595	2.841695	-5.053038
H	-0.989871	1.498058	-4.167101
H	-0.149010	2.806773	-3.252557
H	1.172641	1.230895	-6.317241
H	2.021674	-0.093764	-5.433126
H	0.224376	-0.046857	-5.472405
H	4.463737	2.320179	-4.009028
H	3.733068	0.674100	-4.008955
H	3.784253	1.633792	-5.512601

50

ts-*o1*

C	-0.113253	-0.022320	-0.079123
C	-0.170916	-0.085322	1.294035
C	0.925967	0.384570	2.044011
C	2.061442	0.914435	1.451450
C	2.139167	0.990101	0.047028
C	1.053748	0.463205	-0.730095
N	3.202157	1.494619	-0.654708
C	4.405340	1.976000	-0.165677
O	4.679813	2.027195	1.015585
C	5.337239	2.472389	-1.241381
O	2.878975	1.299746	-3.385127
C	2.704330	2.265731	-4.144856
N	2.505233	2.086507	-5.464514
C	2.399143	0.748299	-6.014005
C	2.717001	3.676318	-3.610185
C	2.285005	3.174830	-6.399510
Cl	2.085334	-1.532544	-1.170745
N	3.132712	-3.188013	-1.752586
C	2.467323	-3.705673	-2.961558
C	4.491873	-2.695029	-2.025799
C	3.047020	-4.044051	-0.548200
C	1.783004	-4.821399	-0.378106
O	3.973005	-4.048512	0.207818
H	2.888300	1.279617	2.054087
H	1.013426	0.711392	-1.793735
H	3.107425	1.460021	-1.689582
H	0.883397	0.335951	3.135047
H	-0.947570	-0.365626	-0.694451
H	-1.051914	-0.483197	1.799884

H	5.157166	3.549338	-1.389109
H	6.373661	2.344018	-0.904851
H	5.183276	1.964277	-2.202388
H	1.832975	4.243465	-3.934400
H	3.611544	4.214076	-3.960451
H	2.735632	3.641233	-2.514659
H	1.434635	0.629842	-6.532909
H	2.466039	0.014442	-5.204604
H	3.208476	0.563773	-6.738381
H	2.805229	4.087268	-6.087139
H	1.210775	3.396363	-6.515949
H	2.684765	2.884069	-7.380906
H	5.115183	-3.535281	-2.364894
H	4.427860	-1.937204	-2.814858
H	4.919260	-2.259688	-1.117714
H	2.970166	-4.632997	-3.273847
H	1.407525	-3.899003	-2.768050
H	2.560924	-2.953358	-3.753608
H	1.764538	-5.225307	0.640291
H	0.893192	-4.201809	-0.553581
H	1.766290	-5.656751	-1.095069

50

am-Cl$^+$-*o1*-dma···dma

C	0.000000	0.000000	0.000000
C	0.000000	0.000000	1.344176
C	1.244827	0.152373	2.056100
C	2.453974	0.356876	1.447437
C	2.506754	0.450799	0.033746
C	1.261385	0.153358	-0.772785
N	3.566411	0.786766	-0.681227
C	4.884007	1.084356	-0.218754
O	5.216639	0.898457	0.921358
C	5.759684	1.648788	-1.293071
O	3.180777	1.050110	-3.303418
C	3.120611	2.137133	-3.911275
N	3.352571	2.183480	-5.229796
C	3.723820	0.966624	-5.931657
C	2.786693	3.407469	-3.173300
C	3.213703	3.372683	-6.050125
Cl	1.560632	-1.431973	-1.623382
N	4.125384	-2.850979	-3.105022
C	3.507982	-2.640415	-4.396923
C	4.996378	-1.788688	-2.649645
C	4.050658	-3.998987	-2.374864
C	3.141063	-5.097841	-2.885408
O	4.685511	-4.140262	-1.336662
H	3.360345	0.481669	2.031813
H	1.147799	0.904285	-1.569599
H	3.415350	0.895810	-1.725497
H	1.219702	0.108809	3.147923
H	-0.913238	-0.139781	-0.581949
H	-0.923988	-0.125834	1.909215
H	5.314176	2.574989	-1.687742
H	6.749689	1.862340	-0.875344
H	5.853510	0.939700	-2.128339
H	1.803212	3.788965	-3.487466
H	3.532102	4.193423	-3.359391
H	2.748420	3.197975	-2.097689
H	2.833757	0.435456	-6.307585
H	4.275961	0.296994	-5.262523
H	4.362050	1.228928	-6.786077
H	2.740994	4.190459	-5.498240
H	2.583624	3.142947	-6.922624
H	4.198009	3.711021	-6.410959
H	6.006527	-1.870463	-3.090655
H	4.563688	-0.824143	-2.948097

H	5.093367	-1.830014	-1.557781
H	4.262945	-2.635804	-5.203026
H	2.770172	-3.416334	-4.625388
H	2.993190	-1.667004	-4.406338
H	3.179140	-5.925345	-2.167254
H	2.100156	-4.753392	-2.979100
H	3.465405	-5.465786	-3.870555

50

dma-Cl⁺···am-dma-*o2*

C	2.882888	0.327101	0.558279
C	1.902997	1.095392	1.178840
C	0.543537	0.802873	1.019683
C	0.153392	-0.293990	0.229164
C	1.148752	-1.071620	-0.392603
C	2.492677	-0.760377	-0.230411
N	-1.174717	-0.659667	-0.000383
C	-2.314650	-0.078251	0.483578
C	-3.586861	-0.784092	0.072360
O	-2.332447	0.904838	1.205647
O	-1.479050	-2.904650	-1.670505
C	-1.751254	-4.012079	-1.184871
C	-1.900562	-4.181119	0.308189
N	-1.925739	-5.095100	-1.971322
C	-2.305973	-6.407055	-1.485041
C	-1.856929	-4.966939	-3.413711
H	-0.218918	1.401641	1.512826
H	0.848025	-1.920443	-1.011512
H	-1.301266	-1.473309	-0.623488
H	2.187806	1.949470	1.798431
H	3.247282	-1.376523	-0.725702
H	3.940586	0.567530	0.685323
H	-3.943336	-1.383238	0.925158
H	-4.356105	-0.032689	-0.150990
H	-3.451360	-1.450684	-0.789133
H	-1.175862	-4.909035	0.701775
H	-2.909687	-4.533554	0.568324
H	-1.723917	-3.215055	0.794530
H	-1.087325	-5.642229	-3.820205
H	-1.608122	-3.934215	-3.676606
H	-2.825577	-5.233587	-3.867317
H	-2.084773	-6.526733	-0.419843
H	-1.740552	-7.174350	-2.034559
H	-3.381543	-6.592059	-1.645779
Cl	0.459513	2.285858	-1.522087
N	0.535676	3.086382	-3.120419
C	-0.719122	3.857885	-3.324911
C	1.780519	4.049881	-3.021846
C	0.709443	2.046487	-4.174132
H	-0.653024	4.339988	-4.307805
H	-1.555718	3.151221	-3.304873
H	-0.819977	4.608072	-2.535077
H	0.865525	2.574983	-5.123995
H	1.567874	1.411380	-3.934841
H	-0.207008	1.447643	-4.216173
O	1.535624	5.202502	-2.959178
C	3.100695	3.371918	-3.039165
H	3.844831	4.078165	-2.653297
H	3.111273	2.446011	-2.450794
H	3.351036	3.128310	-4.083933

50

ts-*o2*

C	-0.106479	0.106223	0.224465
C	-0.030917	0.094946	1.595412
C	1.200076	0.351500	2.268213
C	2.347403	0.730393	1.500321

C	2.249462	0.726021	0.090740
C	1.051607	0.419107	-0.522495
N	3.557857	1.048138	2.053332
C	3.882961	1.200312	3.392297
C	5.318143	1.603433	3.609422
O	3.090135	1.043412	4.297951
O	5.561788	1.521236	0.225104
C	5.883213	2.682677	-0.071182
C	5.075341	3.856400	0.426818
N	6.957363	2.931224	-0.845941
C	7.416304	4.261389	-1.197946
C	7.802376	1.842151	-1.296141
H	1.207458	0.540400	3.340353
H	3.131653	0.978573	-0.500558
H	4.326787	1.217865	1.372159
H	-0.908504	-0.138436	2.202156
H	1.000078	0.426134	-1.613952
H	-1.044127	-0.116717	-0.287031
H	5.474120	2.630109	3.242318
H	5.545225	1.566006	4.681073
H	6.002474	0.944180	3.056508
H	4.626146	4.407990	-0.412639
H	5.699192	4.559335	0.997868
H	4.269030	3.489380	1.072193
H	7.830091	1.809635	-2.396970
H	7.409368	0.894087	-0.916420
H	8.831200	1.983190	-0.927394
H	8.248571	4.579580	-0.547815
H	6.611420	5.000223	-1.128523
H	7.775958	4.253753	-2.237114
Cl	1.926882	-1.810406	2.531246
N	2.668672	-3.685286	2.879514
C	2.342344	-3.997369	4.279942
C	1.907314	-4.498688	1.904813
C	4.120925	-3.589023	2.648188
H	2.732297	-4.996740	4.522511
H	2.823000	-3.247825	4.918704
H	1.258092	-3.979455	4.423166
H	4.565885	-4.588532	2.762854
H	4.330248	-3.196832	1.647956
H	4.542877	-2.910329	3.398656
O	0.965774	-5.118720	2.302621
C	2.380867	-4.453707	0.488727
H	1.608951	-4.906048	-0.143887
H	2.585728	-3.426850	0.156733
H	3.309086	-5.038035	0.392681

50

am-Cl$^+$-*o2*-dma···dma

C	0.030933	0.027082	-0.006532
C	0.014119	0.035108	1.337686
C	1.243710	0.286379	2.137426
C	2.479883	0.626442	1.346308
C	2.447629	0.529972	-0.069840
C	1.271506	0.252891	-0.706488
N	3.613154	0.975479	1.930609
C	3.838992	1.349304	3.288005
C	5.266283	1.709020	3.553972
O	2.945752	1.401311	4.091723
O	5.744306	0.894650	0.362842
C	6.370297	1.866379	-0.104363
C	5.789311	3.254651	-0.024491
N	7.566785	1.696932	-0.685631
C	8.364944	2.777020	-1.236496
C	8.185837	0.384841	-0.731575
H	1.066935	1.061858	2.897471
H	3.366287	0.711670	-0.628917

H	4.459852	1.035610	1.290469
H	-0.898539	-0.155157	1.906372
H	1.267017	0.205363	-1.798148
H	-0.876896	-0.160766	-0.580601
H	5.508194	2.641263	3.018520
H	5.408032	1.860552	4.629726
H	5.944125	0.927062	3.183717
H	5.608577	3.661642	-1.030500
H	6.467657	3.941420	0.502078
H	4.832627	3.216676	0.509776
H	8.295557	0.051521	-1.775680
H	7.569687	-0.336036	-0.185827
H	9.186081	0.429644	-0.273497
H	7.758184	3.662859	-1.448231
H	8.816805	2.442646	-2.181526
H	9.176087	3.056721	-0.544507
Cl	1.610010	-1.238208	3.055407
N	4.518193	-3.155171	2.601362
C	4.366497	-3.916666	3.820816
C	3.986104	-3.627501	1.437418
C	5.297326	-1.943887	2.726221
H	5.350285	-4.221932	4.216656
H	3.860187	-3.314876	4.594253
H	3.770414	-4.811573	3.613584
H	6.363251	-2.170958	2.905309
H	5.229121	-1.316191	1.832054
H	4.932715	-1.357334	3.585006
O	3.356159	-4.676630	1.389270
C	4.198787	-2.796463	0.189789
H	3.752189	-3.335296	-0.653938
H	3.708404	-1.816568	0.281179
H	5.265241	-2.622756	-0.015879

2

Cl_2

Cl	2.027465	2.380952	0.000000
Cl	-0.001253	2.380952	0.000000

51

dma···Cl_2···am-dma (*p*)

C	0.000000	0.000000	0.000000
C	0.000000	0.000000	1.398294
C	0.827934	0.896825	2.074048
C	1.651050	1.784578	1.380727
C	1.650444	1.783053	-0.023324
C	0.813231	0.879495	-0.704180
N	2.436706	2.632794	-0.809553
C	3.253033	3.653998	-0.410649
O	3.470862	3.949960	0.753000
C	3.858624	4.436973	-1.554429
Cl	2.340821	-2.056516	1.436541
Cl	3.826663	-3.489698	1.466811
N	5.845261	-5.311720	1.403983
C	5.409420	-6.055592	2.485341
C	5.818208	-5.575813	3.858280
C	5.626552	-5.870084	0.080567
C	6.973986	-4.401081	1.512062
O	4.709007	-7.041381	2.334747
H	2.295216	2.481257	1.911827
H	0.813444	0.877330	-1.797012
H	2.345955	2.491162	-1.827517
H	0.840551	0.907872	3.166999
H	-0.640228	-0.695003	-0.548858
H	-0.642700	-0.687553	1.952646
H	3.794485	3.914085	-2.517451
H	4.907300	4.663194	-1.318434
H	3.322048	5.395161	-1.640426

H	6.425301	-6.583084	-0.191360
H	4.666841	-6.397505	0.055820
H	5.615600	-5.059259	-0.660824
H	7.935881	-4.943325	1.493738
H	6.954857	-3.705747	0.661737
H	6.922588	-3.805375	2.430618
H	5.546776	-4.522481	4.020548
H	5.309110	-6.199563	4.602206
H	6.906031	-5.668894	3.998665
O	2.102058	2.288558	-3.593150
C	1.256085	2.978133	-4.179743
C	0.399149	3.959958	-3.416902
H	0.723704	3.977341	-2.370294
H	0.480709	4.974550	-3.833004
H	-0.660318	3.664064	-3.450199
N	1.069776	2.872935	-5.512634
C	0.125183	3.672815	-6.268230
C	1.913986	1.996375	-6.300286
H	2.636122	1.500208	-5.644487
H	1.304447	1.234209	-6.811685
H	2.456137	2.576475	-7.064642
H	-0.317562	3.051408	-7.060334
H	-0.691426	4.039917	-5.637838
H	0.621902	4.534992	-6.744739

51

dma···Cl$_2$···am-dma (*o1*)

C	-2.534406	-0.658565	-1.478299
C	-2.913264	0.684679	-1.469001
C	-1.928407	1.663763	-1.343998
C	-0.580092	1.325065	-1.226032
C	-0.197984	-0.025951	-1.233985
C	-1.193269	-1.014097	-1.363548
N	1.122084	-0.471142	-1.109814
C	2.267111	0.268480	-1.012715
O	2.302034	1.488227	-0.995116
C	3.531585	-0.559933	-0.959331
Cl	-1.183562	-1.123467	1.718446
Cl	-1.037454	-1.315844	3.769464
N	-0.694029	-1.601632	6.448292
C	-0.495932	-3.039347	6.519755
C	0.511846	-0.802465	6.599261
C	-1.935337	-1.115283	6.817296
C	-2.076451	0.383475	6.944166
O	-2.876530	-1.864816	7.010338
H	0.183184	2.092913	-1.125476
H	-0.897341	-2.066066	-1.376941
H	1.235509	-1.496601	-1.100019
H	-2.209372	2.720185	-1.334556
H	-3.288778	-1.443300	-1.574990
H	-3.965361	0.964301	-1.557712
H	3.346657	-1.618092	-0.733679
H	4.208362	-0.129716	-0.208515
H	4.033387	-0.491572	-1.937406
H	-0.235924	-3.358273	7.544916
H	-1.413842	-3.555171	6.217760
H	0.322532	-3.328667	5.846343
H	0.854107	-0.778235	7.648910
H	1.311175	-1.242031	5.987068
H	0.357065	0.226546	6.254732
H	-1.753544	0.902752	6.029992
H	-3.131446	0.610836	7.136395
H	-1.470646	0.765051	7.780352
O	1.405415	-3.278510	-1.182764
C	1.746150	-3.825919	-2.240676
C	2.003184	-3.007962	-3.483813
H	1.865217	-1.947067	-3.245869

H	3.026178	-3.161290	-3.857727
H	1.304221	-3.279742	-4.288863
N	1.900494	-5.164949	-2.316637
C	2.344298	-5.867371	-3.504869
C	1.736695	-5.985992	-1.133182
H	1.473263	-5.349994	-0.282365
H	0.940273	-6.730893	-1.290370
H	2.673288	-6.522331	-0.909519
H	1.802313	-6.821294	-3.581751
H	2.140874	-5.294375	-4.415288
H	3.424184	-6.088298	-3.458126

51

dma···Cl$_2$···am-dma (*o2*)

C	0.641303	1.017502	-0.692663
C	-0.018617	-0.033913	-0.050201
C	-0.037776	-0.068895	1.341549
C	0.589343	0.923826	2.099386
C	1.252404	1.980646	1.452369
C	1.269276	2.013370	0.045541
N	1.921170	3.011456	2.120249
C	2.004122	3.243837	3.464385
O	1.487557	2.543472	4.319842
C	2.779271	4.491500	3.826619
Cl	3.404713	-0.362244	2.107674
Cl	5.281805	-1.213663	2.231016
N	7.817975	-2.189715	2.383715
C	8.300751	-1.812751	1.066203
C	8.304095	-1.366635	3.479670
C	7.462997	-3.512661	2.573780
C	7.160760	-3.946235	3.989163
O	7.378569	-4.286514	1.636160
H	0.566937	0.896531	3.186179
H	1.788889	2.831954	-0.458663
H	2.409238	3.682121	1.506711
H	-0.547484	-0.884757	1.860556
H	0.668994	1.062802	-1.784298
H	-0.510640	-0.816574	-0.631889
H	3.392049	4.875168	3.000716
H	3.416507	4.274653	4.694798
H	2.063414	5.272907	4.126365
H	9.376897	-2.035559	0.954042
H	7.749581	-2.365783	0.297940
H	8.150448	-0.734576	0.917099
H	9.360754	-1.585999	3.714174
H	8.227943	-0.308624	3.193635
H	7.704557	-1.510894	4.385835
H	6.401567	-3.304379	4.459351
H	6.793383	-4.978571	3.957547
H	8.067054	-3.908598	4.612869
O	3.184534	4.862898	0.403770
C	2.671380	5.974537	0.214222
C	1.379313	6.353955	0.897610
H	0.985164	5.476524	1.423145
H	1.547578	7.157441	1.630938
H	0.627248	6.706134	0.177149
N	3.249529	6.883506	-0.599074
C	2.687008	8.189990	-0.882464
C	4.448954	6.543190	-1.338652
H	4.740826	5.514520	-1.105407
H	4.265799	6.632142	-2.421810
H	5.272594	7.223685	-1.069499
H	2.096012	8.180585	-1.814018
H	2.051199	8.542990	-0.063391
H	3.506478	8.913089	-1.003215

51

dma(Cl⁻)···am-Cl⁺-*p*-dma

C	-0.240139	0.181899	0.208324
C	-0.340548	0.281519	1.682389
C	0.143901	1.558084	2.255286
C	0.593300	2.582193	1.500786
C	0.636844	2.441081	0.064110
C	0.204568	1.201388	-0.548482
N	1.054427	3.390933	-0.763864
C	1.502511	4.709556	-0.464781
O	1.644480	5.106272	0.662075
C	1.753021	5.521180	-1.697847
Cl	0.559329	-1.106683	2.453263
Cl	2.008973	-3.477048	3.773113
N	5.126898	-6.519093	3.952016
C	4.454650	-6.882123	5.083165
C	3.877589	-5.758712	5.913697
C	5.812364	-7.525984	3.171858
C	5.338556	-5.135302	3.566980
O	4.326235	-8.054409	5.419283
H	0.931227	3.508994	1.954511
H	0.260931	1.118255	-1.634924
H	1.023279	3.152919	-1.793863
H	0.111898	1.656854	3.343222
H	-0.559242	-0.753931	-0.256674
H	-1.397208	0.144631	1.976284
H	2.454928	5.001336	-2.366245
H	2.156784	6.498713	-1.412072
H	0.808067	5.657260	-2.247470
H	6.904815	-7.368481	3.194330
H	5.587698	-8.513524	3.589260
H	5.484175	-7.497171	2.119773
H	5.631292	-5.109398	2.507770
H	4.417658	-4.538270	3.665798
H	6.148696	-4.660241	4.149370
H	3.157996	-5.149301	5.336745
H	3.379834	-6.200214	6.785887
H	4.672942	-5.079606	6.260274
O	0.972481	2.769831	-3.374938
C	-0.006544	3.041860	-4.094554
C	-1.277979	3.584313	-3.492666
H	-1.130011	3.759651	-2.420794
H	-1.577968	4.528047	-3.970339
H	-2.102252	2.865581	-3.616574
N	0.040247	2.855115	-5.423799
C	-1.054891	3.178249	-6.320931
C	1.264133	2.405391	-6.060710
H	2.044919	2.277239	-5.304982
H	1.095919	1.446435	-6.575497
H	1.595567	3.147079	-6.804553
H	-1.036356	2.478050	-7.167517
H	-2.027070	3.076621	-5.826367
H	-0.958883	4.202965	-6.716677

51

dma(Cl⁻)···am-Cl⁺-*o1*-dma

C	-0.071373	0.044046	0.018373
C	-0.095589	0.060310	1.368011
C	0.962162	0.717857	2.080262
C	1.994623	1.383715	1.467280
C	2.028393	1.446453	0.053403
C	1.039393	0.654316	-0.737374
N	2.901178	2.145203	-0.670510
C	3.993827	2.925287	-0.219786
O	4.298520	3.005050	0.943286
C	4.699543	3.629306	-1.339052
Cl	2.015277	-0.779253	-1.451703
Cl	3.470123	-2.877390	-2.641315

N	5.497546	-5.600623	-4.091570
C	4.683191	-5.999556	-5.218975
C	6.267696	-4.382473	-4.264883
C	5.207048	-6.118547	-2.861506
C	5.897269	-5.484374	-1.675274
O	4.419364	-7.045725	-2.714793
H	2.759398	1.888302	2.049847
H	0.694844	1.210985	-1.620012
H	2.746647	2.141380	-1.714948
H	0.940283	0.696285	3.172939
H	-0.845427	-0.458982	-0.565249
H	-0.899078	-0.421247	1.926204
H	5.012846	2.909770	-2.109580
H	5.572738	4.158152	-0.941040
H	4.015703	4.350268	-1.813791
H	5.300442	-6.086030	-6.126226
H	4.217298	-6.967334	-5.003383
H	3.883869	-5.259770	-5.408655
H	6.573809	-4.304636	-5.317710
H	5.667973	-3.496077	-3.992799
H	7.183929	-4.396003	-3.659724
H	5.504266	-4.461423	-1.556160
H	5.655165	-6.073160	-0.782096
H	6.989363	-5.433934	-1.792387
O	2.412314	2.210763	-3.309529
C	1.778117	3.139964	-3.838773
C	1.150180	4.221274	-2.995042
H	1.398351	4.048892	-1.941295
H	1.511973	5.217682	-3.287393
H	0.055005	4.213891	-3.100651
N	1.631125	3.201191	-5.173955
C	0.945910	4.275169	-5.868325
C	2.266209	2.213854	-6.026548
H	2.785637	1.475554	-5.408186
H	1.509934	1.704085	-6.643679
H	2.991089	2.702209	-6.697522
H	0.389066	3.853465	-6.717428
H	0.228578	4.786477	-5.218327
H	1.663612	5.015382	-6.259535

51

dma(Cl$^-$)···am-Cl$^+$-*o2*-dma

C	-0.045247	0.064632	0.092367
C	-0.038801	0.084501	1.527070
C	1.041799	0.571017	2.175538
C	2.233265	1.049932	1.452873
C	2.108234	1.148096	-0.022093
C	0.982842	0.565543	-0.660741
N	3.043753	1.715183	-0.785206
C	4.159792	2.488384	-0.386508
O	4.401274	2.733767	0.768586
C	4.965584	2.980754	-1.550387
Cl	3.547016	-0.295367	1.706998
Cl	5.491512	-2.207780	2.096721
N	8.297179	-4.565988	2.395595
C	7.802915	-5.678035	1.613196
C	8.807377	-3.437814	1.638837
C	7.920476	-4.467694	3.704690
C	8.233830	-3.165909	4.407505
O	7.347599	-5.380349	4.288113
H	2.693014	1.940138	1.895132
H	0.938683	0.560306	-1.750693
H	2.893247	1.634510	-1.826015
H	1.094889	0.586594	3.266335
H	-0.911725	-0.359151	-0.421291
H	-0.901845	-0.298311	2.072897
H	5.268873	2.139548	-2.190760

H	5.850390	3.510345	-1.180079
H	4.356289	3.664715	-2.161422
H	8.578936	-6.032742	0.917652
H	7.518095	-6.494963	2.285068
H	6.915318	-5.381491	1.024917
H	9.294654	-3.813769	0.727898
H	7.990513	-2.750146	1.357951
H	9.563822	-2.881526	2.207573
H	7.621150	-2.369979	3.953890
H	7.964158	-3.273444	5.465197
H	9.293980	-2.884151	4.330340
O	2.620732	1.471550	-3.434464
C	2.104067	2.367812	-4.125064
C	1.595721	3.636061	-3.486876
H	1.679912	3.553176	-2.397028
H	2.183924	4.504378	-3.820361
H	0.543704	3.821252	-3.747711
N	1.985617	2.233643	-5.457444
C	1.351430	3.221764	-6.311676
C	2.387121	1.001366	-6.110094
H	2.779145	0.302883	-5.364619
H	1.522746	0.543751	-6.617307
H	3.165105	1.204481	-6.862561
H	0.275167	3.014597	-6.434194
H	1.476273	4.237012	-5.919458
H	1.824094	3.186918	-7.302897

35

am-Cl⁺-*p*-dma

C	0.234432	1.187606	-0.551764
C	-0.212539	0.170503	0.203561
C	-0.353533	0.294084	1.675936
C	0.166248	1.558185	2.254675
C	0.618165	2.576458	1.497489
C	0.659208	2.433902	0.058353
Cl	0.410033	-1.124850	2.499890
N	1.069322	3.381693	-0.769774
C	1.513151	4.706444	-0.472312
C	1.739842	5.521397	-1.706519
O	1.668424	5.097216	0.653902
O	0.970546	2.758994	-3.370626
C	-0.008989	3.038364	-4.087999
N	0.035564	2.856336	-5.417292
C	1.256451	2.403088	-6.057776
C	-1.276647	3.584718	-3.482217
C	-1.059193	3.188104	-6.312021
H	0.962541	3.501537	1.950009
H	0.301289	1.098941	-1.637157
H	1.032483	3.143495	-1.802165
H	0.136465	1.656862	3.342391
H	-0.523853	-0.769658	-0.257593
H	-1.431137	0.233384	1.920020
H	2.441261	5.009581	-2.381748
H	2.134771	6.503648	-1.424765
H	0.787443	5.645109	-2.246185
H	-1.128478	3.751625	-2.409059
H	-1.569922	4.533723	-3.953592
H	-2.105720	2.872546	-3.611577
H	2.038862	2.271063	-5.304456
H	1.083263	1.445626	-6.573640
H	1.588231	3.144862	-6.801264
H	-1.044515	2.491344	-7.161451
H	-2.031273	3.088937	-5.816820
H	-0.958532	4.213995	-6.703337

35

am-Cl⁺-*o1*-dma

C	1.006964	0.657171	-0.746719
C	-0.075960	0.007797	0.040340
C	-0.090635	0.054064	1.383488
C	0.972438	0.729889	2.083631
C	2.001384	1.386099	1.462375
C	2.037628	1.433779	0.046105
N	2.905221	2.116741	-0.682267
C	4.000583	2.912615	-0.231707
C	4.691367	3.625633	-1.351305
Cl	1.876514	-0.649381	-1.655463
O	4.305582	2.985596	0.929155
O	2.444280	2.217003	-3.300064
C	1.795878	3.137758	-3.830235
N	1.655437	3.198439	-5.164692
C	2.312078	2.224671	-6.016947
C	1.146268	4.206048	-2.987403
C	0.952641	4.260550	-5.860585
H	2.772199	1.889225	2.037831
H	0.568981	1.321936	-1.509404
H	2.748678	2.120218	-1.734049
H	0.954084	0.720330	3.176464
H	-0.845802	-0.513707	-0.532221
H	-0.885572	-0.424087	1.956460
H	5.003508	2.912138	-2.127774
H	5.563118	4.159054	-0.956452
H	3.997929	4.343814	-1.816303
H	1.395579	4.041176	-1.932691
H	1.488930	5.208989	-3.280253
H	0.051673	4.177070	-3.095296
H	2.853646	1.502156	-5.399125
H	1.566491	1.693673	-6.629138
H	3.020432	2.730480	-6.692323
H	0.413088	3.829716	-6.716080
H	0.218561	4.753001	-5.214791
H	1.657821	5.017401	-6.242190

35

am-Cl^{+}-*o2*-dma

C	0.957805	0.582396	-0.669605
C	-0.070318	0.115392	0.098420
C	-0.031155	0.097513	1.540297
C	1.078505	0.504353	2.180811
C	2.274030	1.001439	1.446501
C	2.124615	1.105000	-0.050358
Cl	3.632867	-0.157960	1.775604
N	3.053283	1.657239	-0.812921
C	4.184198	2.430776	-0.414831
C	4.977059	2.930981	-1.579710
O	4.419821	2.665992	0.740778
O	2.602142	1.465403	-3.429009
C	2.092404	2.373734	-4.112969
N	1.977476	2.251110	-5.444617
C	2.378073	1.023950	-6.108253
C	1.590900	3.637640	-3.462724
C	1.352843	3.250499	-6.293361
H	2.604172	1.971964	1.847237
H	0.890254	0.597561	-1.757999
H	2.879182	1.605953	-1.861350
H	1.160550	0.478506	3.269480
H	-0.967668	-0.257803	-0.401150
H	-0.900131	-0.263237	2.091484
H	5.251372	2.099119	-2.244585
H	5.876689	3.438442	-1.214464
H	4.366657	3.639488	-2.161464
H	1.677660	3.547142	-2.373740
H	2.180598	4.506710	-3.791304
H	0.538634	3.827469	-3.719083

H	2.759375	0.314011	-5.368180
H	1.515246	0.578782	-6.628570
H	3.163677	1.232592	-6.851079
H	0.278934	3.042067	-6.431575
H	1.470545	4.260104	-5.885156
H	1.838291	3.229698	-7.278715

50

dma···am-Cl⁺-*p*-dma

C	1.426074	-0.098987	1.284986
C	1.608215	-0.284858	-0.135711
C	0.571989	-0.646786	-0.920166
C	-0.791641	-0.746380	-0.376294
C	-0.895567	-0.730778	1.090755
C	0.141378	-0.366955	1.879134
Cl	-1.771545	-2.046151	-1.137568
N	2.492739	0.310108	1.970466
C	2.604020	0.610818	3.352996
O	1.703875	0.433204	4.133199
C	3.945418	1.174983	3.710046
O	4.709132	0.751987	0.506612
C	4.932959	1.841942	-0.050836
N	6.108601	2.078529	-0.657201
C	7.171331	1.091734	-0.611324
C	3.885699	2.927155	-0.066257
C	6.446830	3.329059	-1.312028
O	-2.017680	1.821631	-0.992535
C	-3.091617	2.118569	-0.454178
N	-3.463844	3.406957	-0.294355
C	-4.724929	3.815998	0.295150
C	-4.022868	1.044482	0.055879
C	-2.644096	4.474755	-0.832344
H	0.024773	-0.284851	2.955601
H	2.605225	-0.136654	-0.553299
H	3.366464	0.479525	1.403707
H	-1.870905	-0.943093	1.535558
H	0.712008	-0.792418	-1.993808
H	-1.292920	0.230981	-0.688386
H	4.744216	0.480368	3.410836
H	3.987517	1.353791	4.790124
H	4.107390	2.120491	3.169351
H	4.277945	3.869761	0.341408
H	3.540971	3.119579	-1.093455
H	3.025240	2.611728	0.534934
H	8.072784	1.532338	-0.156914
H	6.849097	0.231931	-0.016261
H	7.423986	0.755122	-1.629260
H	5.552697	3.871359	-1.636426
H	7.037993	3.979100	-0.645833
H	7.049465	3.111035	-2.205258
H	-5.051024	1.190202	-0.304541
H	-3.664575	0.069650	-0.294952
H	-4.047866	1.041770	1.156594
H	-4.580776	4.777207	0.808428
H	-5.507034	3.949294	-0.471613
H	-5.077787	3.093561	1.039590
H	-3.209584	5.049177	-1.584169
H	-2.335933	5.163631	-0.029740
H	-1.752857	4.047092	-1.302188

50

dma-H⁺···am-Cl-*p*-dma

C	0.543462	-0.431794	0.049408
C	0.297004	-0.253939	1.422185
C	-0.411102	-1.199769	2.151821
C	-0.886210	-2.341896	1.508320
C	-0.670671	-2.528729	0.146806

C	0.046029	-1.581505	-0.583173
Cl	-1.744157	-3.562237	2.425354
N	1.279461	0.563328	-0.601455
C	1.818894	0.528912	-1.854158
O	1.691279	-0.406596	-2.632234
C	2.635598	1.747184	-2.215216
O	1.771003	2.809416	1.013246
C	2.871902	3.039227	1.534491
N	3.047766	4.089593	2.362407
C	1.959932	5.015708	2.609072
C	4.054122	2.143352	1.253796
C	4.313441	4.439900	2.977342
O	2.345137	-3.069741	1.830928
C	2.516496	-3.417429	0.582110
N	3.303656	-2.655700	-0.118806
C	3.646549	-2.929047	-1.510621
C	1.863725	-4.645220	0.055000
C	3.904403	-1.459871	0.471174
H	0.228648	-1.733527	-1.644095
H	0.673711	0.642656	1.919419
H	1.475069	1.404630	-0.034107
H	-1.054524	-3.419689	-0.352949
H	-0.584911	-1.052094	3.219071
H	1.686984	-3.636520	2.264829
H	2.483276	2.588756	-1.527397
H	2.384562	2.052584	-3.240253
H	3.700804	1.468174	-2.203602
H	4.904795	2.711534	0.850500
H	4.389356	1.631473	2.168504
H	3.750788	1.388665	0.521434
H	2.252529	6.033615	2.304683
H	1.080140	4.706537	2.036374
H	1.707462	5.034144	3.681408
H	4.992442	3.582463	3.024922
H	4.810492	5.256906	2.427627
H	4.131168	4.780739	4.007193
H	2.627459	-5.428804	-0.071430
H	1.103997	-5.007111	0.759419
H	1.390127	-4.456806	-0.916133
H	3.131473	-2.198958	-2.152411
H	4.732933	-2.819713	-1.624065
H	3.364284	-3.945603	-1.797182
H	4.819779	-1.729964	1.017826
H	4.160128	-0.772115	-0.342110
H	3.198738	-0.975116	1.154647

NMR spectra

***N*-(4-chlorophenyl)cyclohexanecarboxamide: (3)**

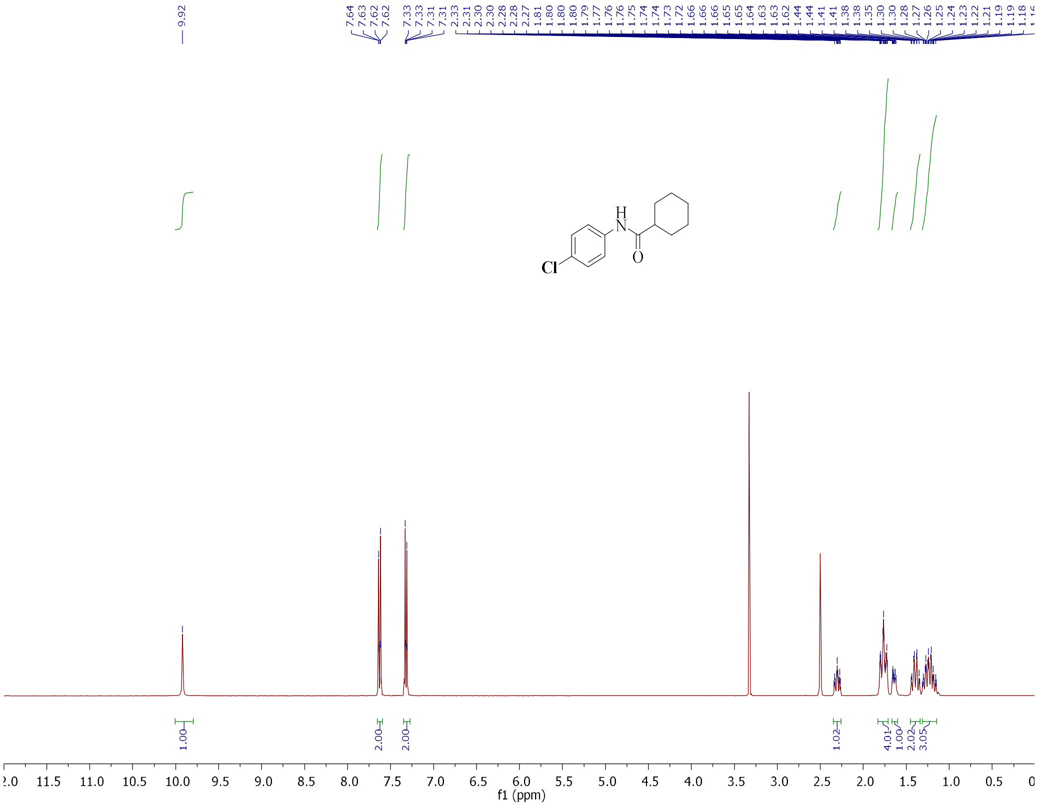

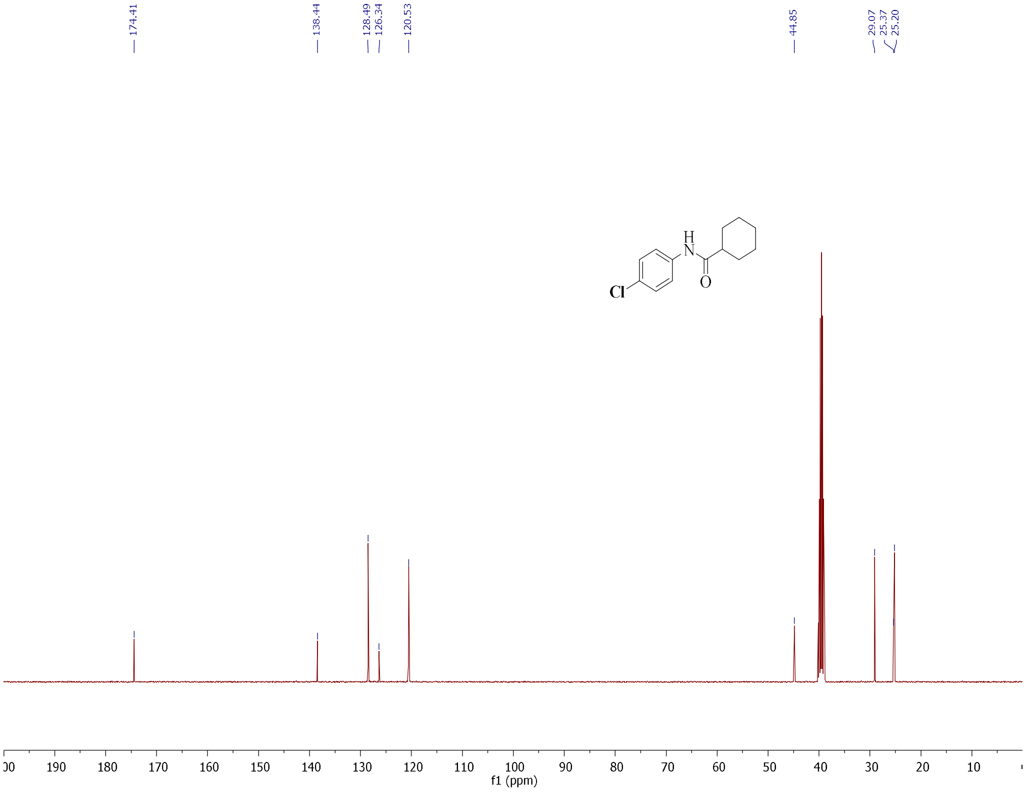

_N_-(4-chloro-3-methylphenyl)cyclohexanecarboxamide: (5)

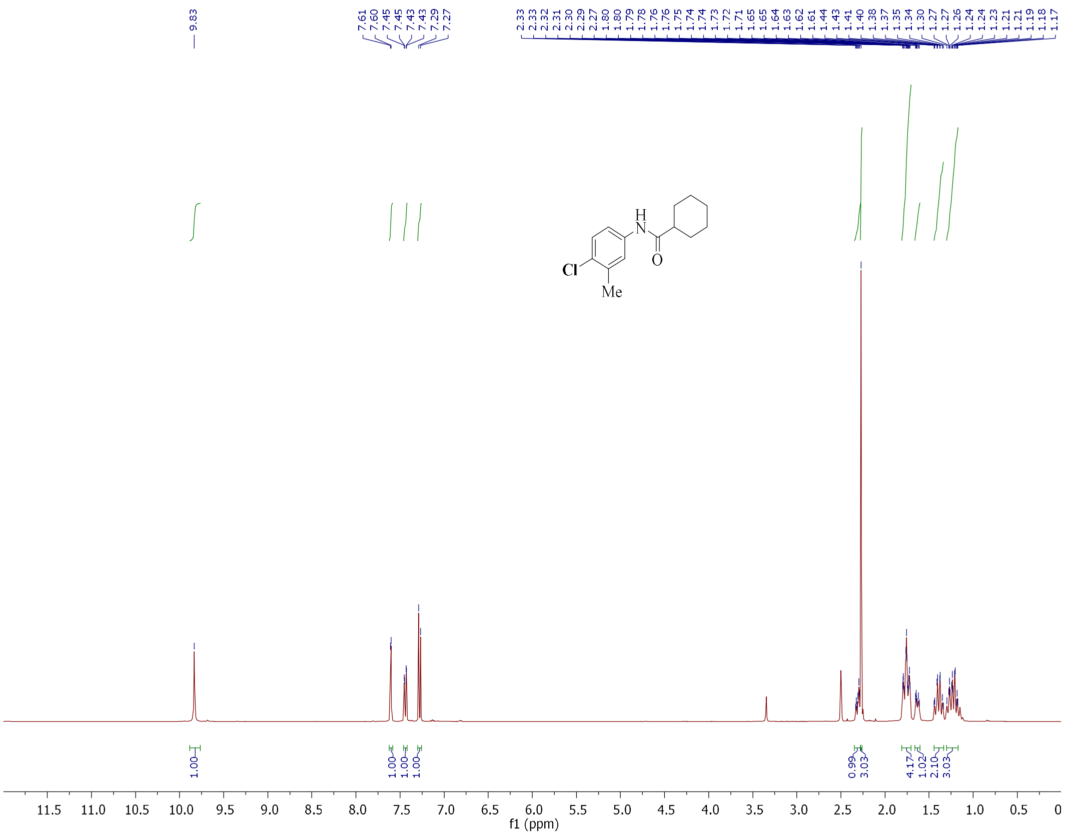

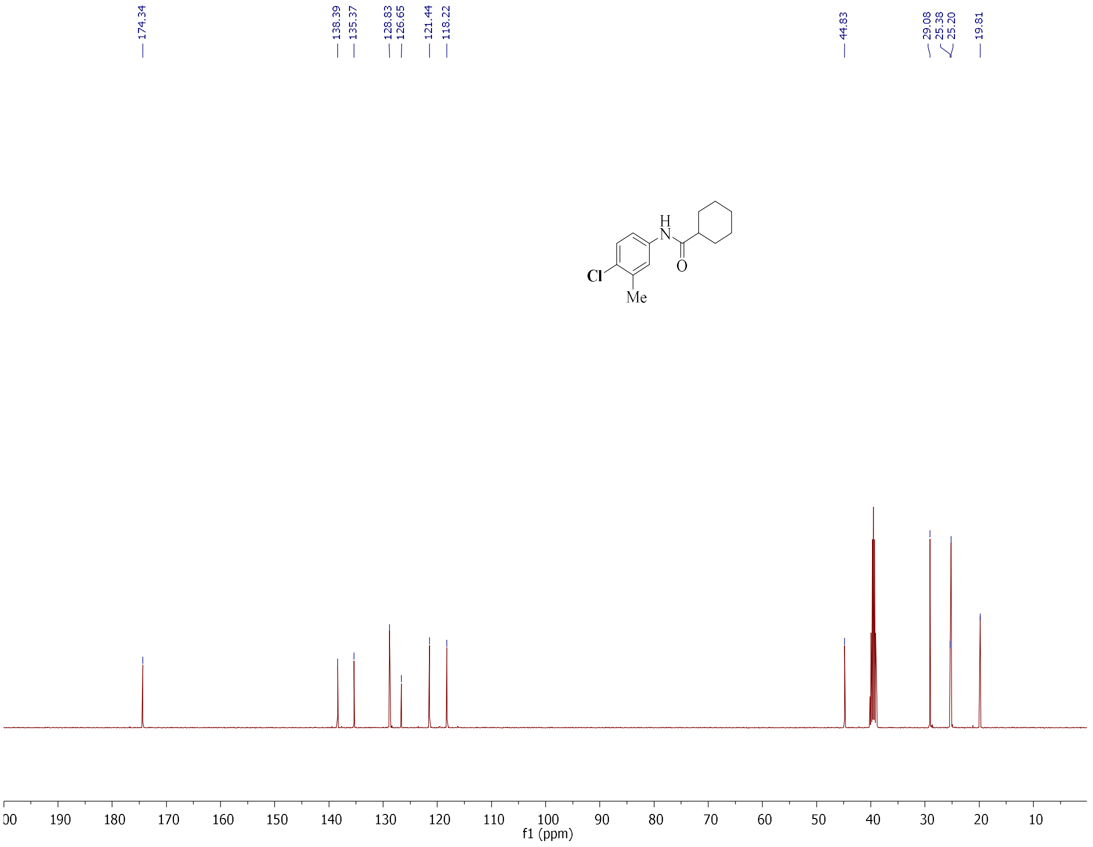

(2-Chloro-5-(cyclohexanecarboxamido)phenyl)boronic acid: (6)

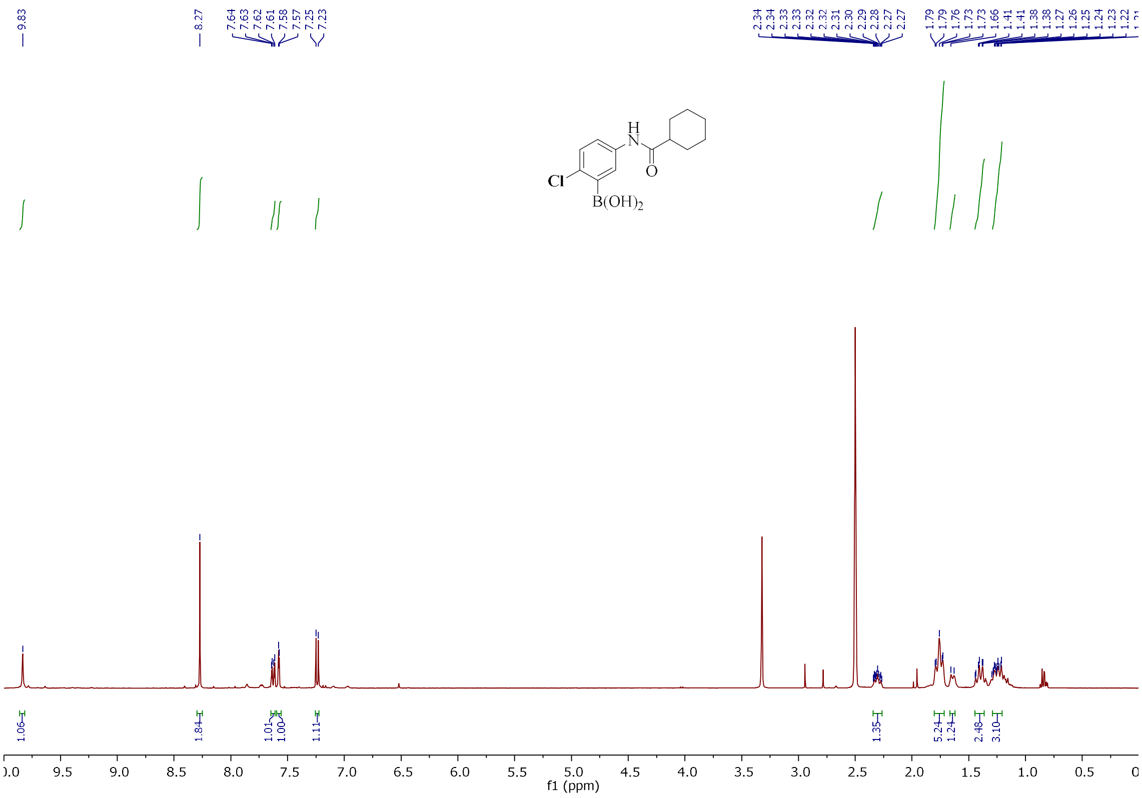

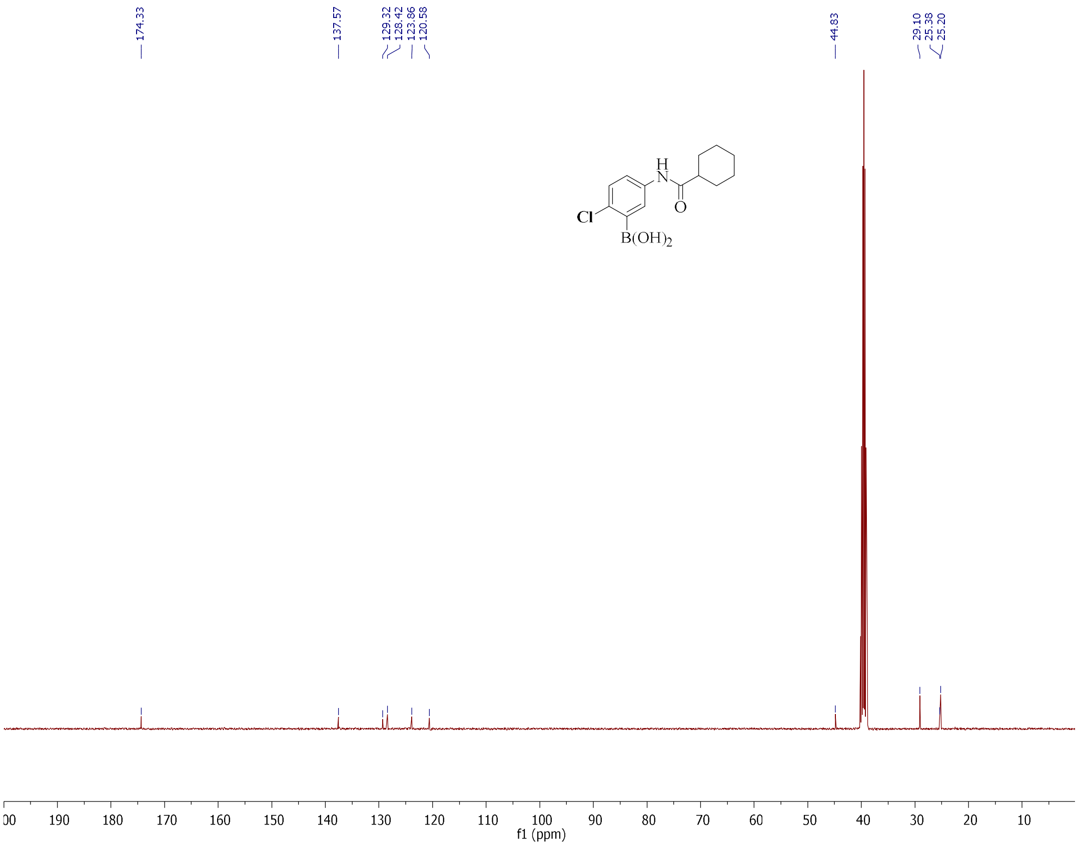

***N*-(3,4-dichlorophenyl)cyclohexanecarboxamide: (7)**

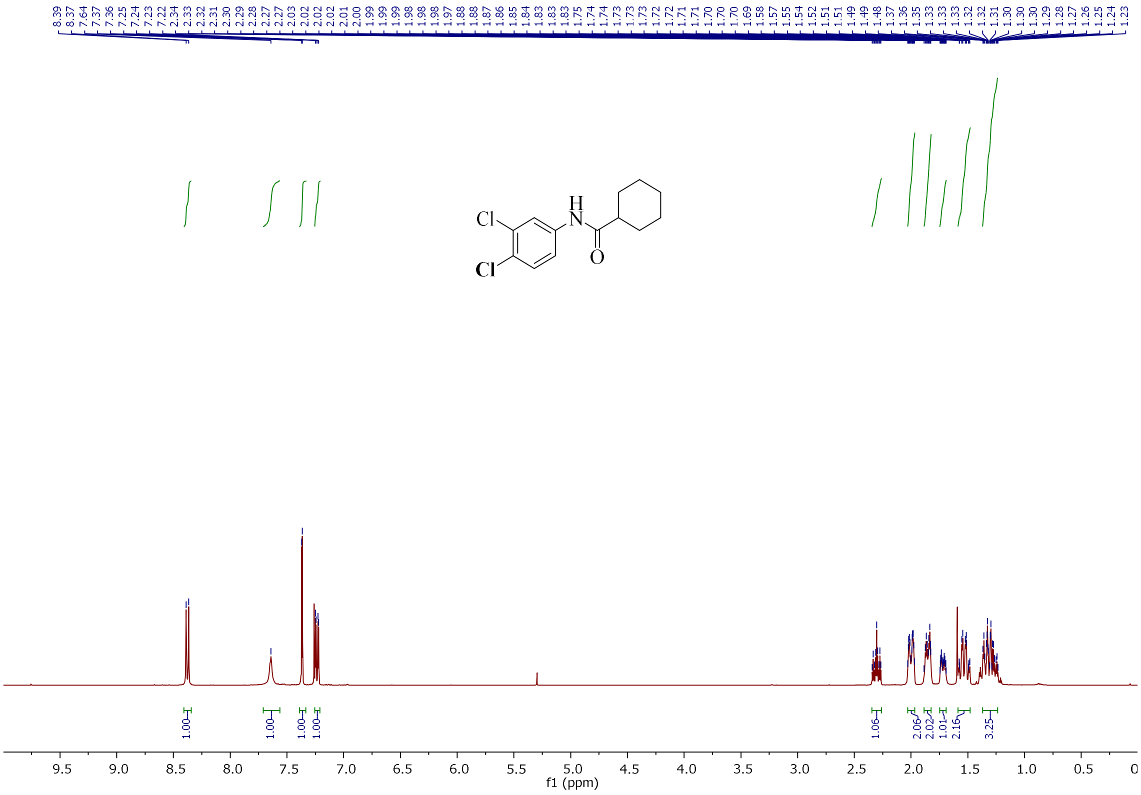

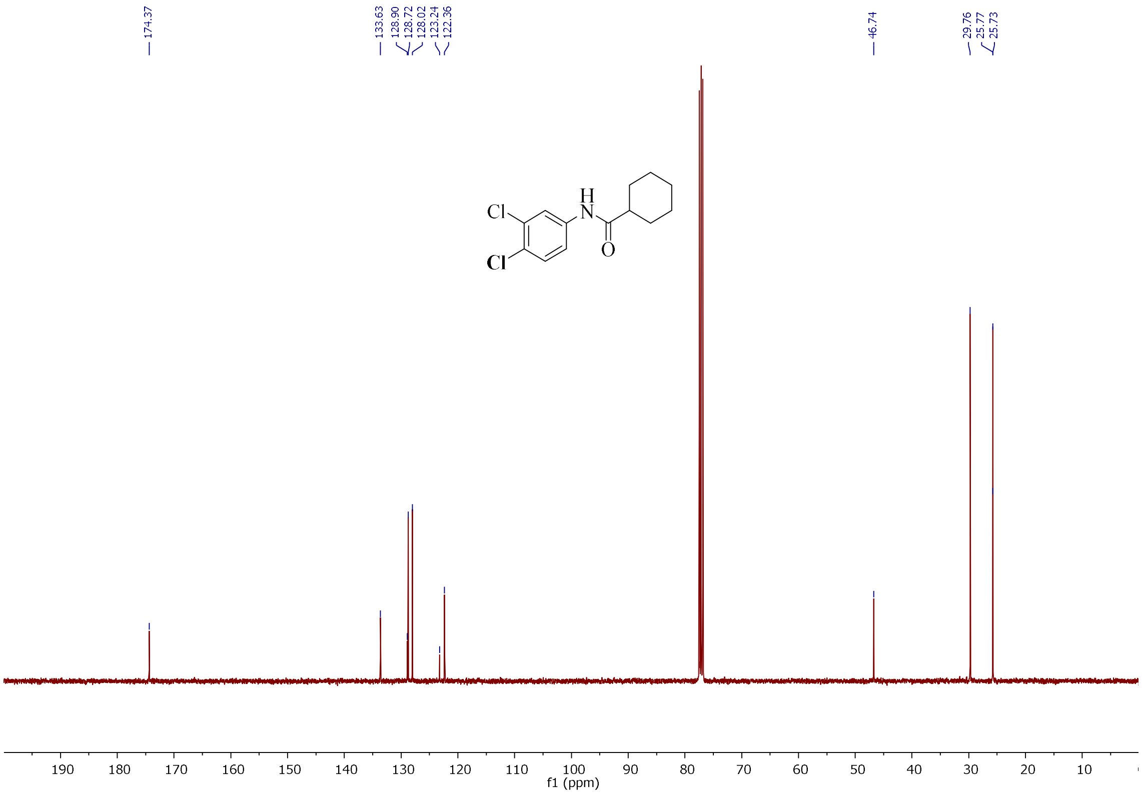

***N*-(3-bromo-4-chlorophenyl)cyclohexanecarboxamide: (8)**

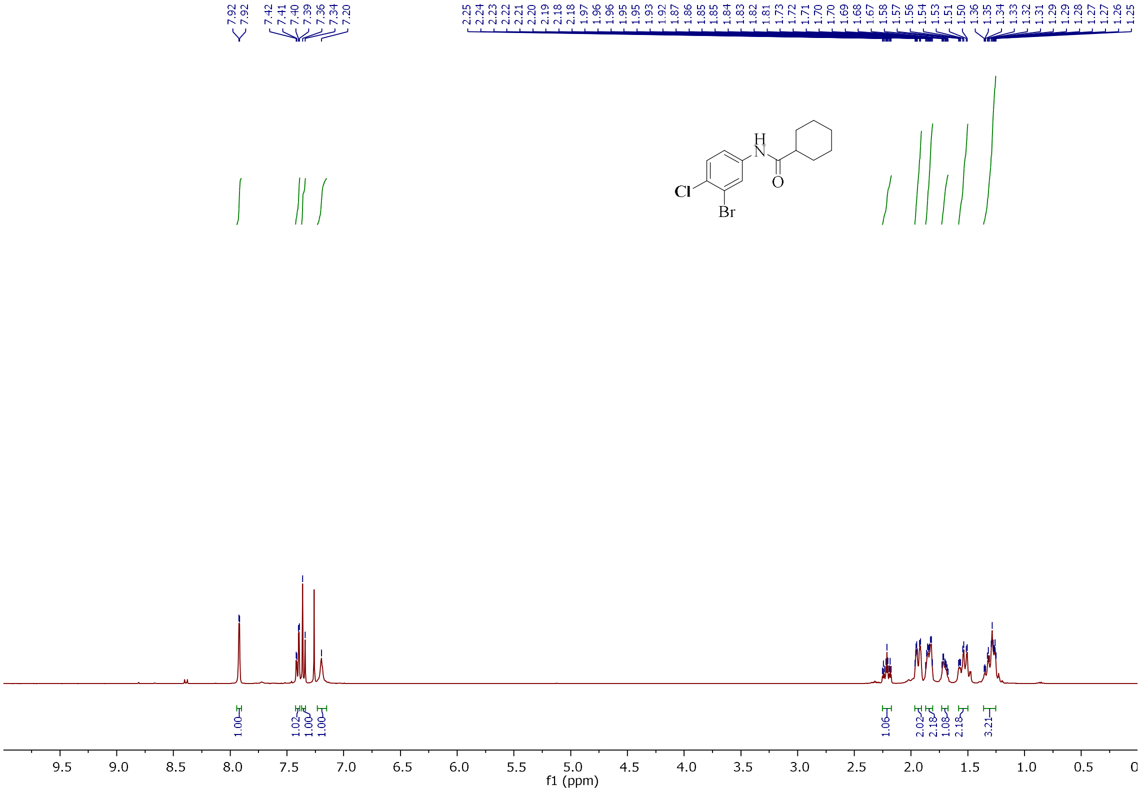

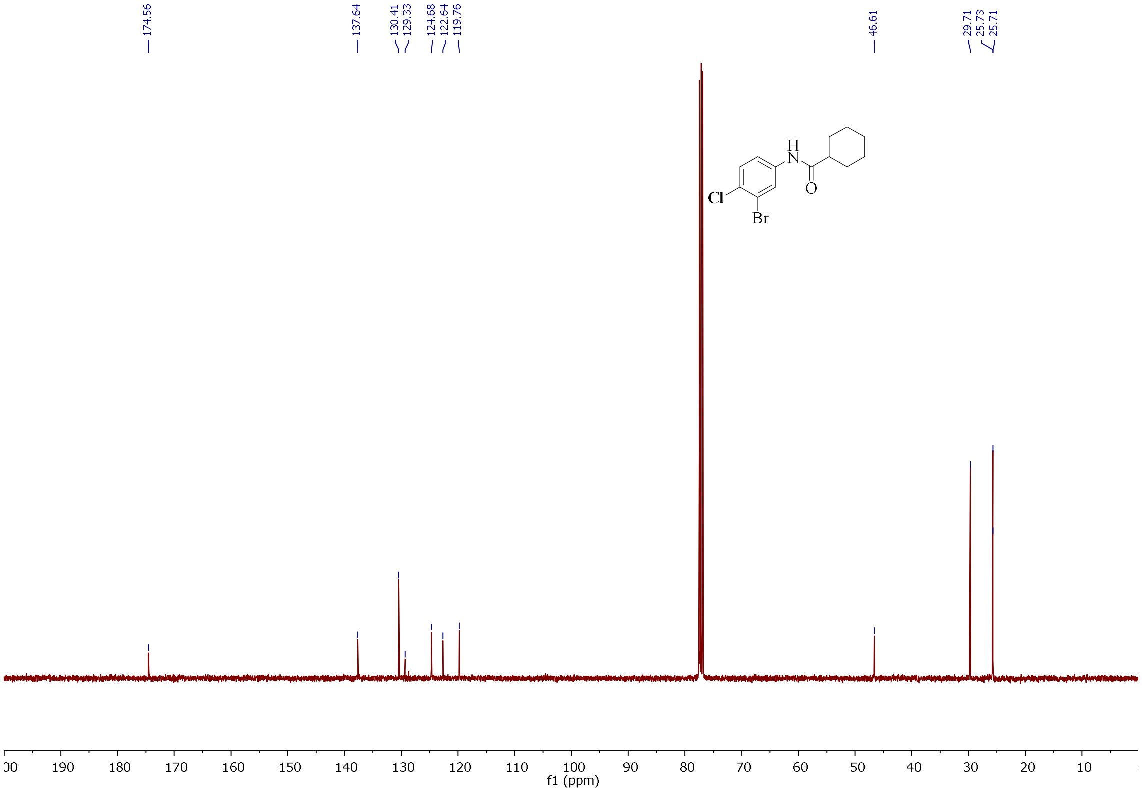

2-Chloro-5-(cyclohexanecarboxamido)benzoic acid: (9)

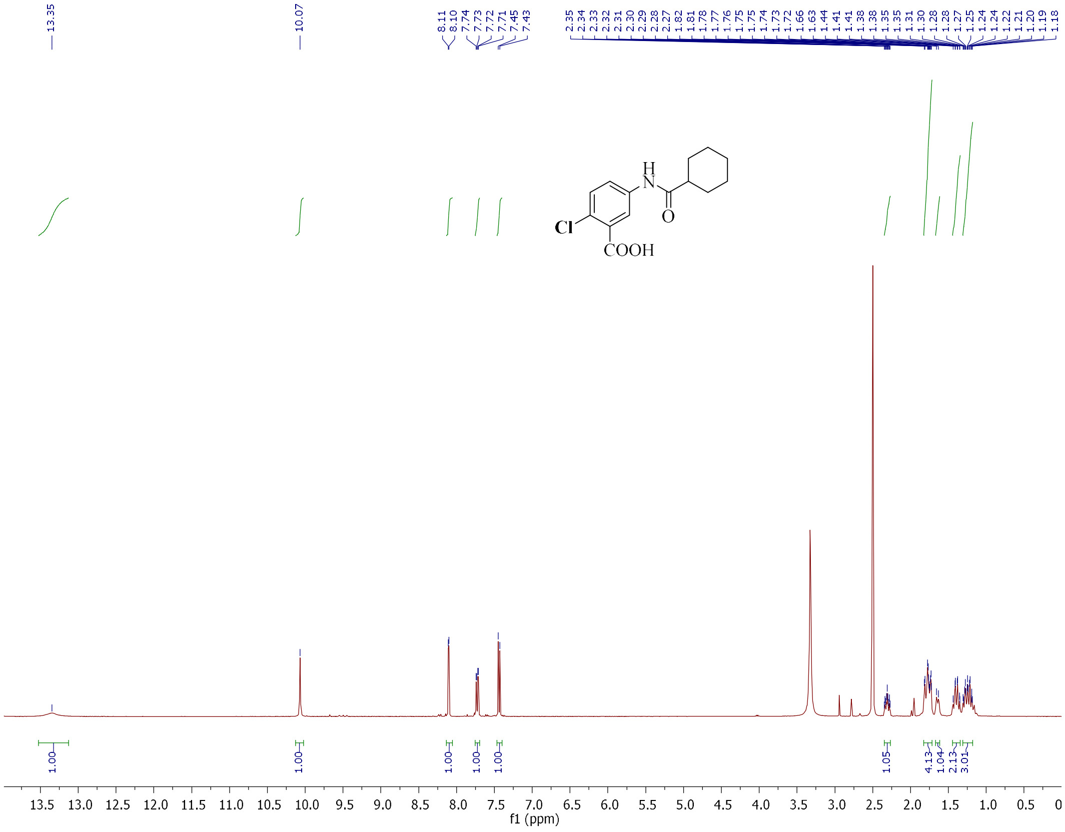

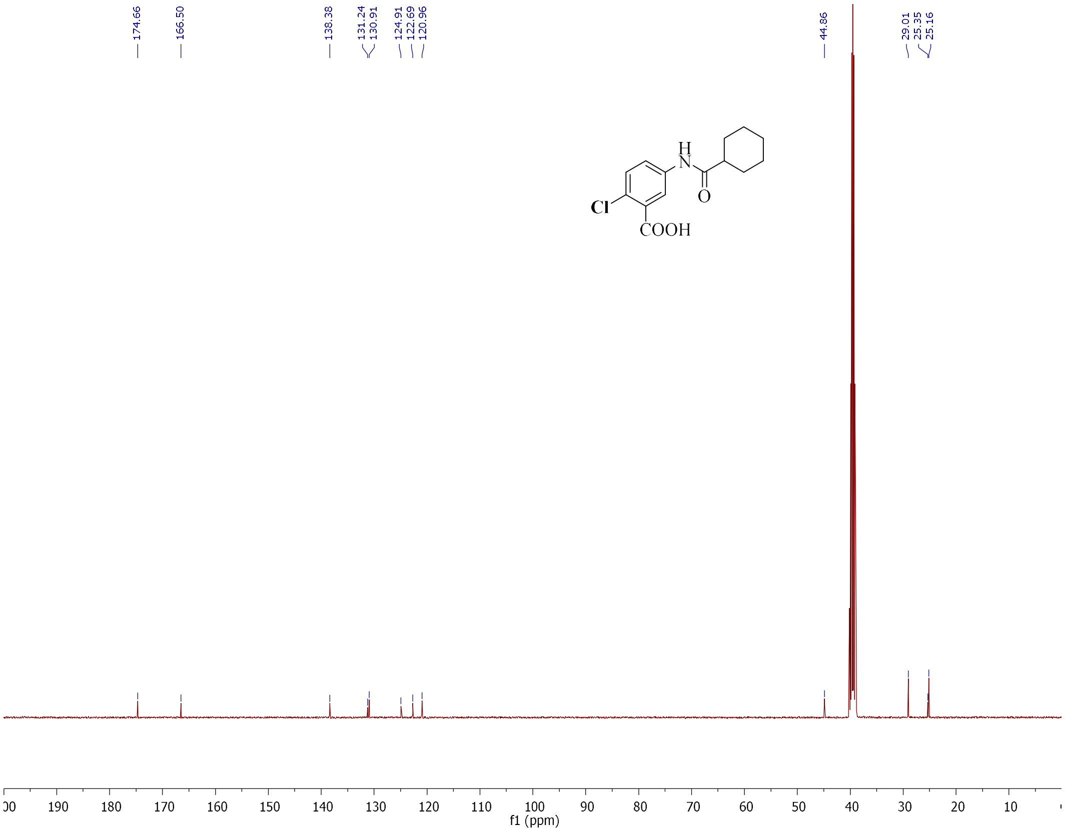

Ethyl 2-chloro-5-(cyclohexanecarboxamido)benzoate: (10)

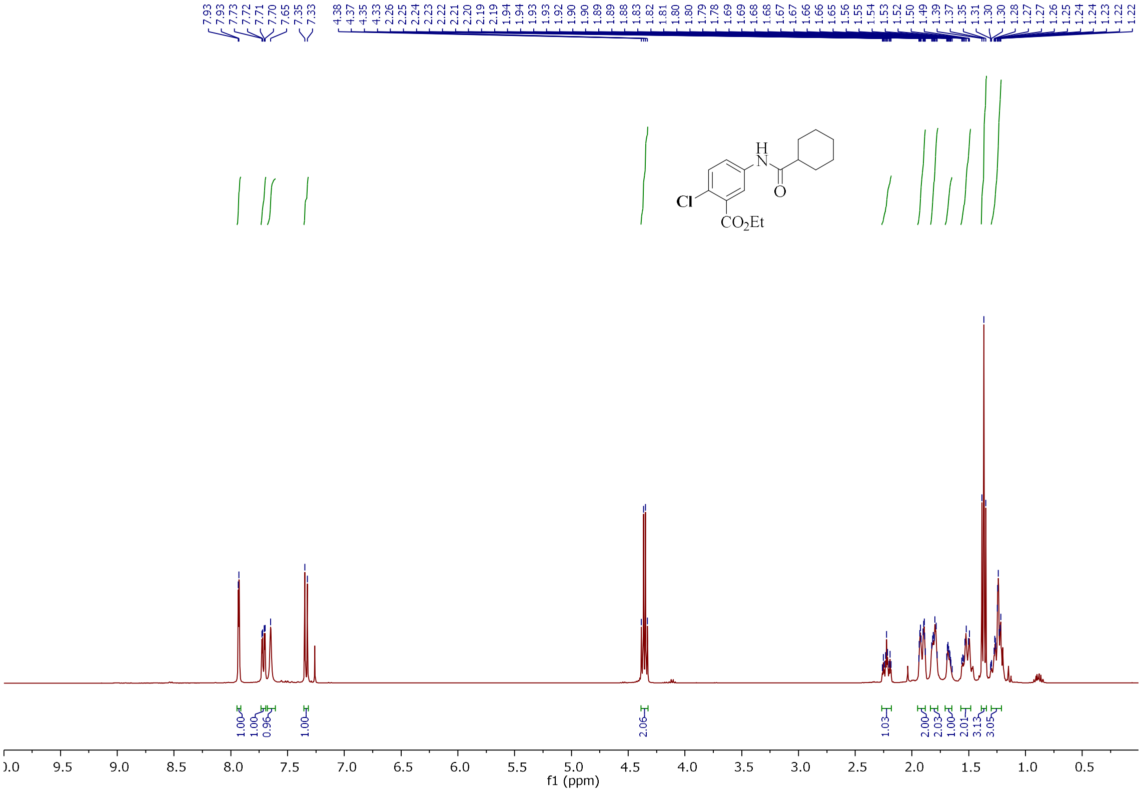

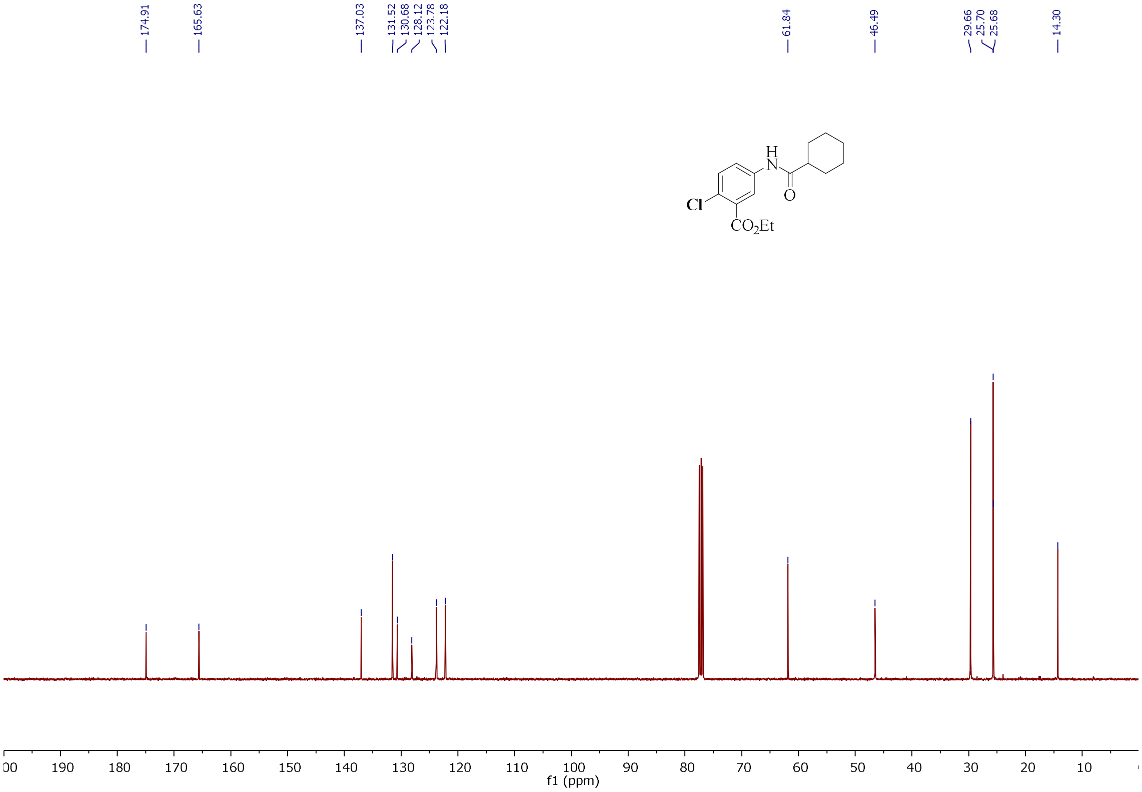

***N*-(3-benzoyl-4-chlorophenyl)cyclohexanecarboxamide: (11)**

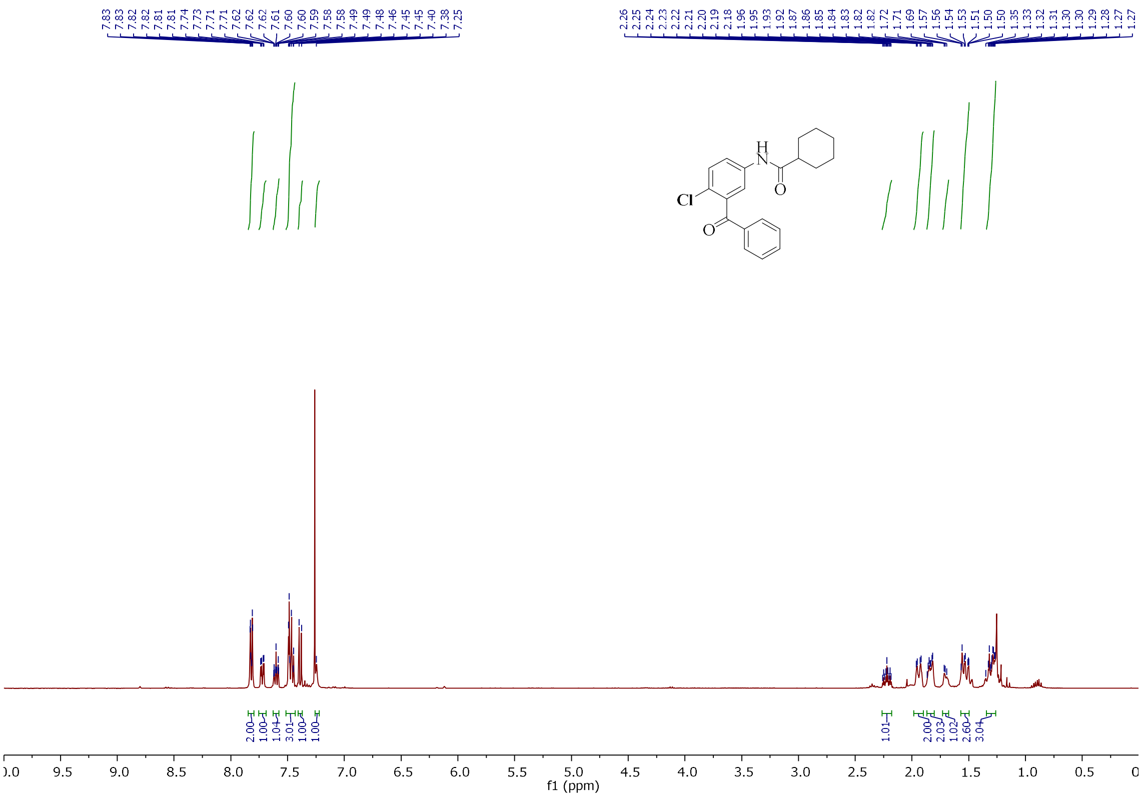

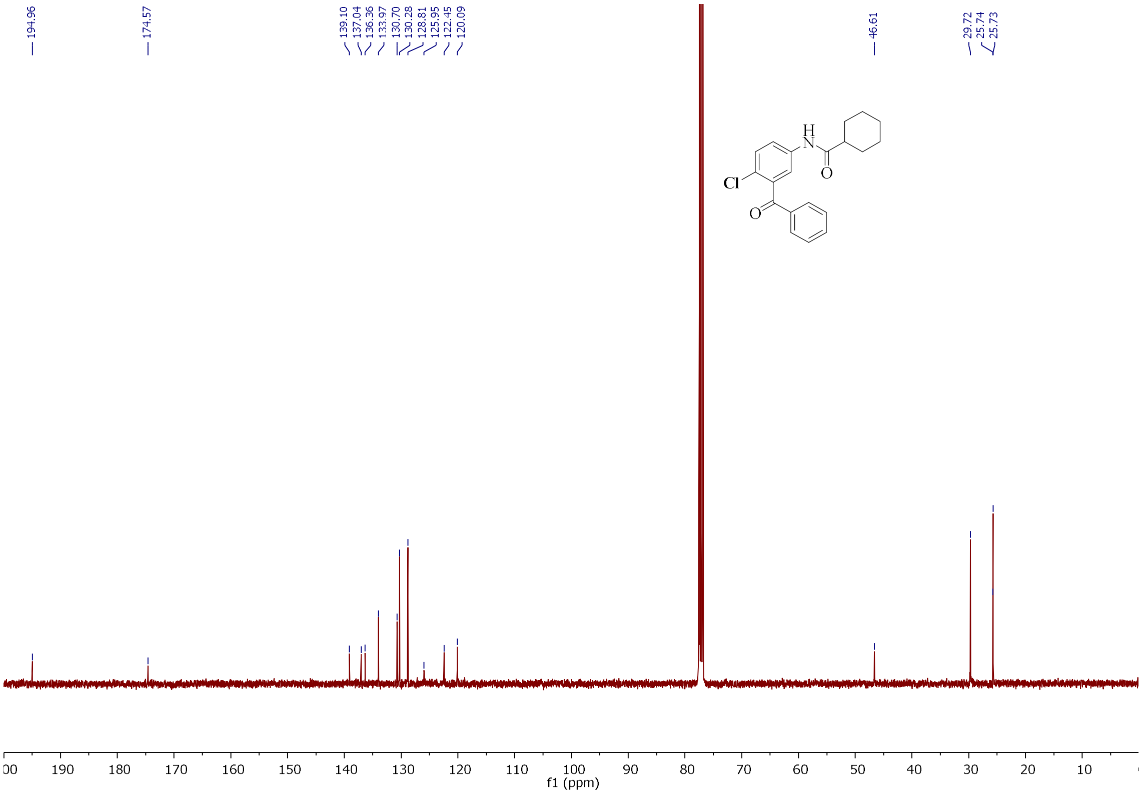

***N*-(5-chloro-2-methoxyphenyl)cyclohexanecarboxamide: (12)**

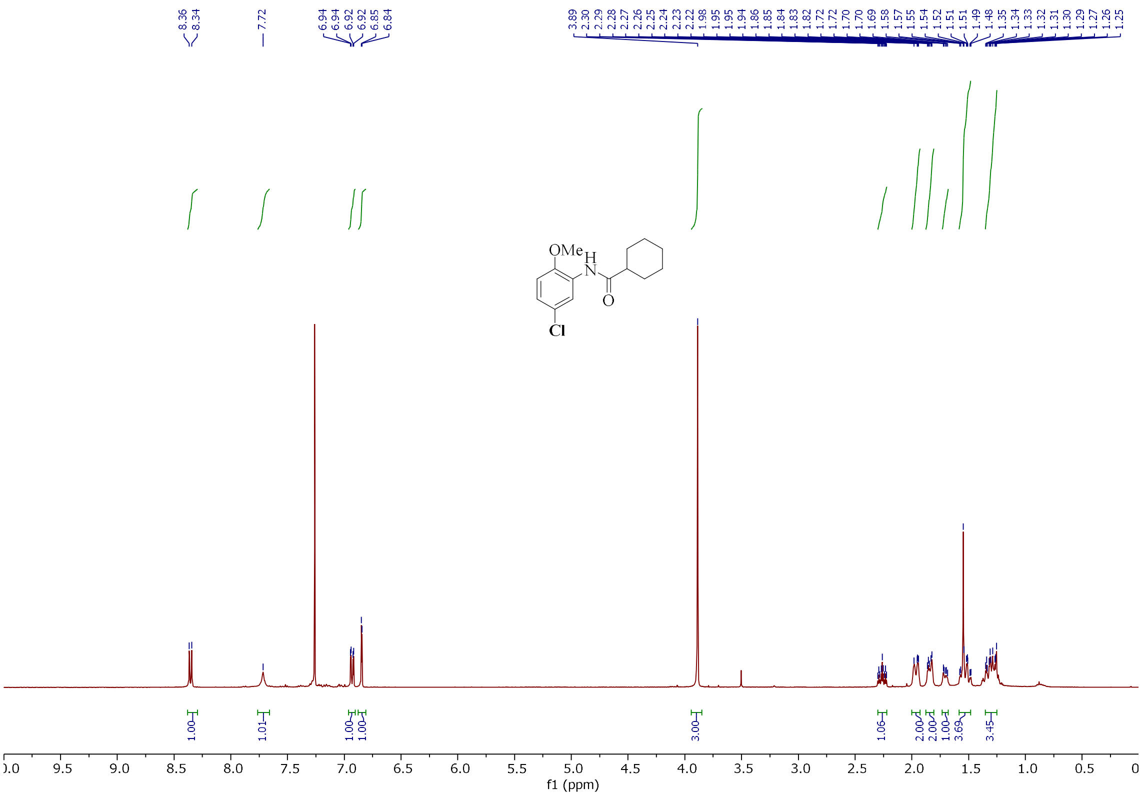

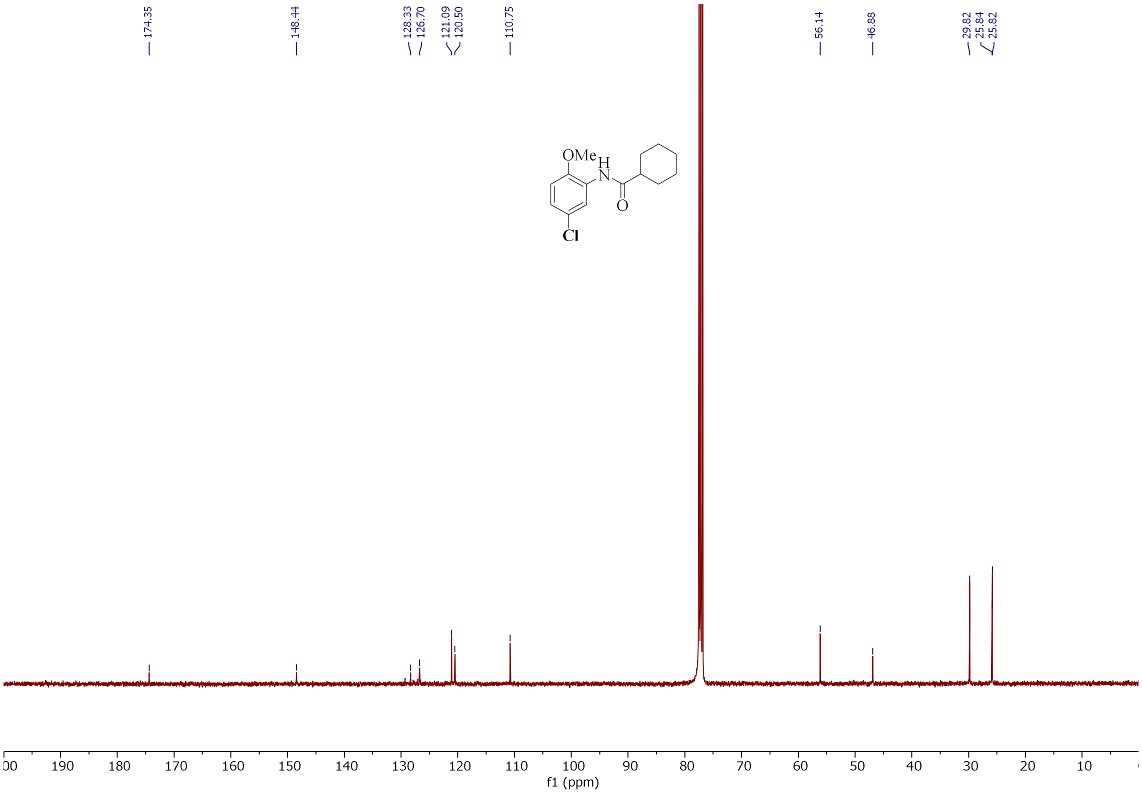

_N_-(2-benzoyl-4-chlorophenyl)cyclohexanecarboxamide: (13)

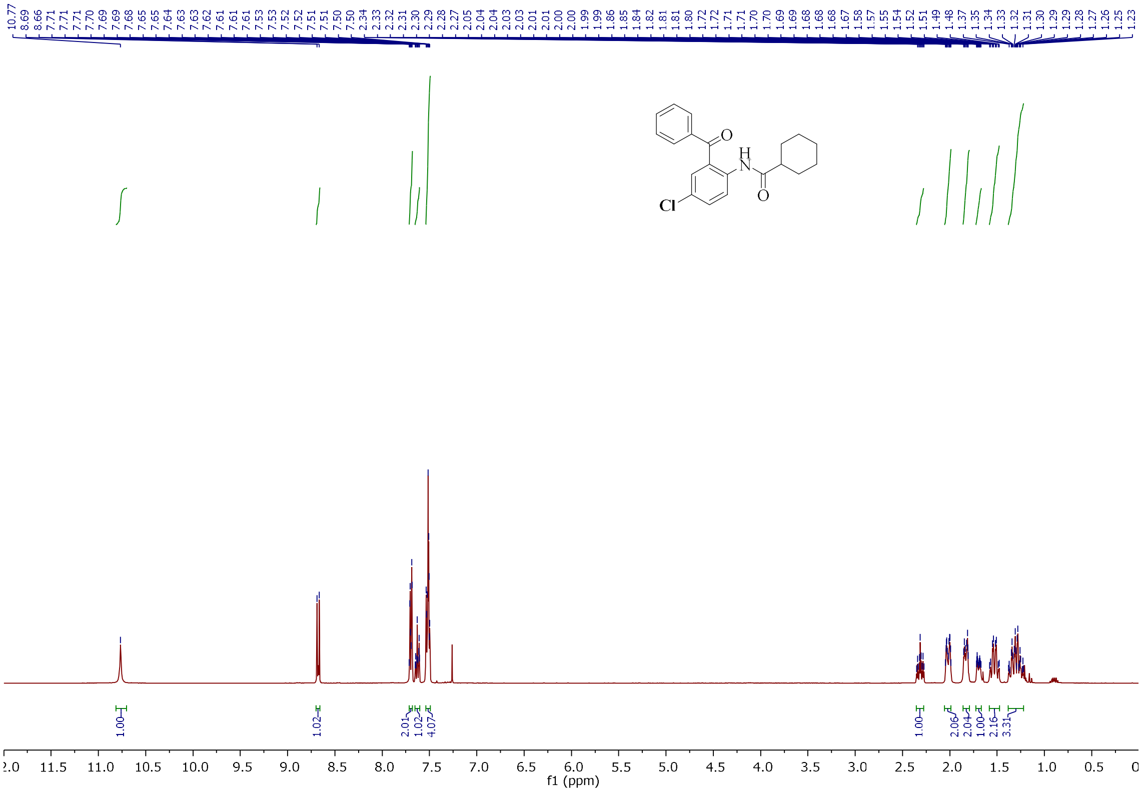

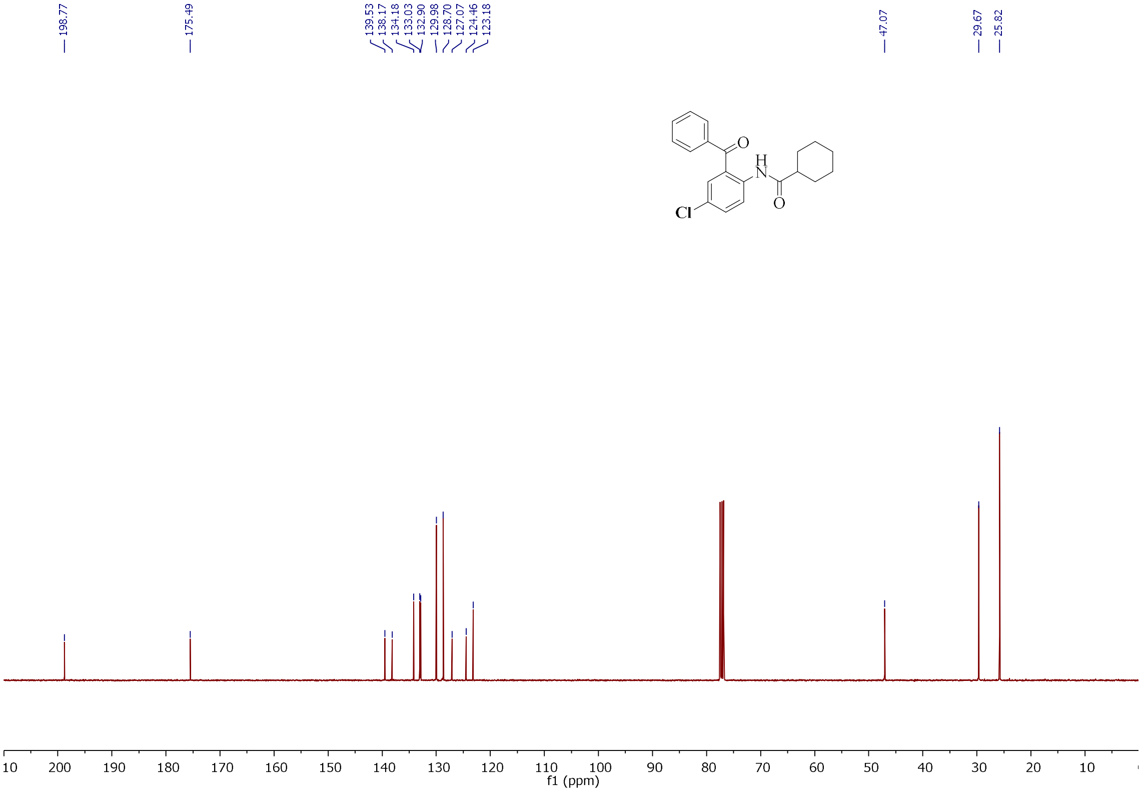

N-(4-chloro-2-methoxy-5-methylphenyl)cyclohexanecarboxamide: (14)

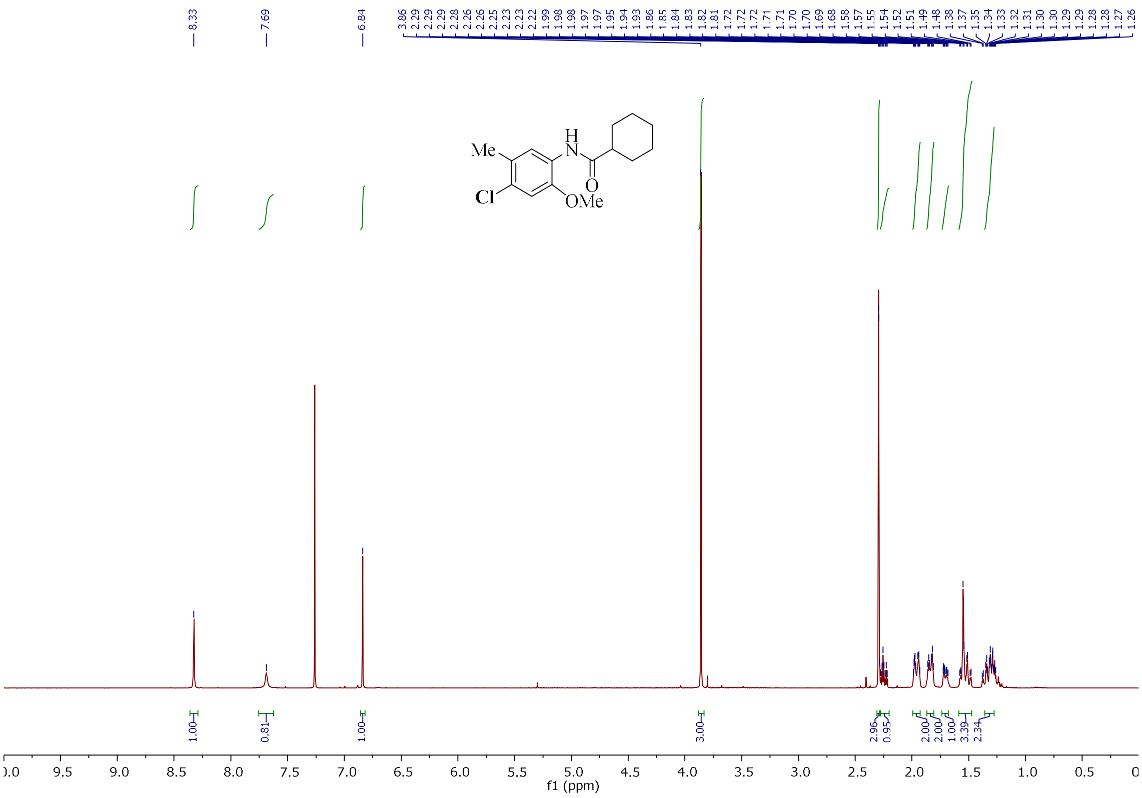

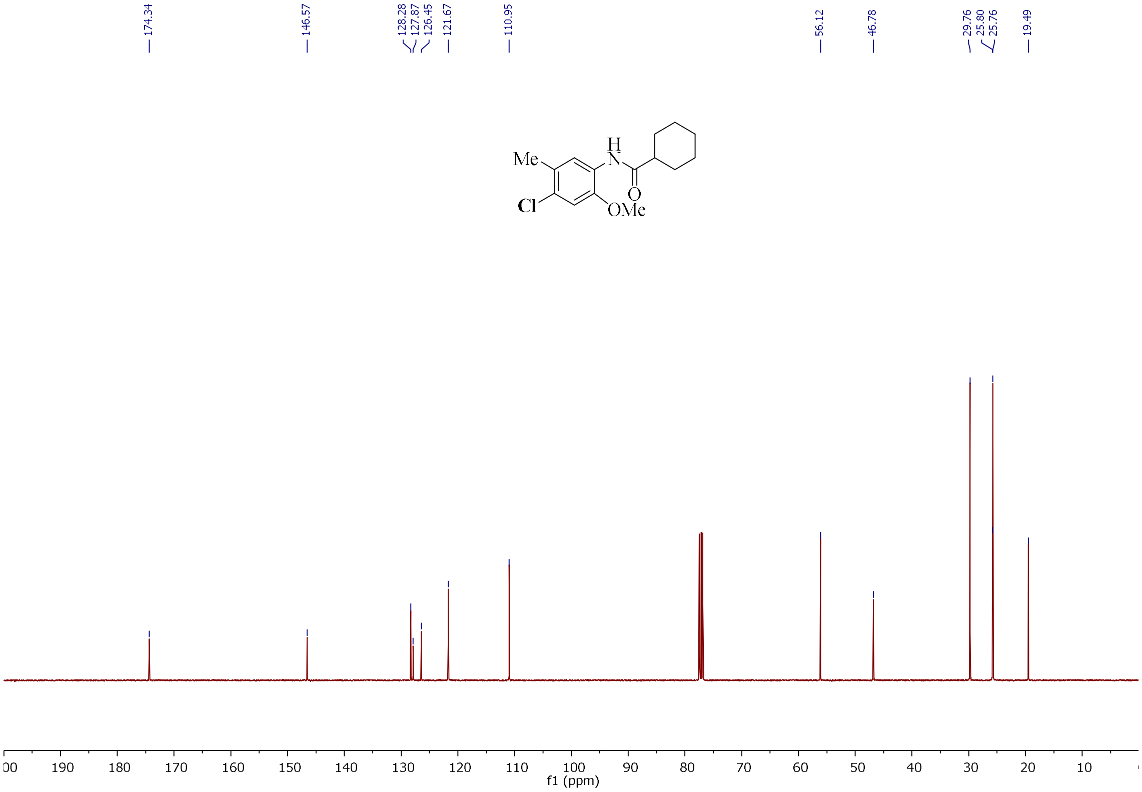

***N*-(2,4-dichloro-5-methoxyphenyl)cyclohexanecarboxamide: (15)**

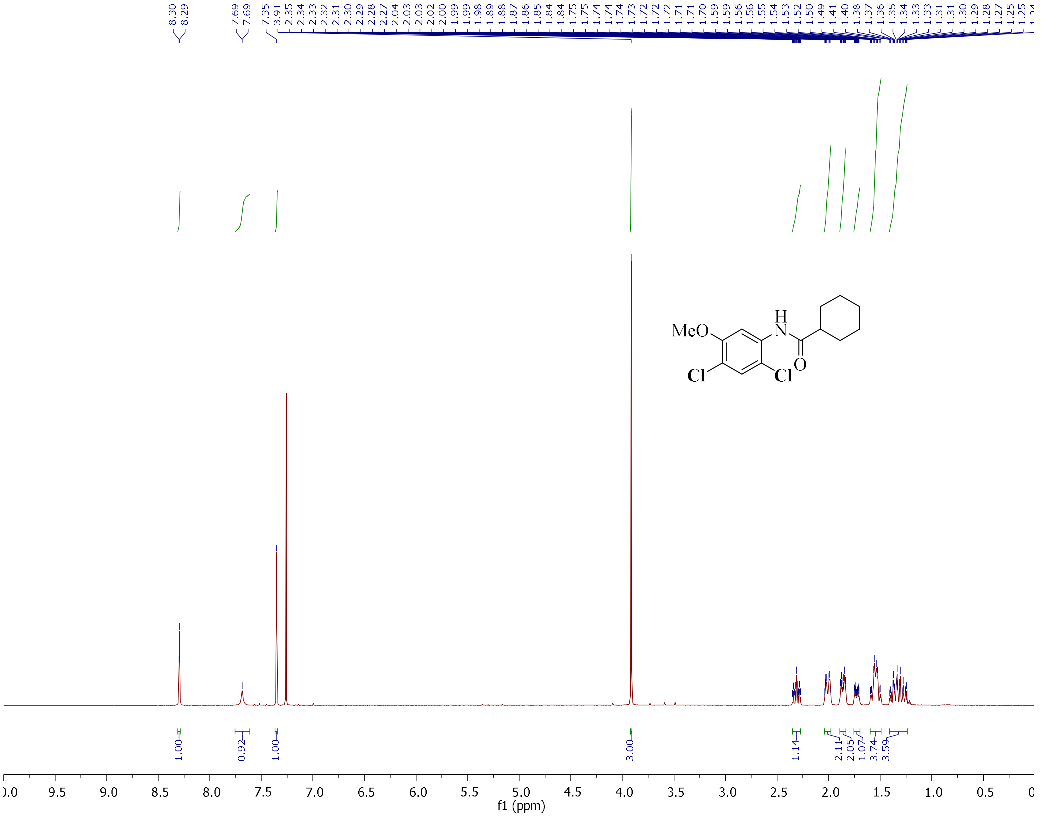

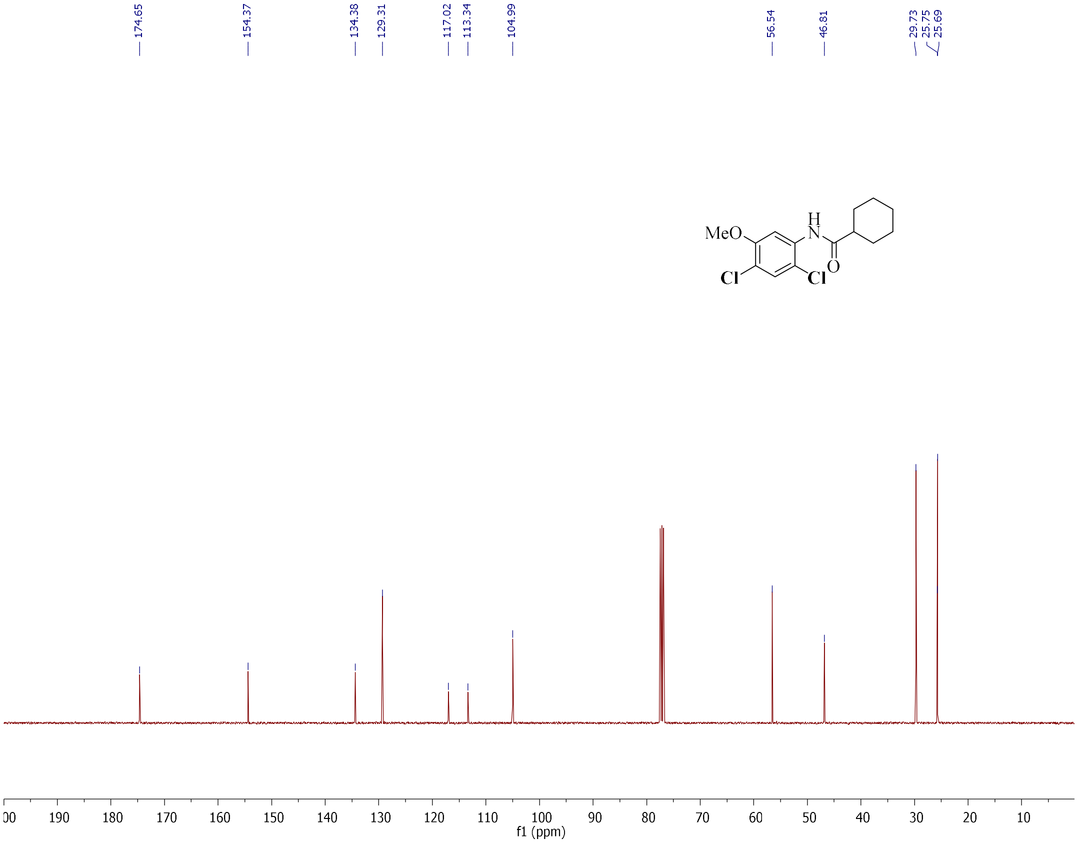

_N_-(3,4-dichloro-2-methylphenyl)cyclohexanecarboxamide: (16)

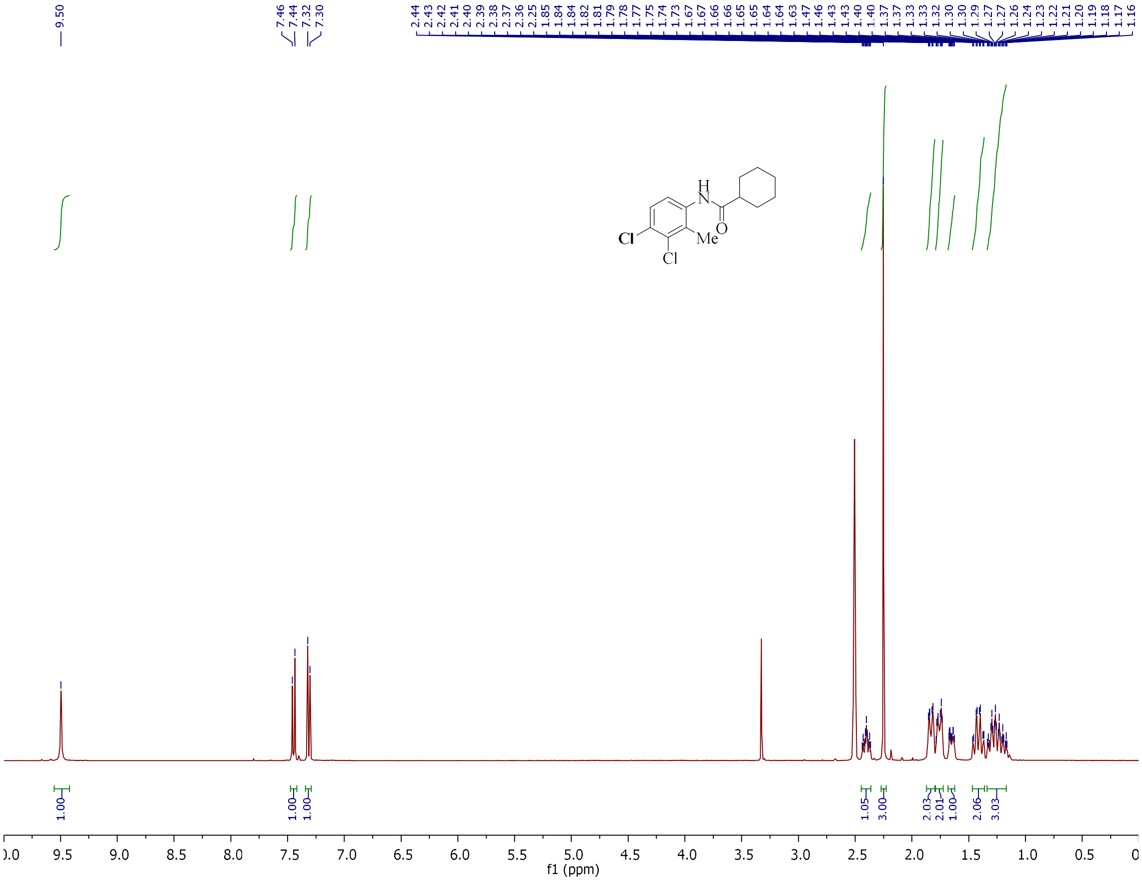

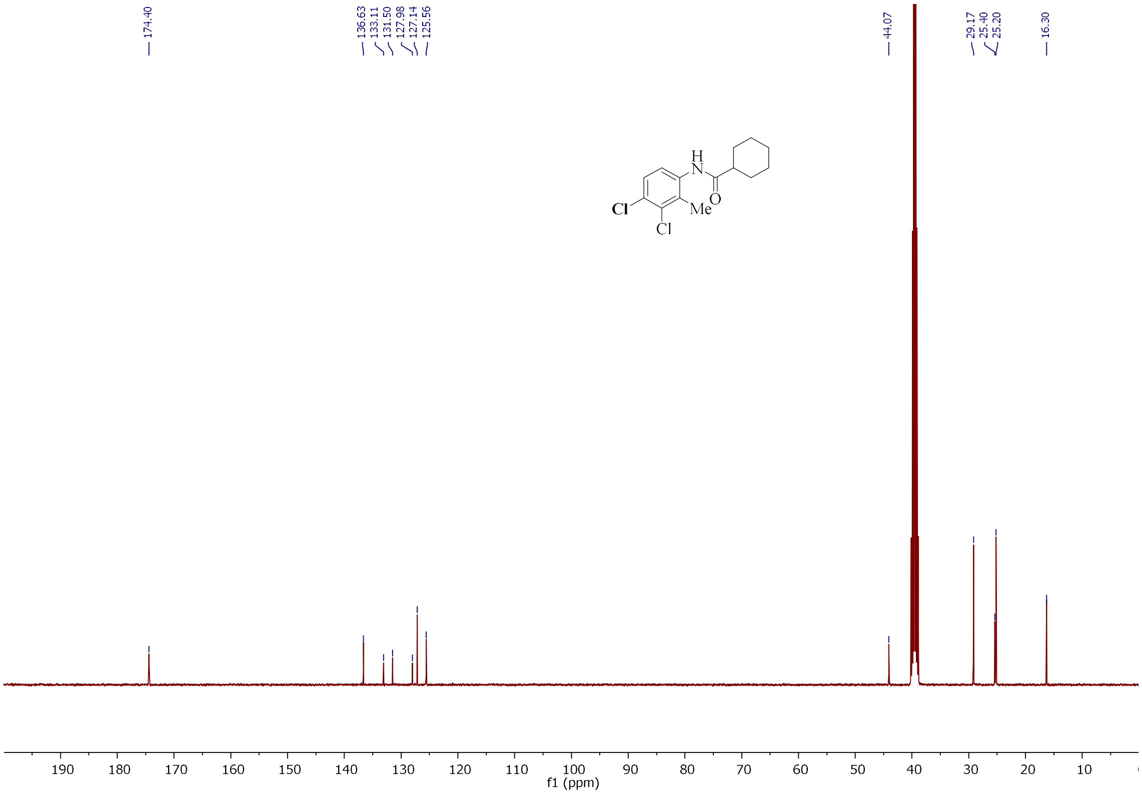

***N,N'*-(4-chloro-1,2-phenylene)dicyclohexanecarboxamide: (17)**

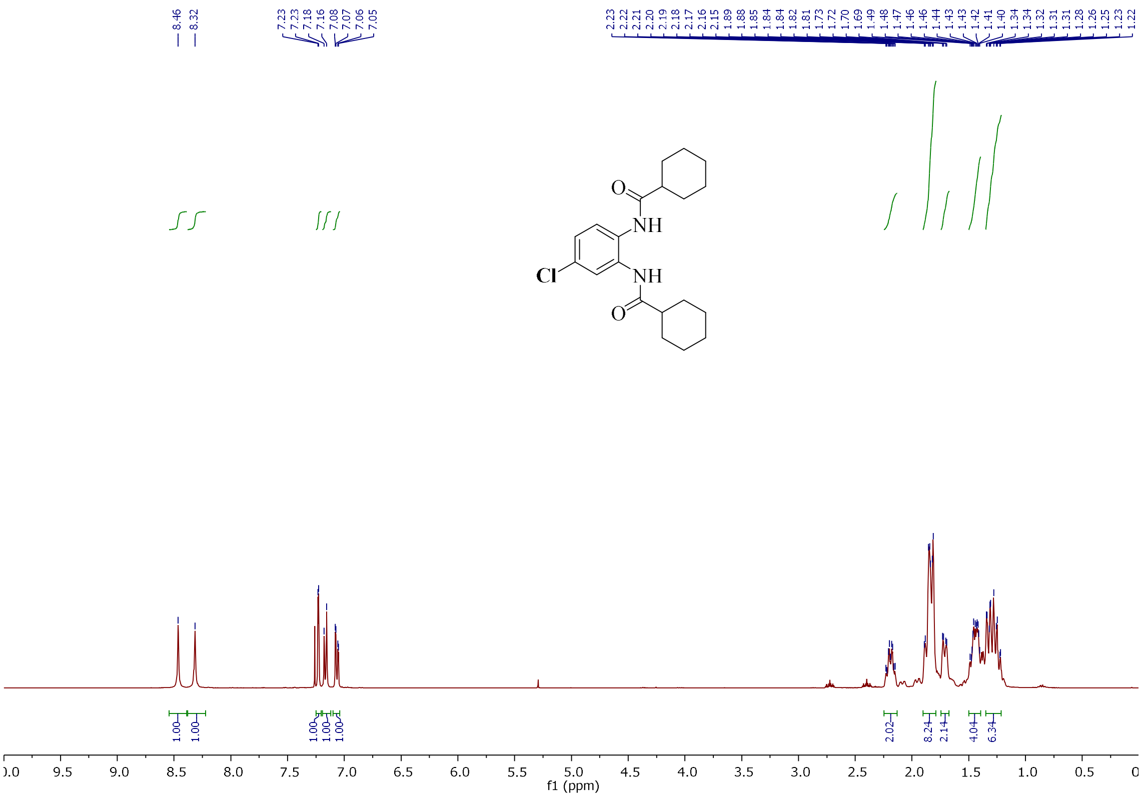

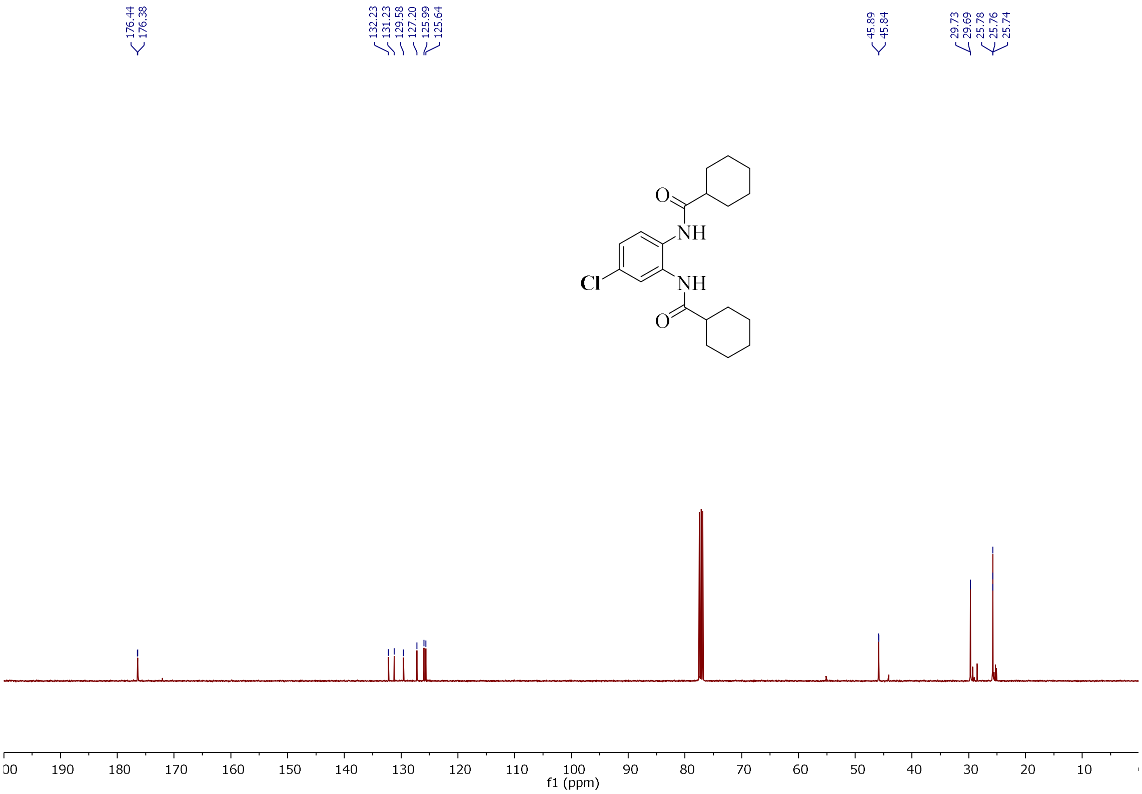

***N*-(4-chlorophenyl)-N-methylcyclohexanecarboxamide: (18)**

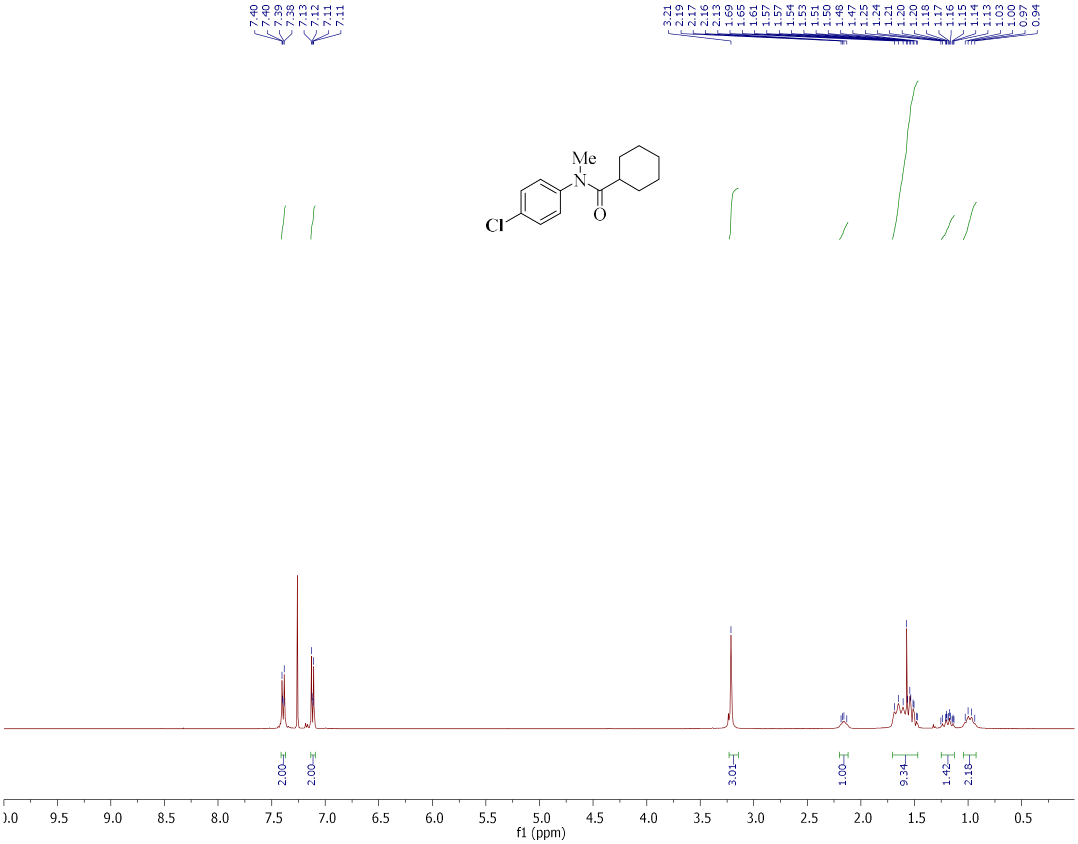

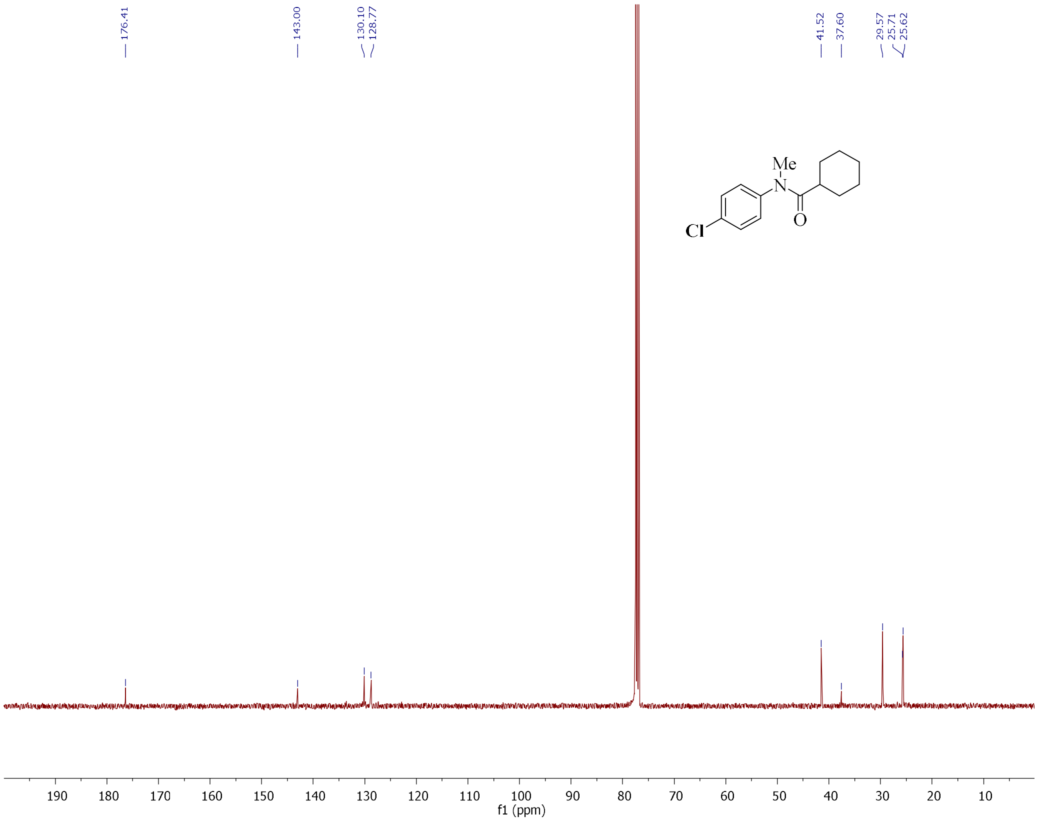

N-benzyl-N-(4-chlorophenyl)cyclohexanecarboxamide: (19)

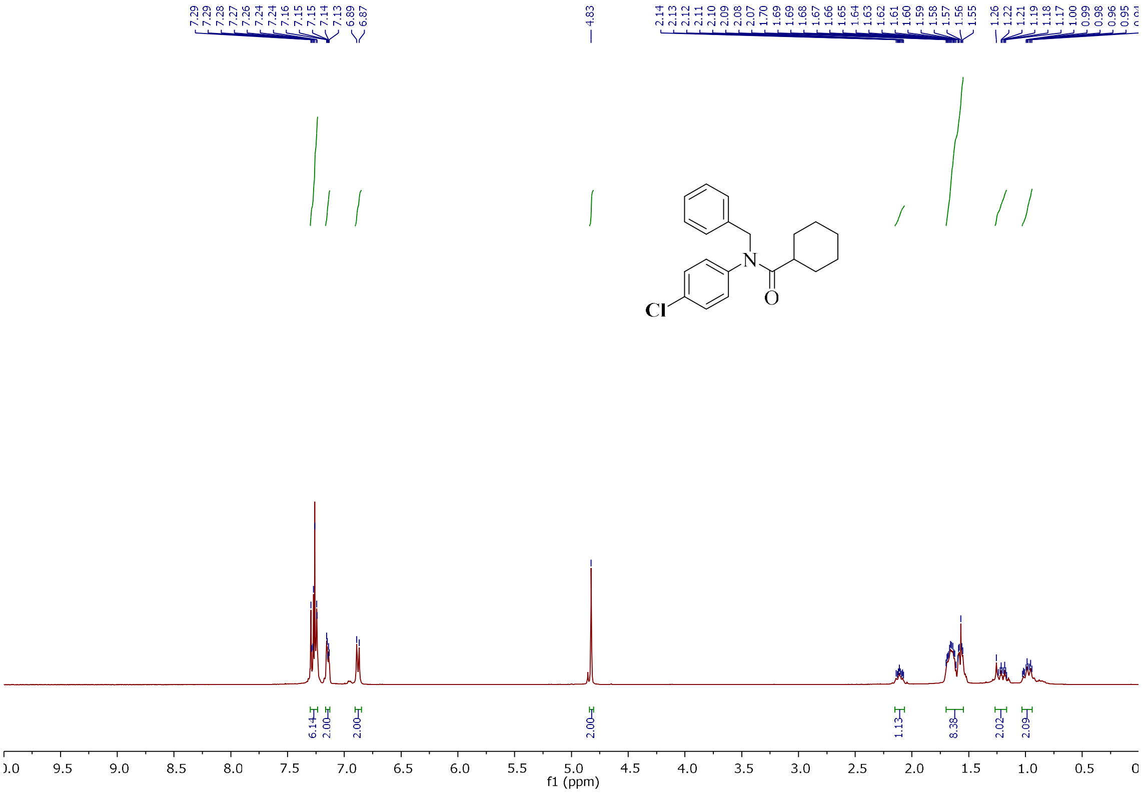

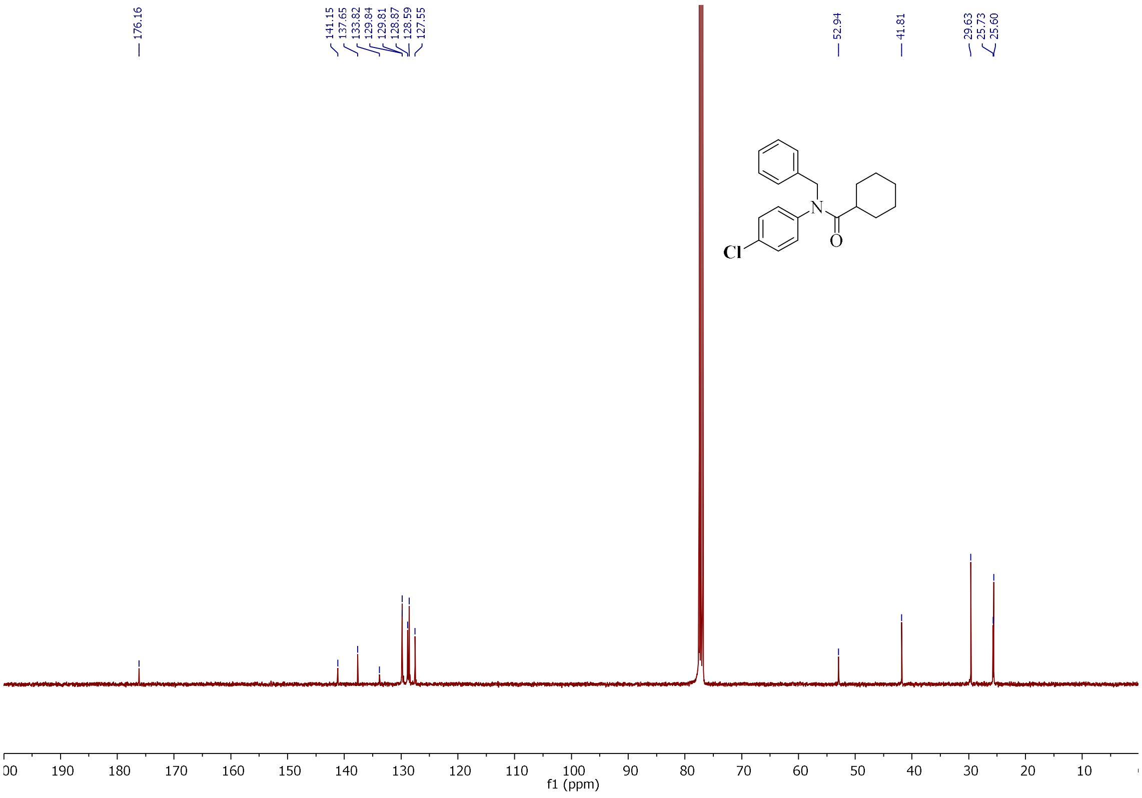

_N_-(4-chlorophenyl)acetamide: (20)

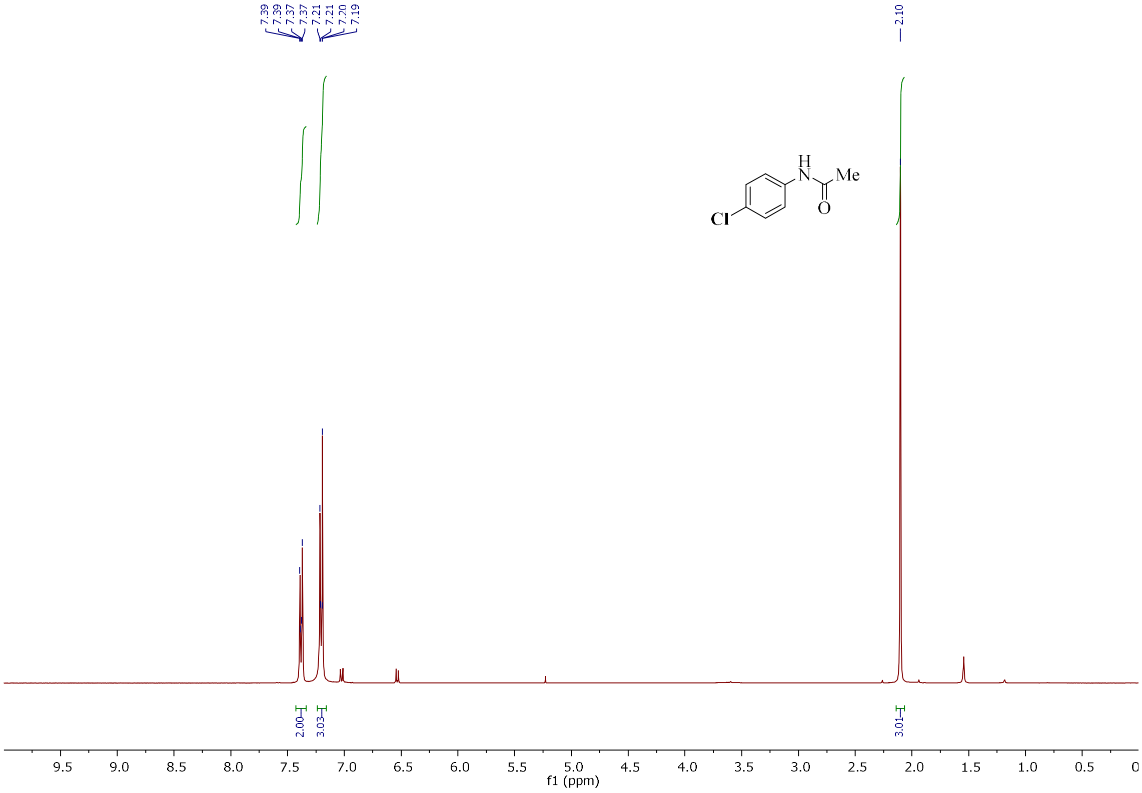

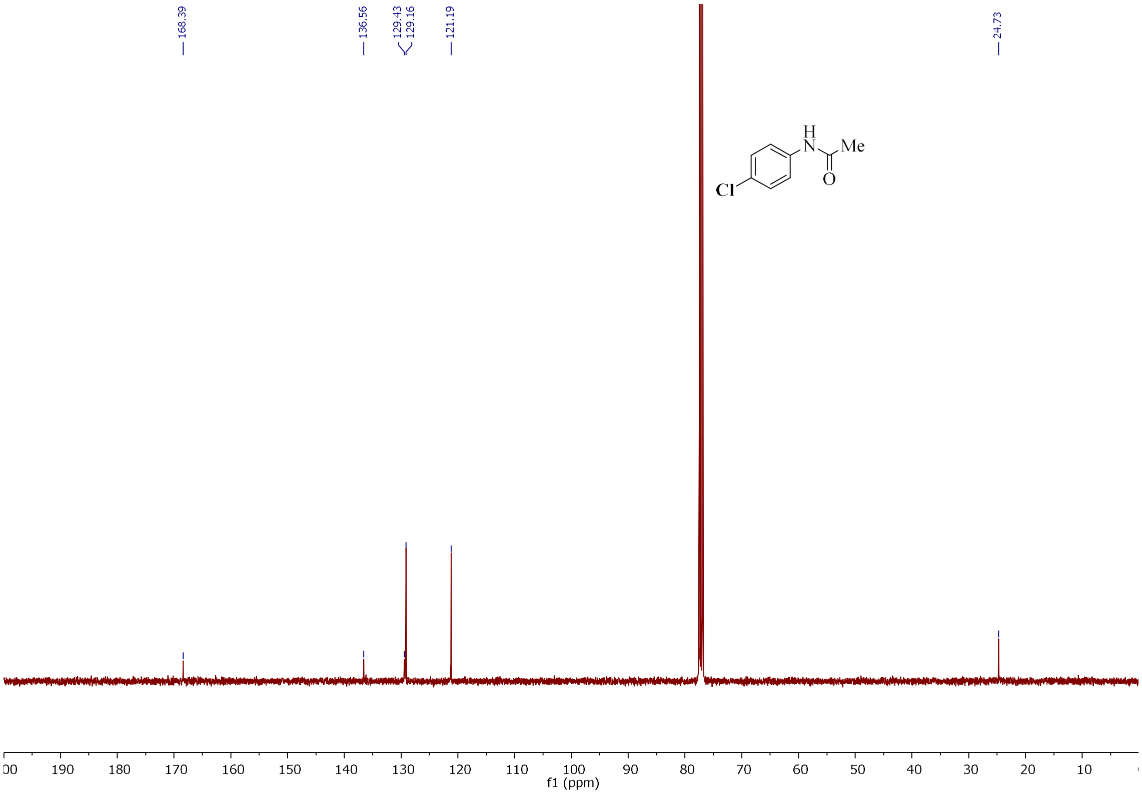

***N*-(4-chlorophenyl)propionamide: (21)**

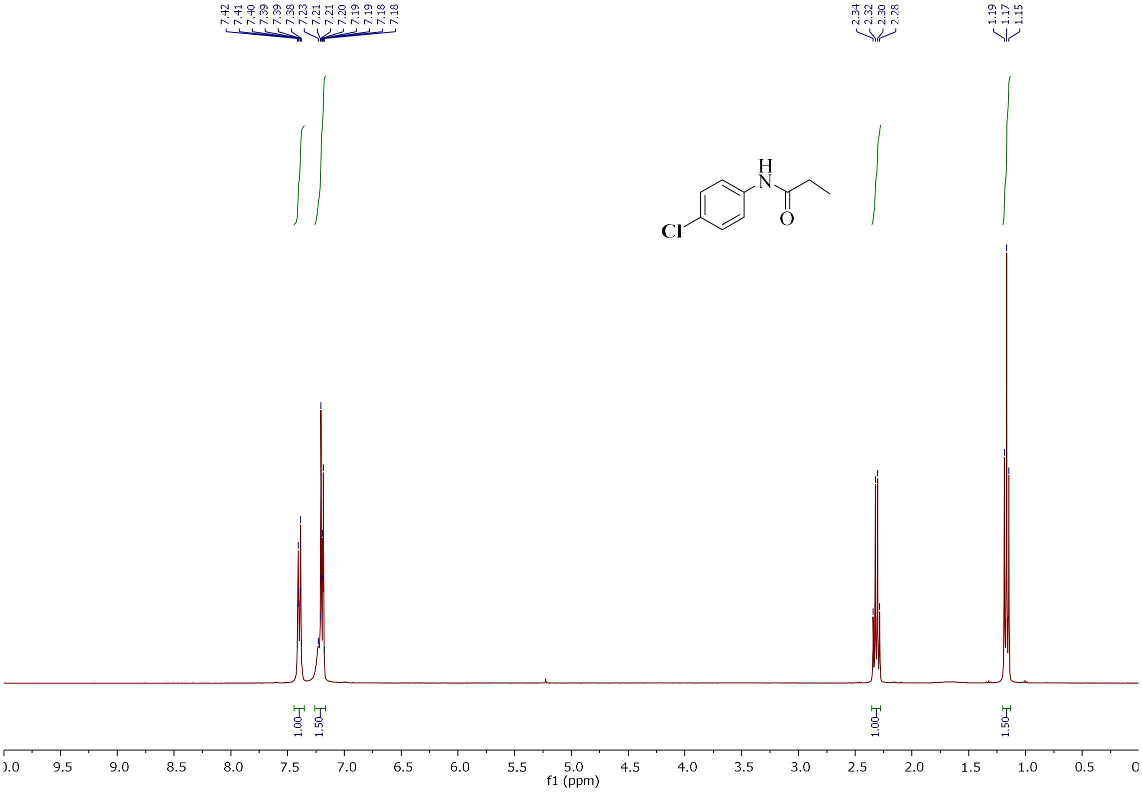

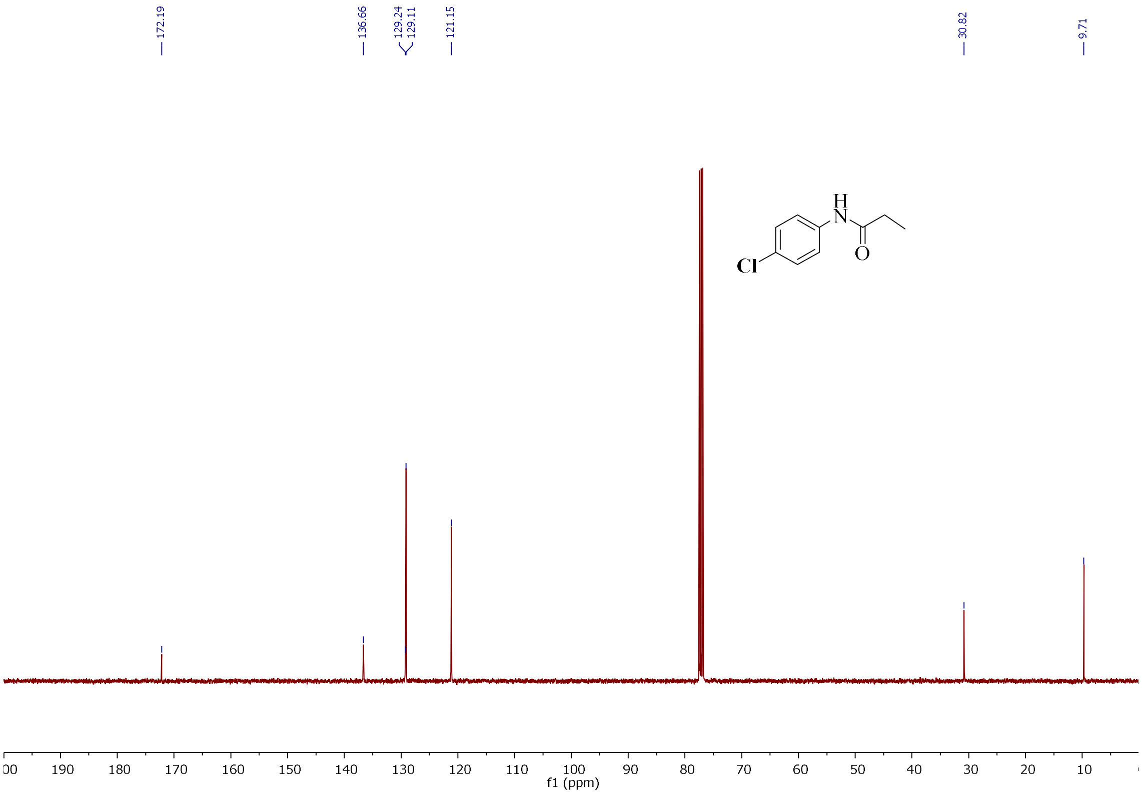

***N*-(4-chlorophenyl)hexanamide: (22)**

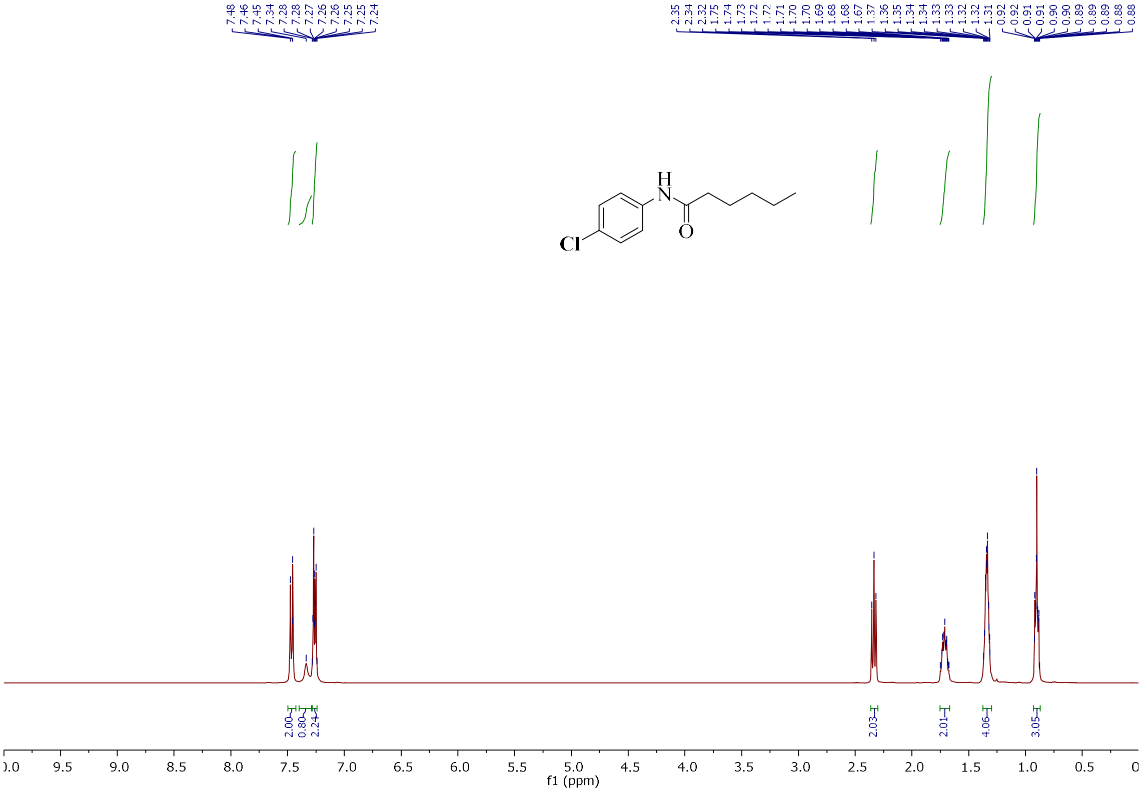

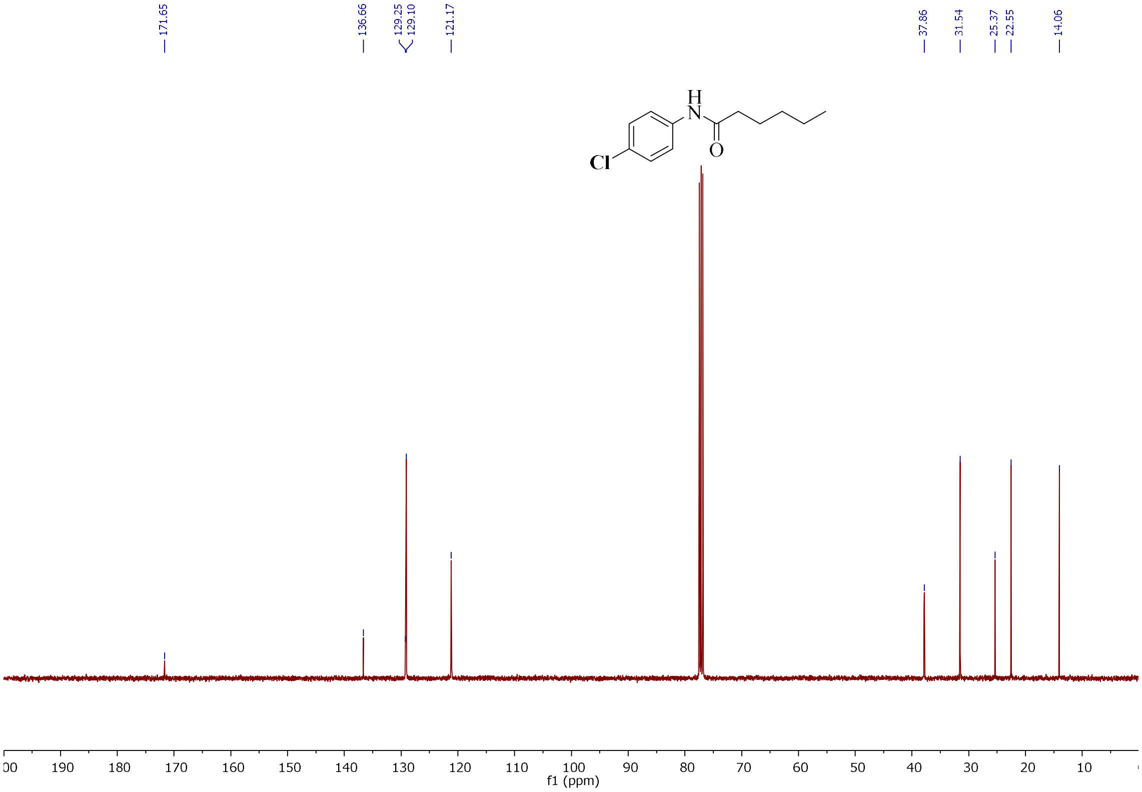

N-(4-chlorophenyl)-2-phenylacetamide: (23)

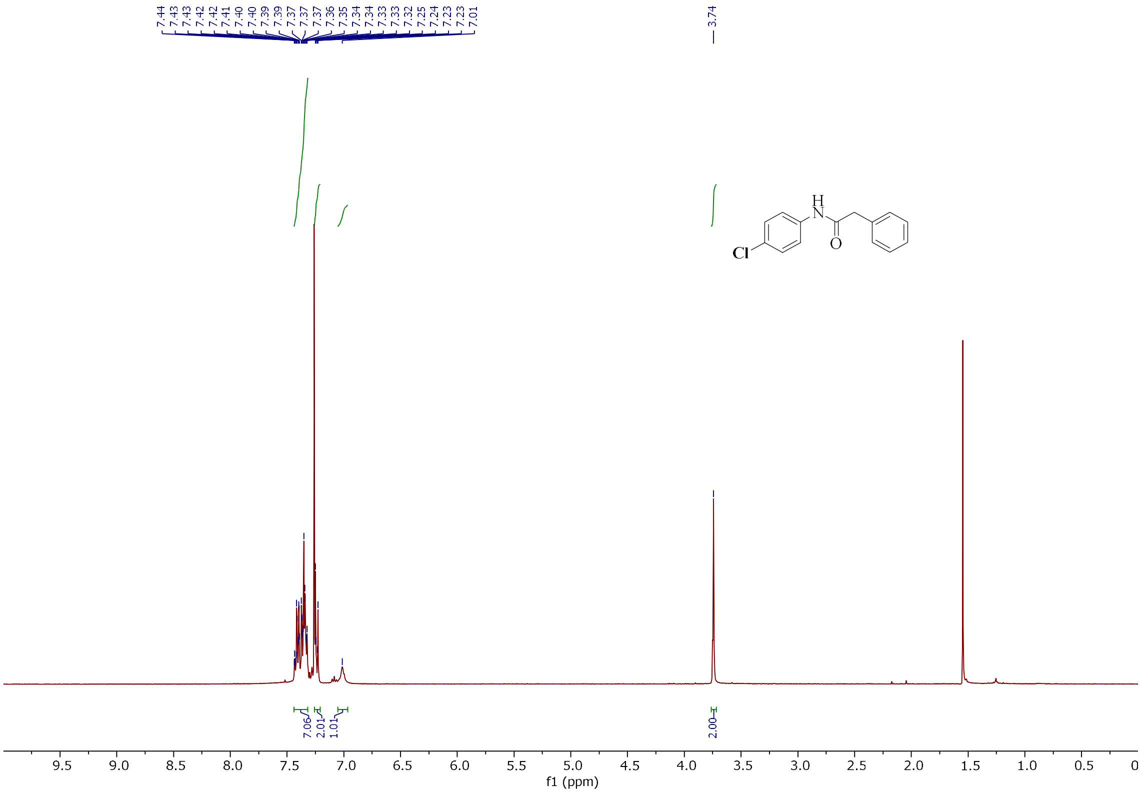

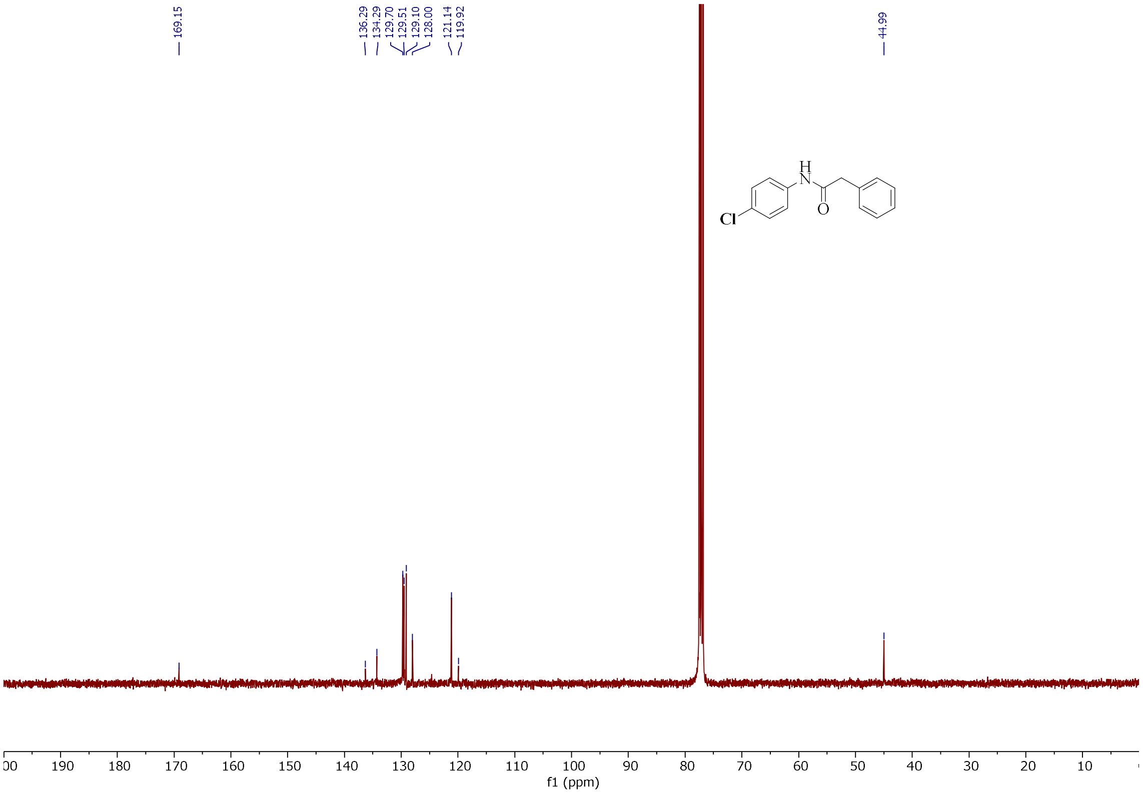

2-chloro-*N*-(4-chlorophenyl)acetamide: (24)

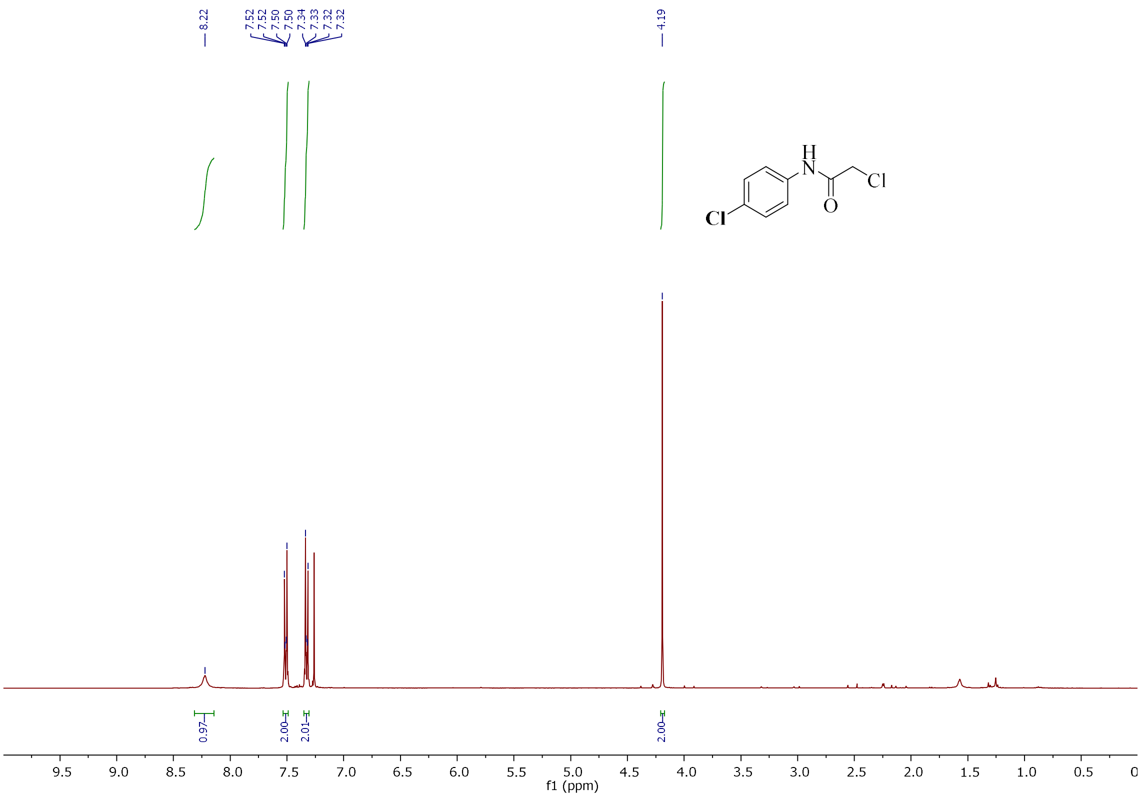

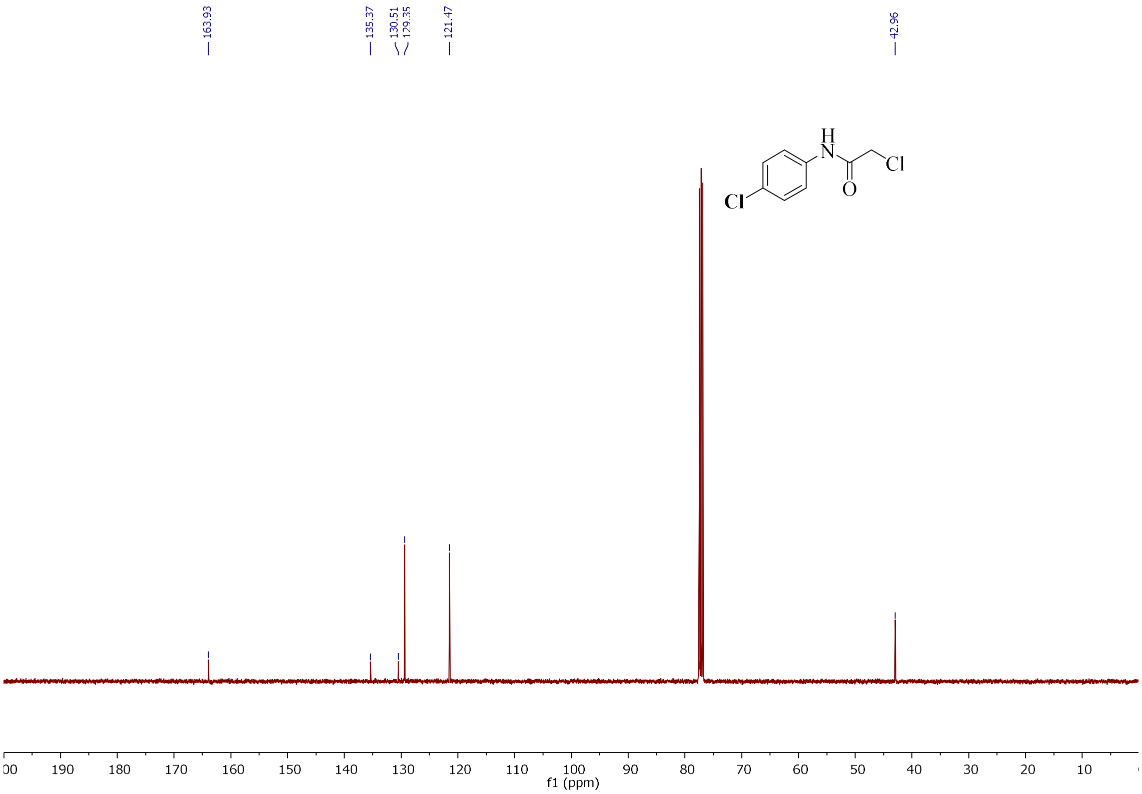

***N*-(4-chlorophenyl)isobutyramide: (25)**

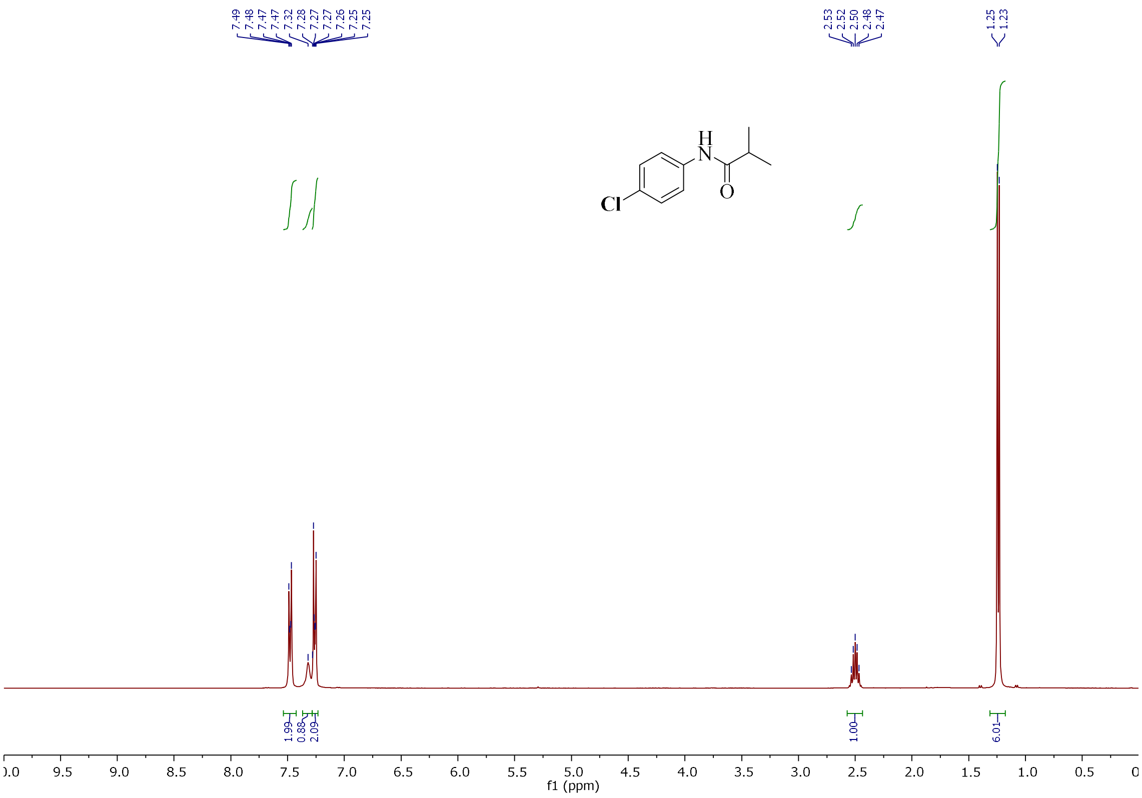

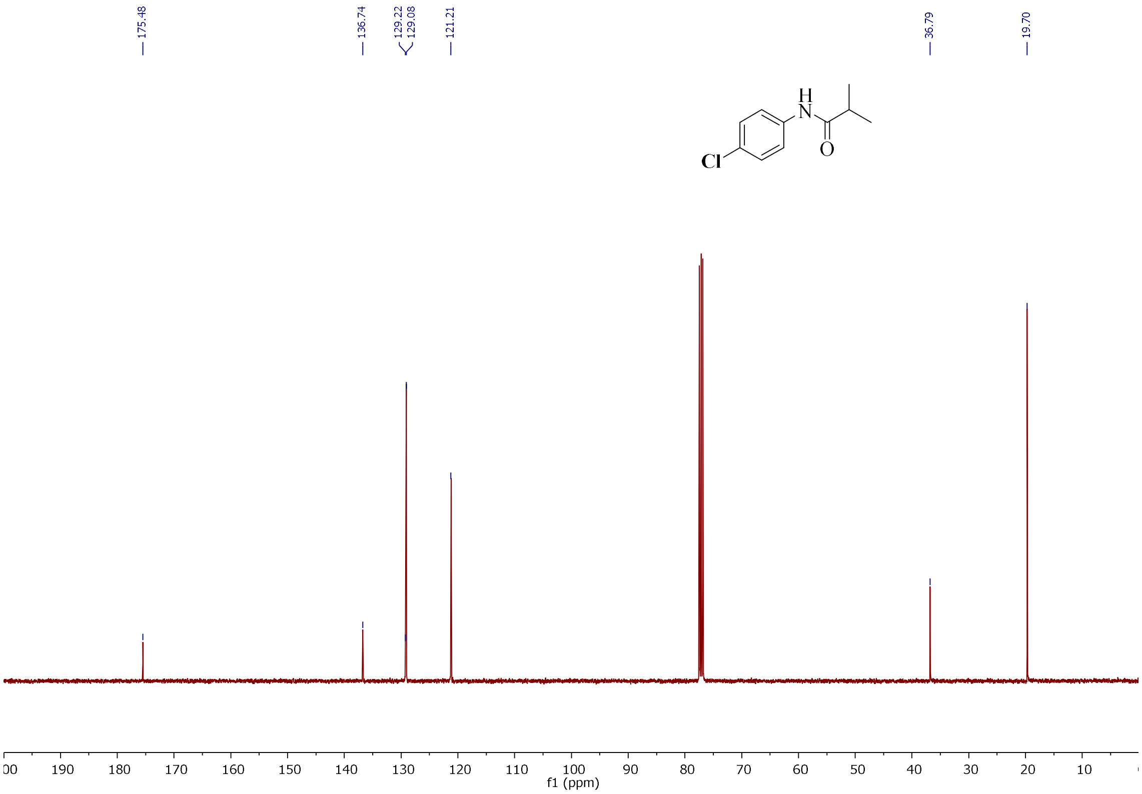

***N*-(4-chlorophenyl)pivalamide: (26)**

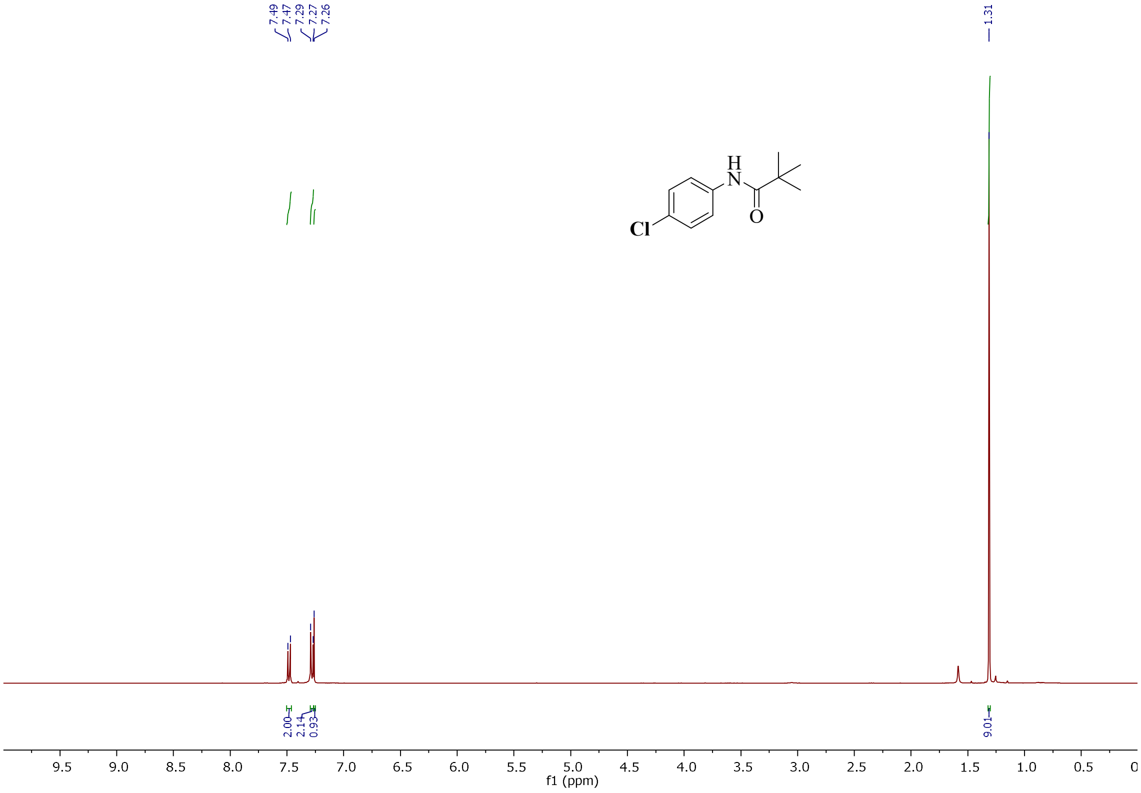

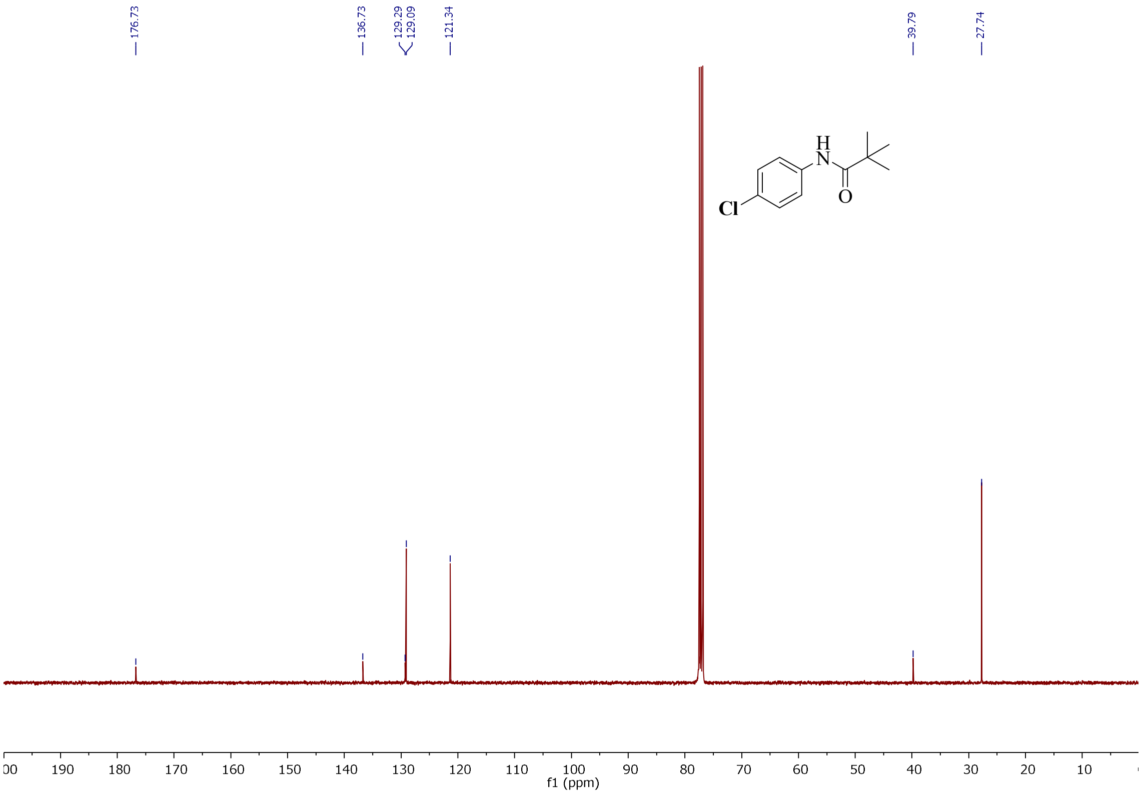

***N*-(4-chlorophenyl)adamantane-1-carboxamide: (27)**

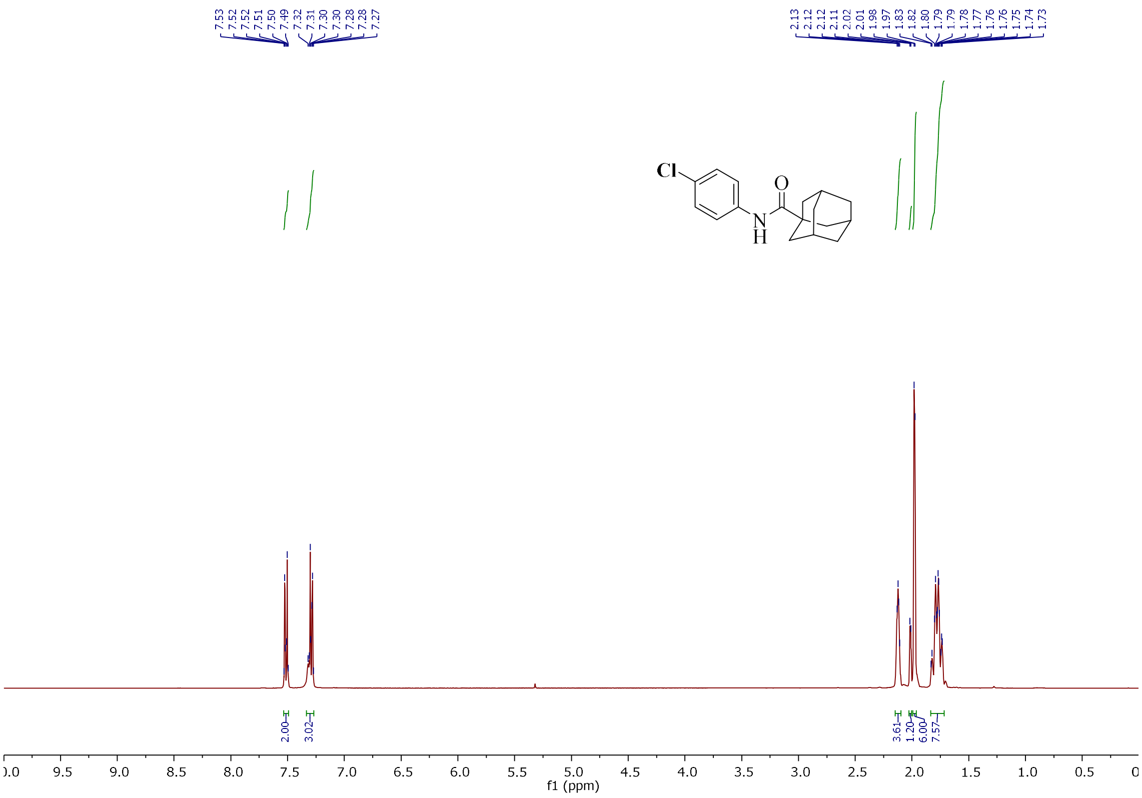

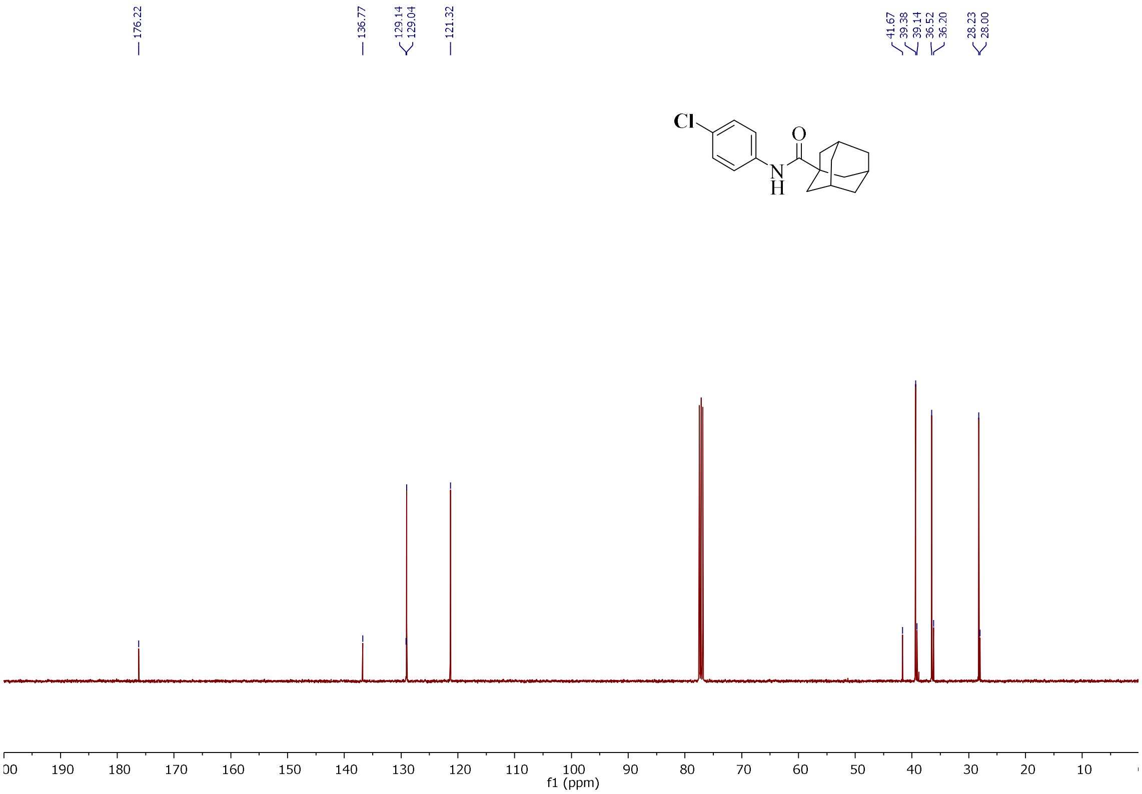

***N*-(4-chlorophenyl)cyclopropanecarboxamide: (28)**

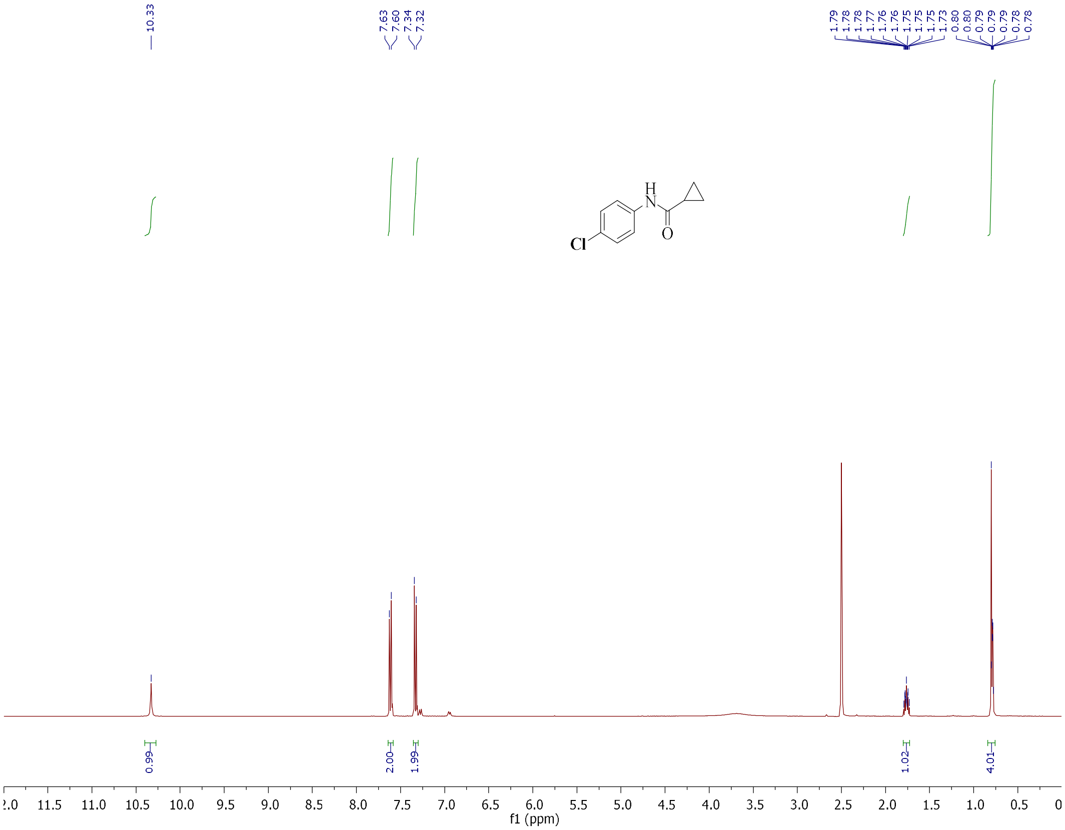

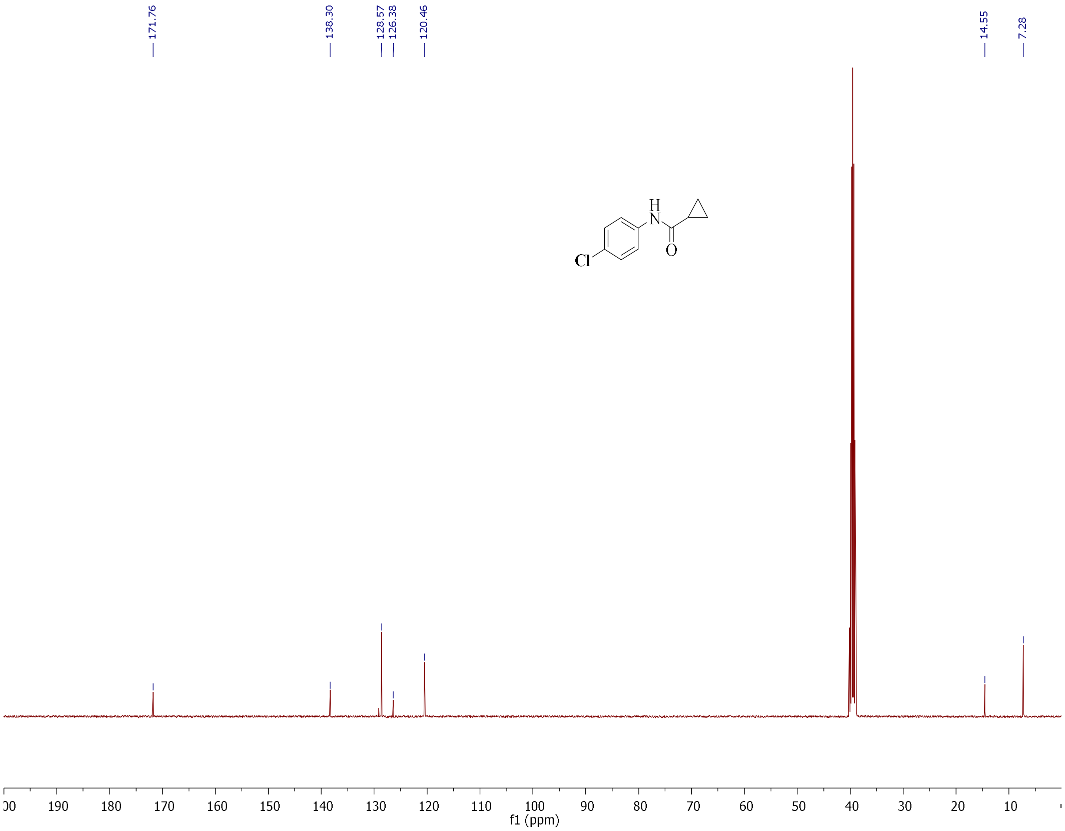

***N*-(4-chlorophenyl)cyclobutanecarboxamide: (29)**

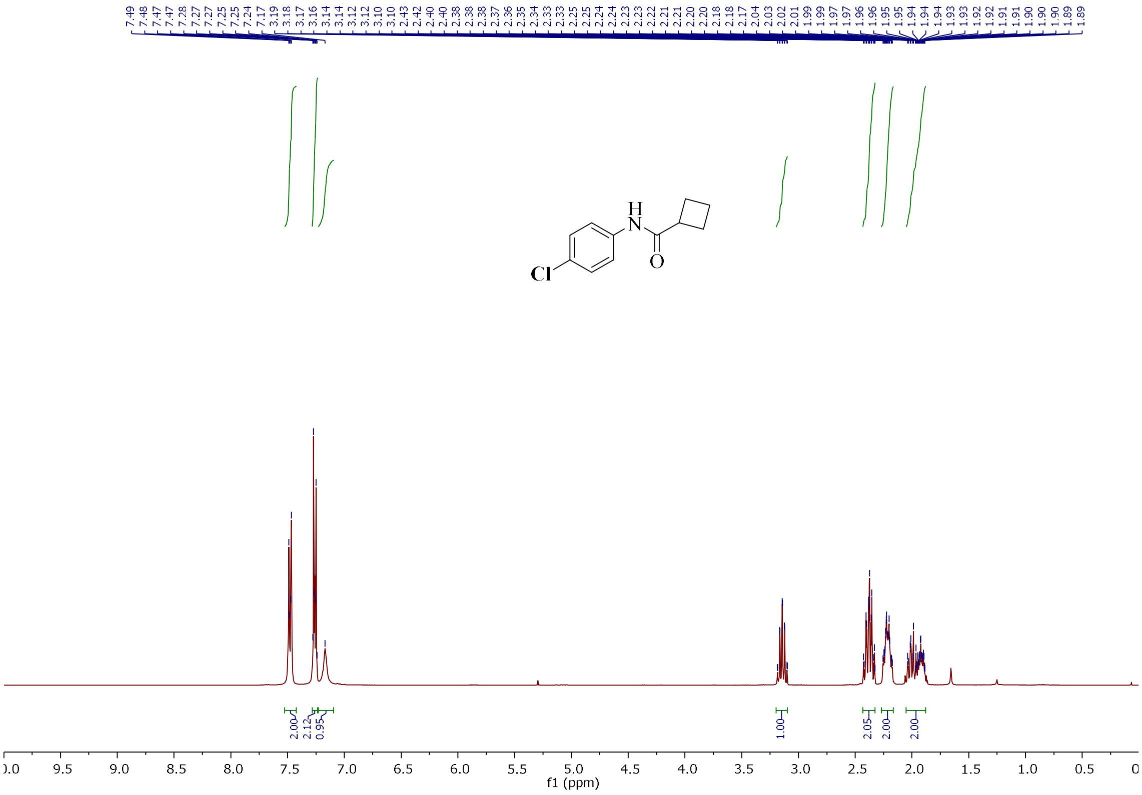

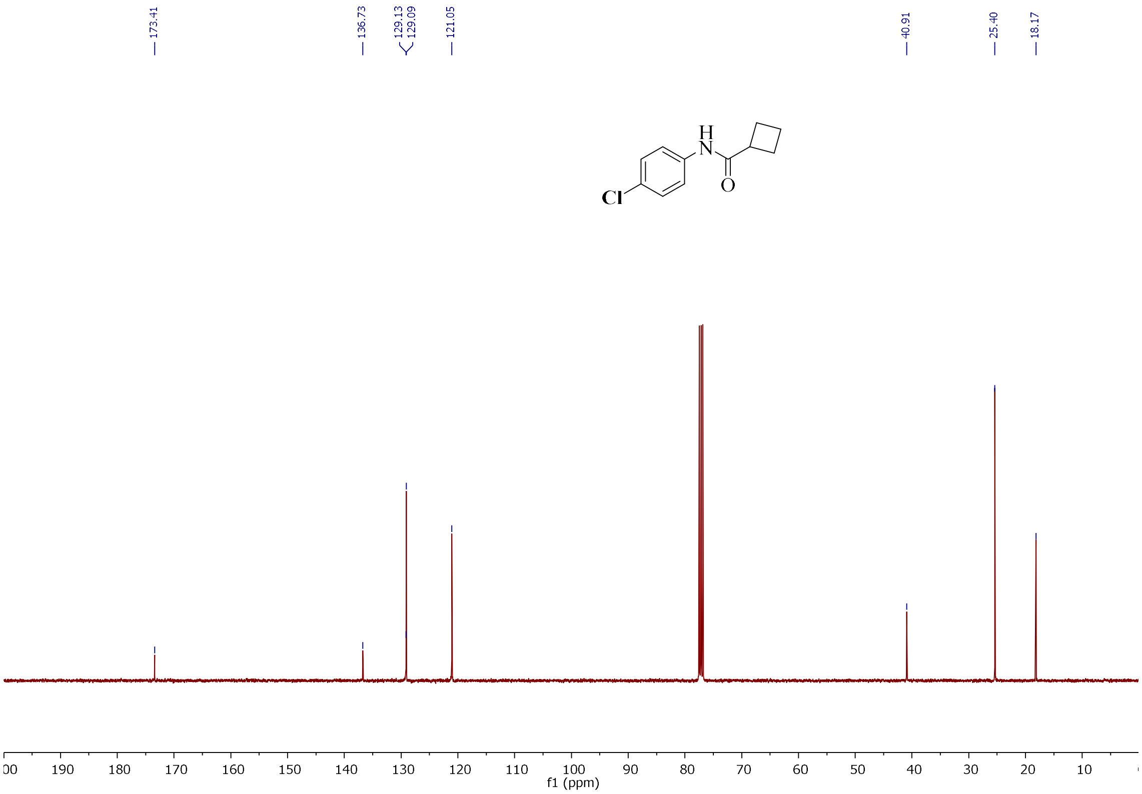

***N*-(4-chlorophenyl)cyclopentanecarboxamide: (30)**

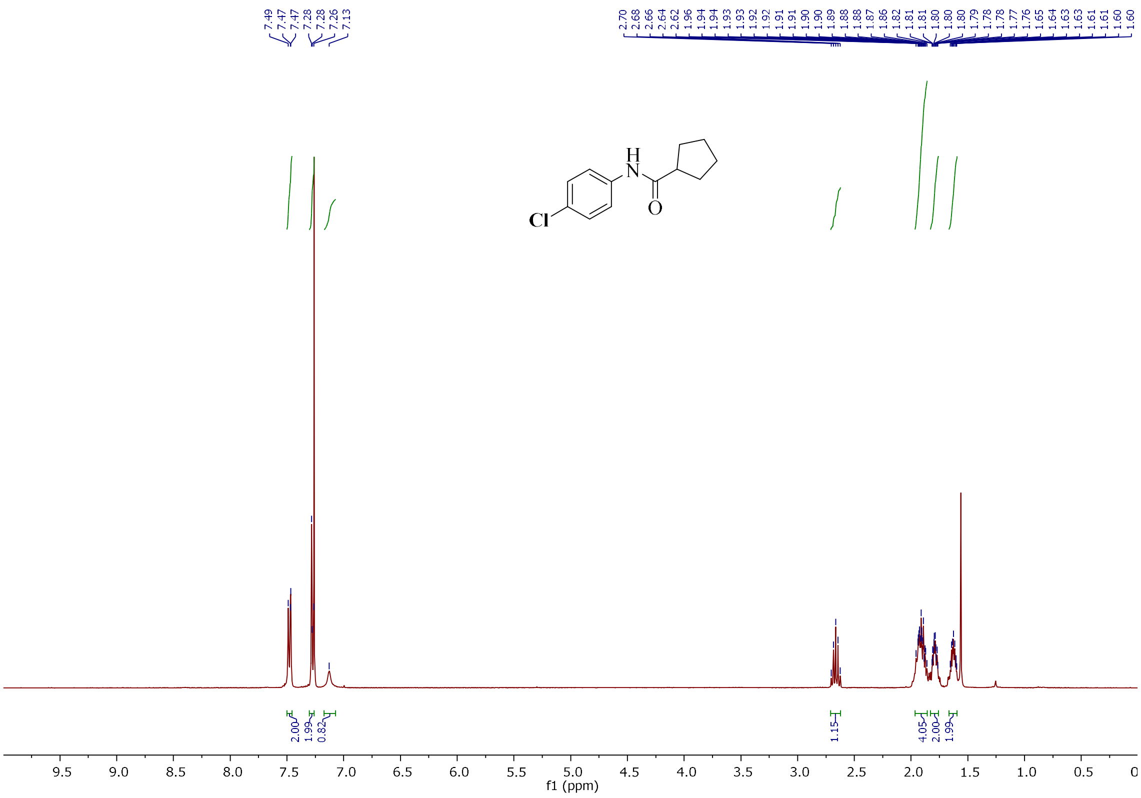

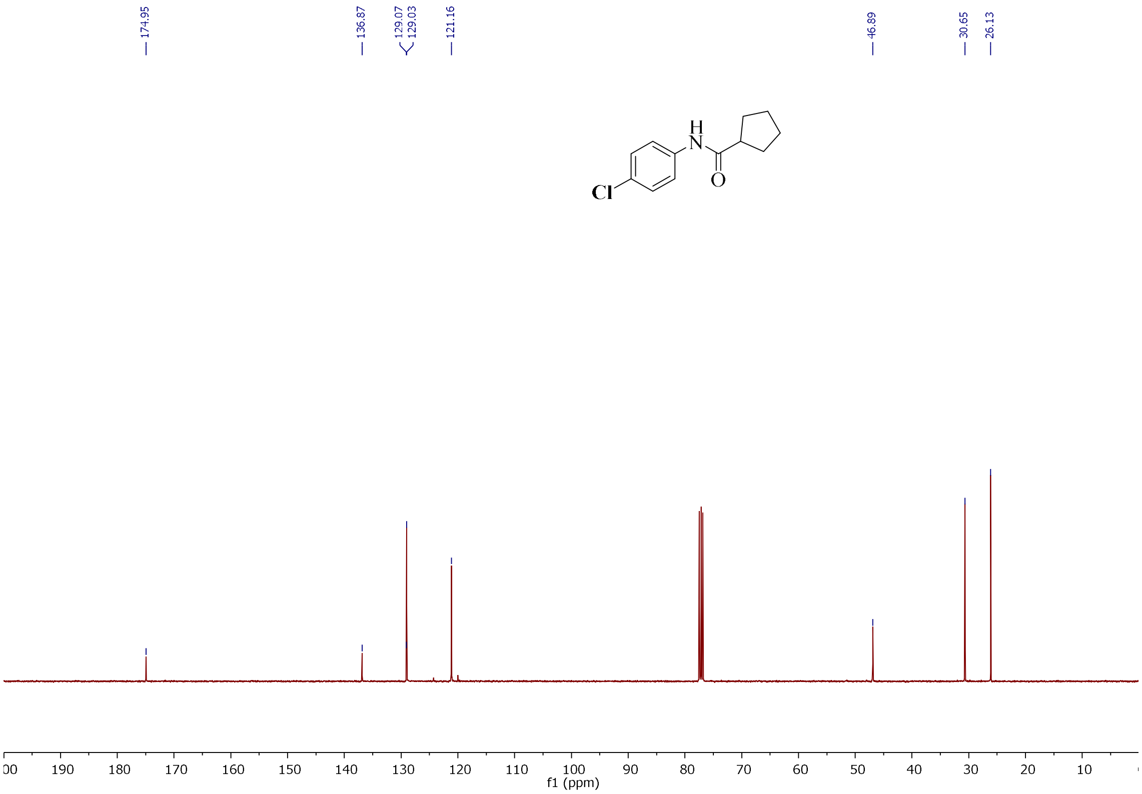

_N_-(4-chlorophenyl)cycloheptanecarboxamide: (31)

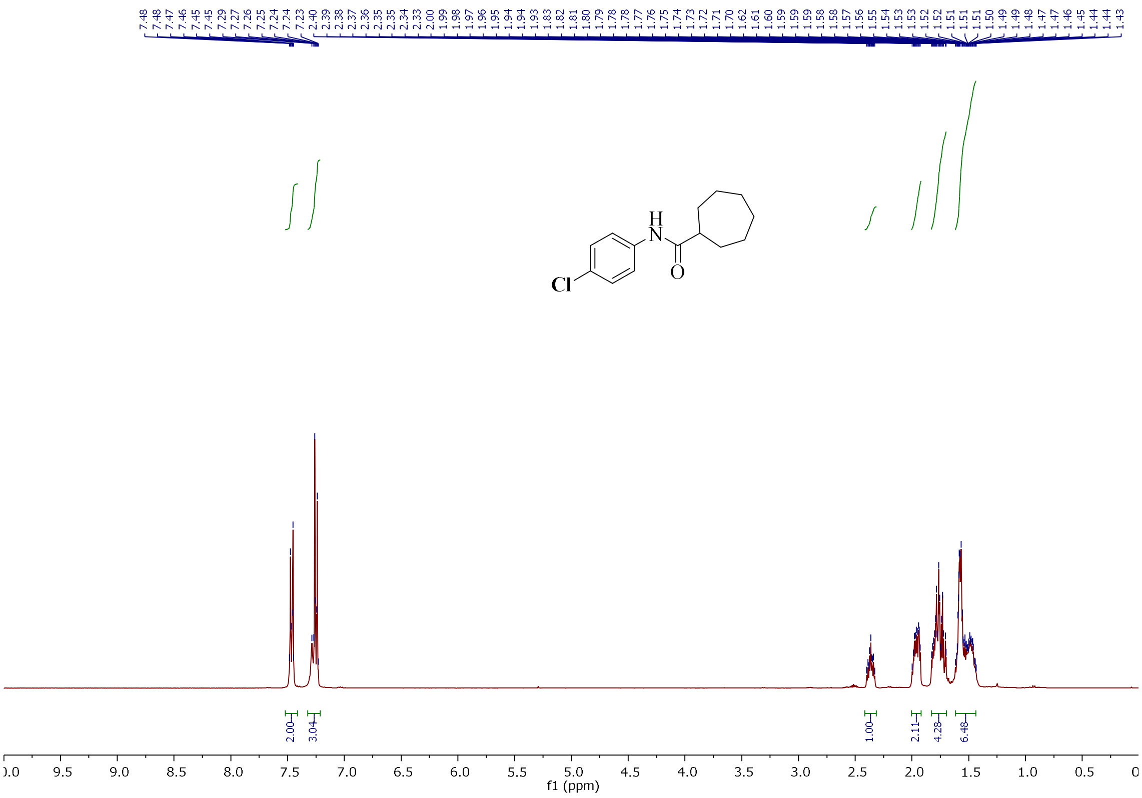

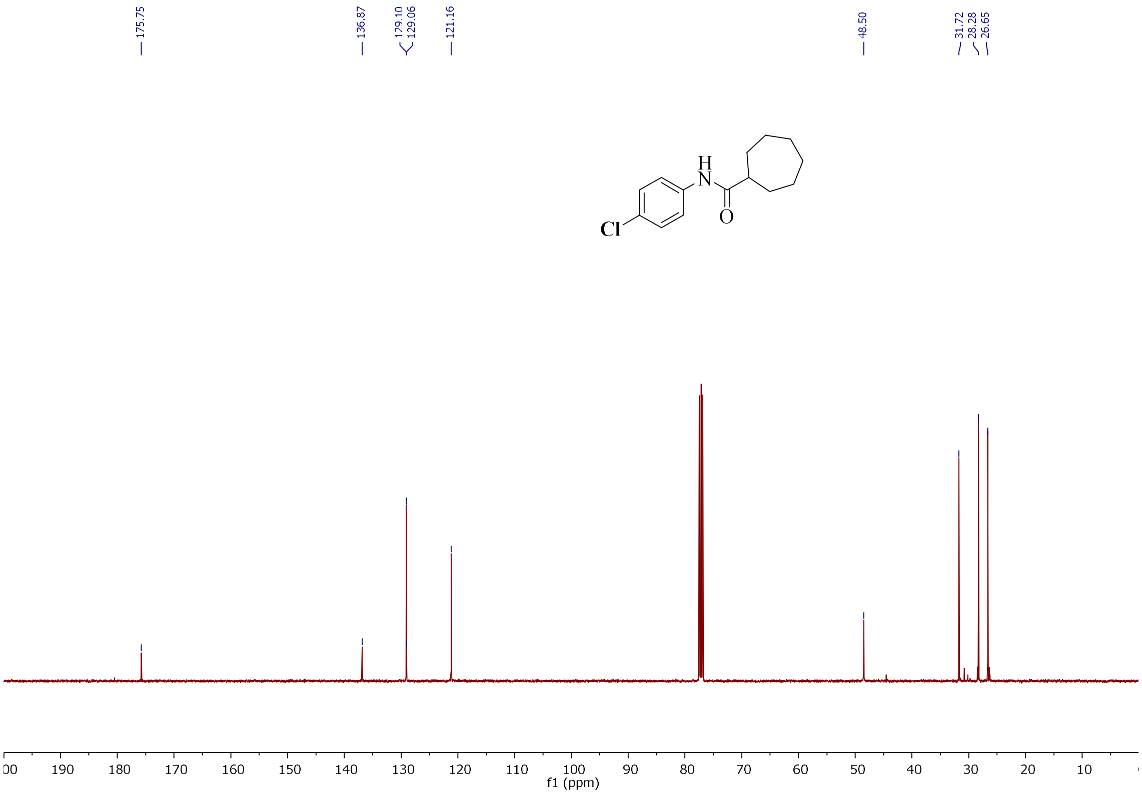

***N*-(4-chlorophenyl)benzamide: (32)**

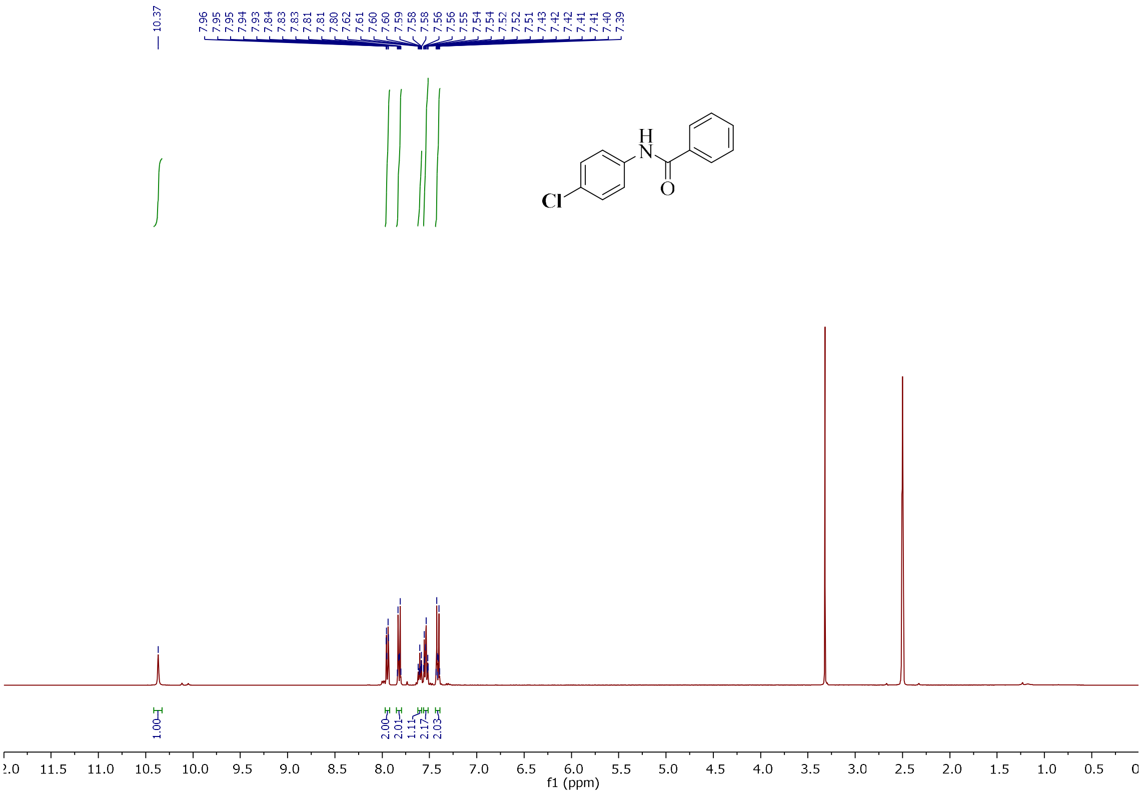

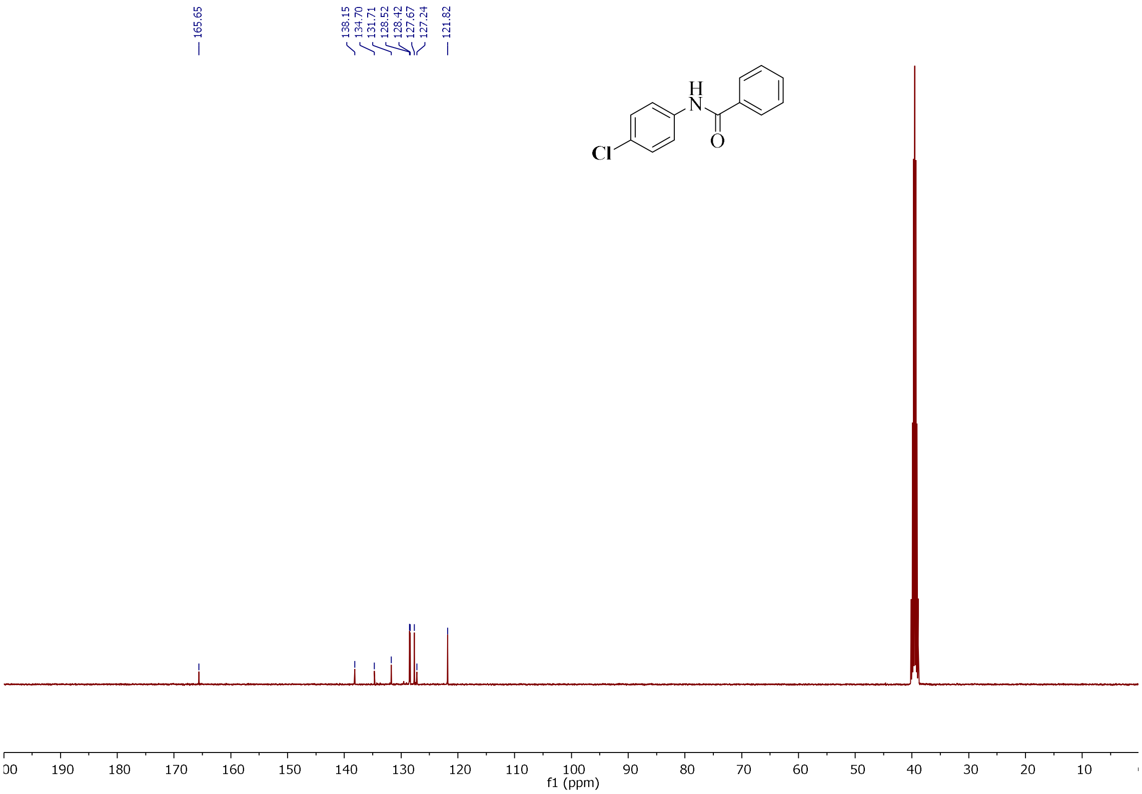

***N*-(4-chlorophenyl)-4-methoxybenzamide: (33)**

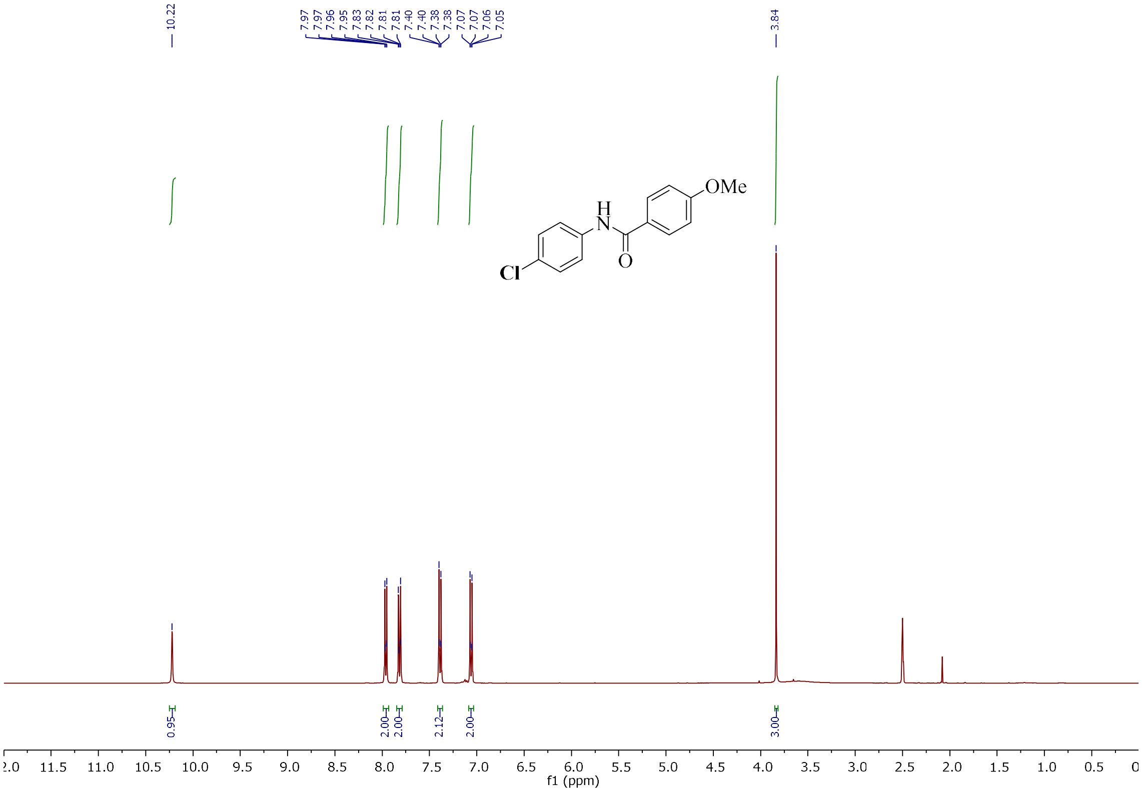

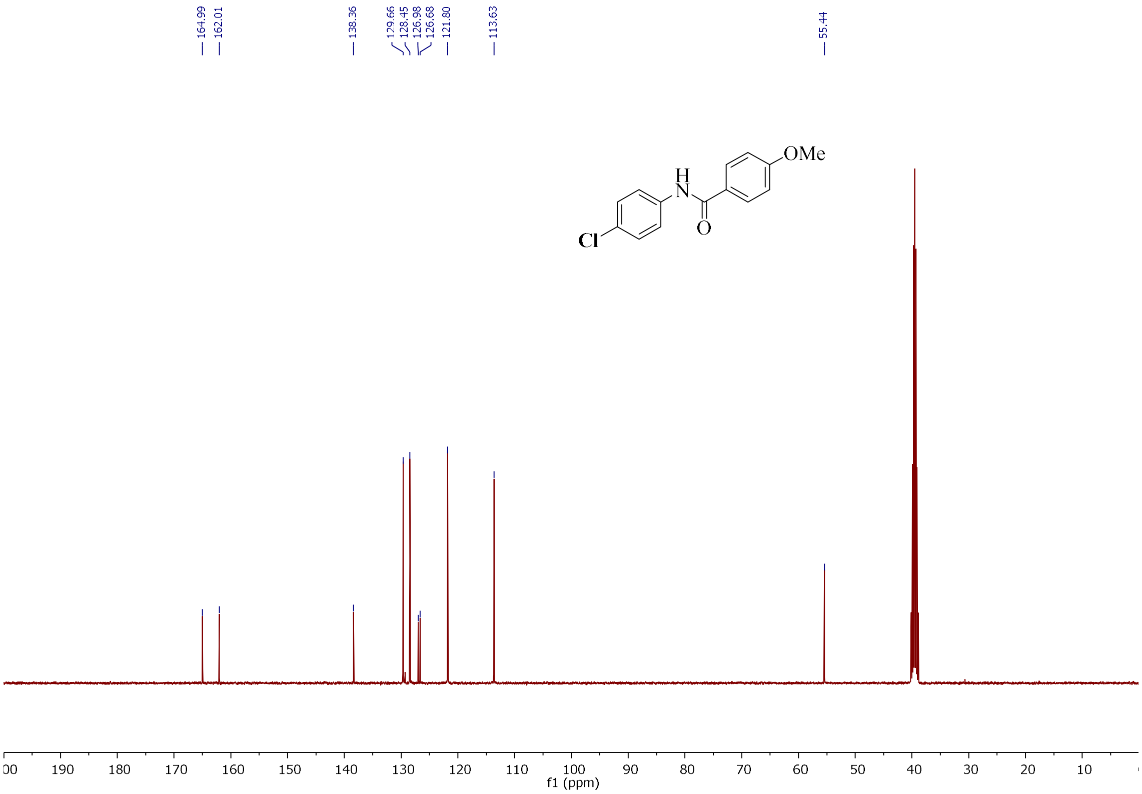

***N*-(4-chlorophenyl)-4-methylbenzamide: (34)**

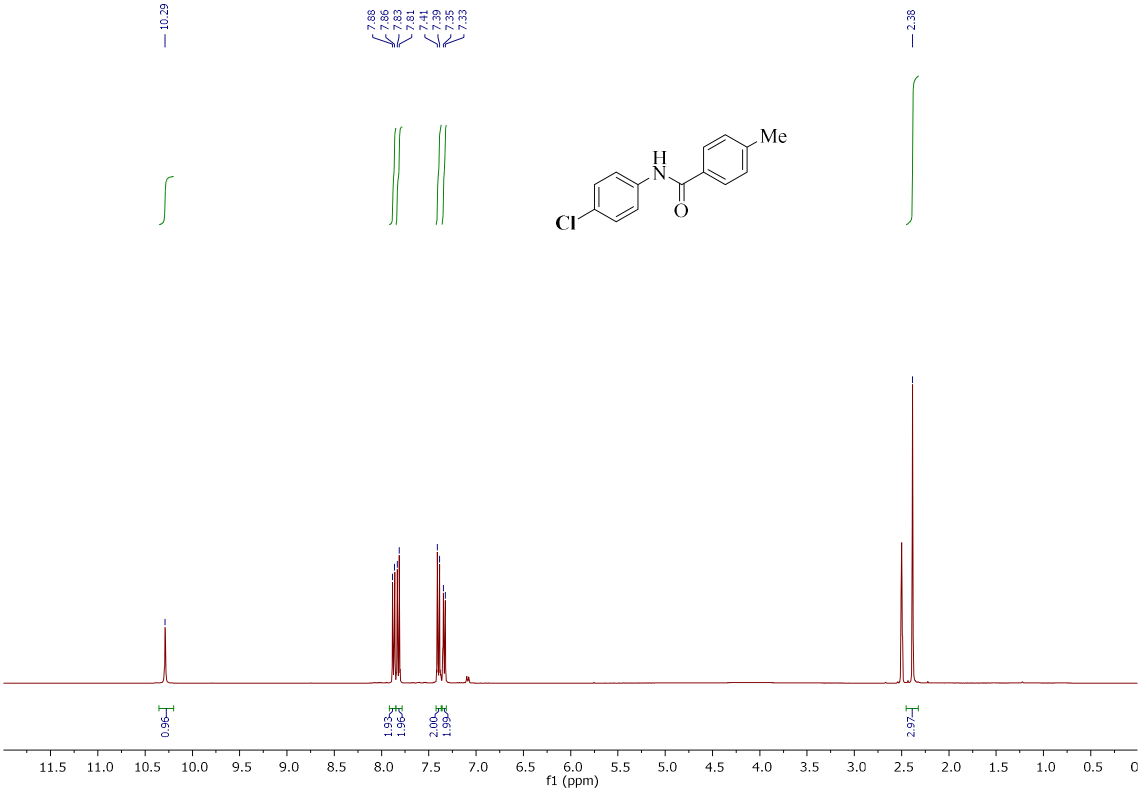

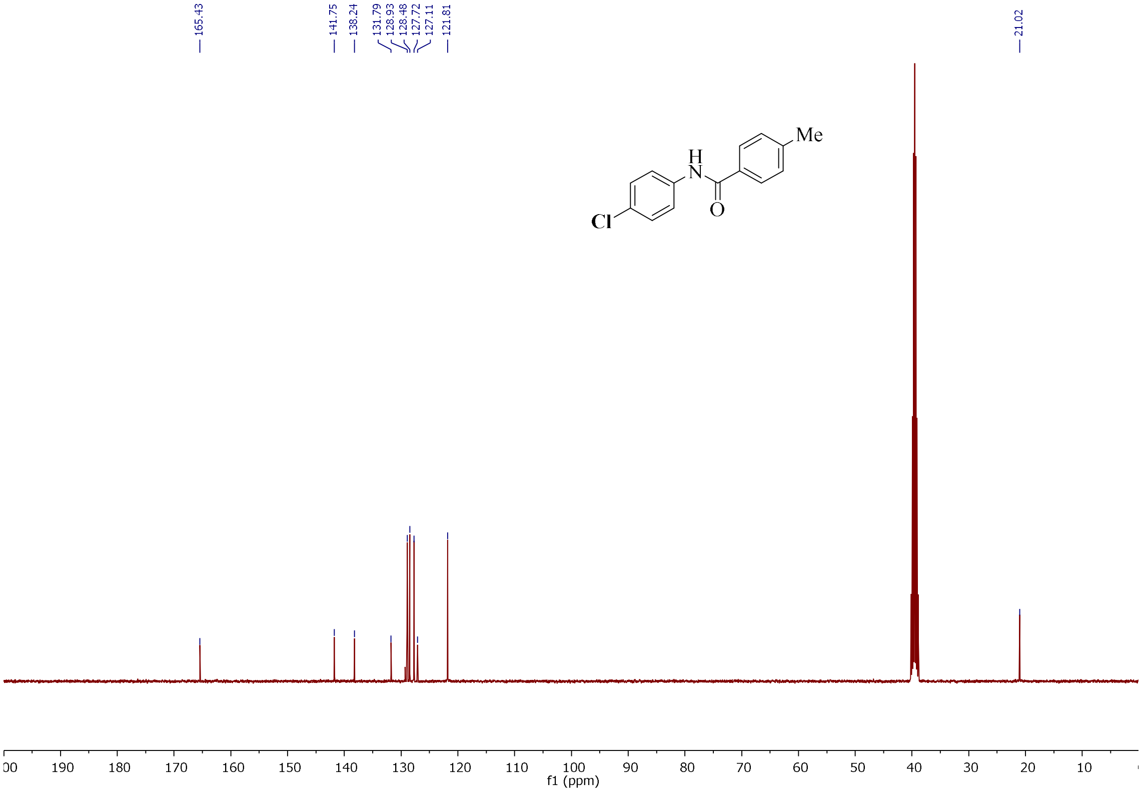

_N_-(4-chlorophenyl)-2,4-dimethoxybenzamide: (35)

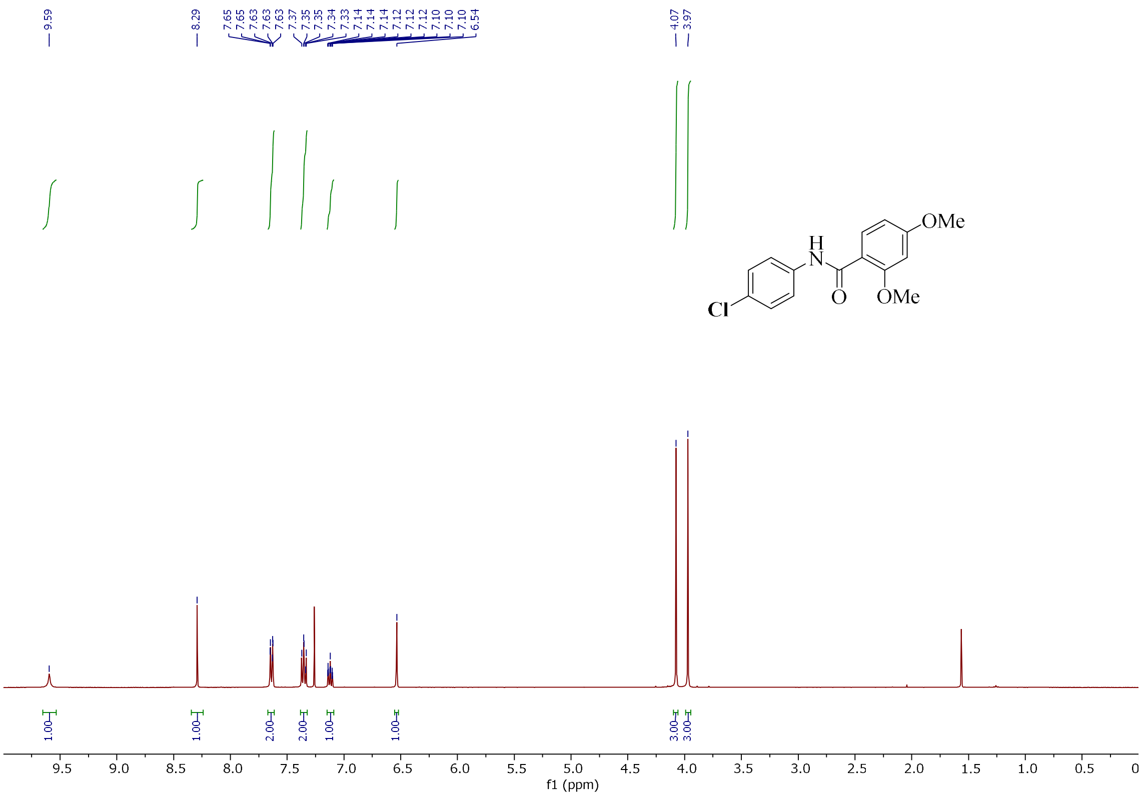

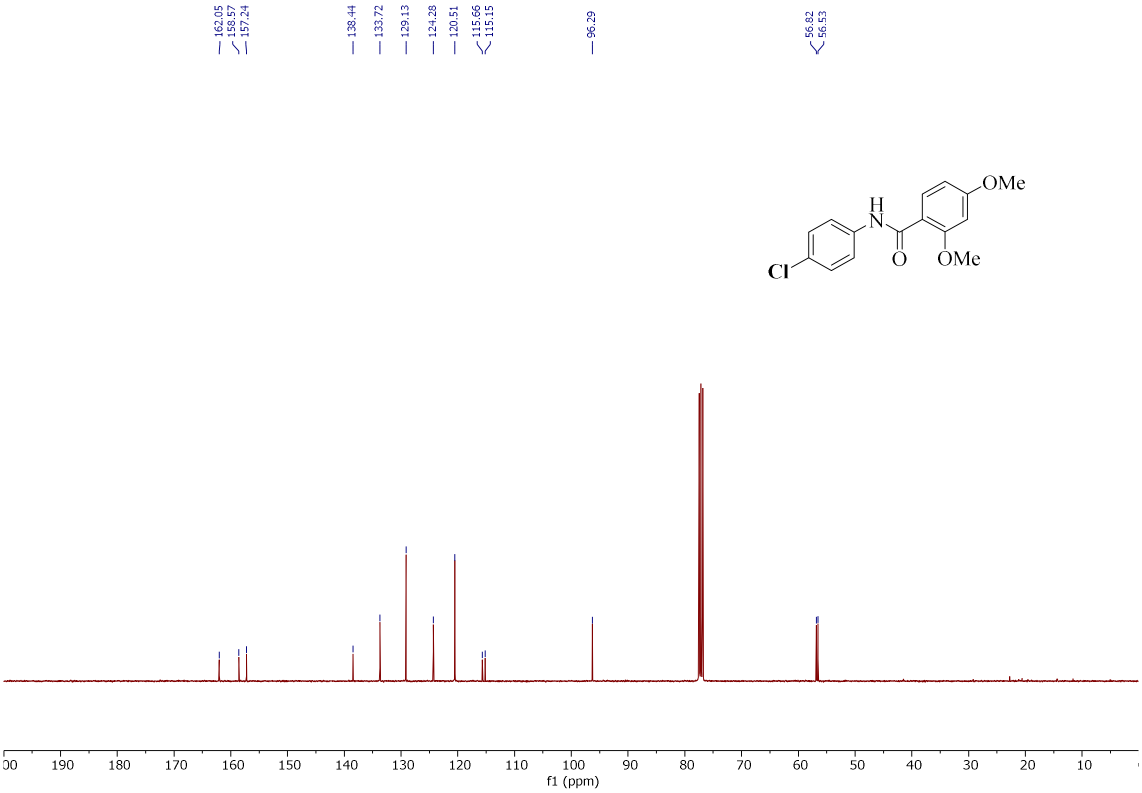

***N*-(4-chlorophenyl)-2-fluorobenzamide: (36)**

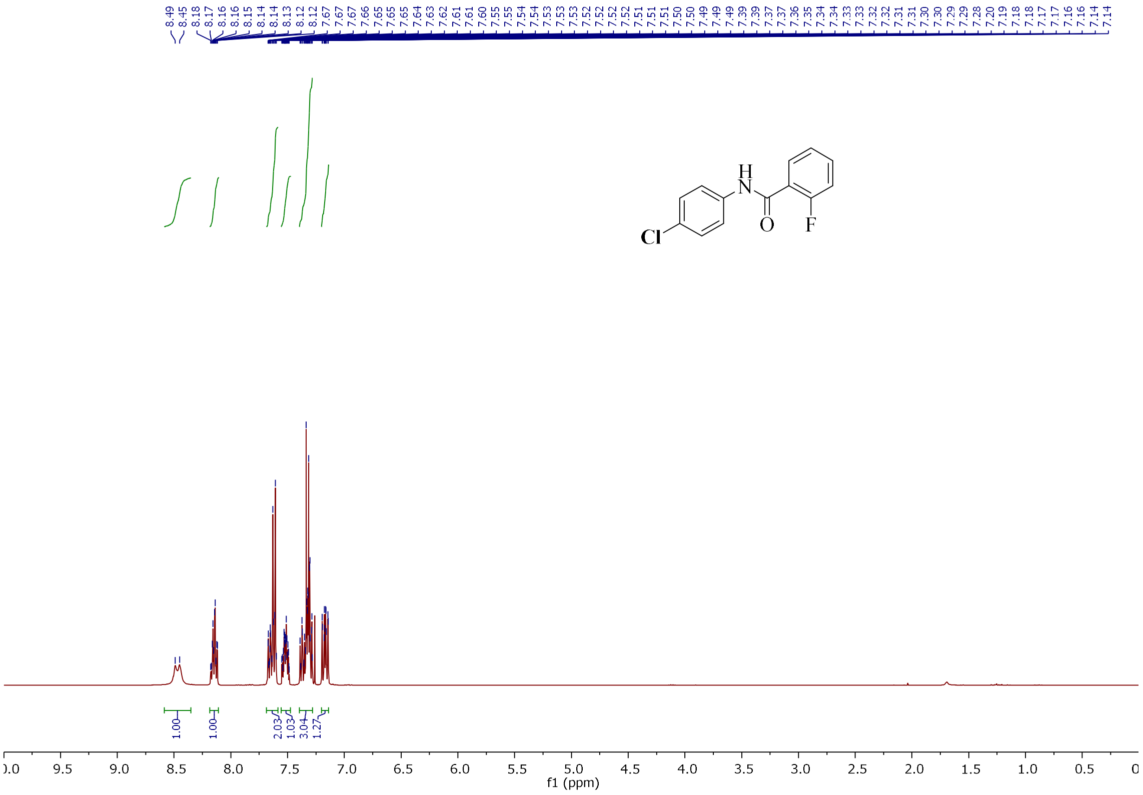

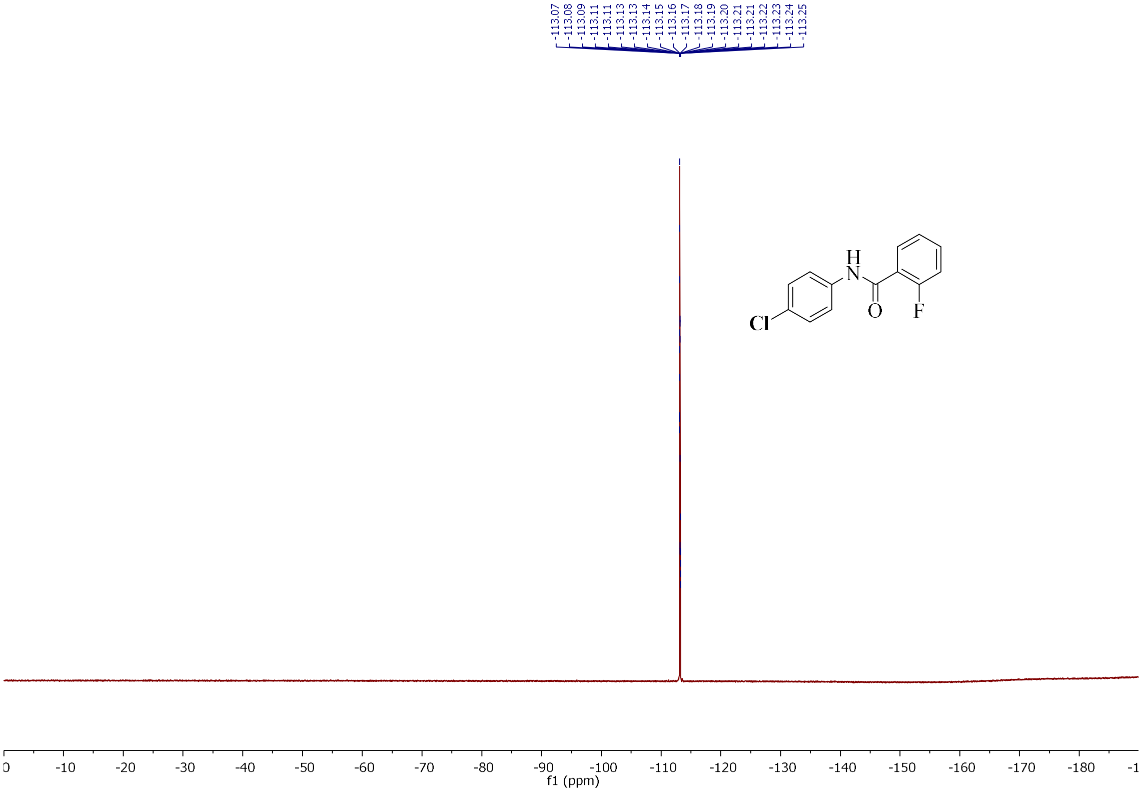

3-bromo-*N*-(4-chlorophenyl)benzamide: (37)

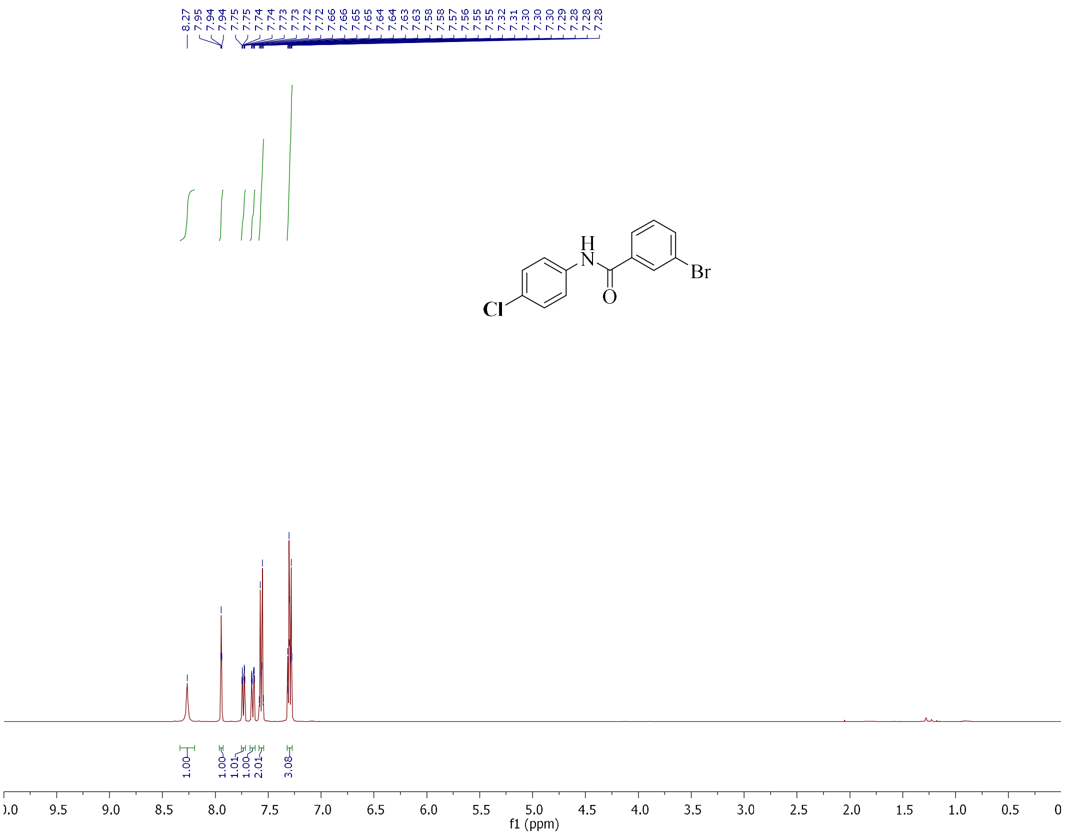

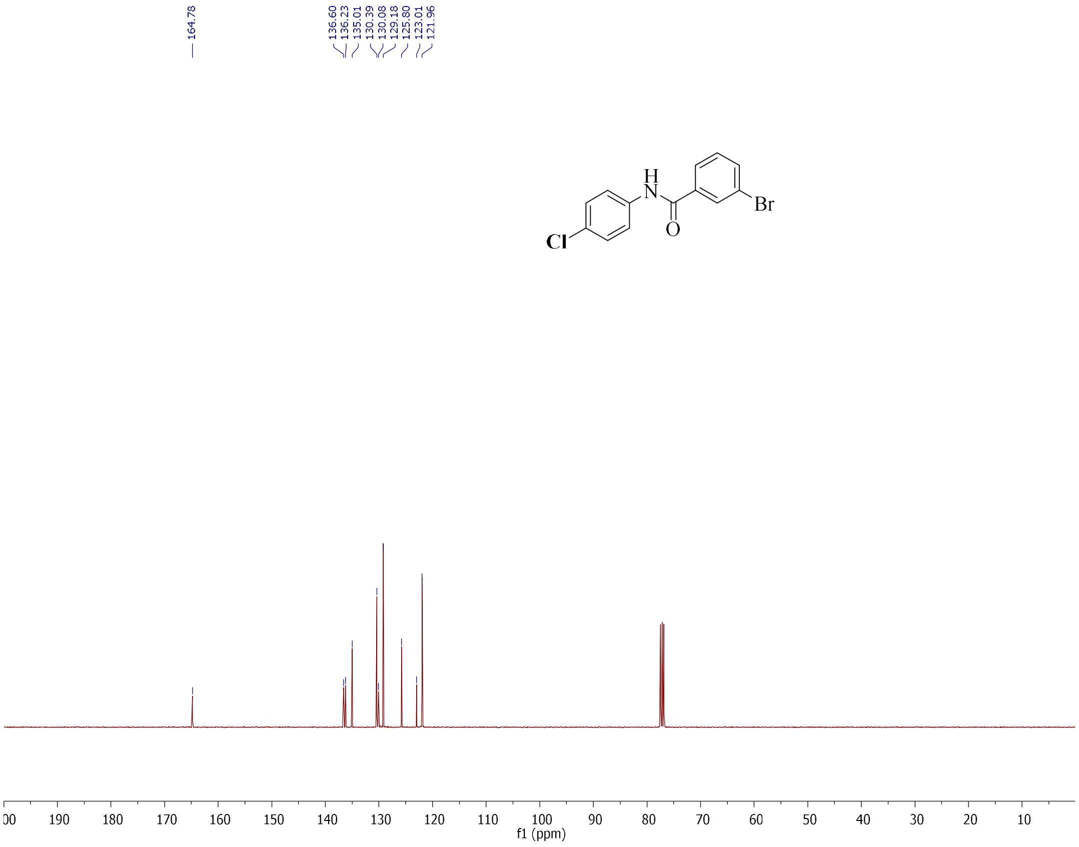

2,4-dichloro-N-(4-chlorophenyl)benzamide: (38)

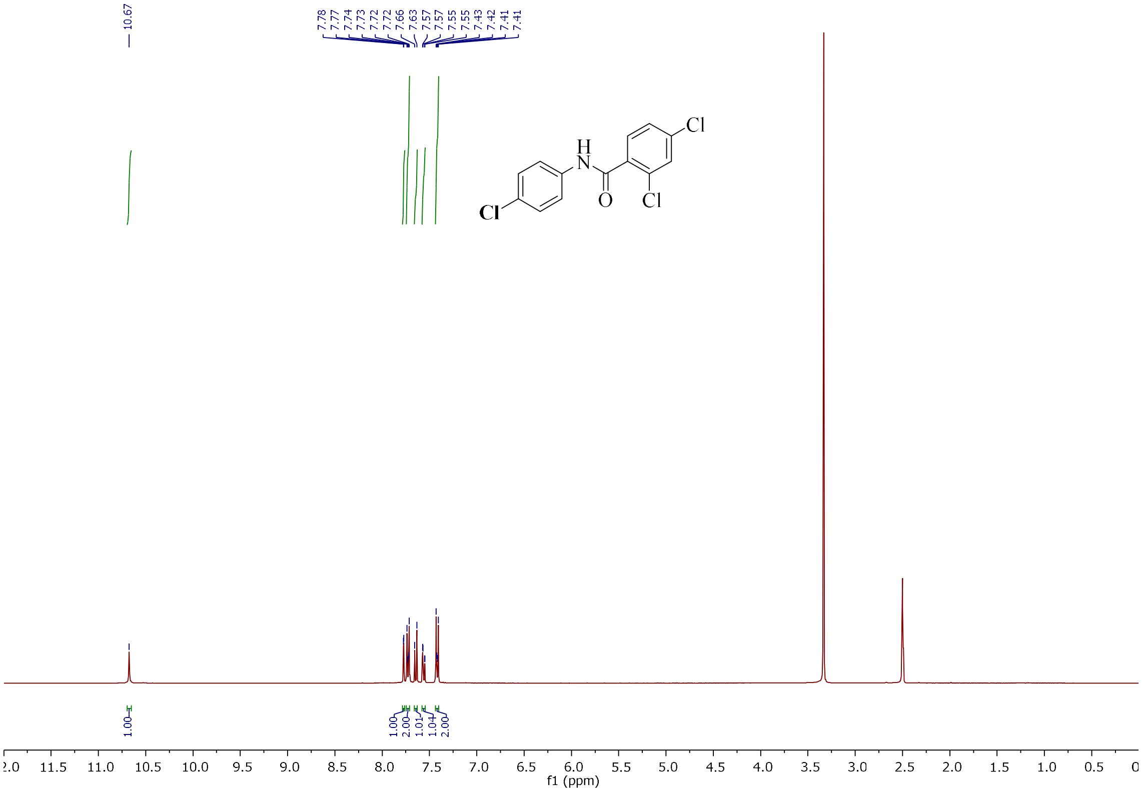

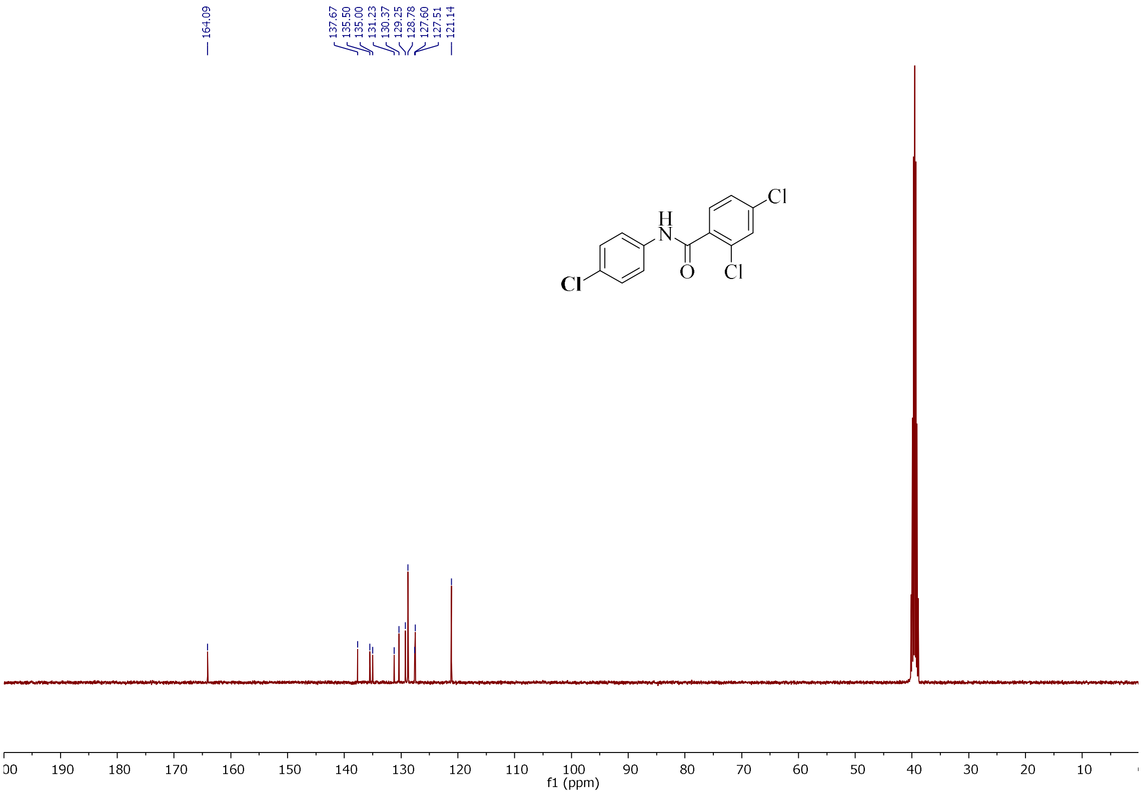

3,5-Dichloro-*N*-(4-chlorophenyl)benzamide: (39)

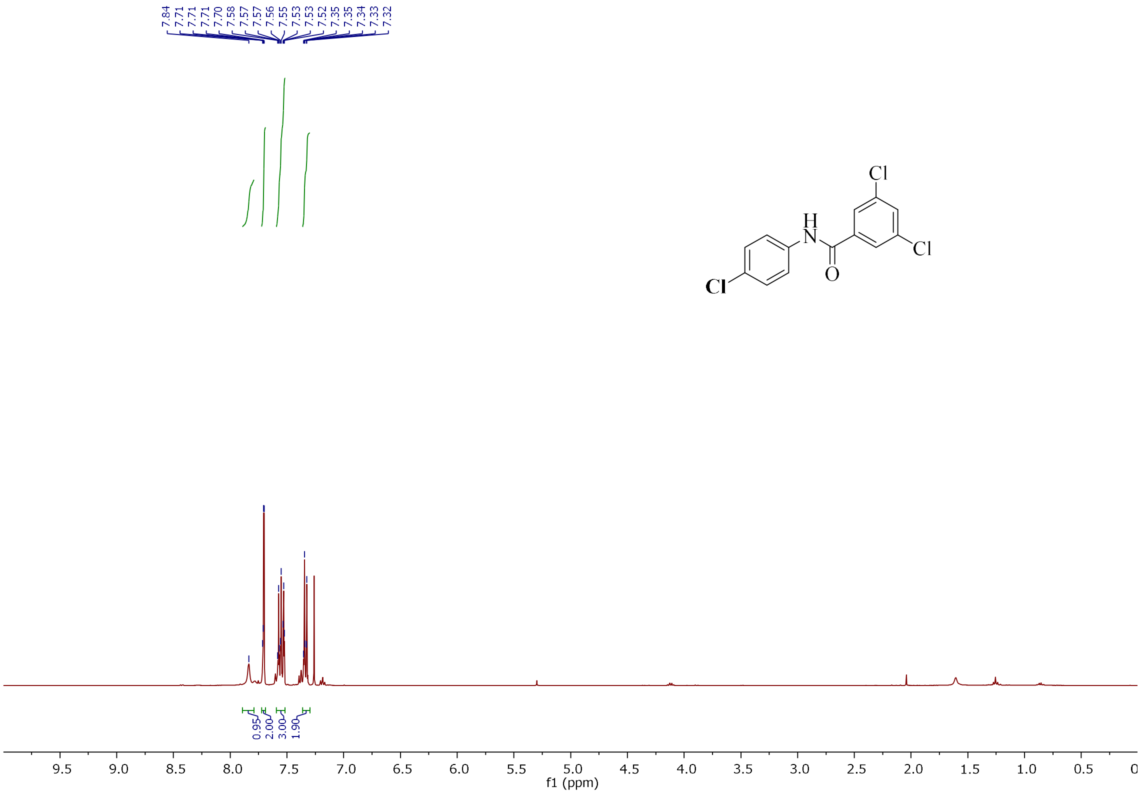

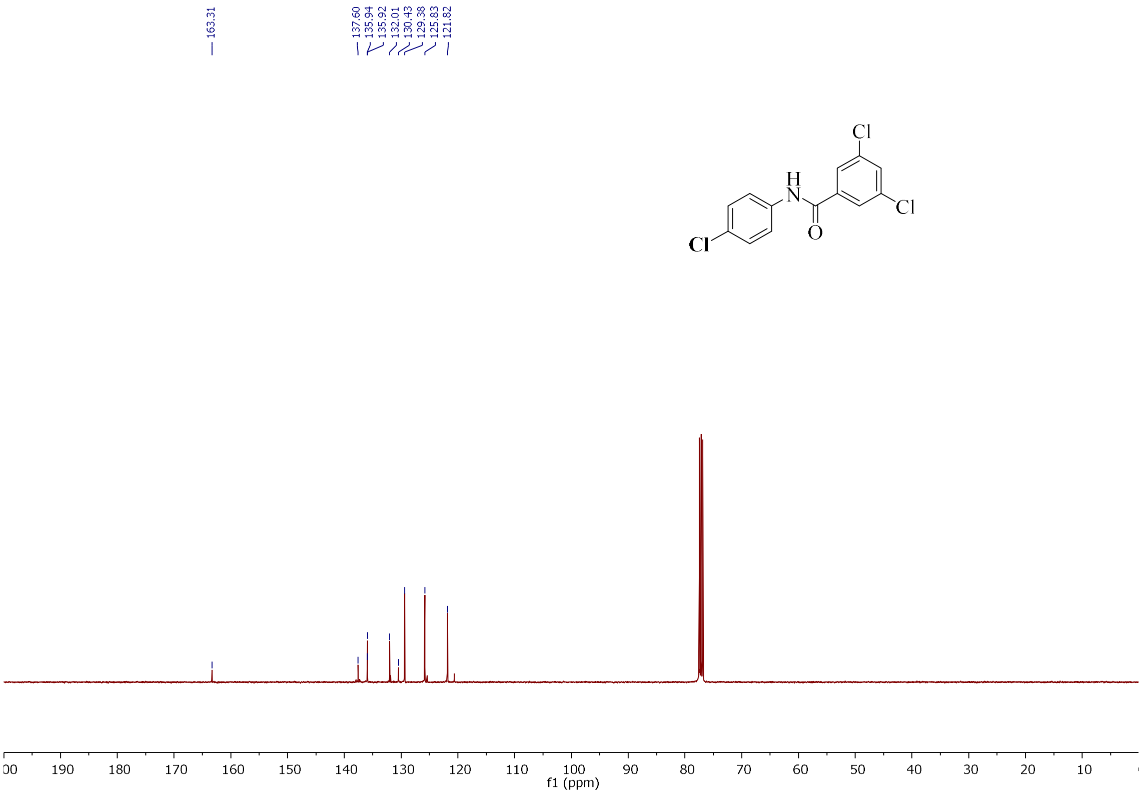

_N_-(4-chlorophenyl)-4-fluorobenzamide: (40)

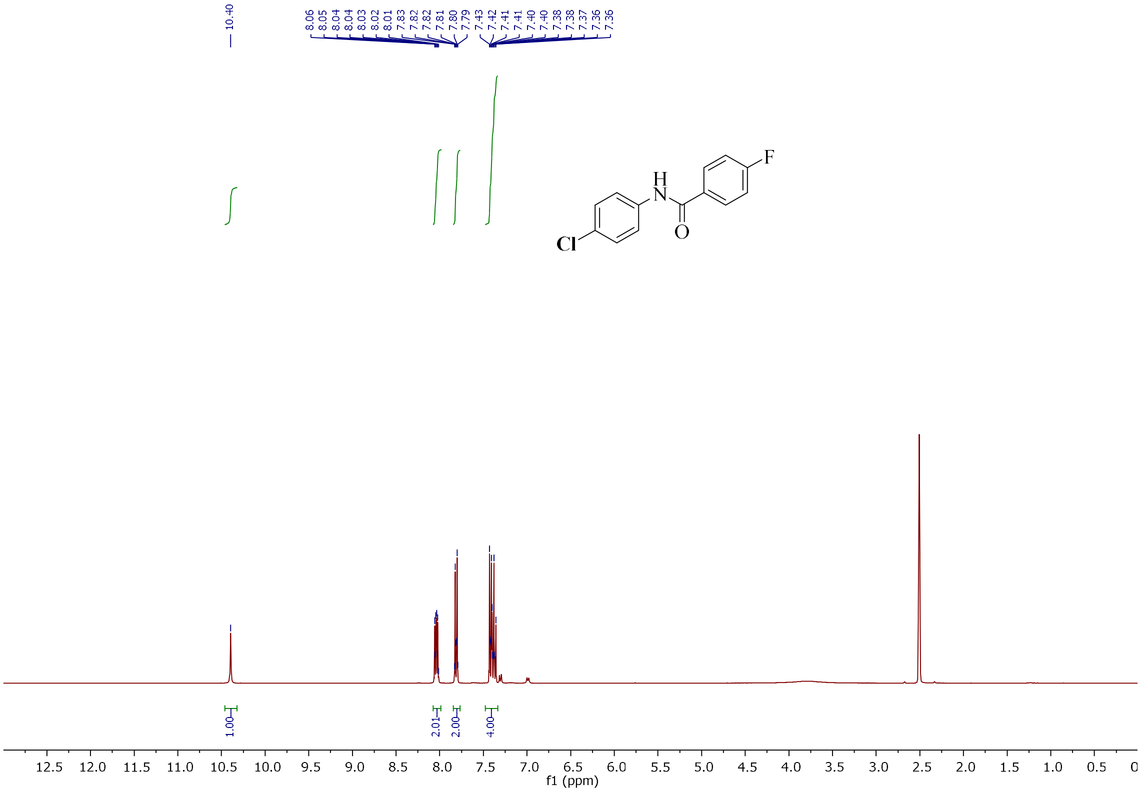

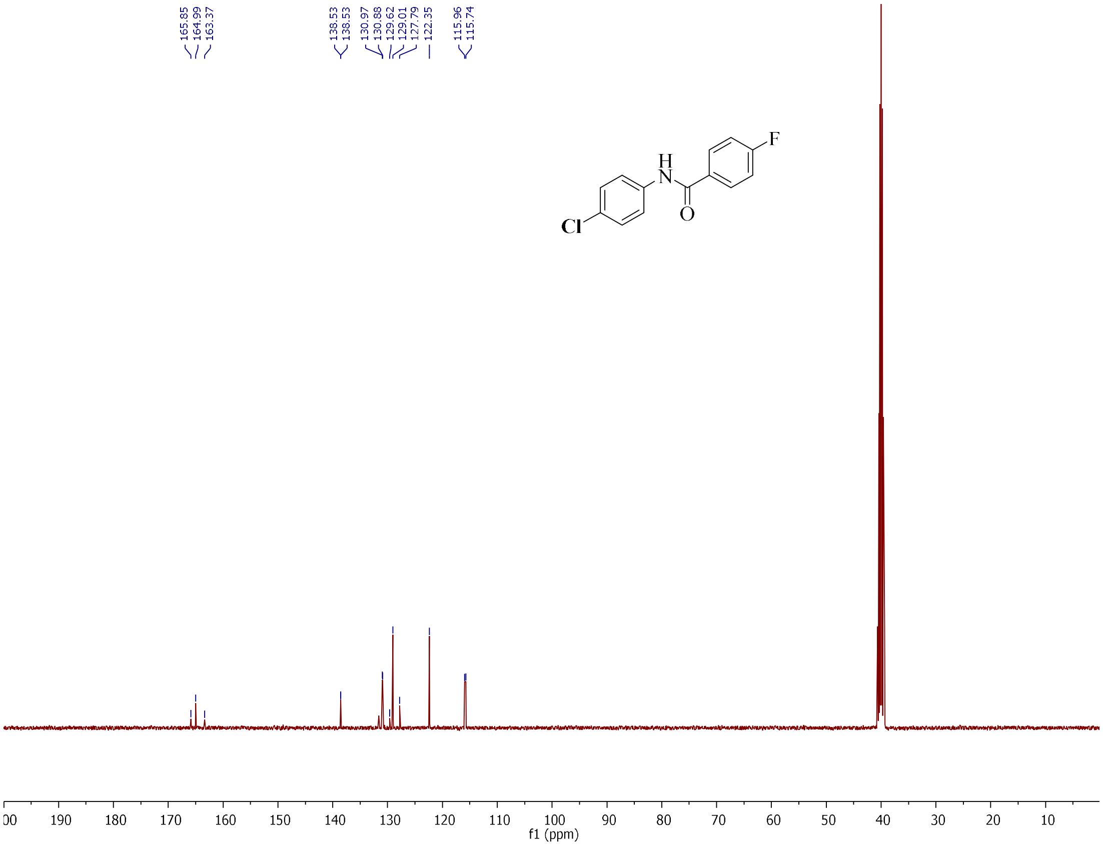

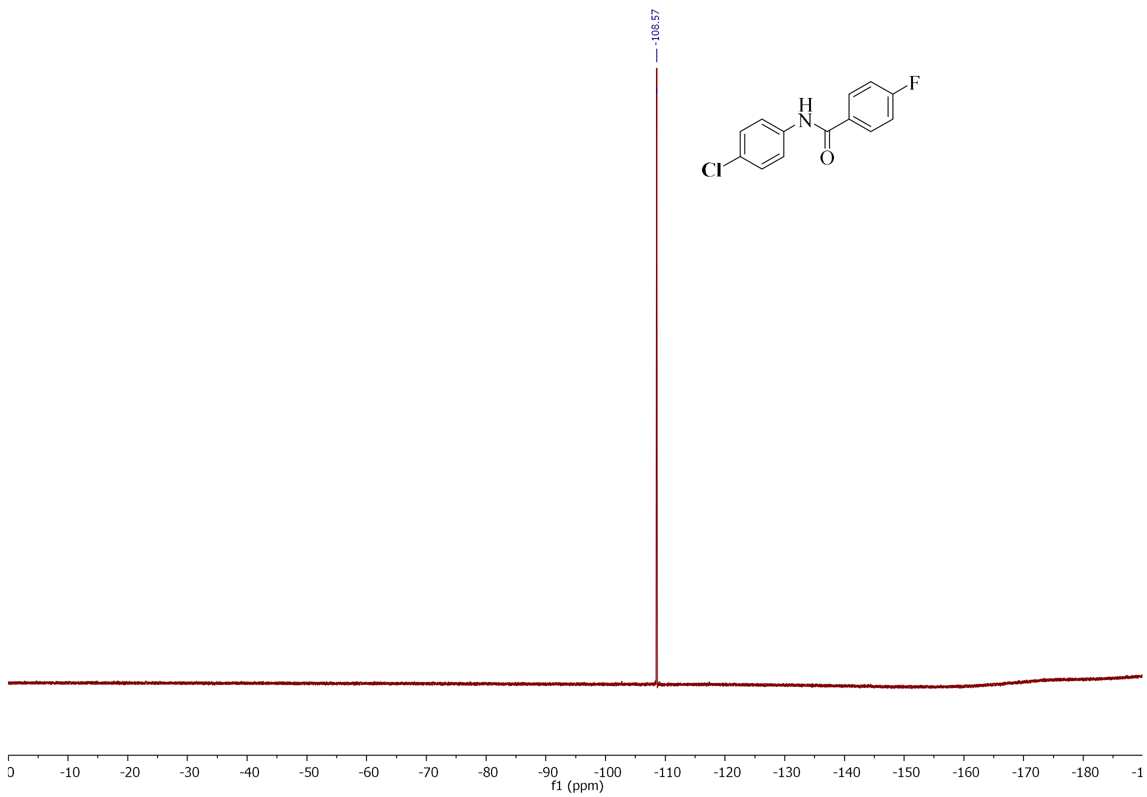
-108.57
H
N
F
Cl
O
0
-10
-20
-30
-40
-50
-60
-70
-80
-90
-100
-110
-120
-130
-140
-150
-160
-170
-180
f1 (ppm)

4-Chloro-*N*-(4-chlorophenyl)benzamide: (41)

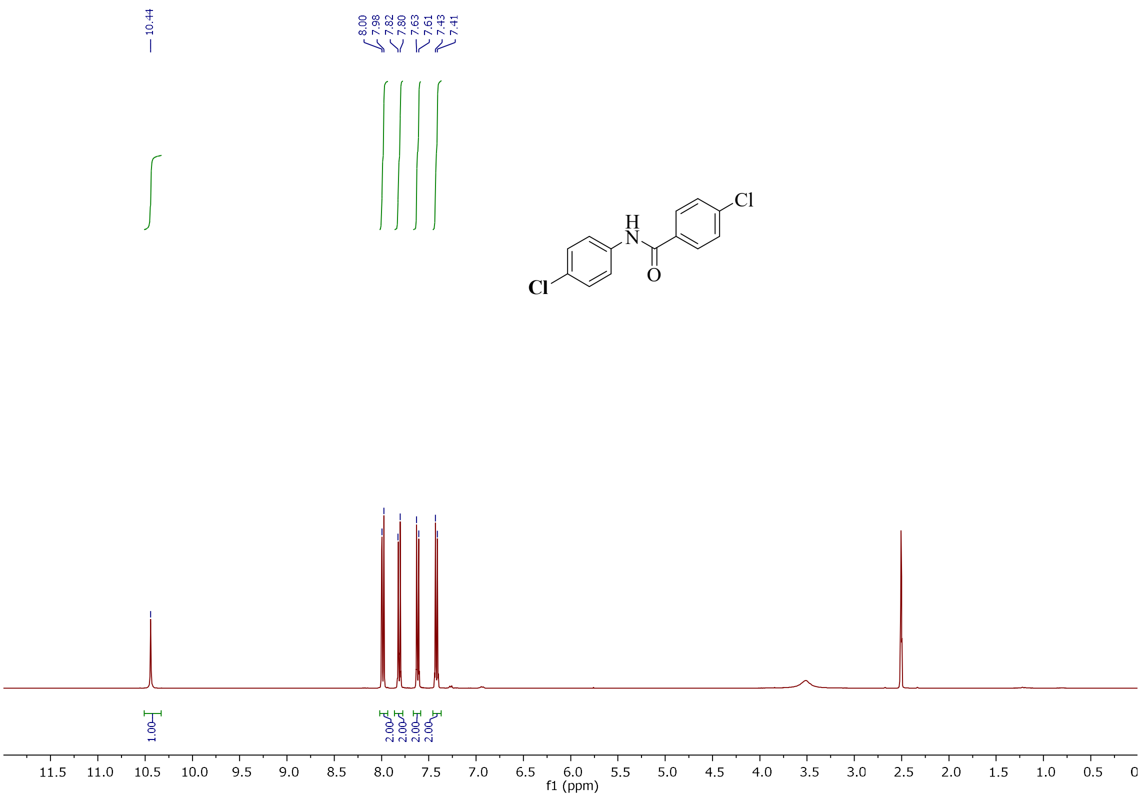

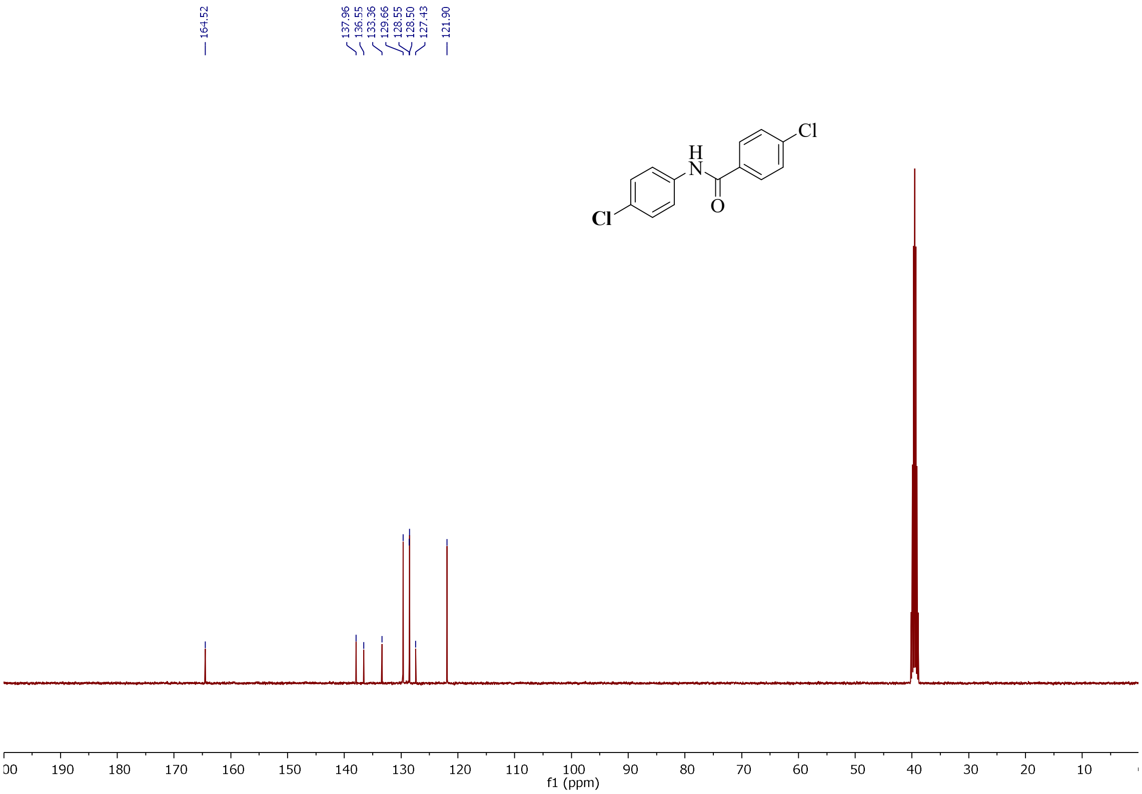

4-Bromo-N-(4-chlorophenyl)benzamide: (42)

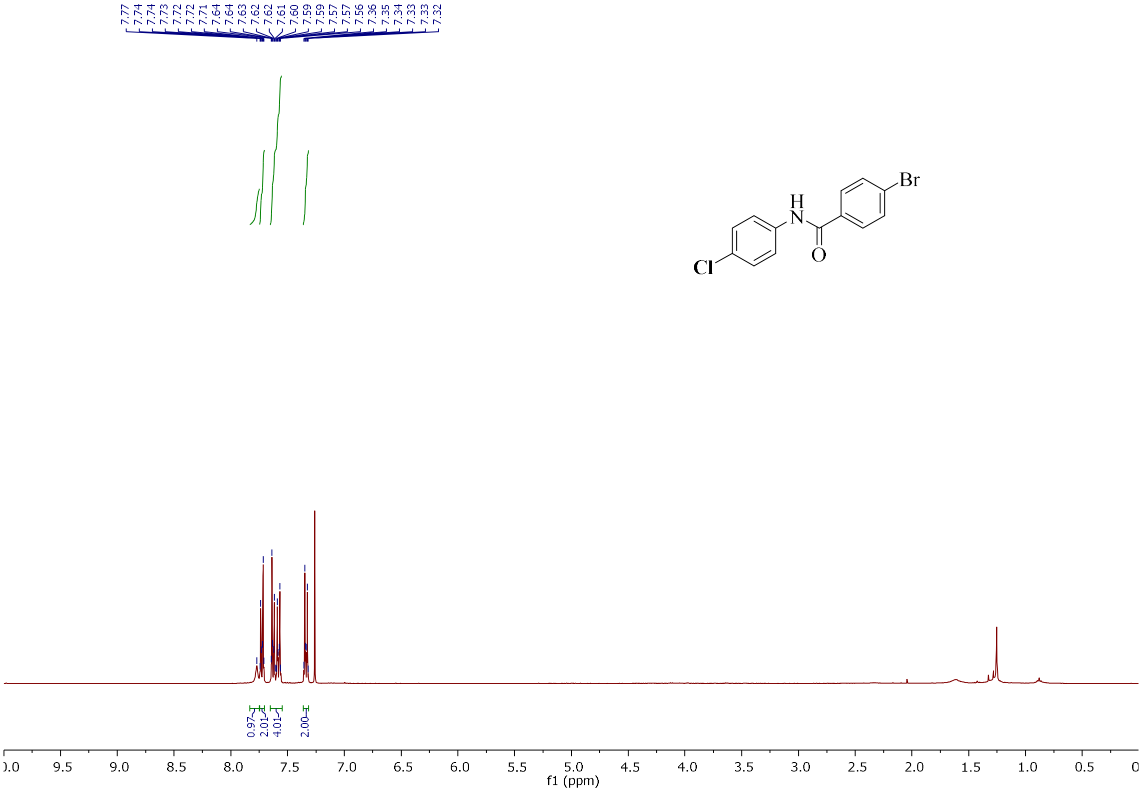

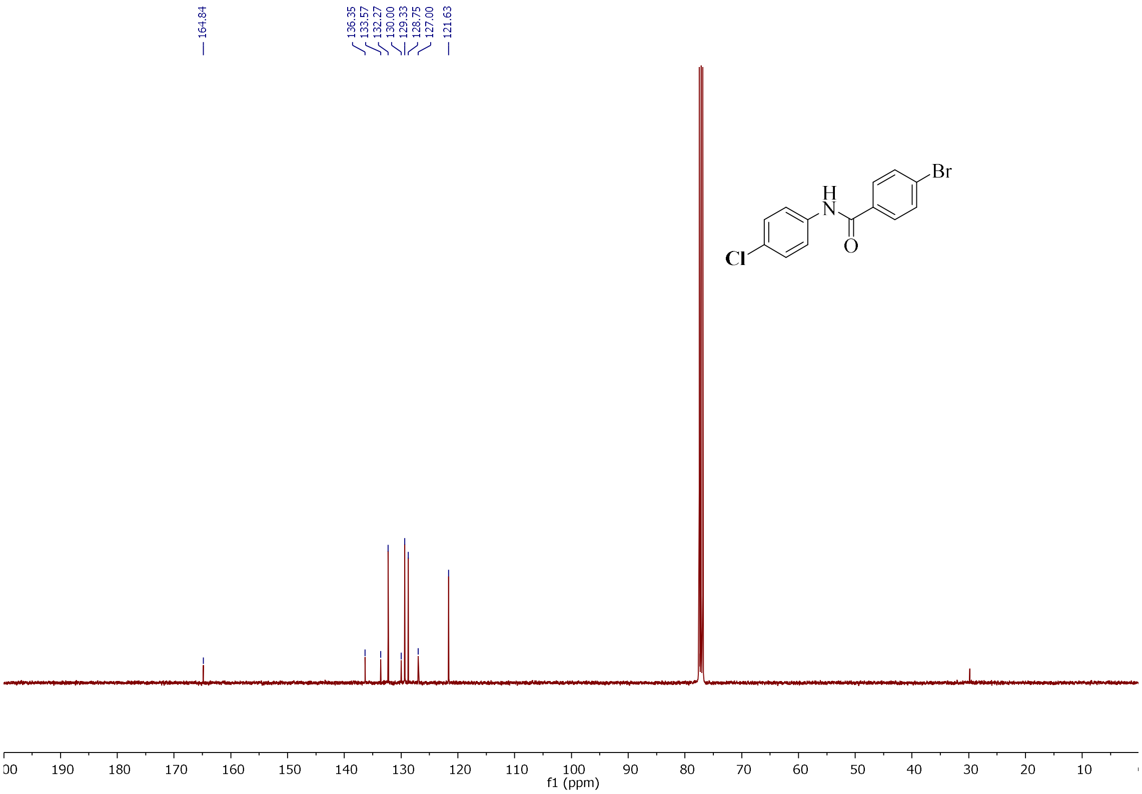

***N*-(4-chlorophenyl)-1-naphthamide: (43)**

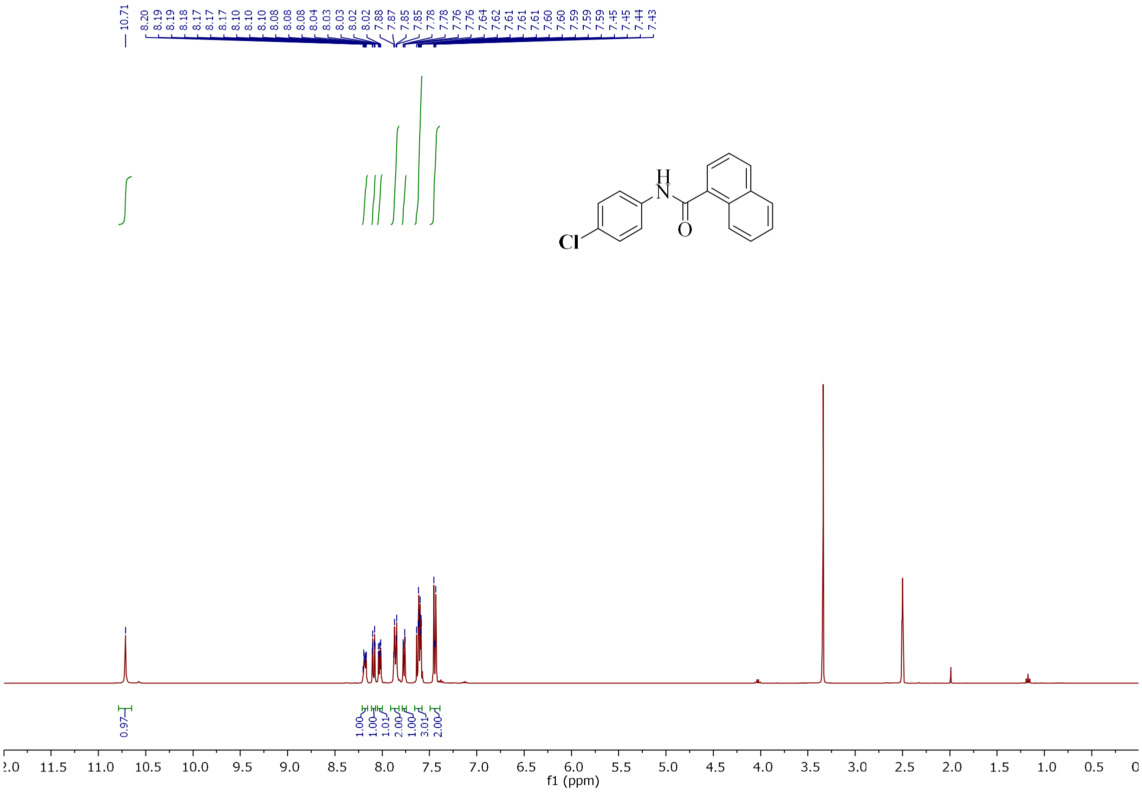

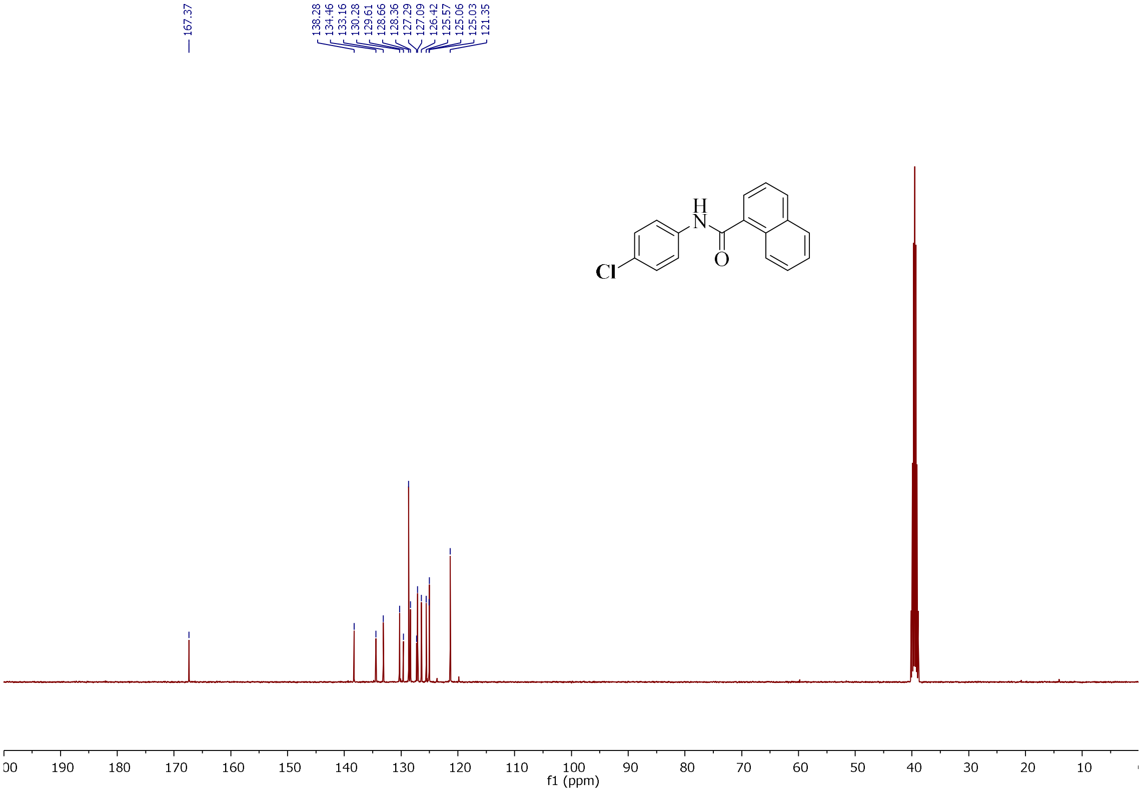

***N*-(4-chlorophenyl)-2-naphthamide: (44)**

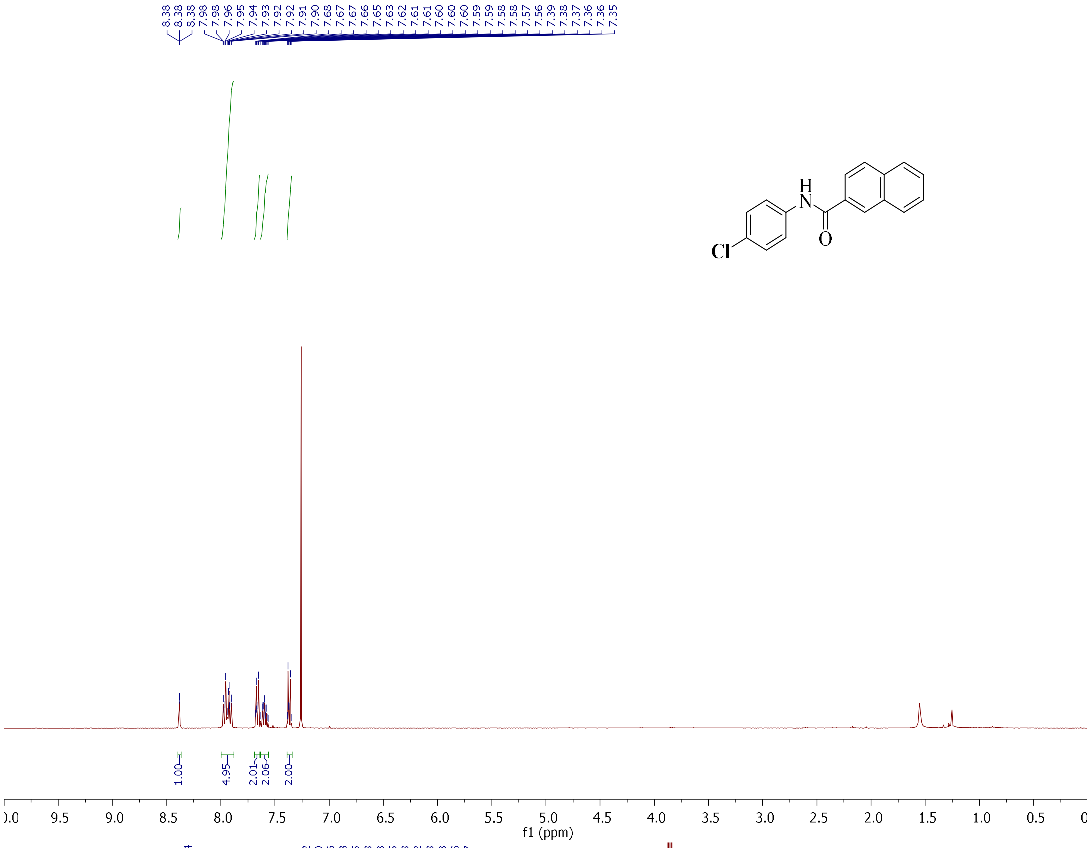

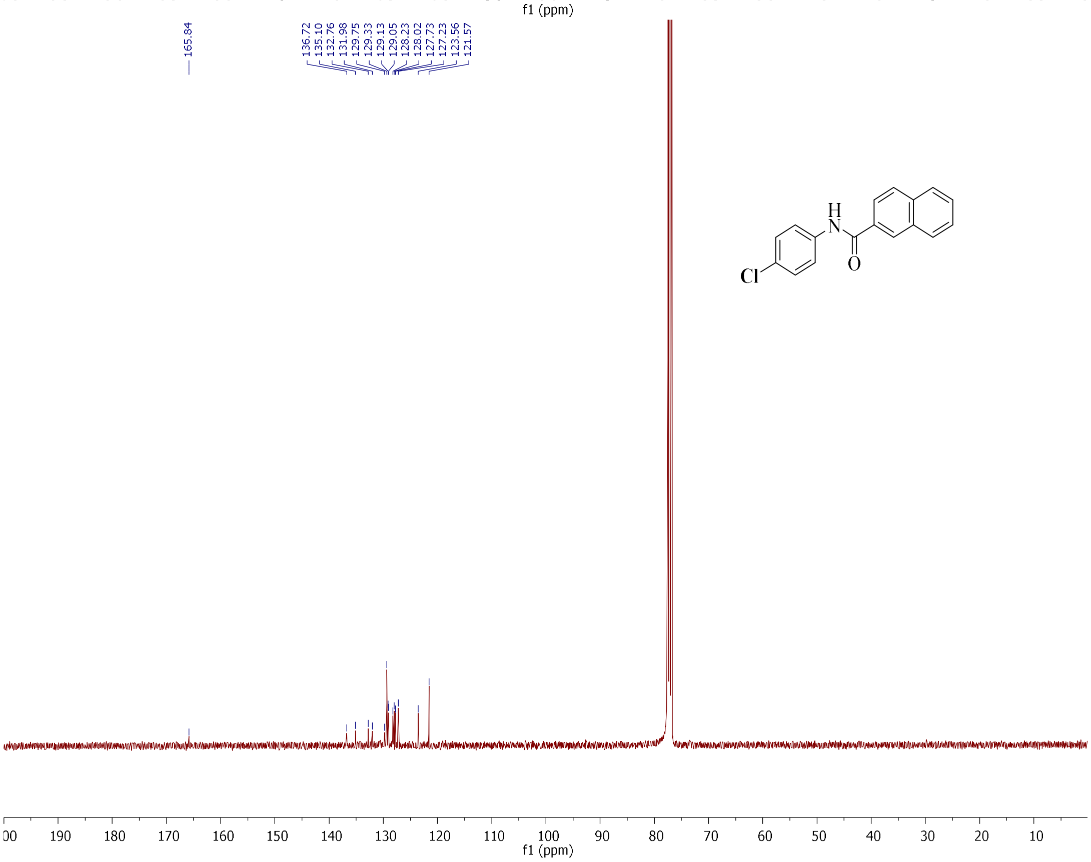

***N*-(4-chlorophenyl)benzo[*b*]thiophene-2-carboxamide: (45)**

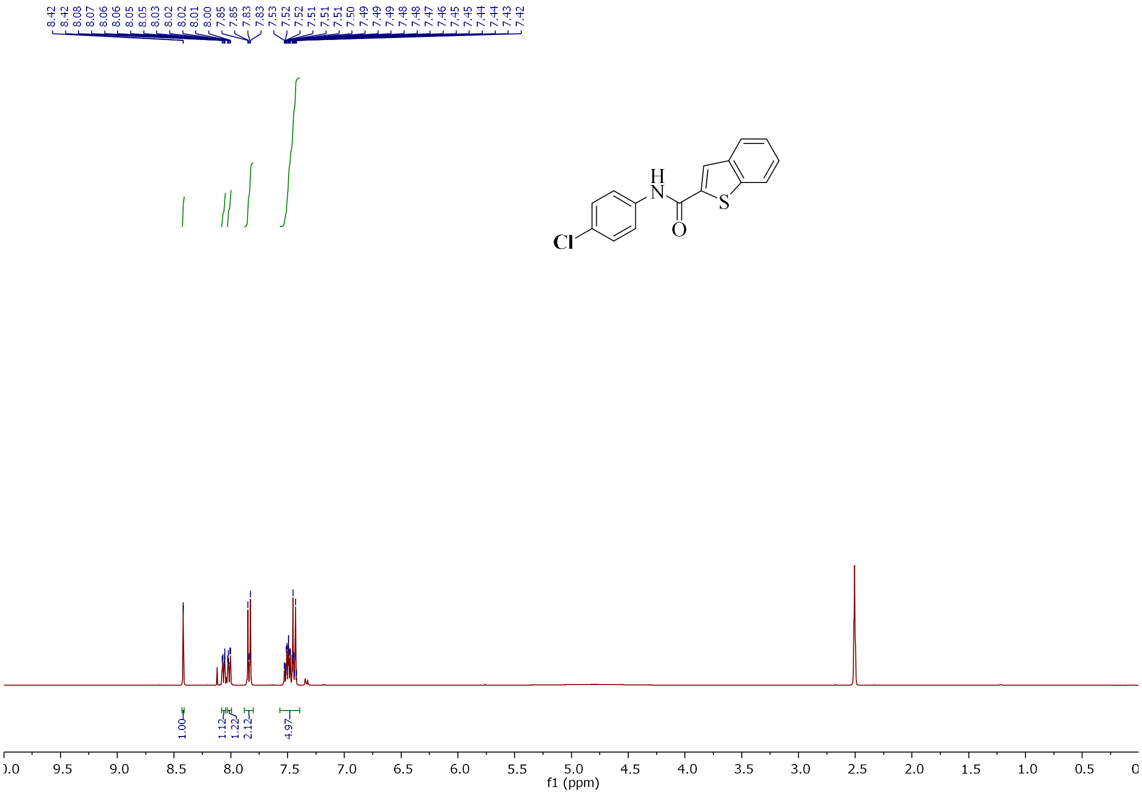

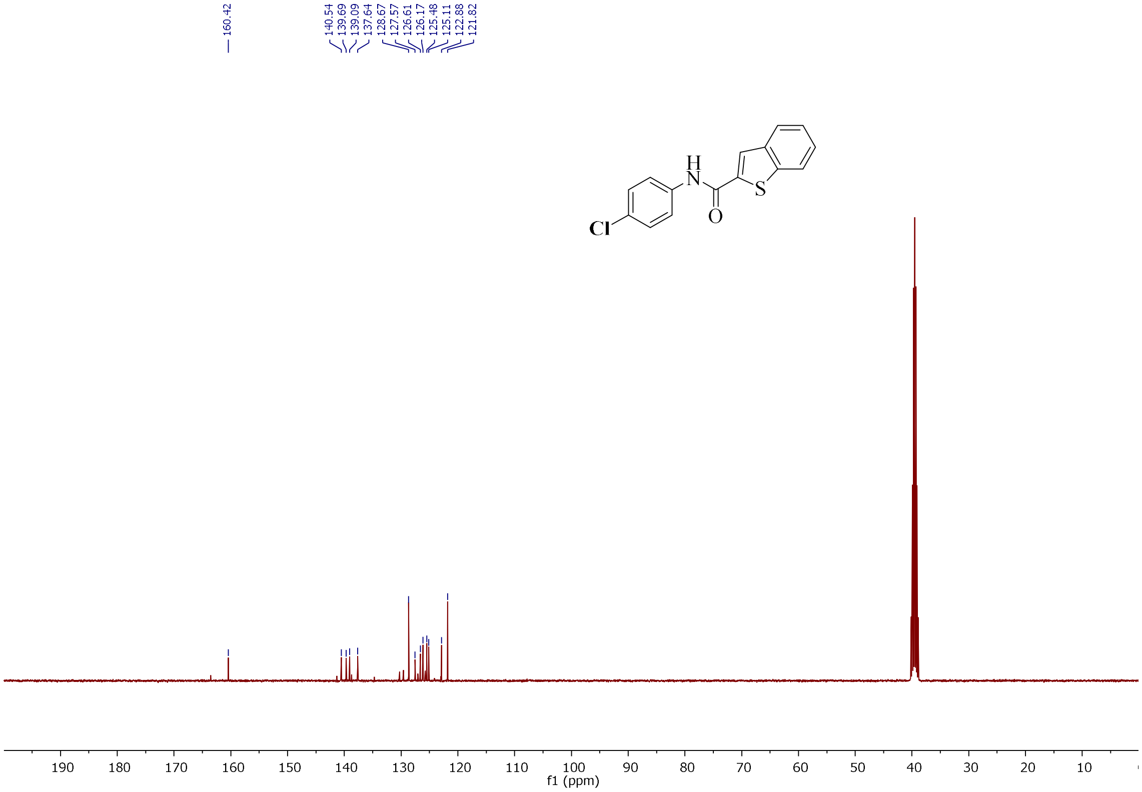

***N*-(4-chlorophenyl)benzofuran-2-carboxamide: (46)**

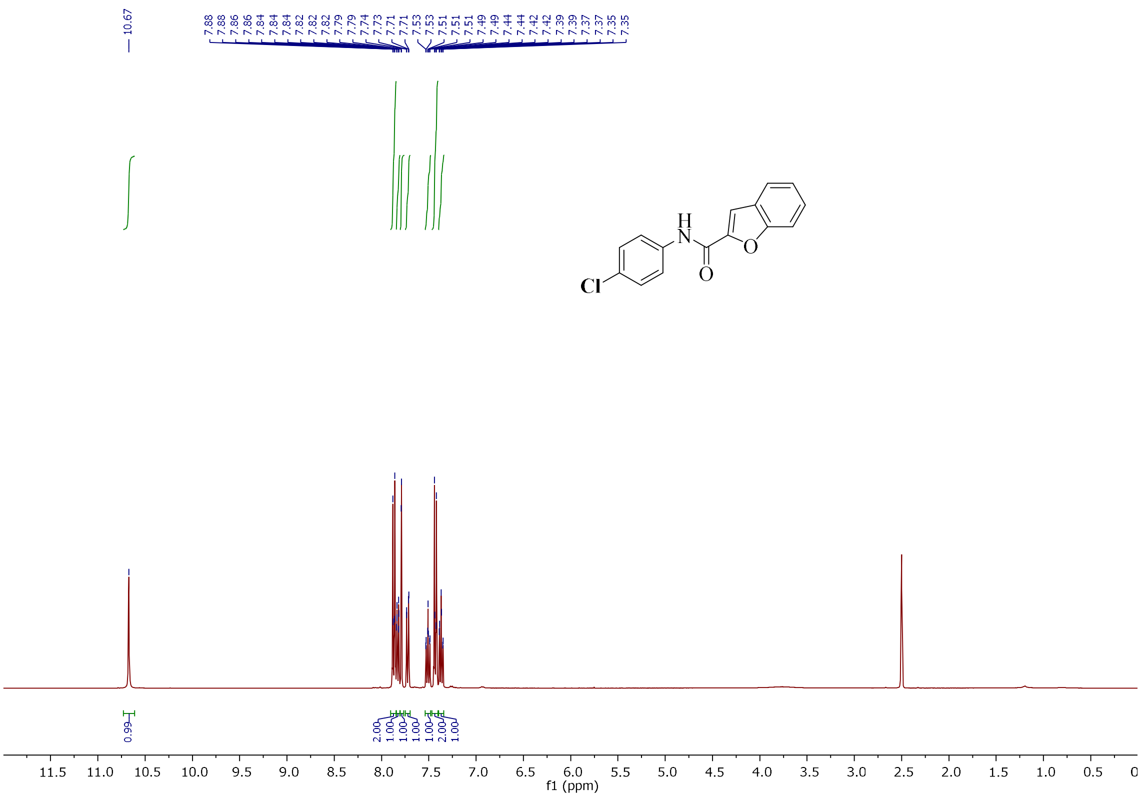

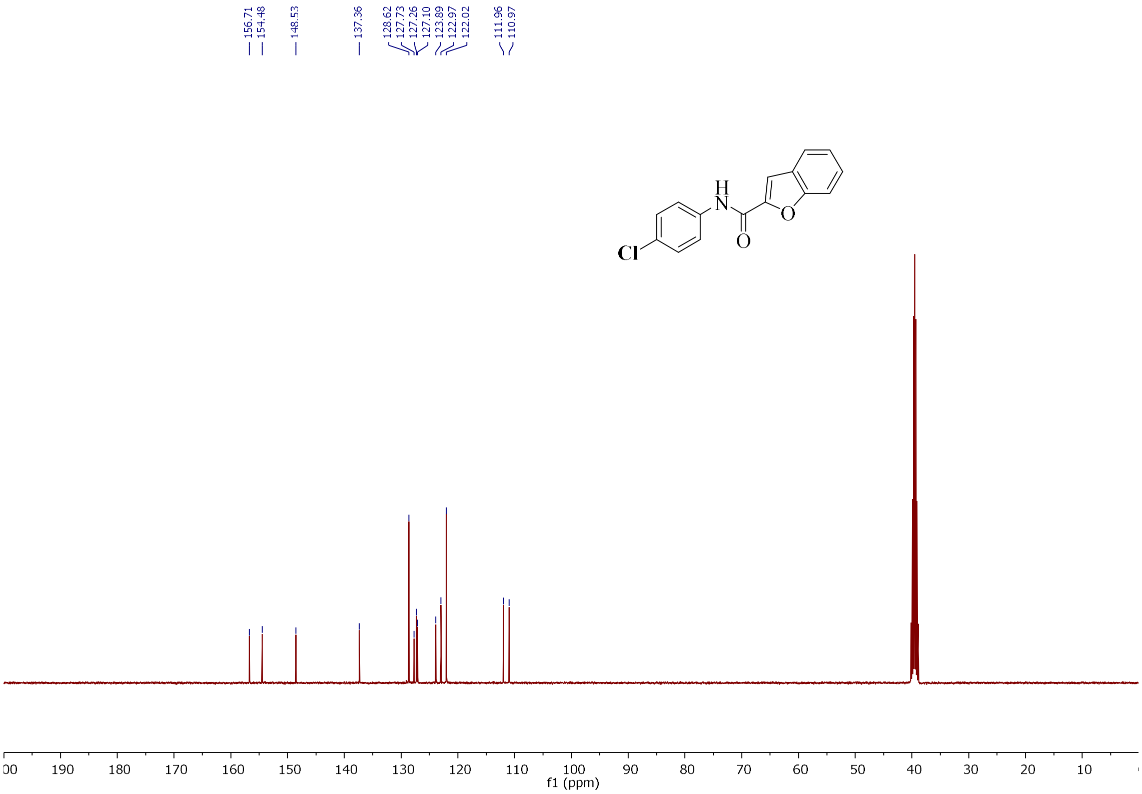

Ethyl (4-chlorophenyl)carbamate: (47)

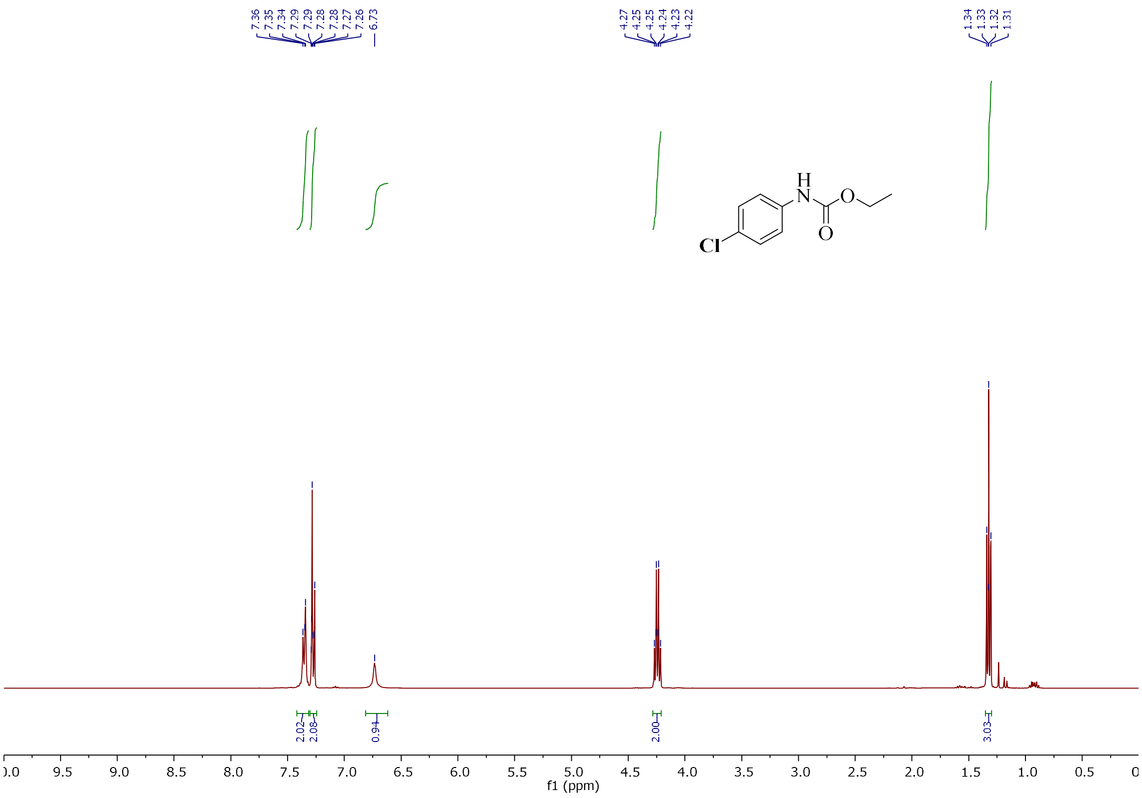

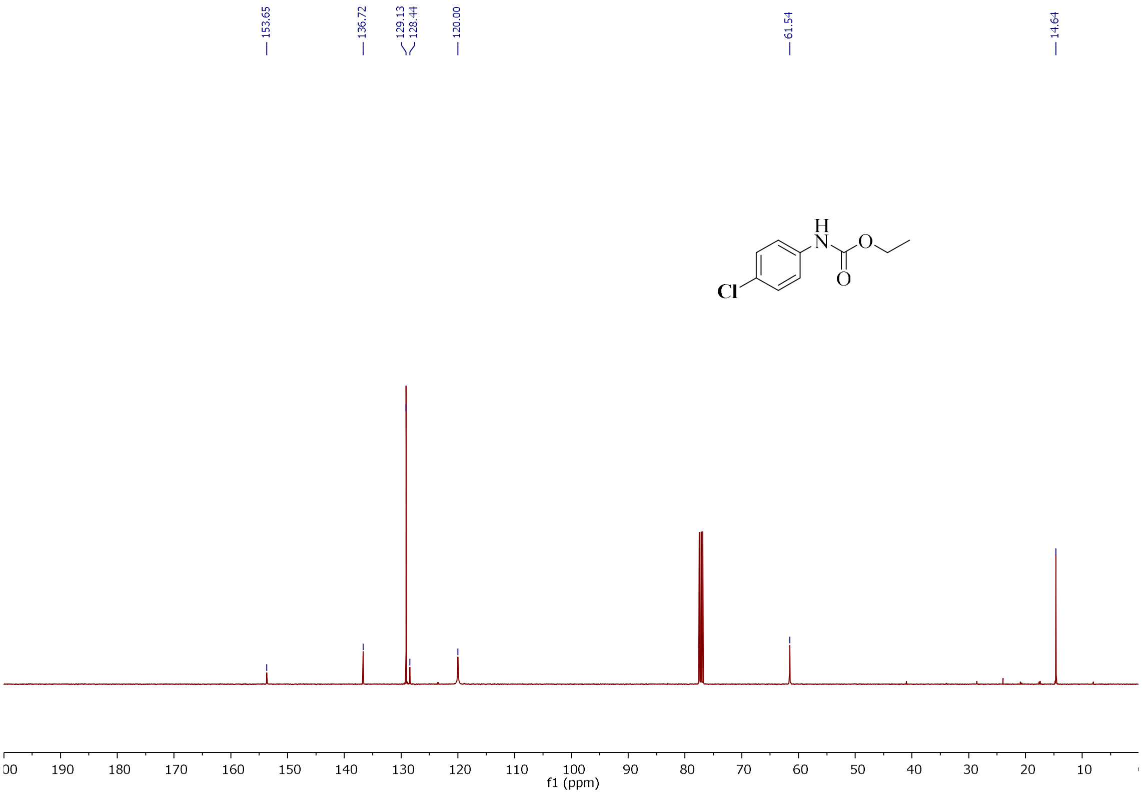

Butyl (4-chlorophenyl)carbamate: (48)

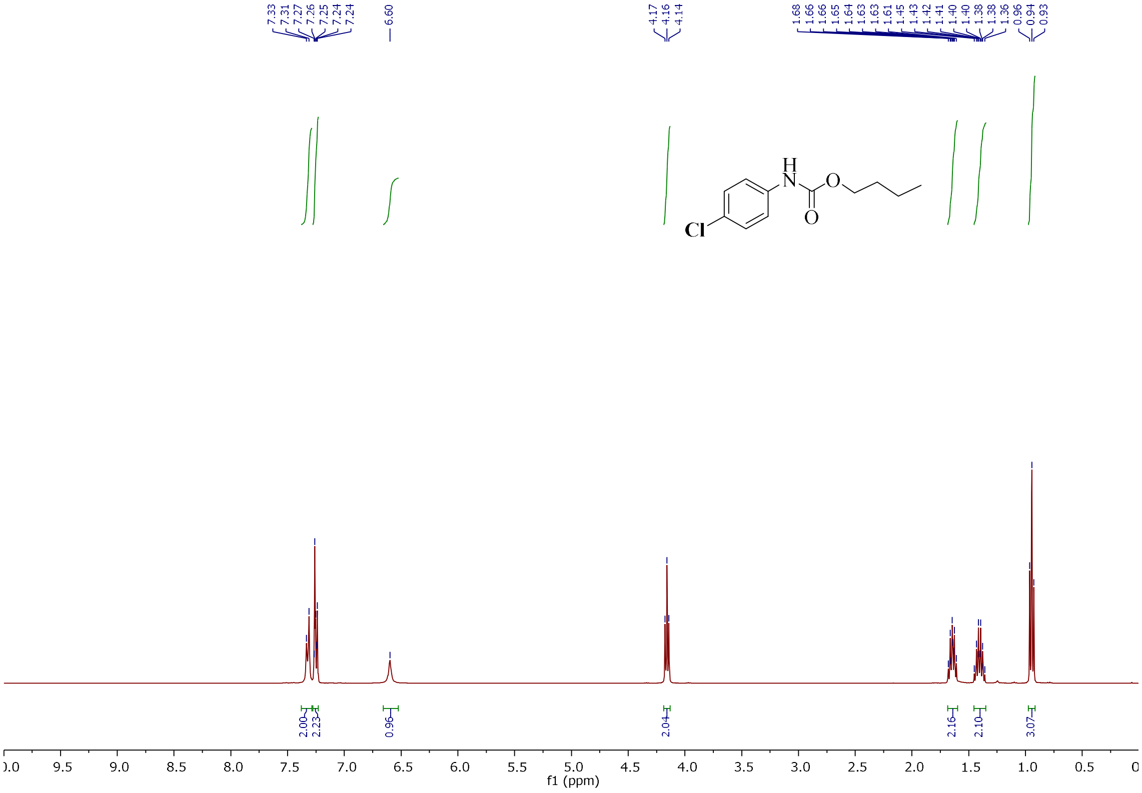

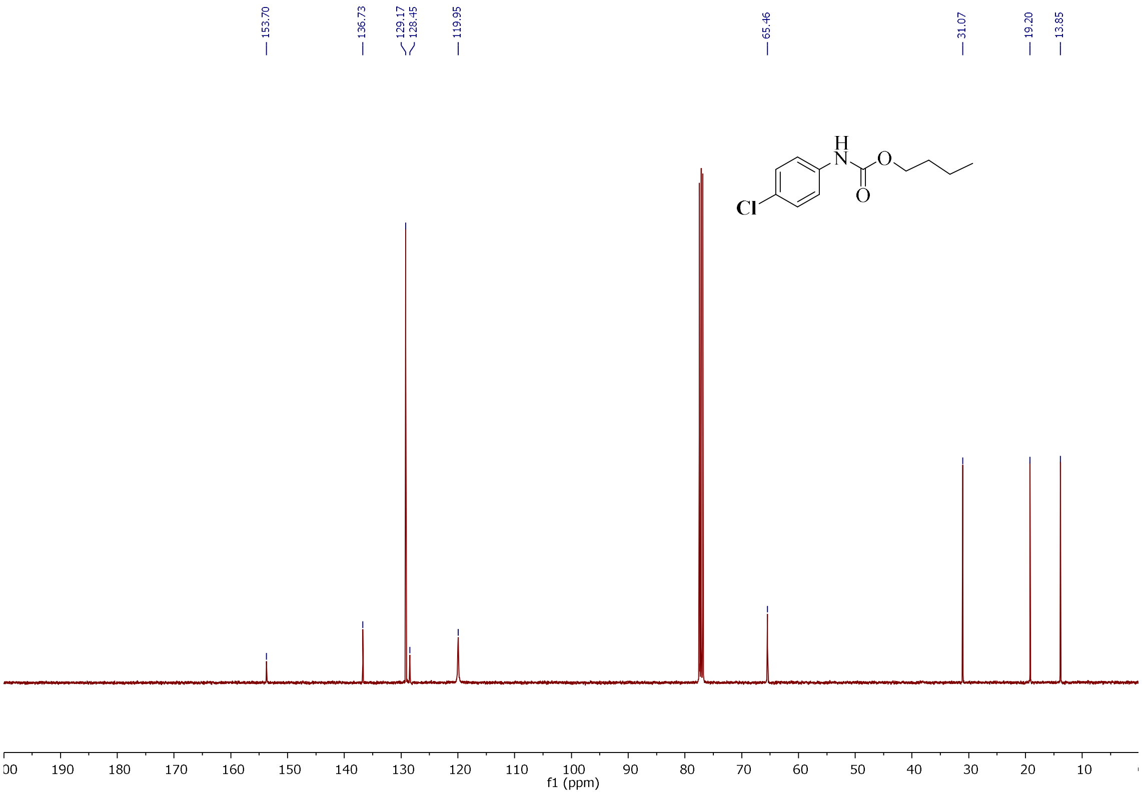

Isobutyl (4-chlorophenyl)carbamate: (49)

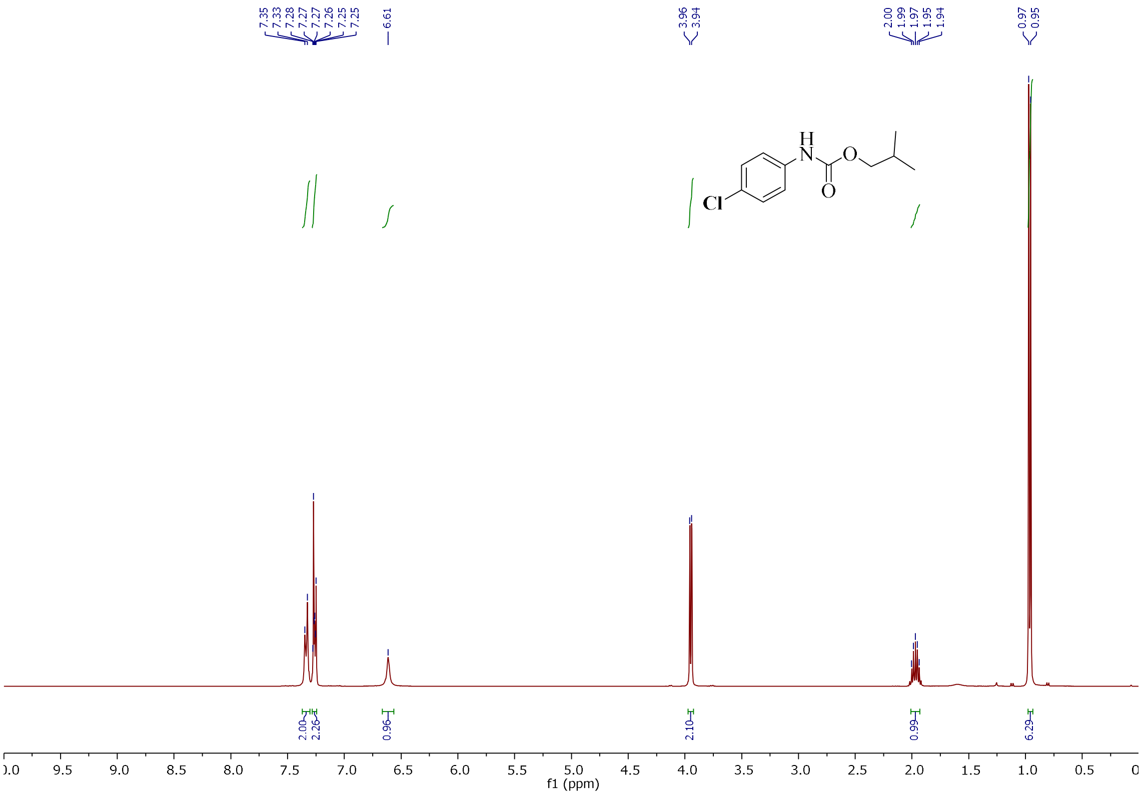

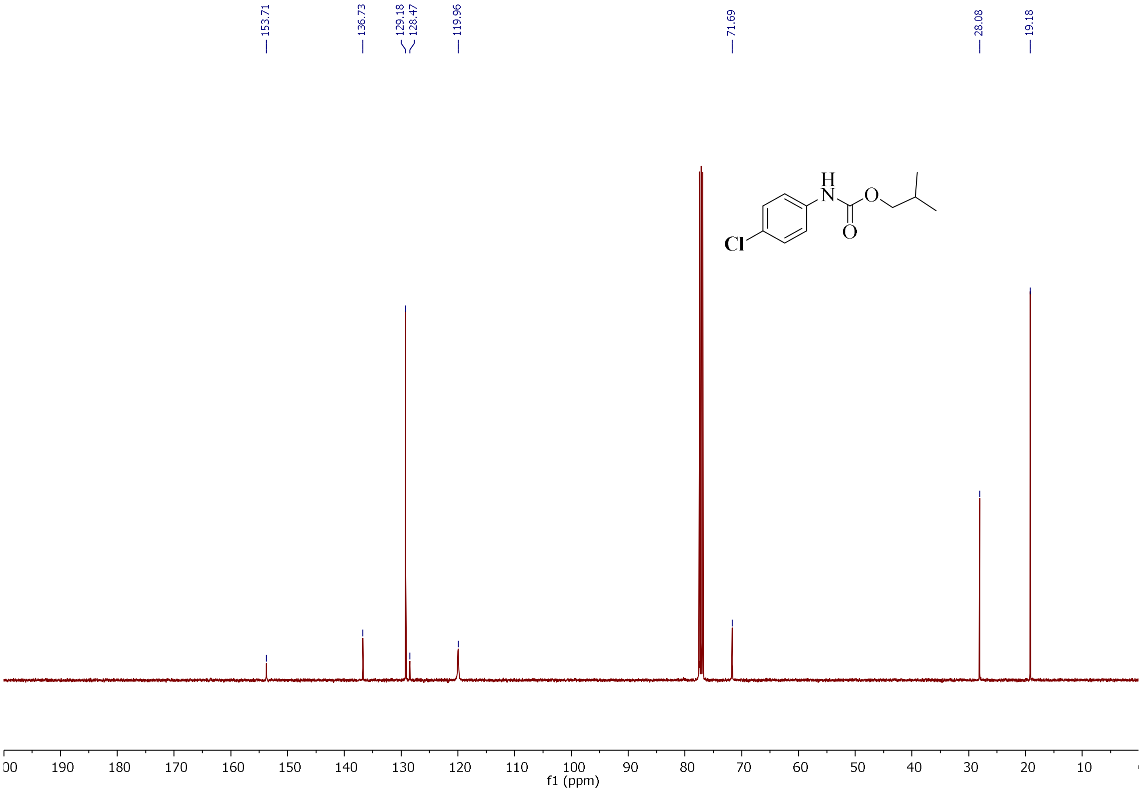

Benzyl (4-chlorophenyl)carbamate: (50)

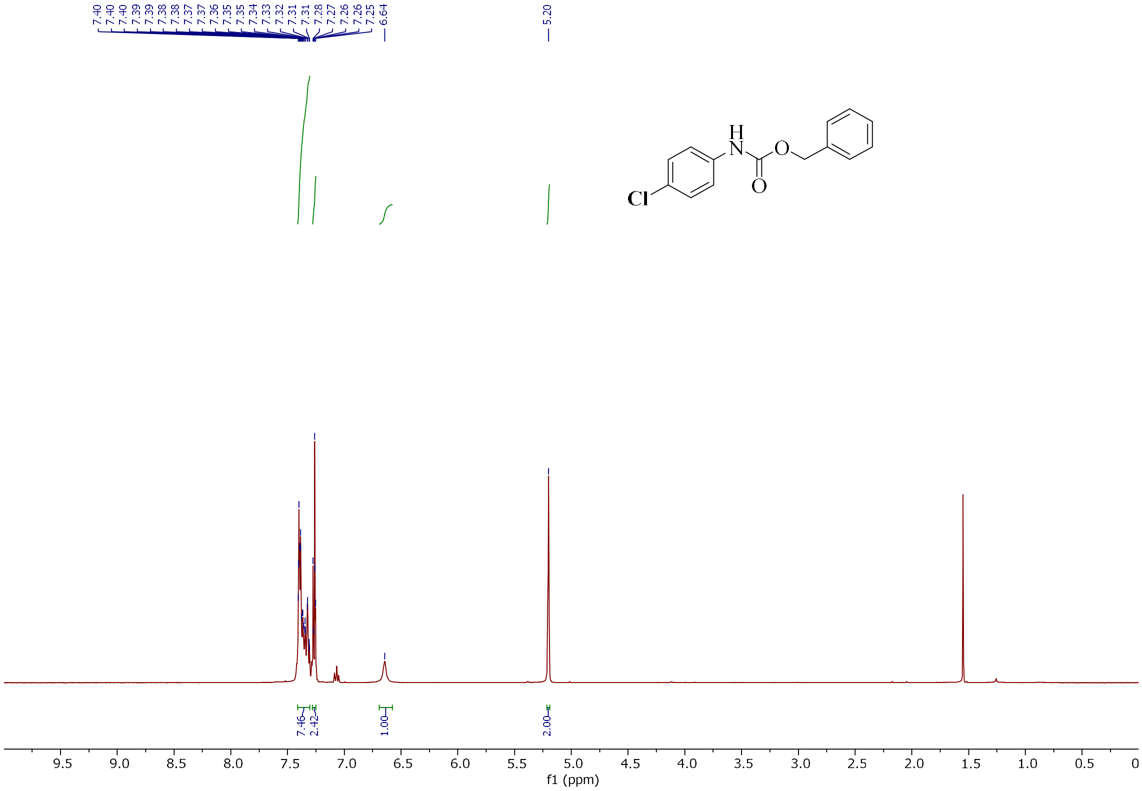

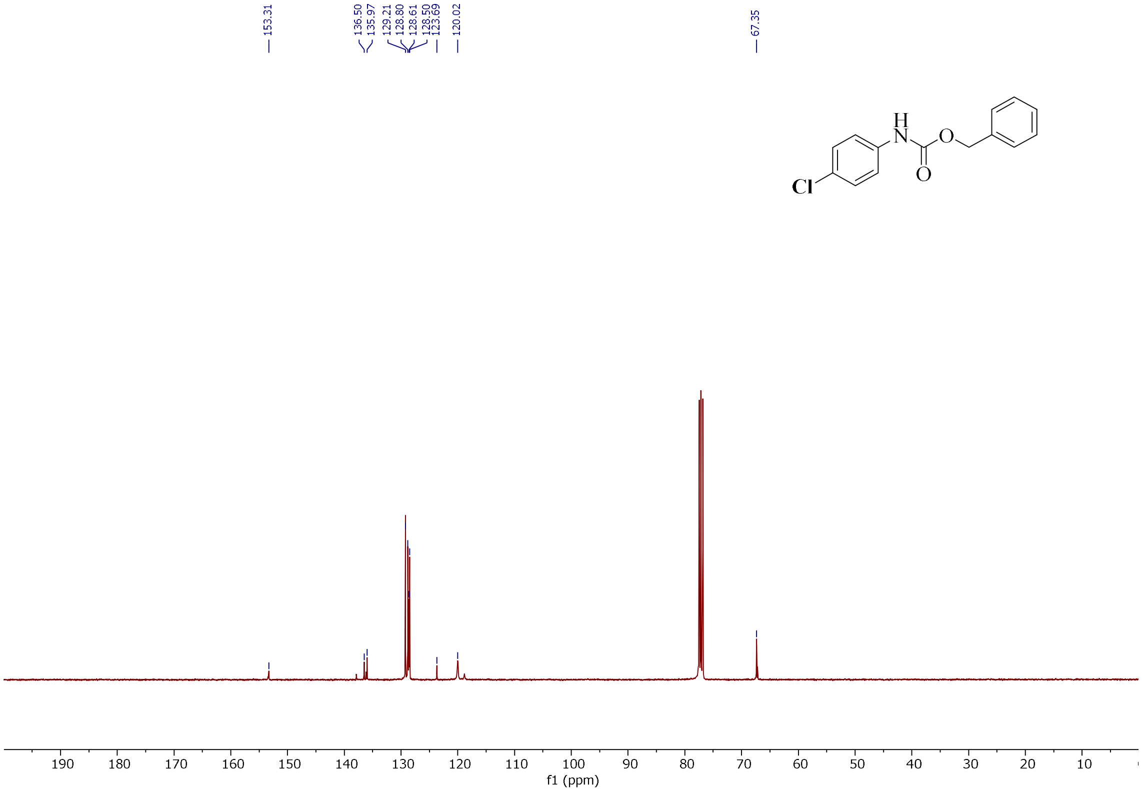

***N*-(4-chlorophenyl)-4-methylpiperazine-1-carboxamide: (51)**

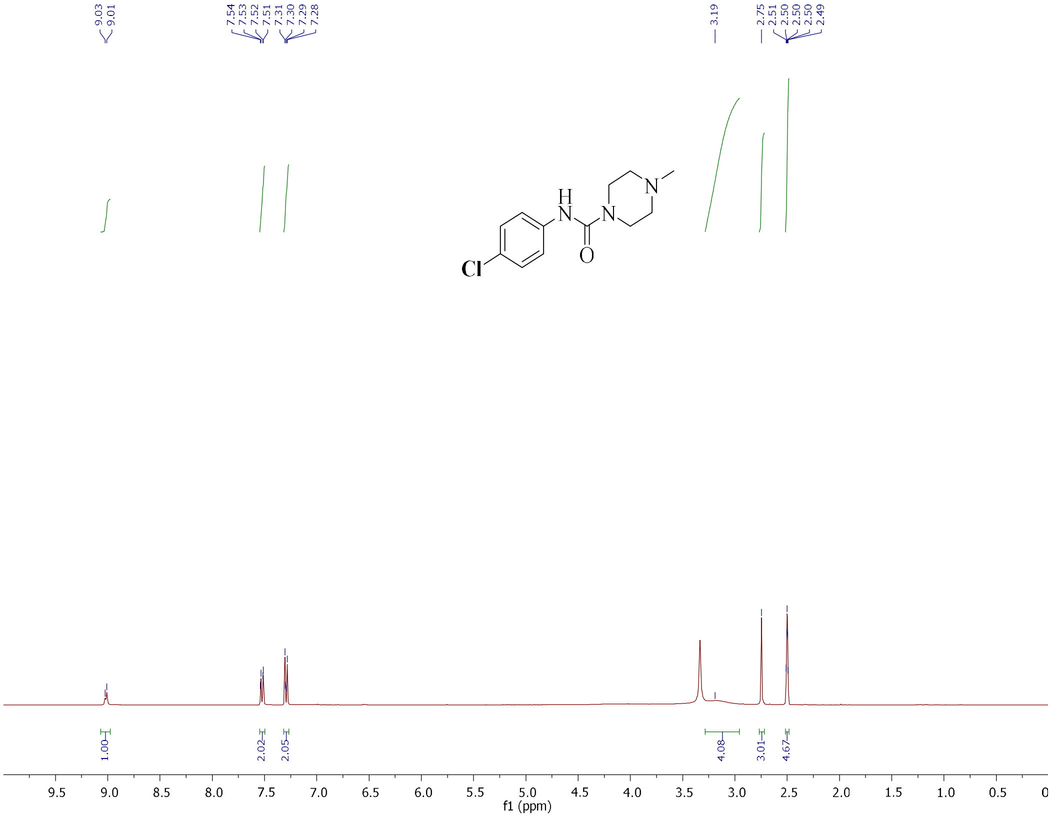

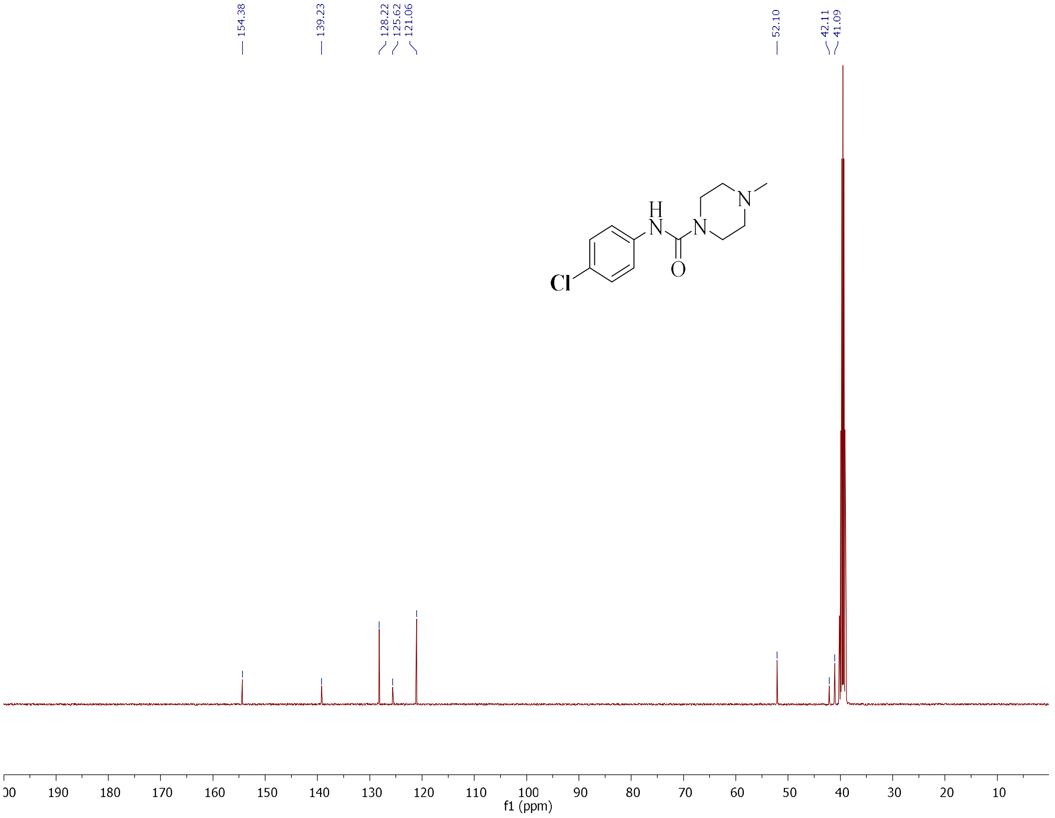

***N*-(4-chlorophenyl)morpholine-4-carboxamide: (52)**

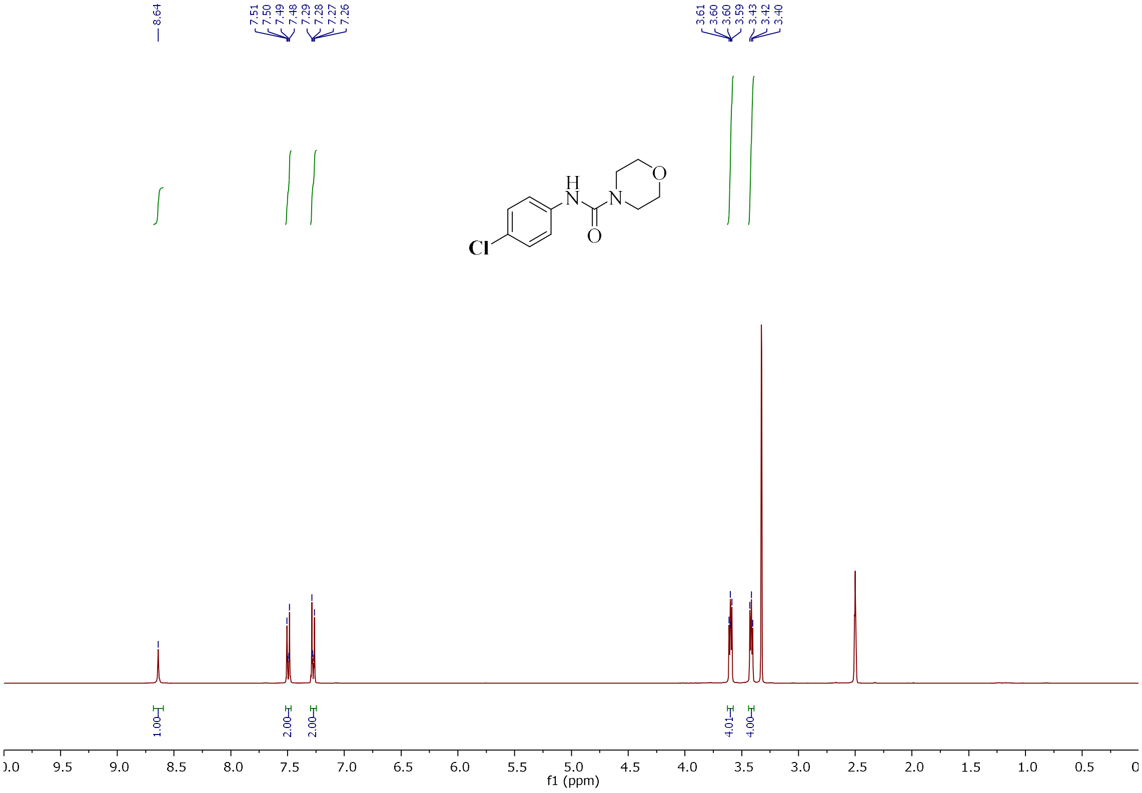

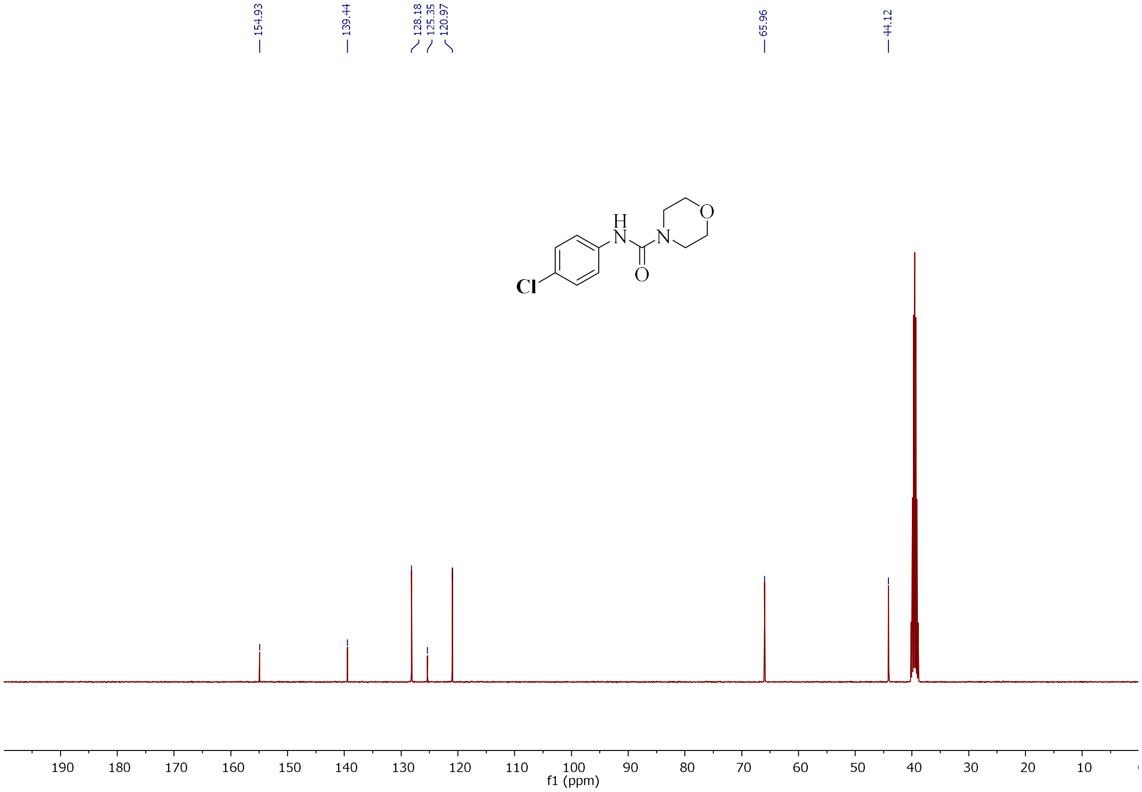

***N*-(4-chlorophenyl)-3-methylbut-2-enamide: (53)**

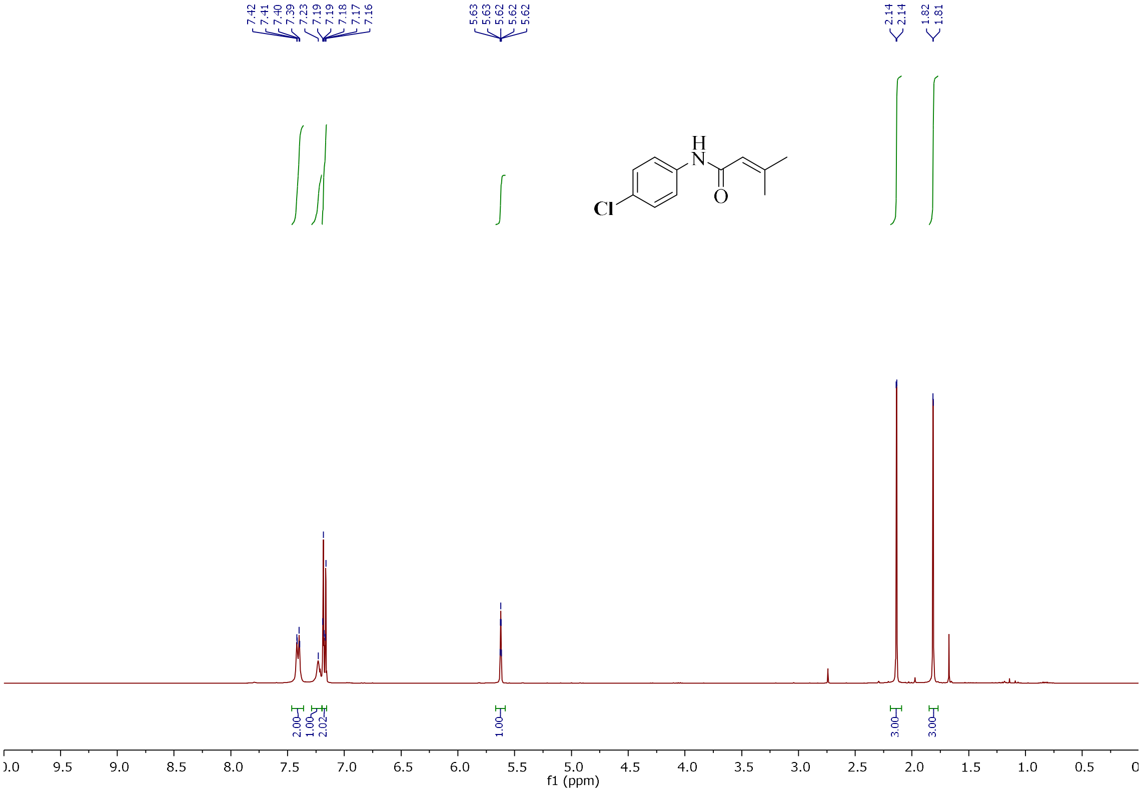

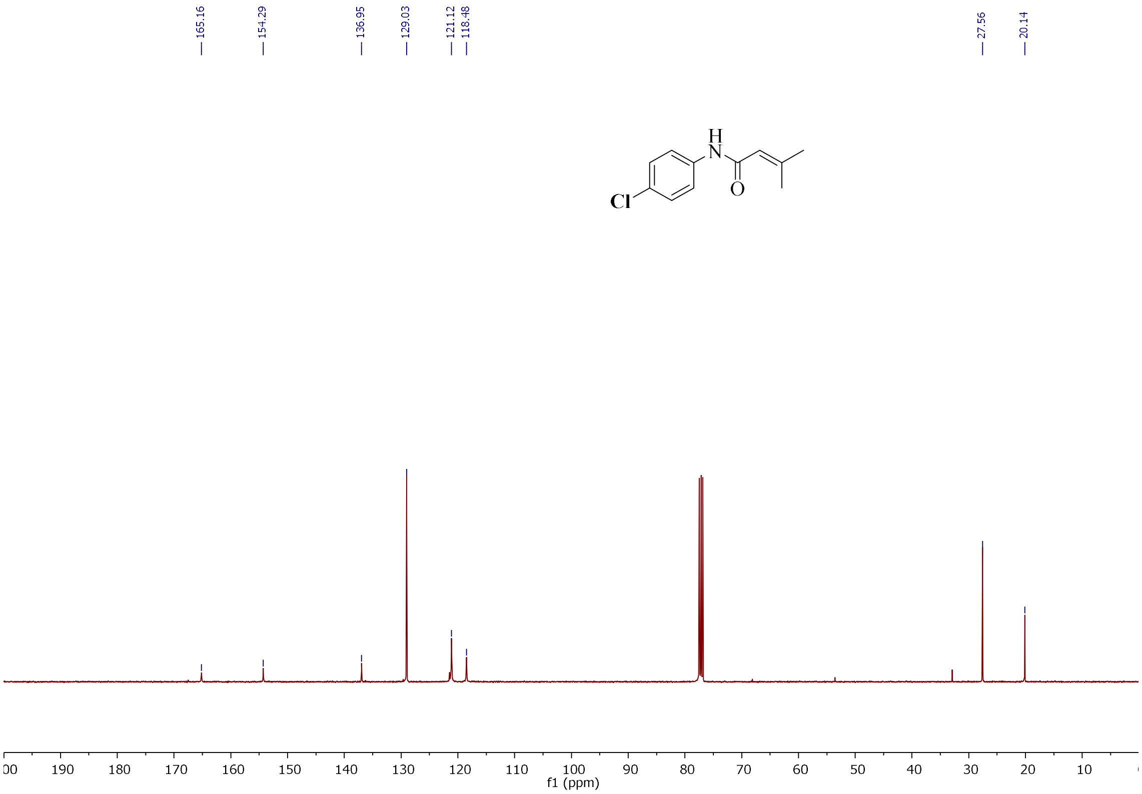

***N*-(4-chlorophenyl)cinnamamide: (54)**

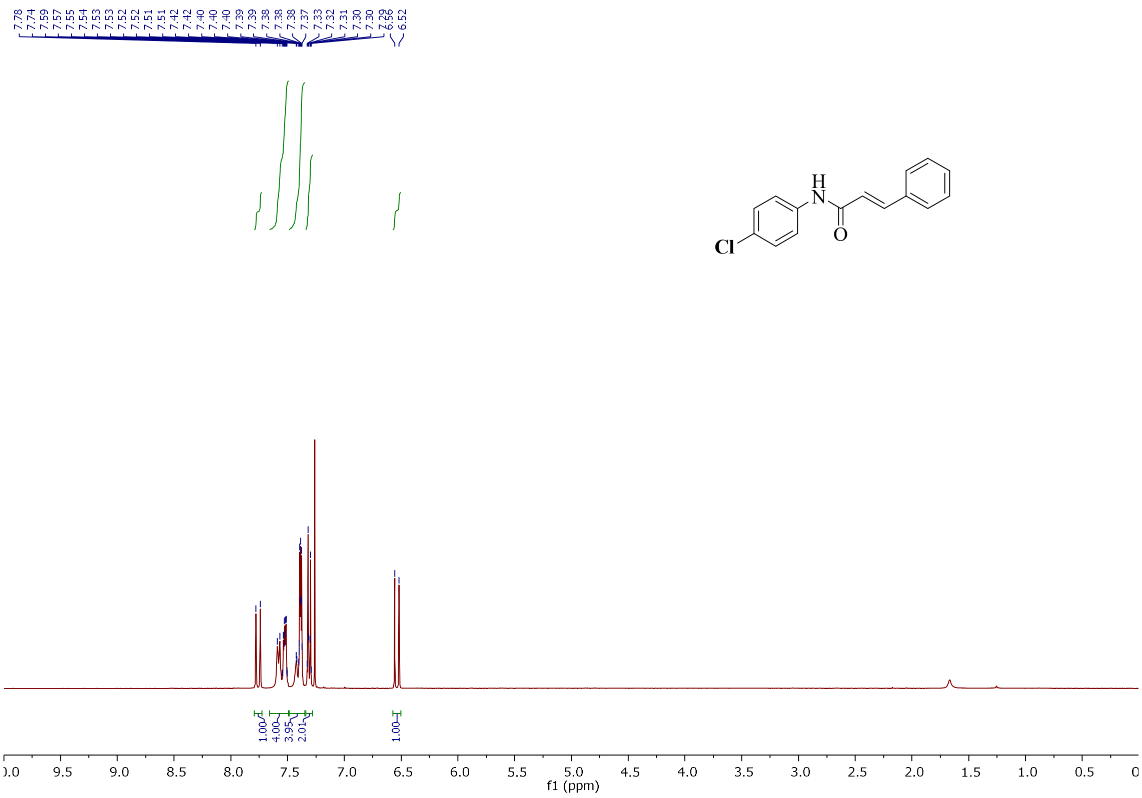

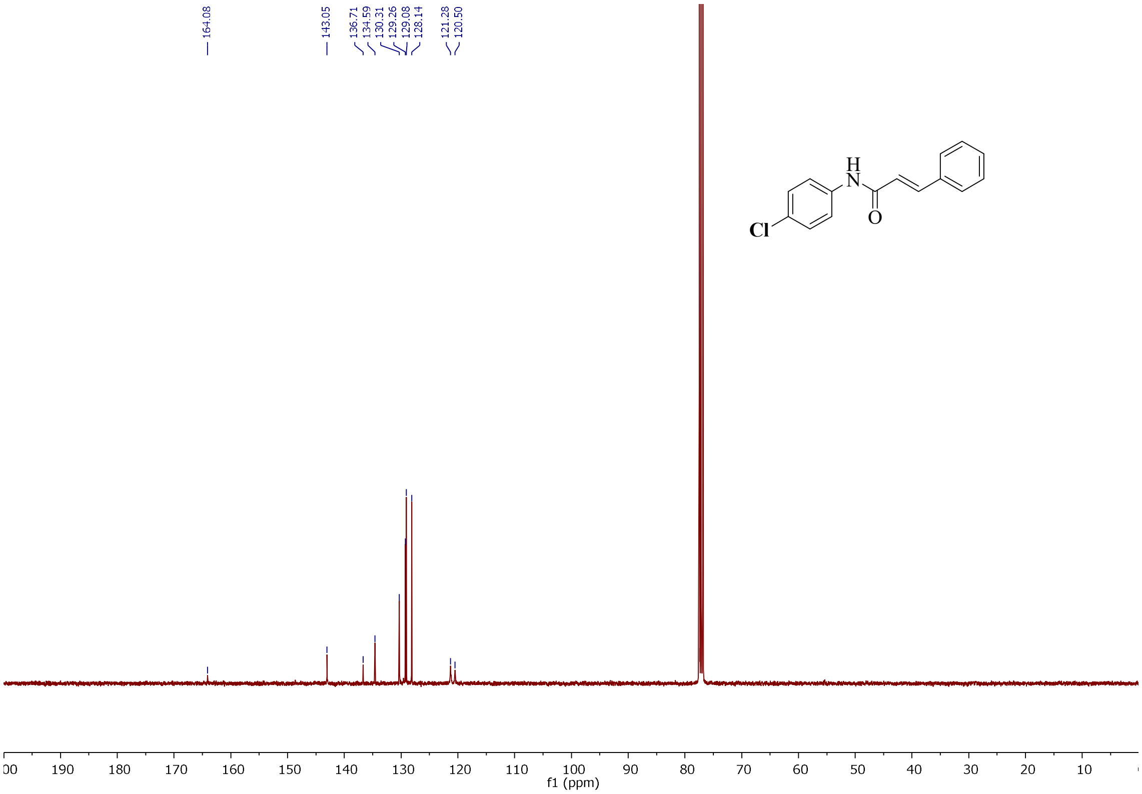

N-(5-chloro-2,4-dimethoxyphenyl)cyclohexanecarboxamide: (55)

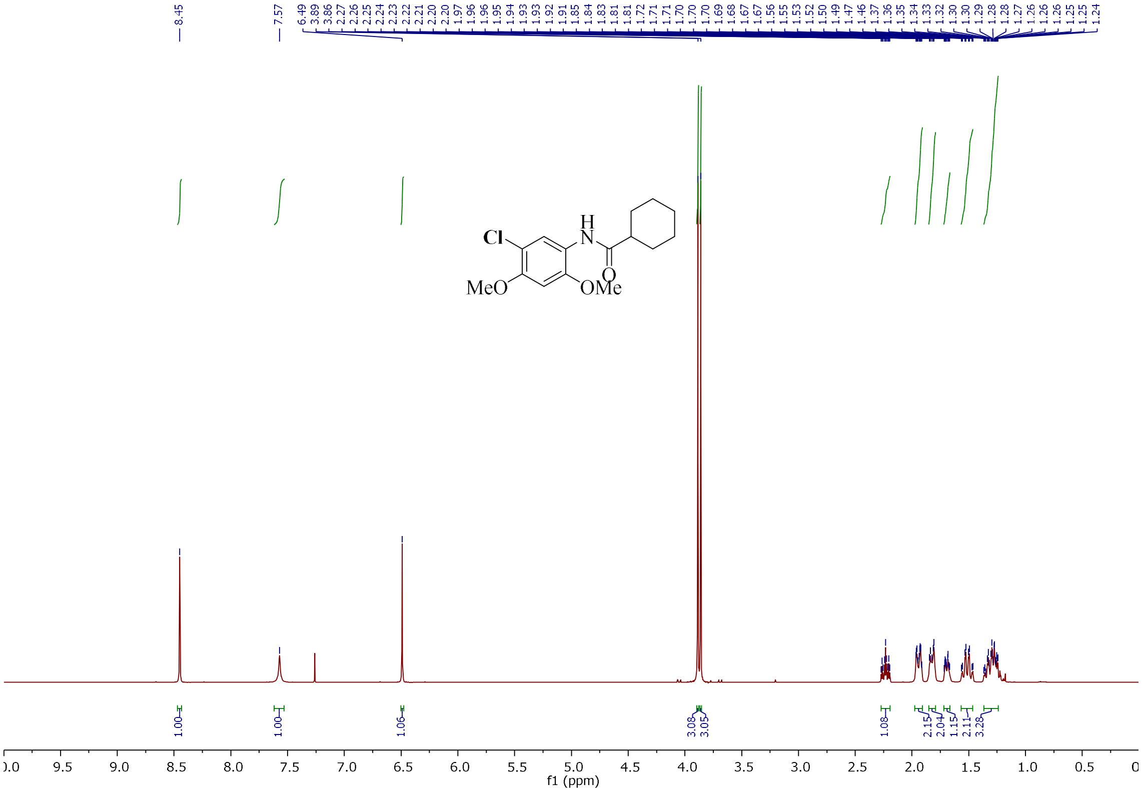

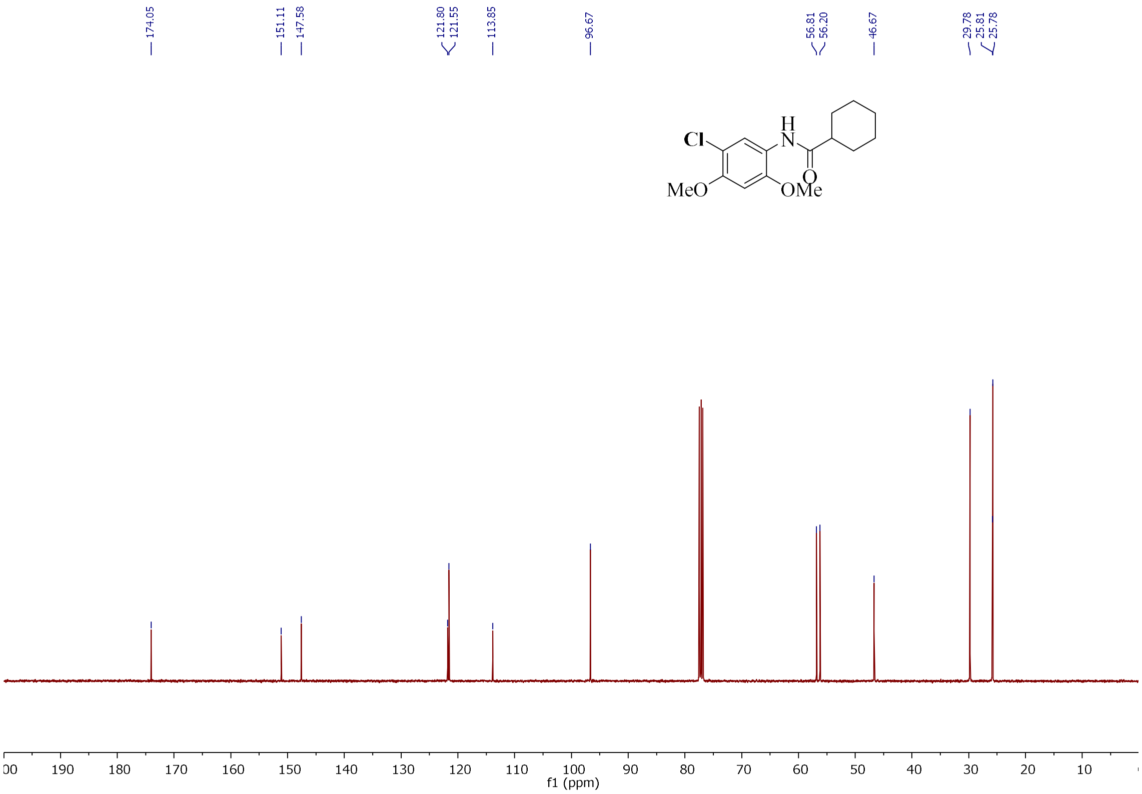

***N*-(2-chloro-4,5-dimethoxyphenyl)cyclohexanecarboxamide: (56)**

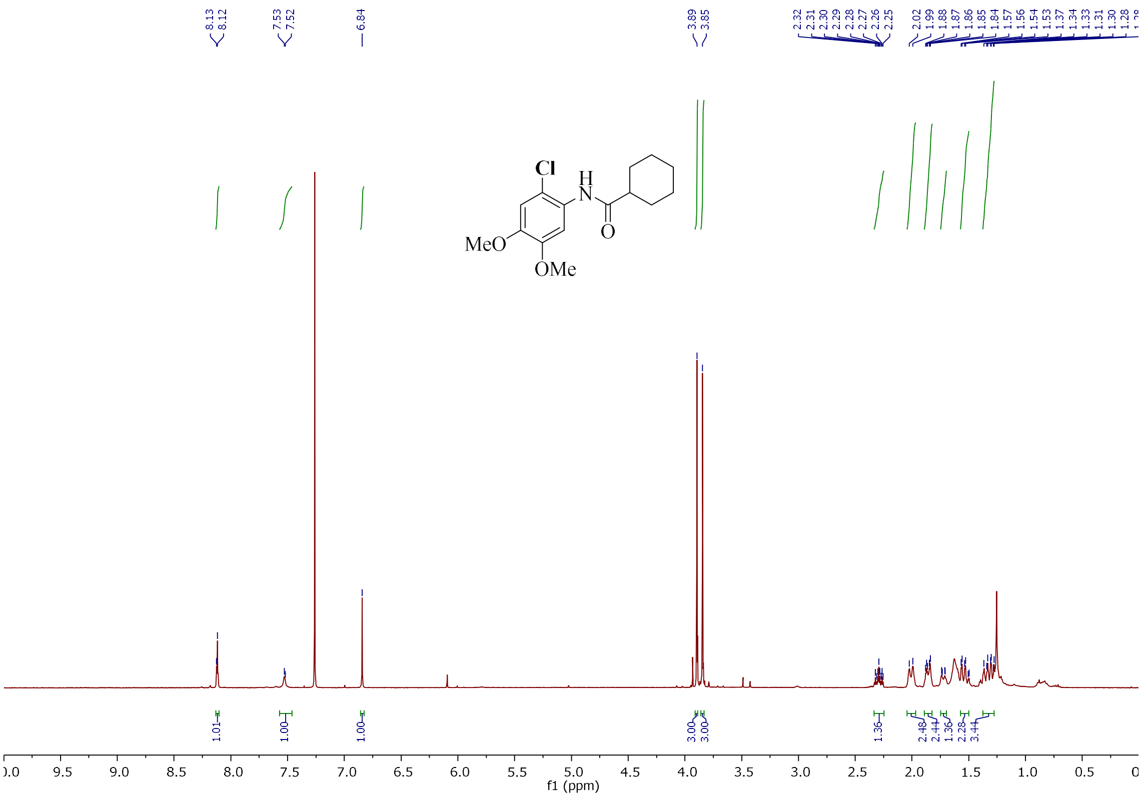

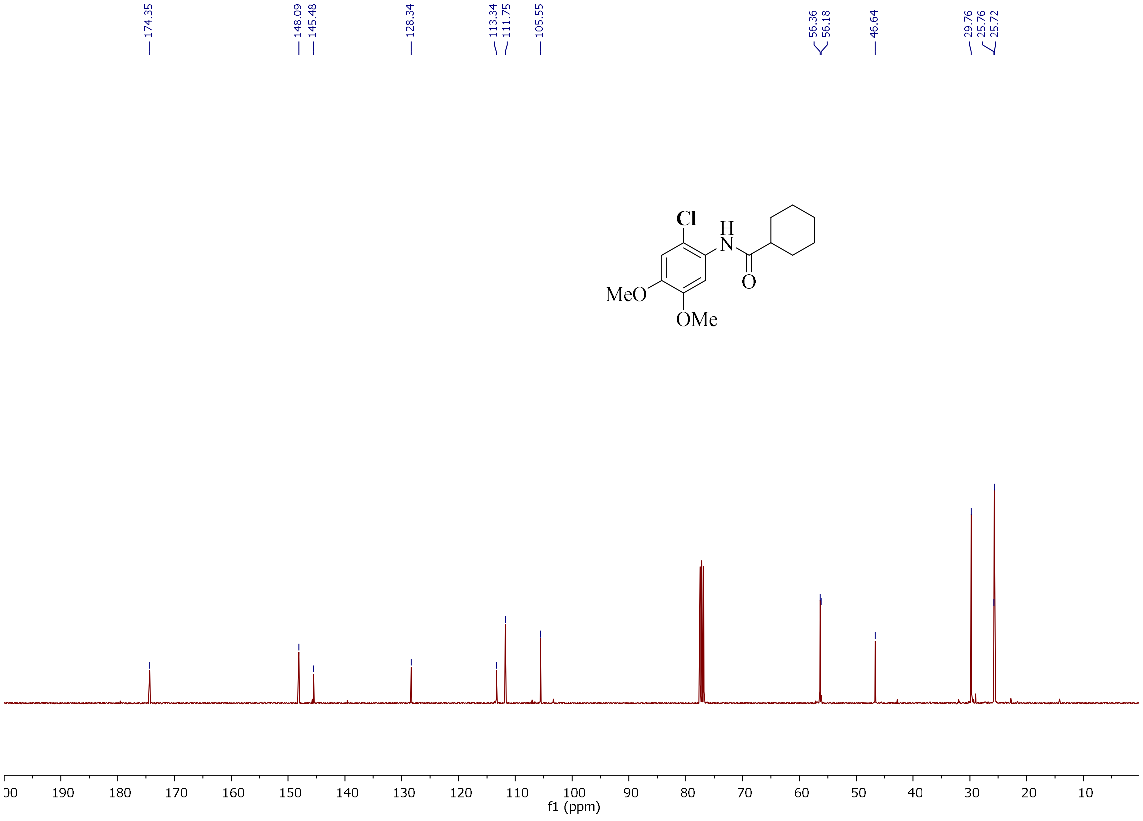

_N_-(2-chloro-3,4,5-trimethoxyphenyl)cyclohexanecarboxamide: (57)

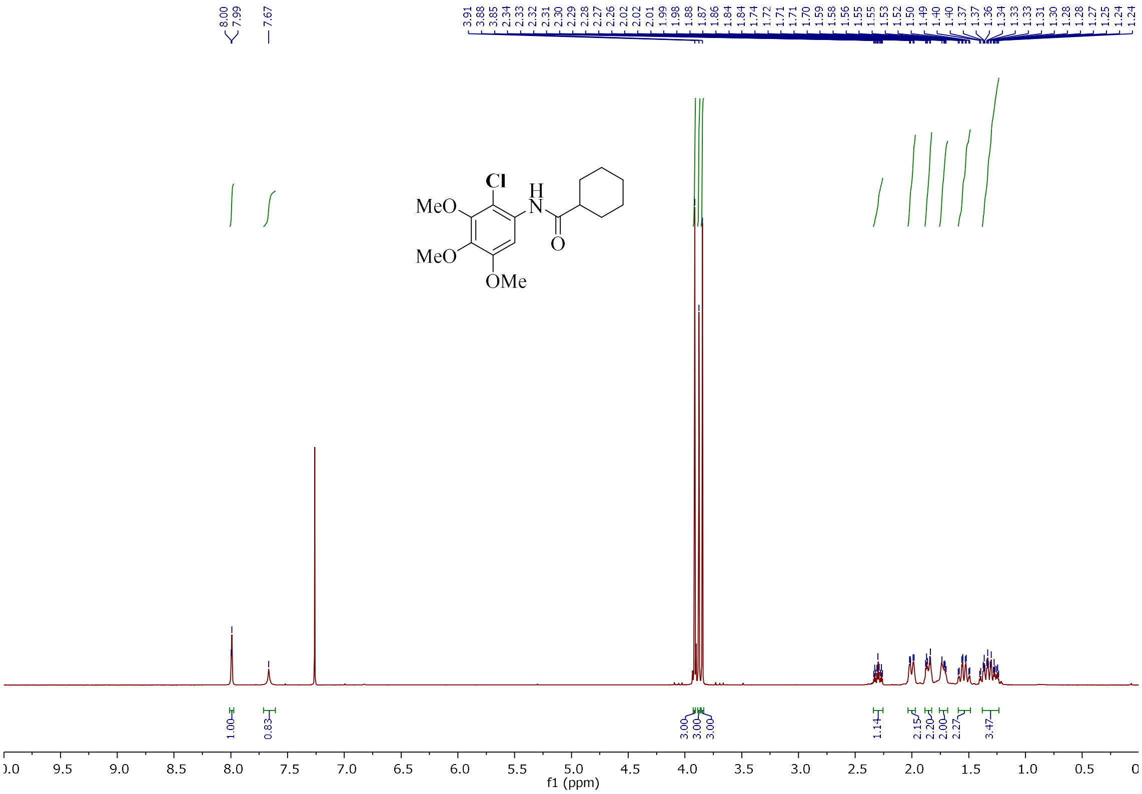

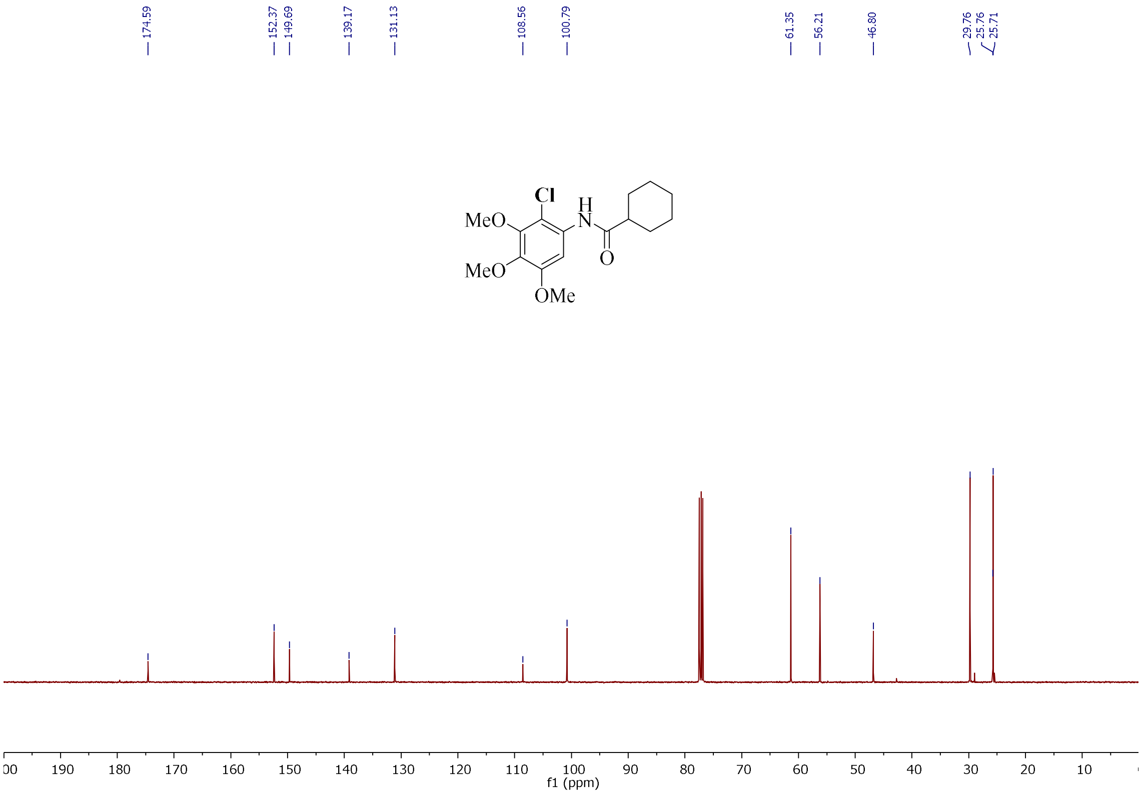

***N*-(5-chloro-4-methoxy-2-methylphenyl)cyclohexanecarboxamide: (58)**

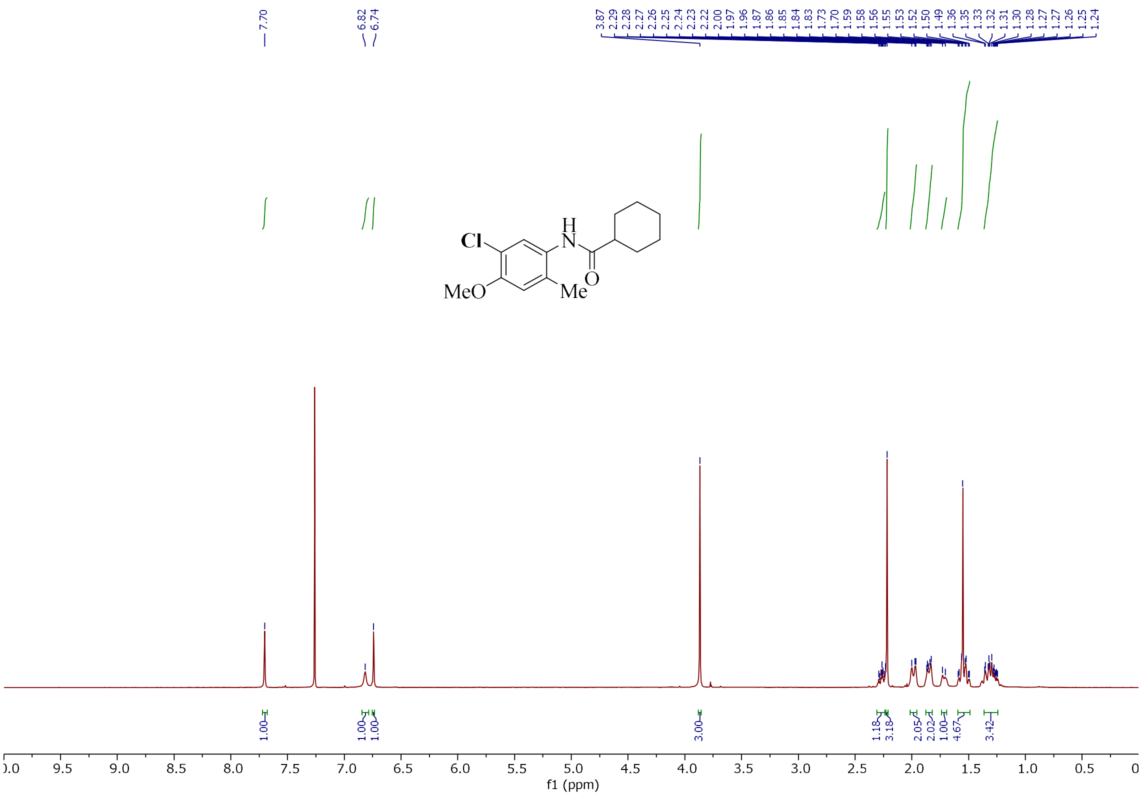

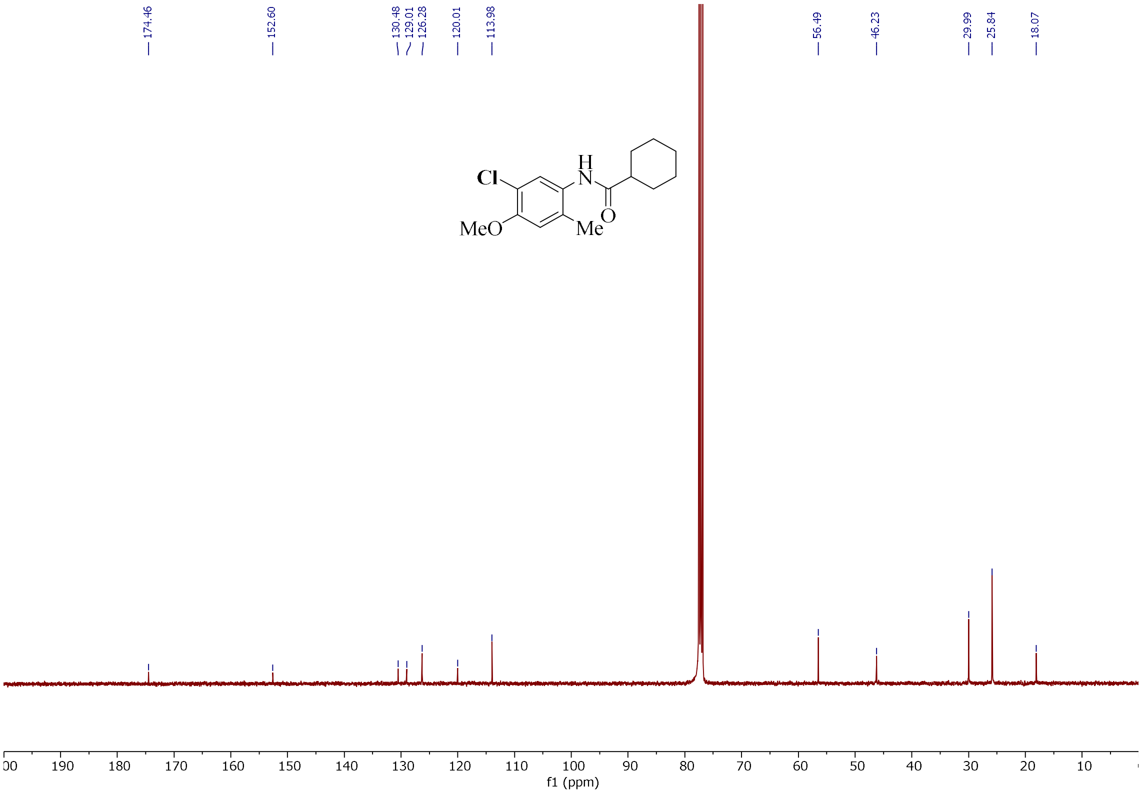

***N*-(3-chloro-4-methoxyphenyl)cyclohexanecarboxamide: (59)**

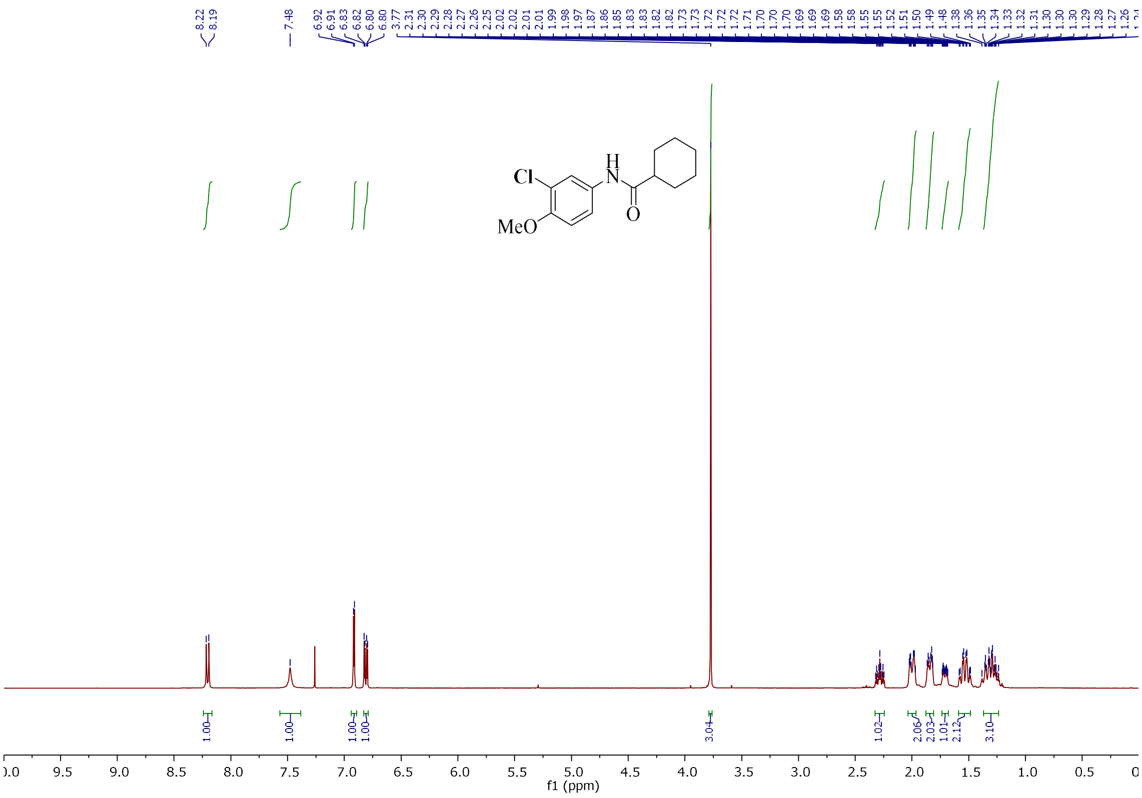

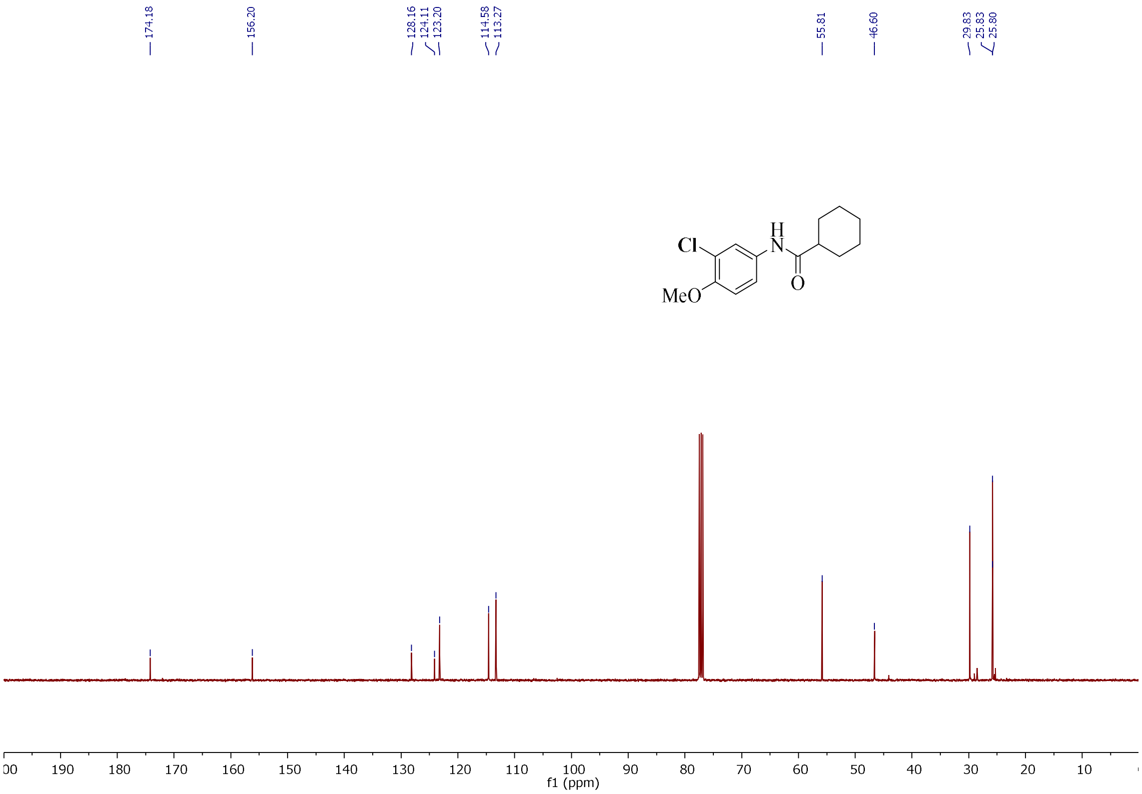

***N*-(2-chloro-4-methylphenyl)cyclohexanecarboxamide: (60)**

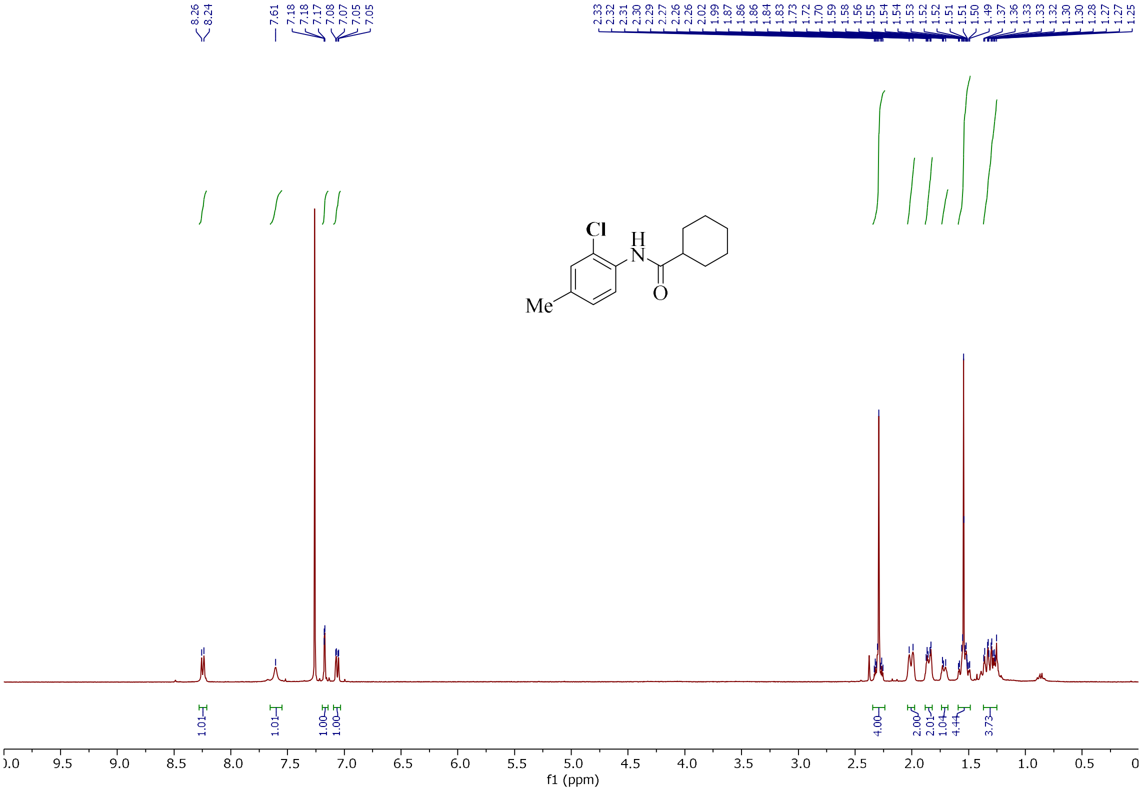

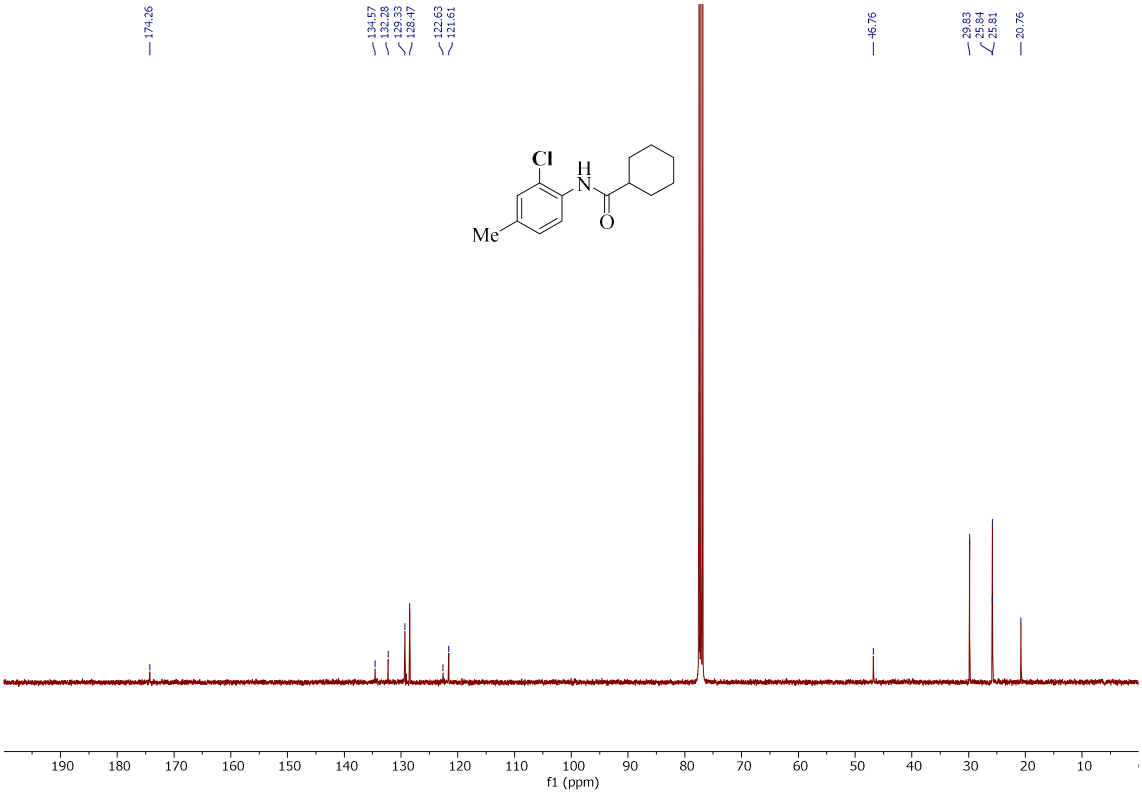

_N_-(2-chloro-4-fluorophenyl)cyclohexanecarboxamide: (61)

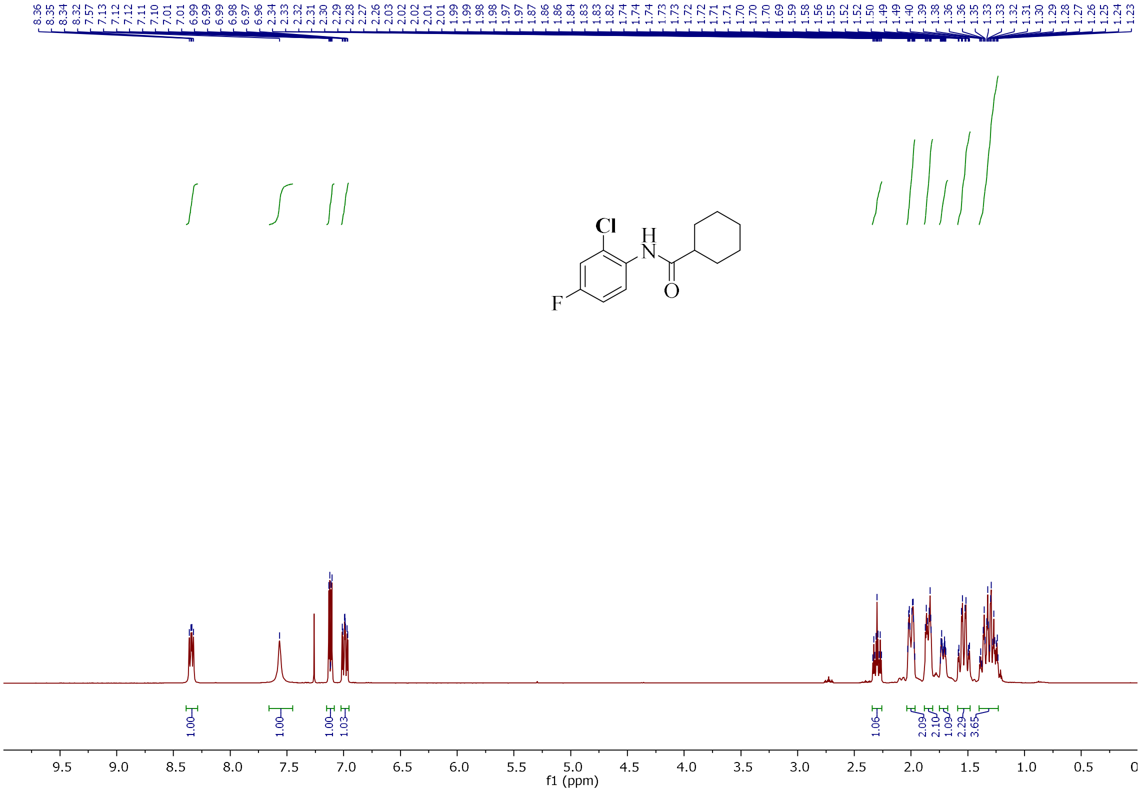

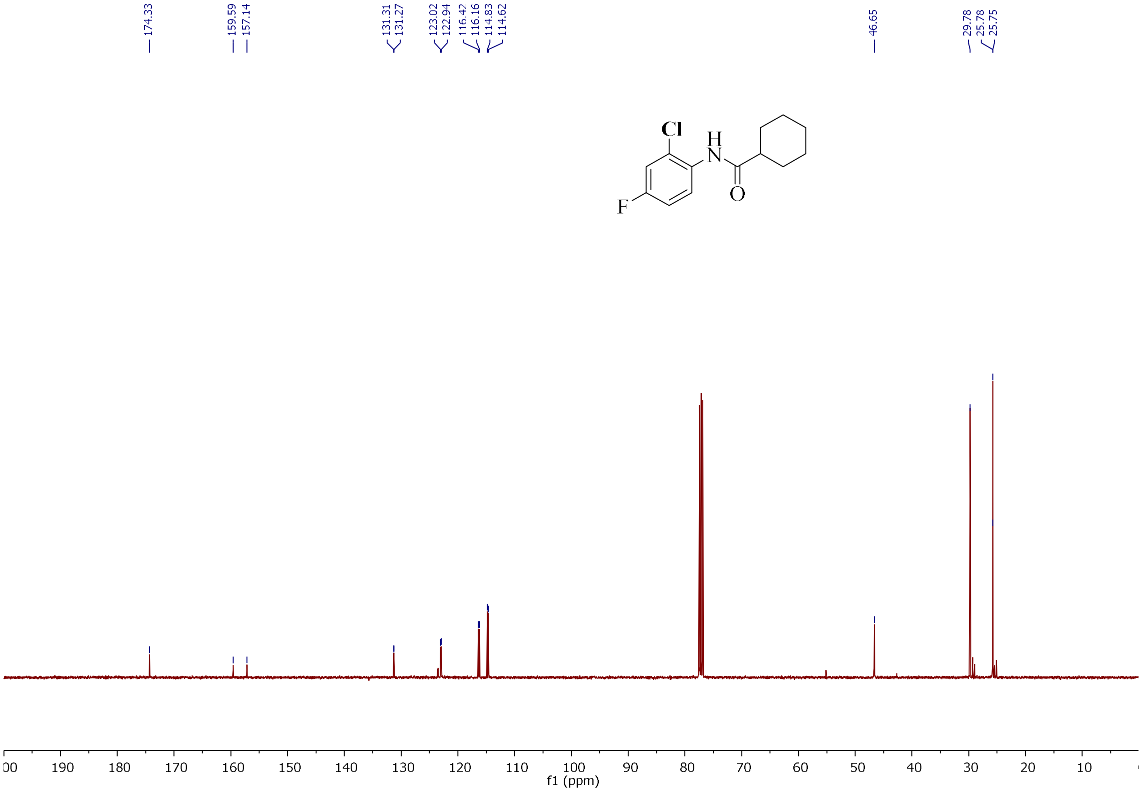

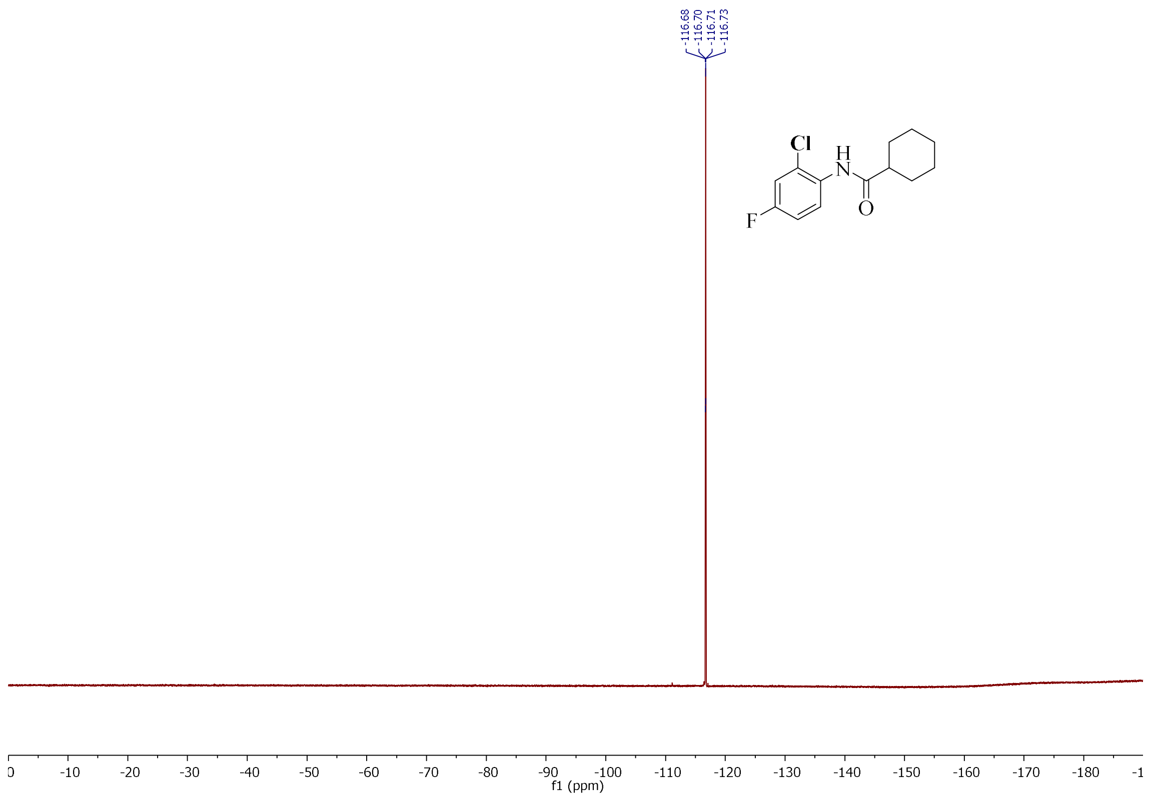

-116.68
-116.70
-116.71
-116.73
Cl
H
N
O
F
0
-10
-20
-30
-40
-50
-60
-70
-80
-90
-100
-110
-120
-130
-140
-150
-160
-170
-180
f1 (ppm)

***N*-(2,4-dichlorophenyl)cyclohexanecarboxamide: (4)**

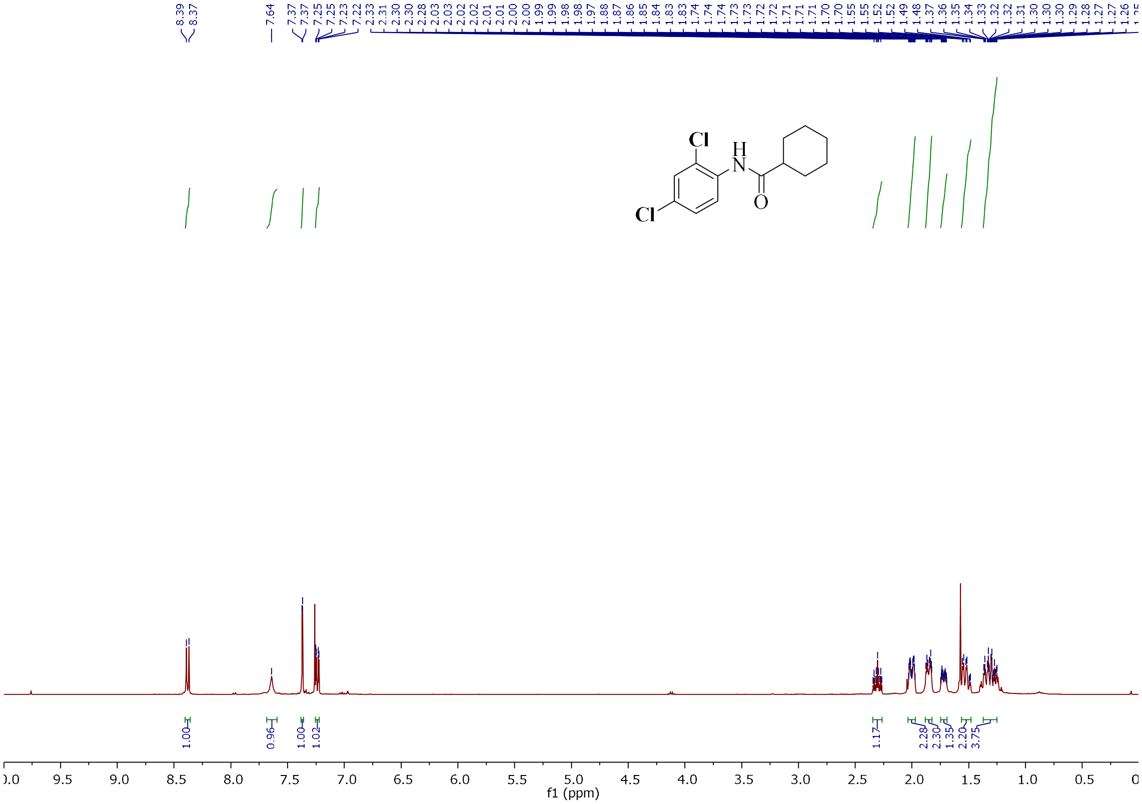

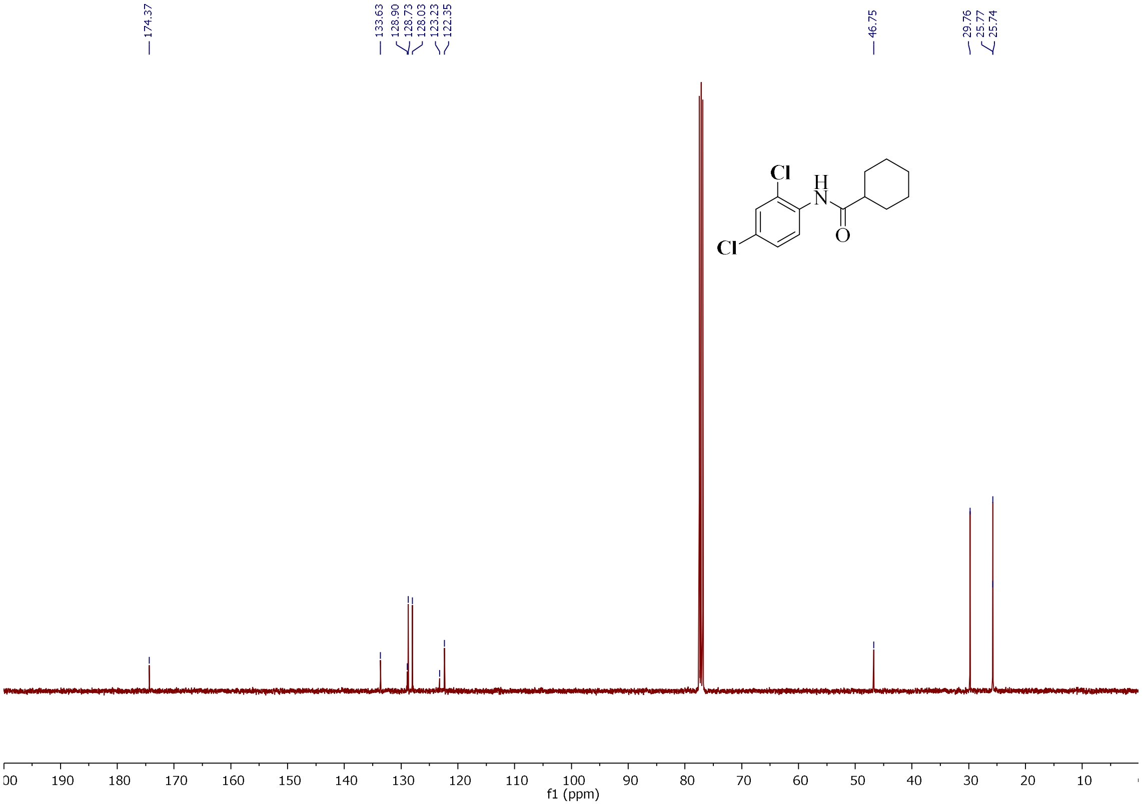

***N*-(2-chloro-3,5-dimethylphenyl)cyclohexanecarboxamide: (62)**

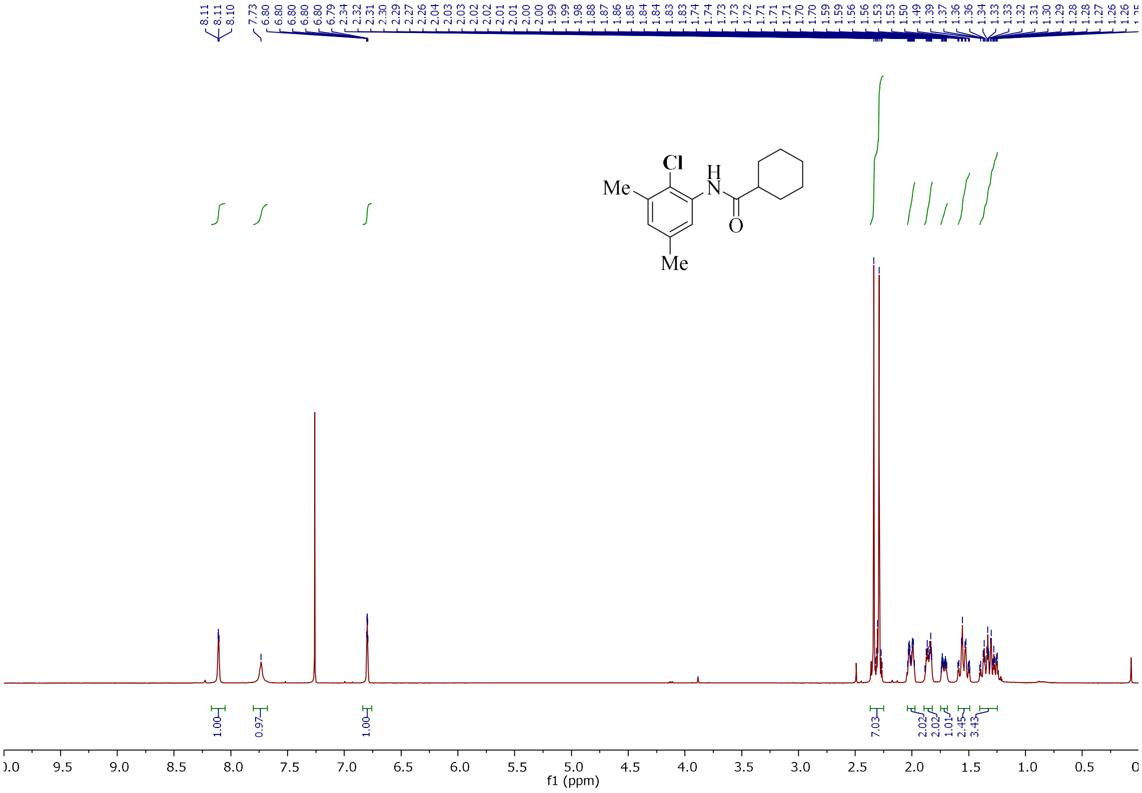

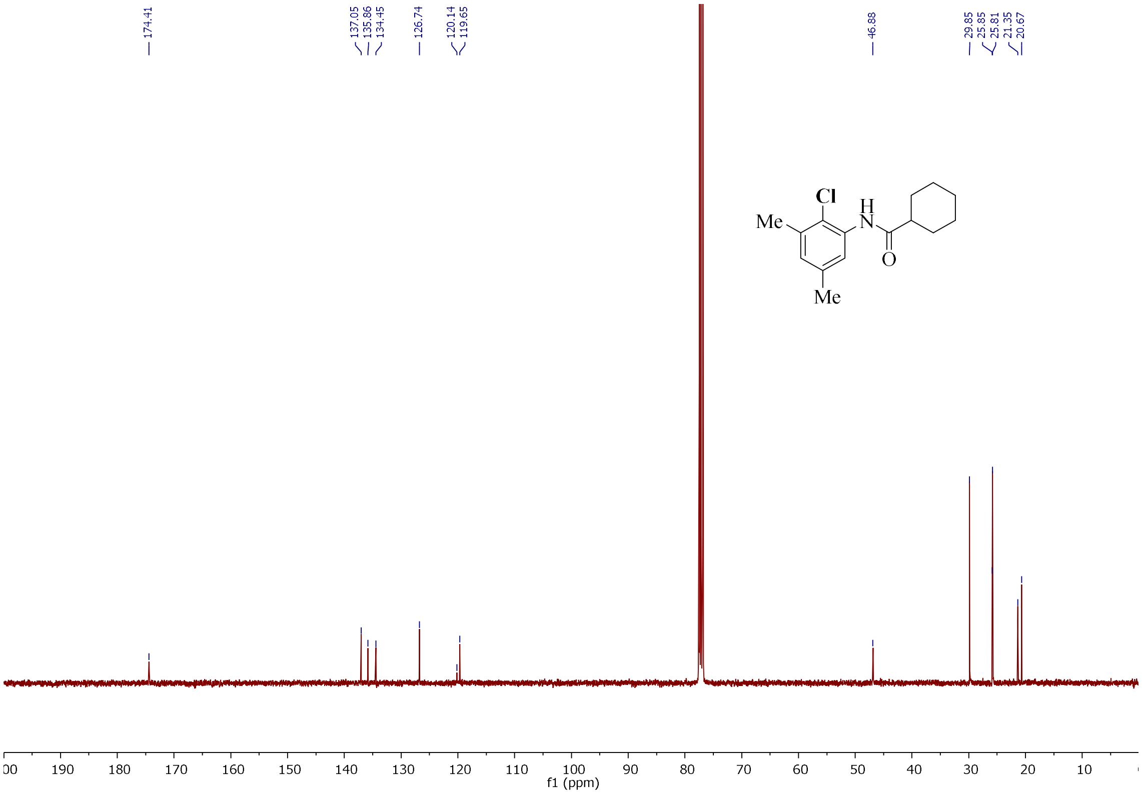

N-(2-chloro-3,5-dimethoxyphenyl)cyclohexanecarboxamide: (63)

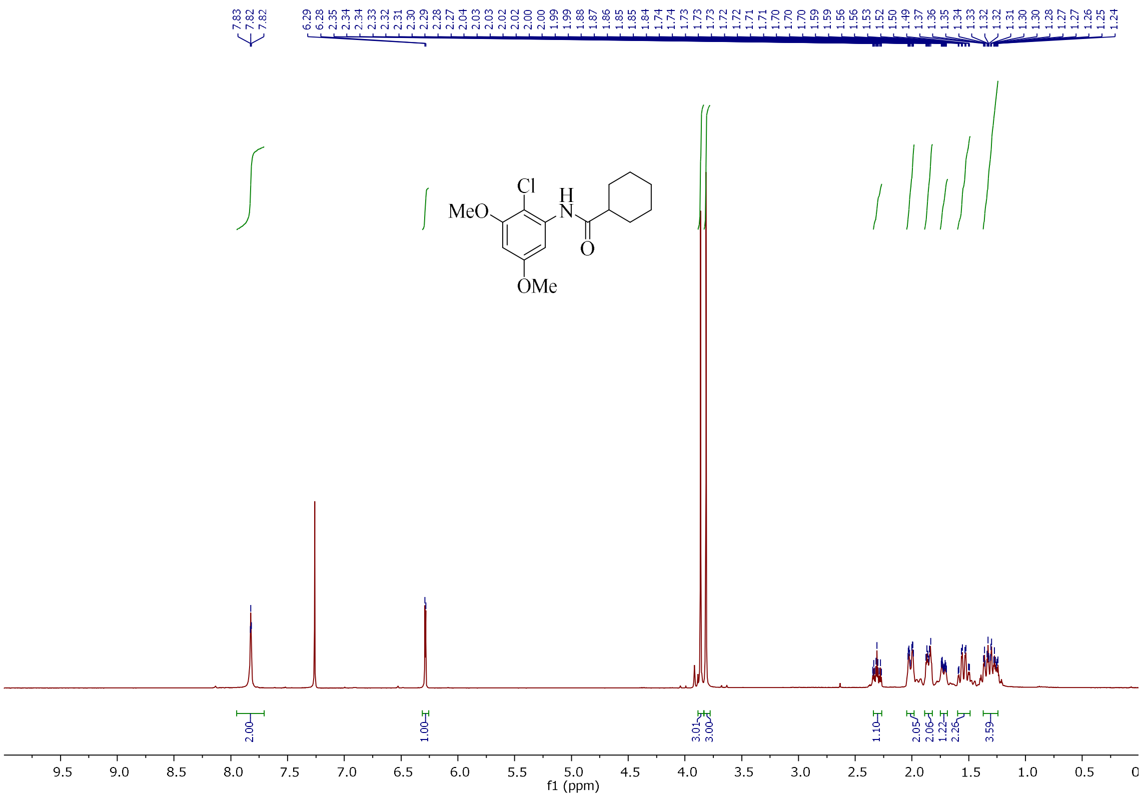

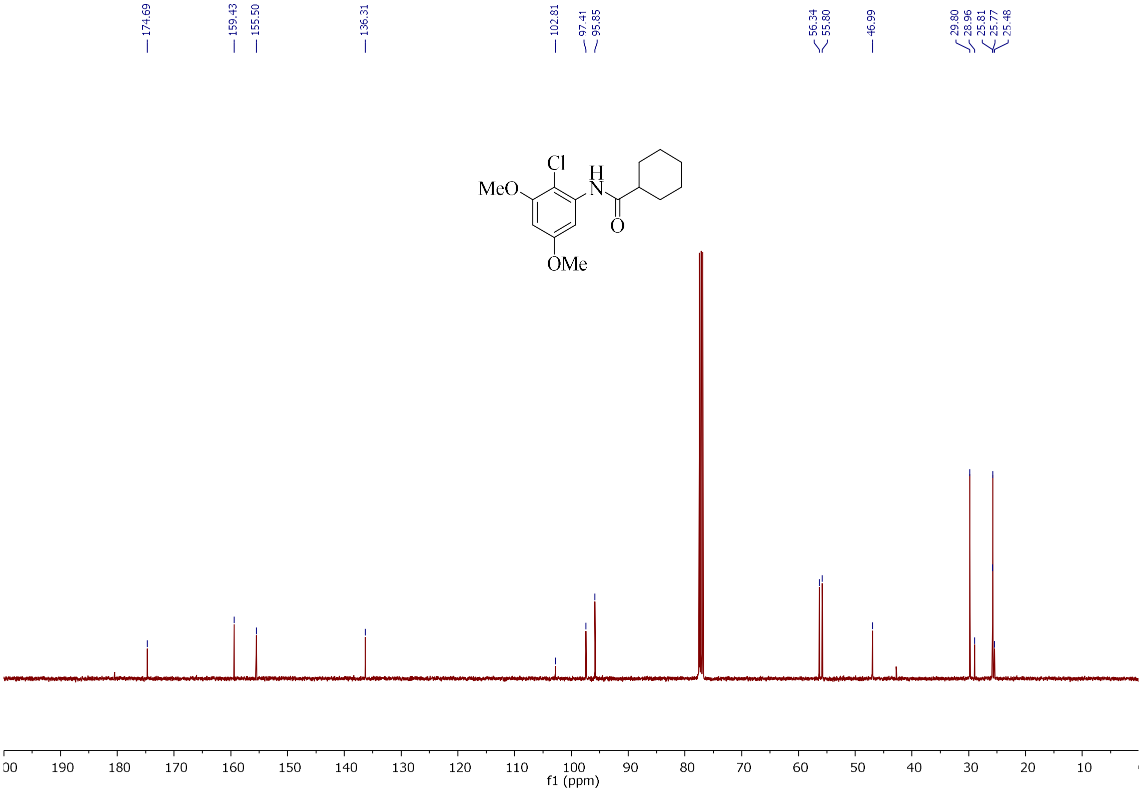

***N*-(4-bromo-2-chloronaphthalen-1-yl)cyclohexanecarboxamide: (64)**

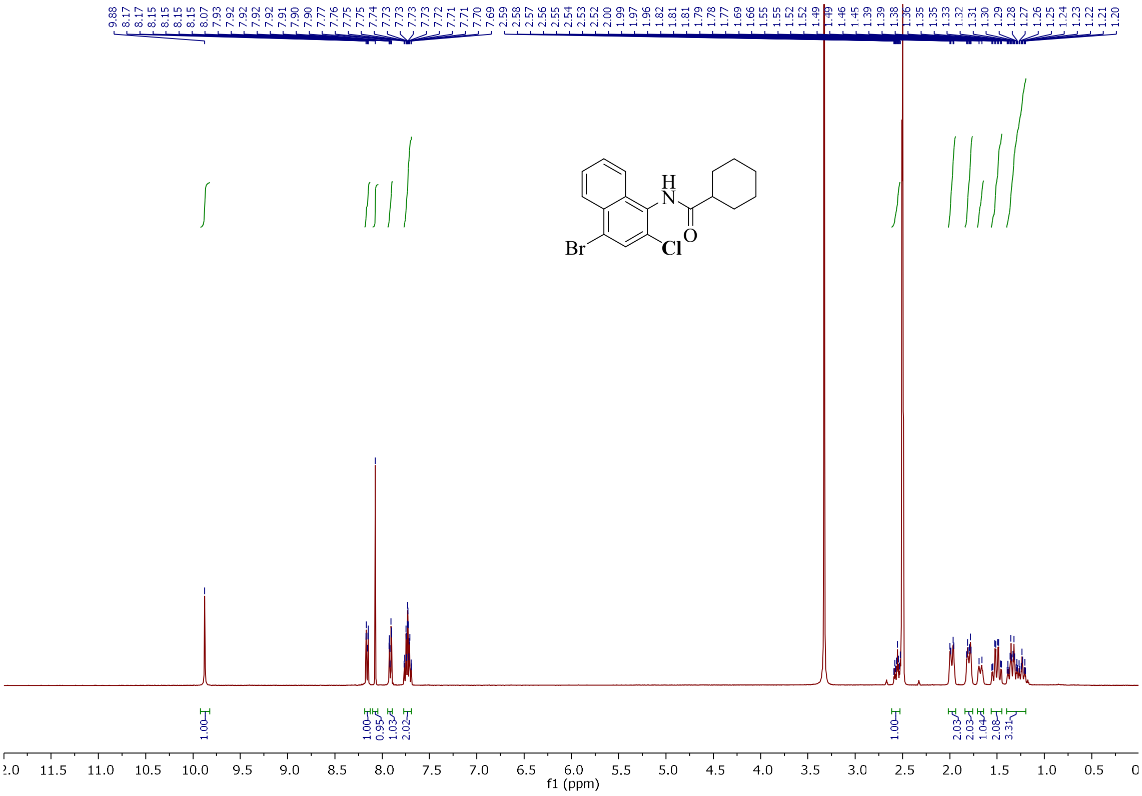

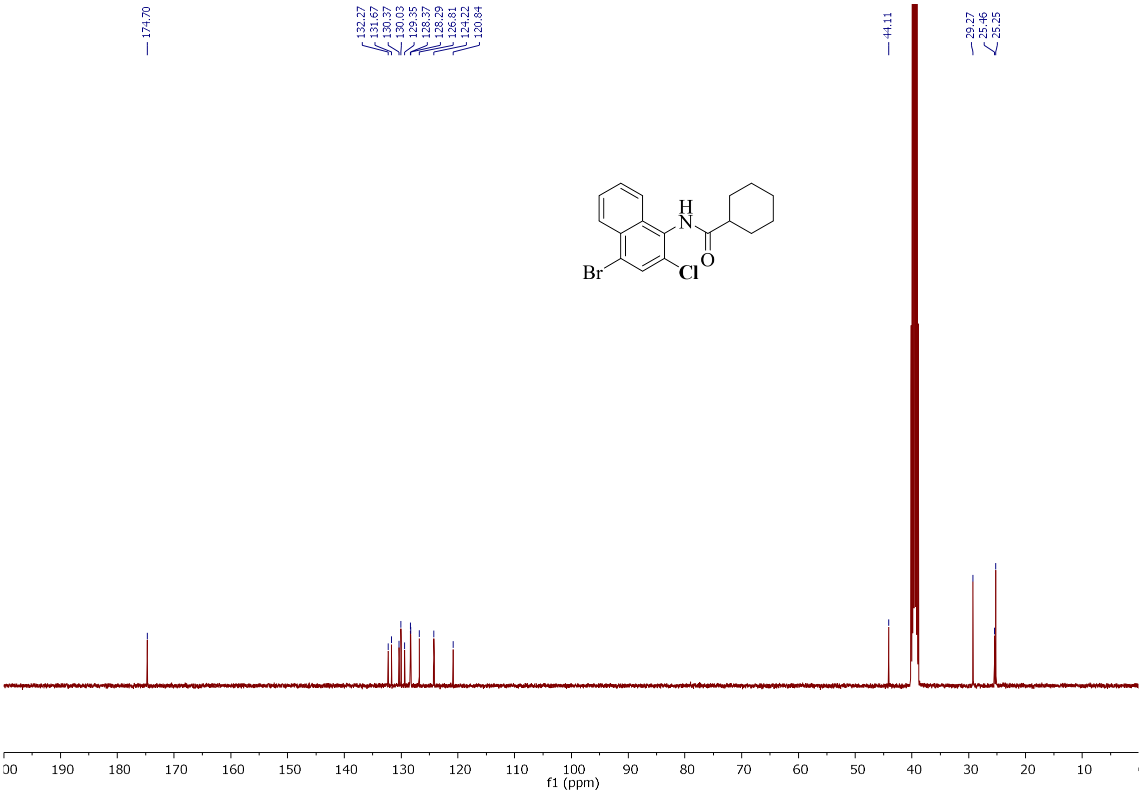

N-(1-chloronaphthalen-2-yl)cyclohexanecarboxamide: (65)

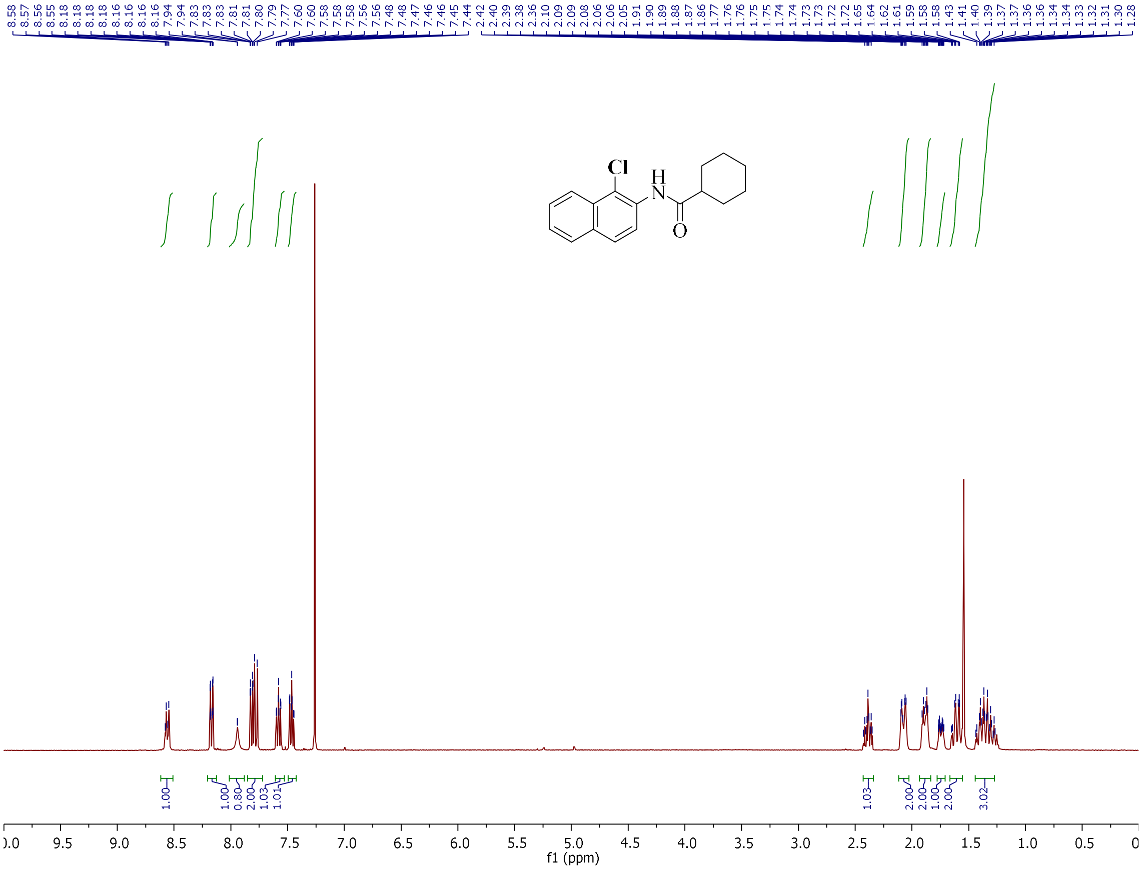

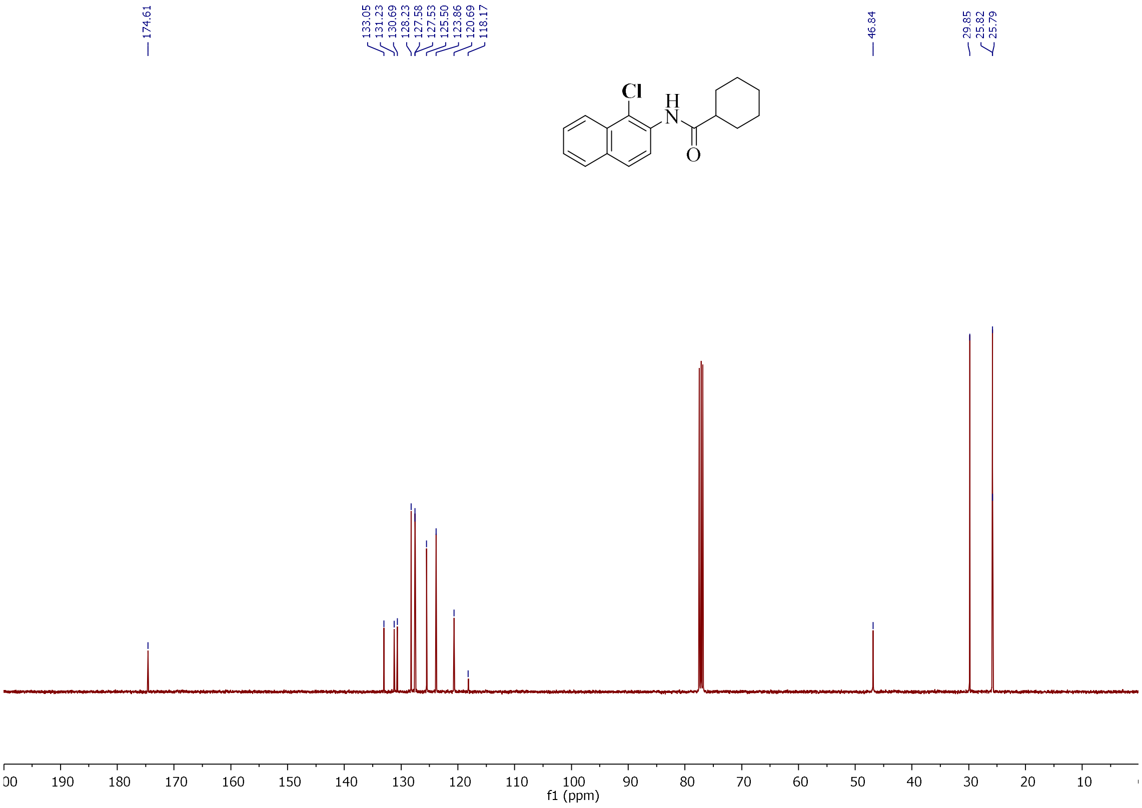

N-(1-chloronaphthalen-2-yl)acetamide: (66)

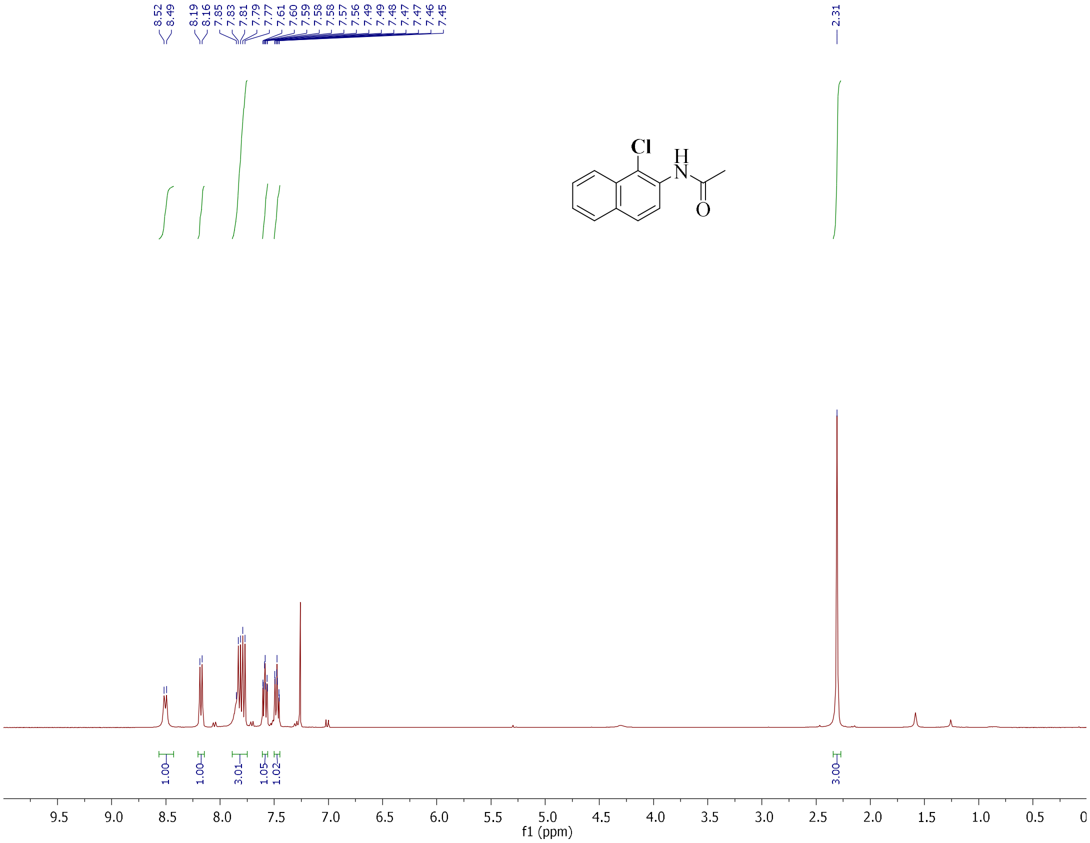

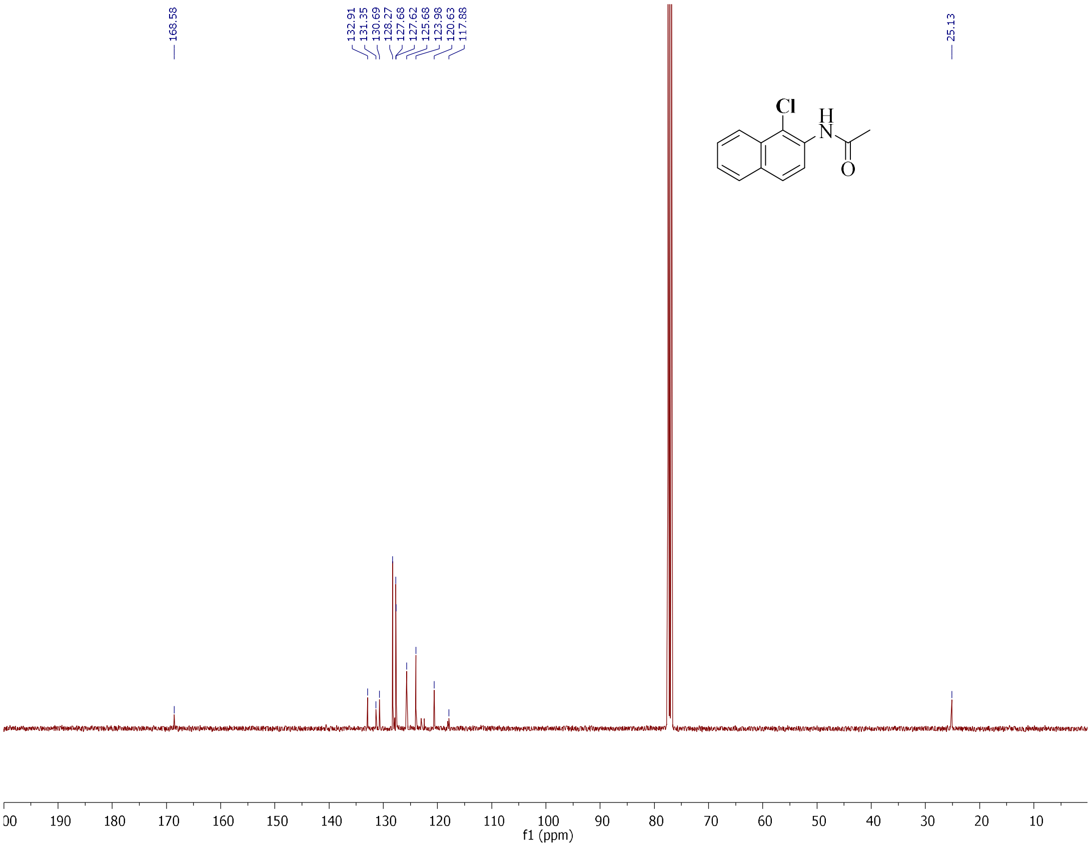

_N_-(1-chloronaphthalen-2-yl)-2-phenylacetamide: (67)

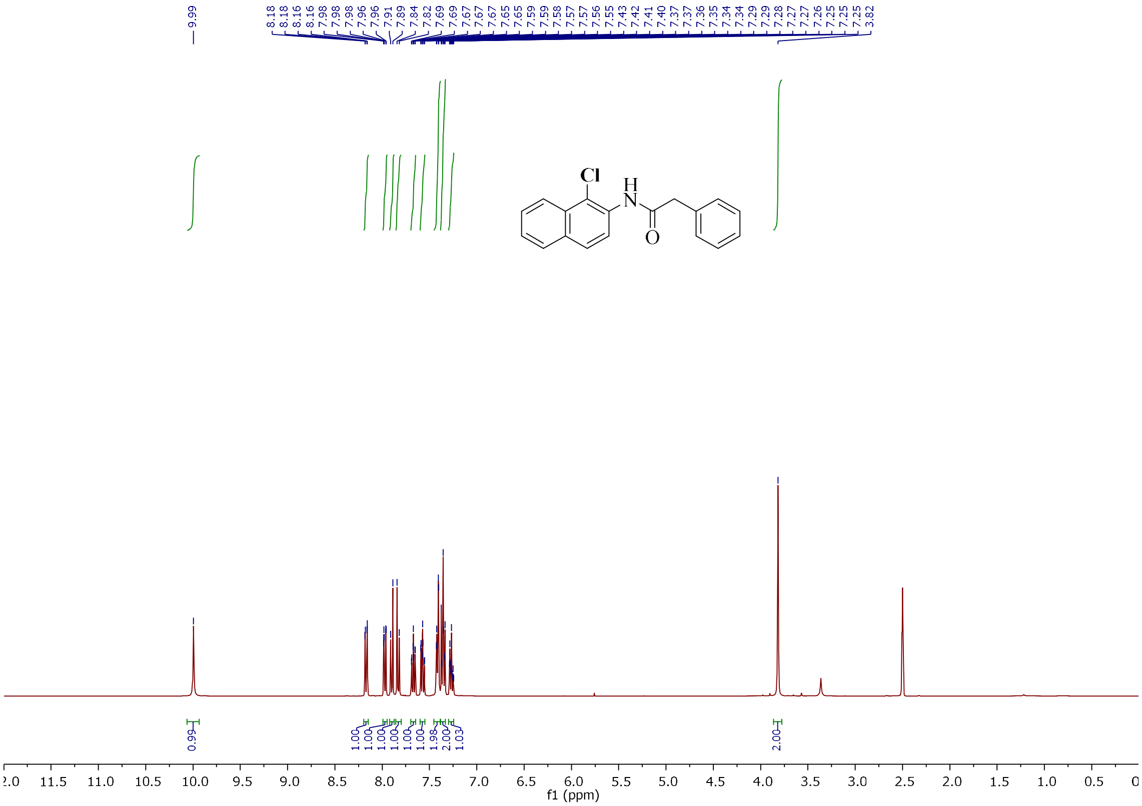

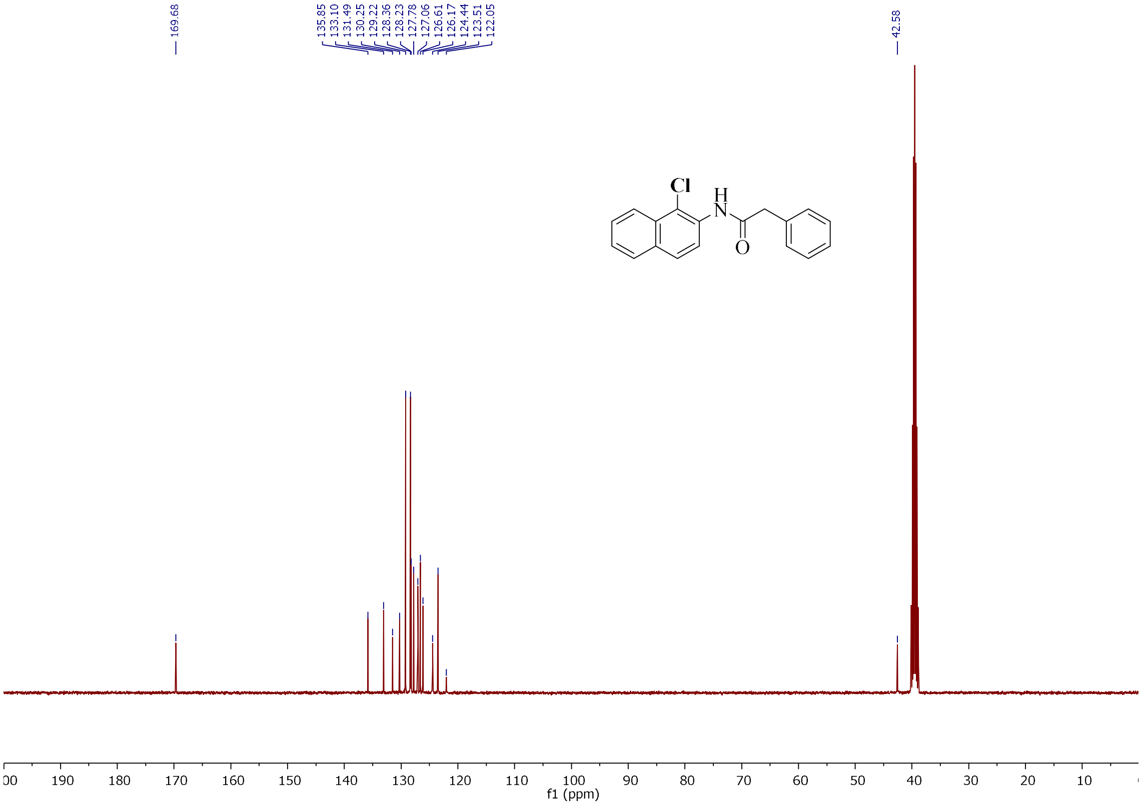

_N_-(1-chloronaphthalen-2-yl)benzamide: (68)

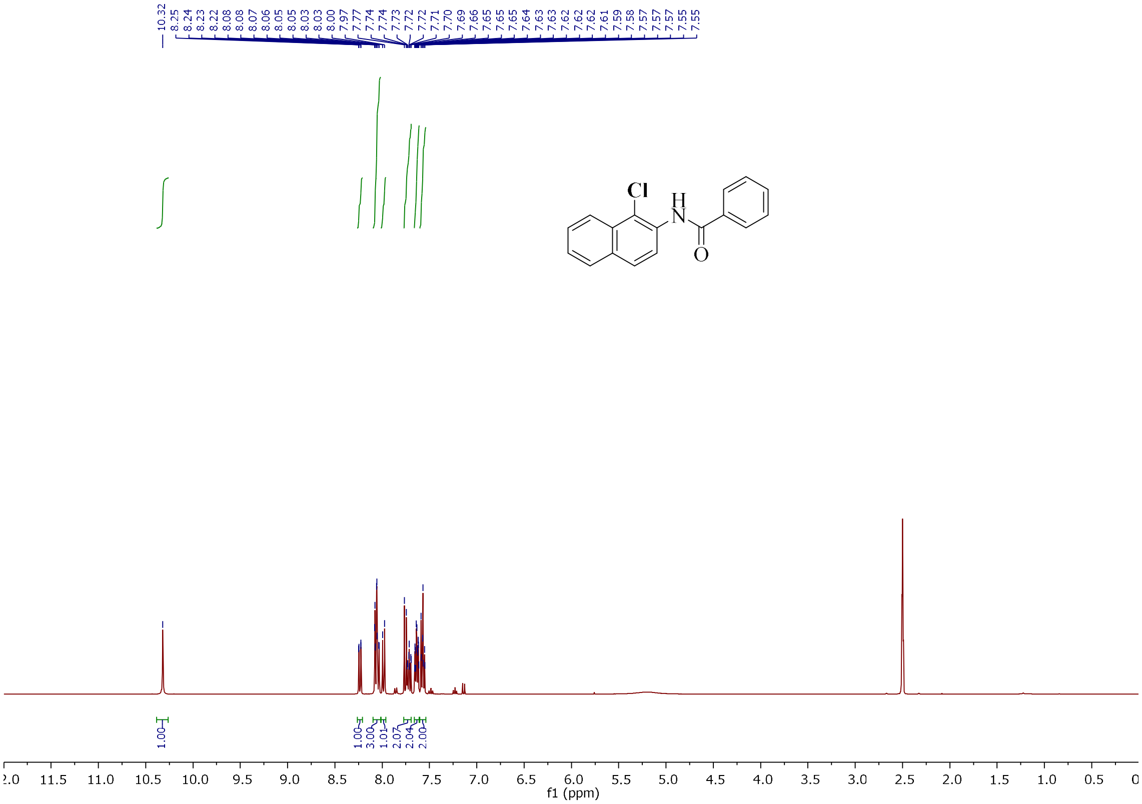

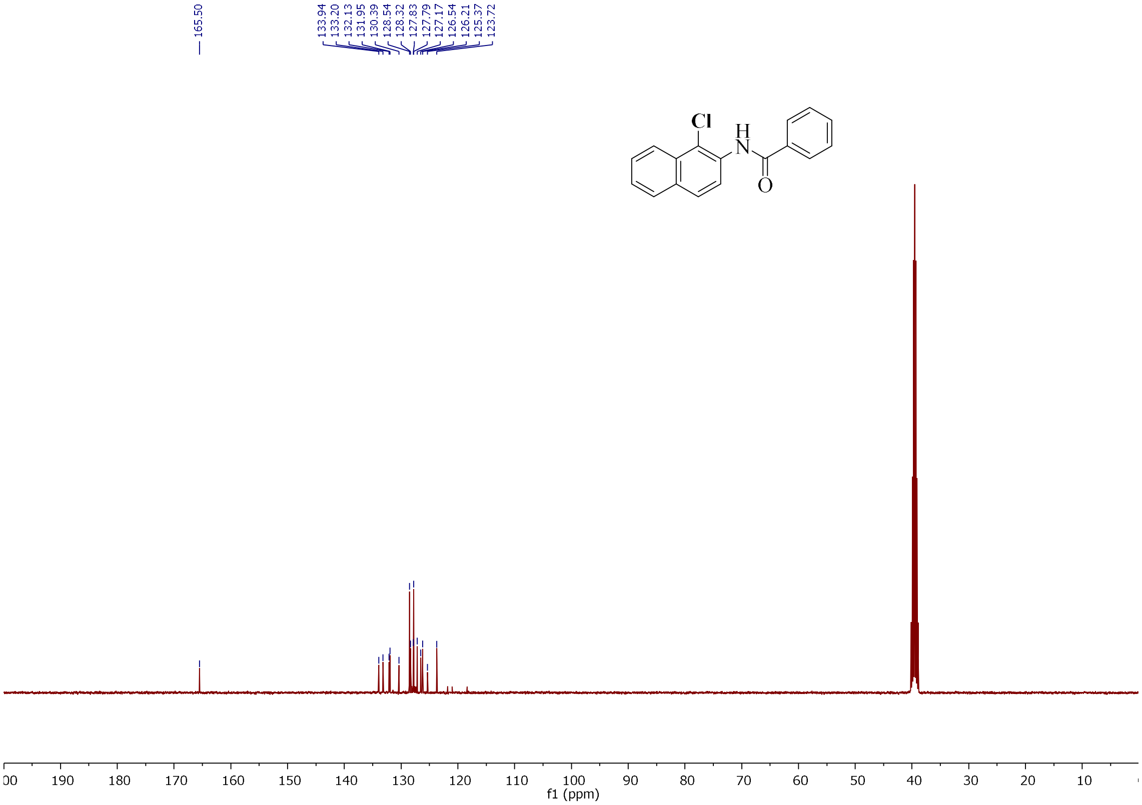

***N*-(1-chloronaphthalen-2-yl)-1-naphthamide: (69)**

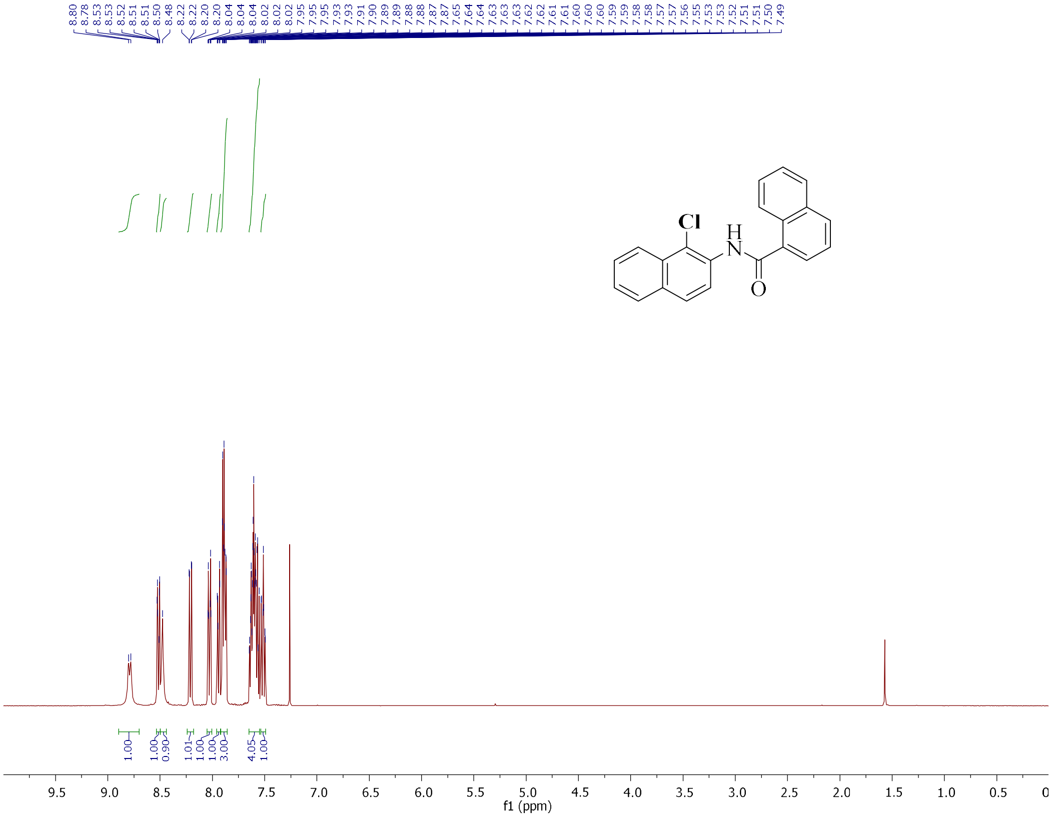

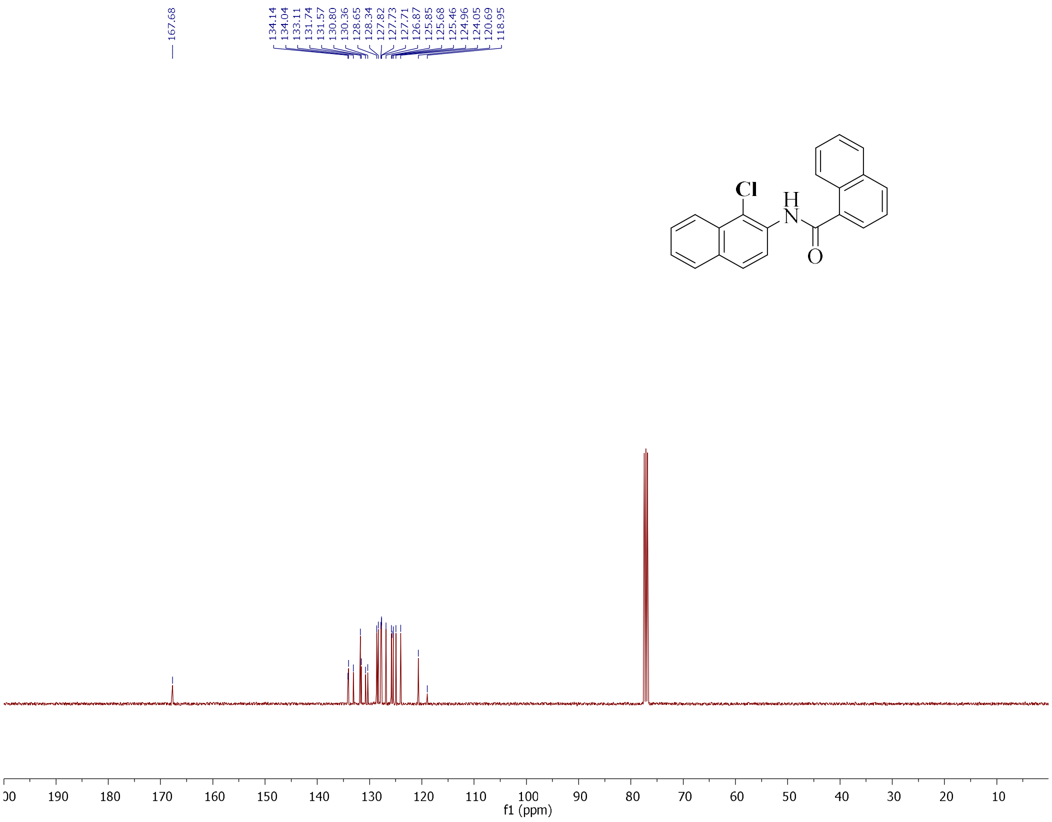

2-chloro-*N*-(1-chloronaphthalen-2-yl)acetamide: (70)

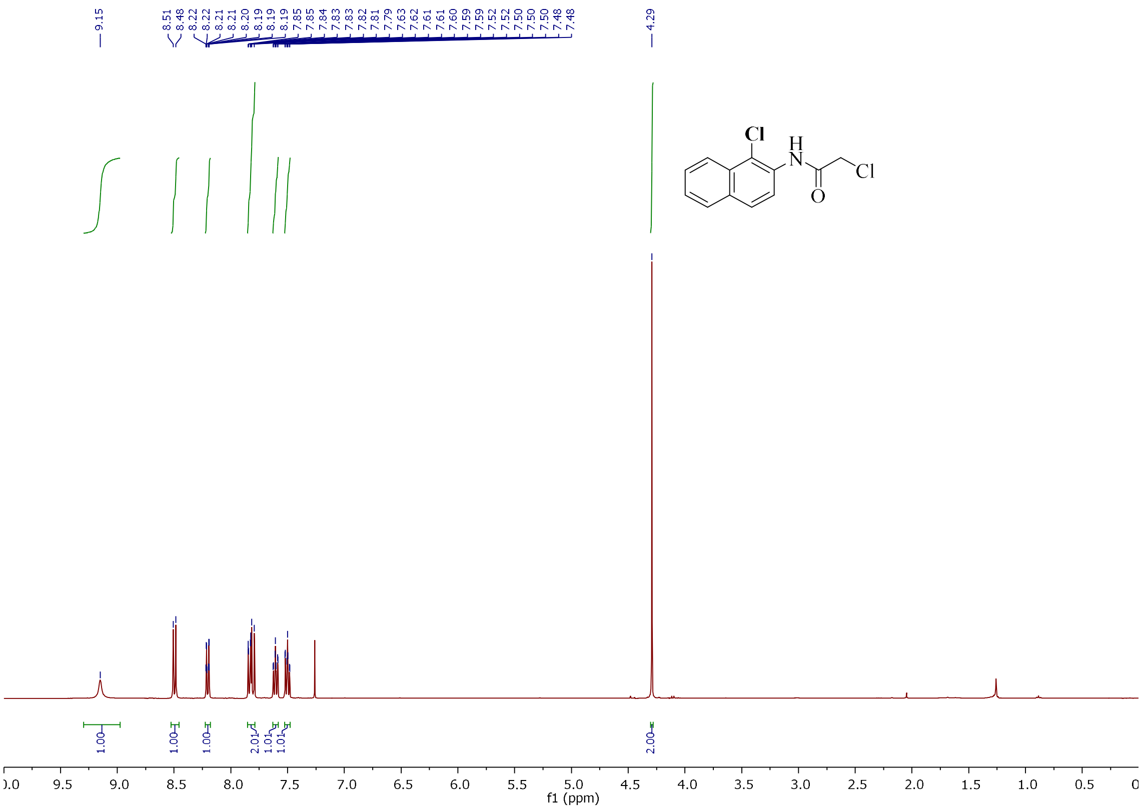

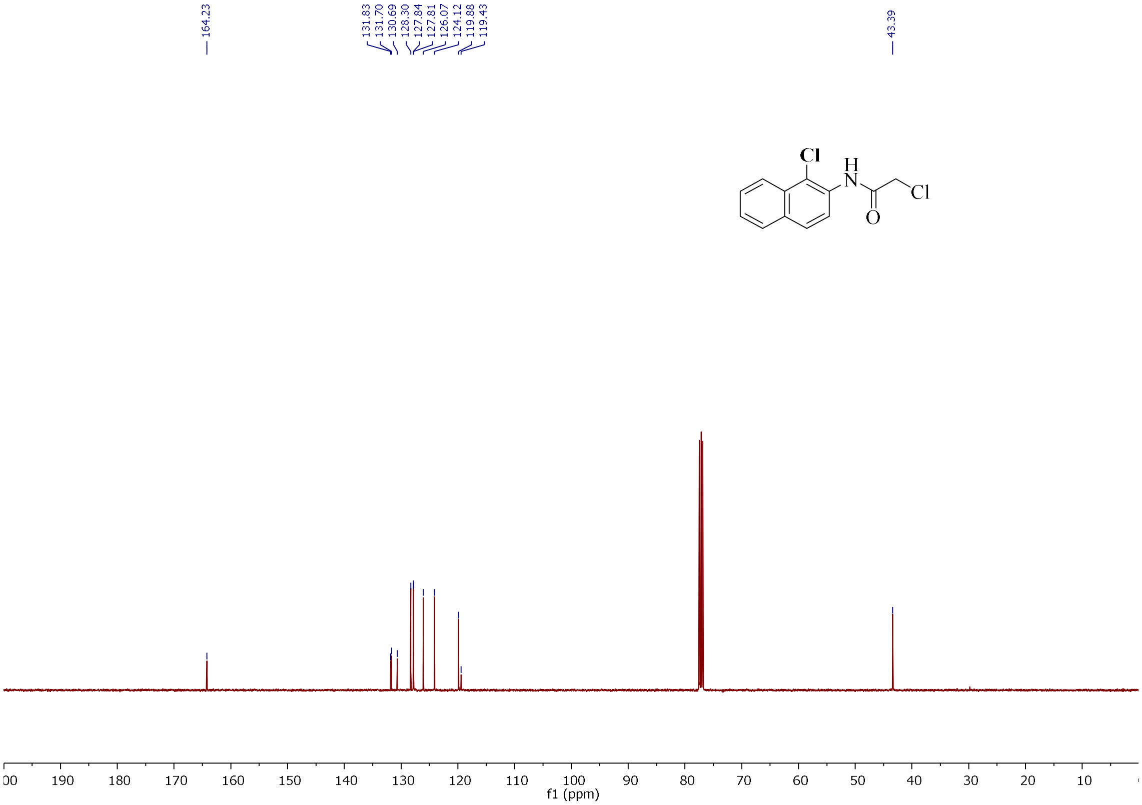

5-chloro-*N*-(1-chloronaphthalen-2-yl)pentanamide: (71)

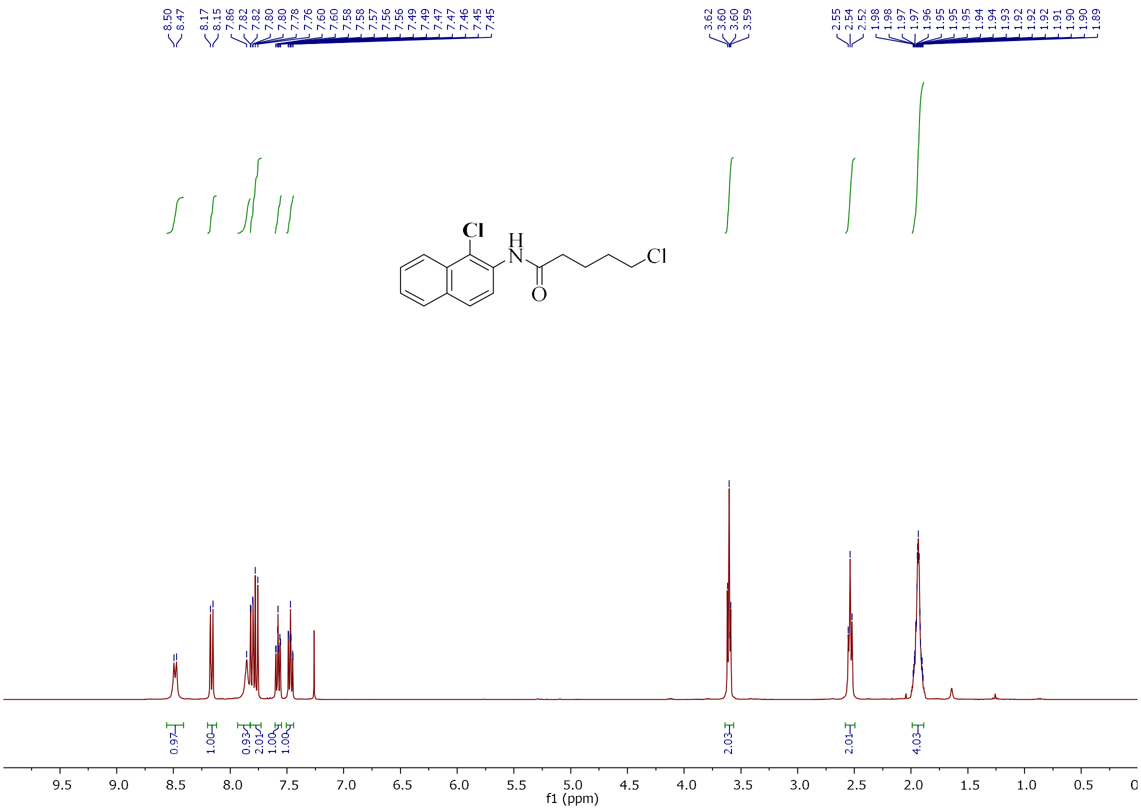

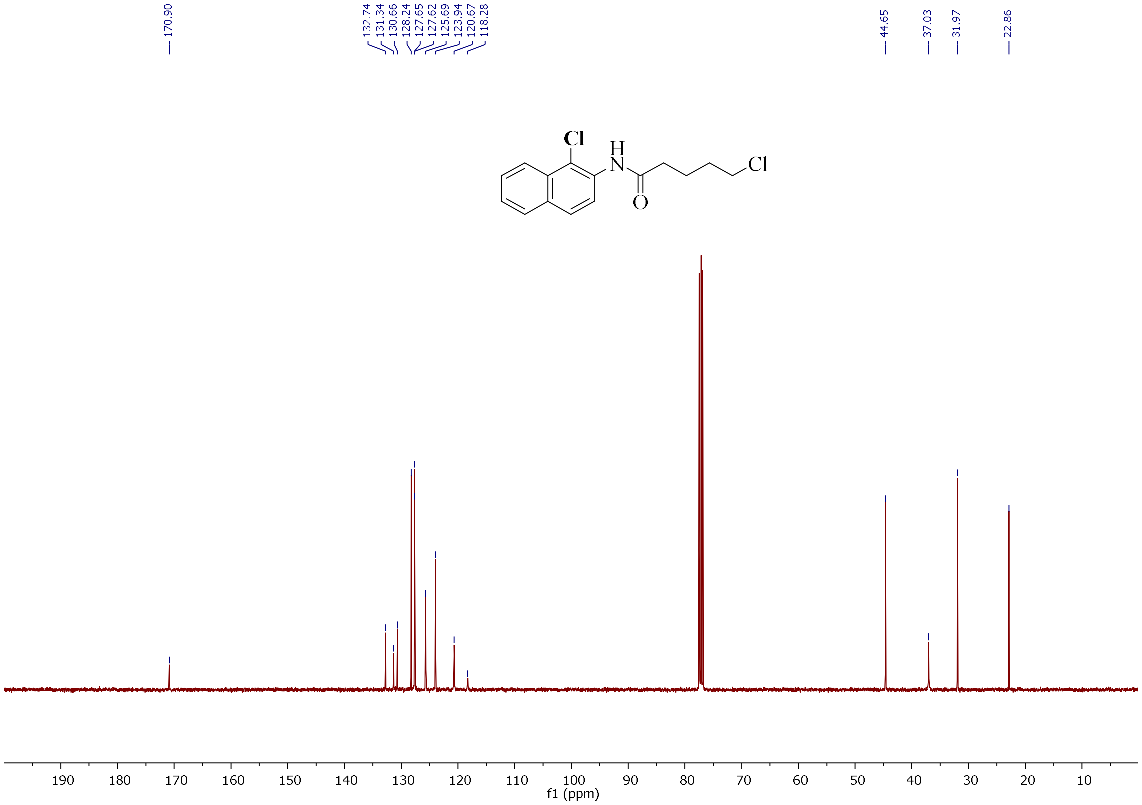

_N_-(1-chloronaphthalen-2-yl)benzo[_b_]thiophene-2-carboxamide: (72)

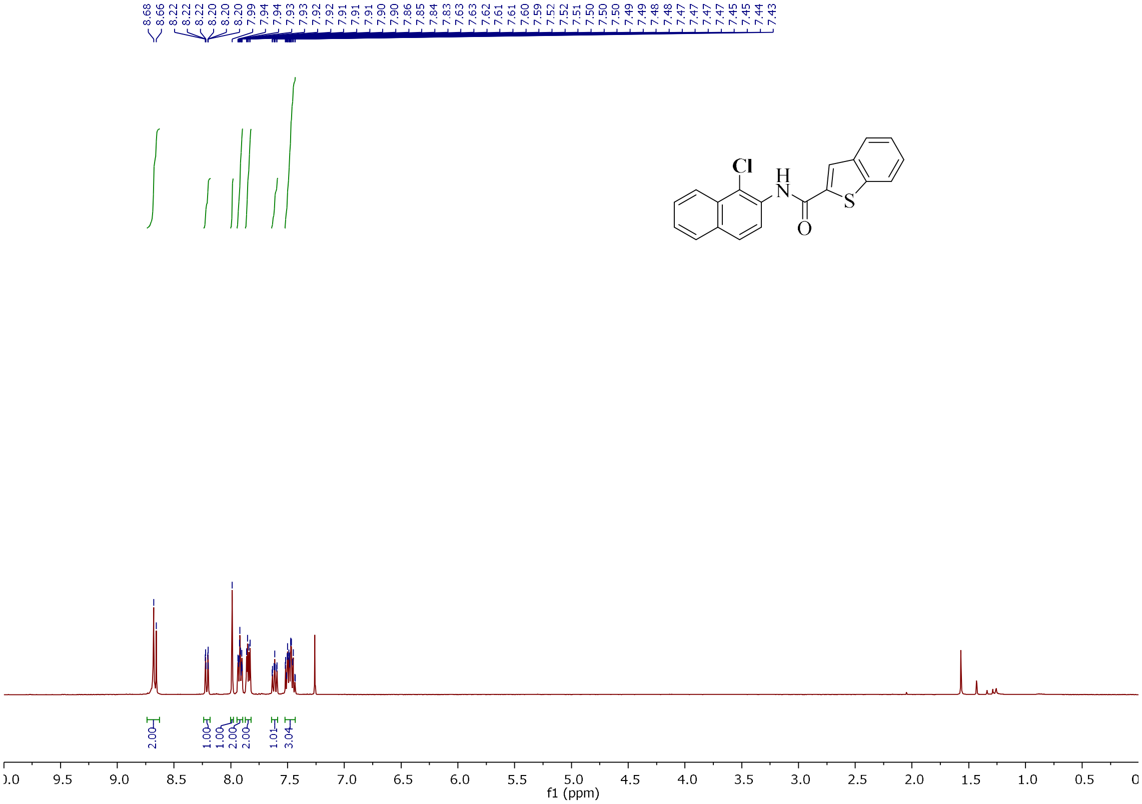

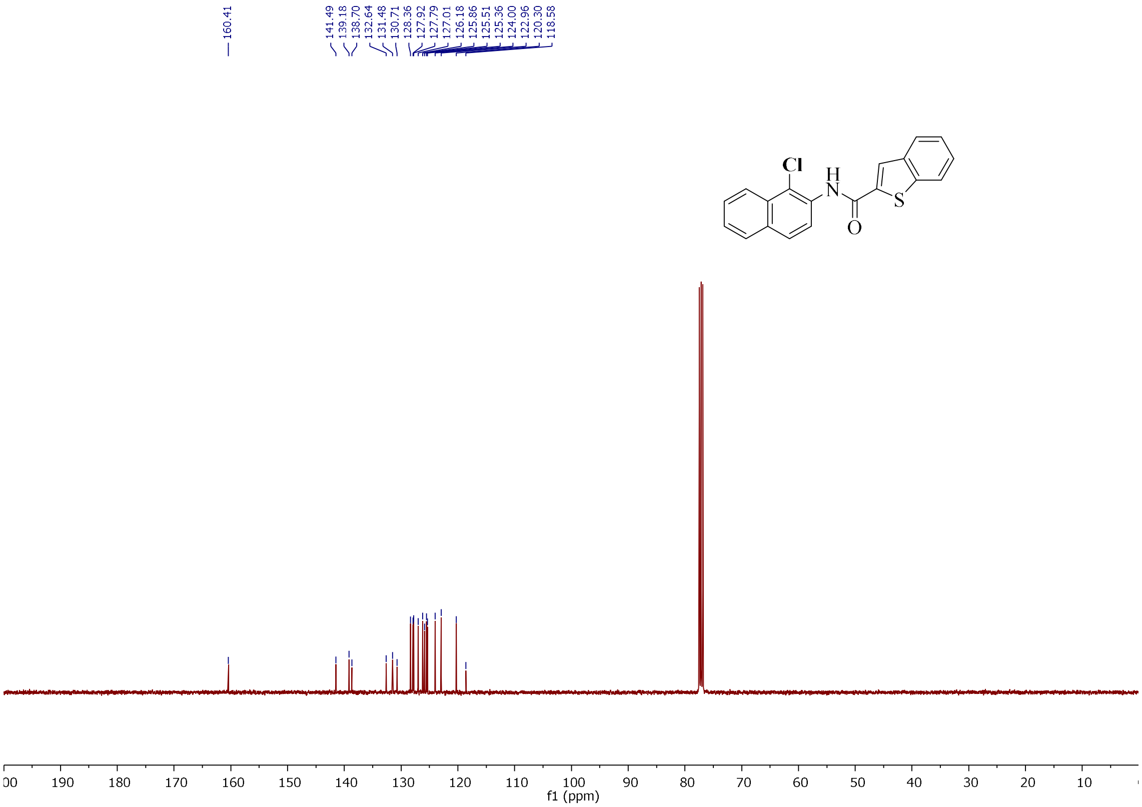

Ethyl (1-chloronaphthalen-2-yl)carbamate: (73)

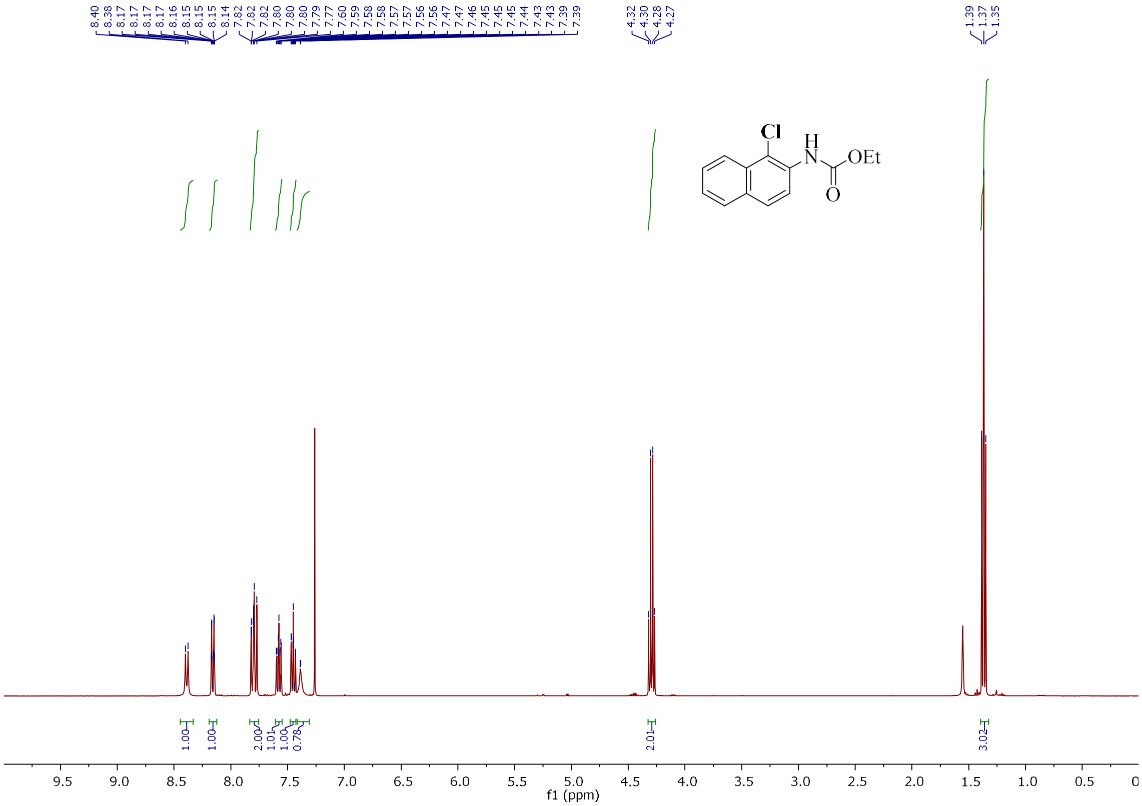

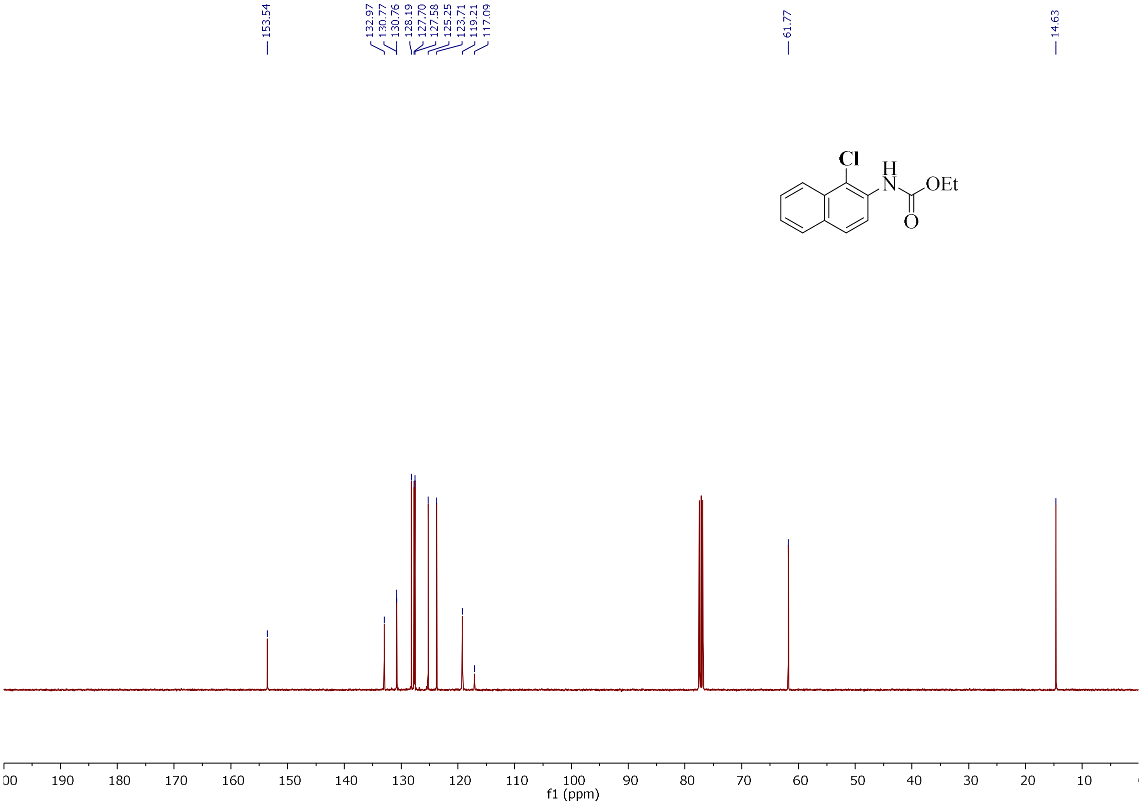

Ethyl (3-chloro-4-methoxyphenyl)carbamate: (74)

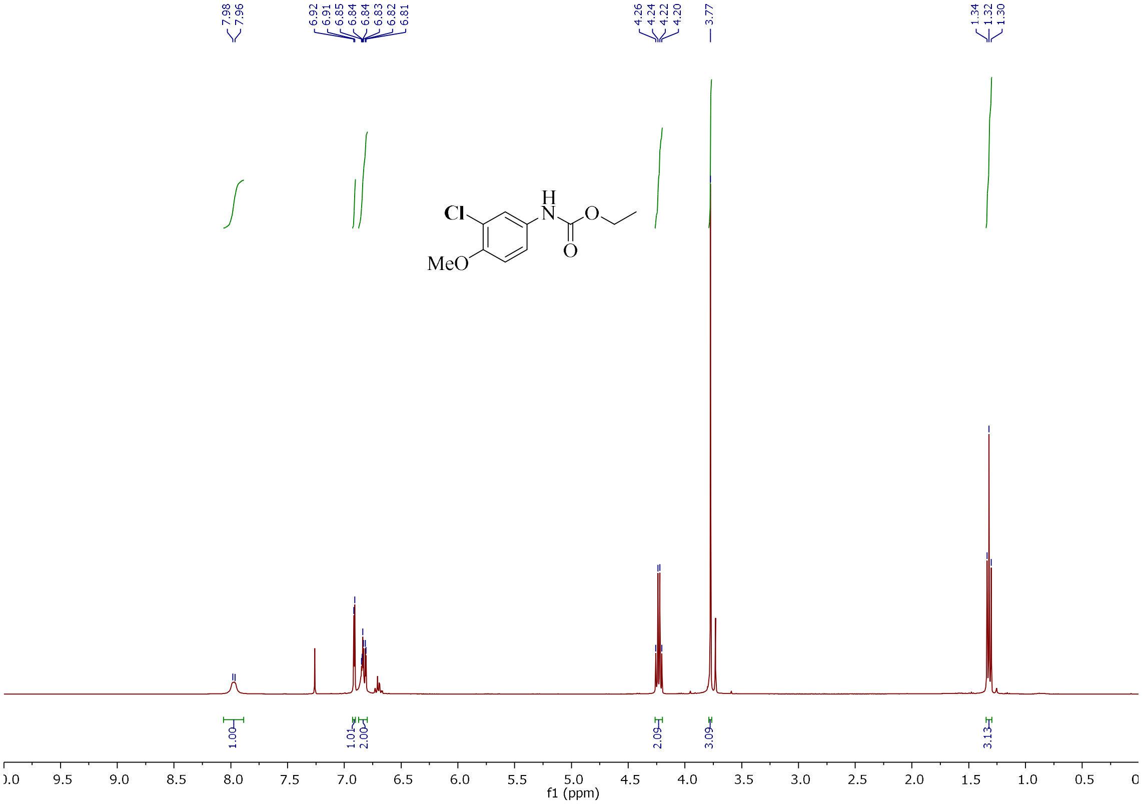

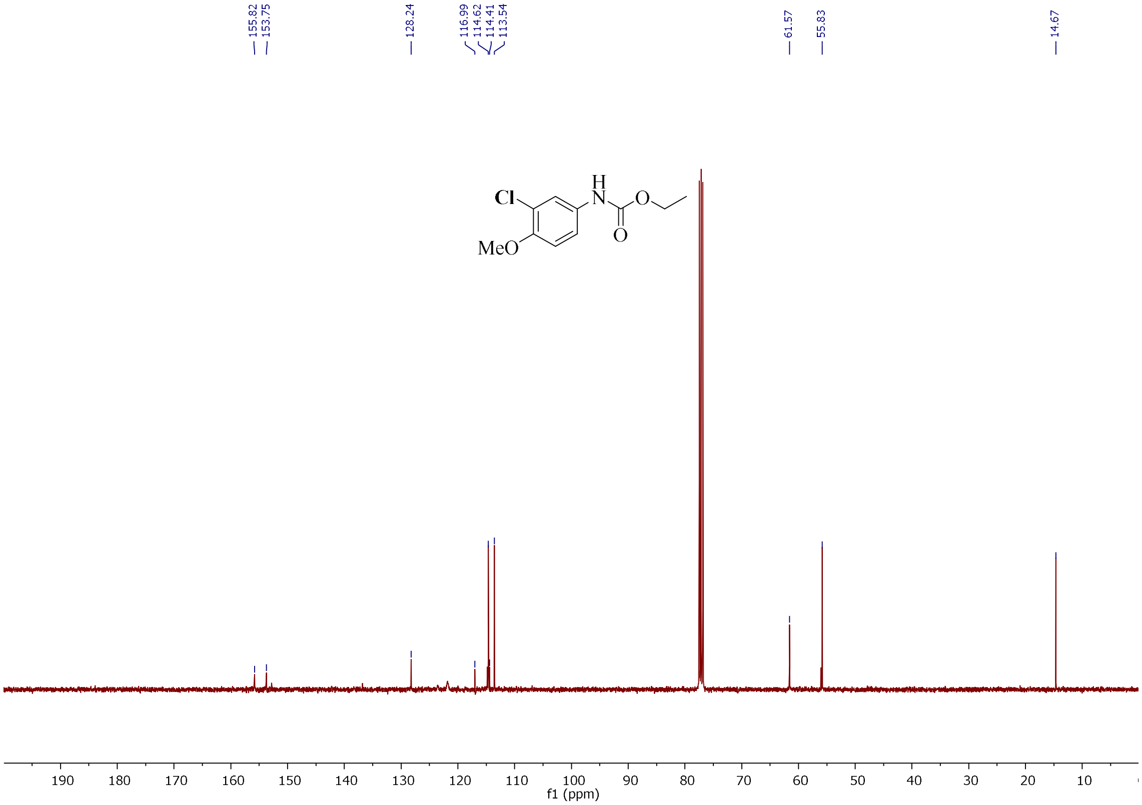

***N*-(3-chloro-4-methoxyphenyl)-2-phenylacetamide: (75)**

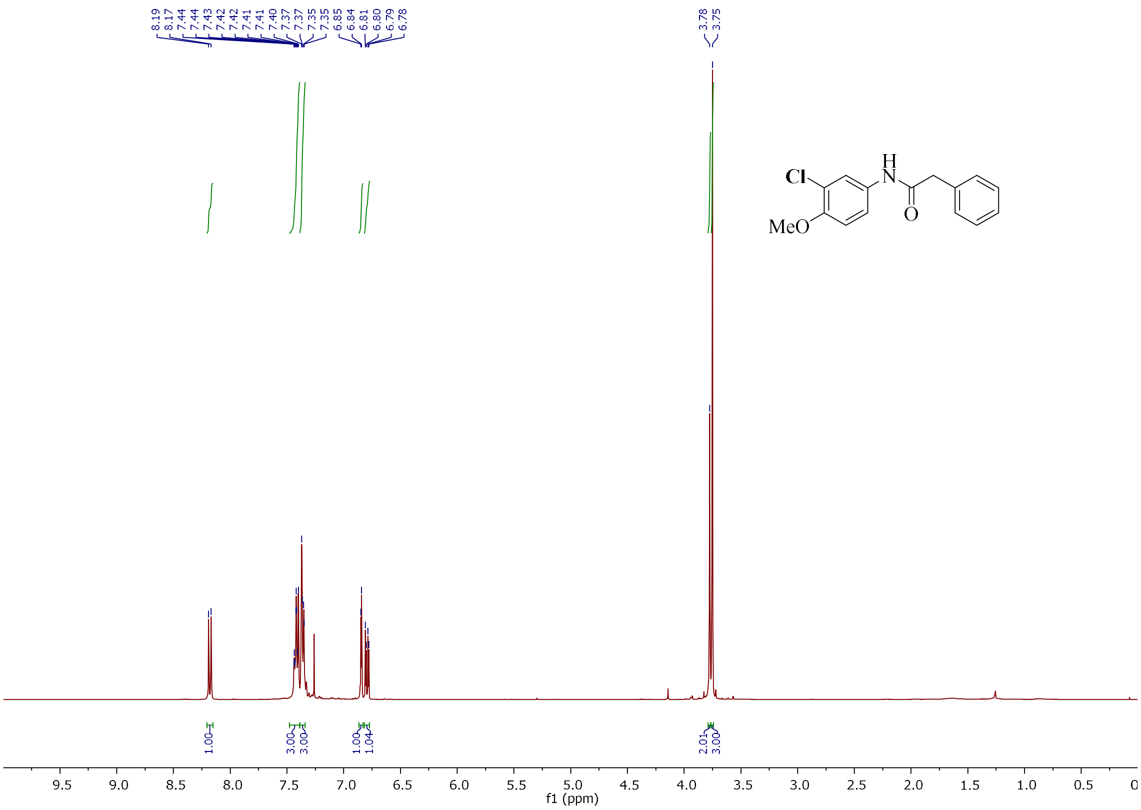

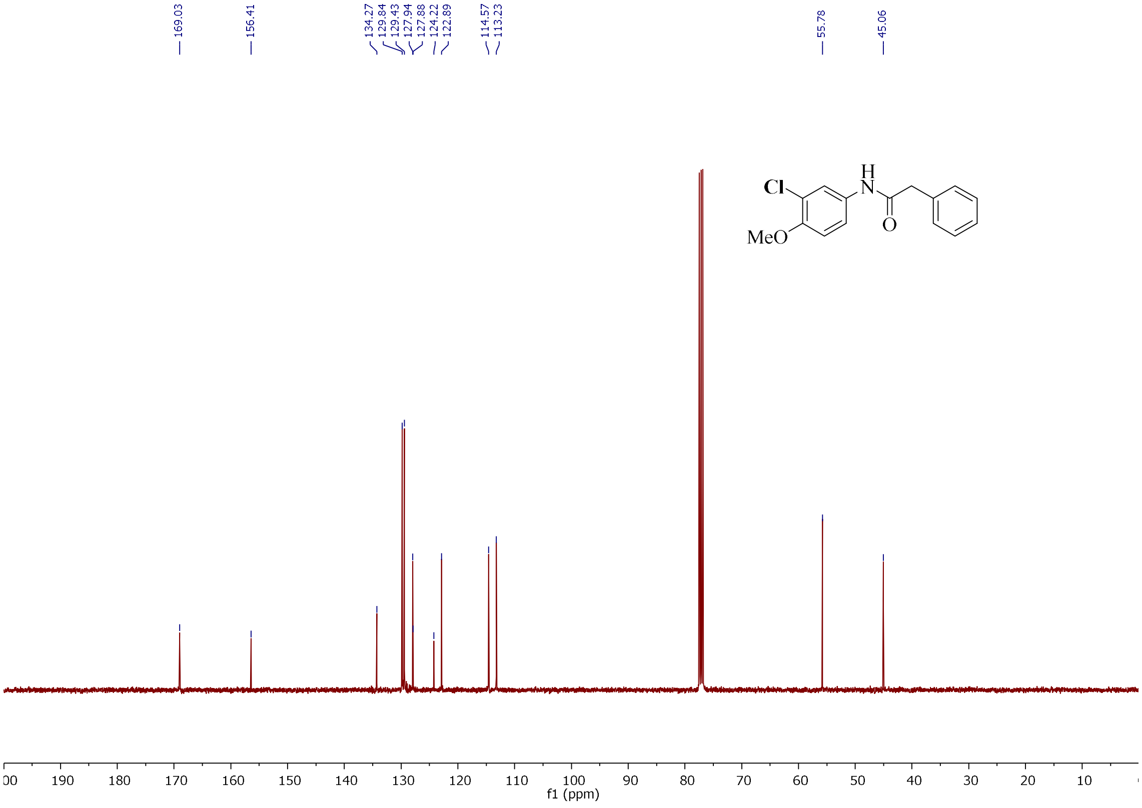

_N_-(3-chloro-4-methoxyphenyl)pivalamide: (76)

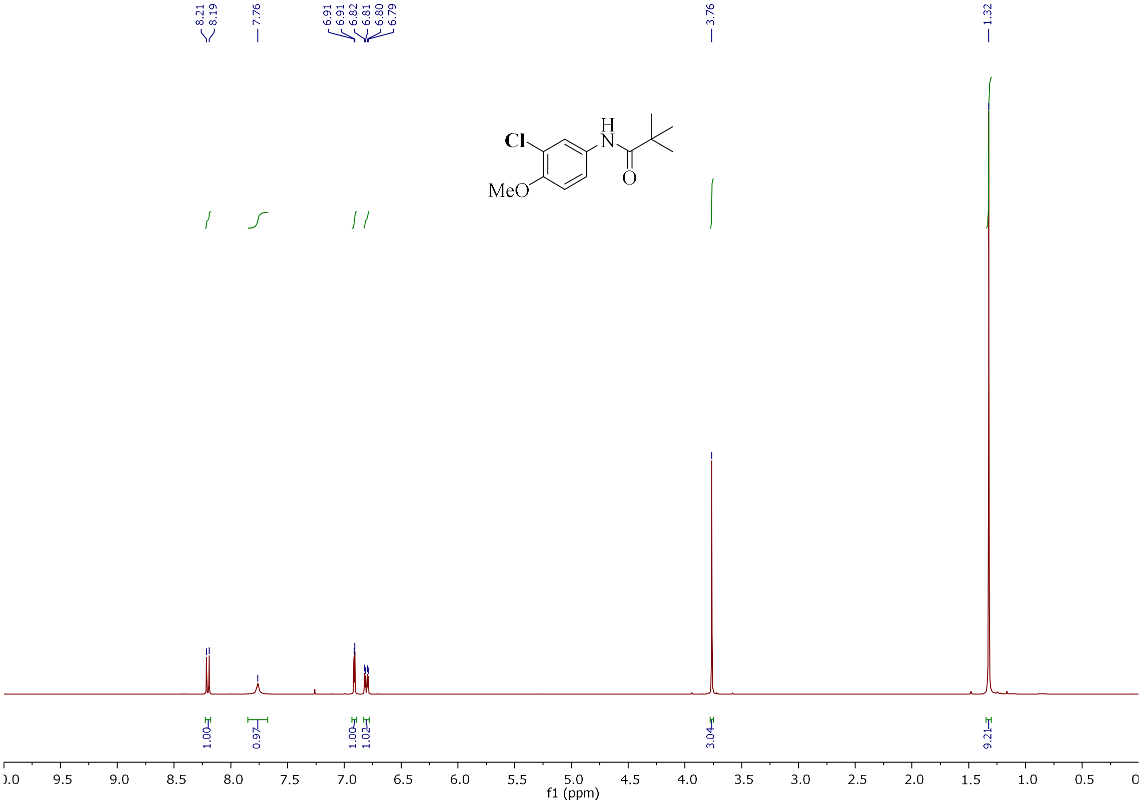

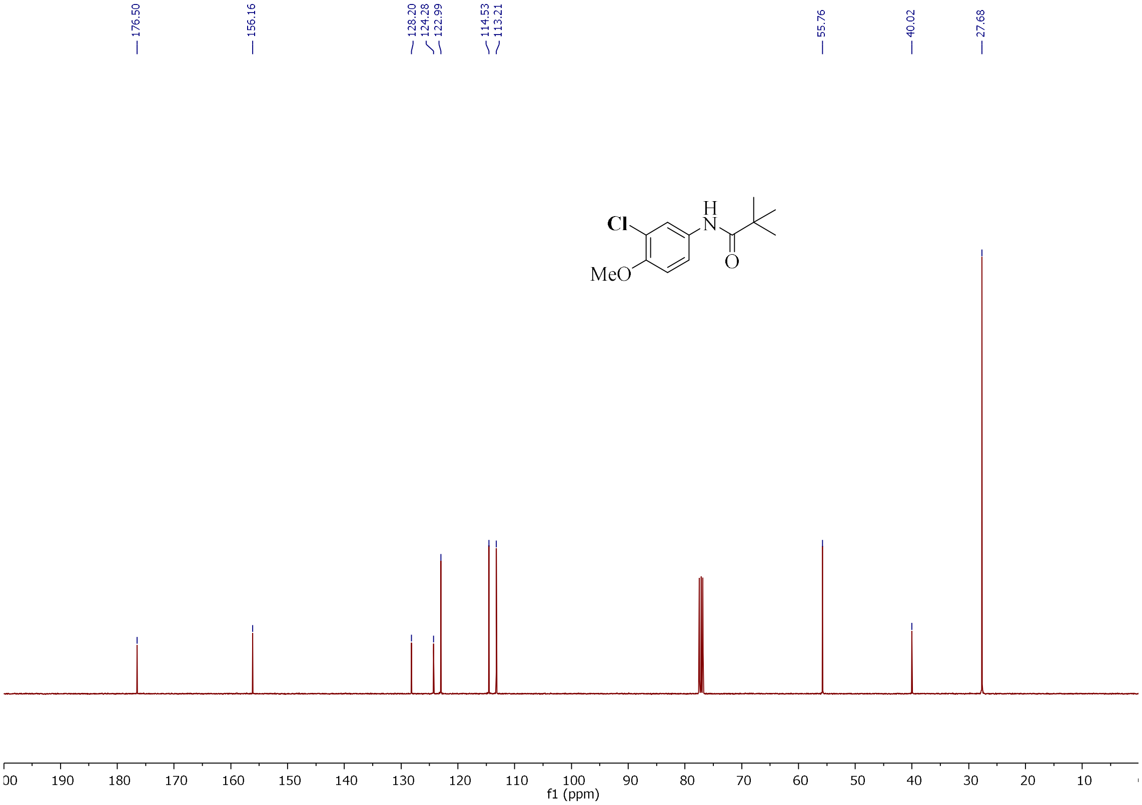

2-chloro-*N*-(3-chloro-4-methoxyphenyl)acetamide: (77)

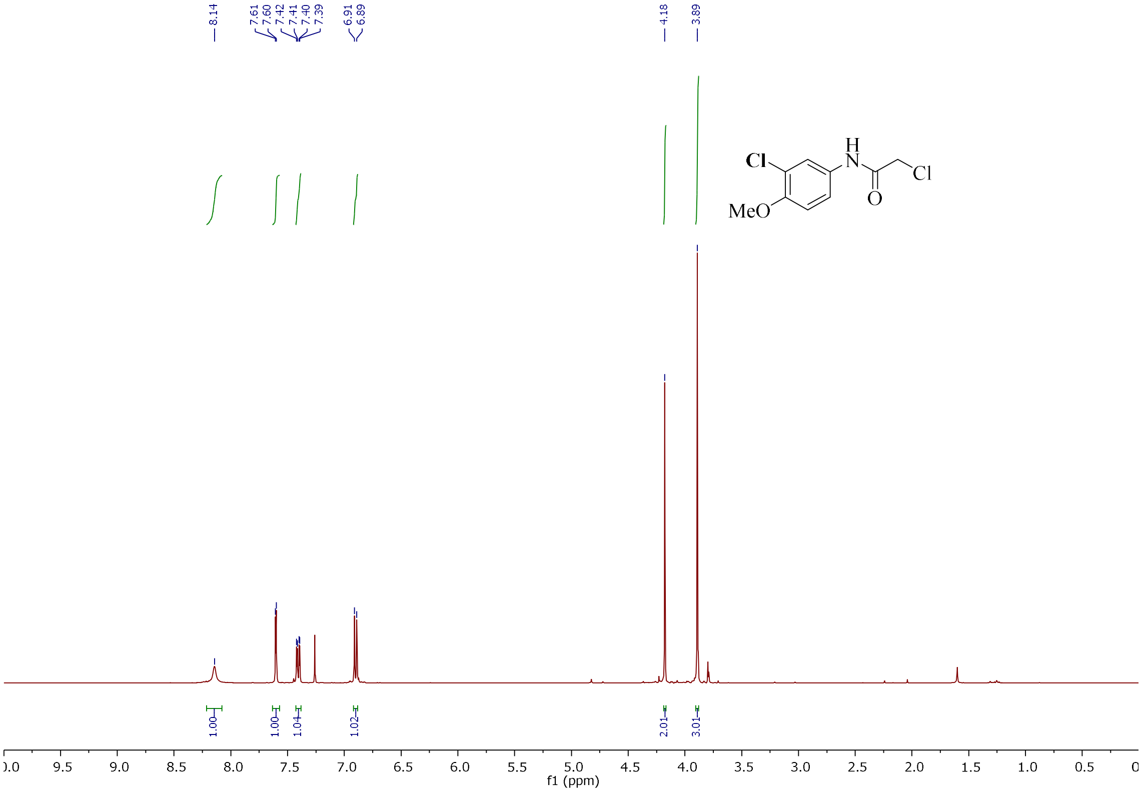

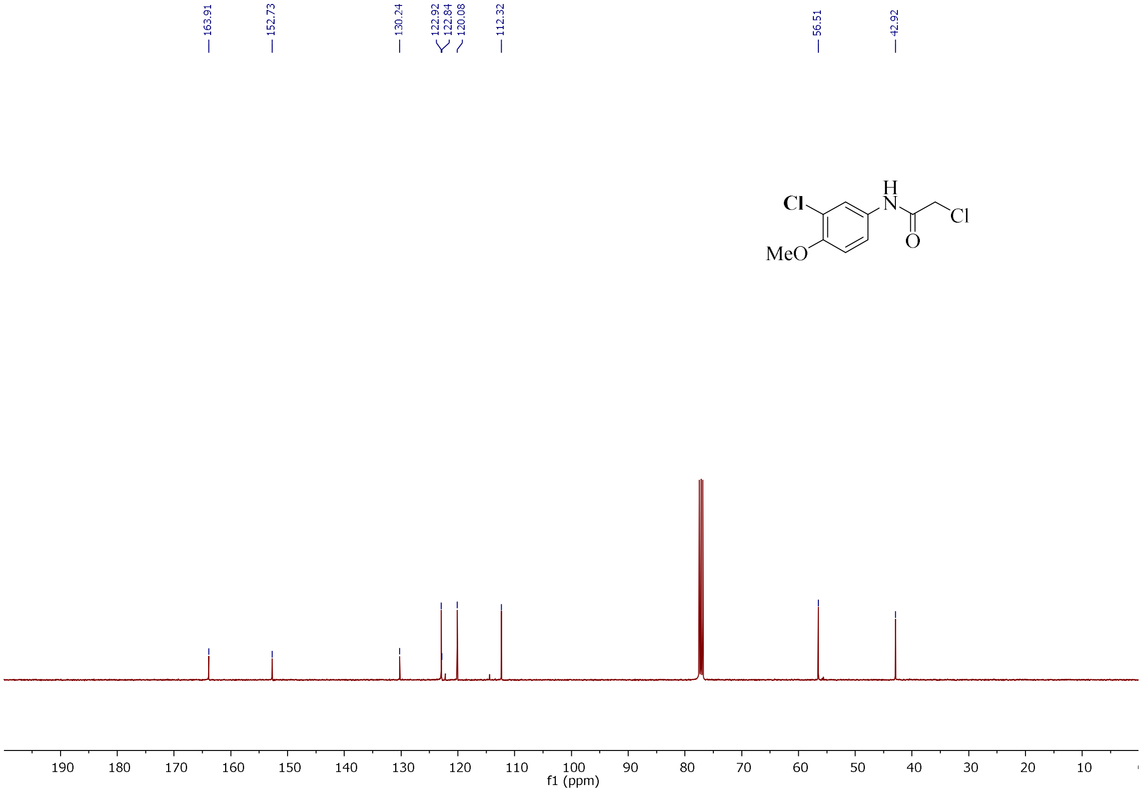

_N_-(4-bromophenyl)acetamide: (78)

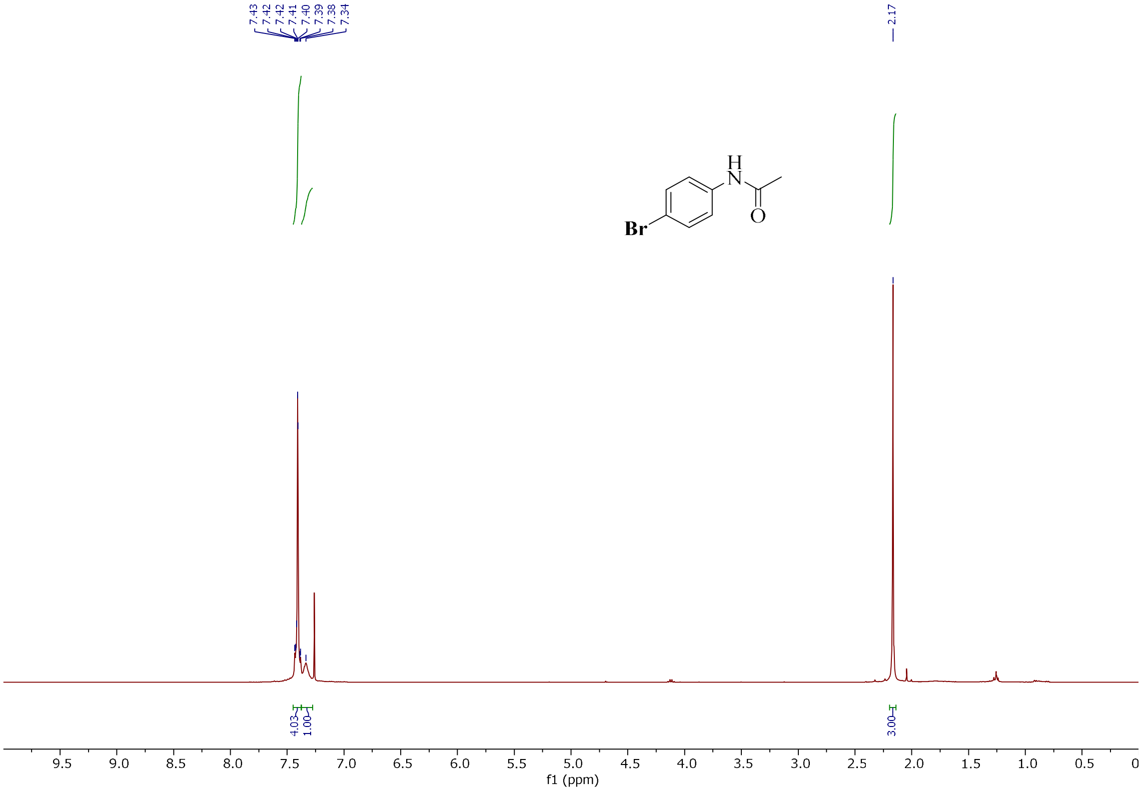

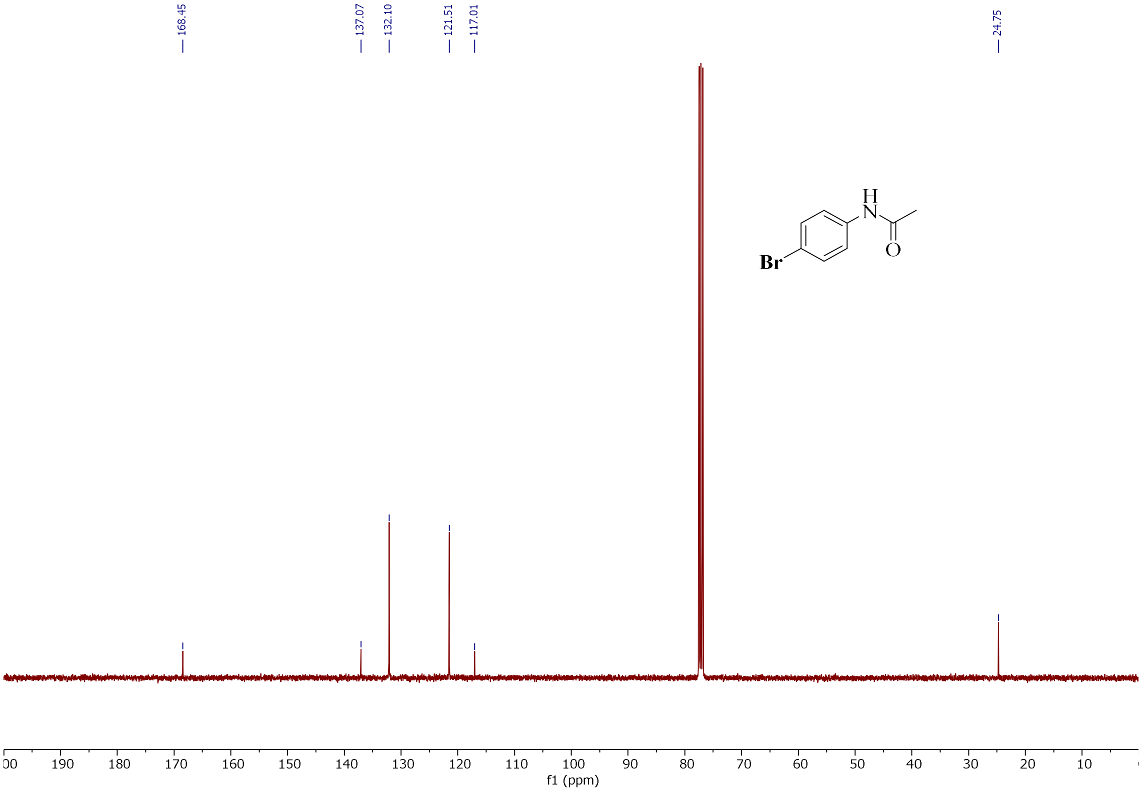

***N*-(4-bromophenyl)propionamide: (79)**

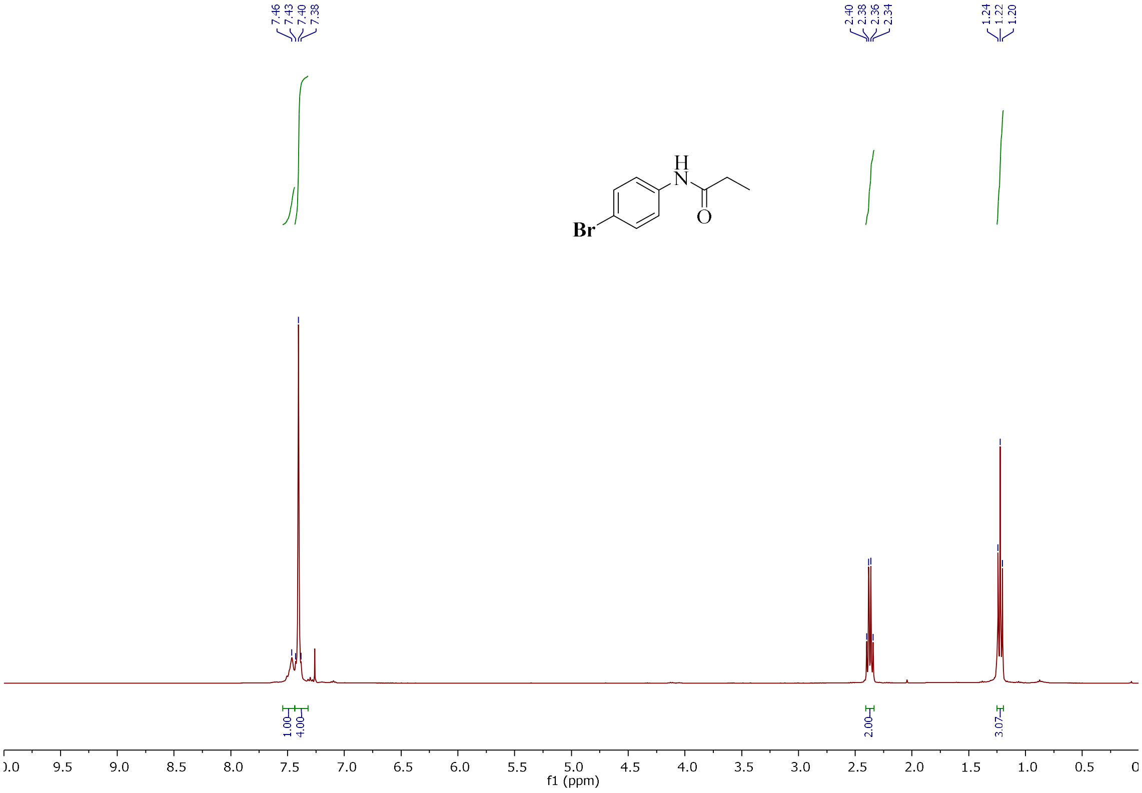

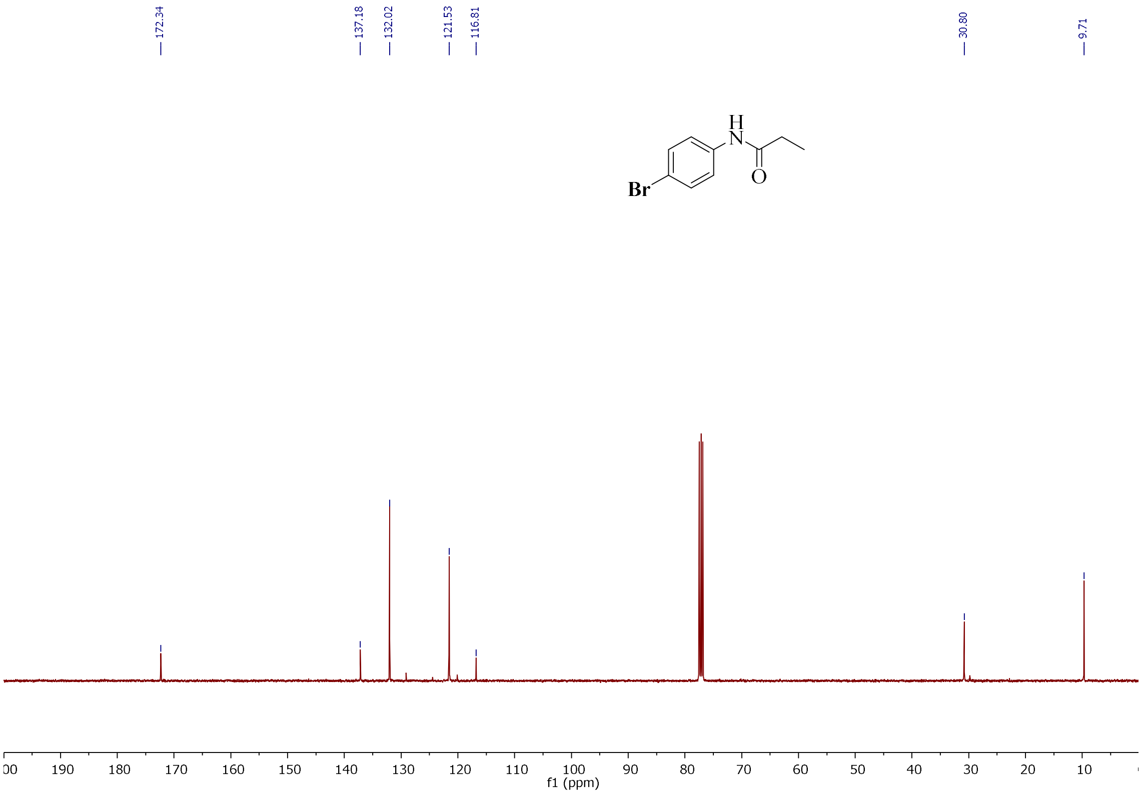

***N*-(4-bromo-2-fluorophenyl)propionamide: (80)**

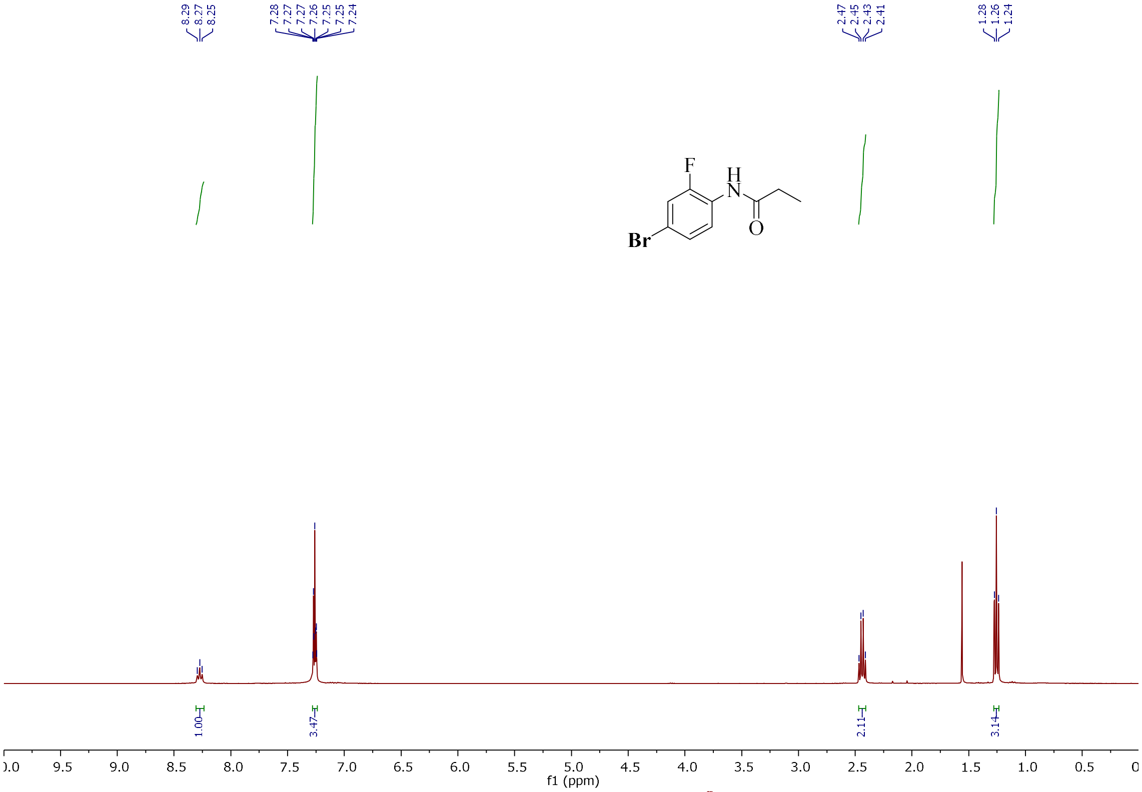

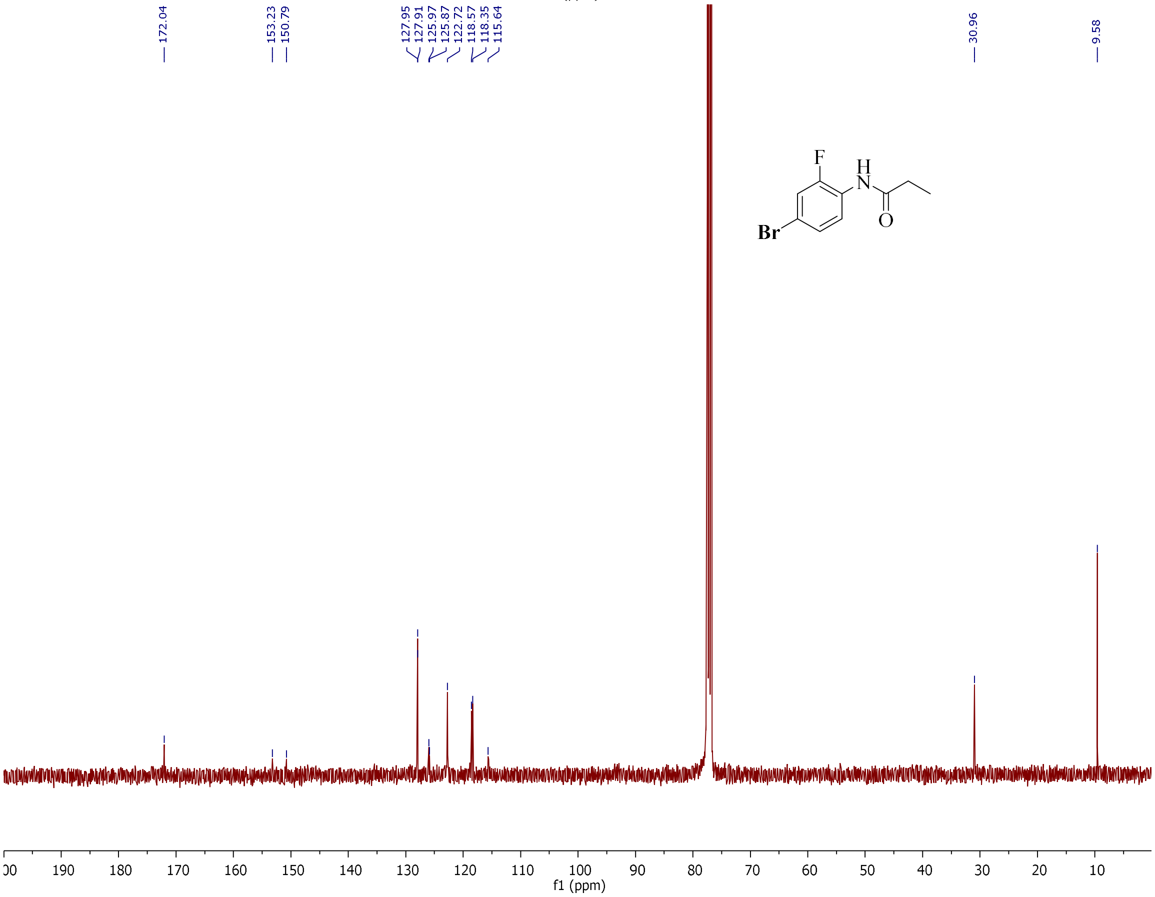

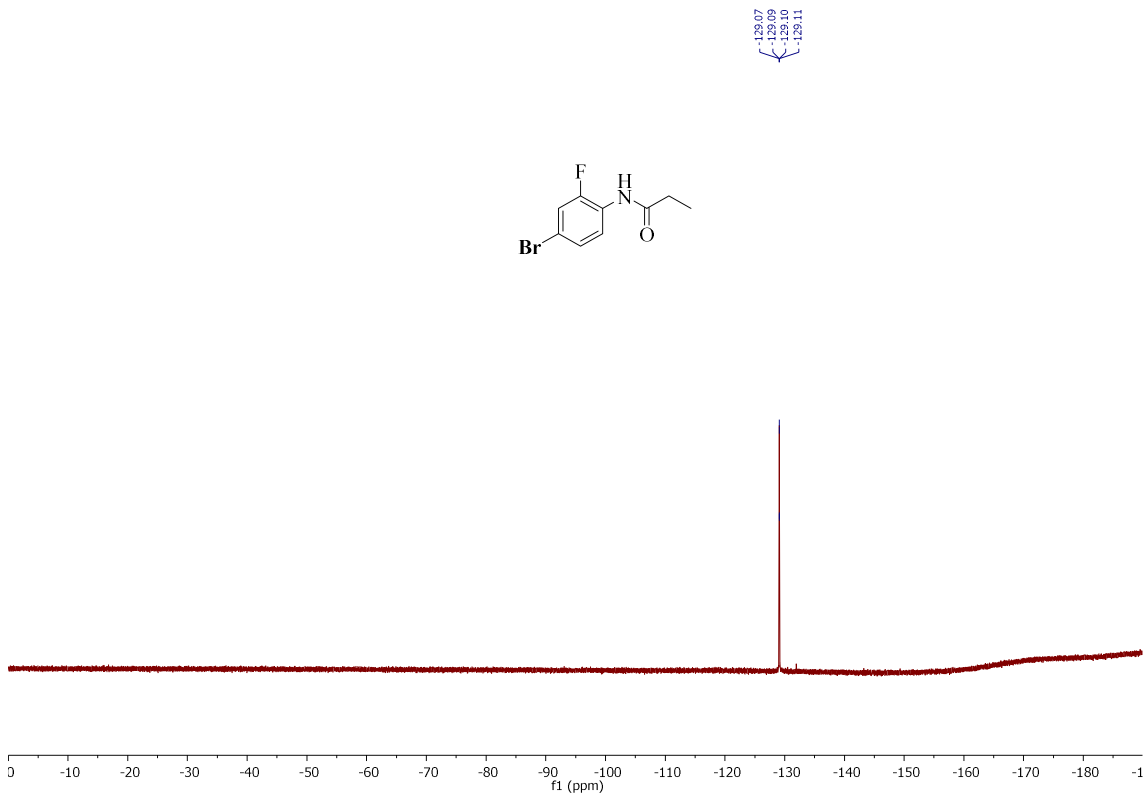

-129.07
-129.09
-129.10
-129.11
F
H
N
O
Br
f1 (ppm)

***N*-(3,4-dibromophenyl)propionamide: (81)**

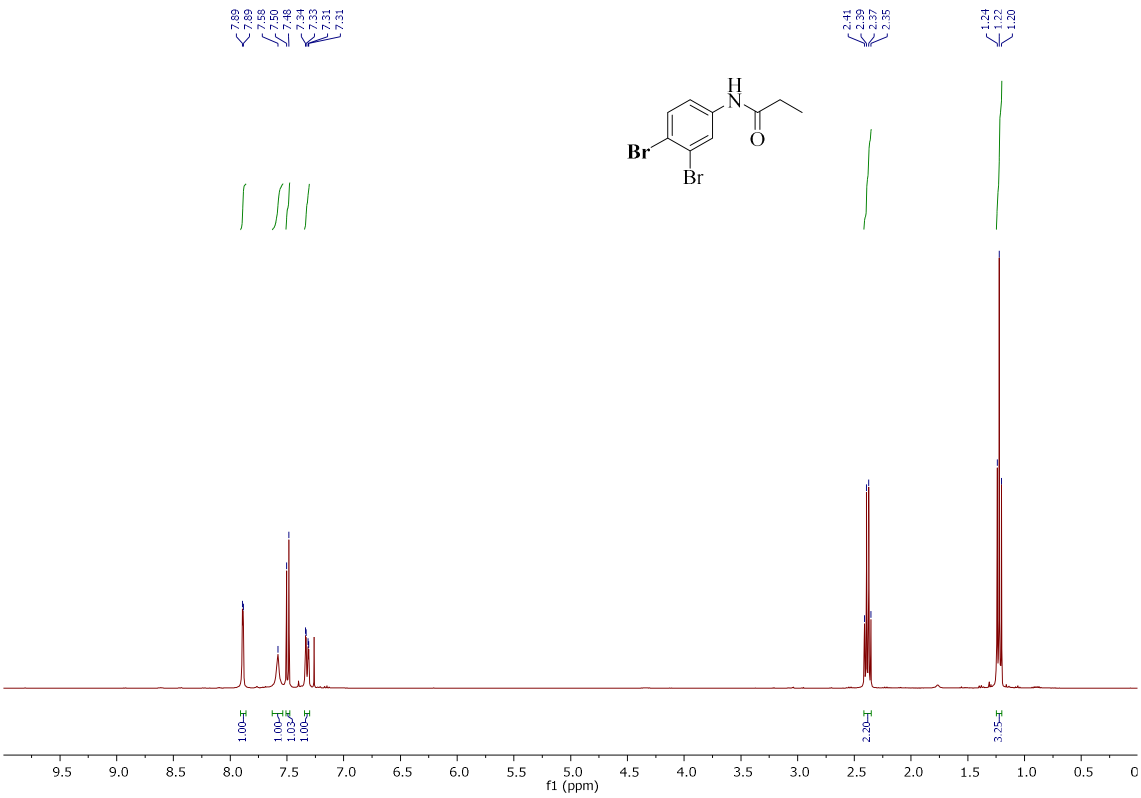

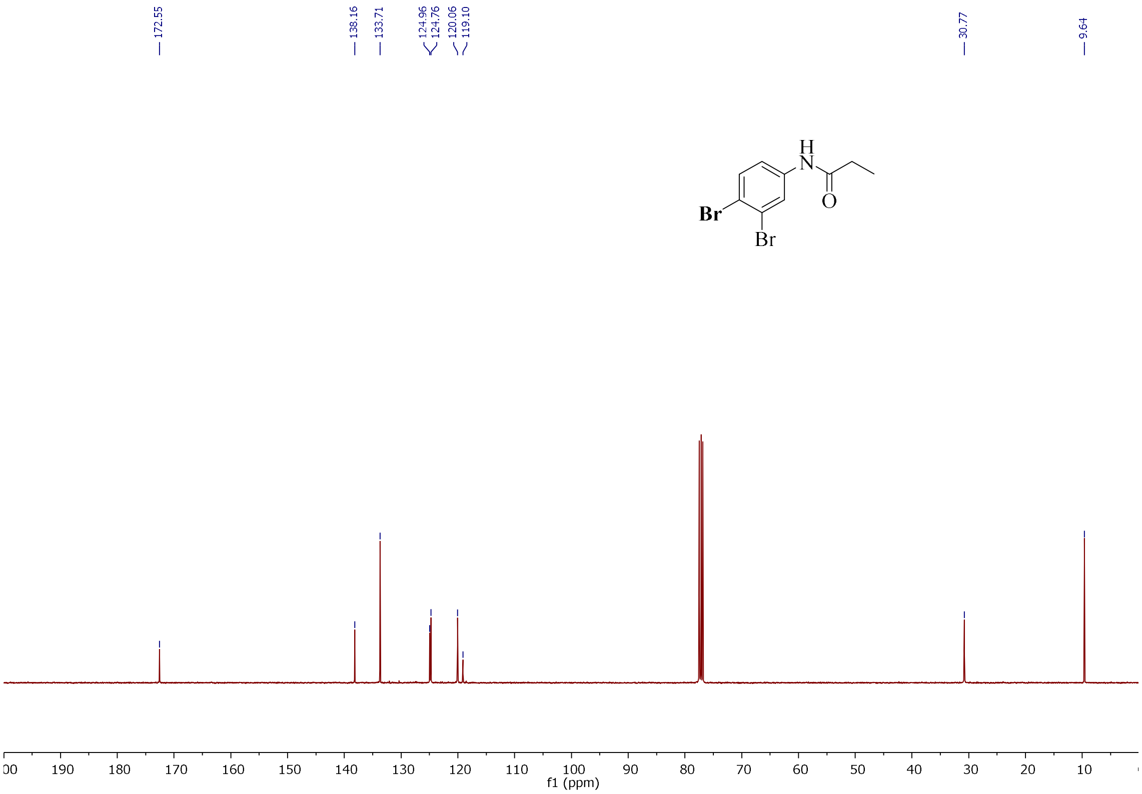

***N*-(4-bromophenyl)benzamide: (82)**

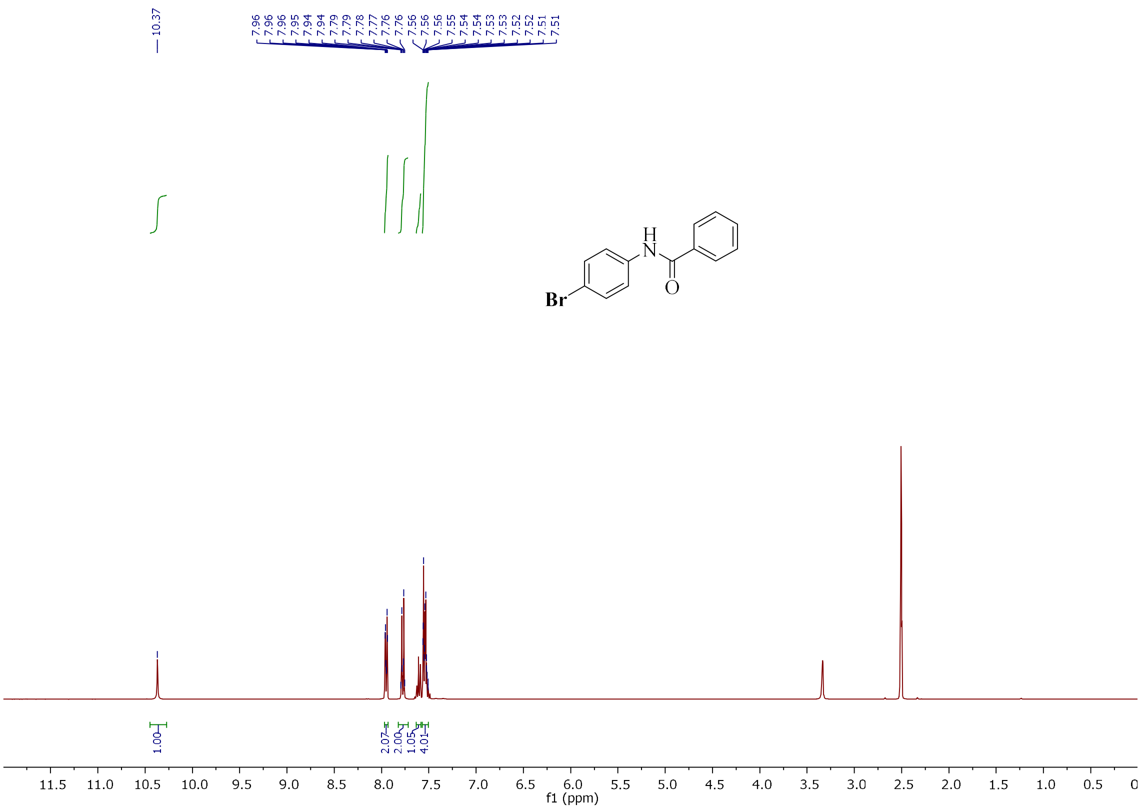

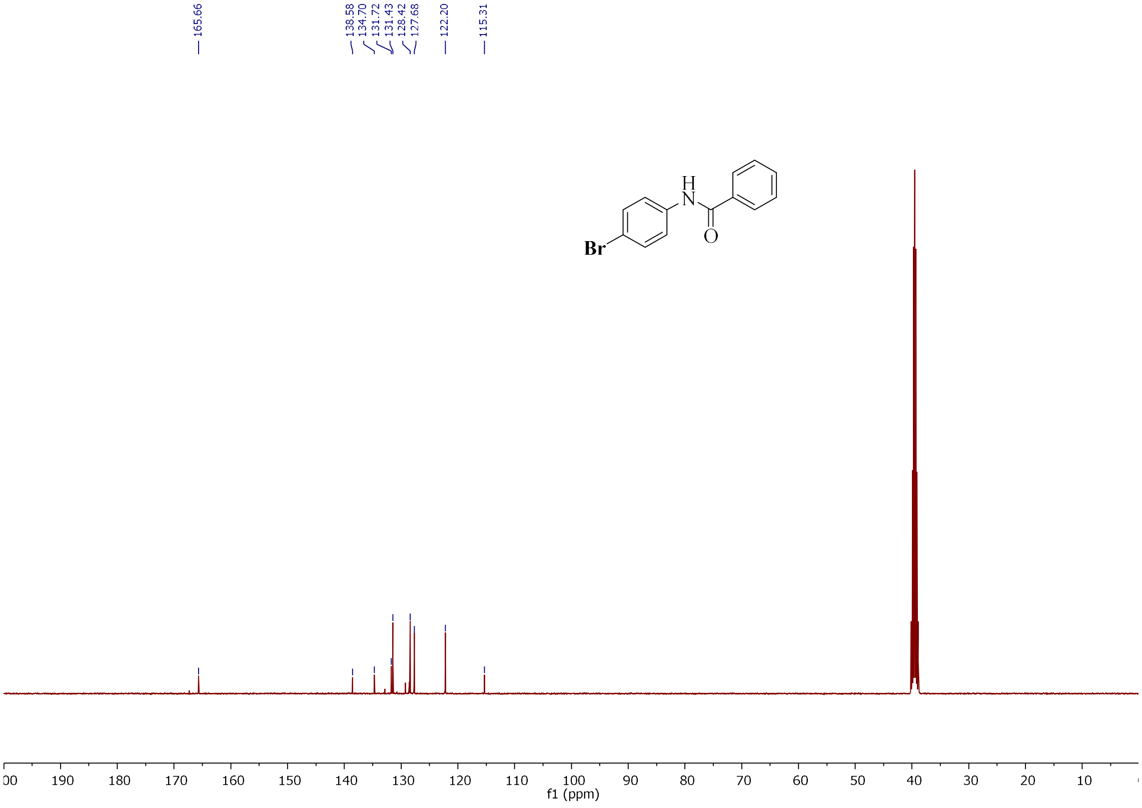

***N*-(4-bromo-3-methylphenyl)benzamide: (83)**

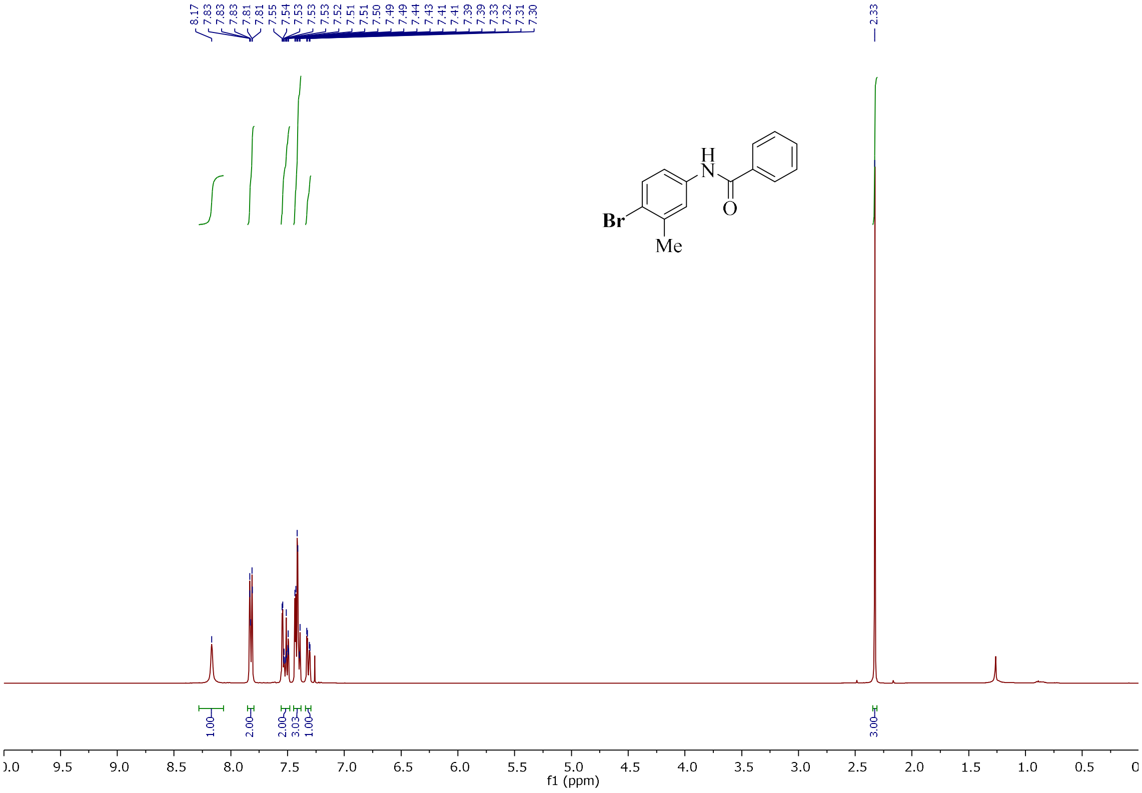

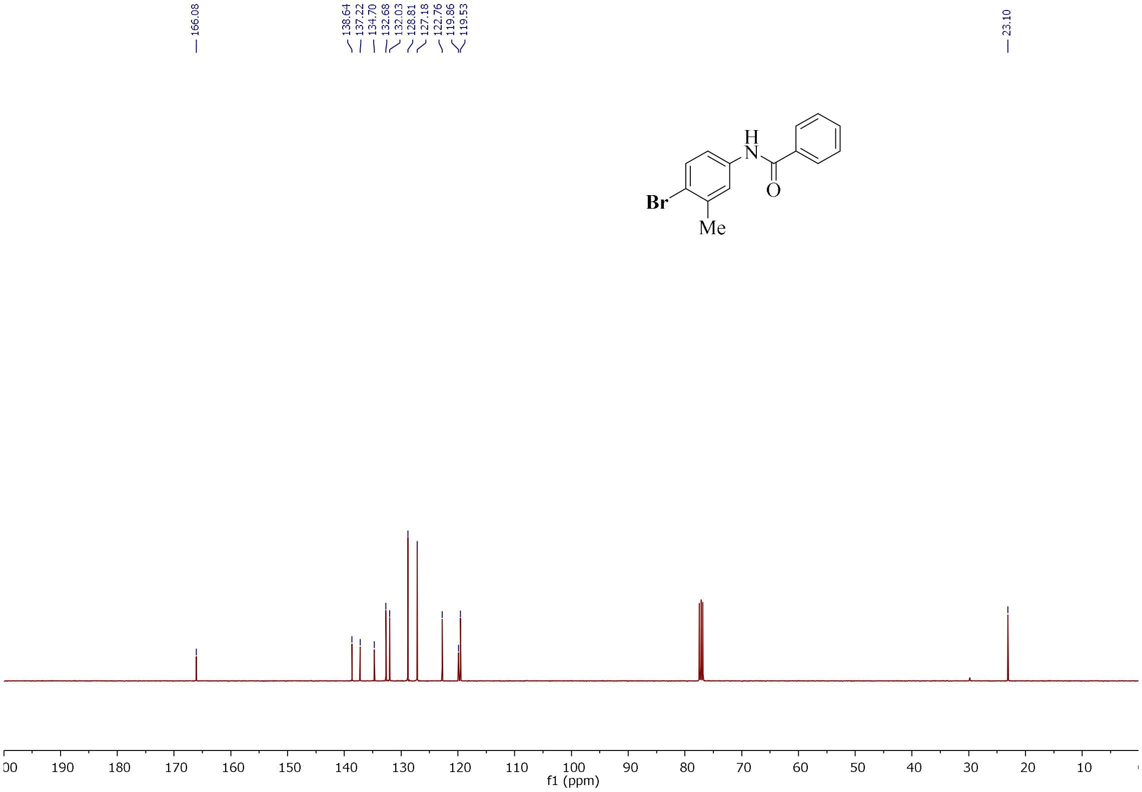

_N_-(4-bromo-3,5-dimethylphenyl)benzamide: (84)

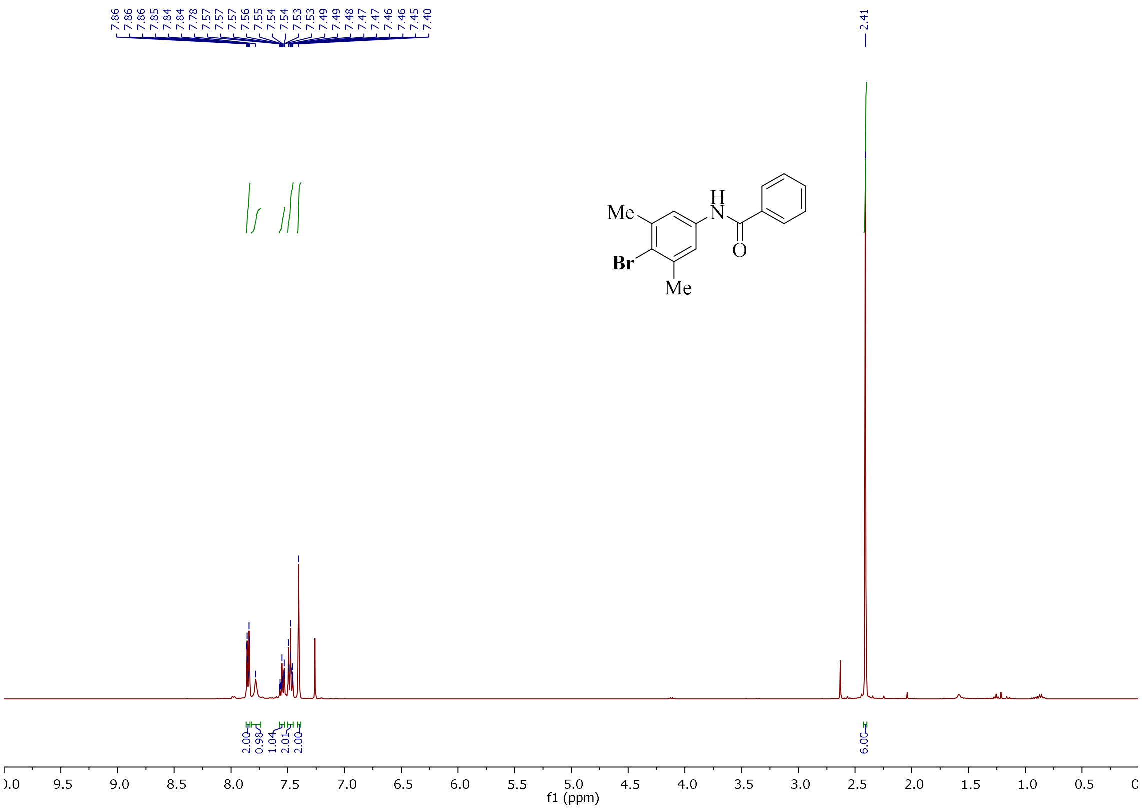

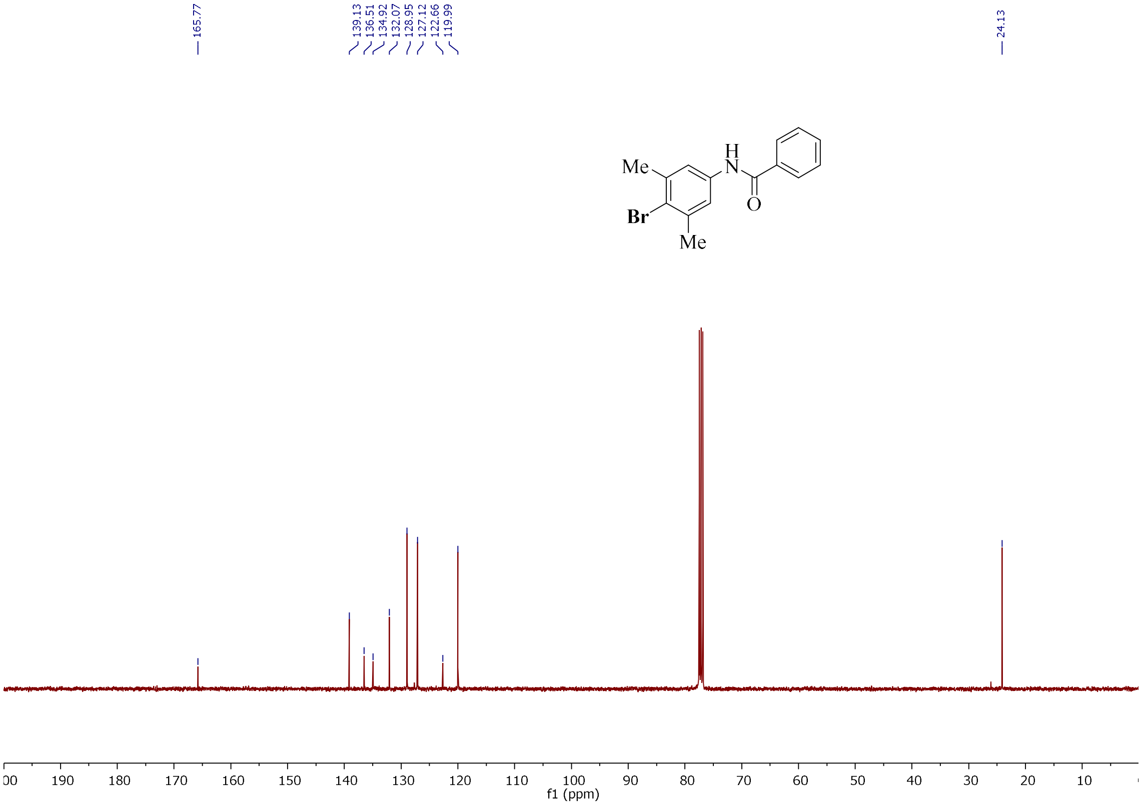

N-(4-bromo-2-methoxy-5-methylphenyl)benzamide: (85)

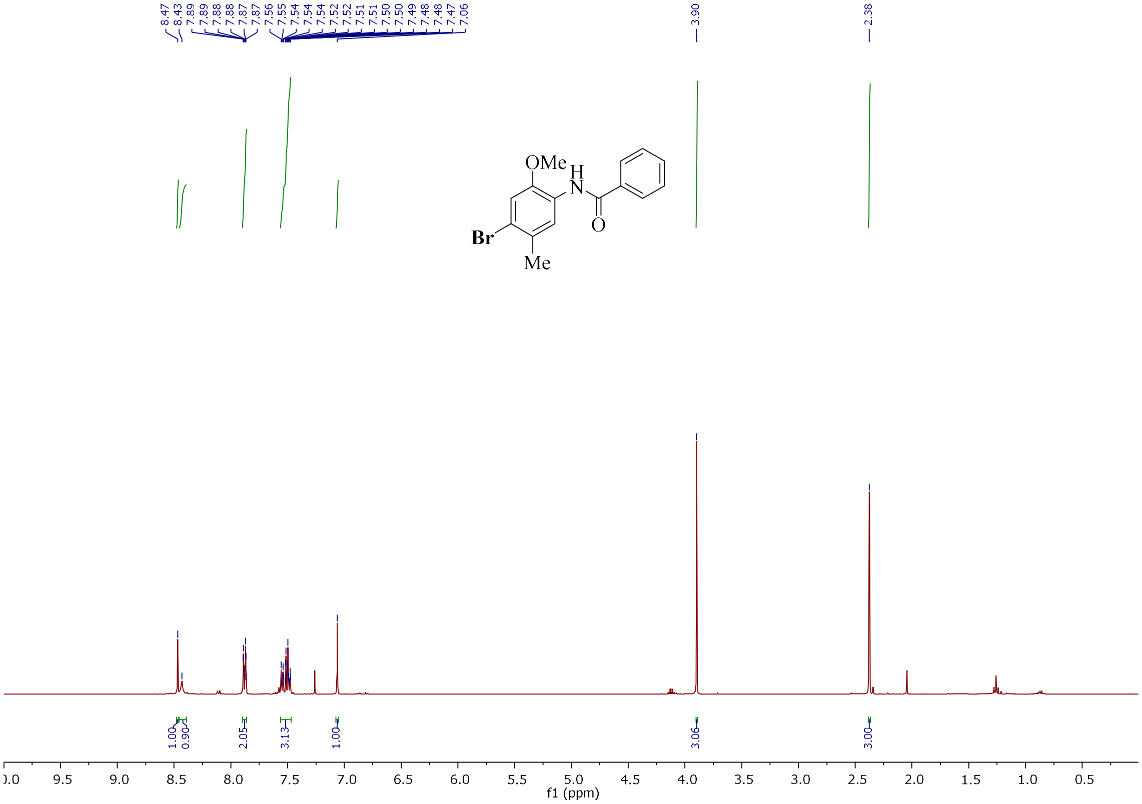

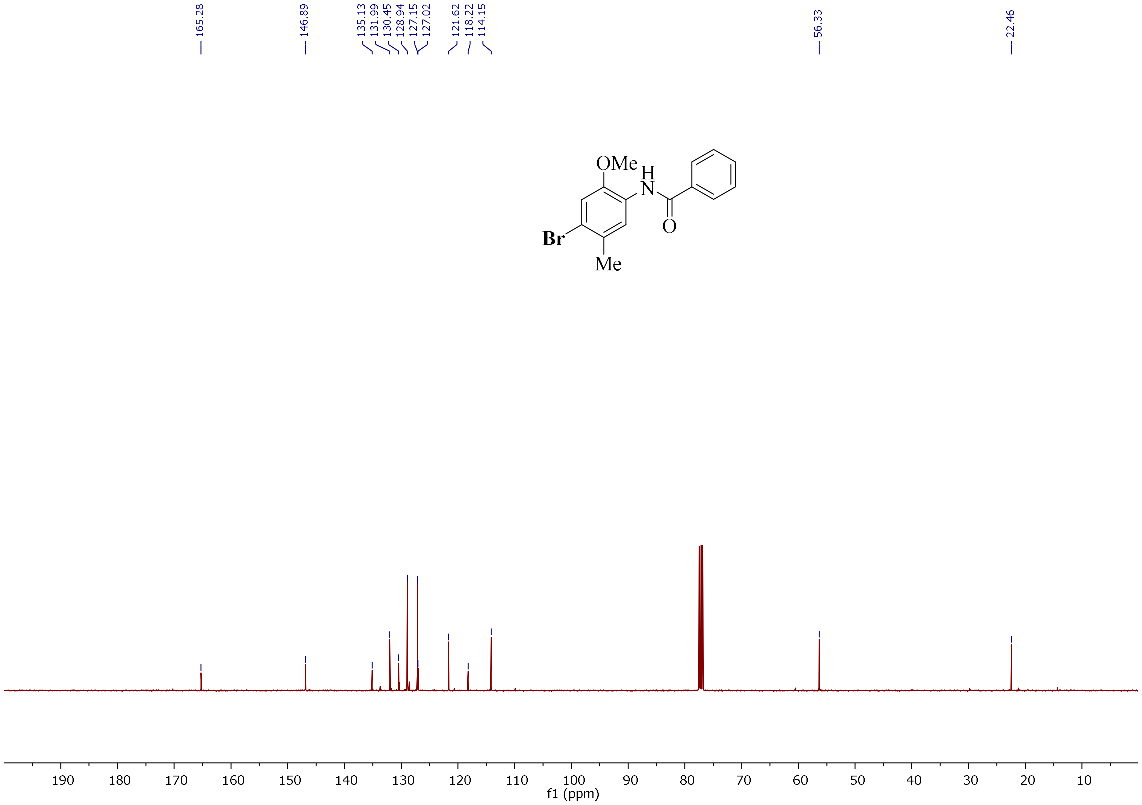

2-Bromo-*N*-(4-bromo-3-methylphenyl)propanamide: (86)

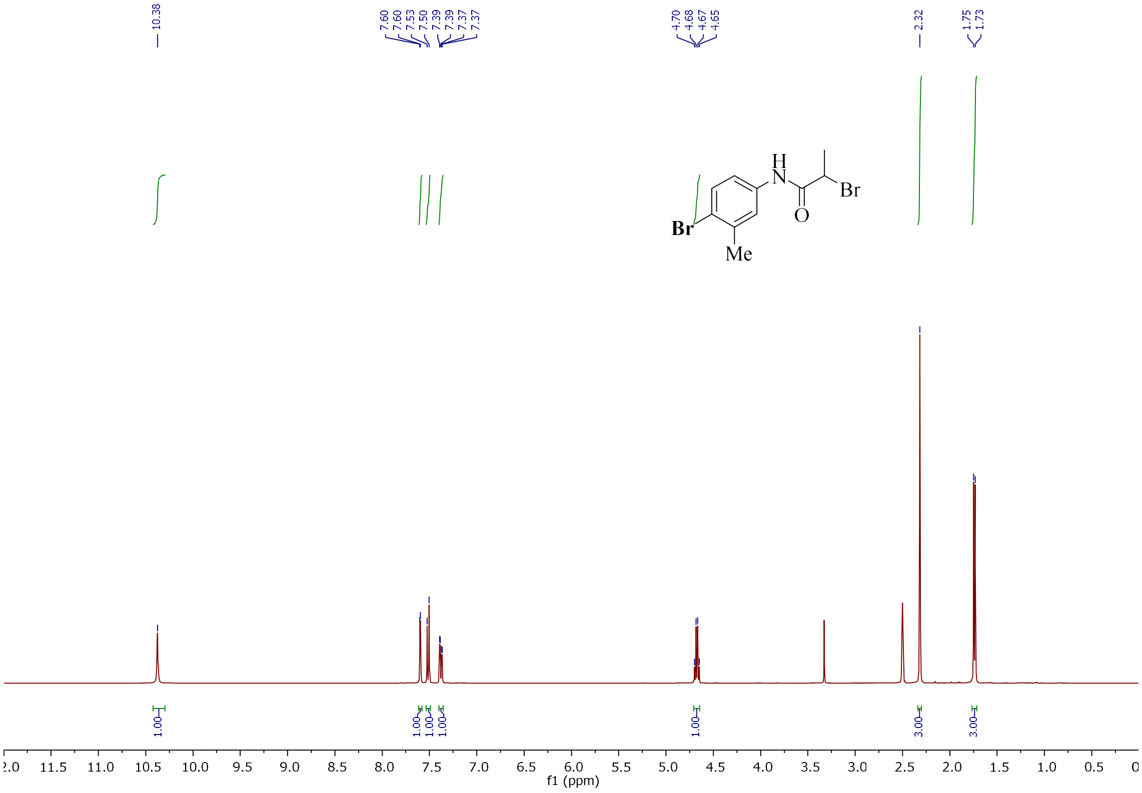

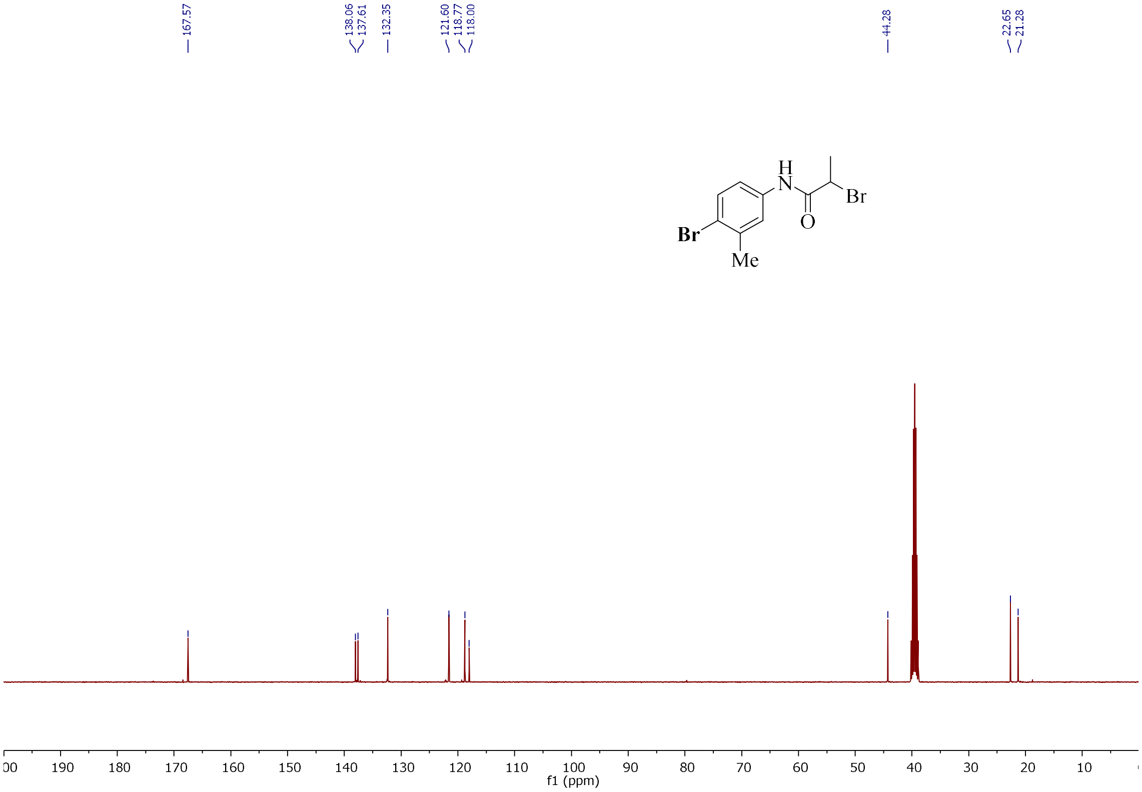

2-Bromo-*N*-(5-bromo-2-methoxyphenyl)propanamide: (87)

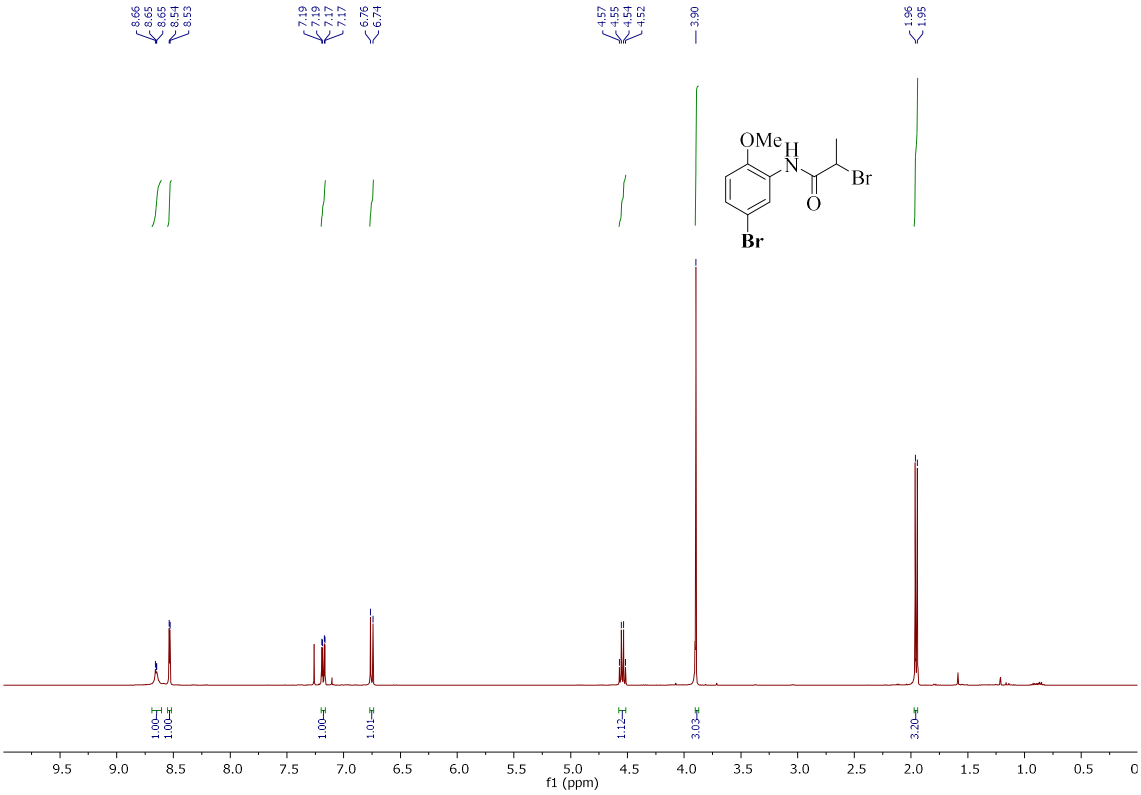

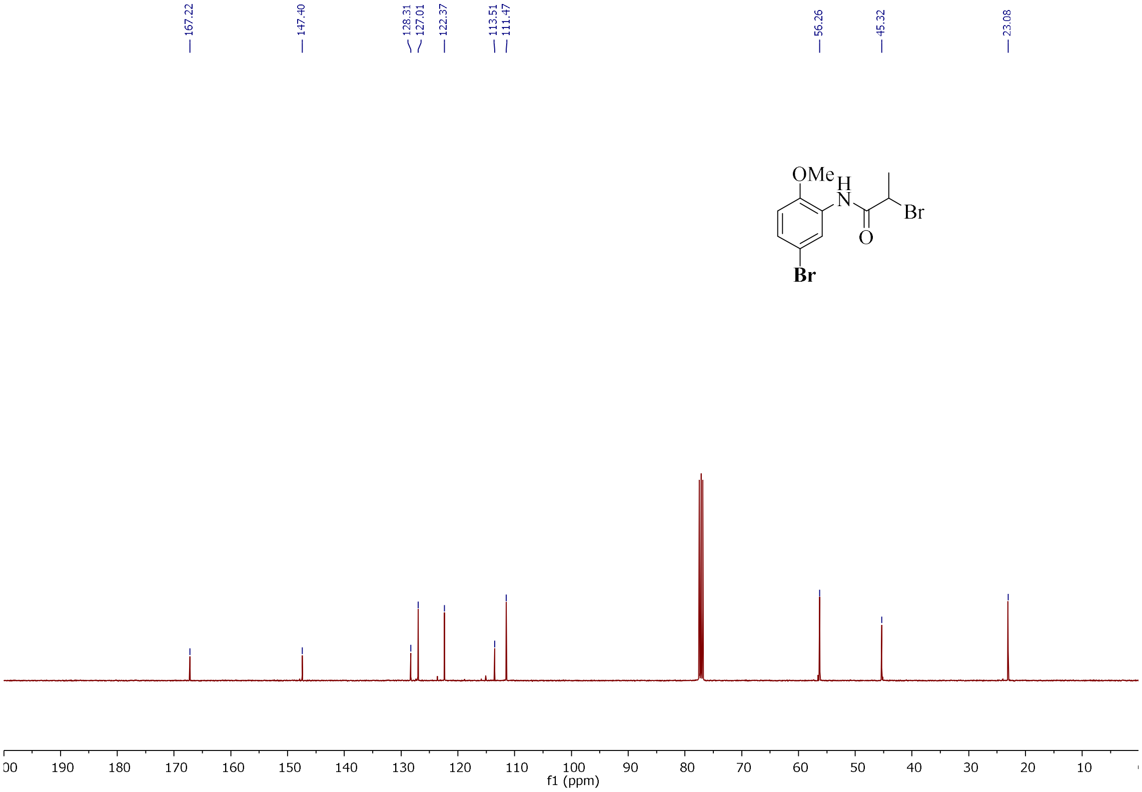

2-Bromo-N-(3-bromo-2,6-dimethoxyphenyl)propanamide: (88)

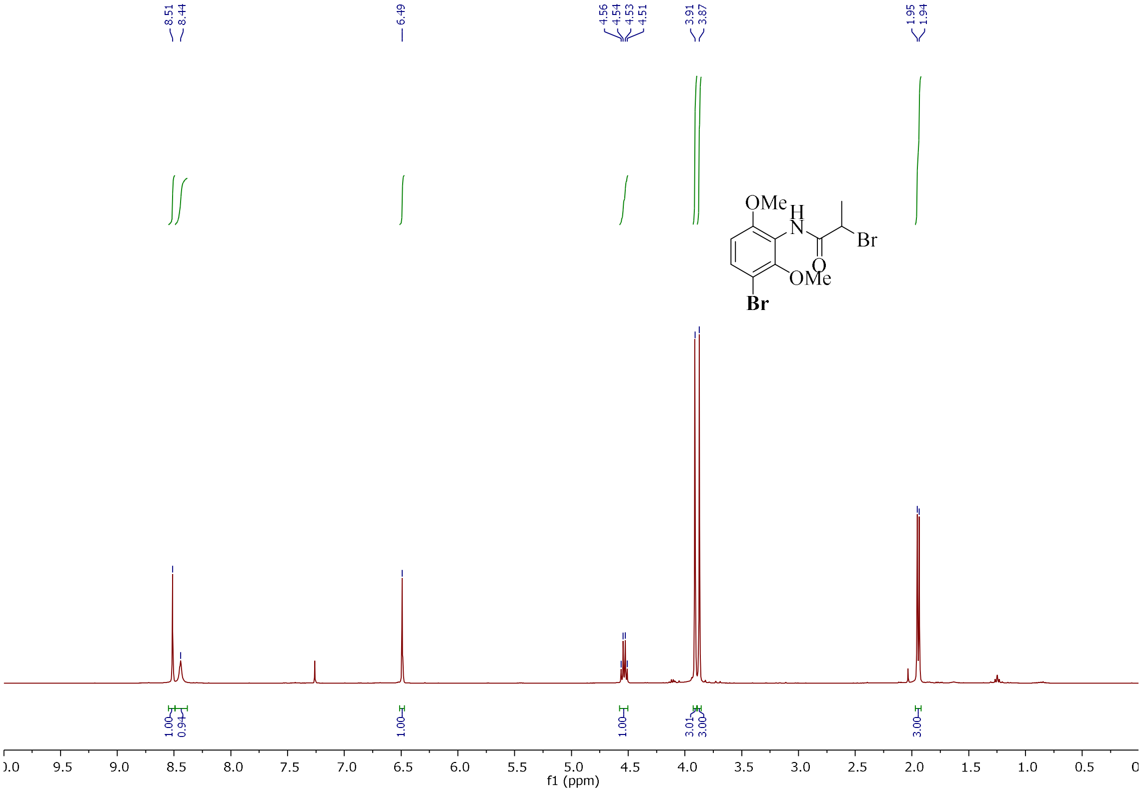

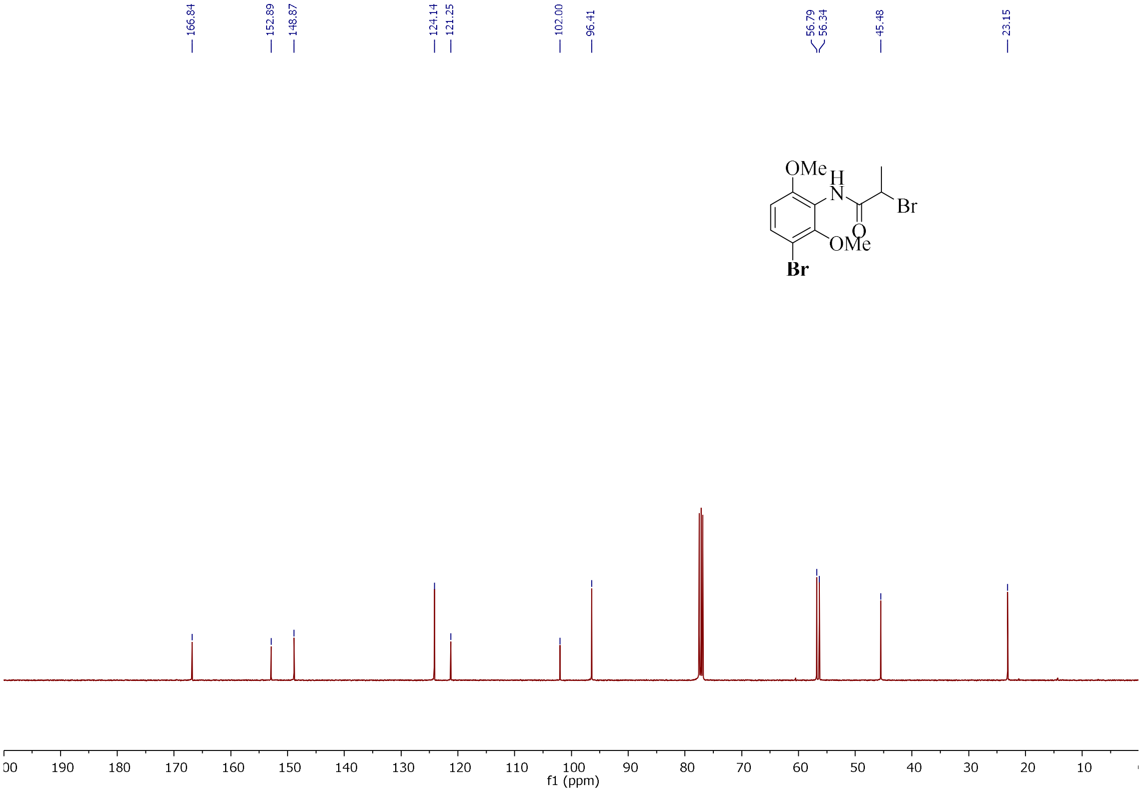

***N*-(1-bromonaphthalen-2-yl)acetamide: (89)**

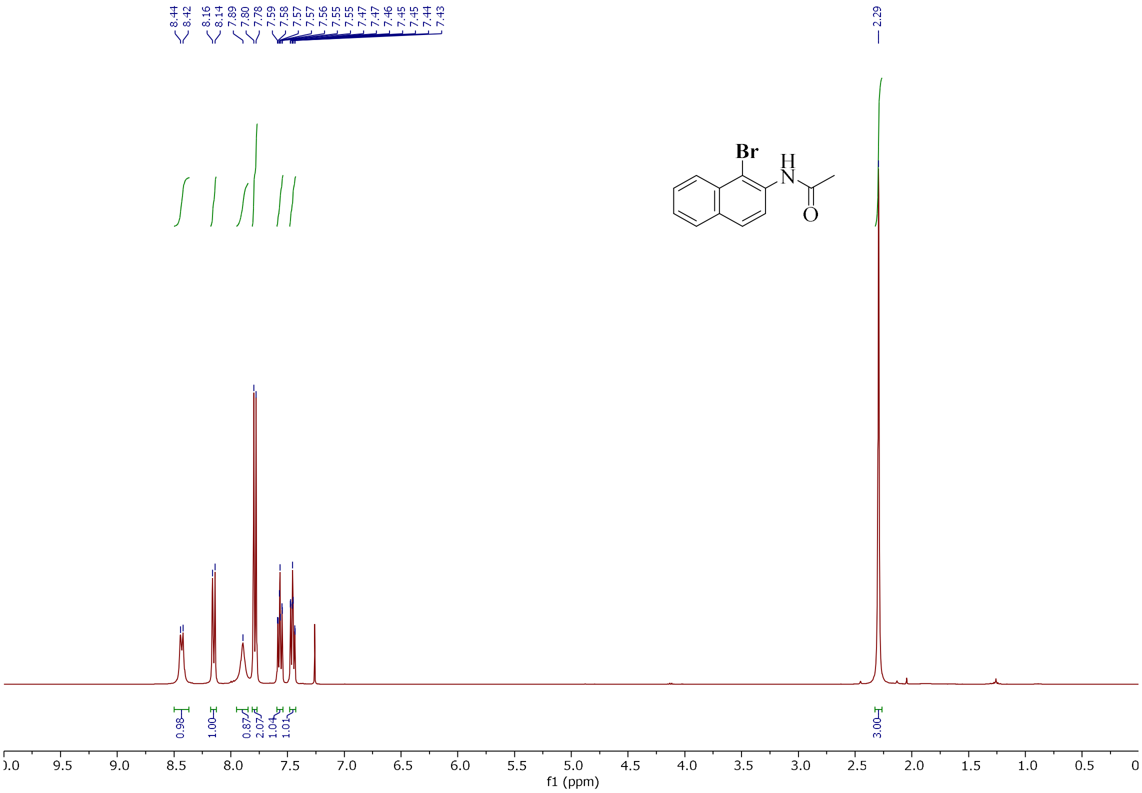

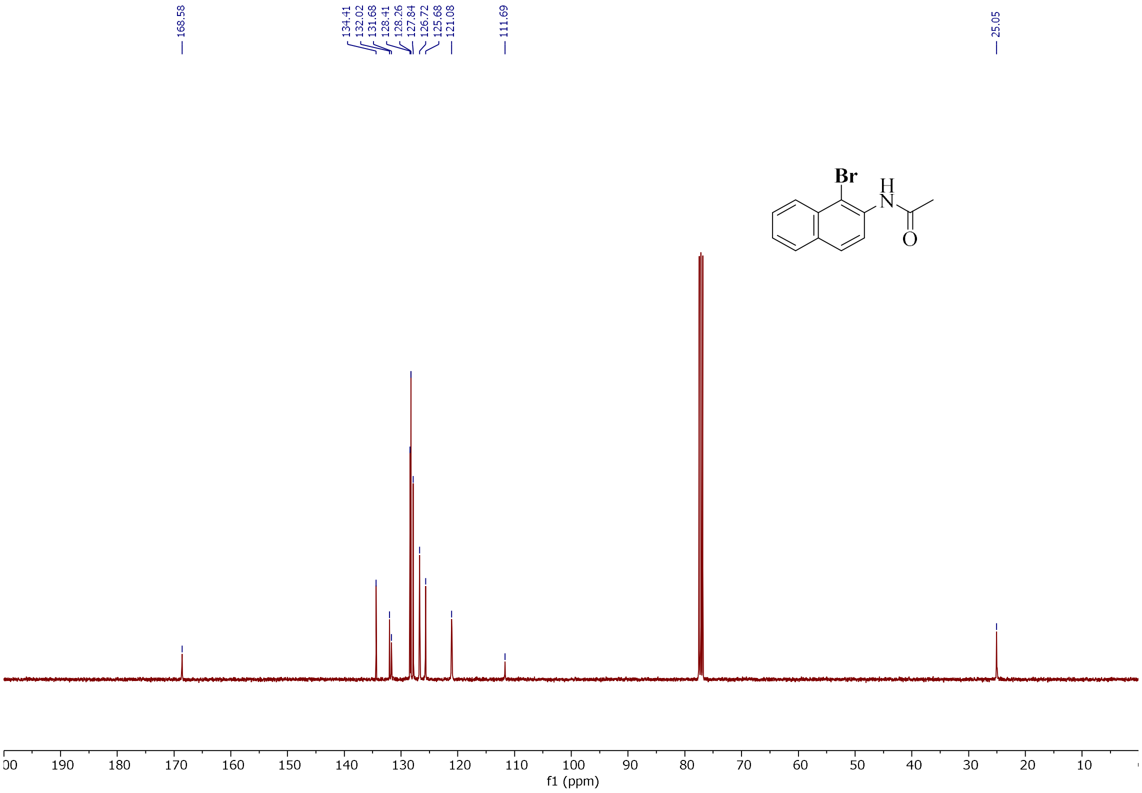

N-(1-bromonaphthalen-2-yl)propionamide: (90)

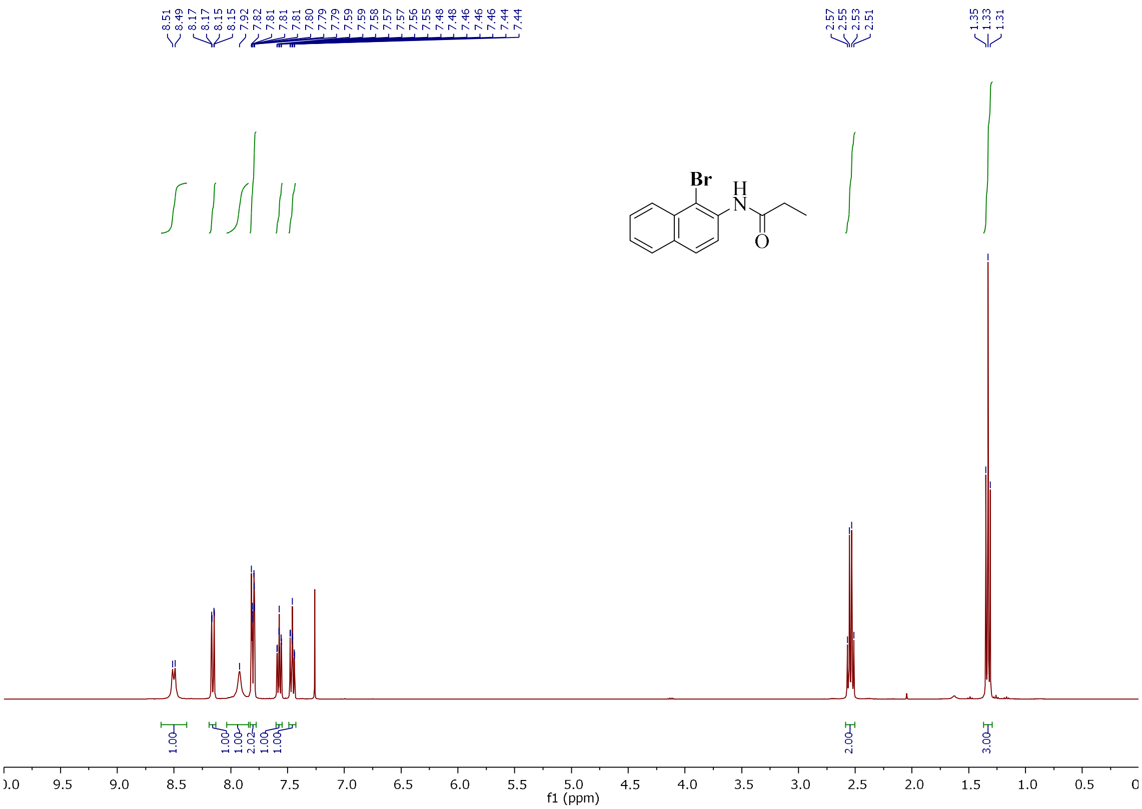

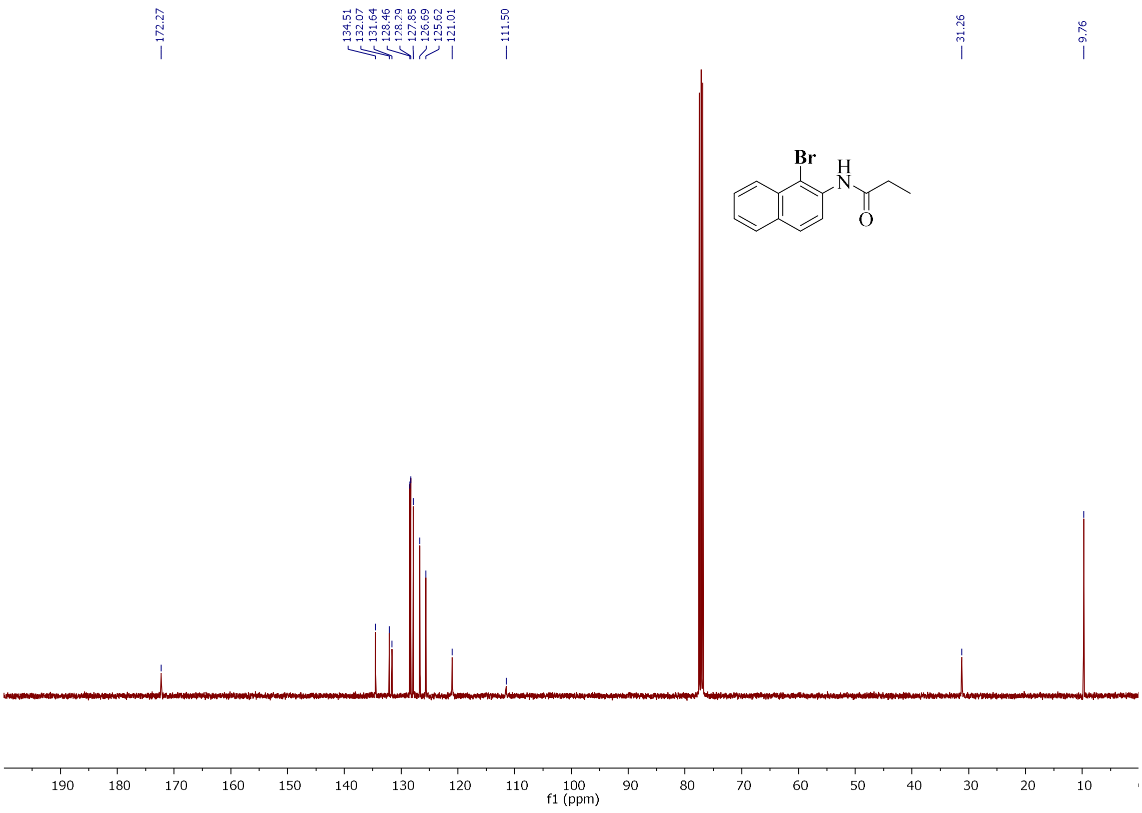

N-(1-bromonaphthalen-2-yl)benzamide: (91)

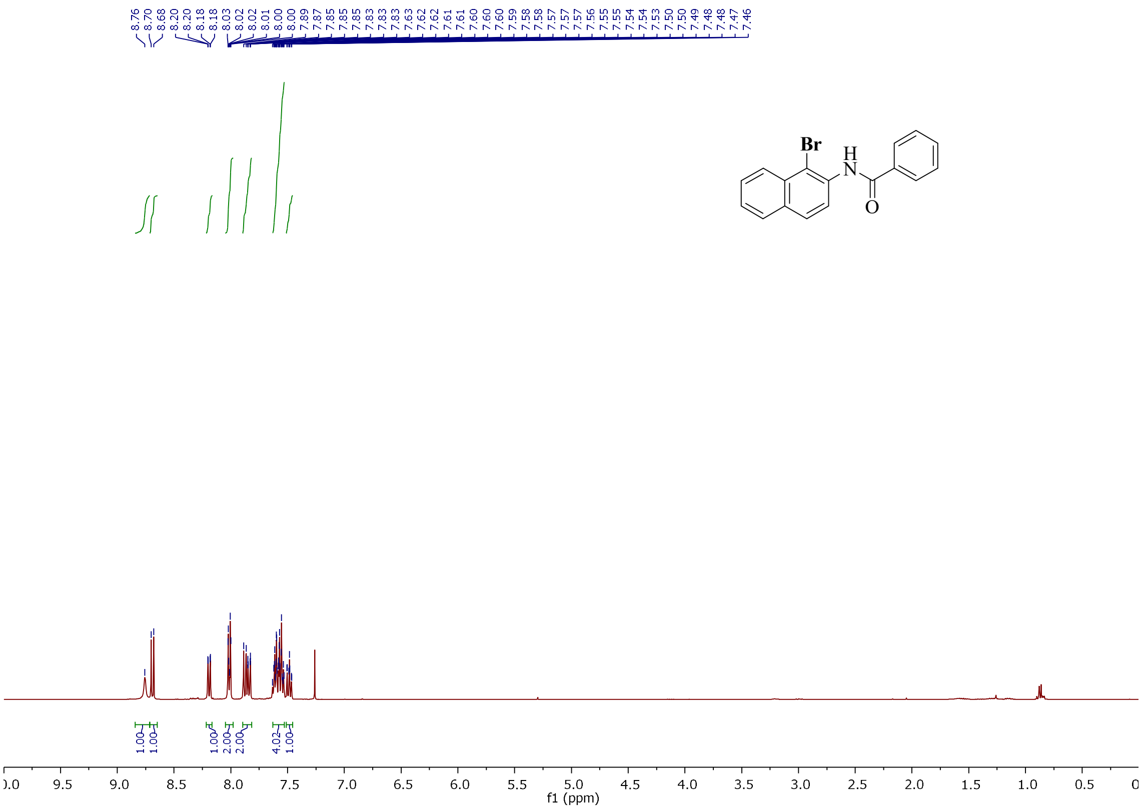

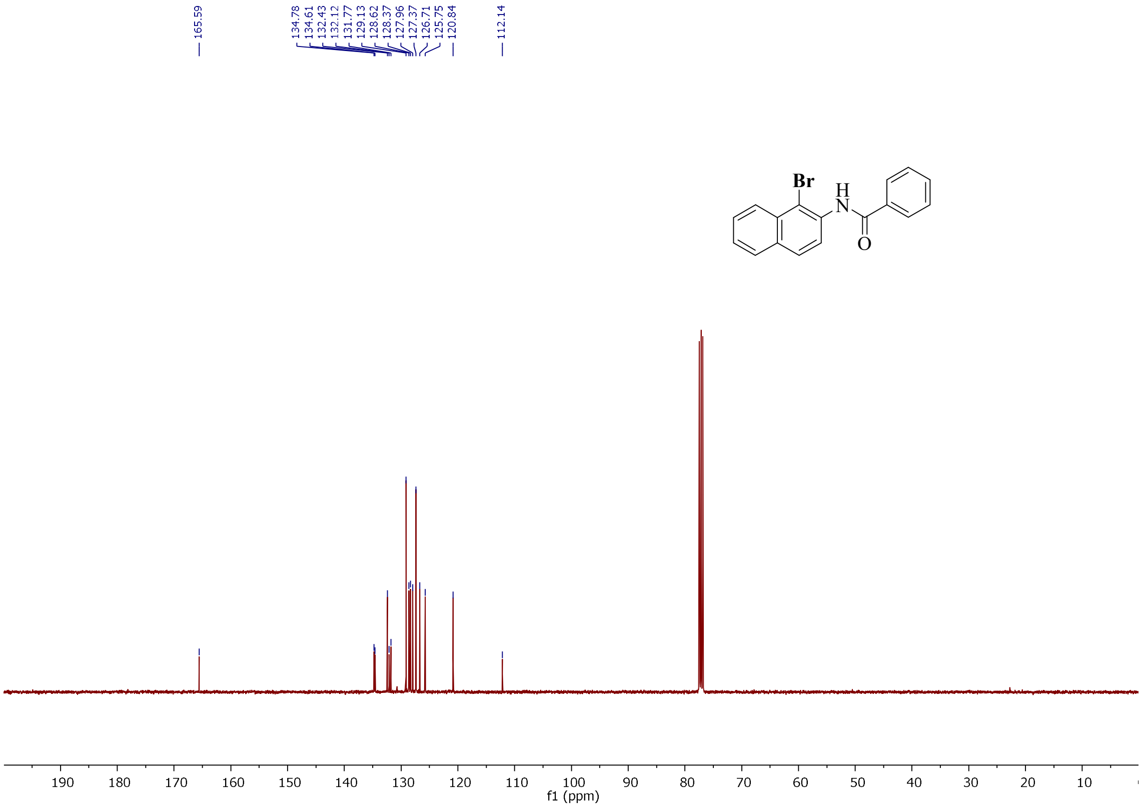

2-bromo-*N*-(1-bromonaphthalen-2-yl)acetamide: (92)

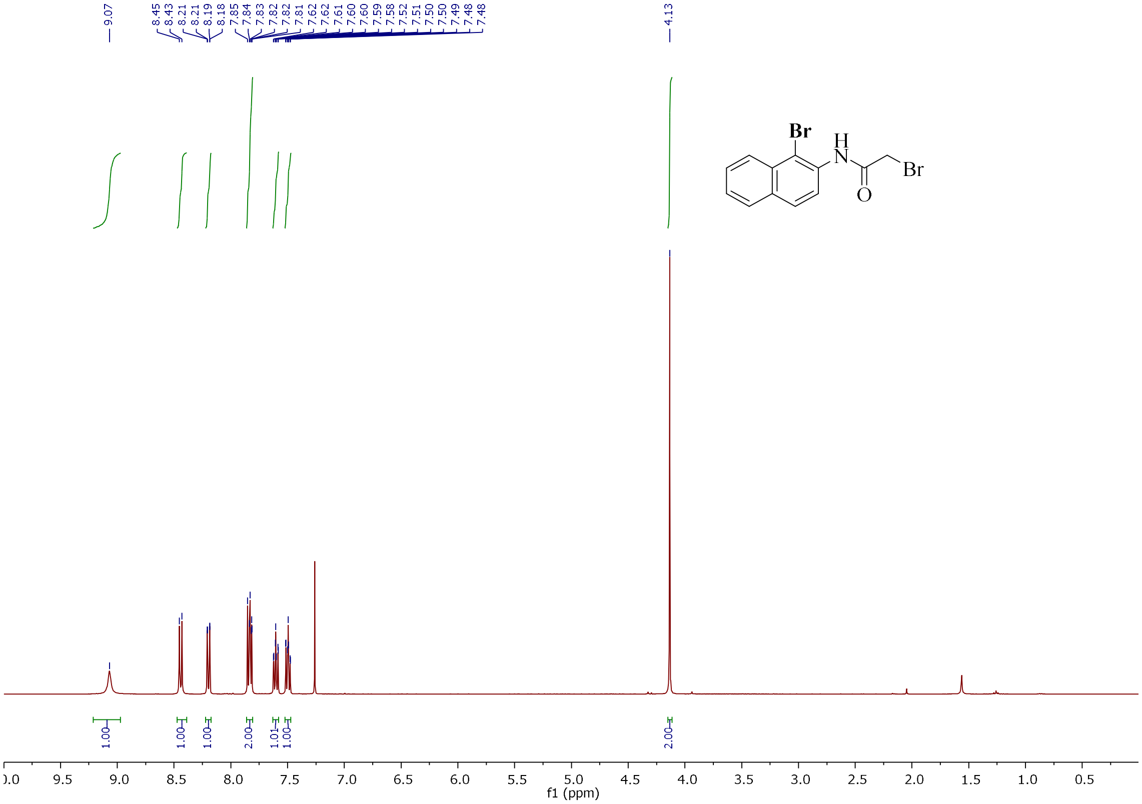

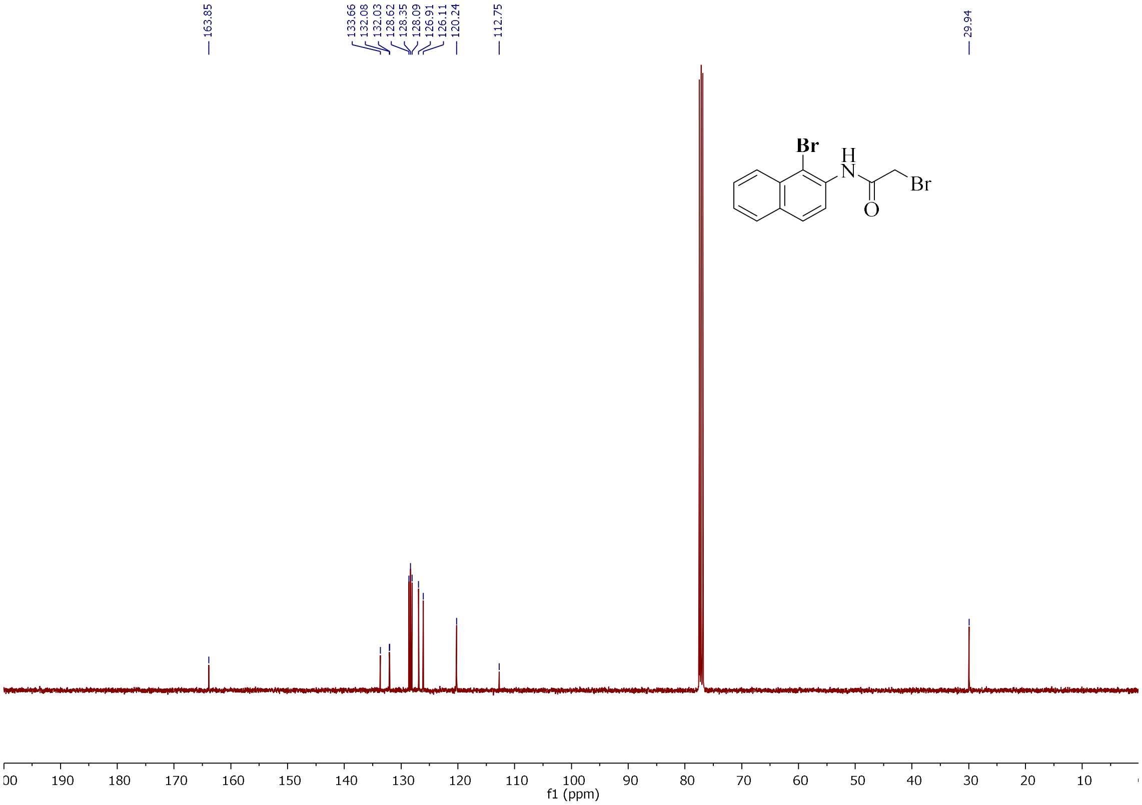

2-bromo-*N*-(1-bromonaphthalen-2-yl)propanamide: (93)

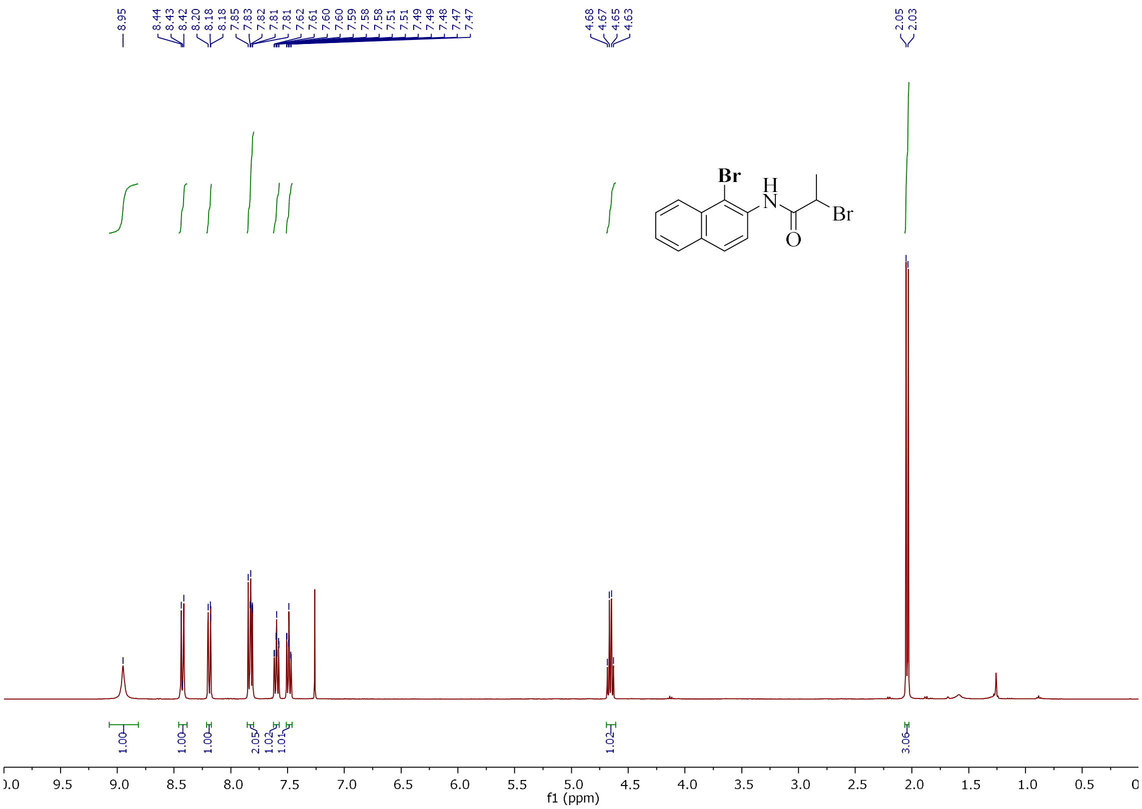

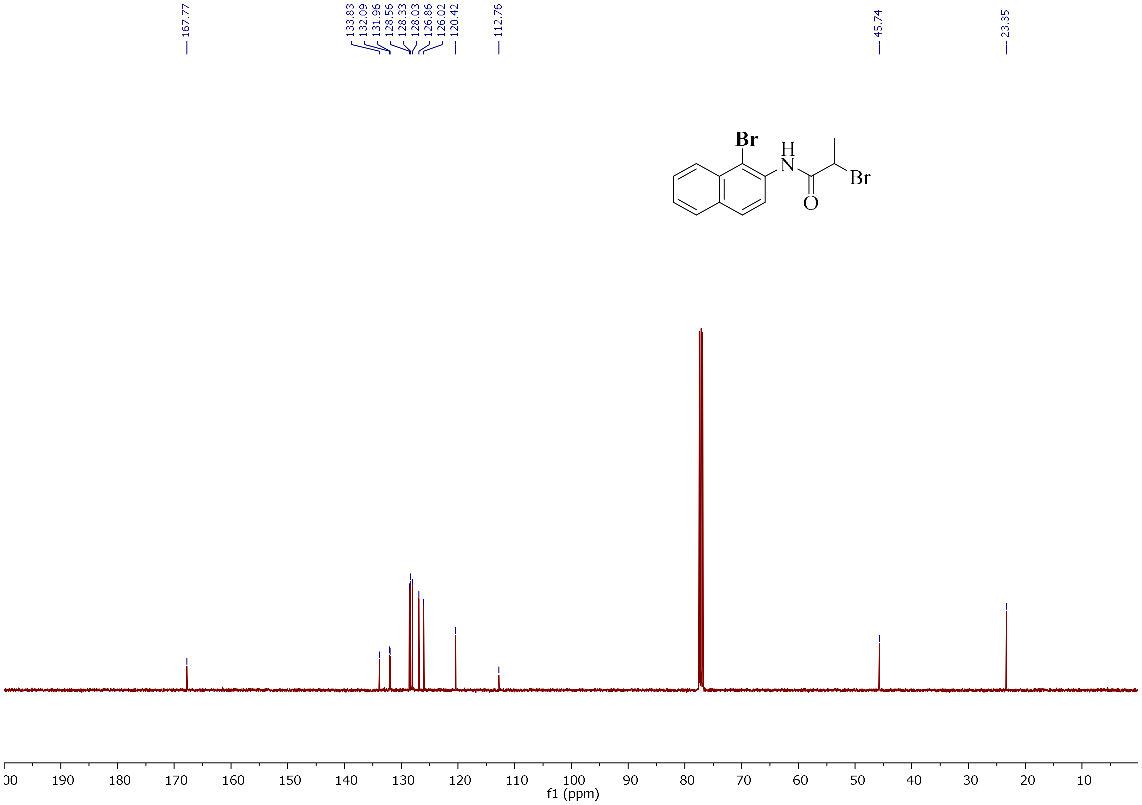

2-bromo-*N*-(1-bromonaphthalen-2-yl)-2-methylpropanamide: (94)

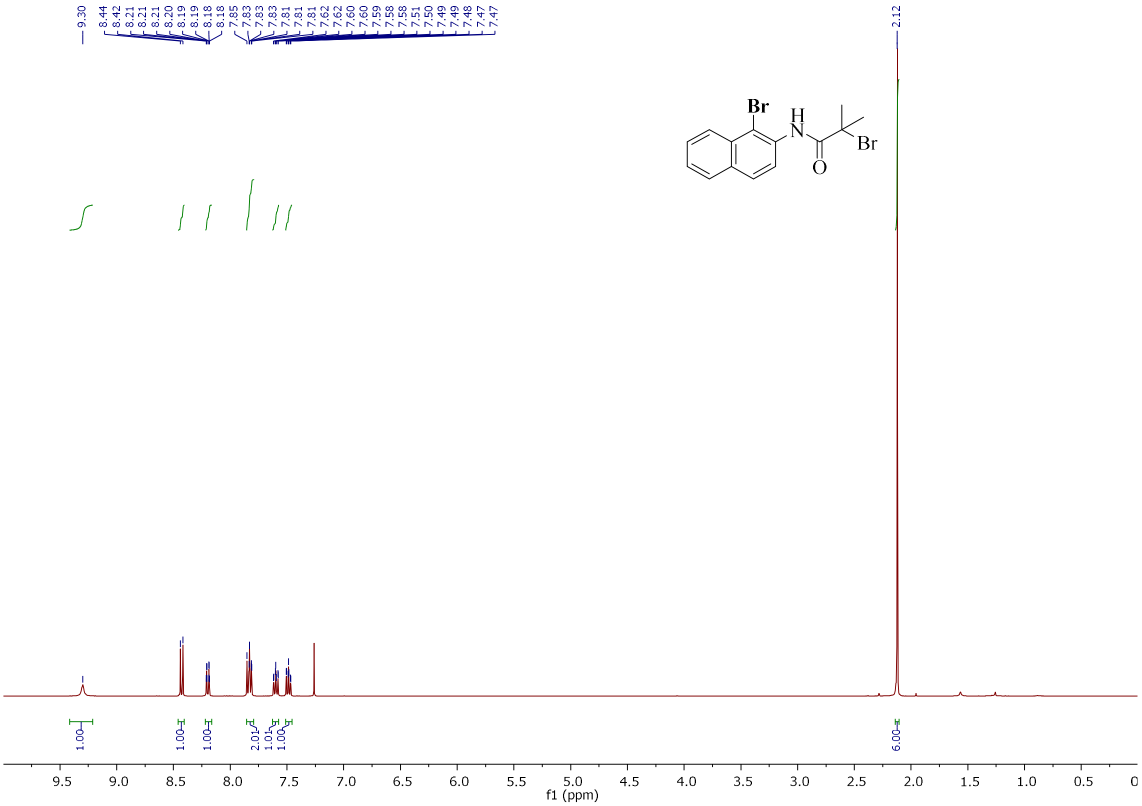

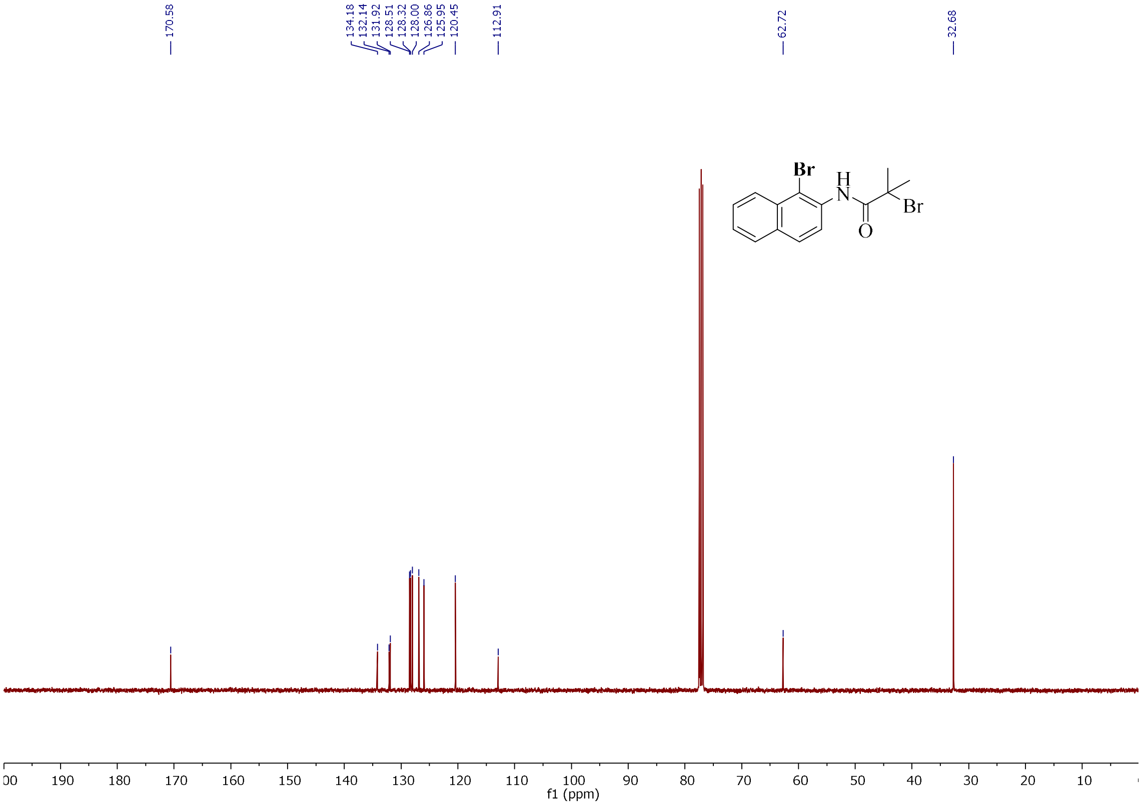

***N*-(2-bromo-4,5-dimethoxyphenyl)propionamide: (95)**

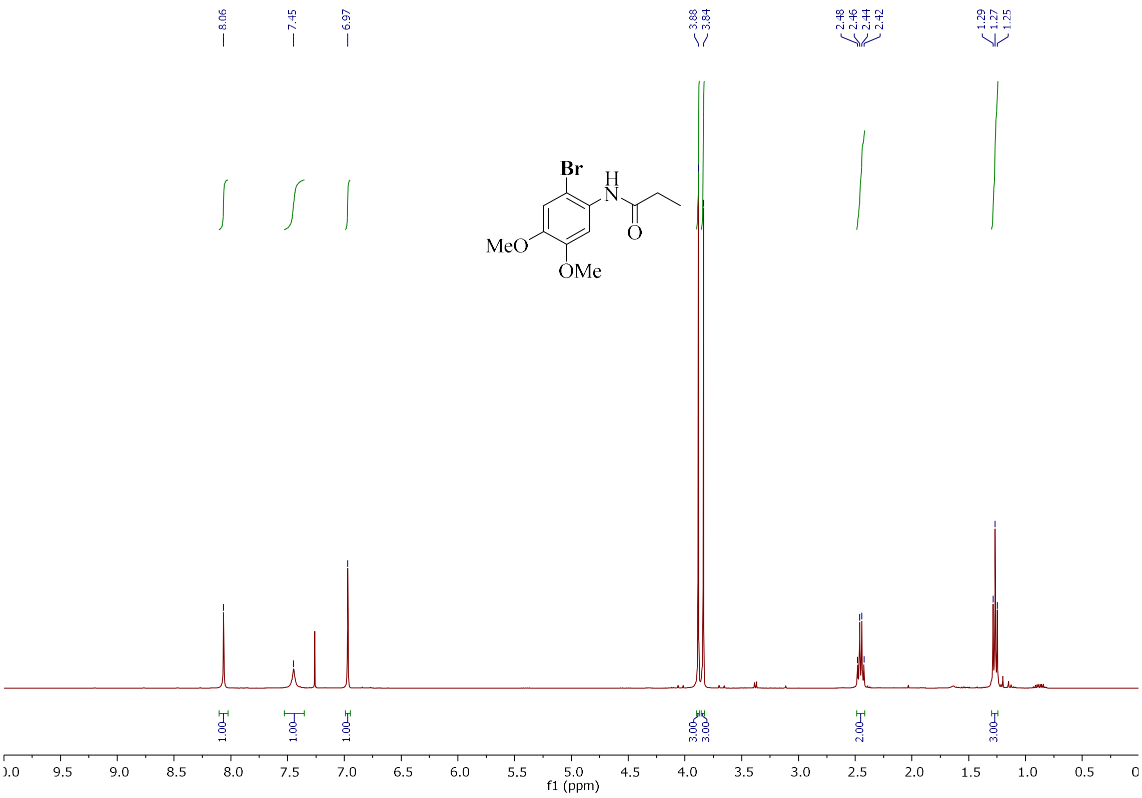

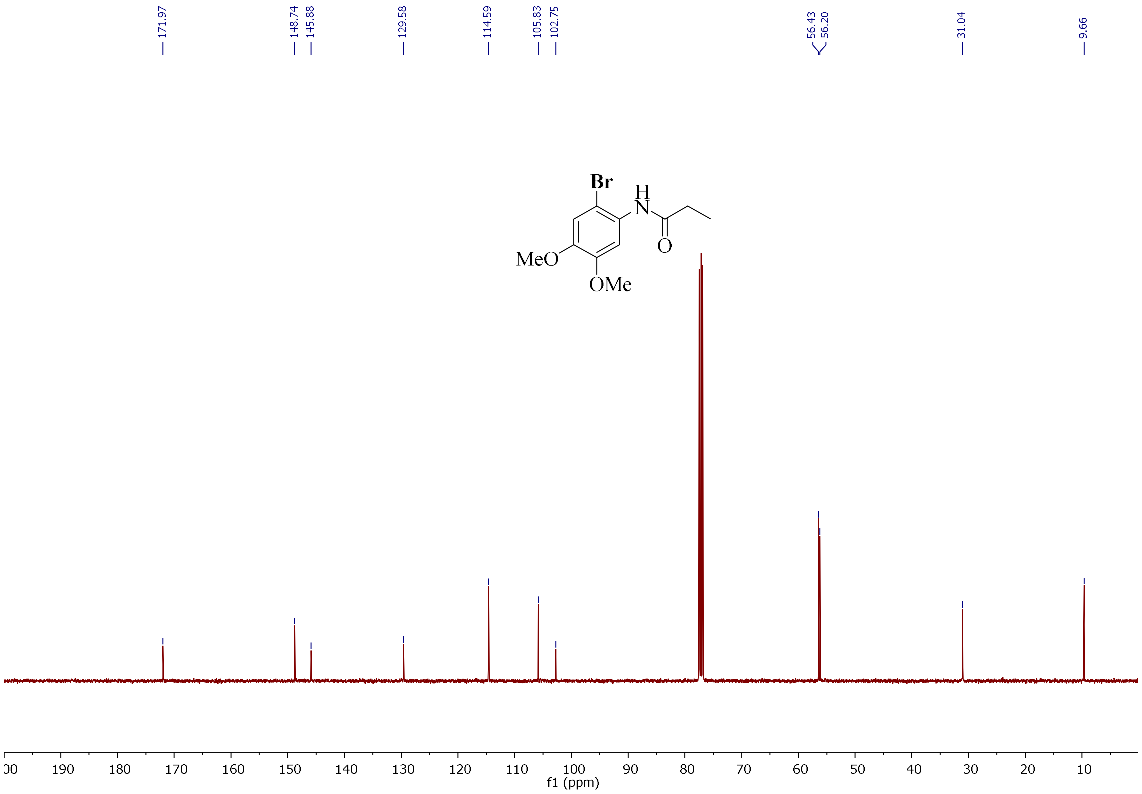

N-(2-bromo-4,5-dimethylphenyl)propionamide: (96)

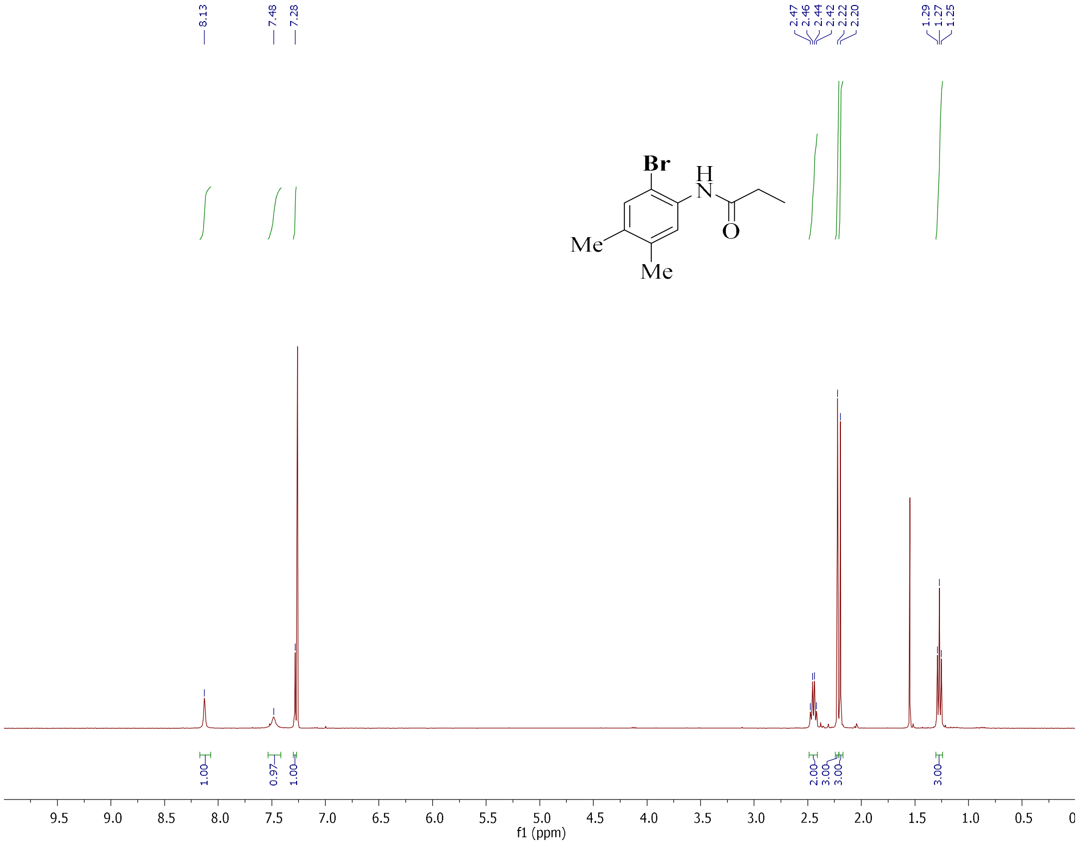

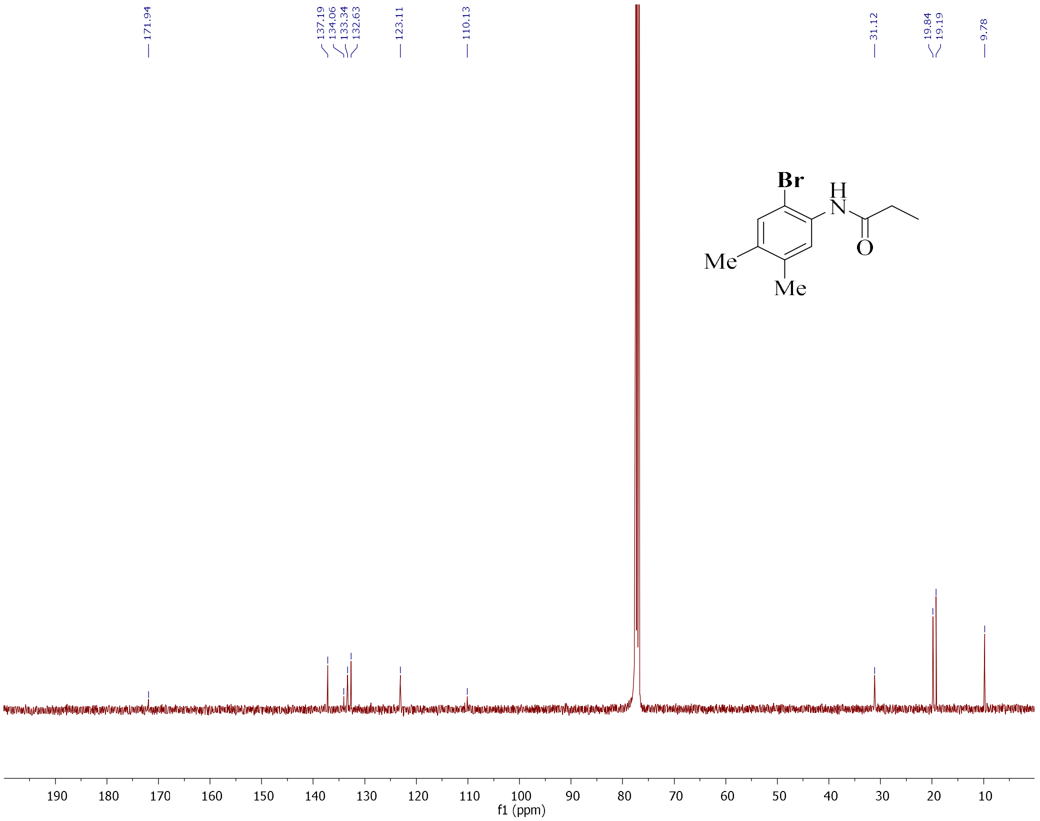

_N_-(2-bromo-3,4,5-trimethoxyphenyl)propionamide: (97)

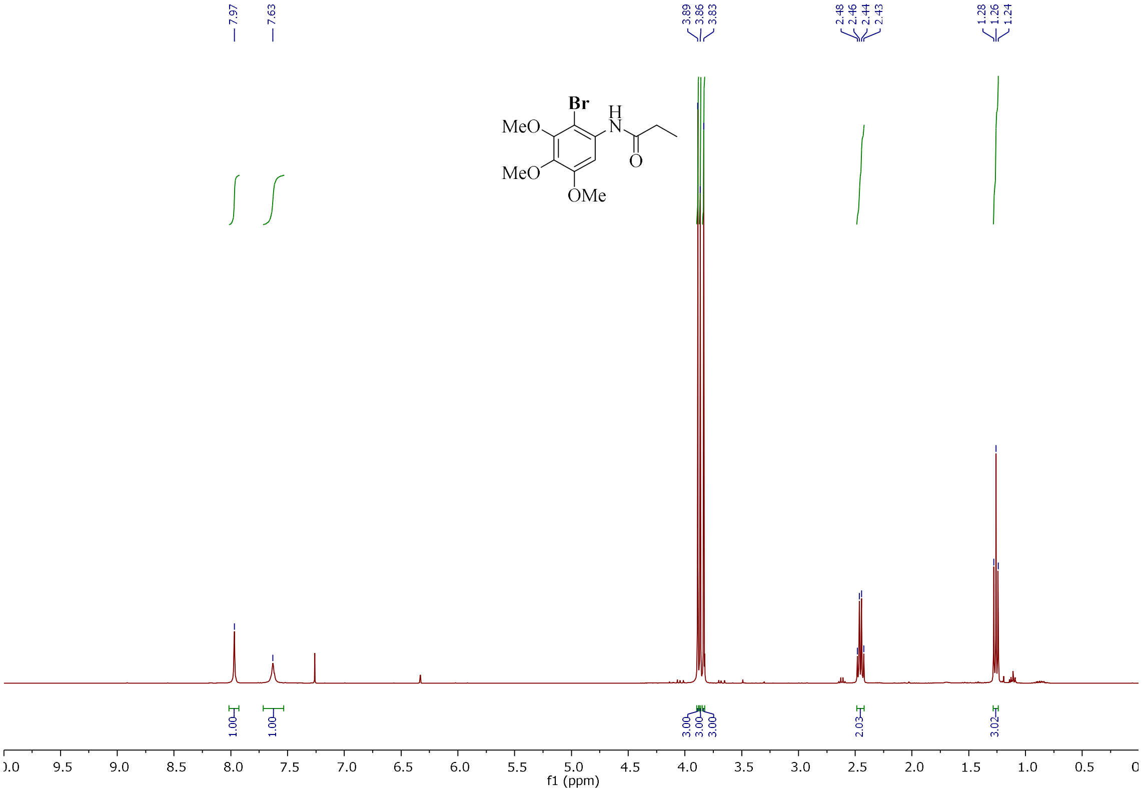

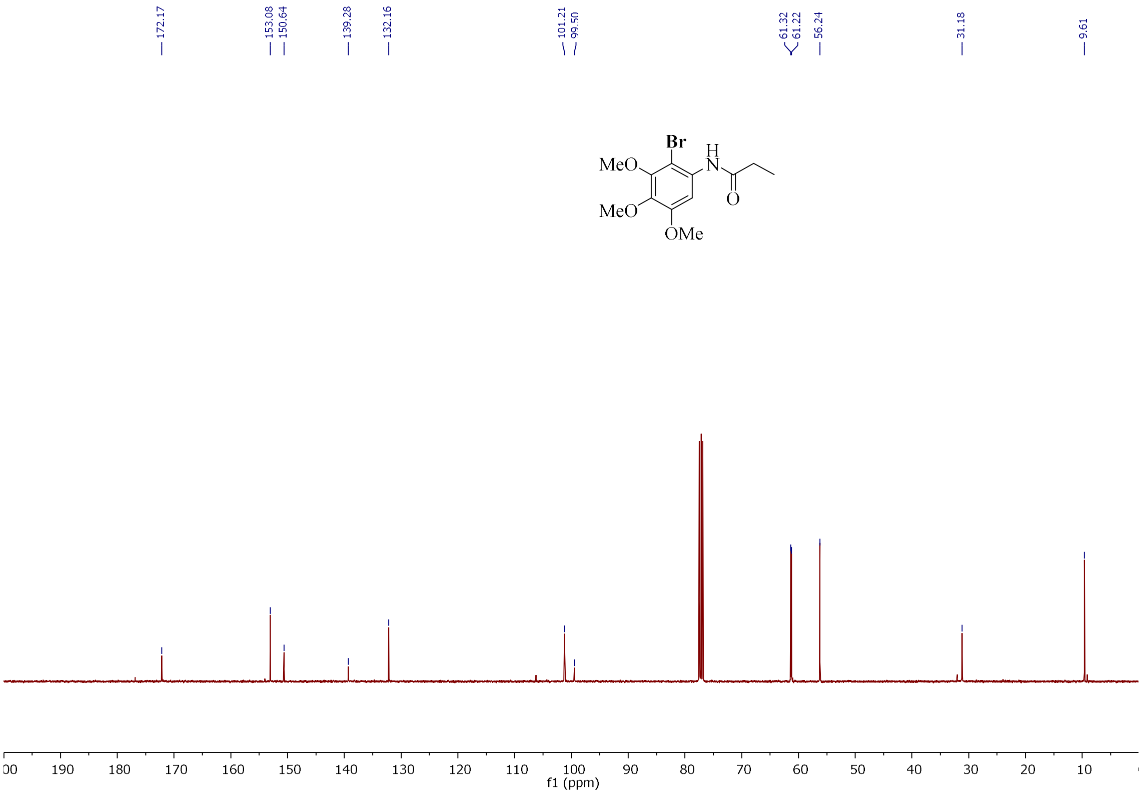

2-Bromo-*N*-(1-bromoanthracen-2-yl)propanamide: (98)

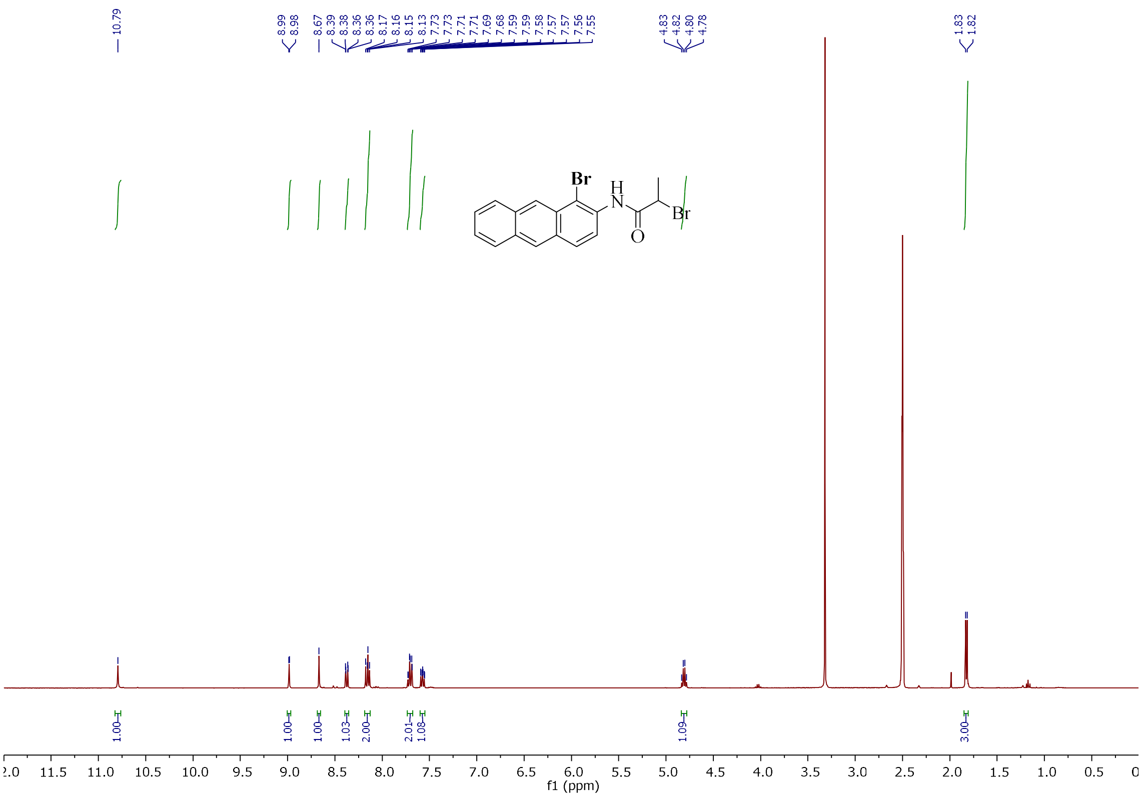

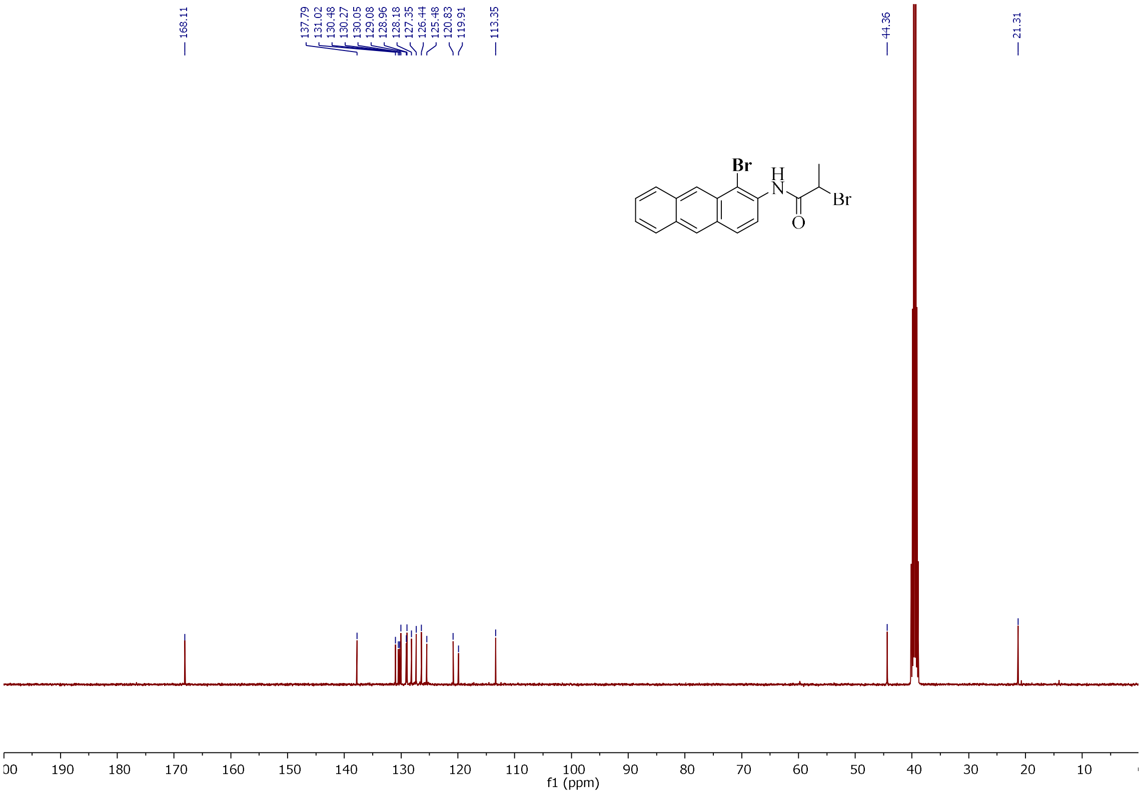

_N_-(4-iodophenyl)acetamide: (99)

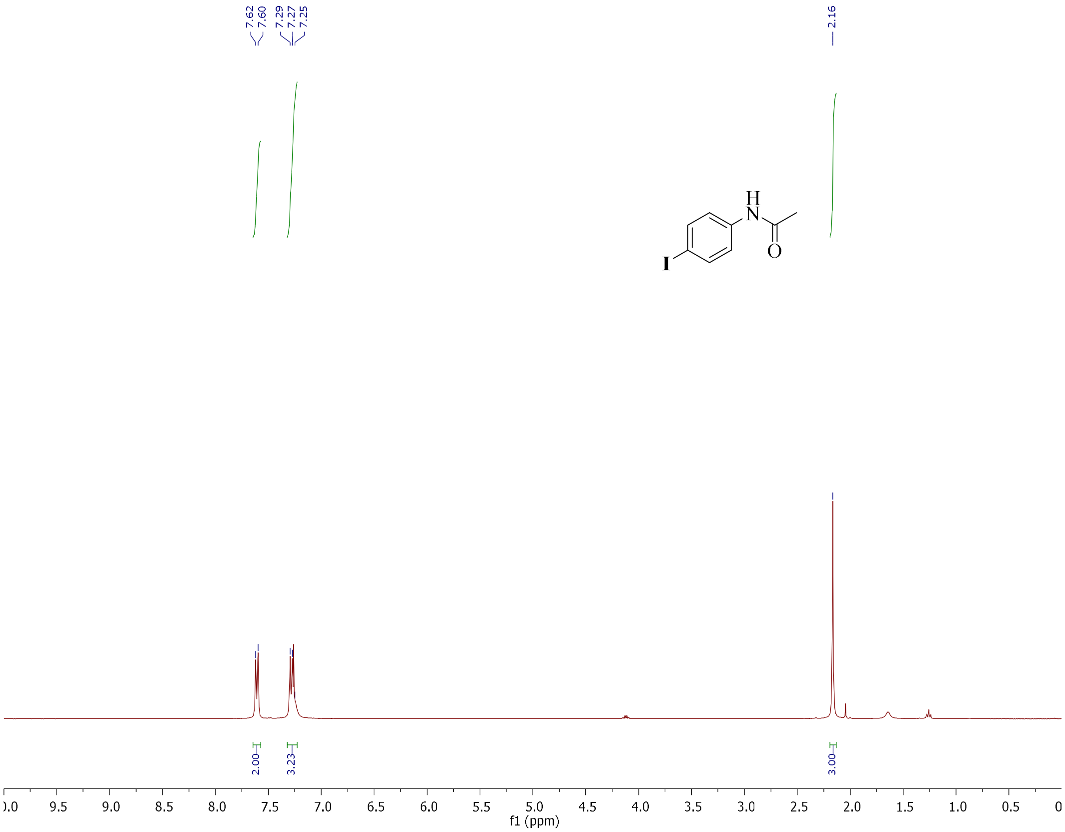

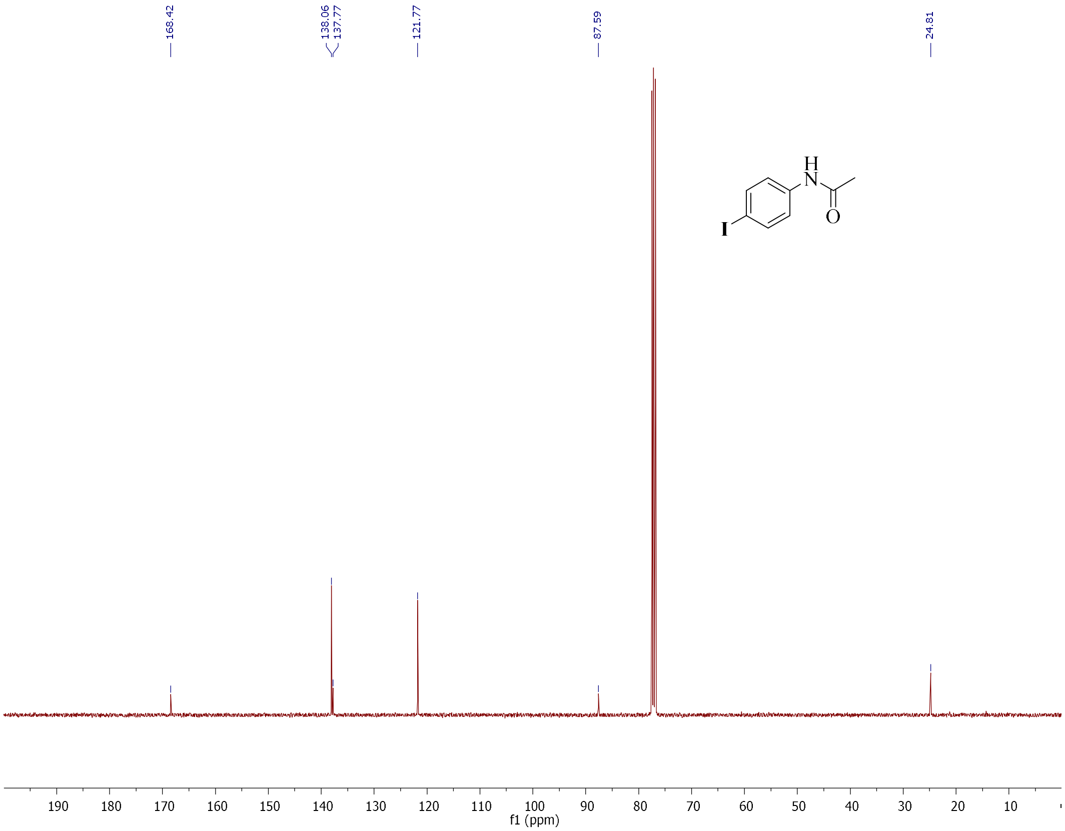

***N*-(4-iodo-3-methylphenyl)acetamide: (100)**

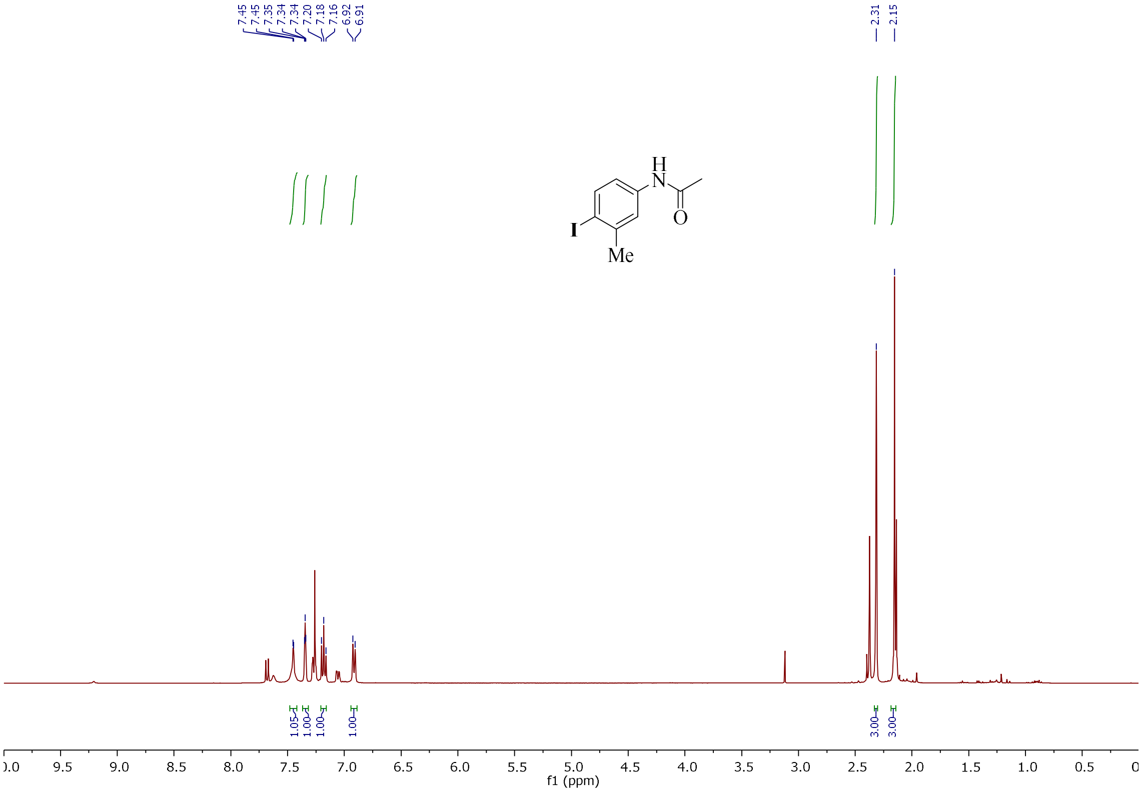

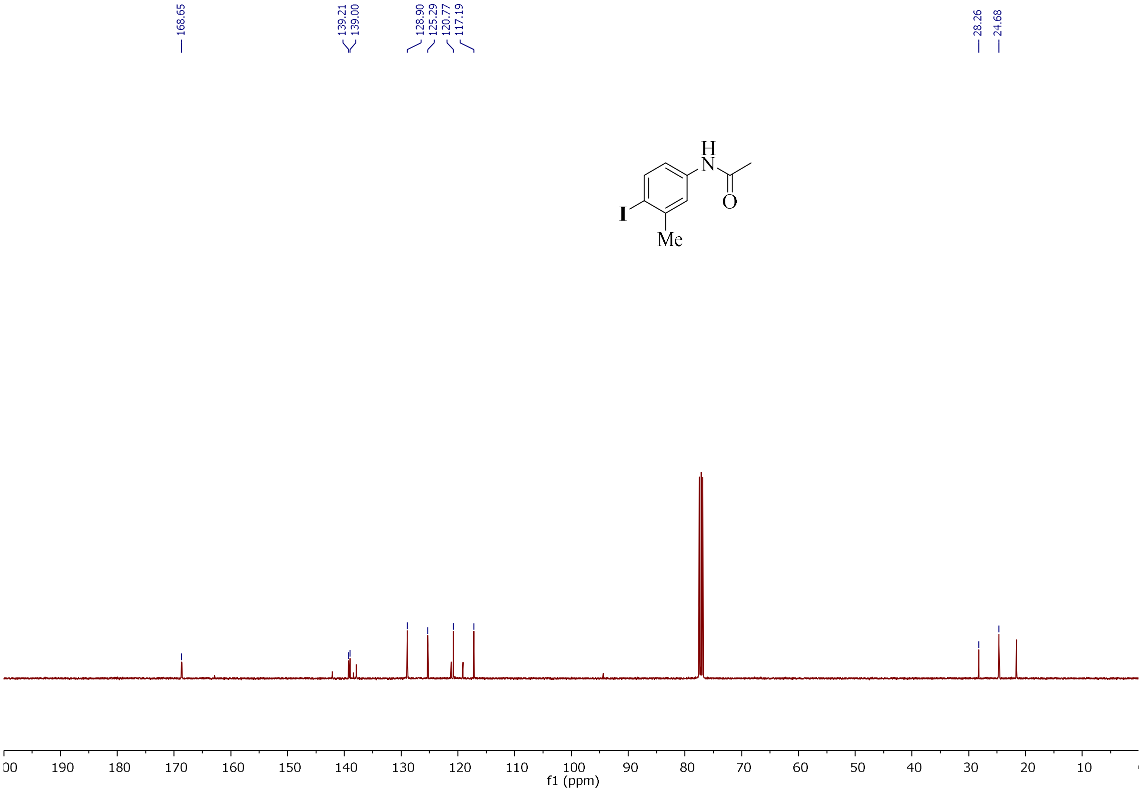

***N*-(2-iodo-5-methoxyphenyl)acetamide: (101)**

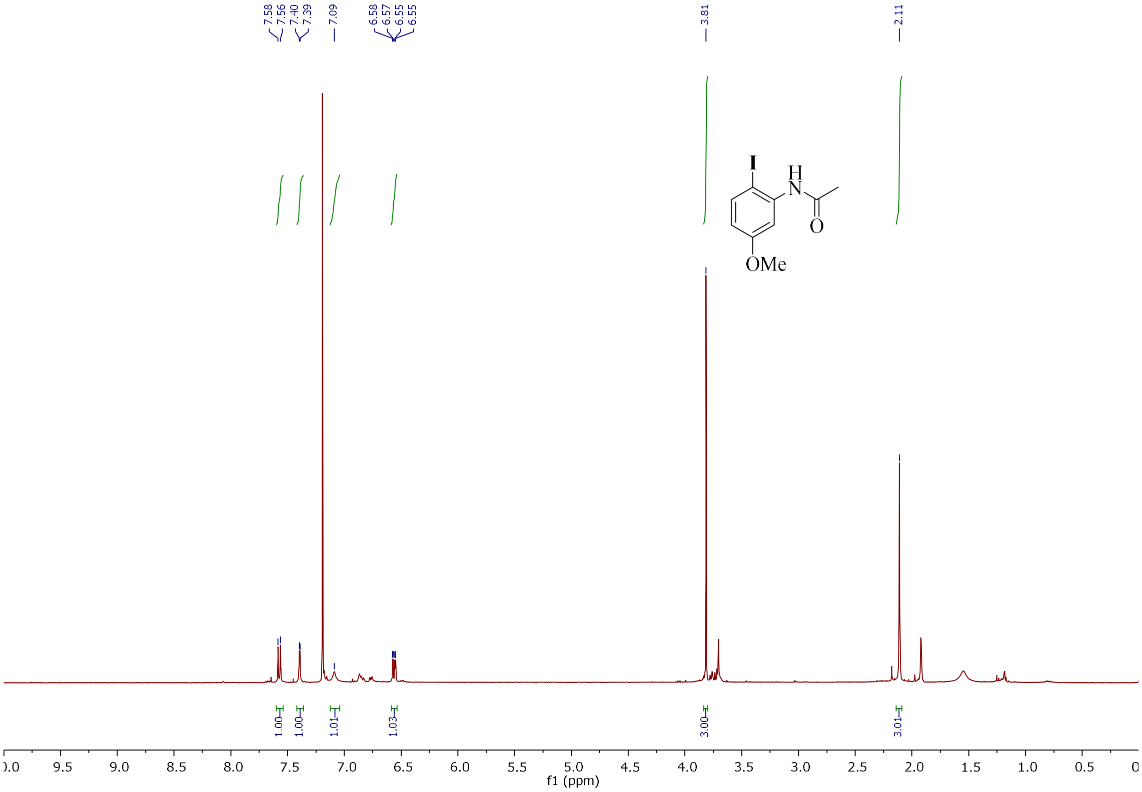

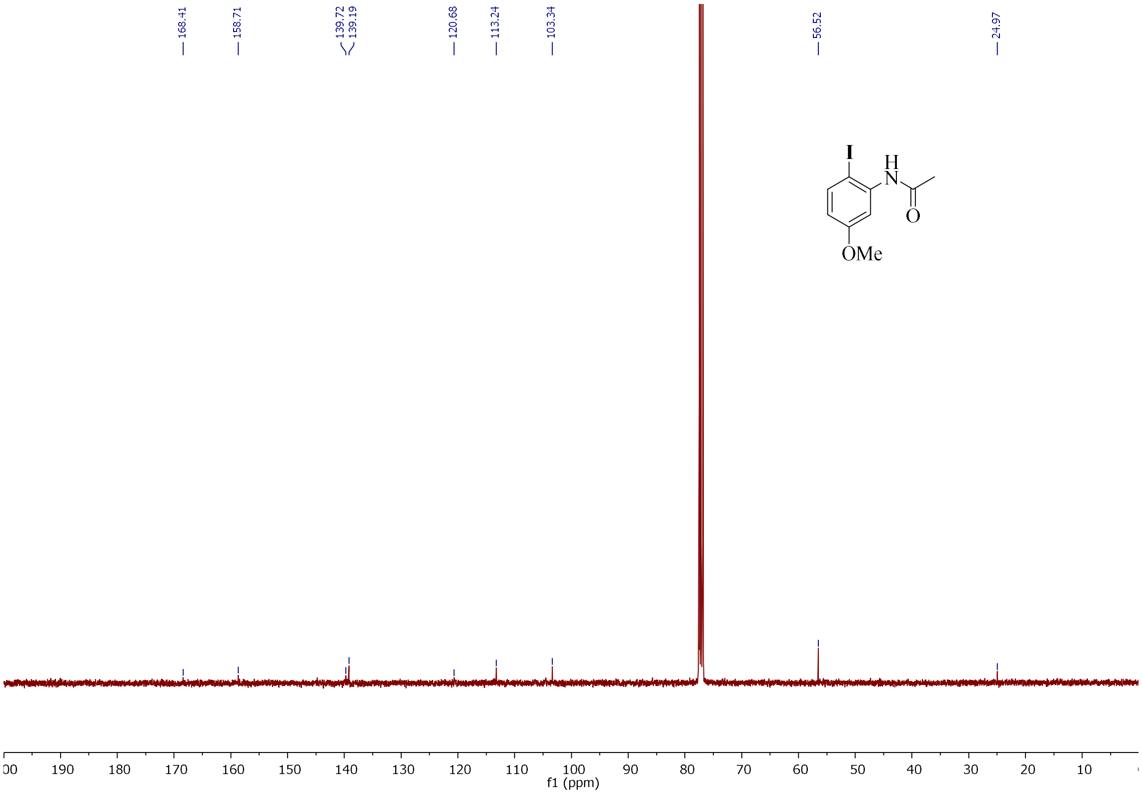

***N*-(2,4-dichloro-5-methoxyphenyl)cyclohexanecarboxamide: (15)**

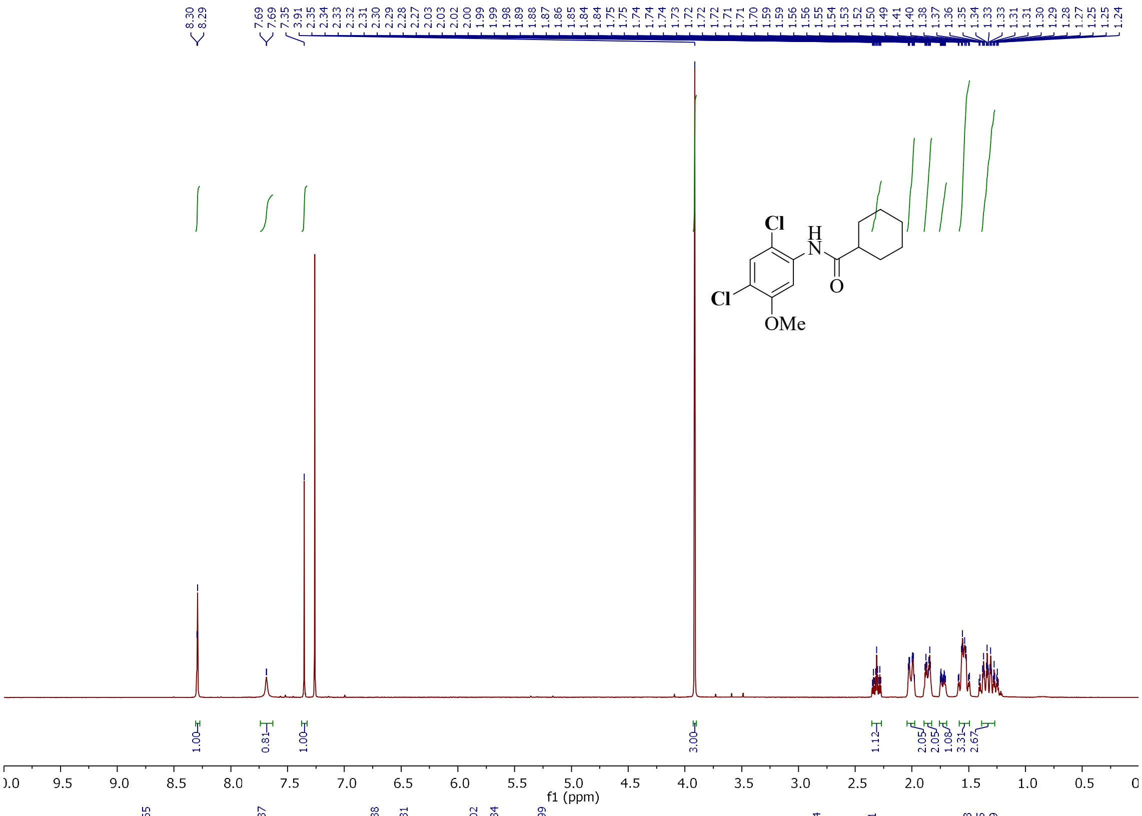

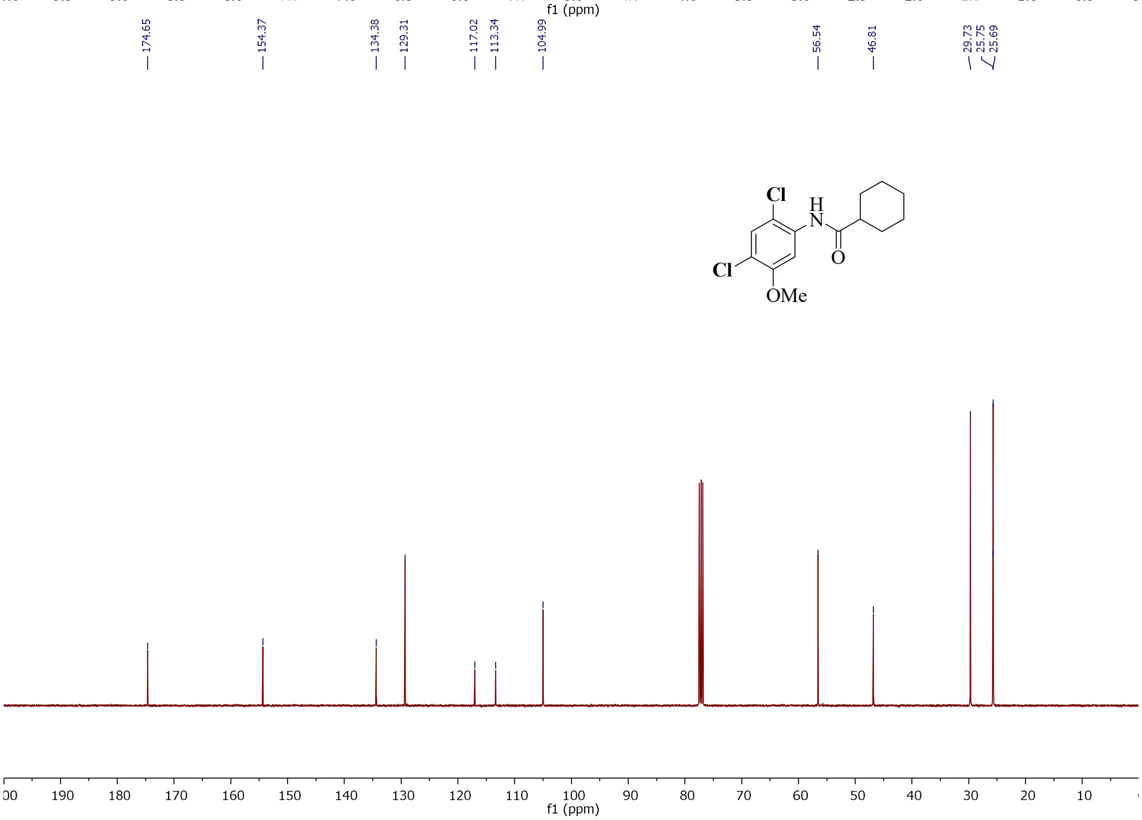

***N*-(2,4-dichloro-3,5-dimethylphenyl)cyclohexanecarboxamide: (102)**

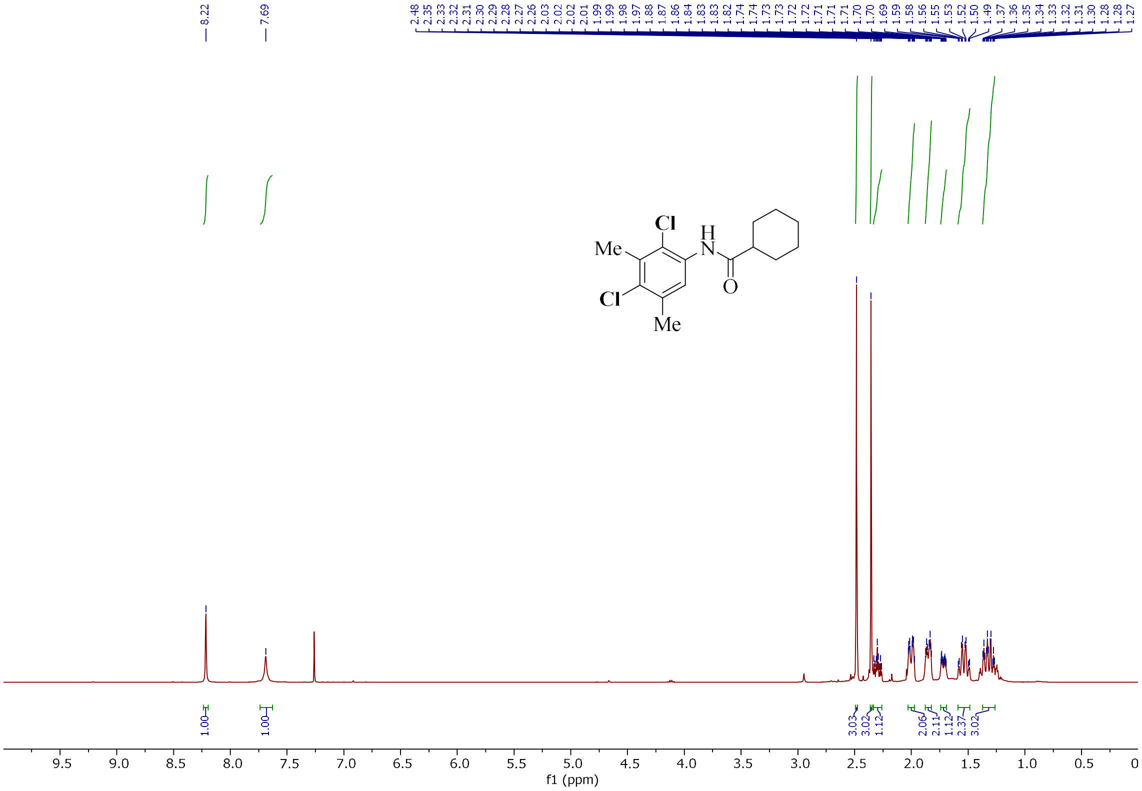

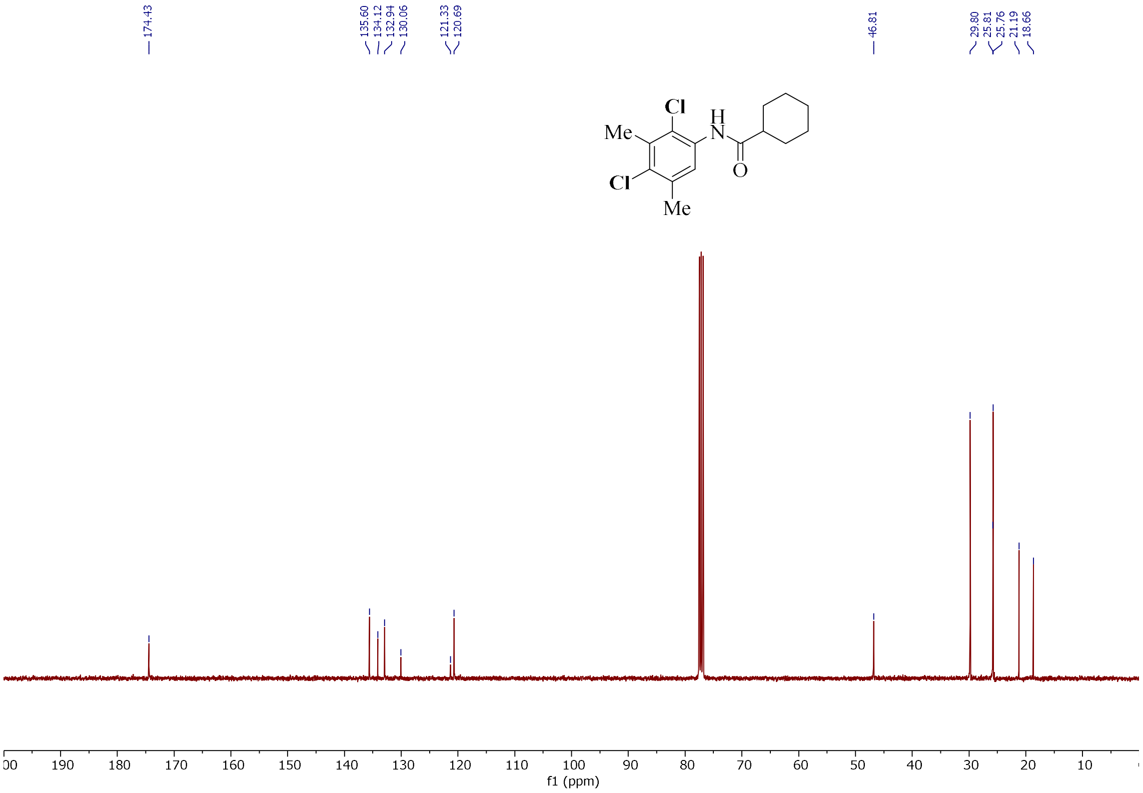

***N,N'*-(4,5-dichloro-1,2-phenylene)dicyclohexanecarboxamide: (103)**

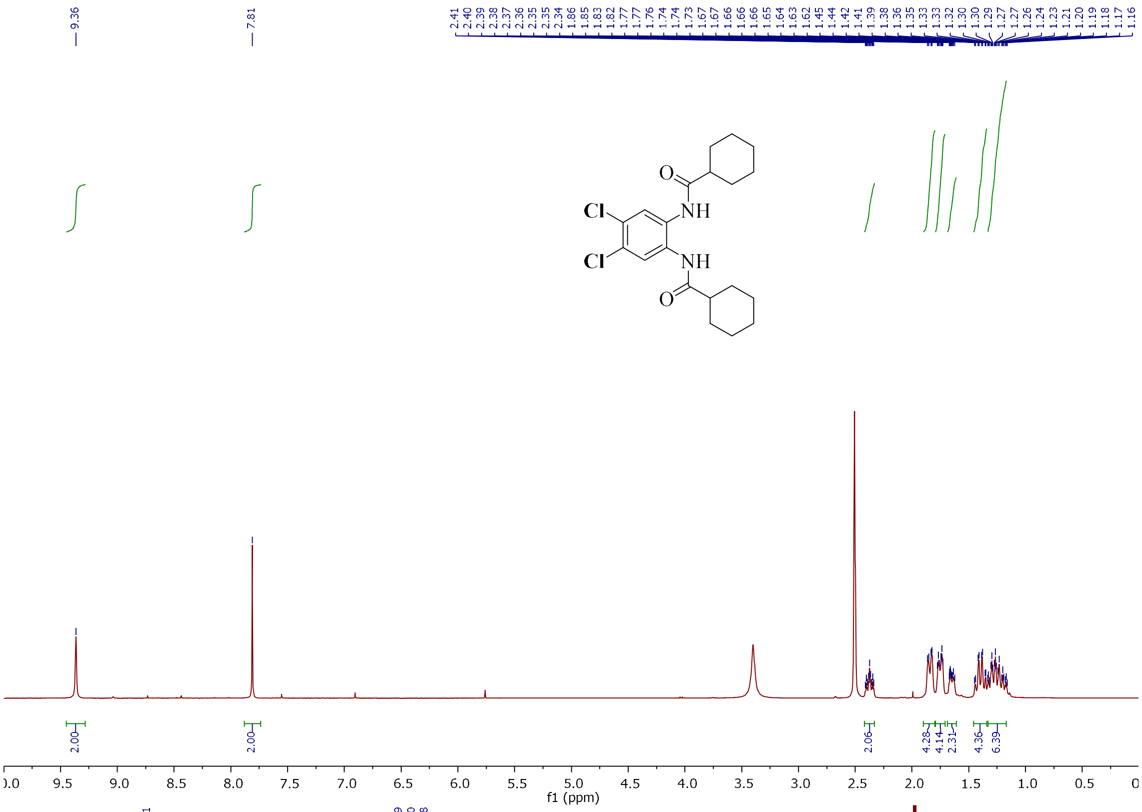

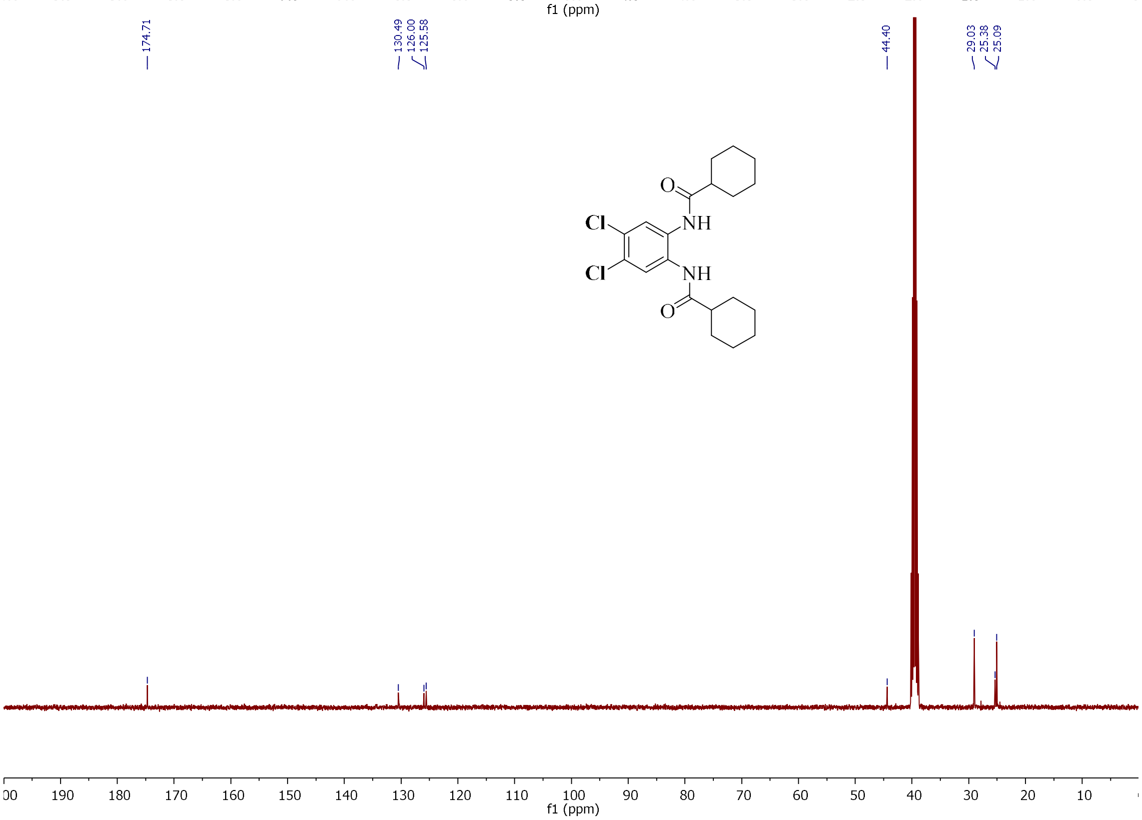

***N*-(2,4-dichlorophenyl)cyclopropanecarboxamide: (104)**

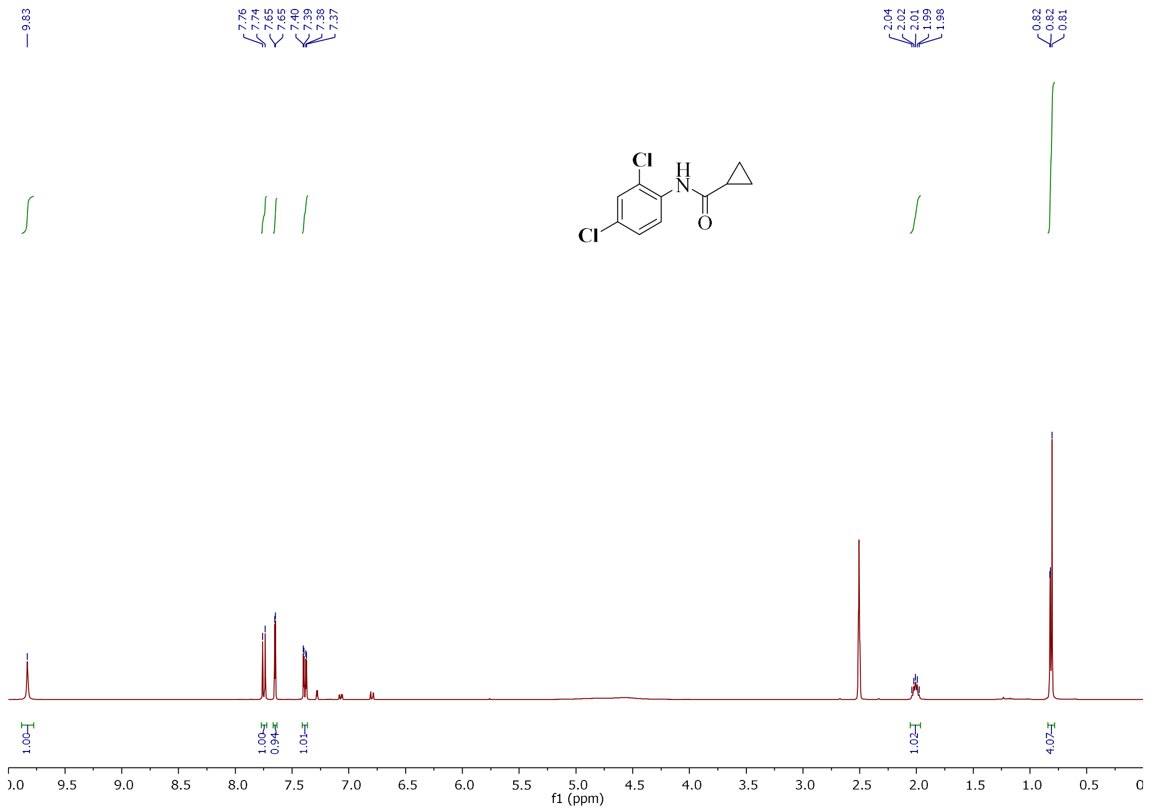

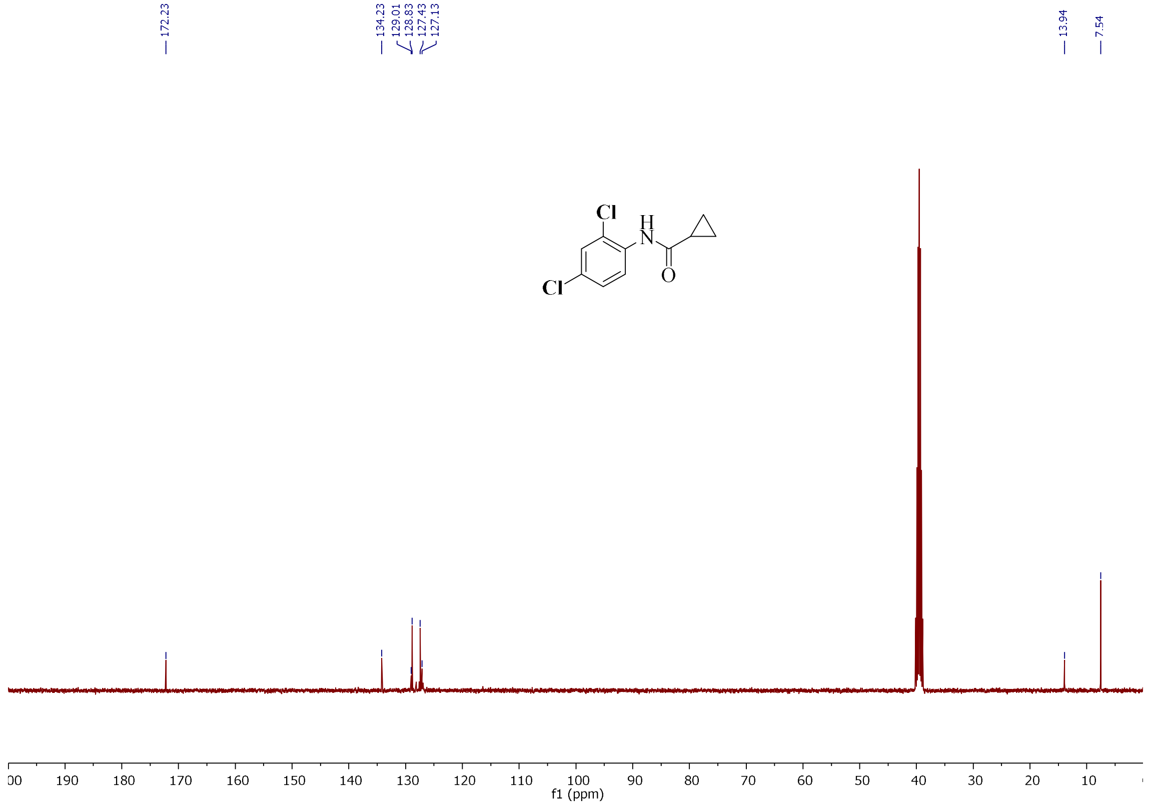

_N_-(2,4-dichlorophenyl)cyclobutanecarboxamide: (105)

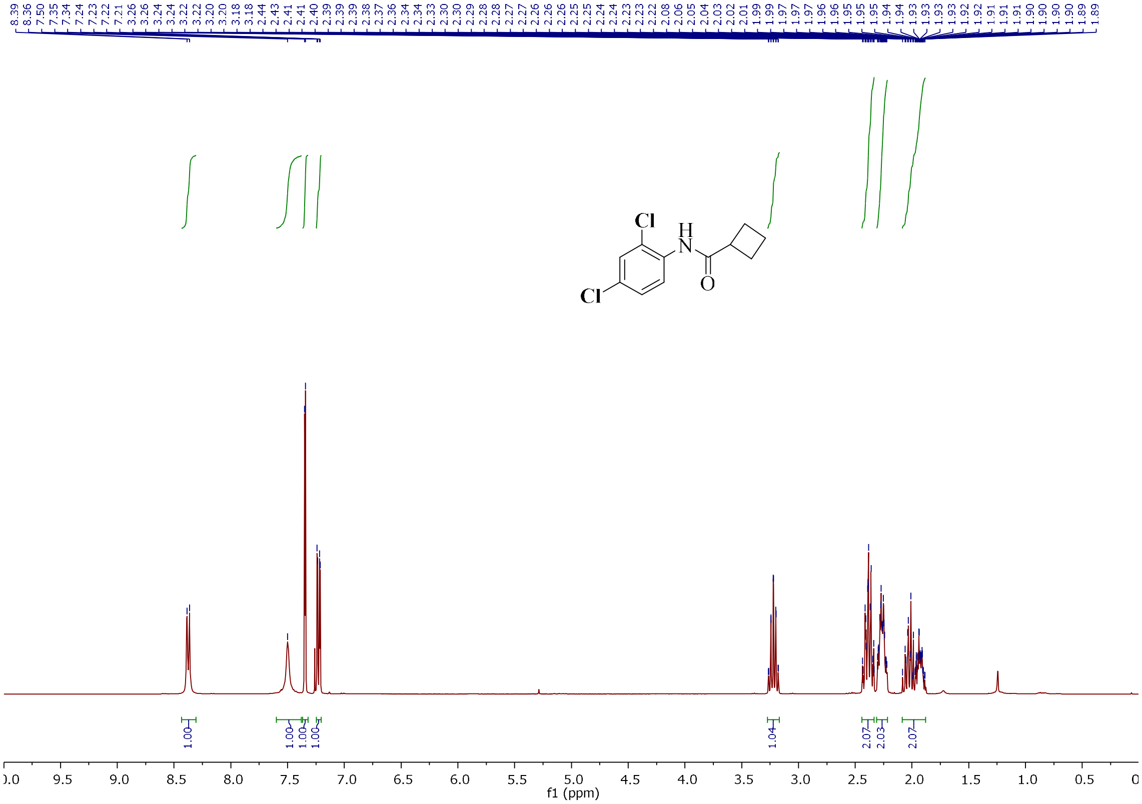

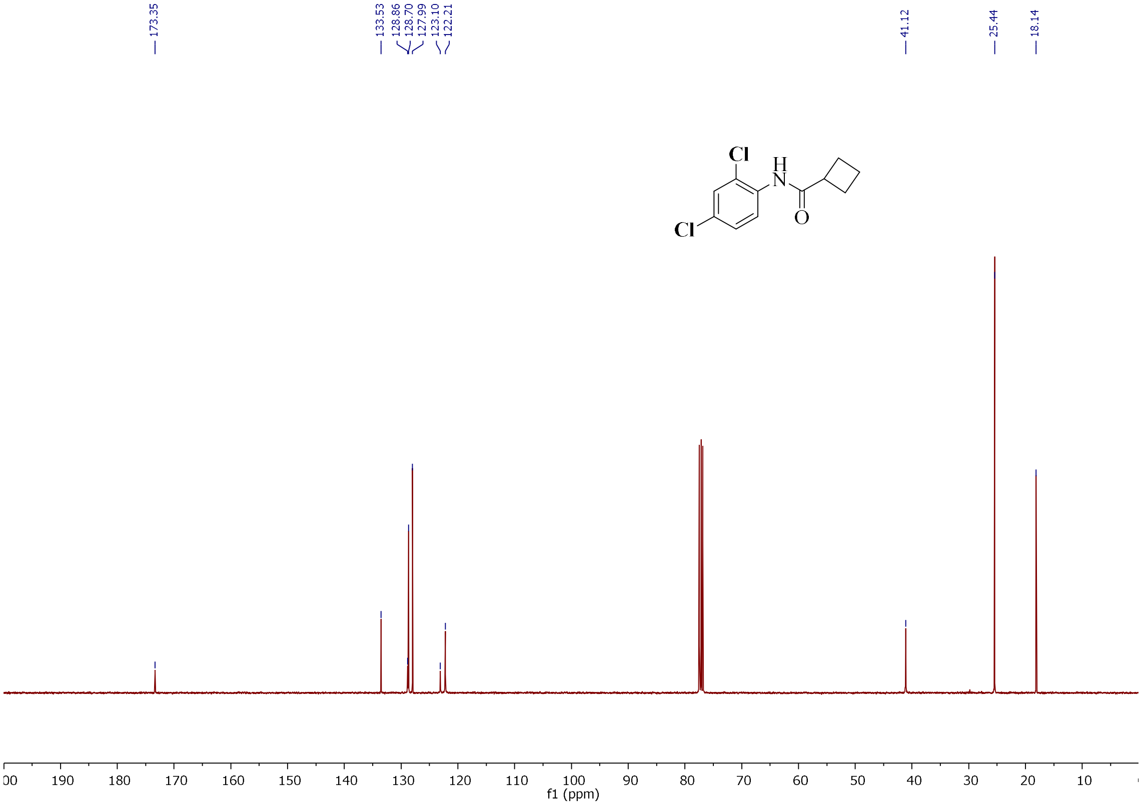

N-(2,4-dichlorophenyl)cyclopentanecarboxamide: (106)

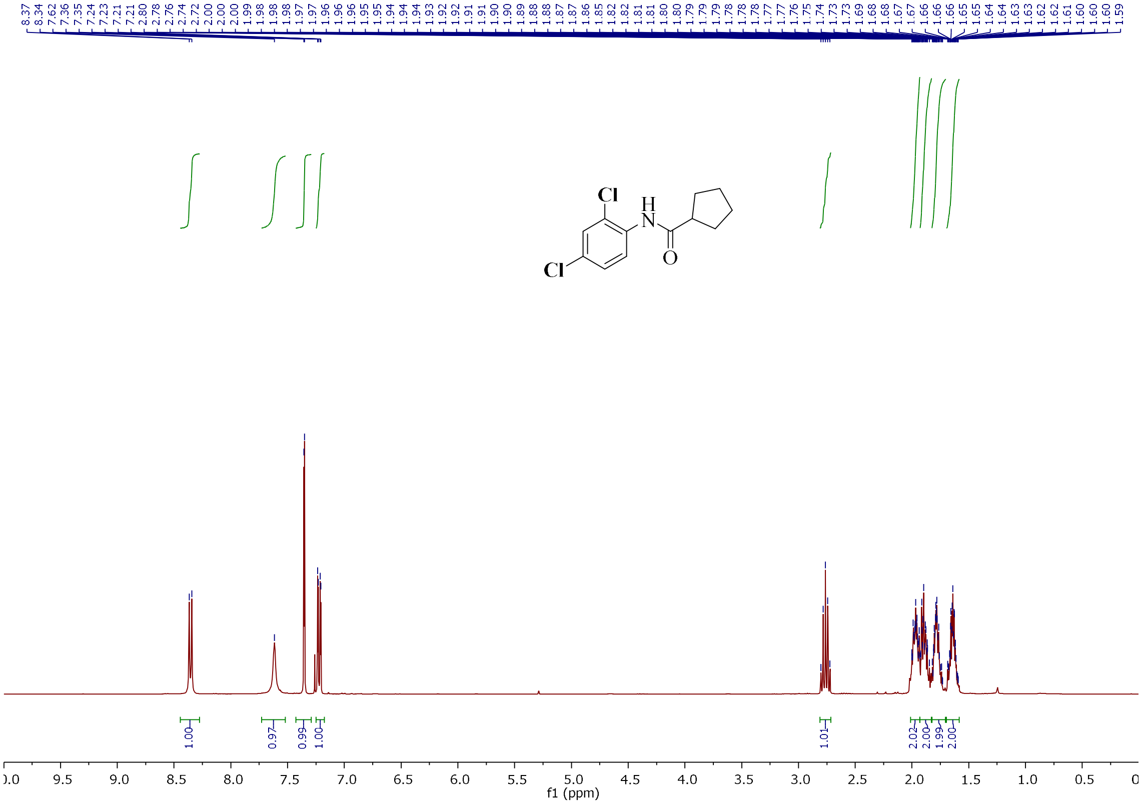

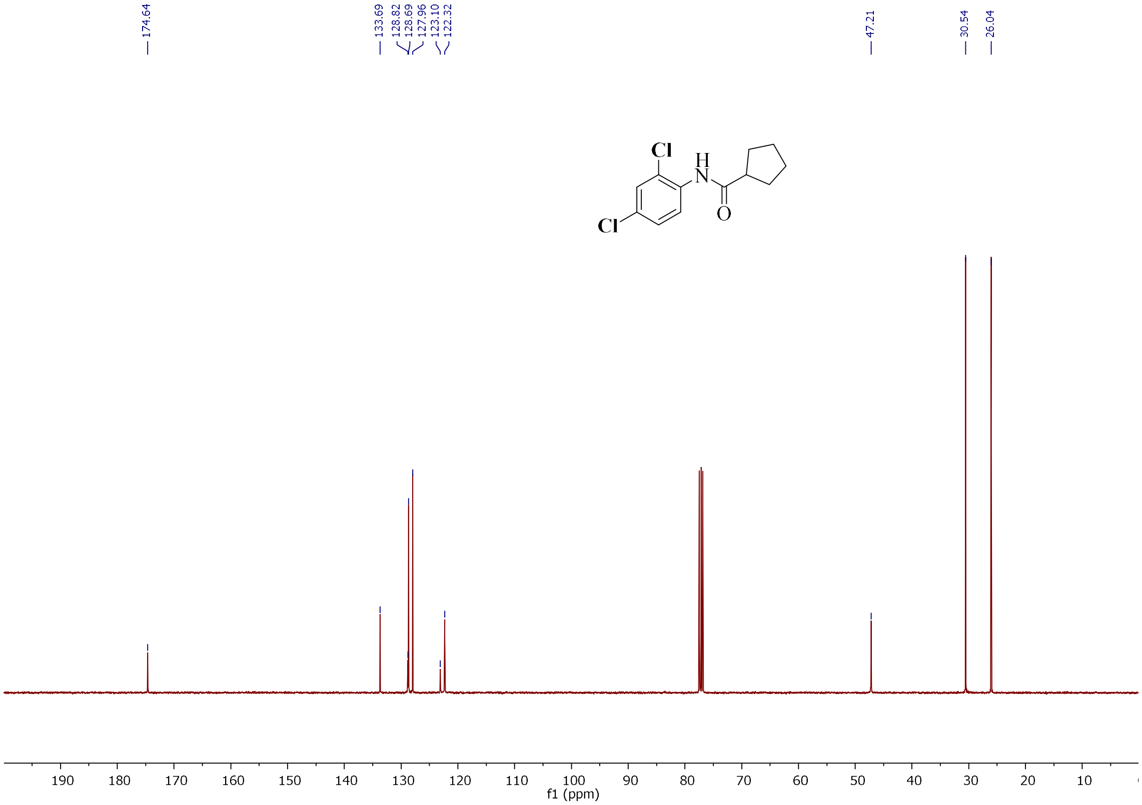

***N*-(2,4-dichlorophenyl)cyclohexanecarboxamide: (4)**

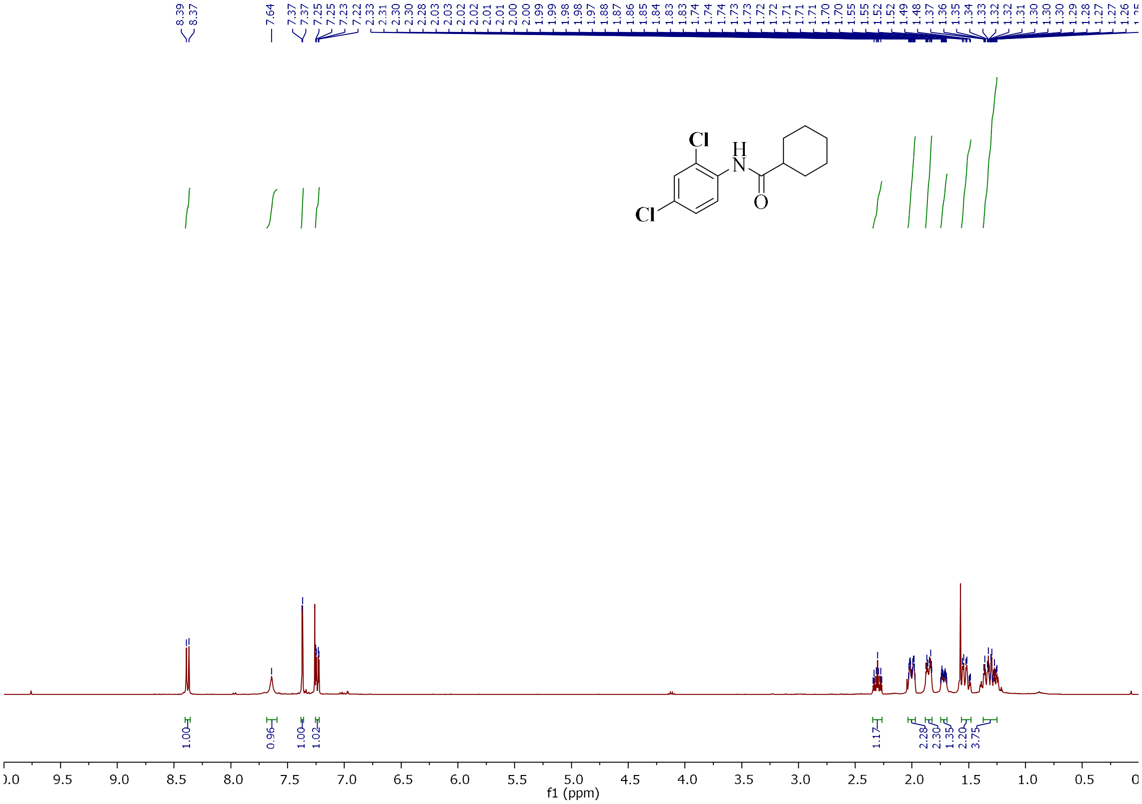

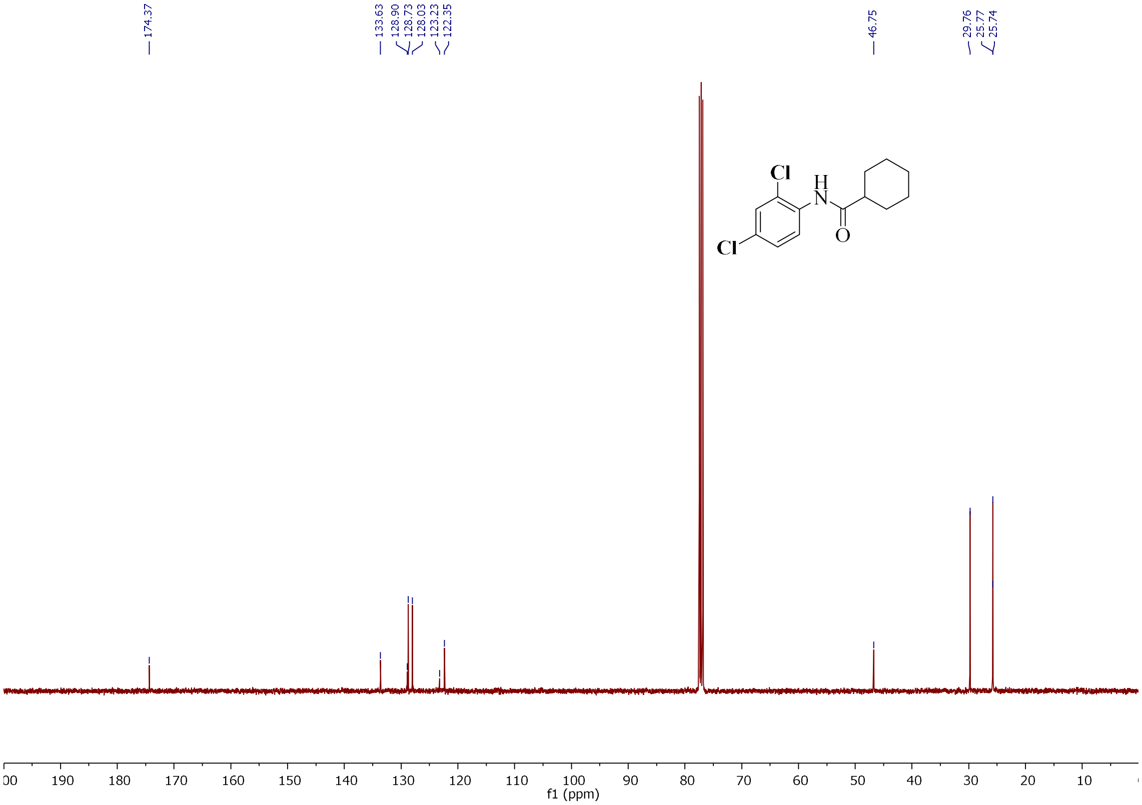

N-(2,4-dichlorophenyl)cycloheptanecarboxamide: (107)

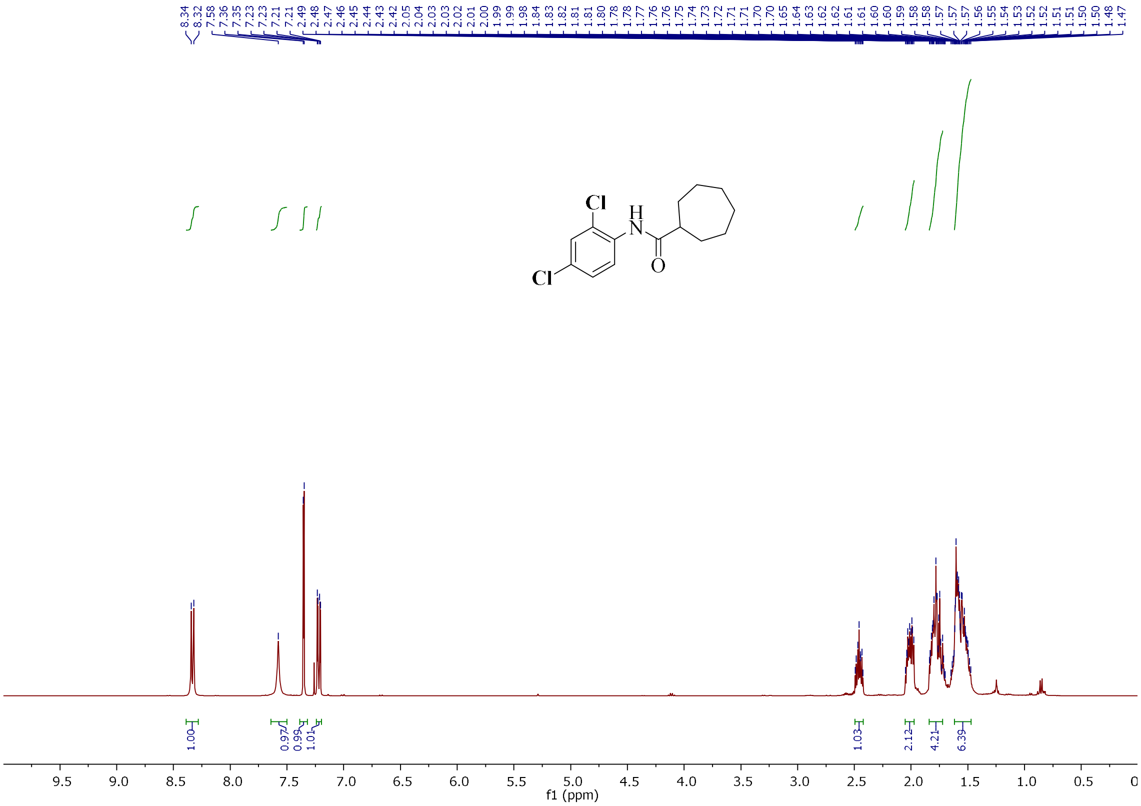

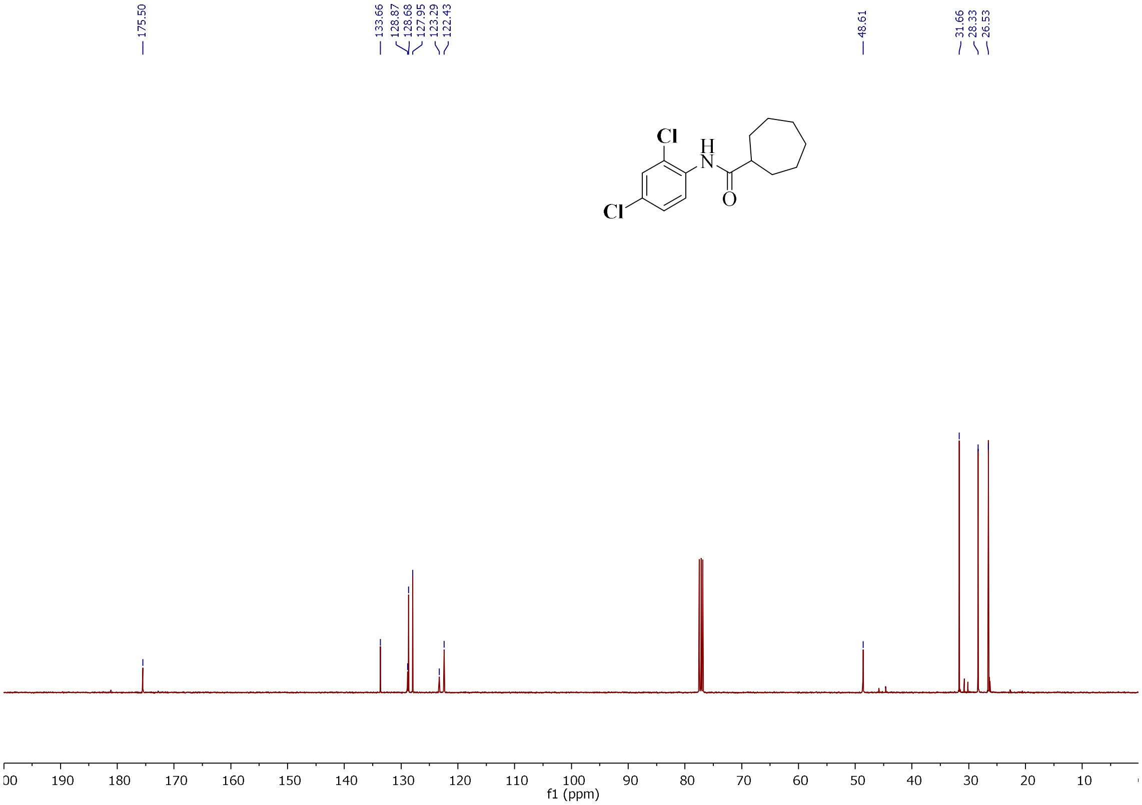

***N*-(2,4-dichlorophenyl)hexanamide: (108)**

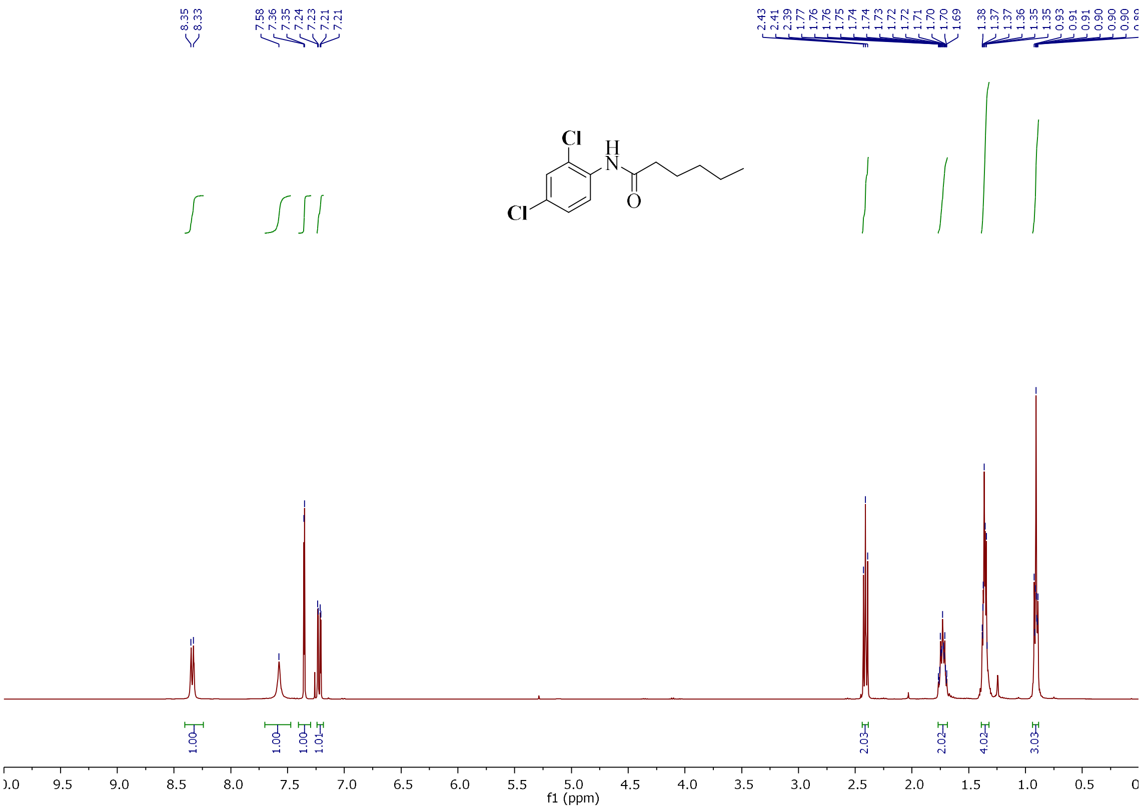

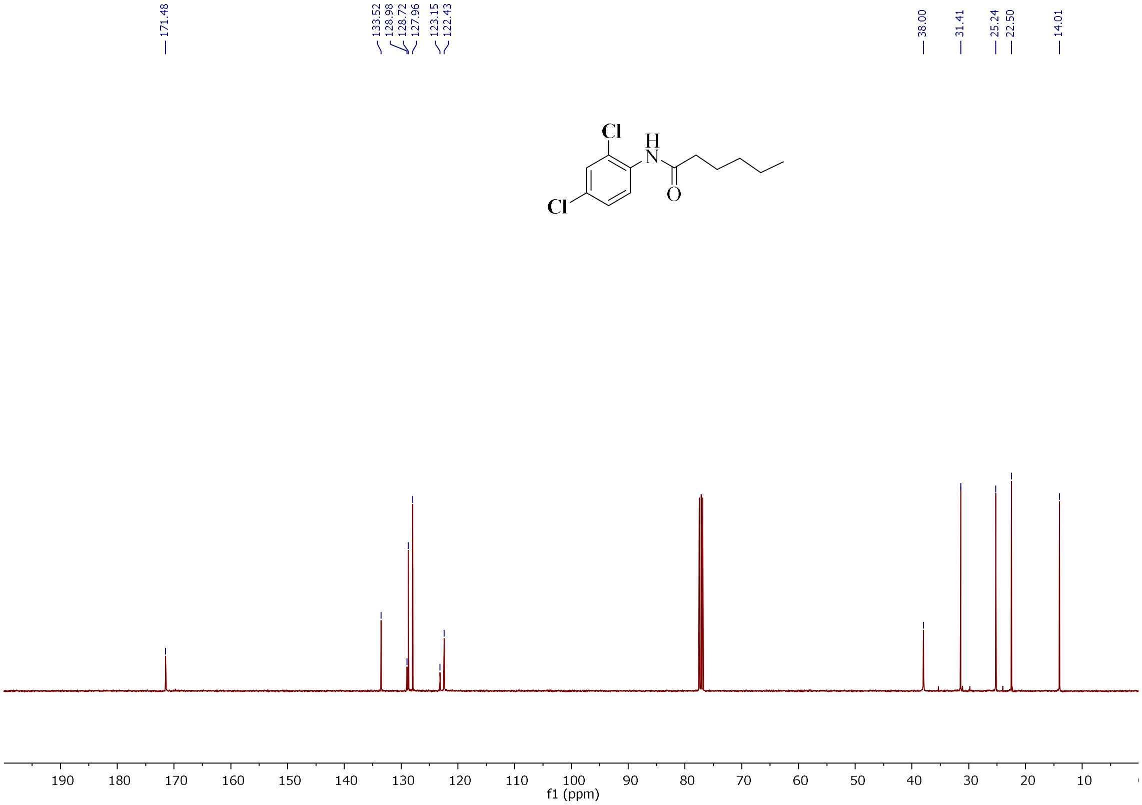

***N*-(2,4-dichlorophenyl)isobutyramide: (109)**

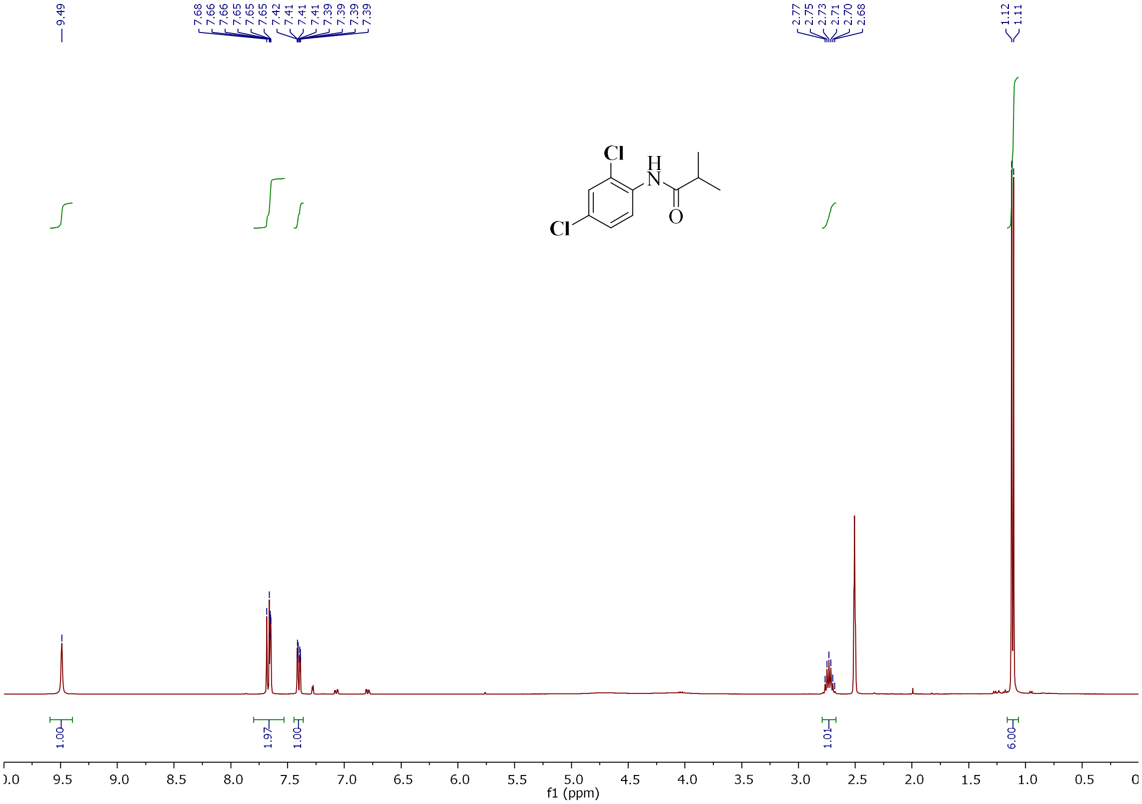

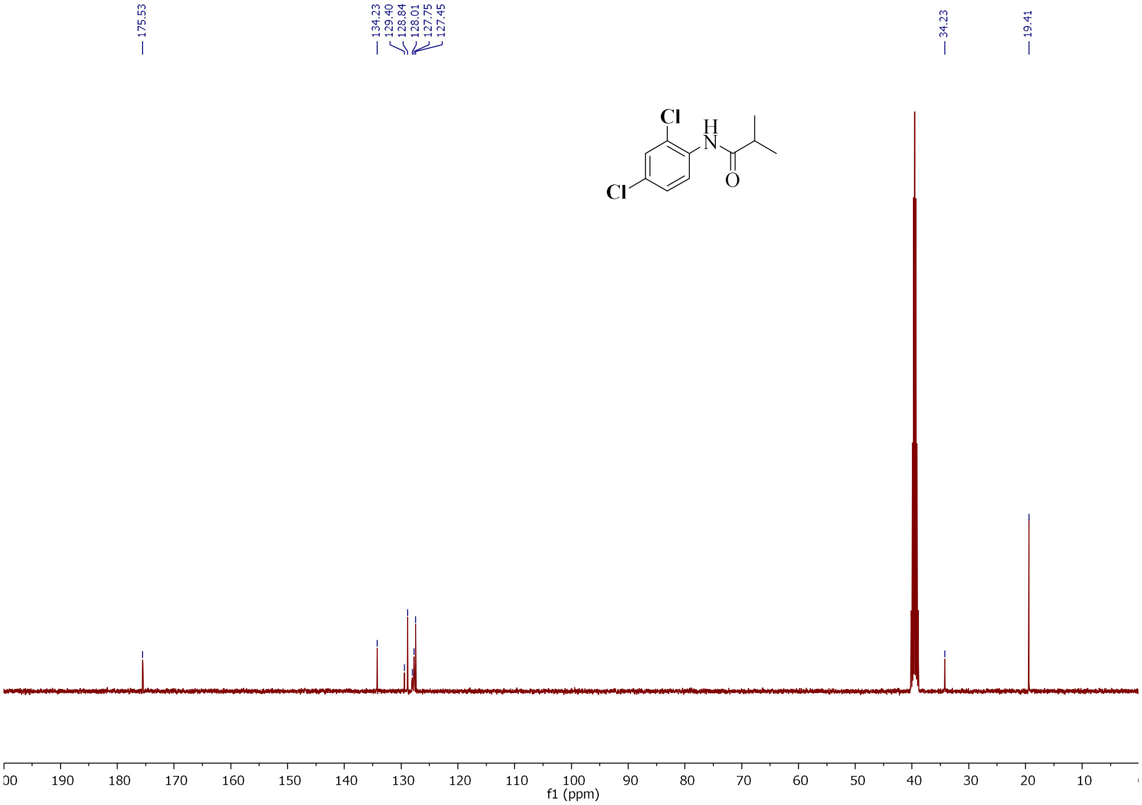

***N*-(2,4-dichlorophenyl)pivalamide: (110)**

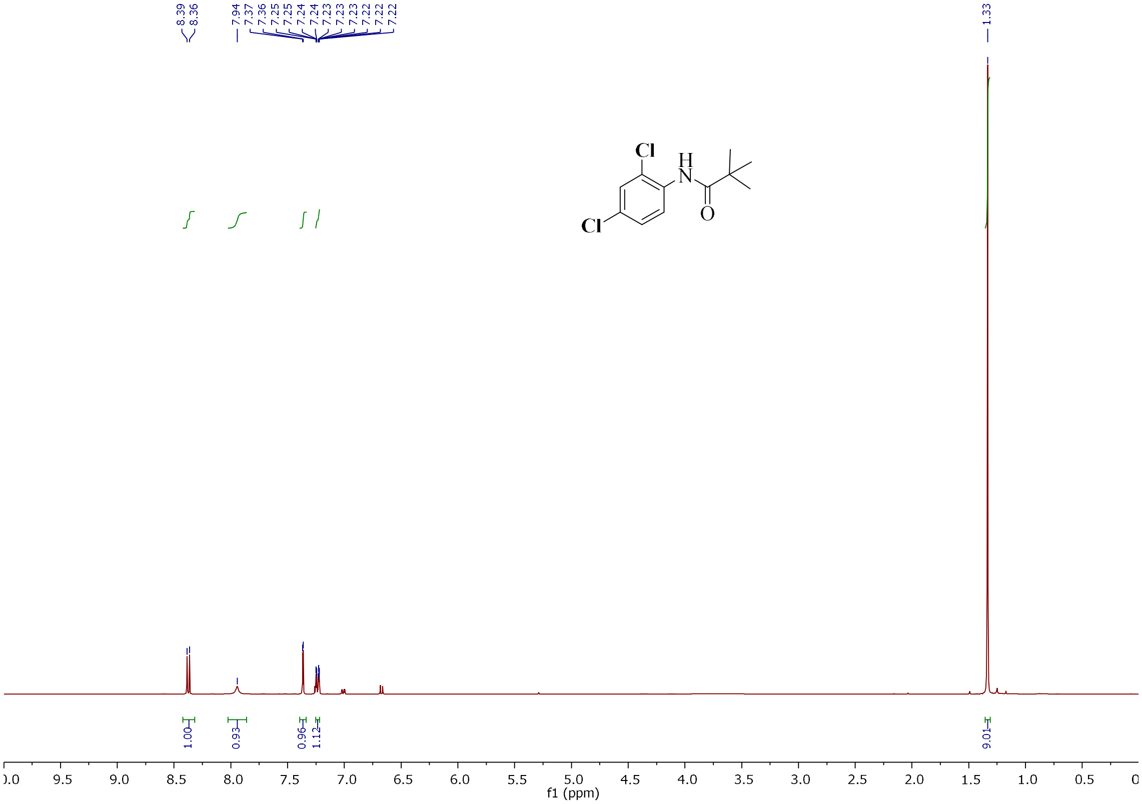

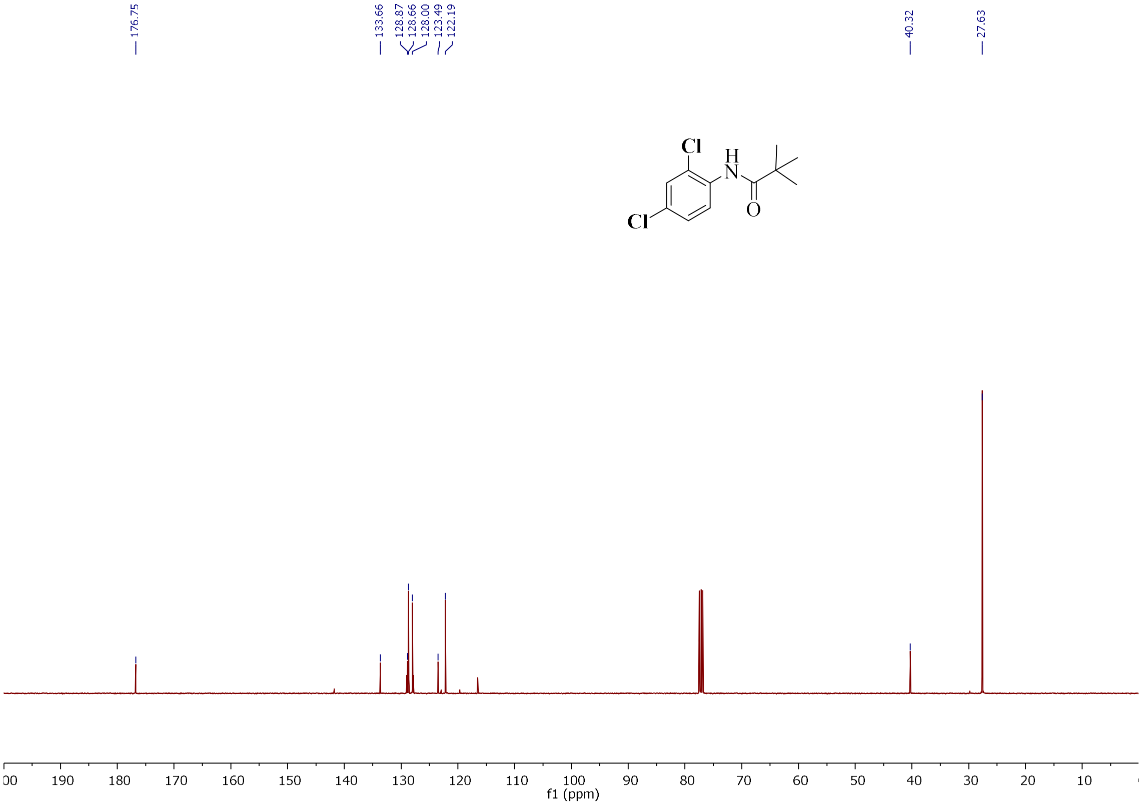

Ethyl (2,4-dichlorophenyl)carbamate: (111)

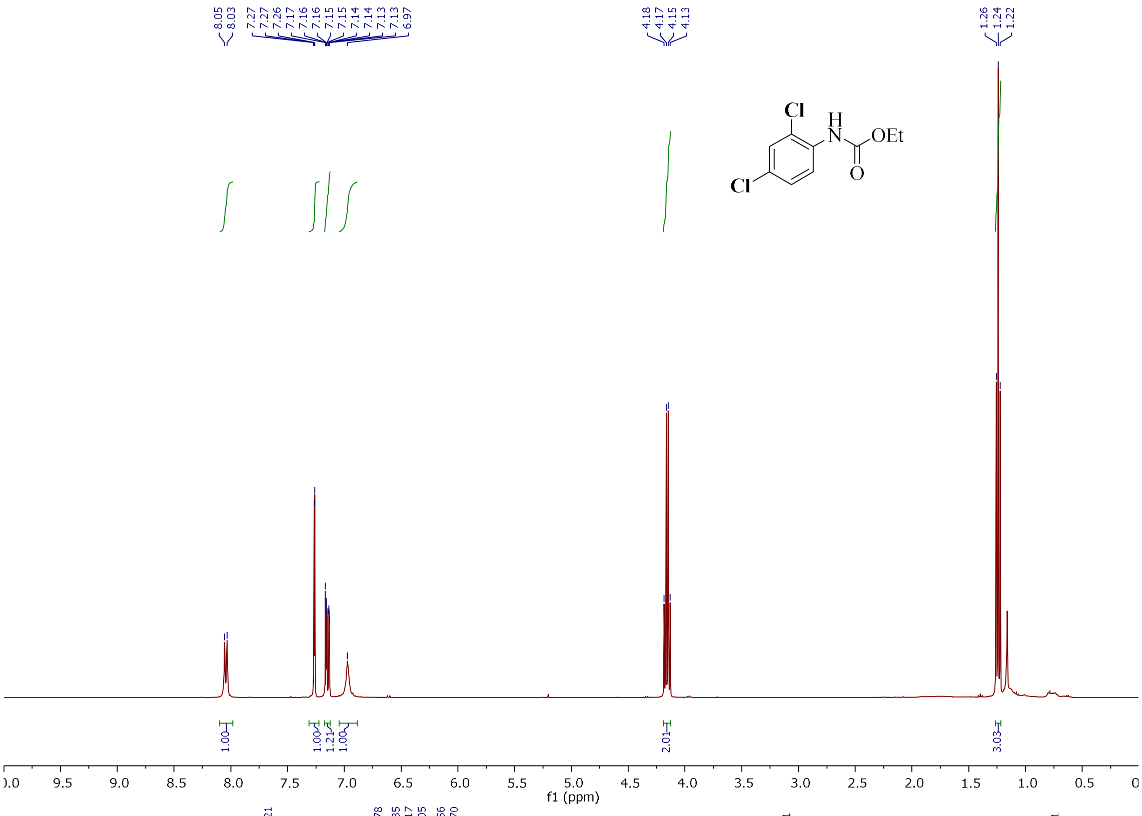

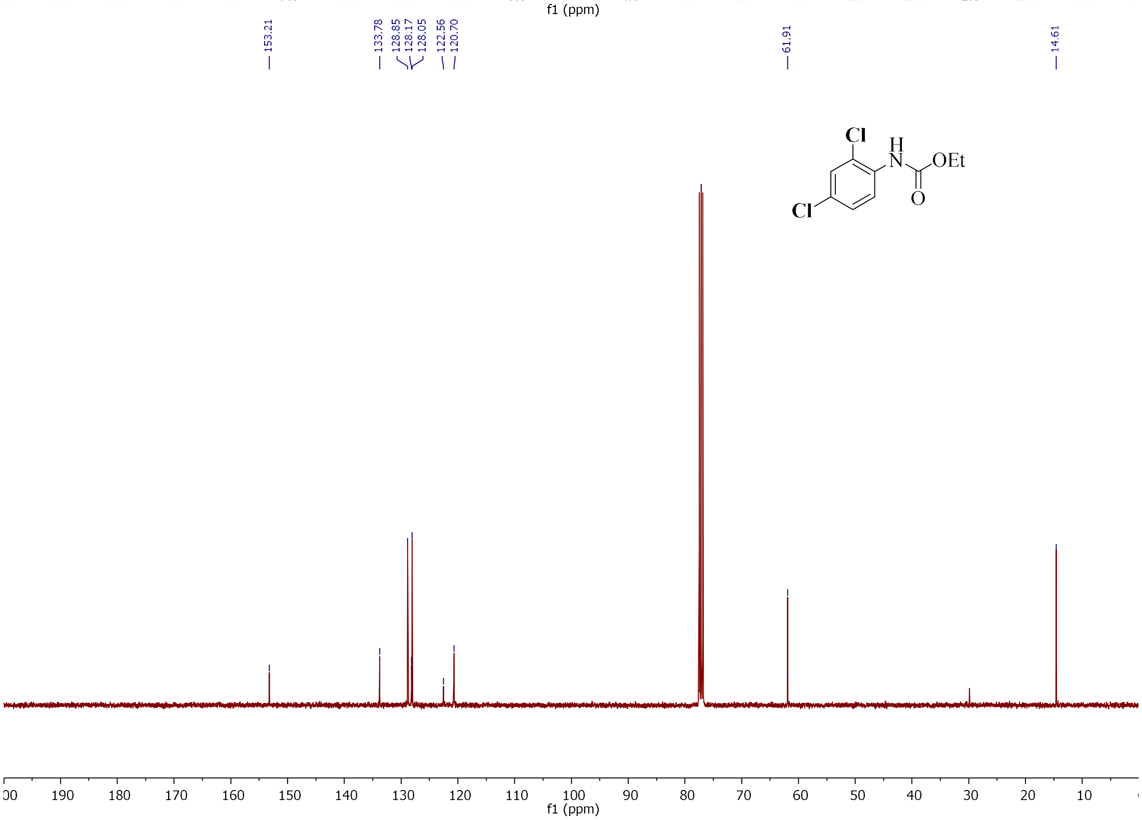

N-(2,4-dibromo-3,5-dimethoxyphenyl)propionamide: (112)

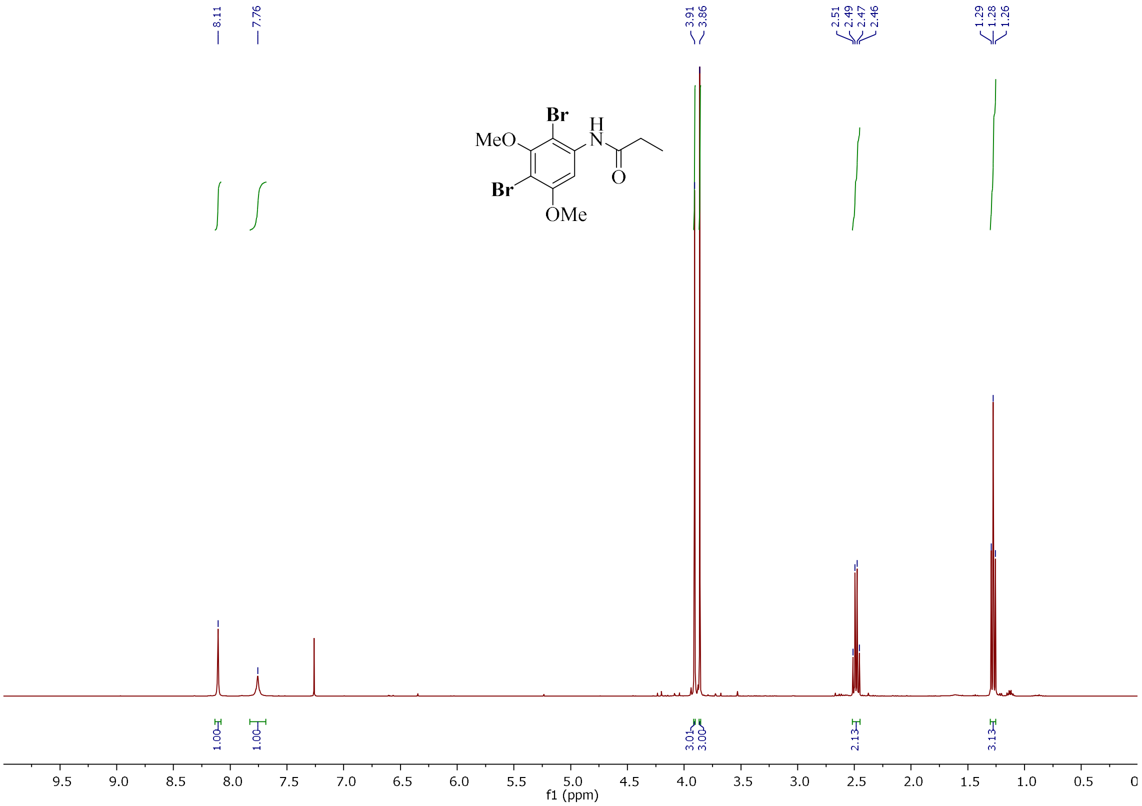

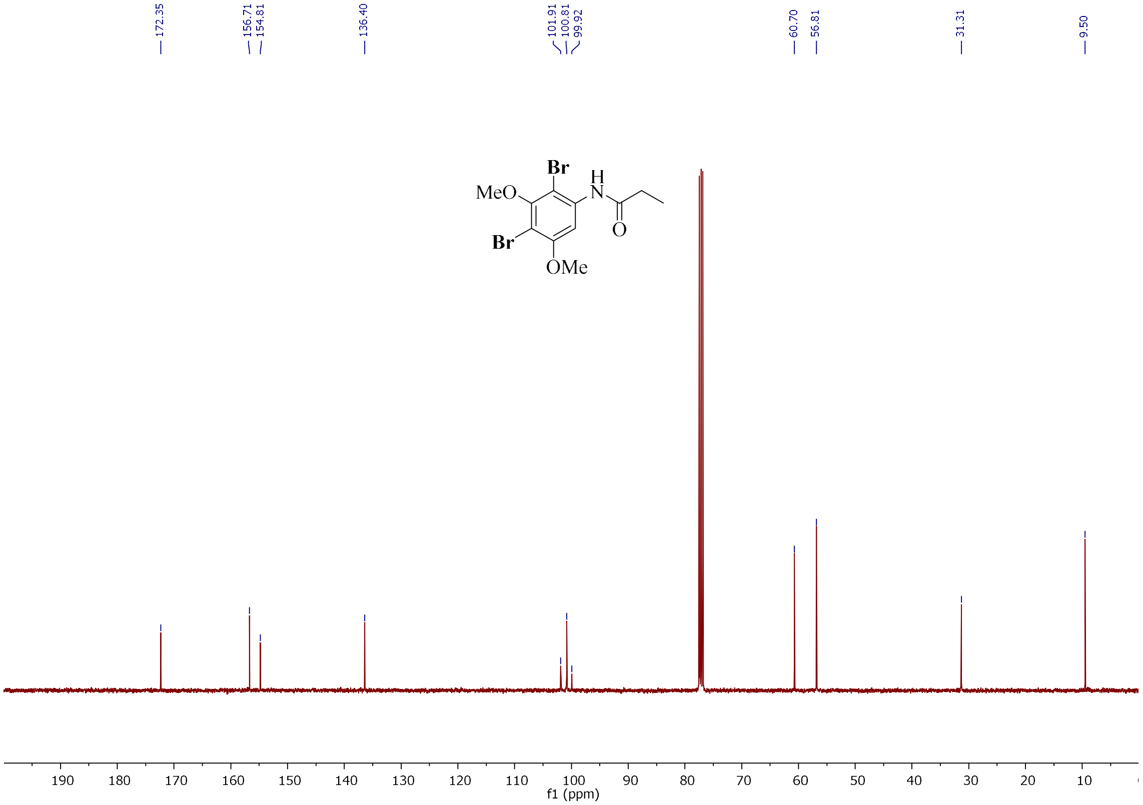

***N*-(2,4-dibromo-3,5-dimethylphenyl)propionamide: (113)**

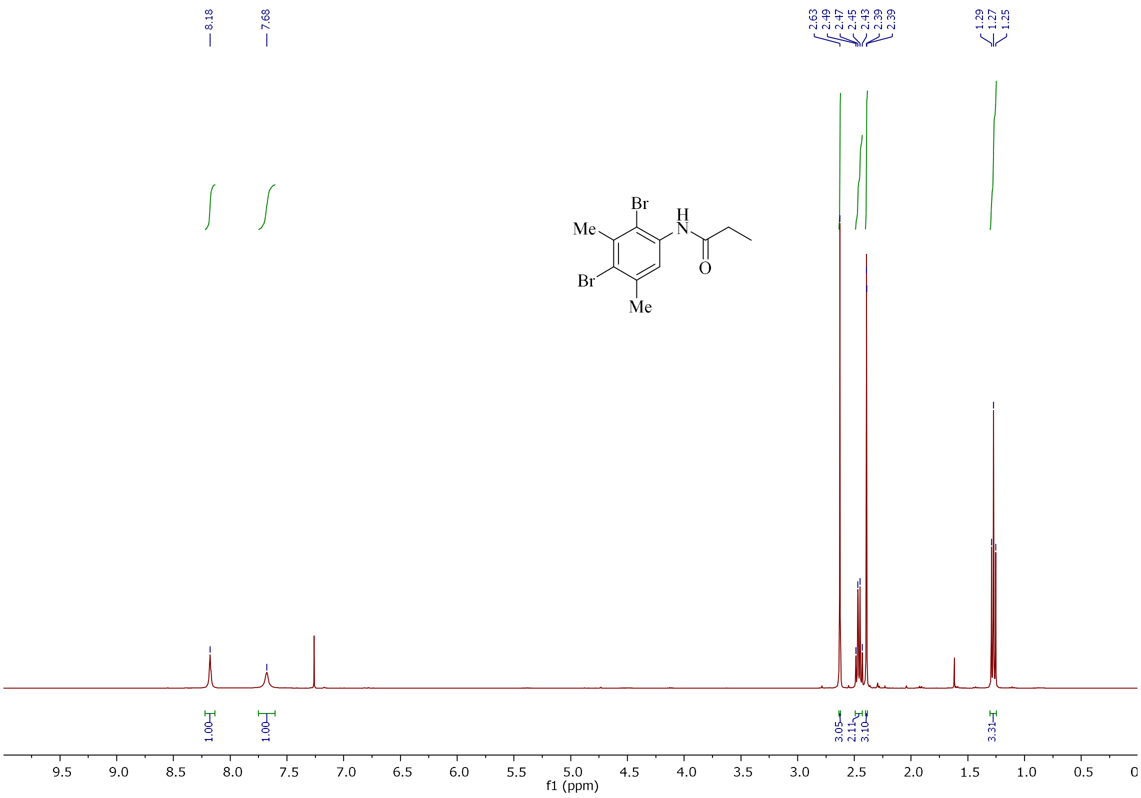

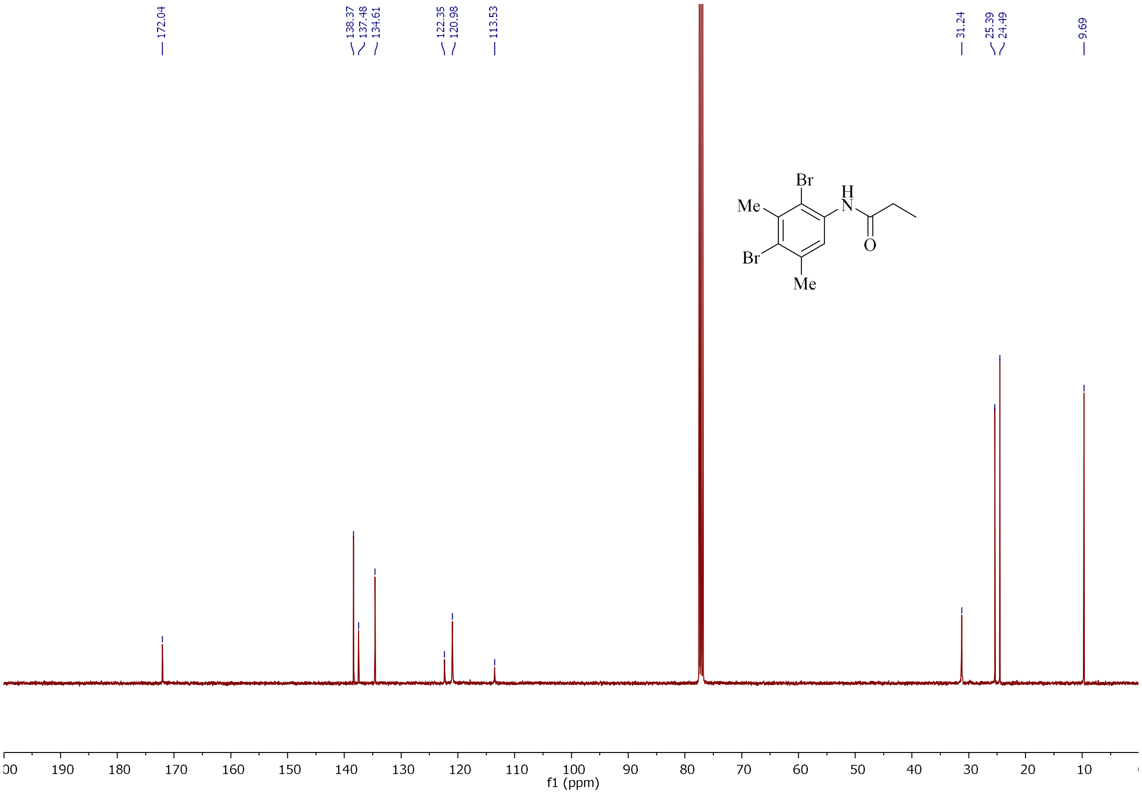

N-(2,4-dibromo-5-methoxyphenyl)propionamide: (114)

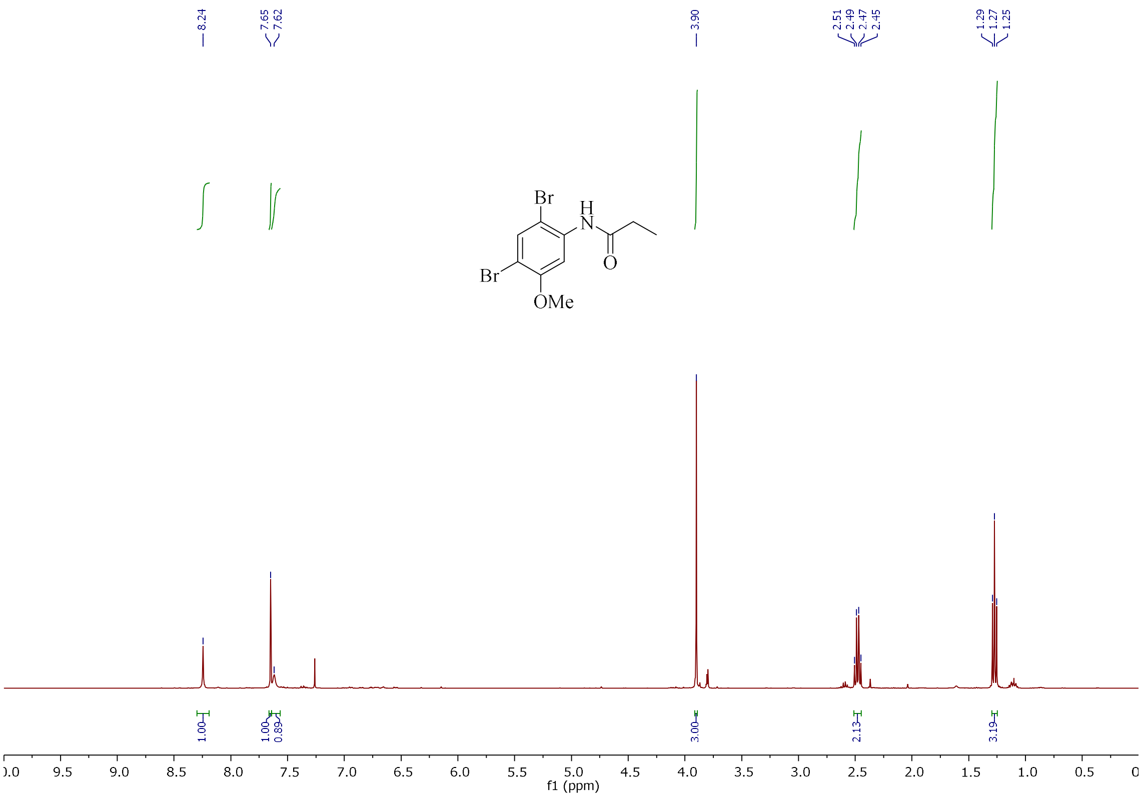

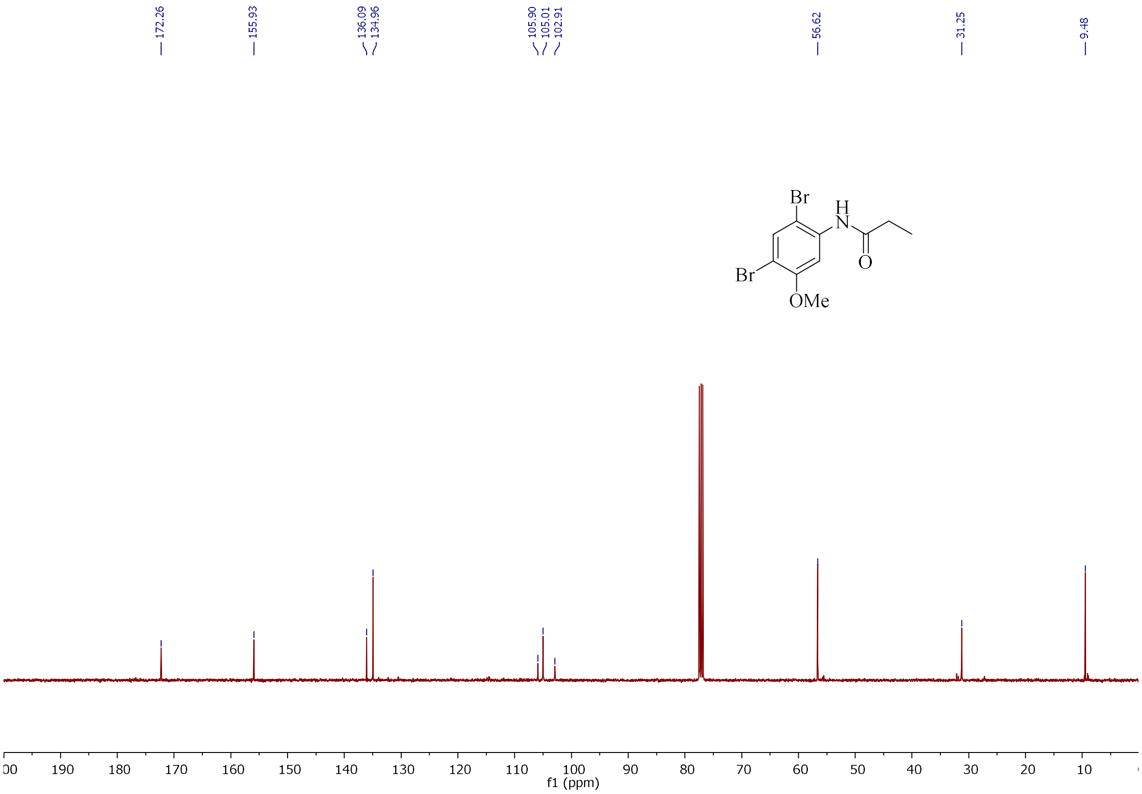

N-(2,4-dibromo-5-methoxyphenyl)benzamide: (115)

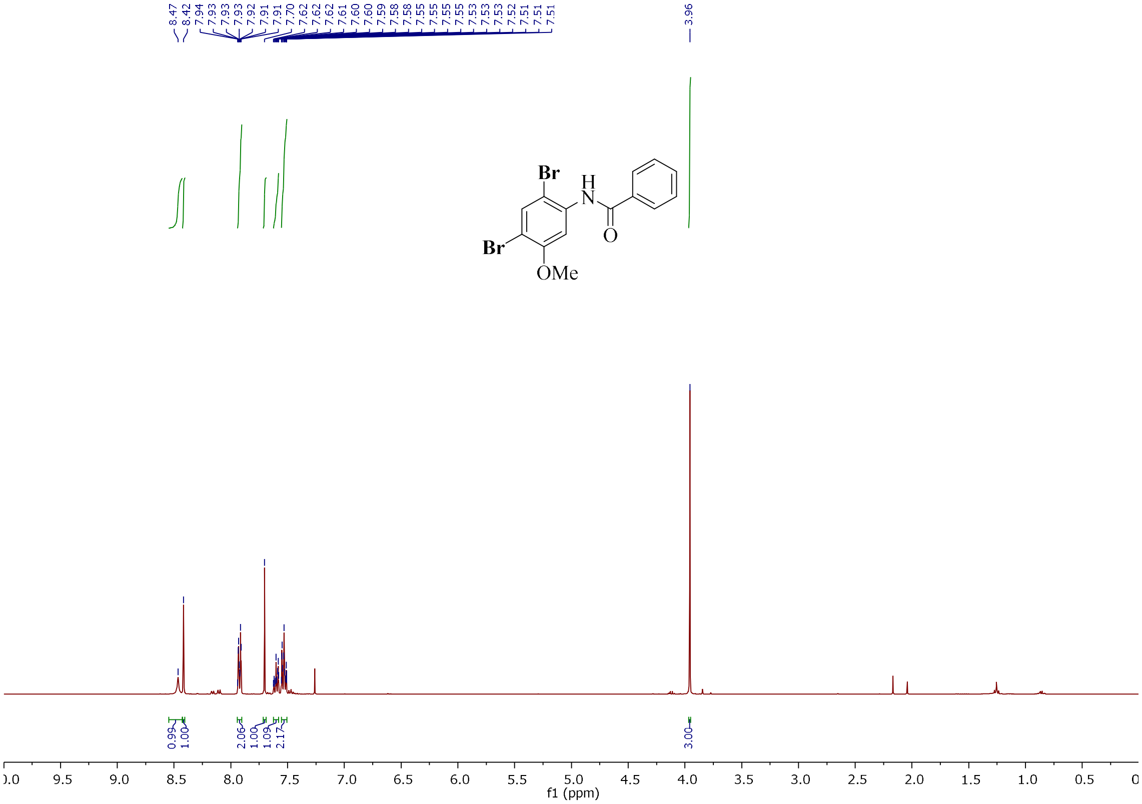

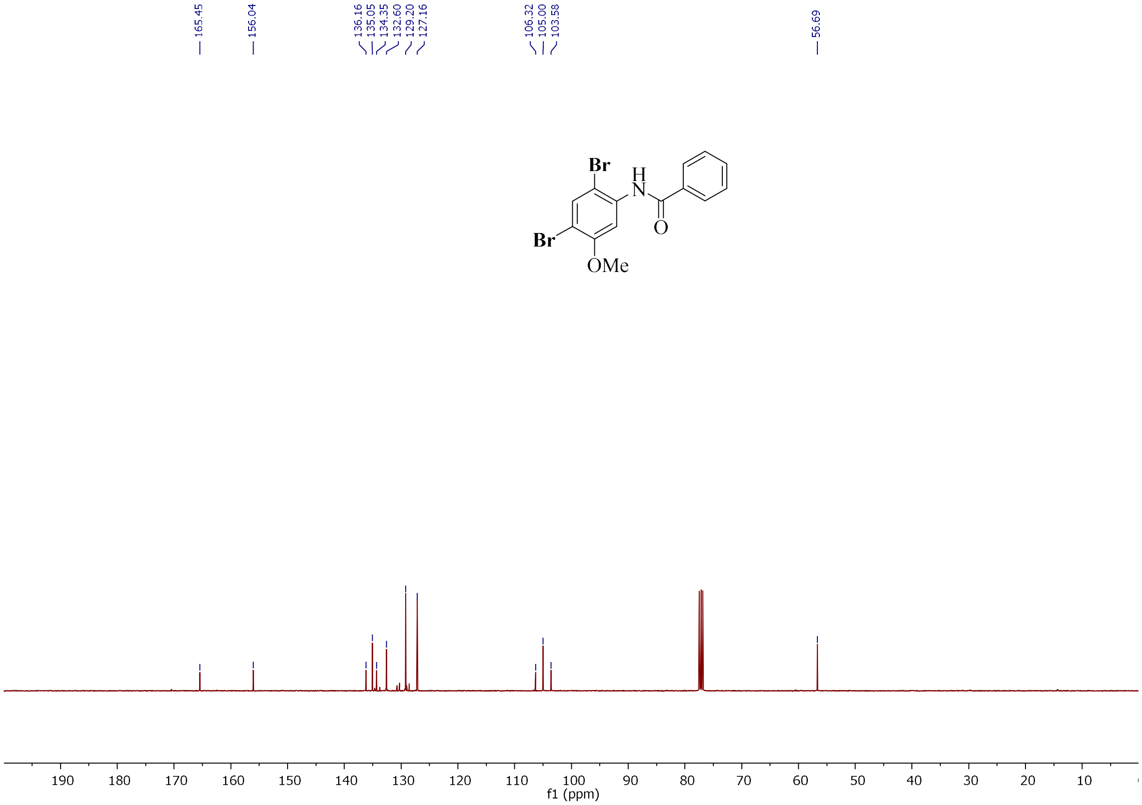

***N*-(2,4-dibromo-3,5-dimethoxyphenyl)benzamide: (116)**

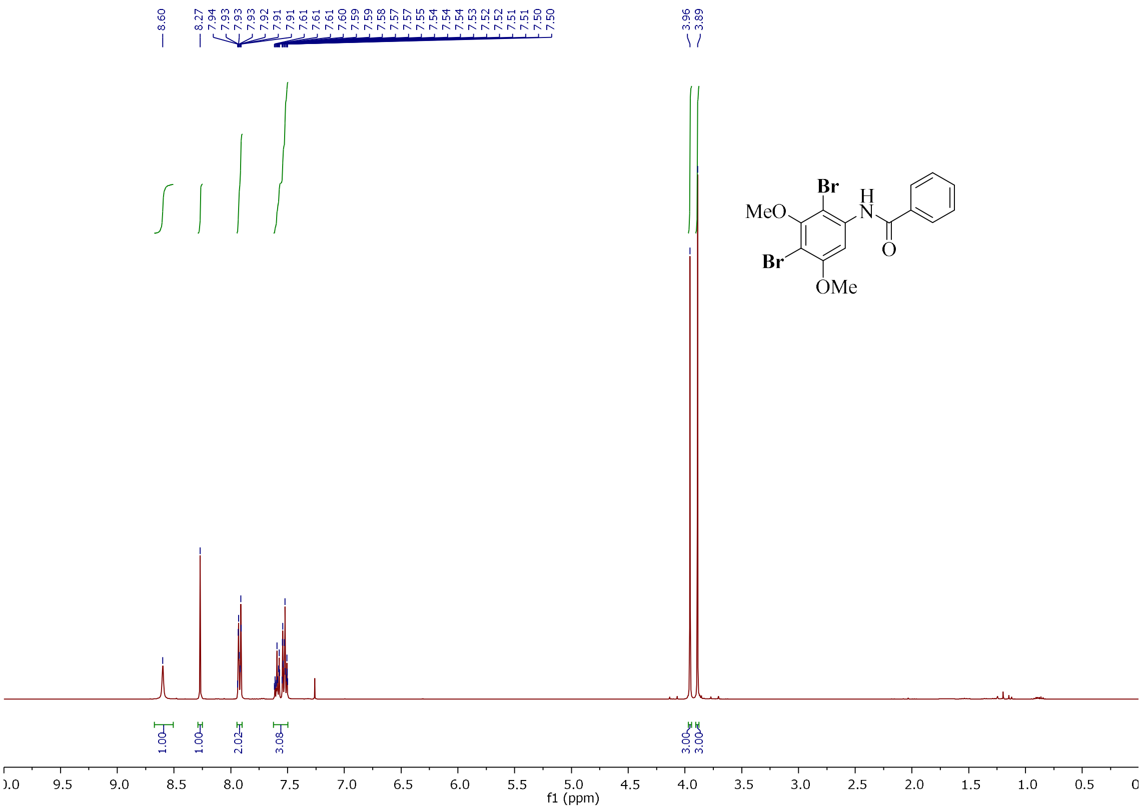

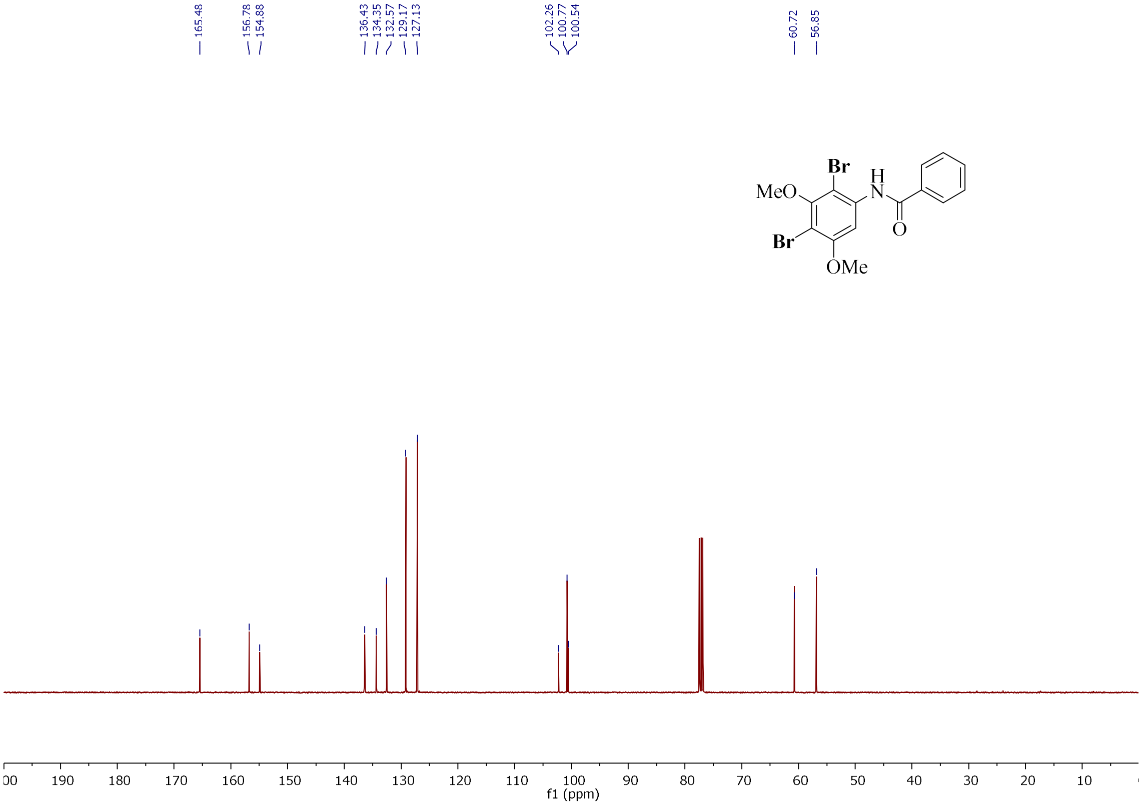

***N*-(5-chloro-2-oxo-2*H*-chromen-6-yl)cyclohexanecarboxamide: (117)**

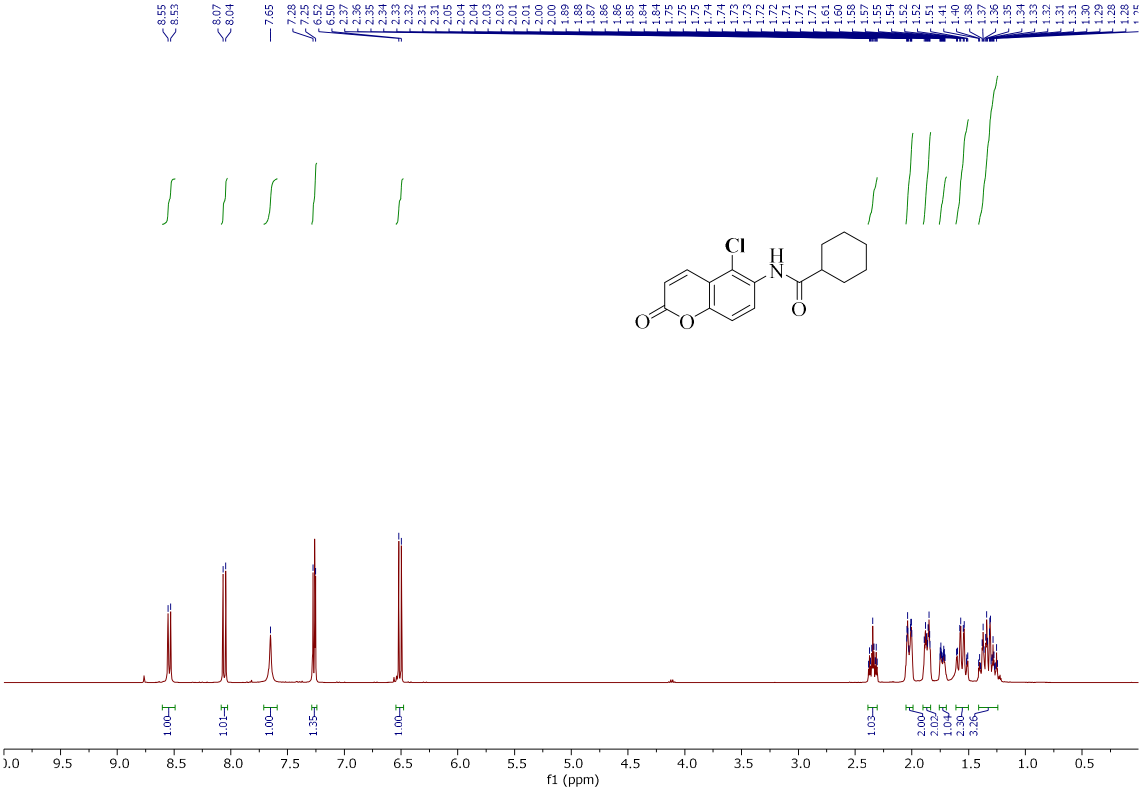

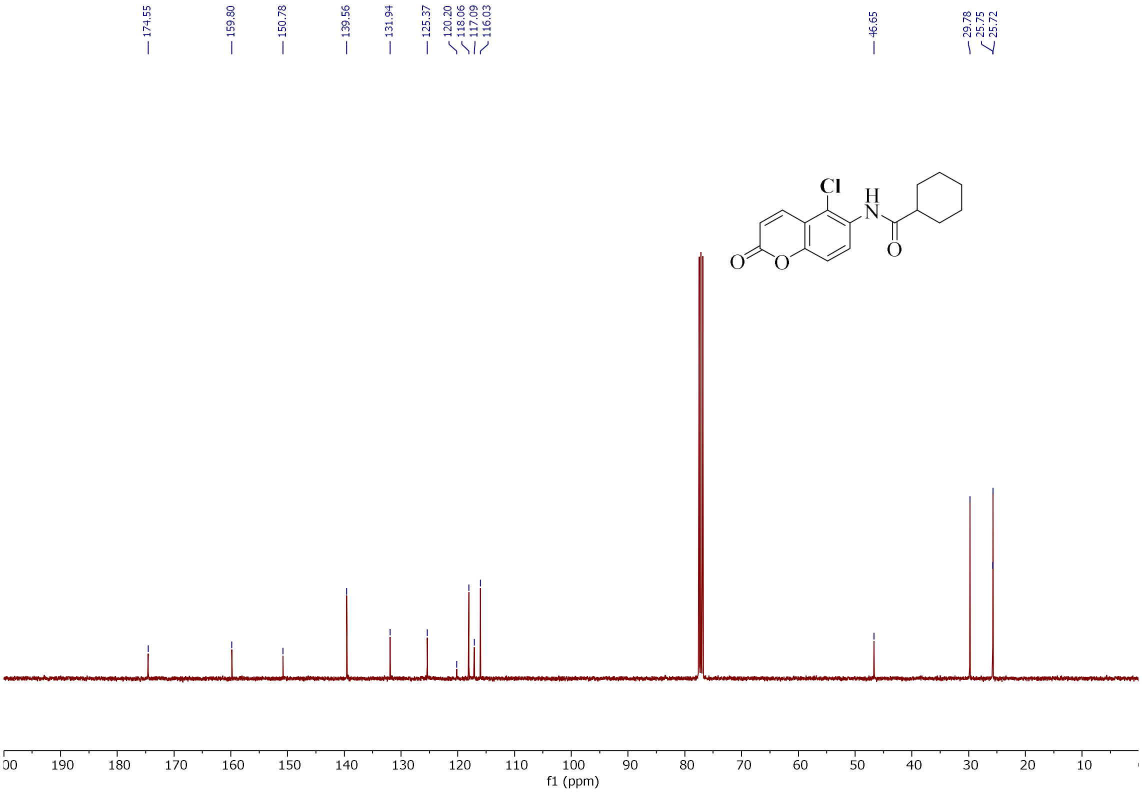

_N_-(8-chloro-4-methyl-2-oxo-2_H_-chromen-7-yl)cyclohexanecarboxamide: (118)

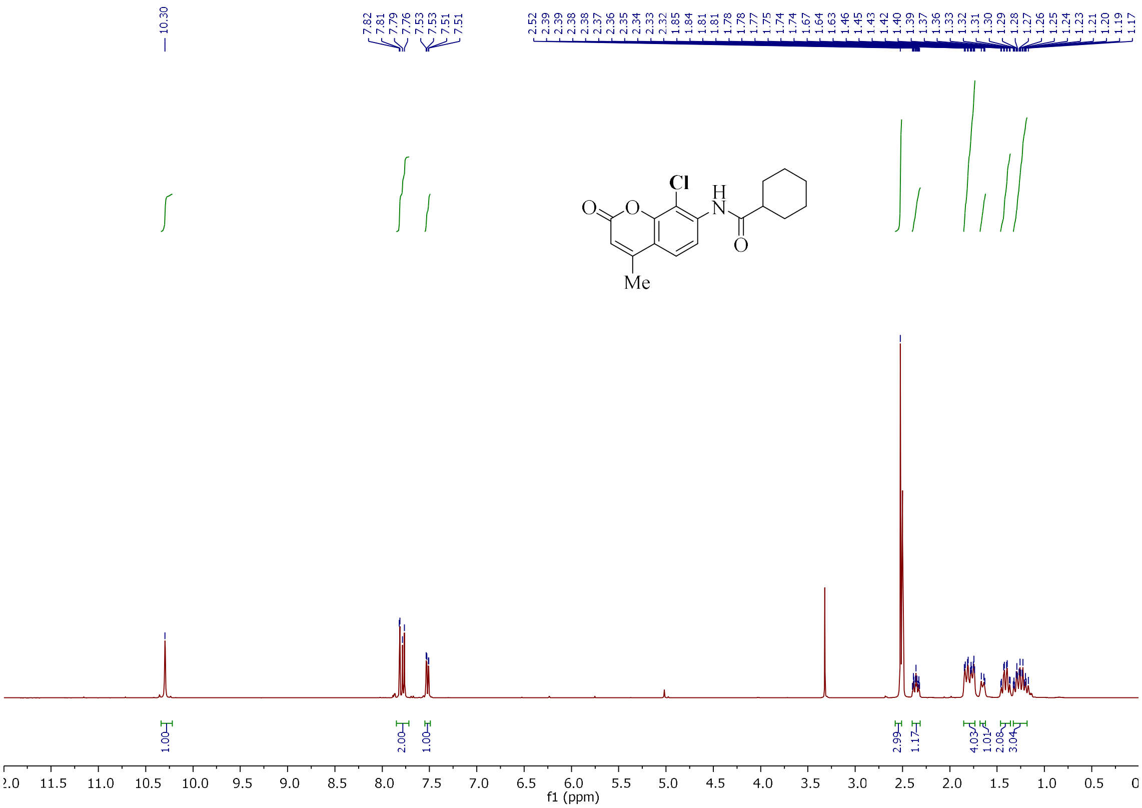

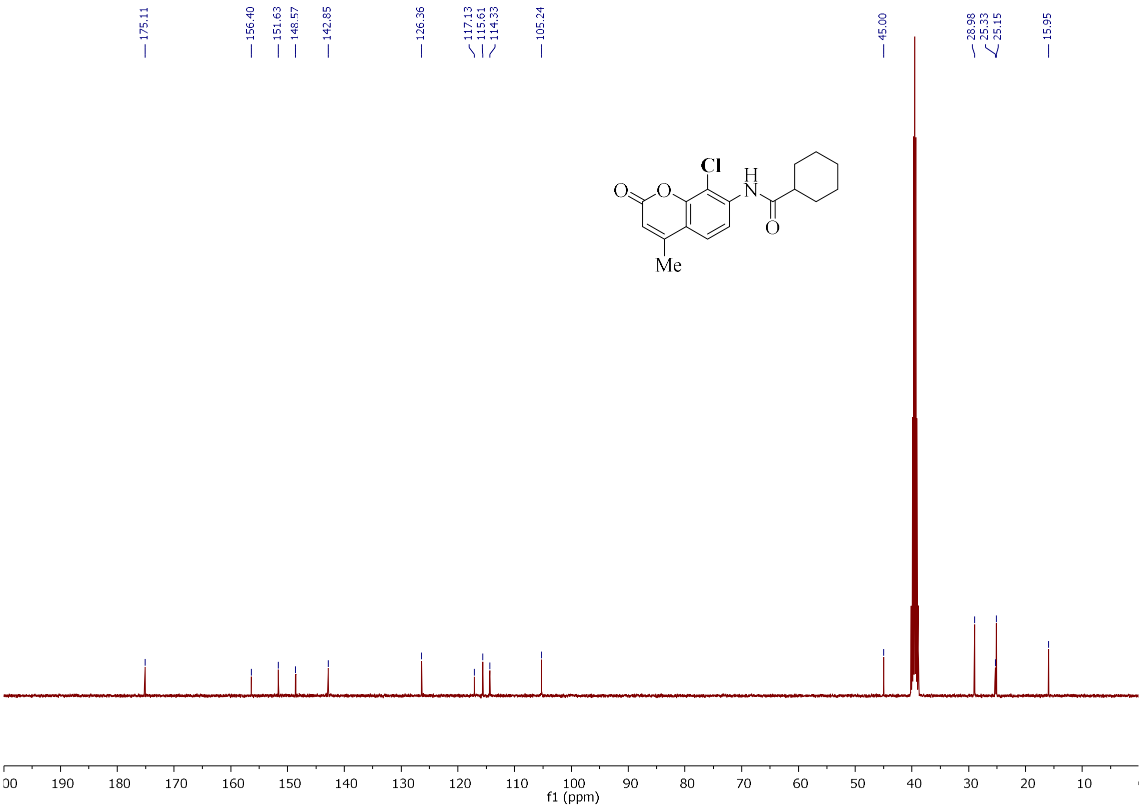

***N*-(4-chloroquinolin-3-yl)cyclohexanecarboxamide: (119)**

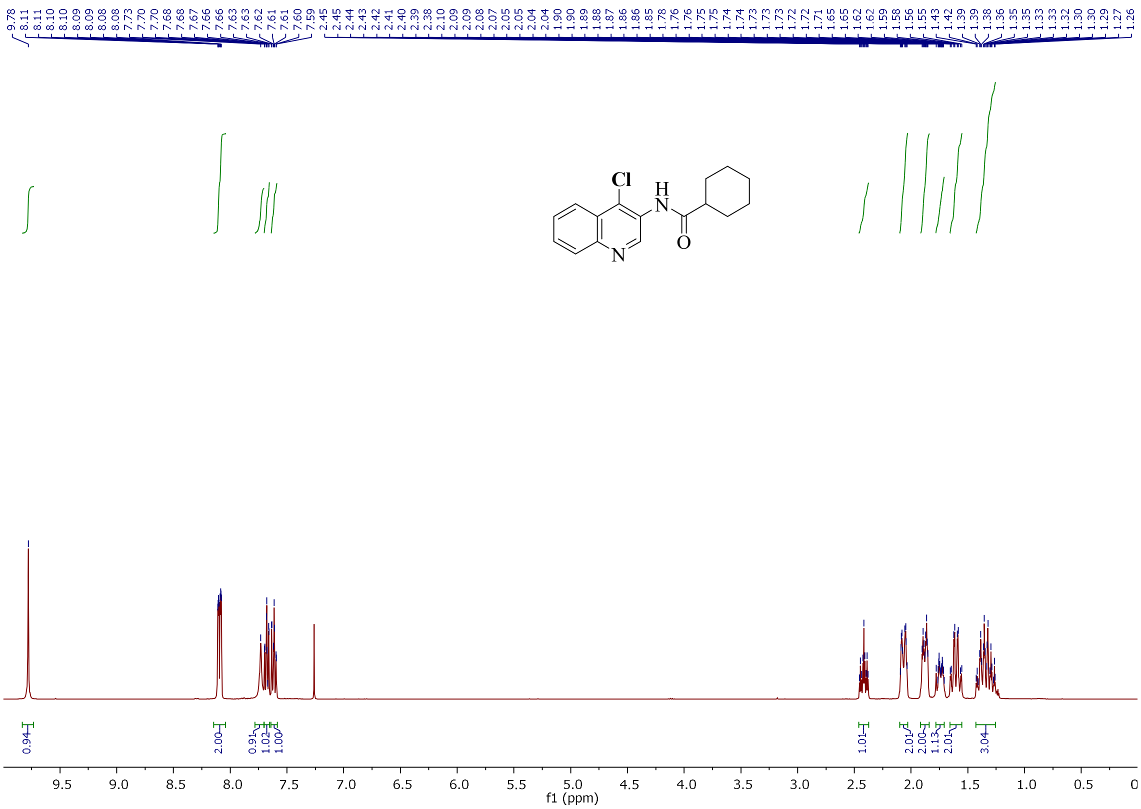

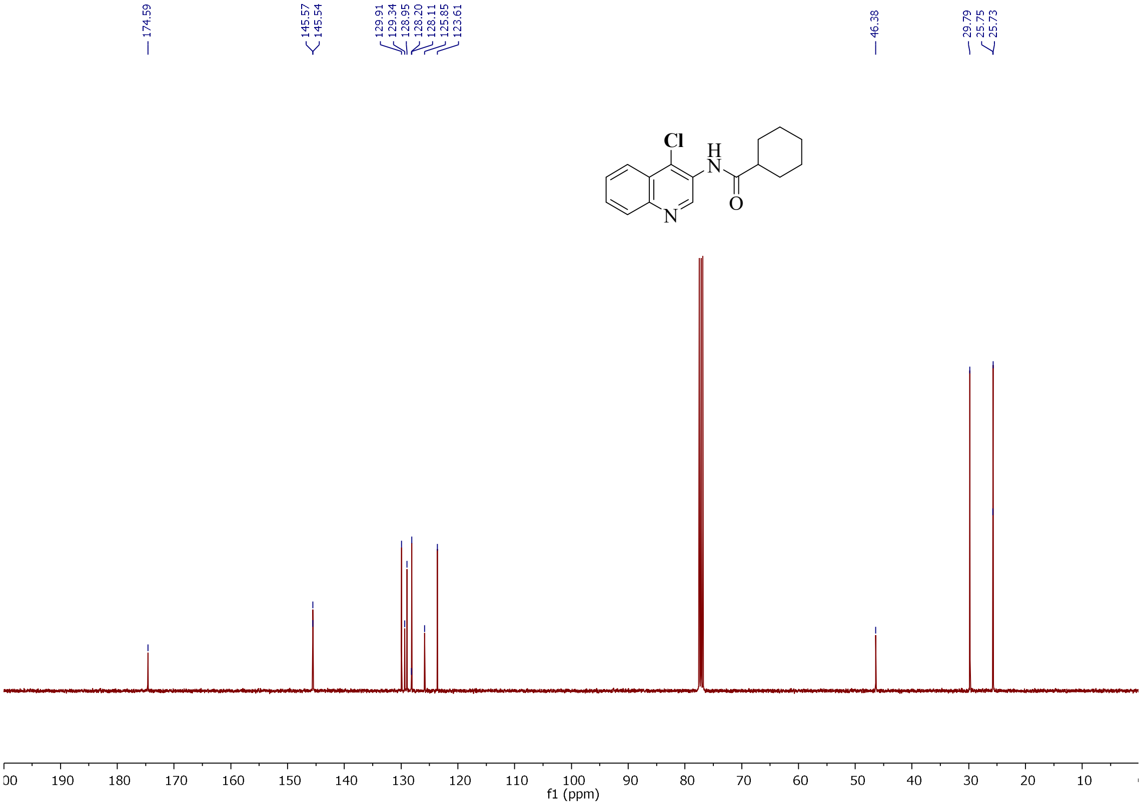

***N*-(5-chloroquinolin-6-yl)cyclohexanecarboxamide: (120)**

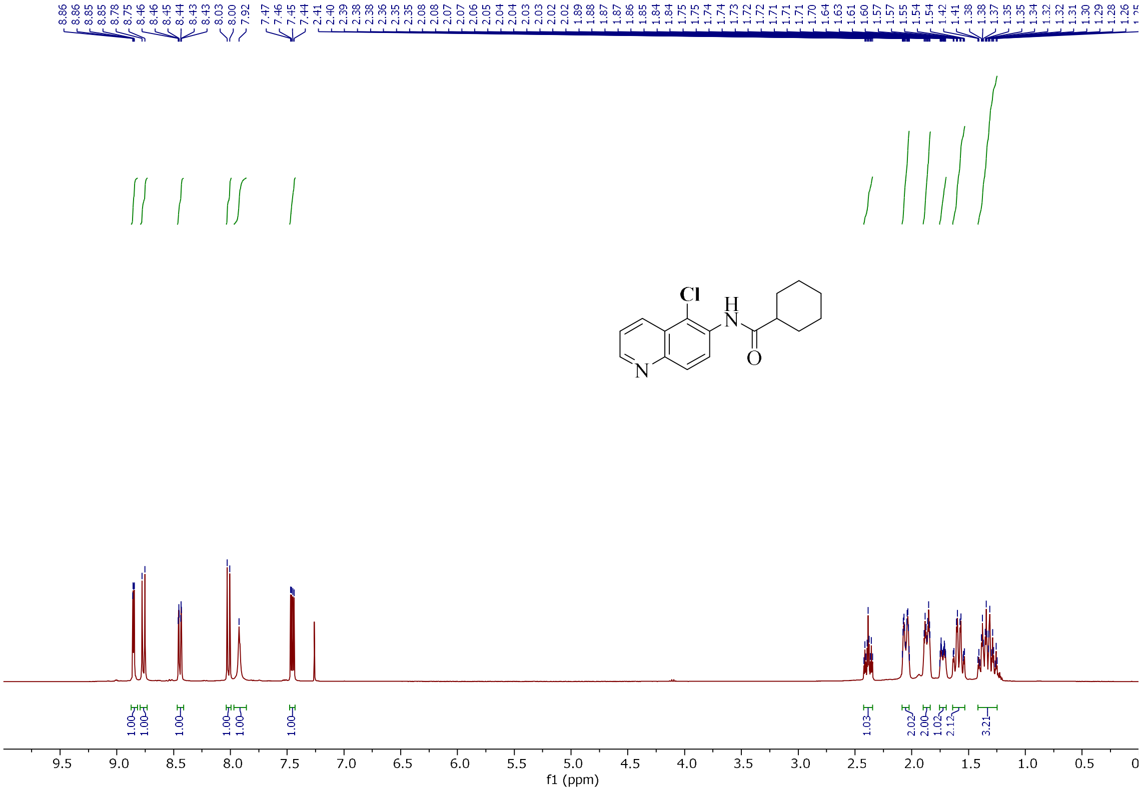

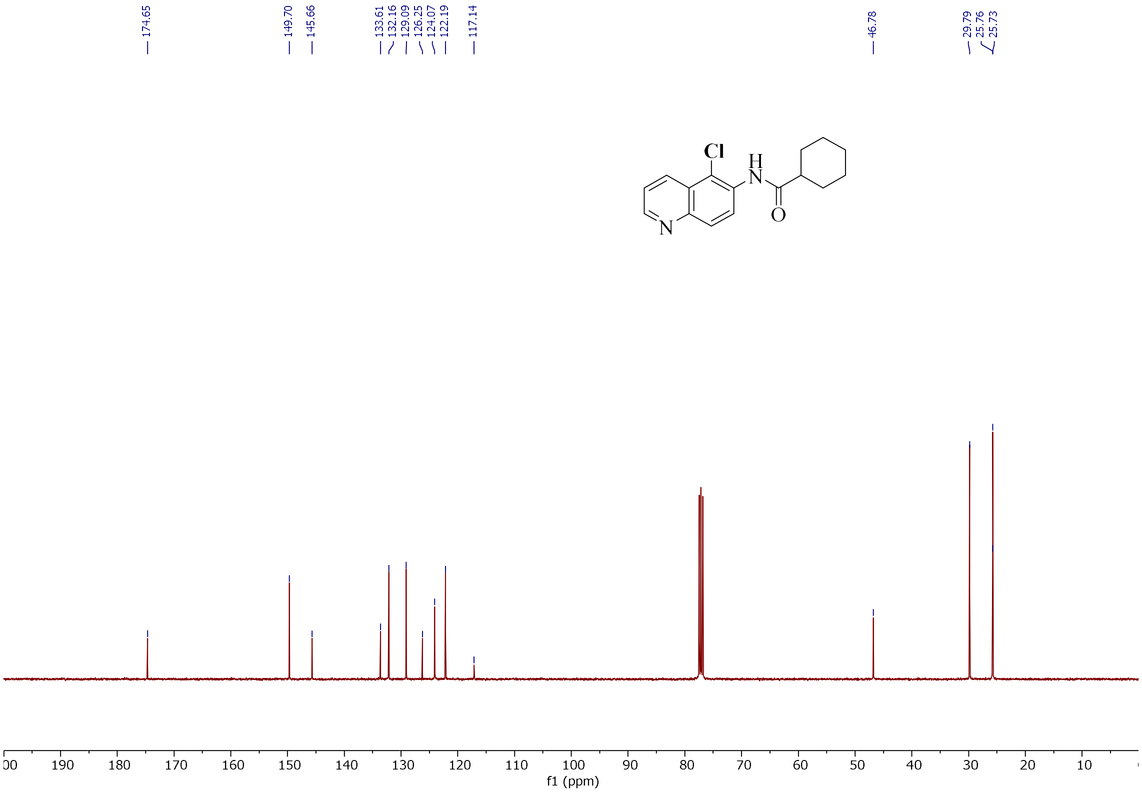

***N*-(6-chloroisoquinolin-7-yl)cyclohexanecarboxamide: (121)**

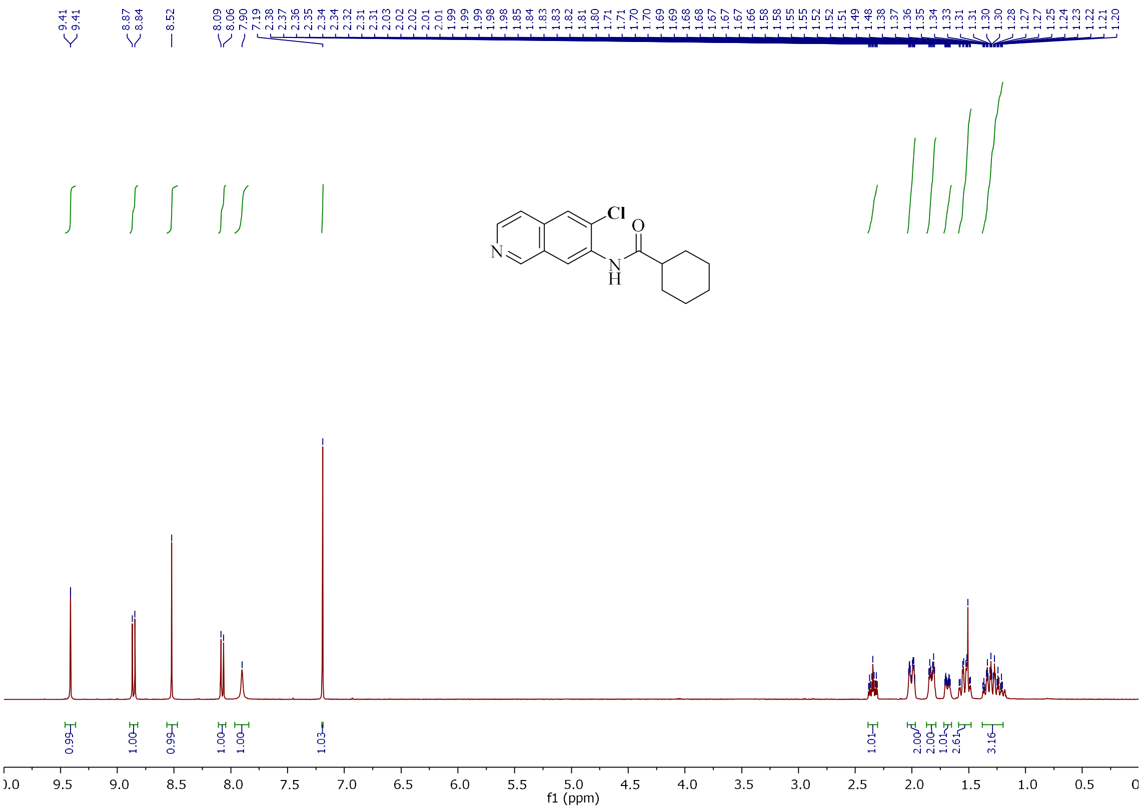

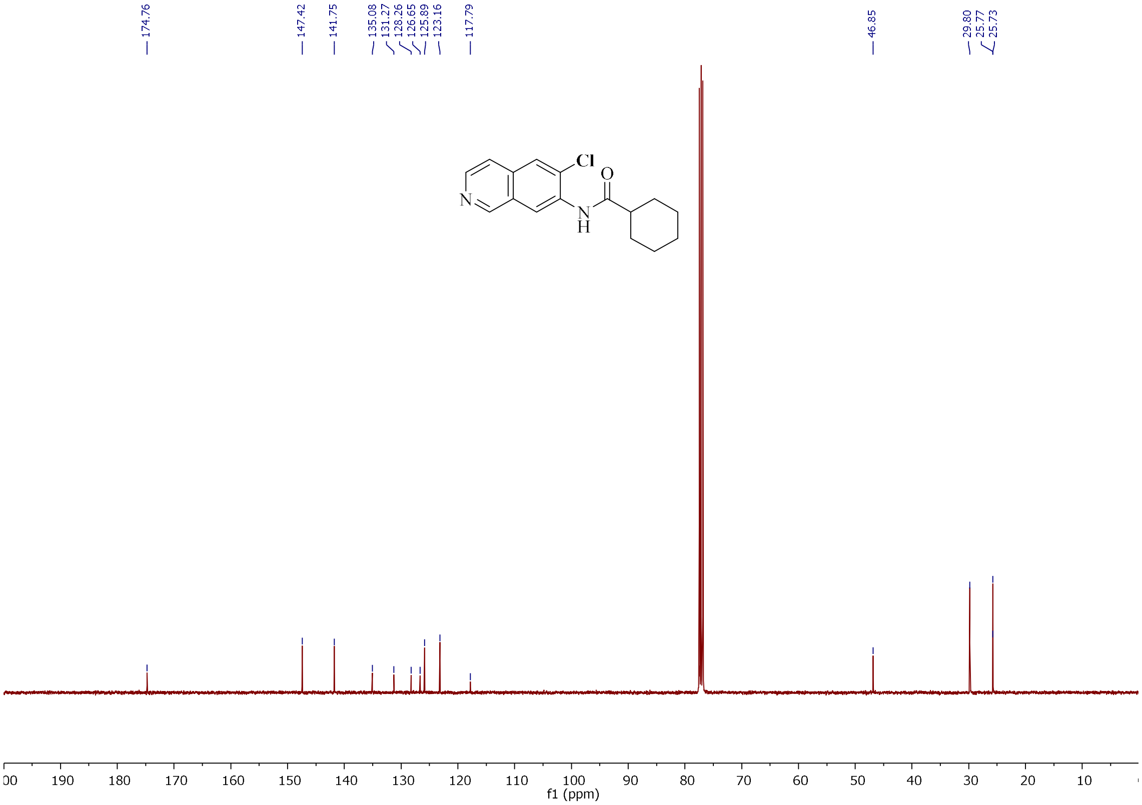

_N_-(7-chlorobenzo[d]thiazol-6-yl)cyclohexanecarboxamide: (122)

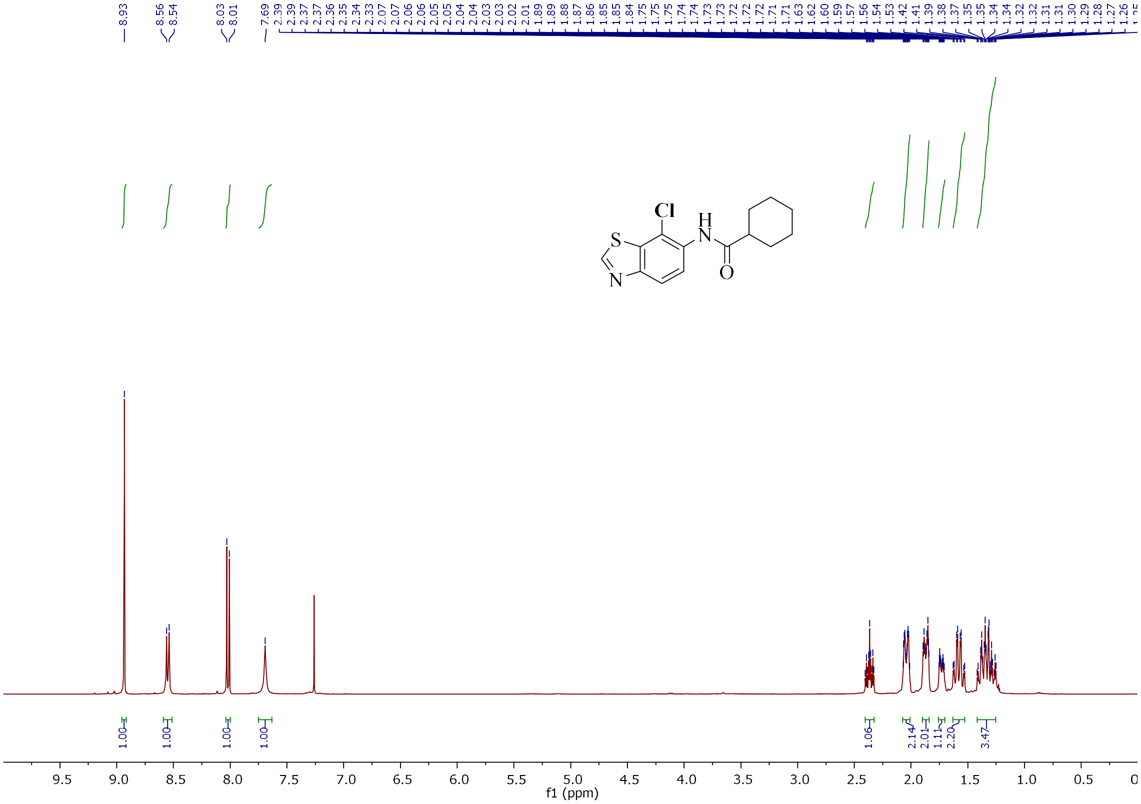

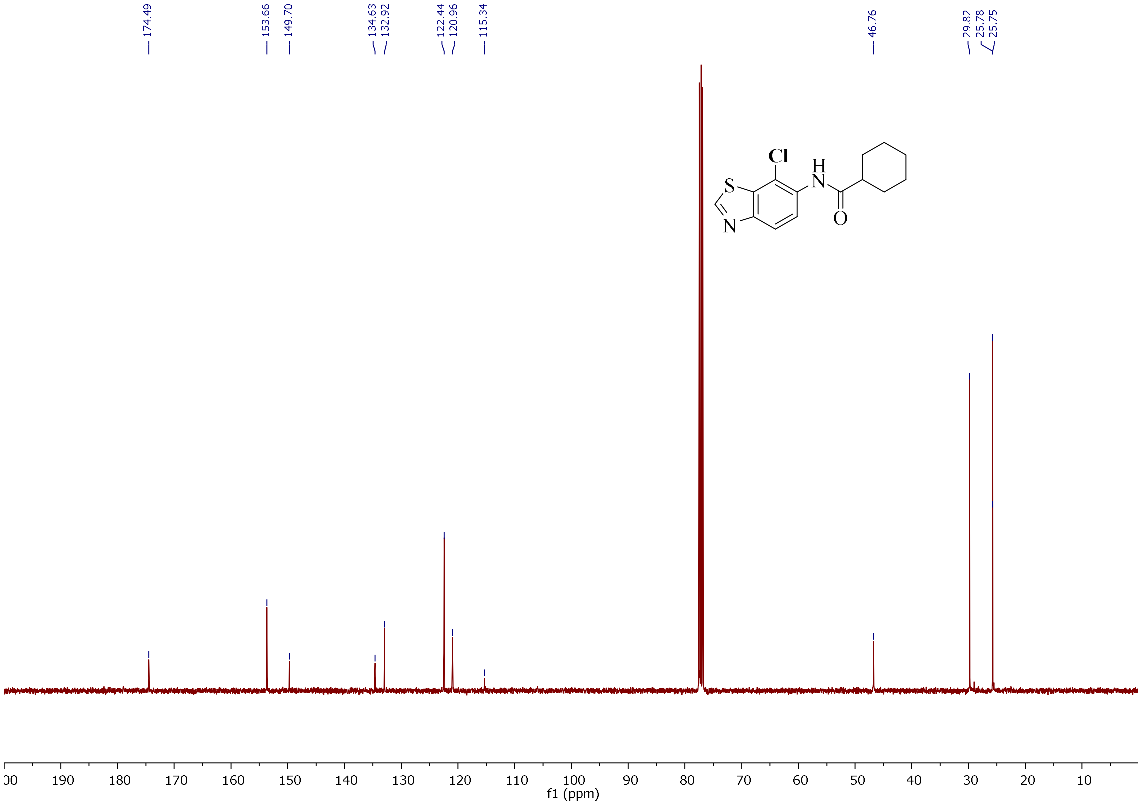

***N*-(3-(tert-butyl)-4-chloroisoxazol-5-yl)cyclohexanecarboxamide: (123)**

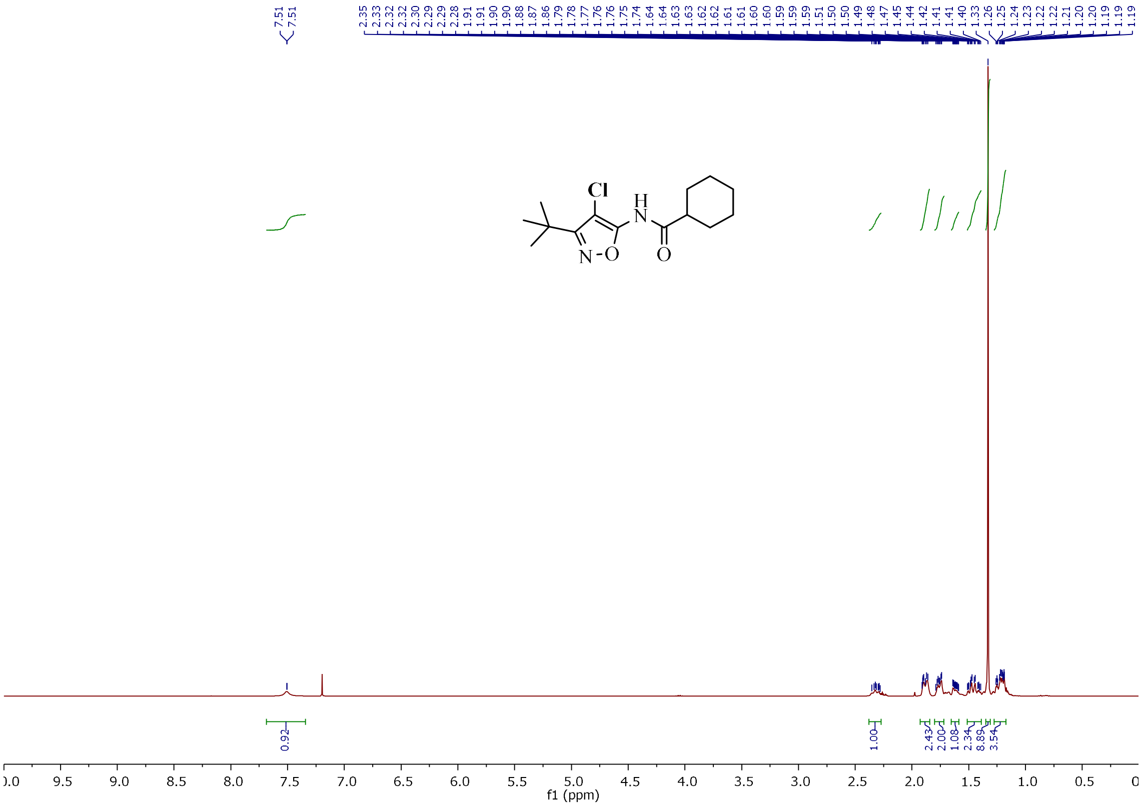

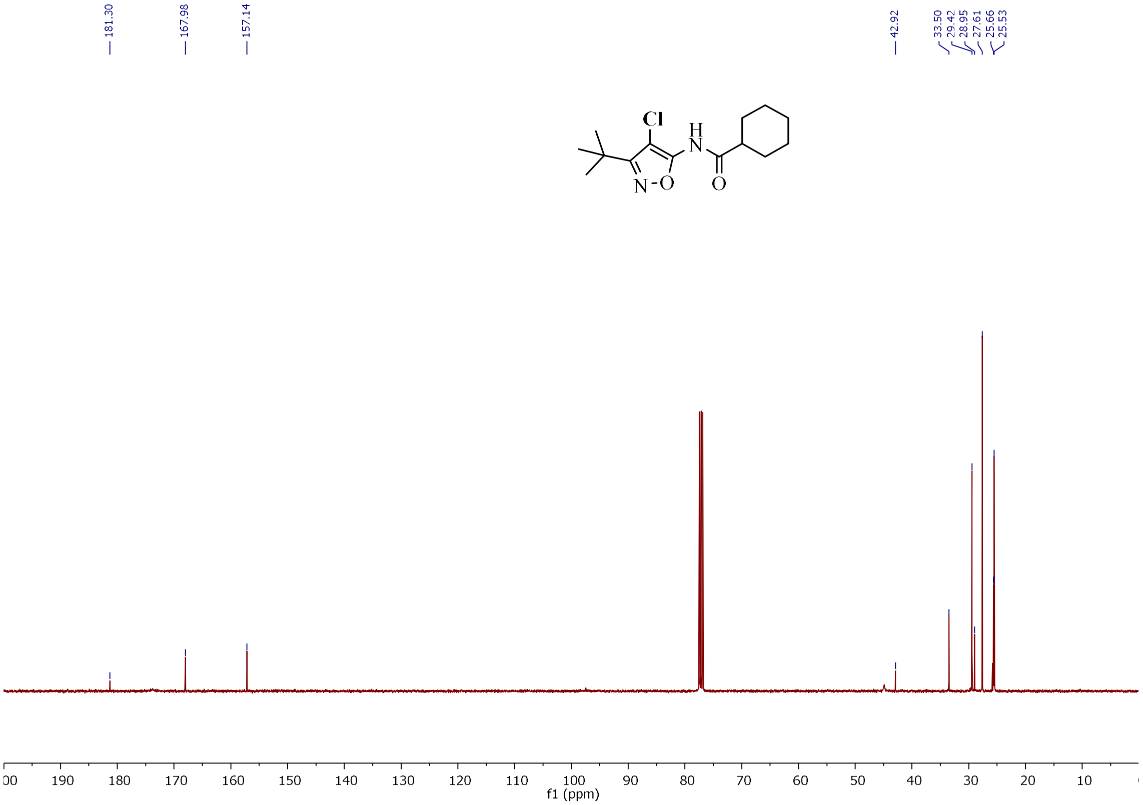

N-(4-chloro-1,3-diphenyl-1H-pyrazol-5-yl)cyclohexanecarboxamide: (124)

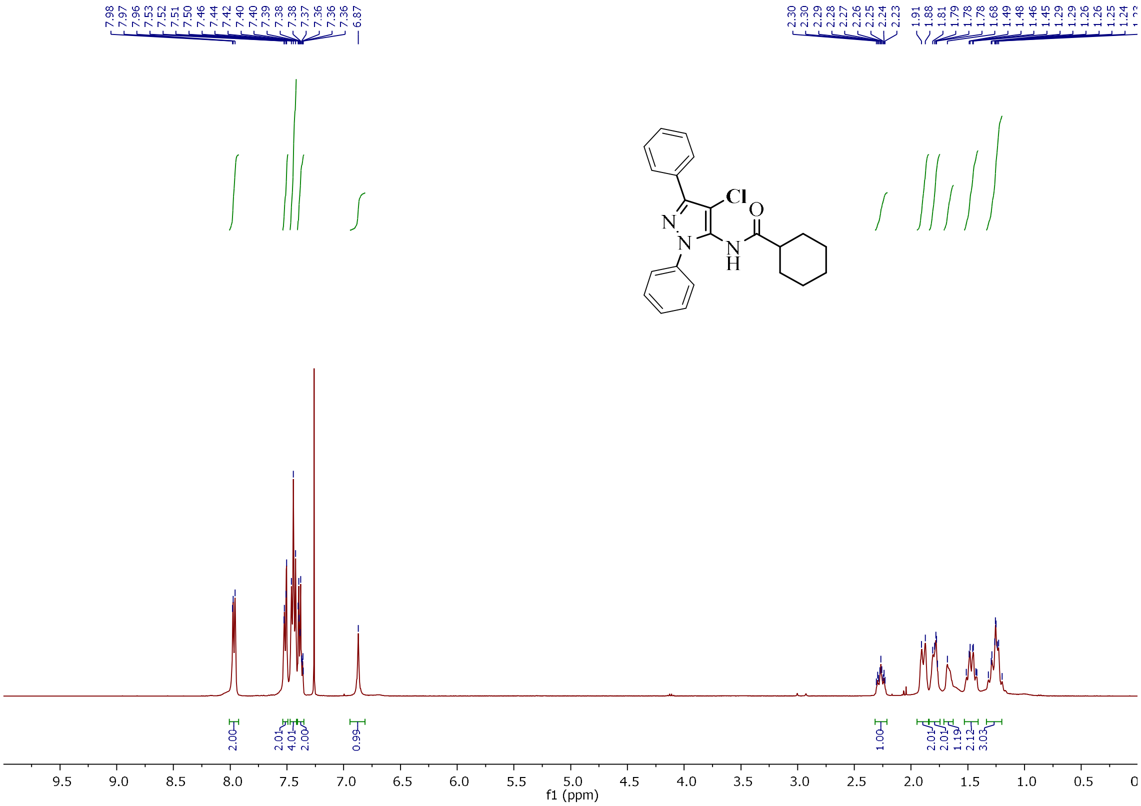

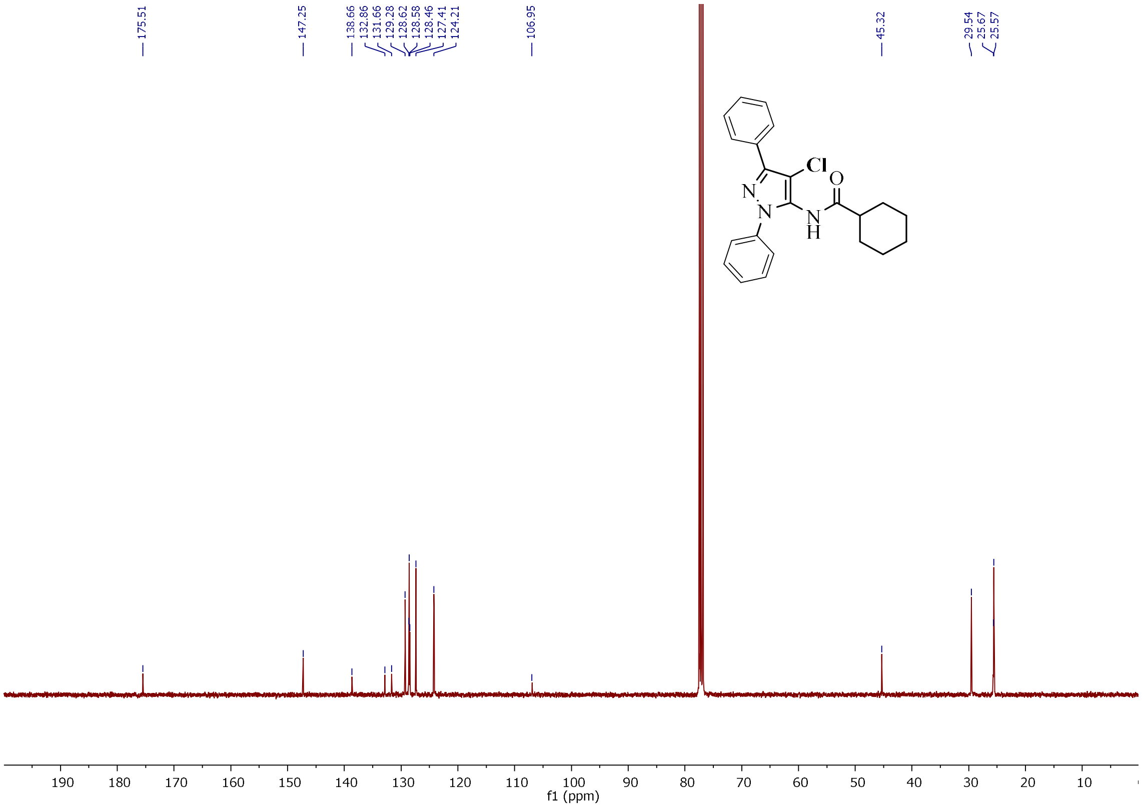

***N*-(7-chloro-1*H*-indazol-6-yl)cyclohexanecarboxamide: (125)**

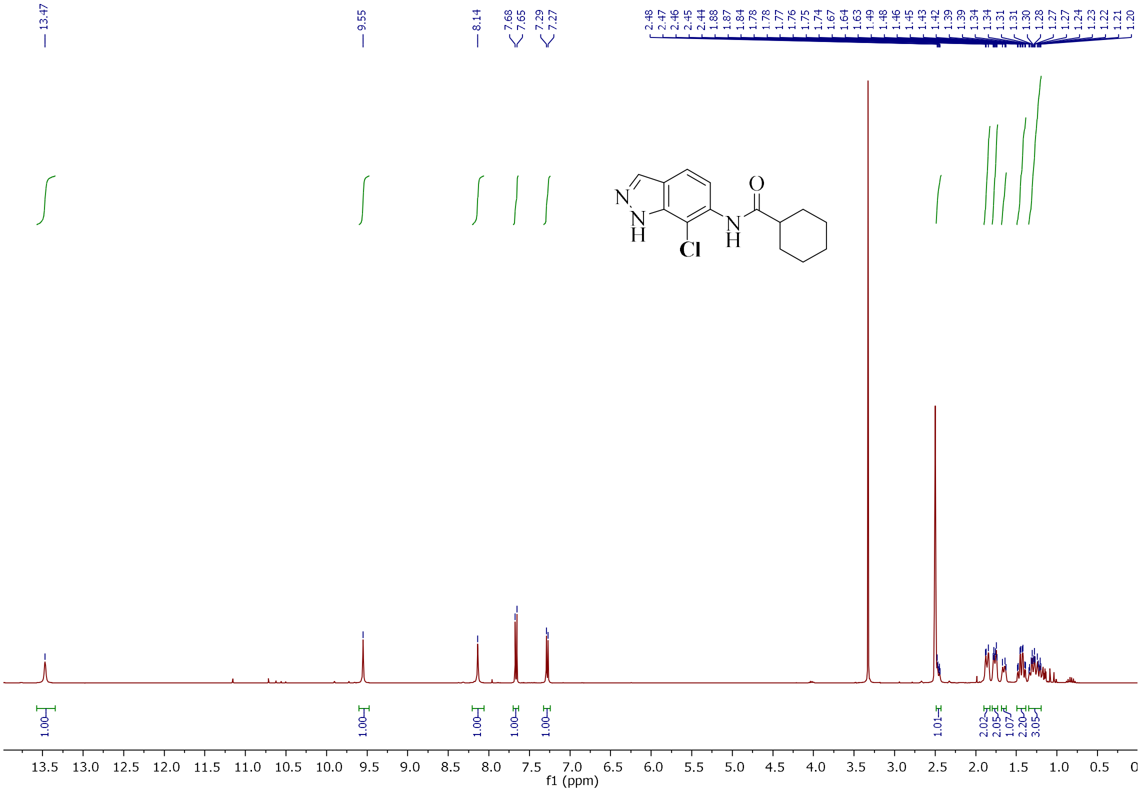

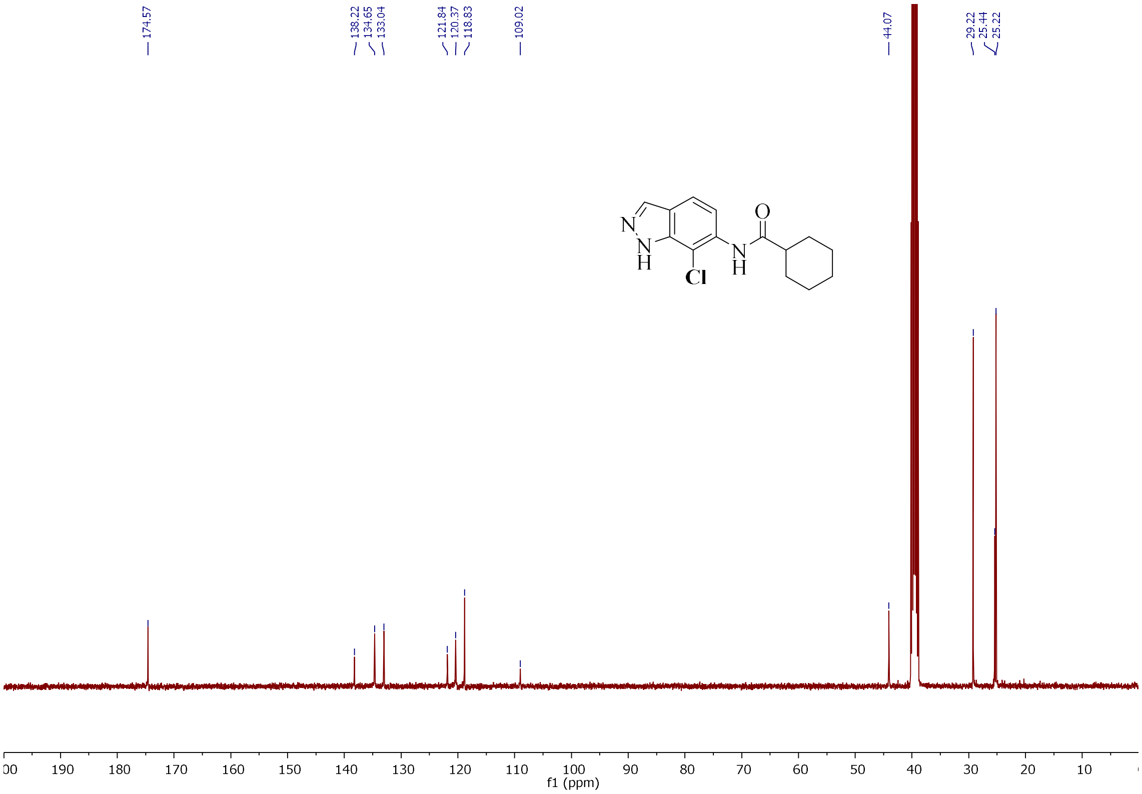

N-(5-(tert-butyl)-4-chloro-1-(cyclohexanecarbonyl)-1H-pyrazol-3-yl)cyclohexanecarboxamide: (126)

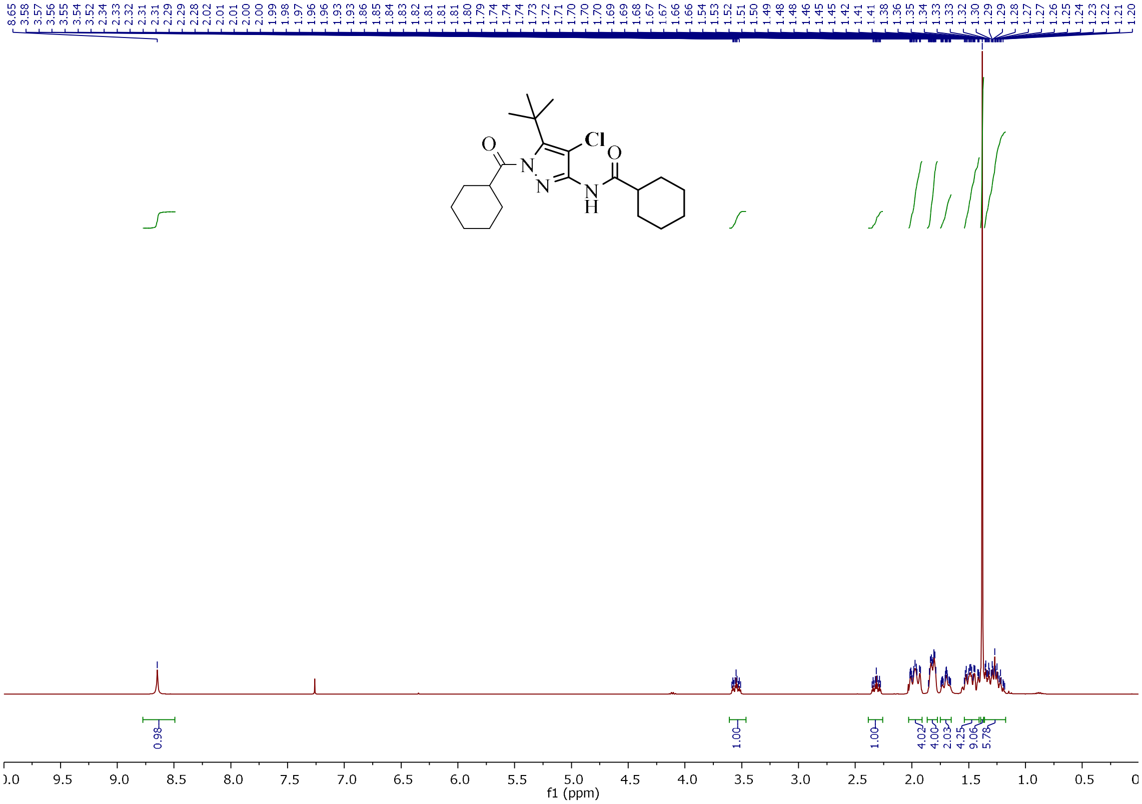

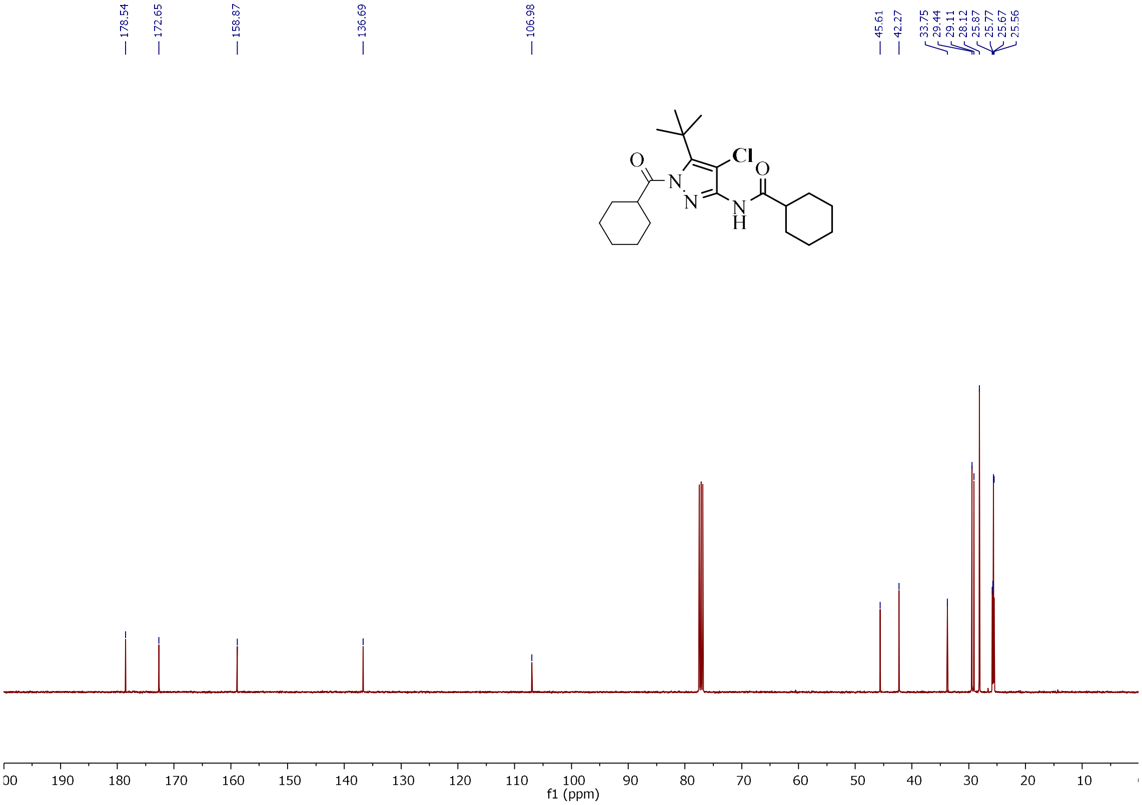

N-(4-chloro-2-oxo-2H-chromen-3-yl)cyclohexanecarboxamide: (127)

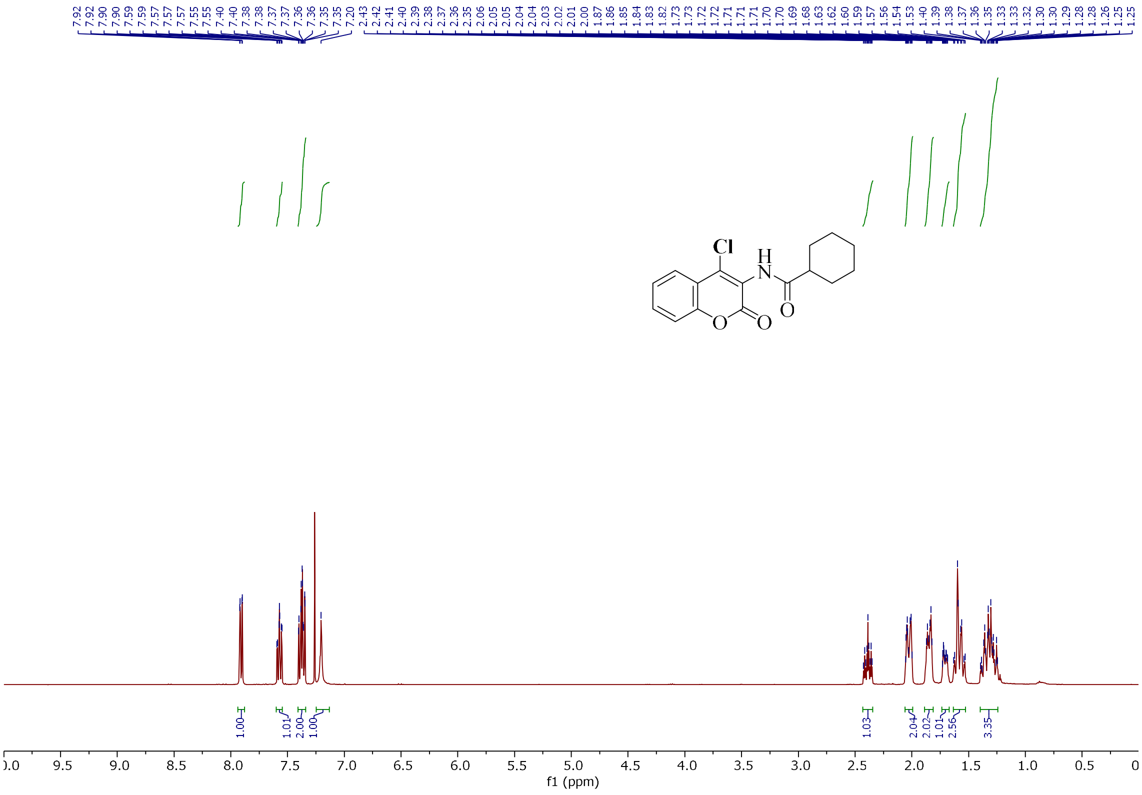

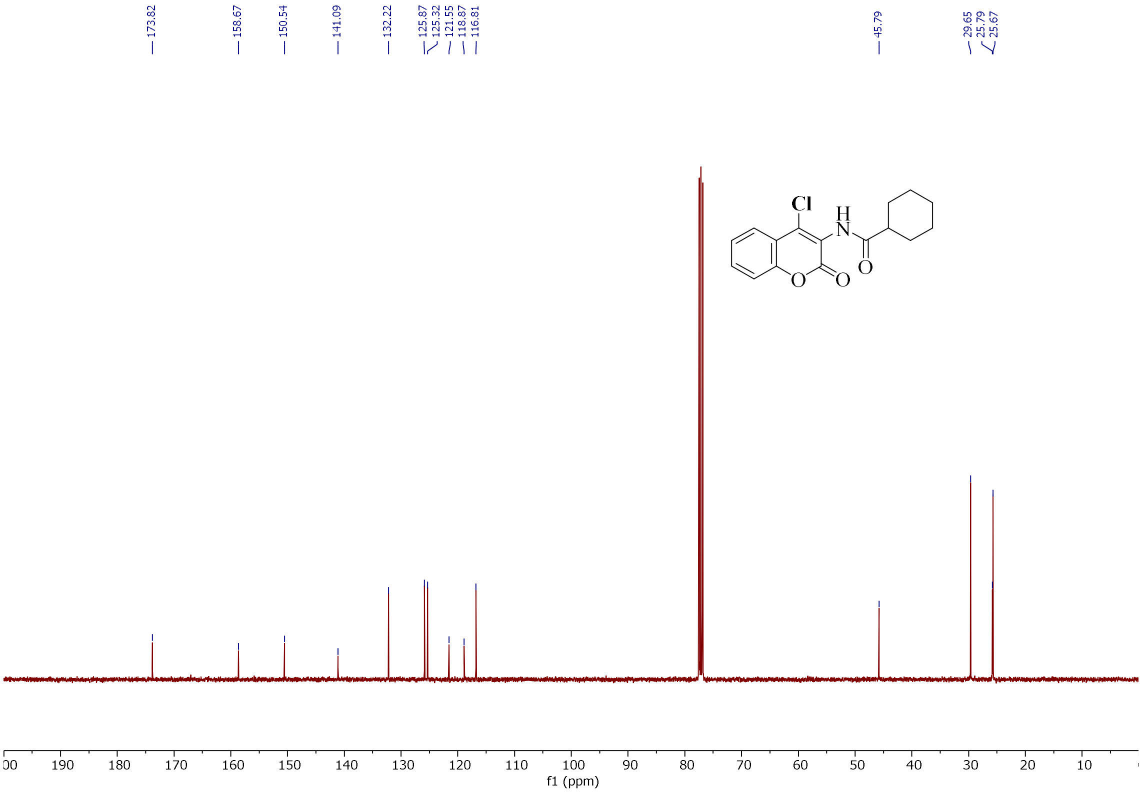

***N*-(4-chloro-2-oxo-2*H*-chromen-3-yl)acetamide: (128)**

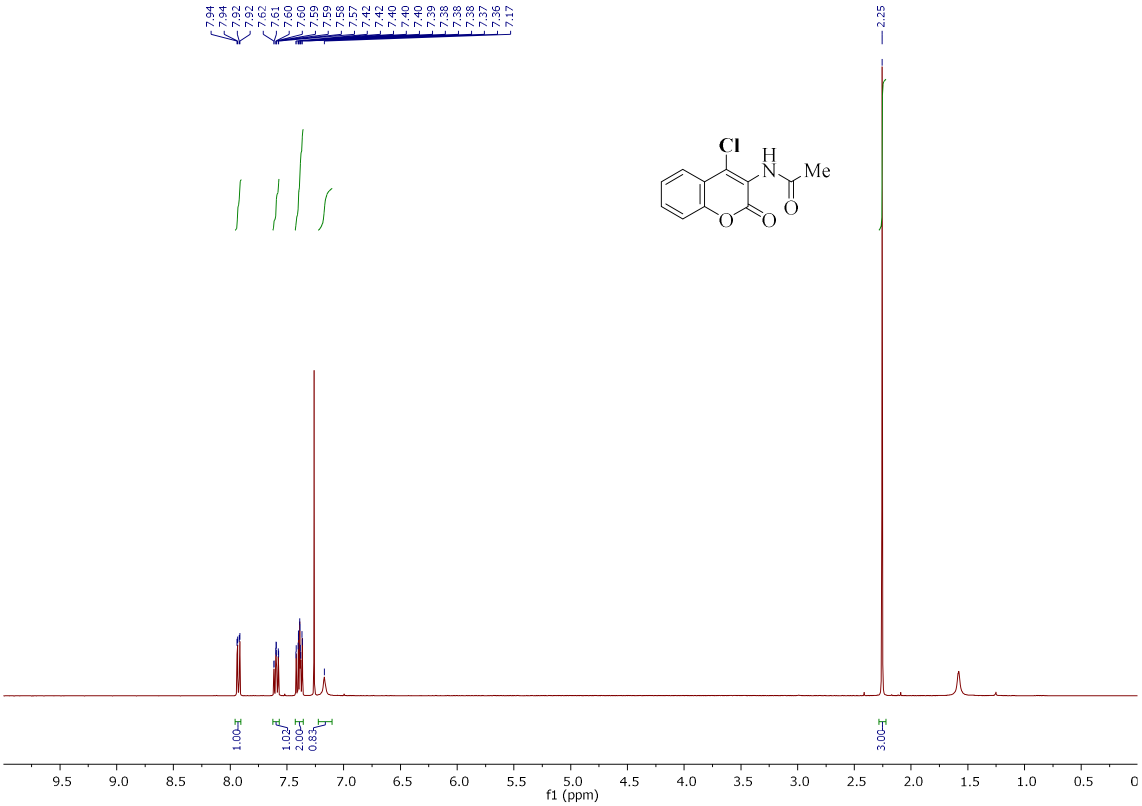

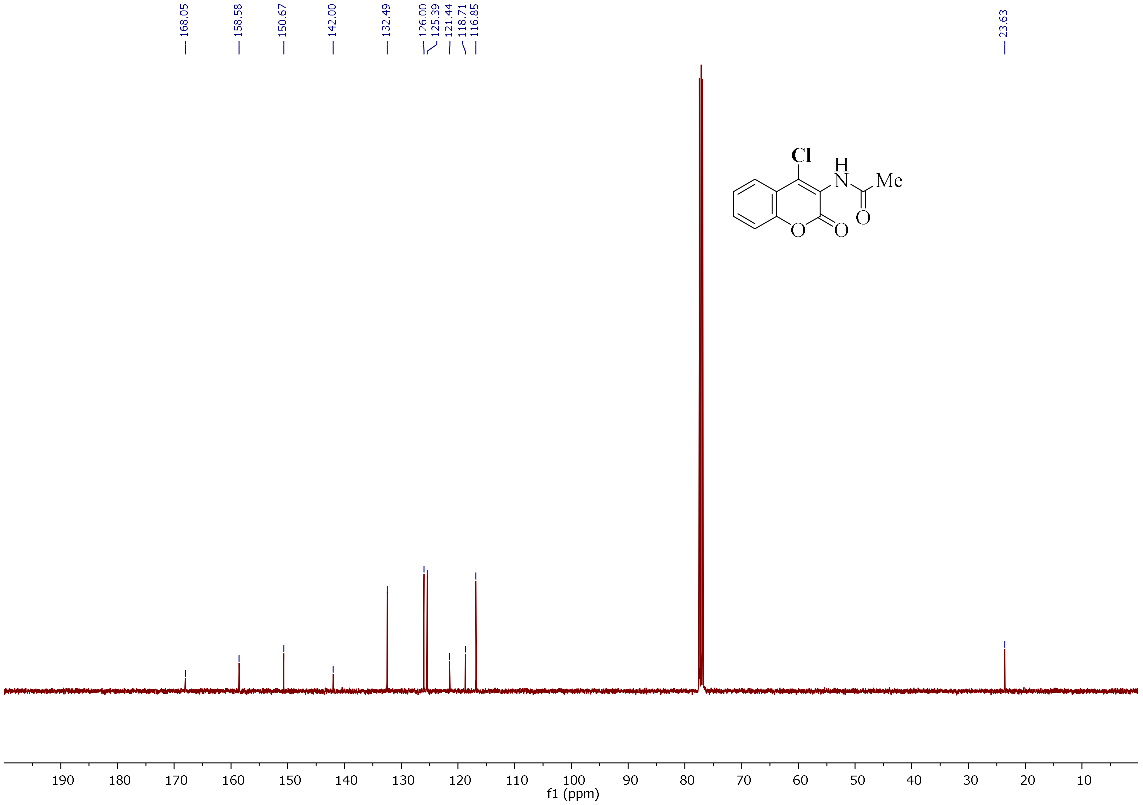

***N*-(4-chloro-2-oxo-2H-chromen-3-yl)-2-phenylacetamide: (129)**

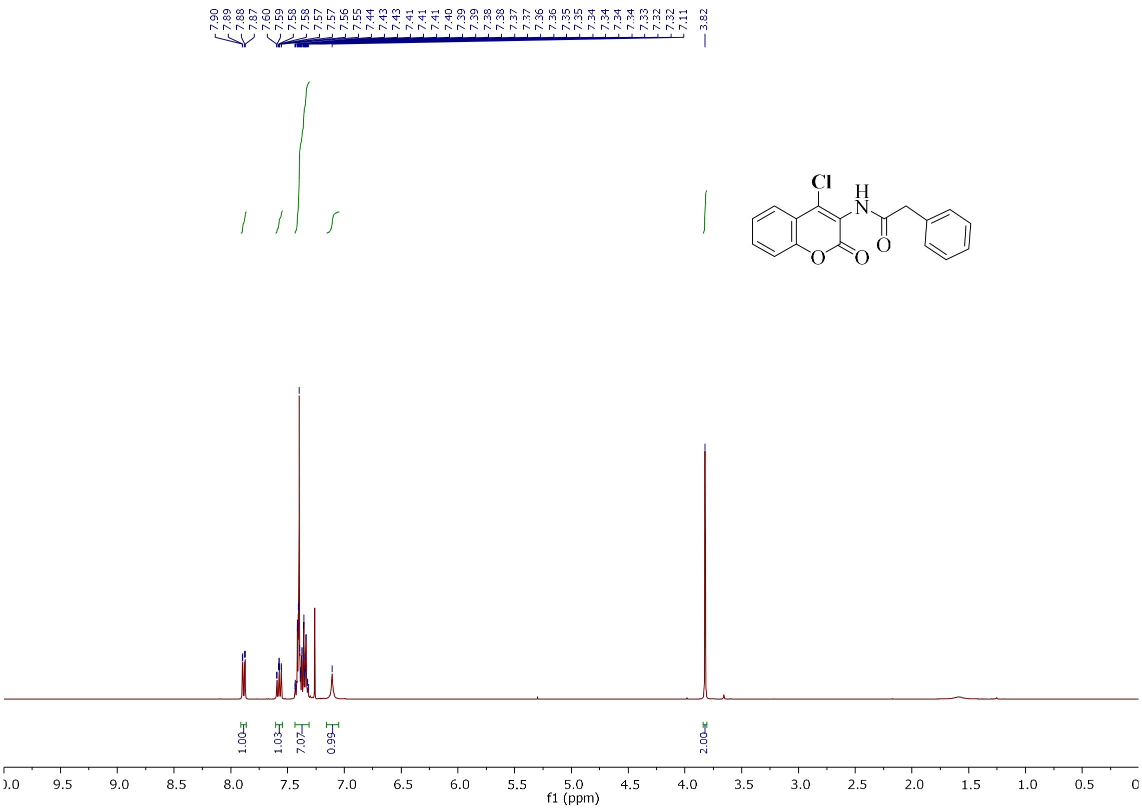

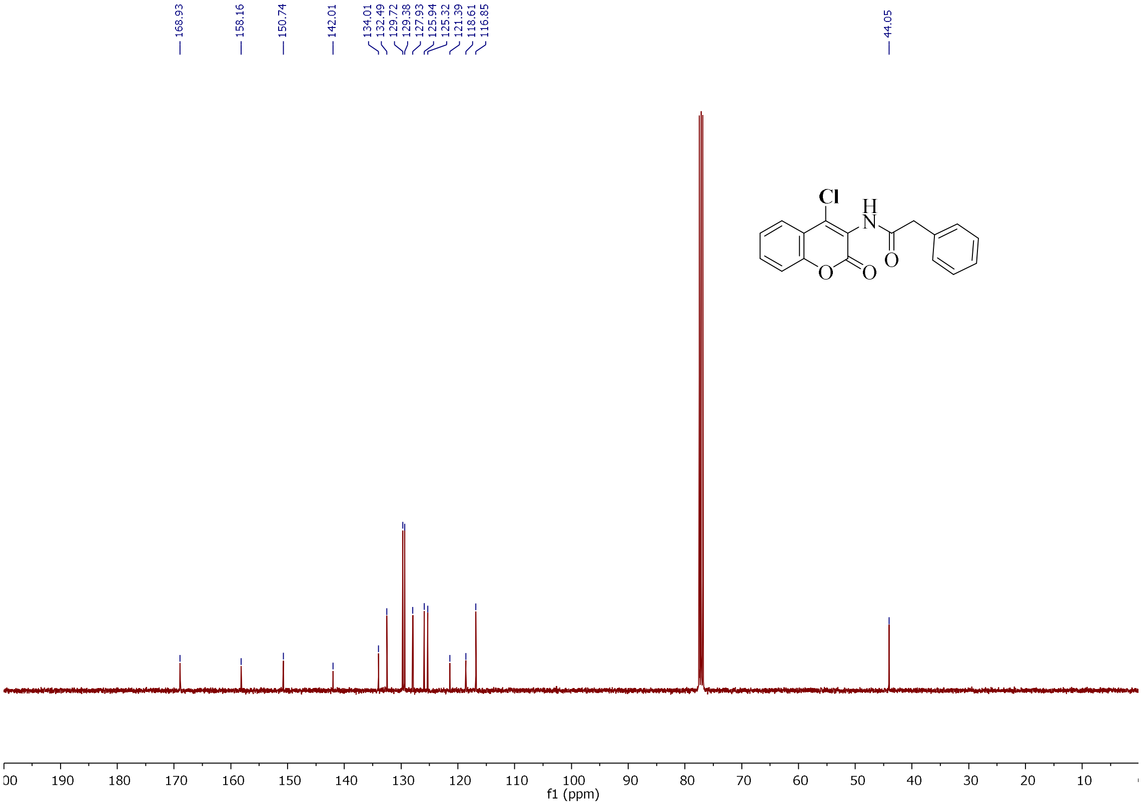

N-(4-chloro-2-oxo-2H-chromen-3-yl)-1-naphthamide: (130)

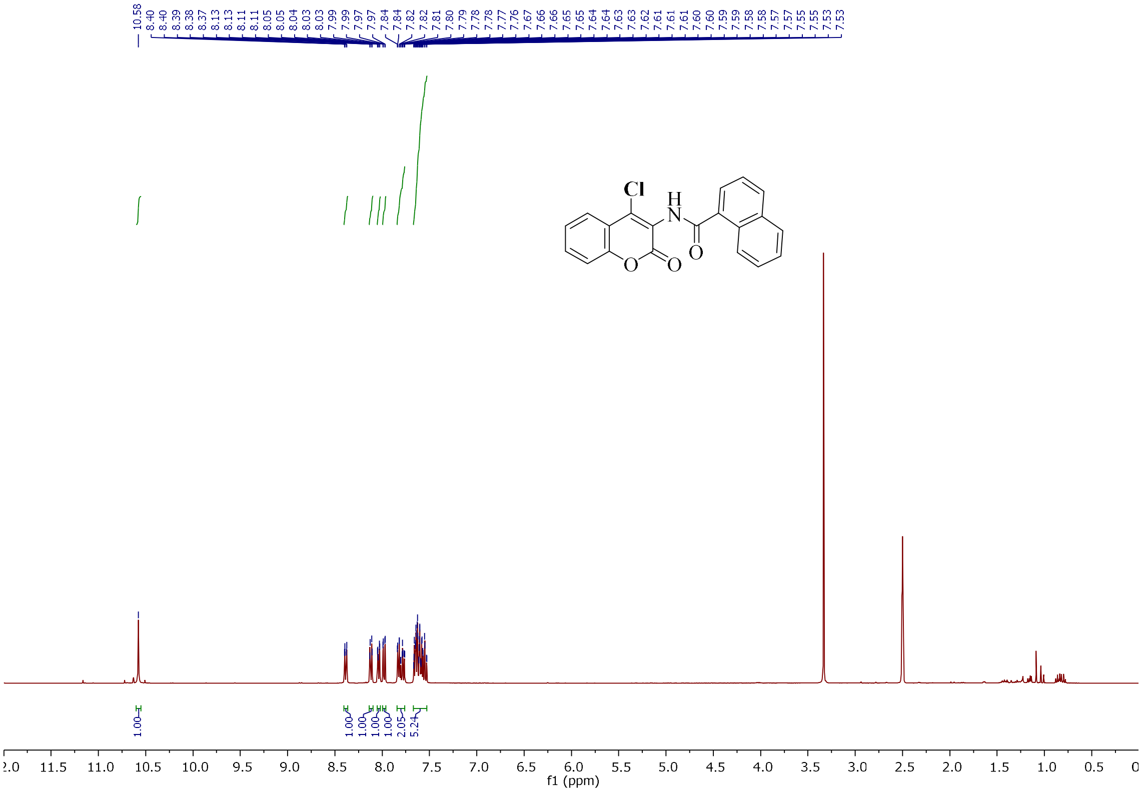

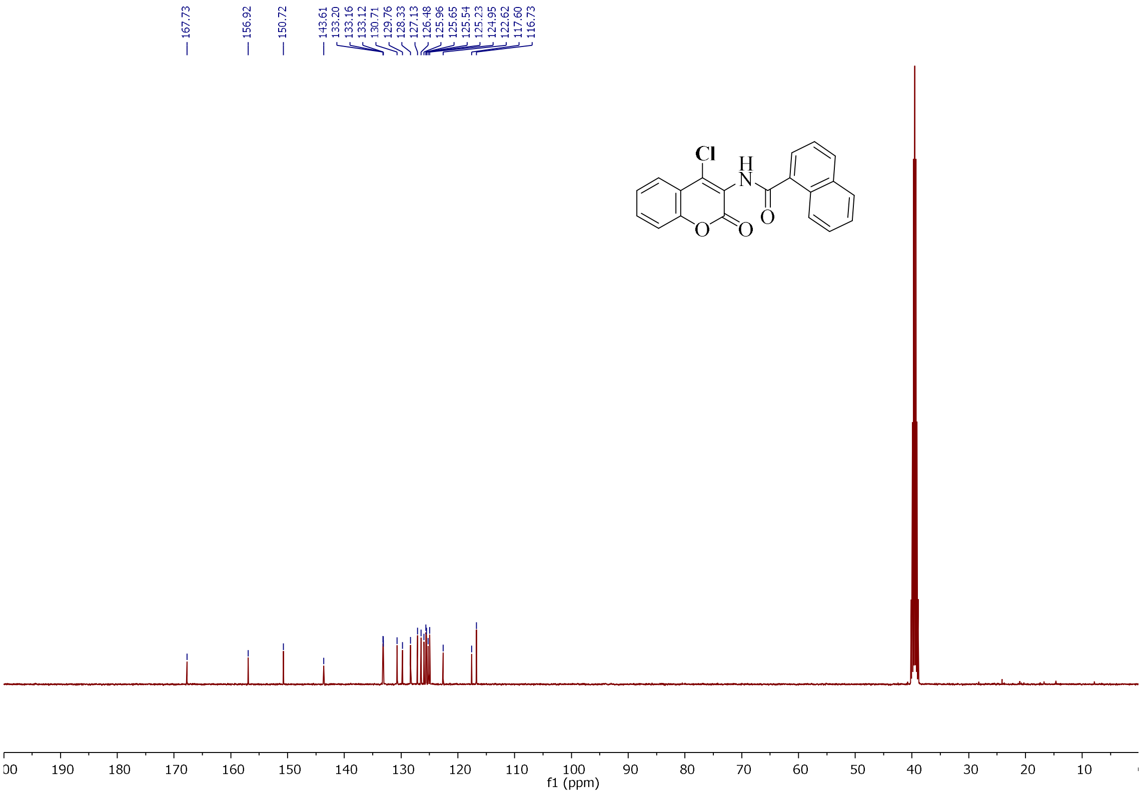

N-(8-bromo-4-methyl-2-oxo-2H-chromen-7-yl)propionamide: (131)

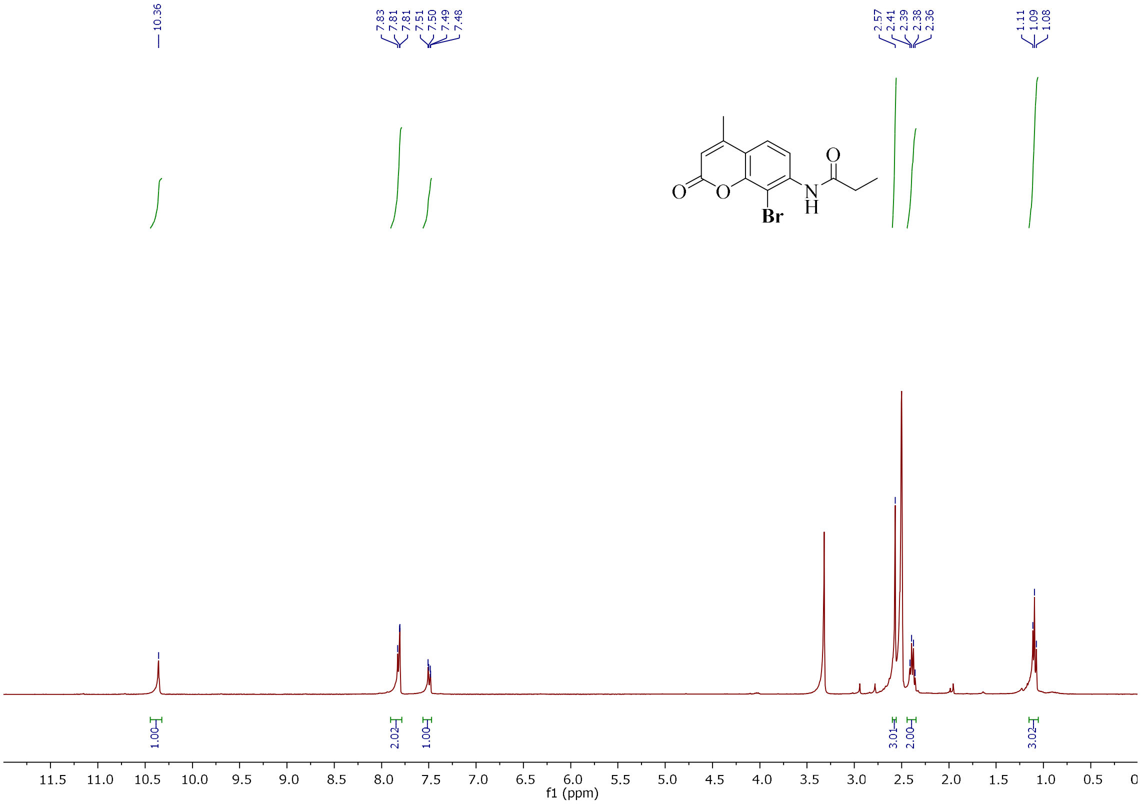

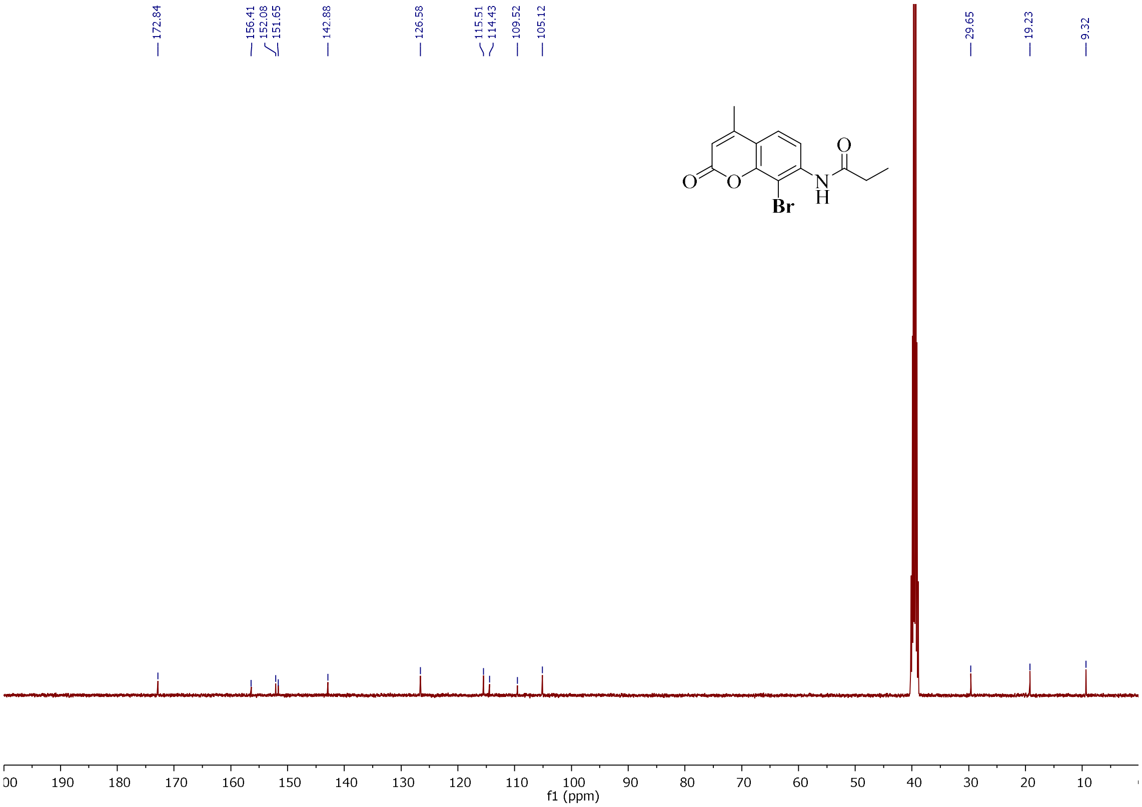

_N_-(4-bromo-5-(_tert_-butyl)-1-propionyl-1_H_-pyrazol-3-yl)propionamide: (132)

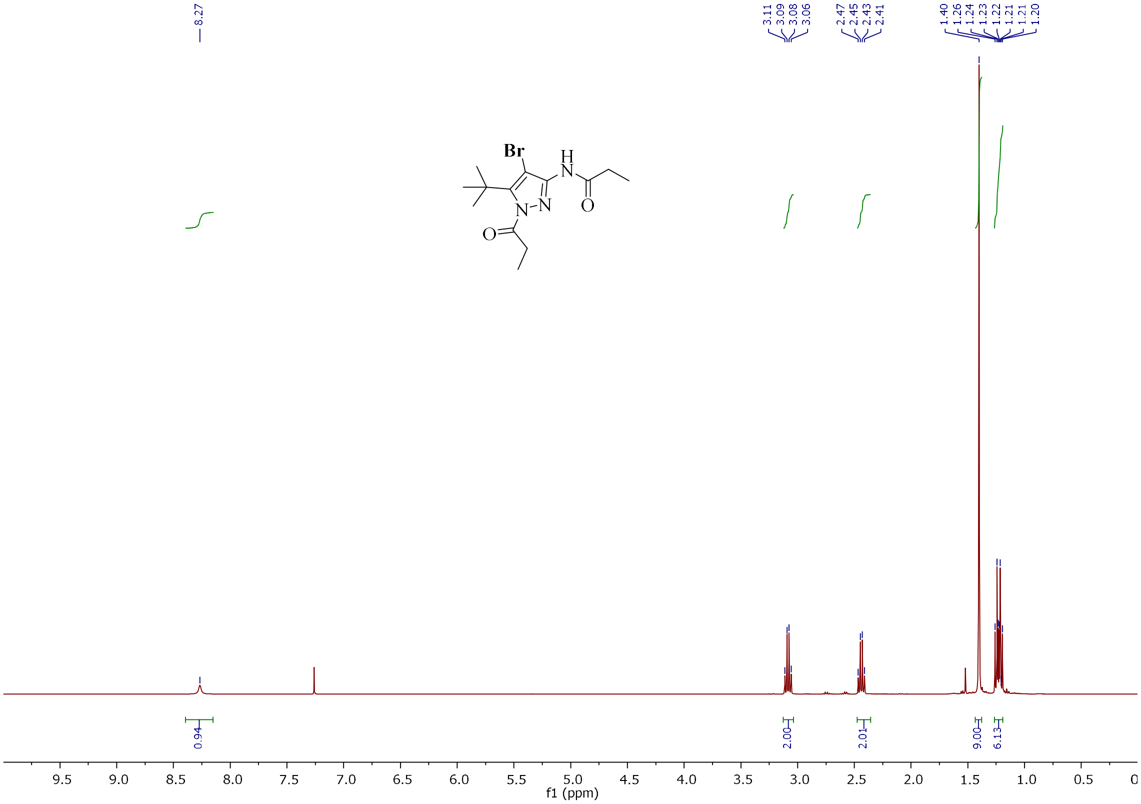

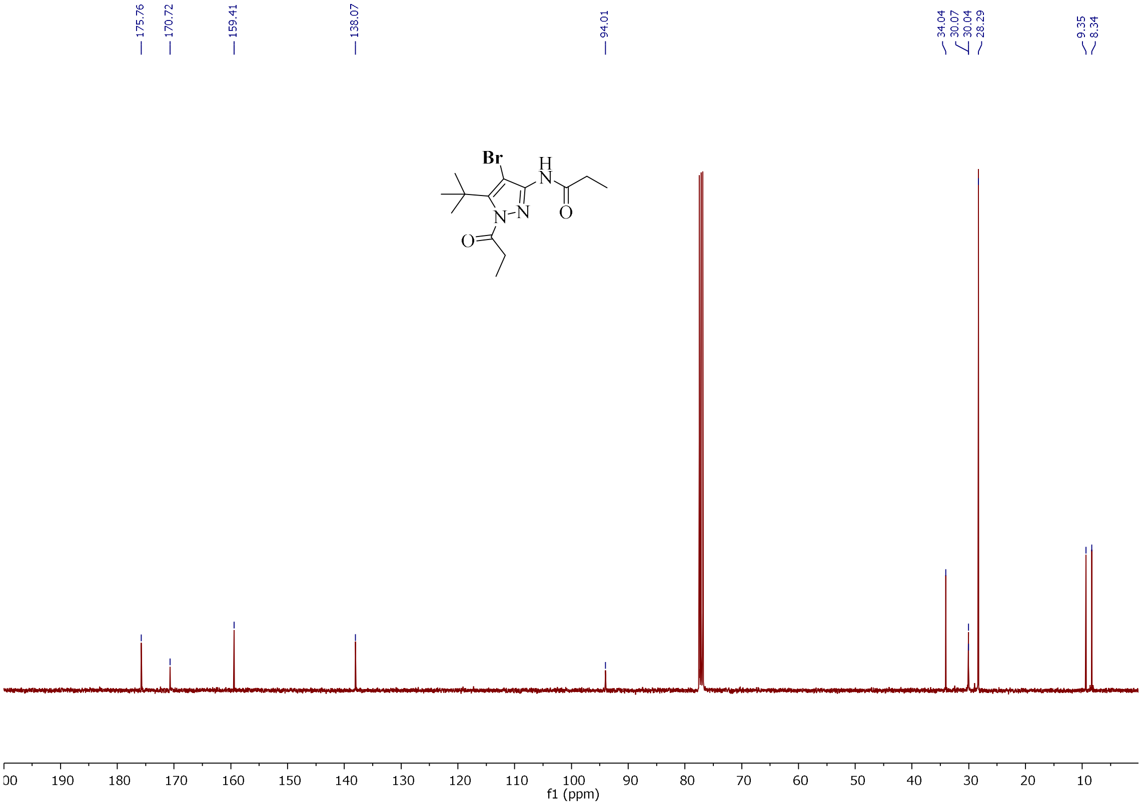

_N_-(4-bromo-1,3-diphenyl-1_H_-pyrazol-5-yl)propionamide: (133)

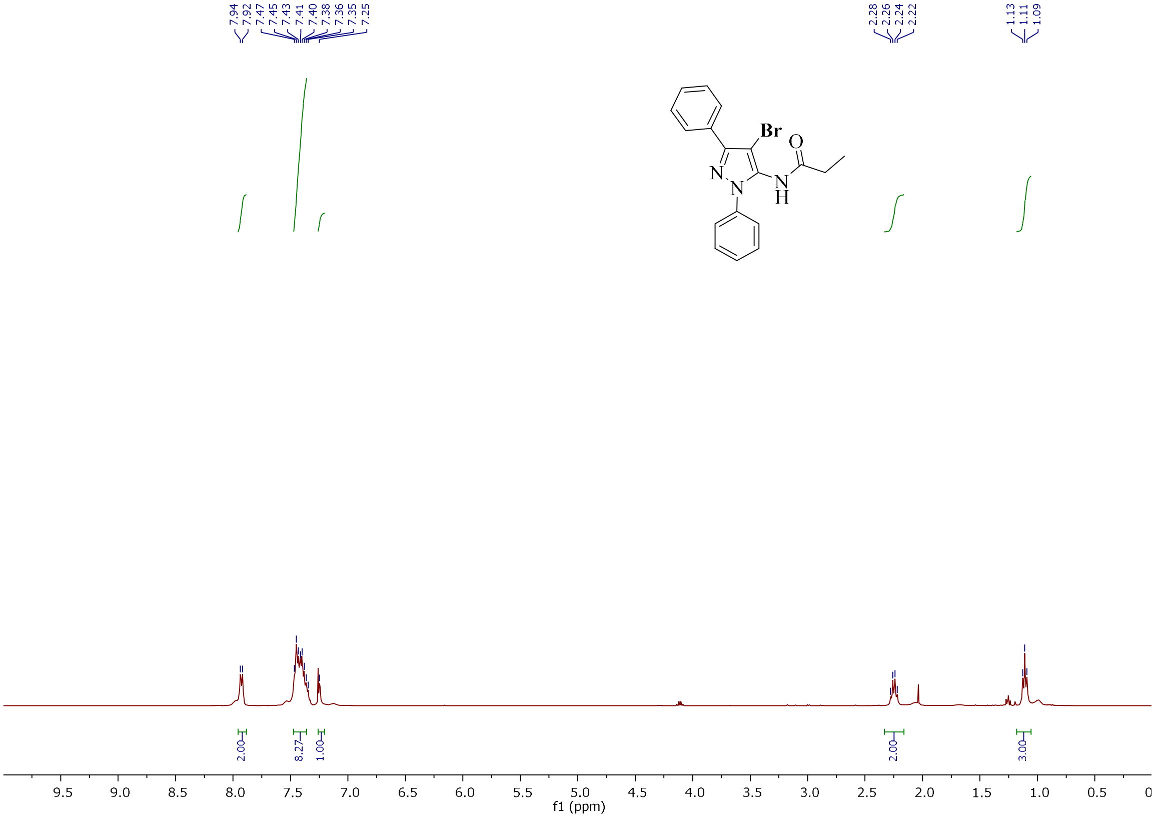

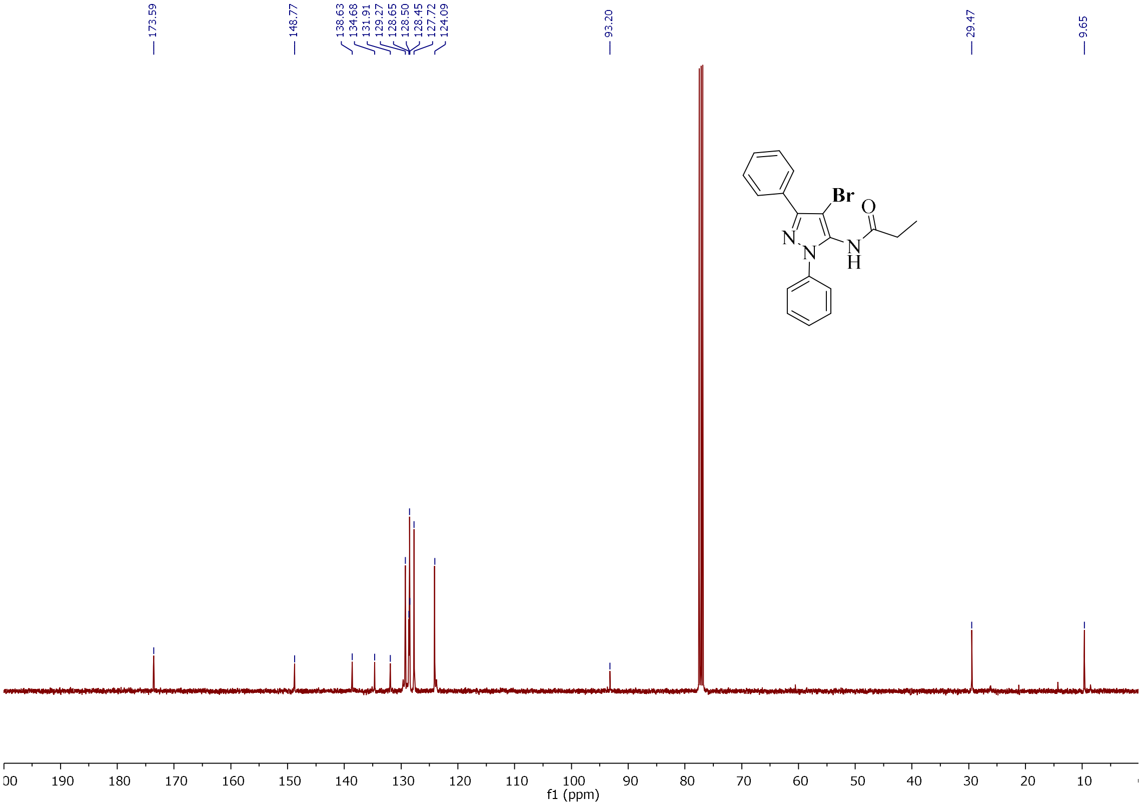

_N_-(7-bromo-2-methylbenzo[_d_]thiazol-6-yl)propionamide: (134)

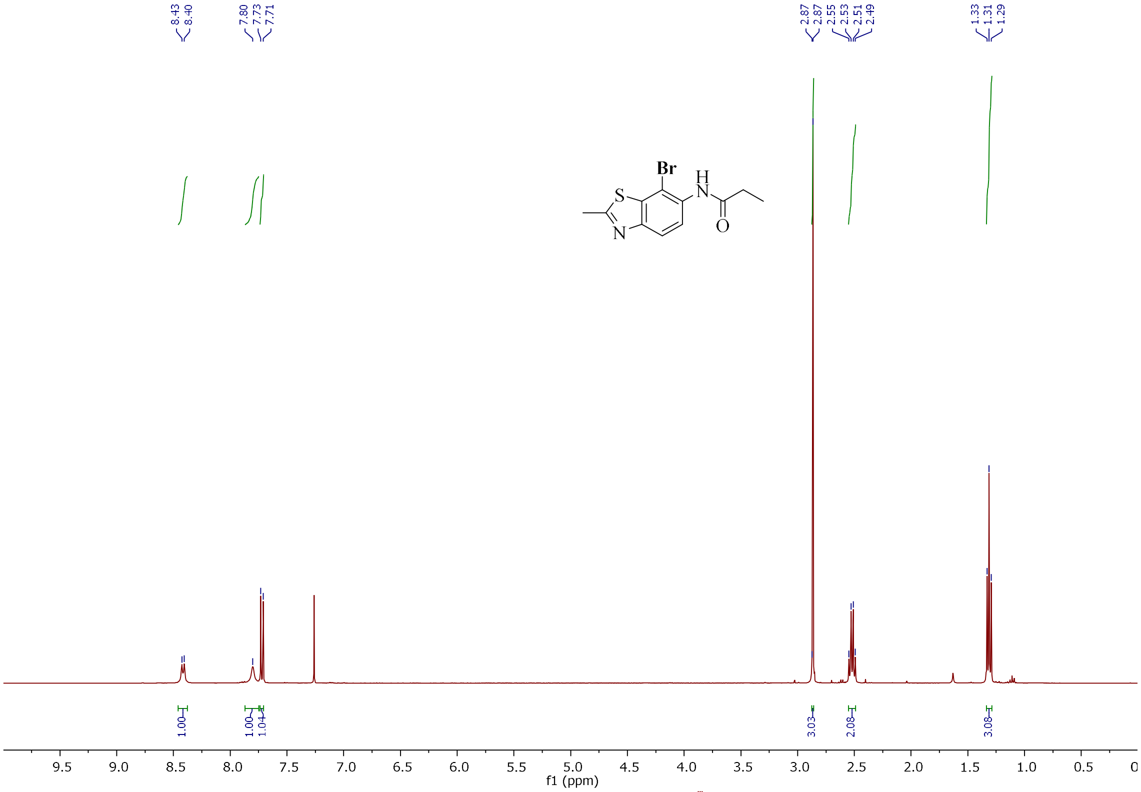

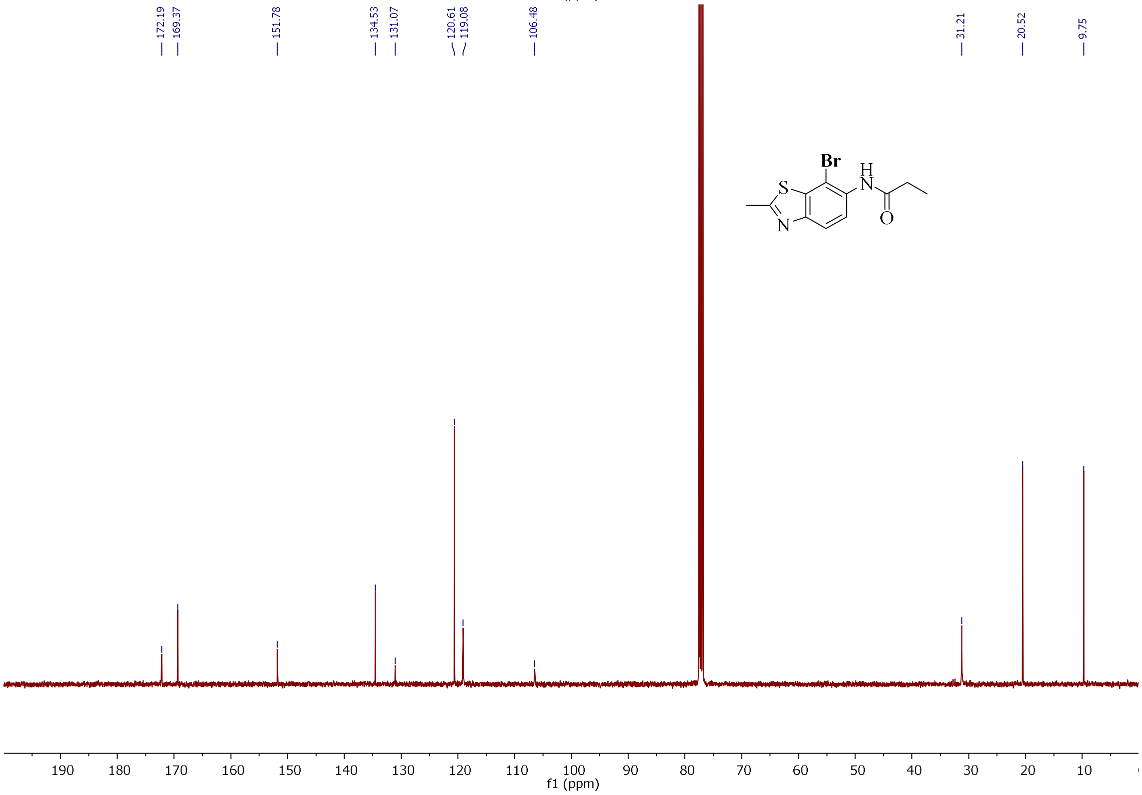

***N*-(4-chlorophenyl)-5-methylisoxazole-4-carboxamide: (135)**

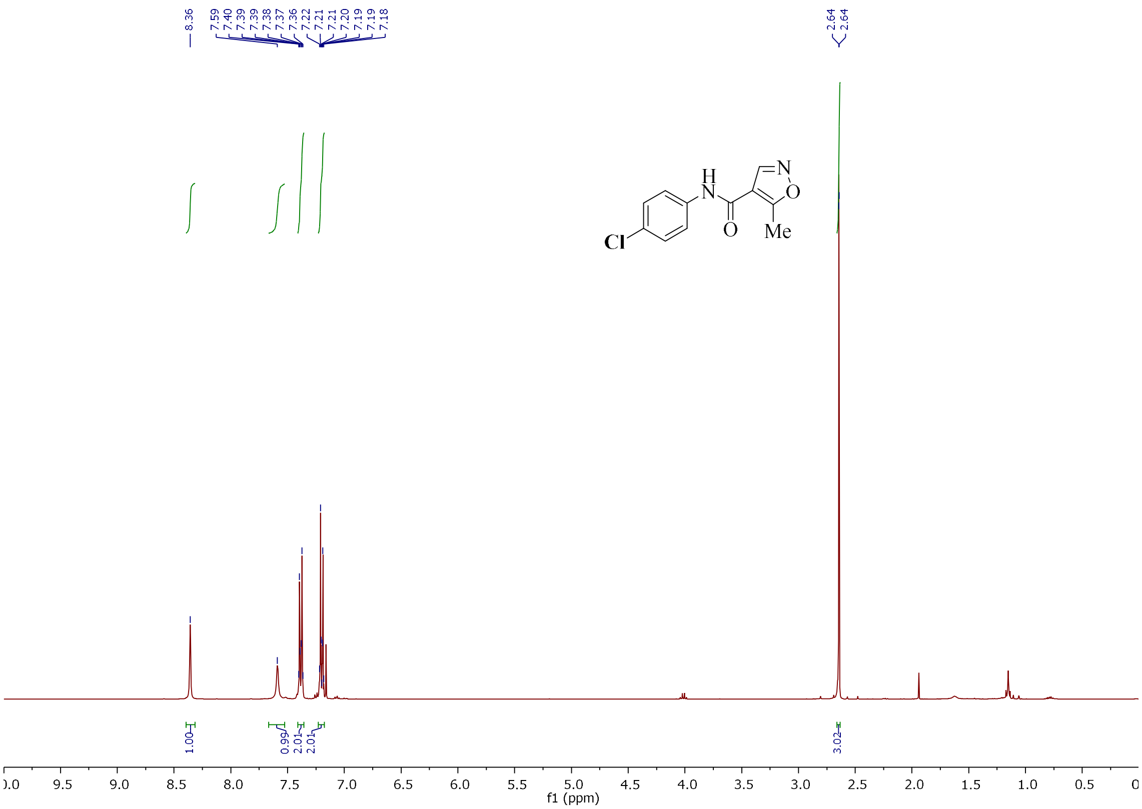

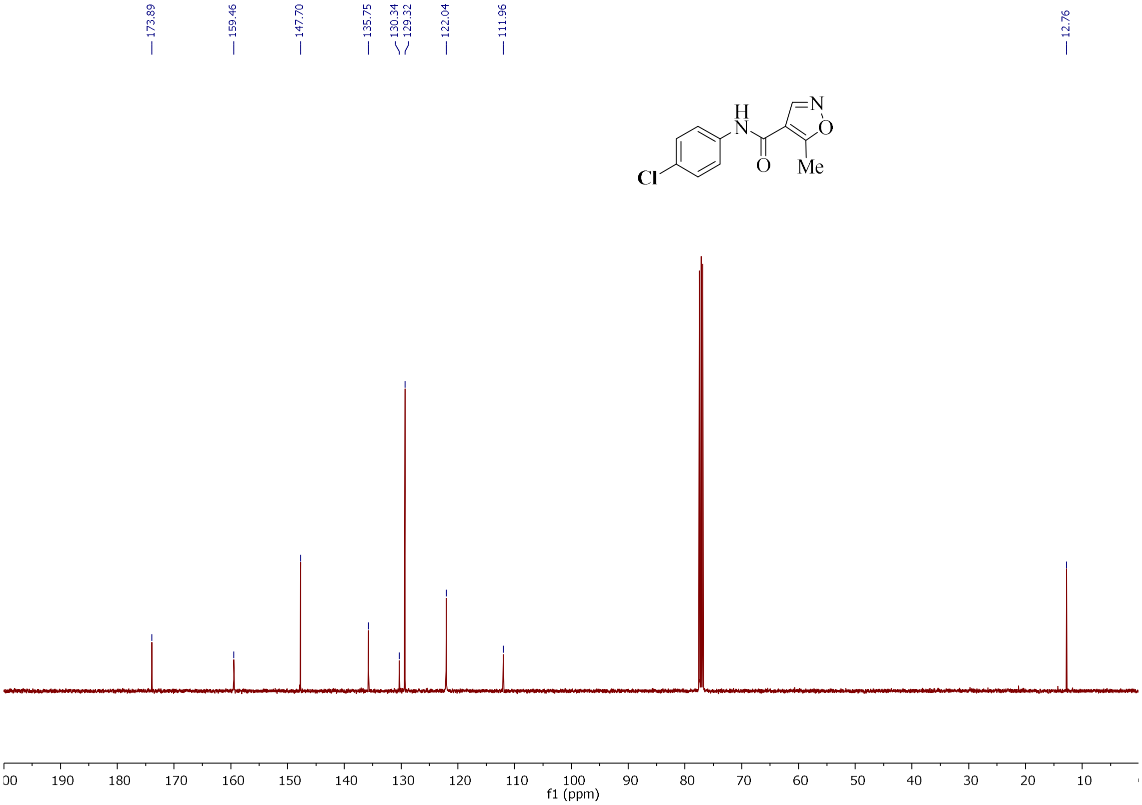

2-((4-chlorophenyl)carbamoyl)phenyl acetate: (136)

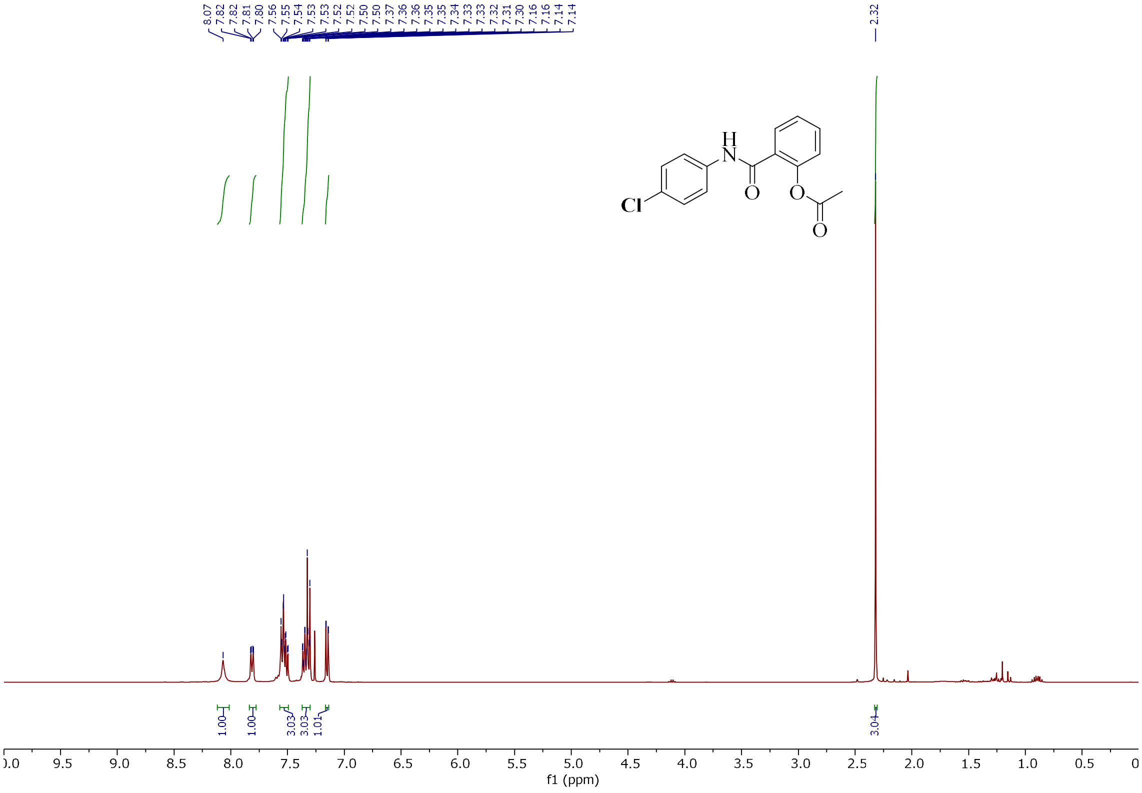

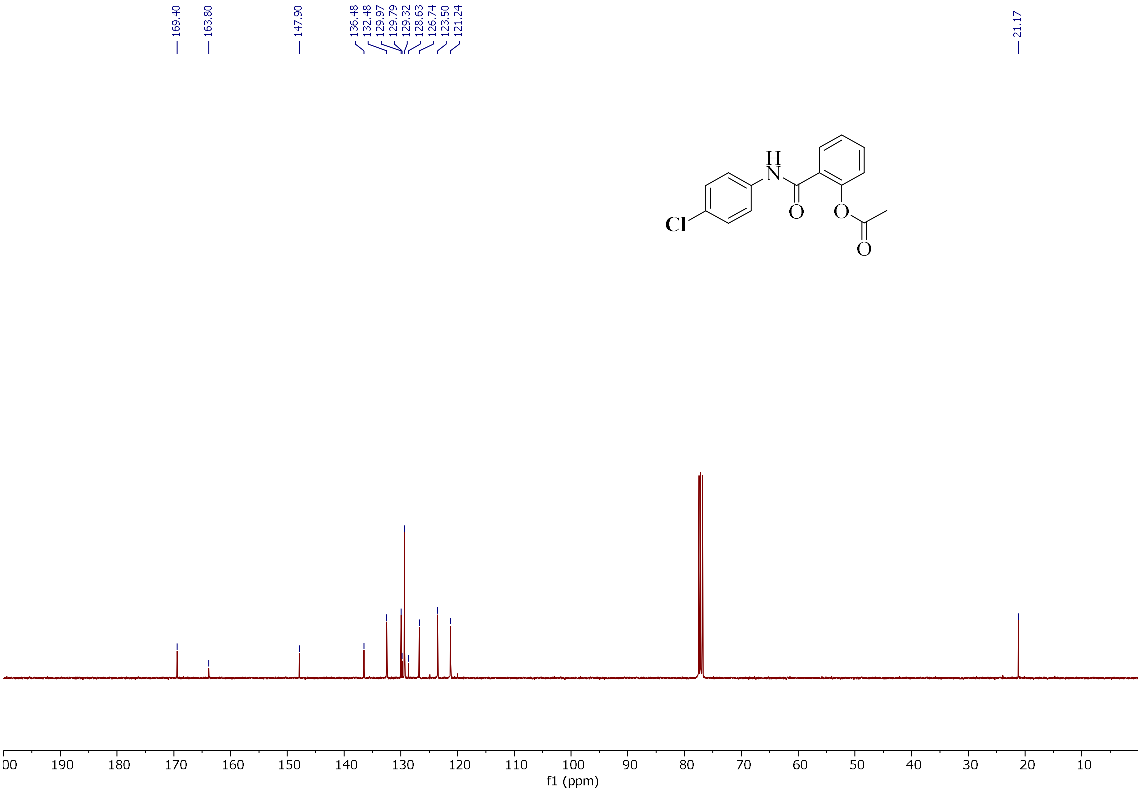

3,5-dichloro-*N*-(4-chloro-2-methylbenzo[*d*]thiazol-5-yl)benzamide: (137)

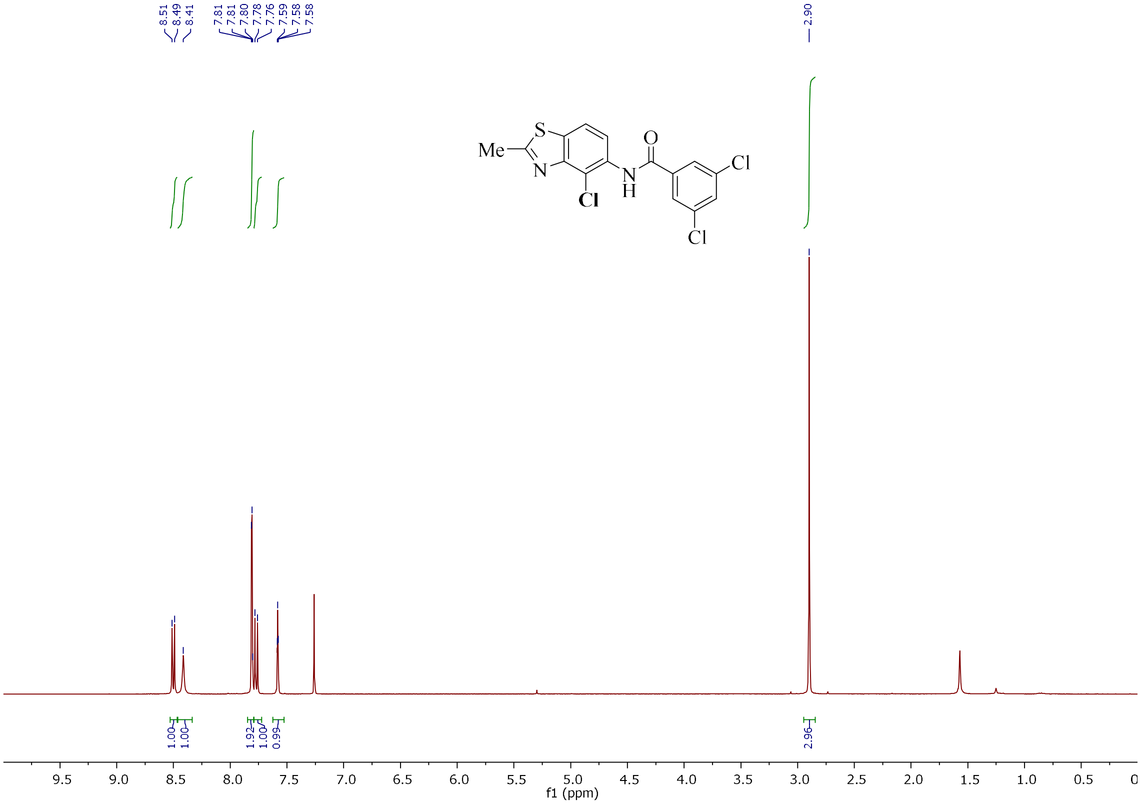

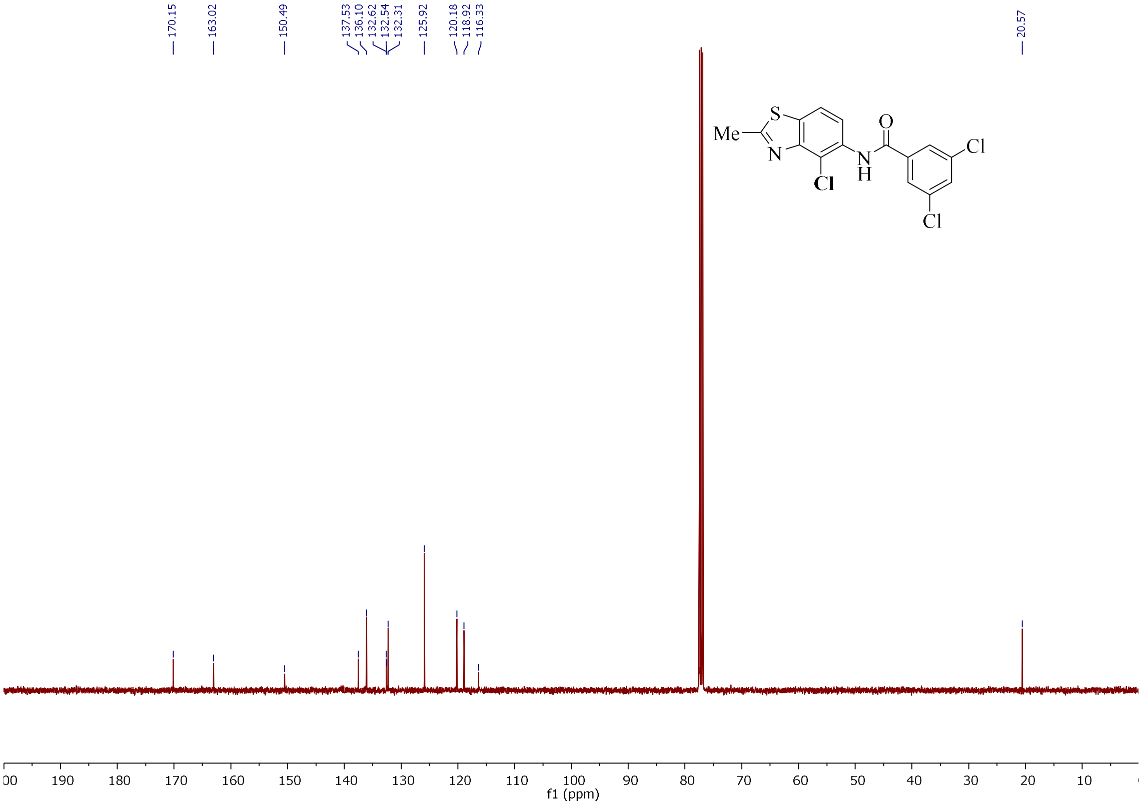

4-Acetamido-5-chloro-2-methoxybenzoic acid: (138)

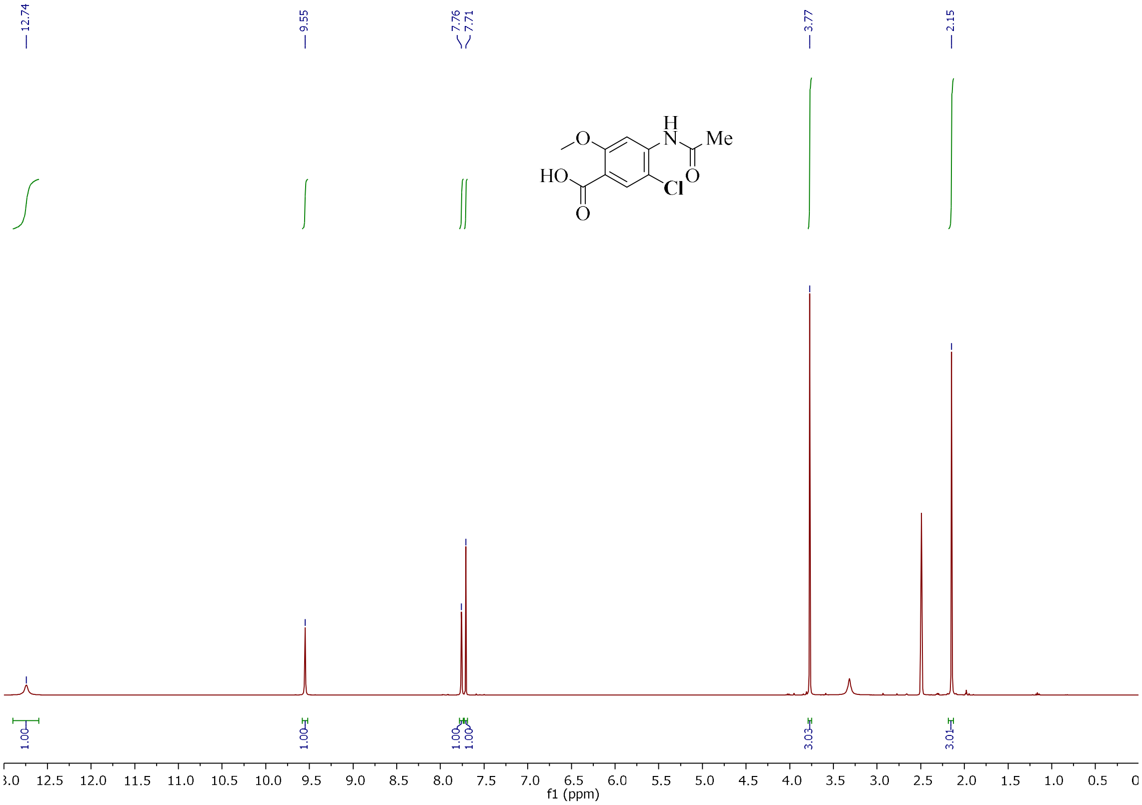

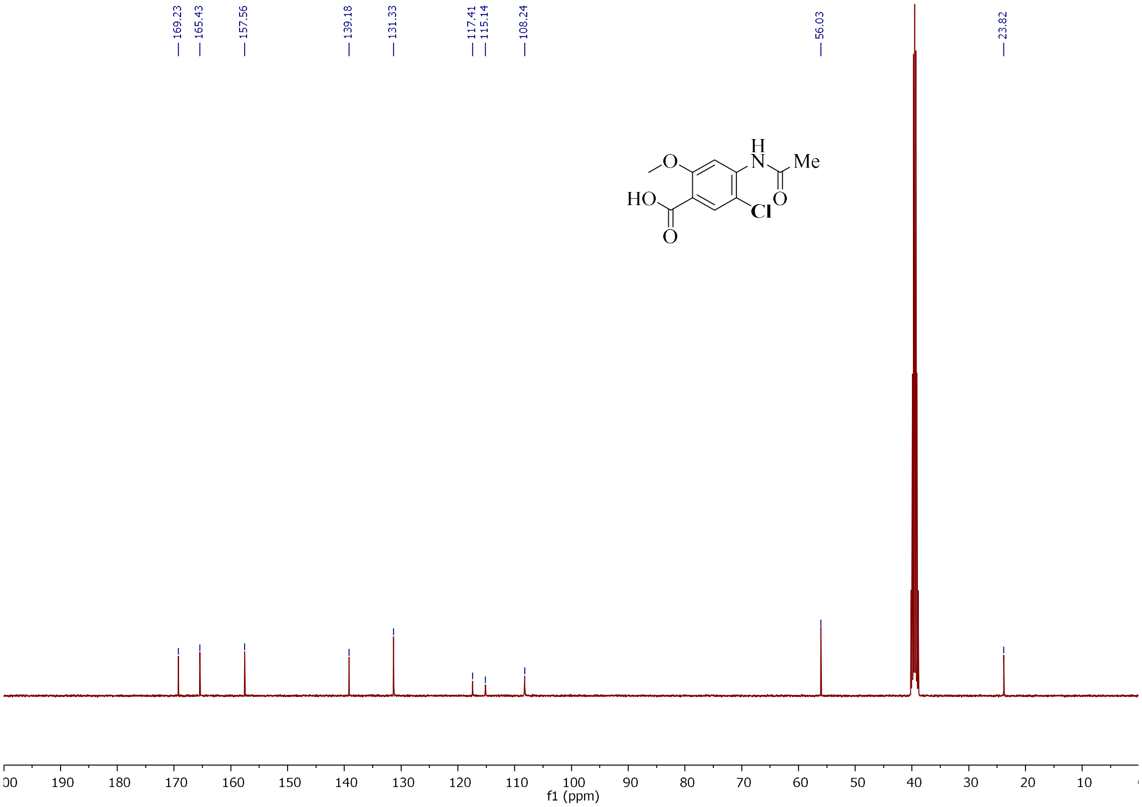

N-(4-(N-(5-chloropyrimidin-2-yl)sulfamoyl)phenyl)cyclohexanecarboxamide: (139)

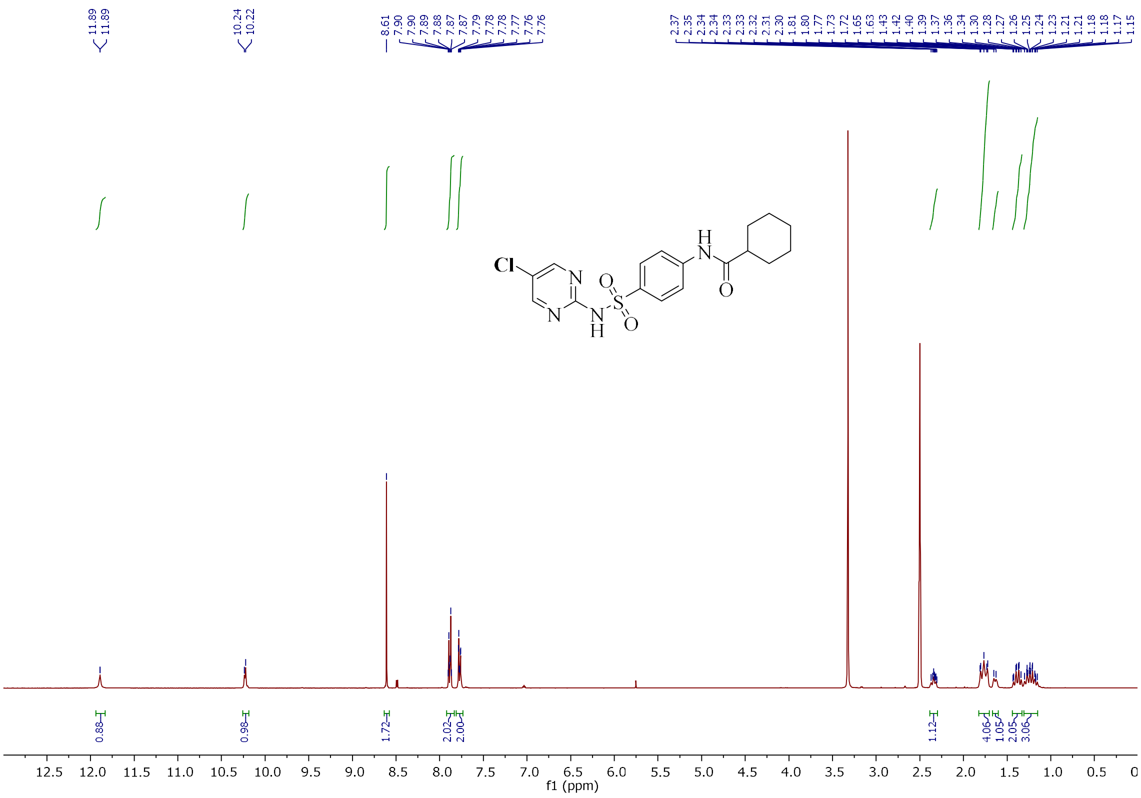

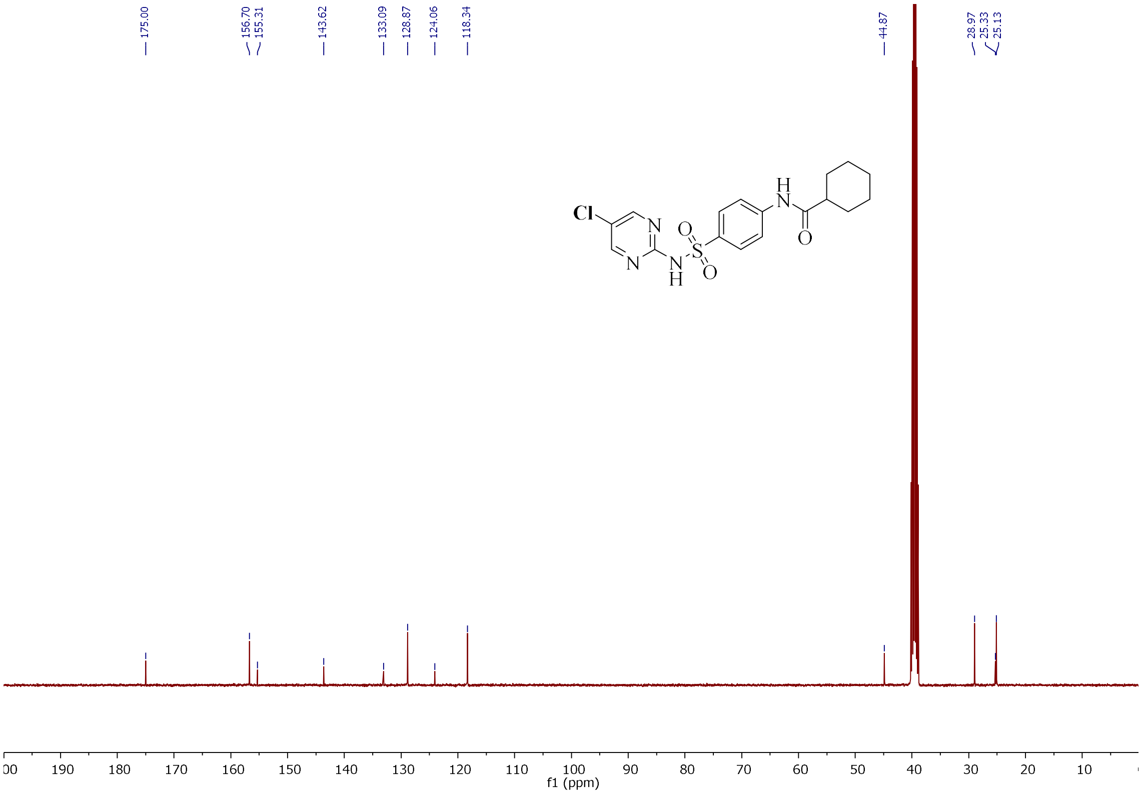

4-(3-chloro-4-(cyclohexanecarboxamido)-5-fluorophenoxy)-N-methylpicolinamide: (140)

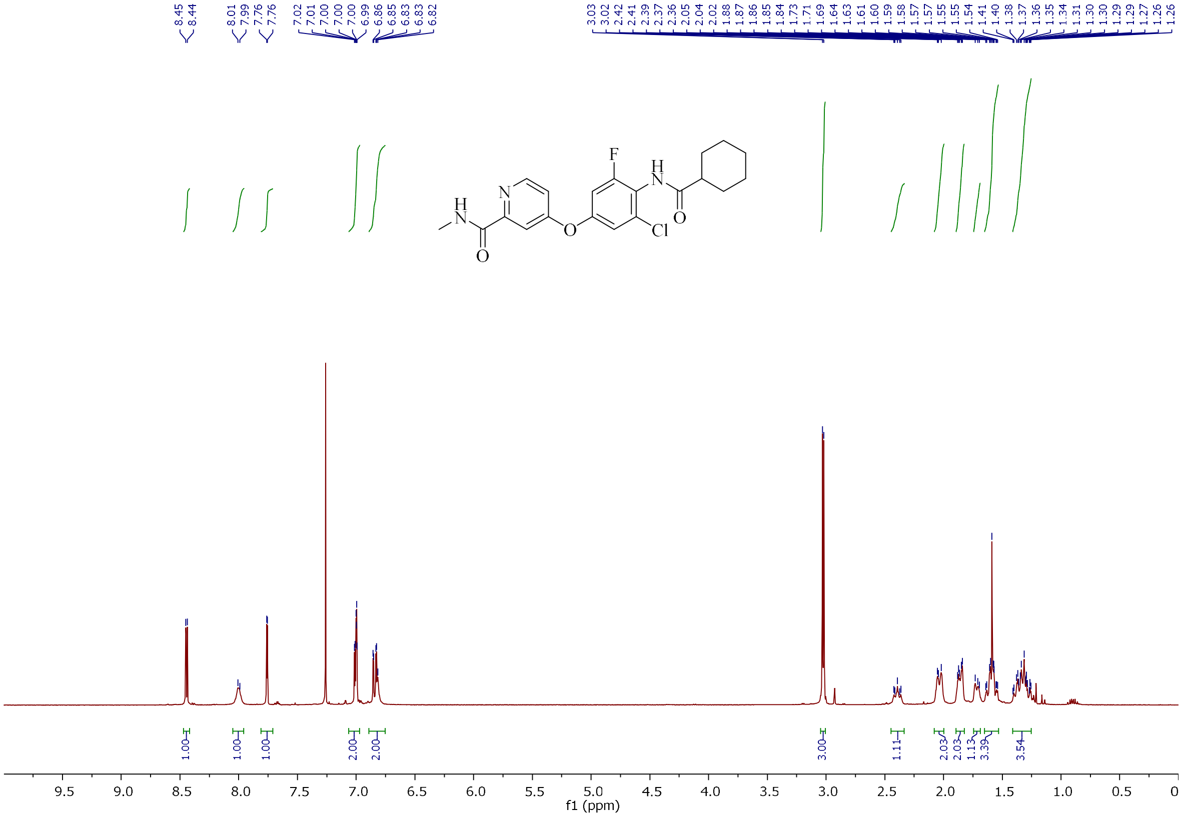

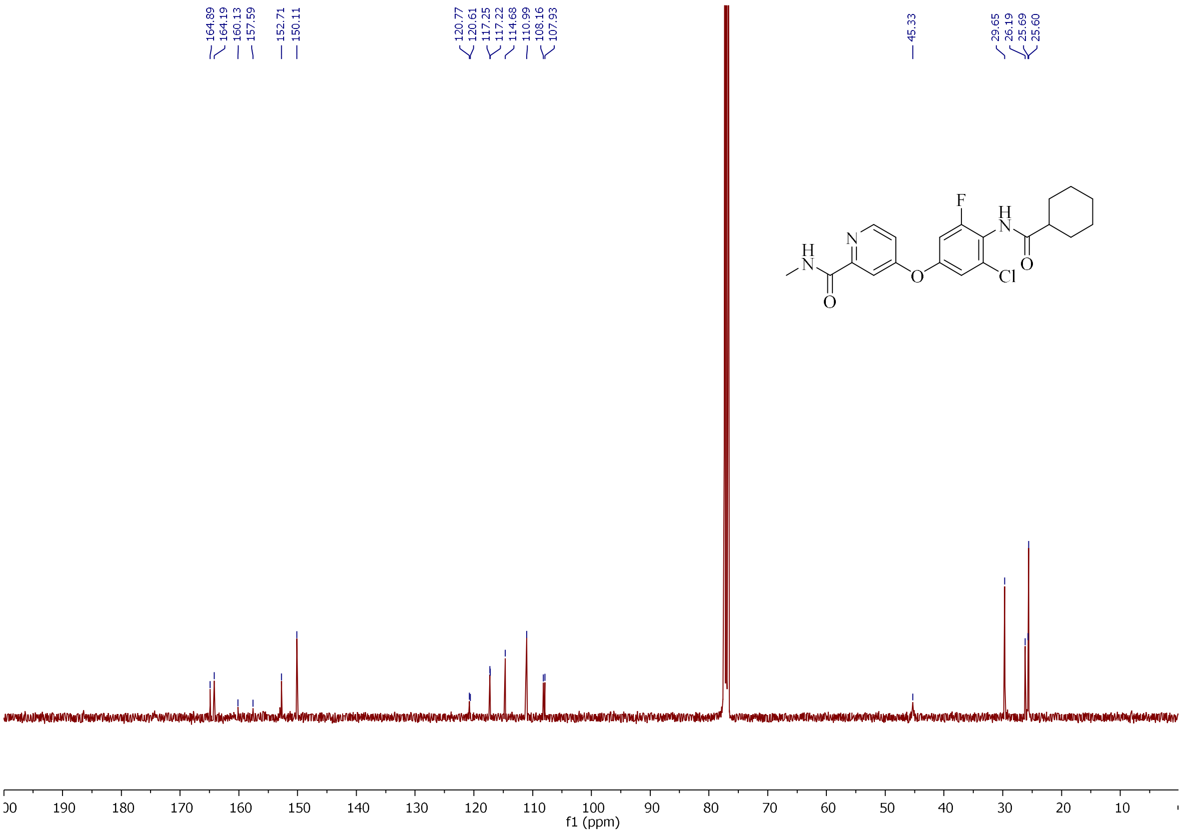

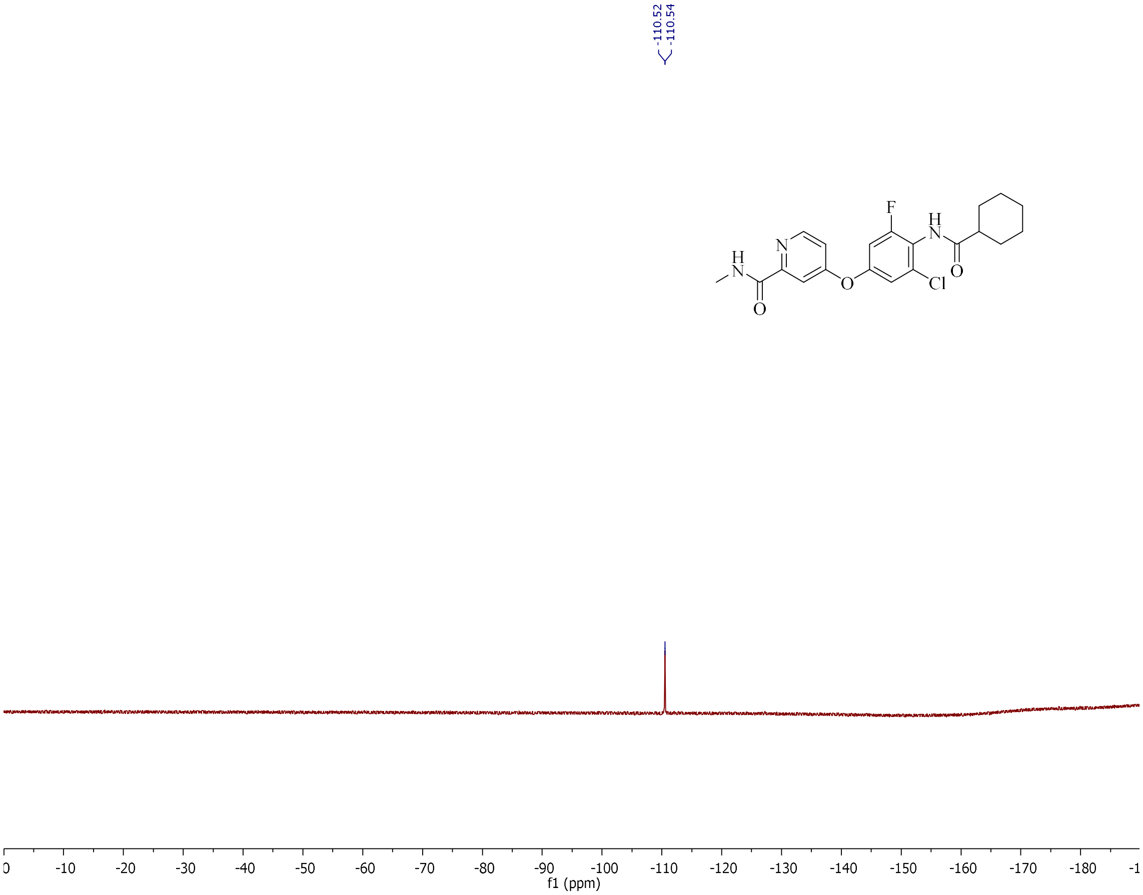
-110.52
-110.54
F
H
N
O
N
H
N
O
O
Cl
0
-10
-20
-30
-40
-50
-60
-70
-80
-90
-100
-110
-120
-130
-140
-150
-160
-170
-180
f1 (ppm)

2-((1-Chloronaphthalen-2-yl)carbamoyl)phenyl acetate: (141)

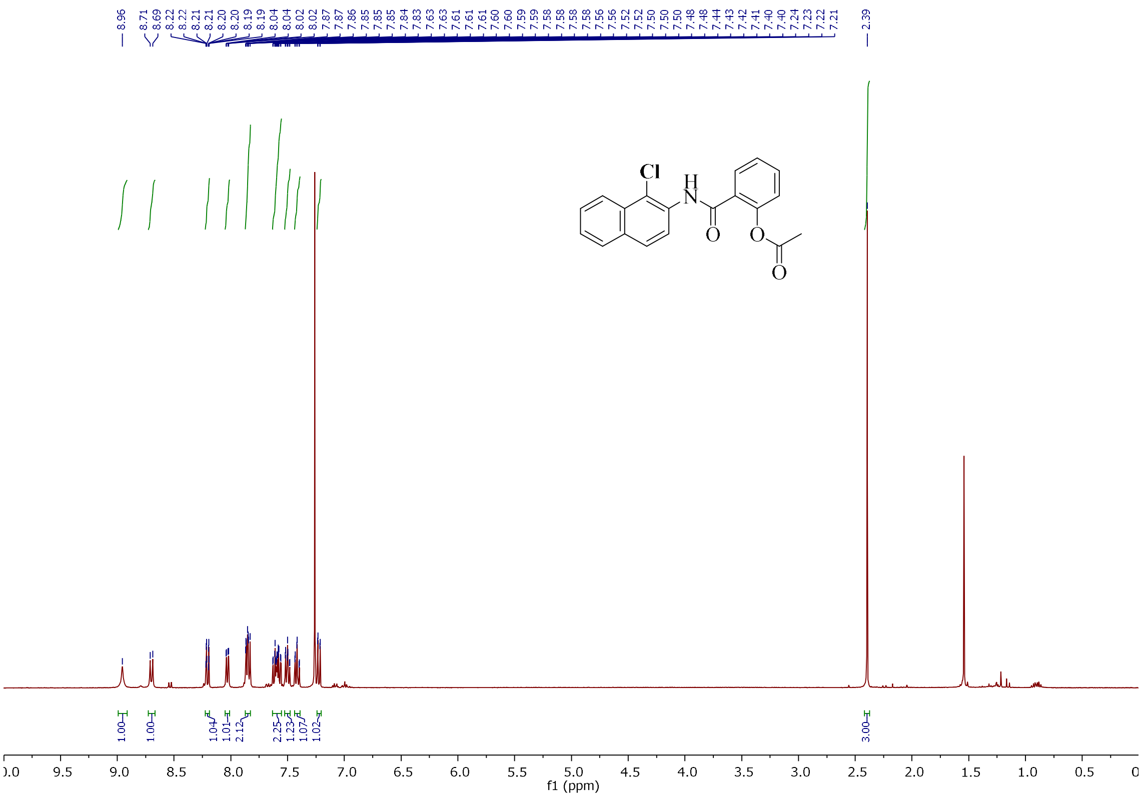

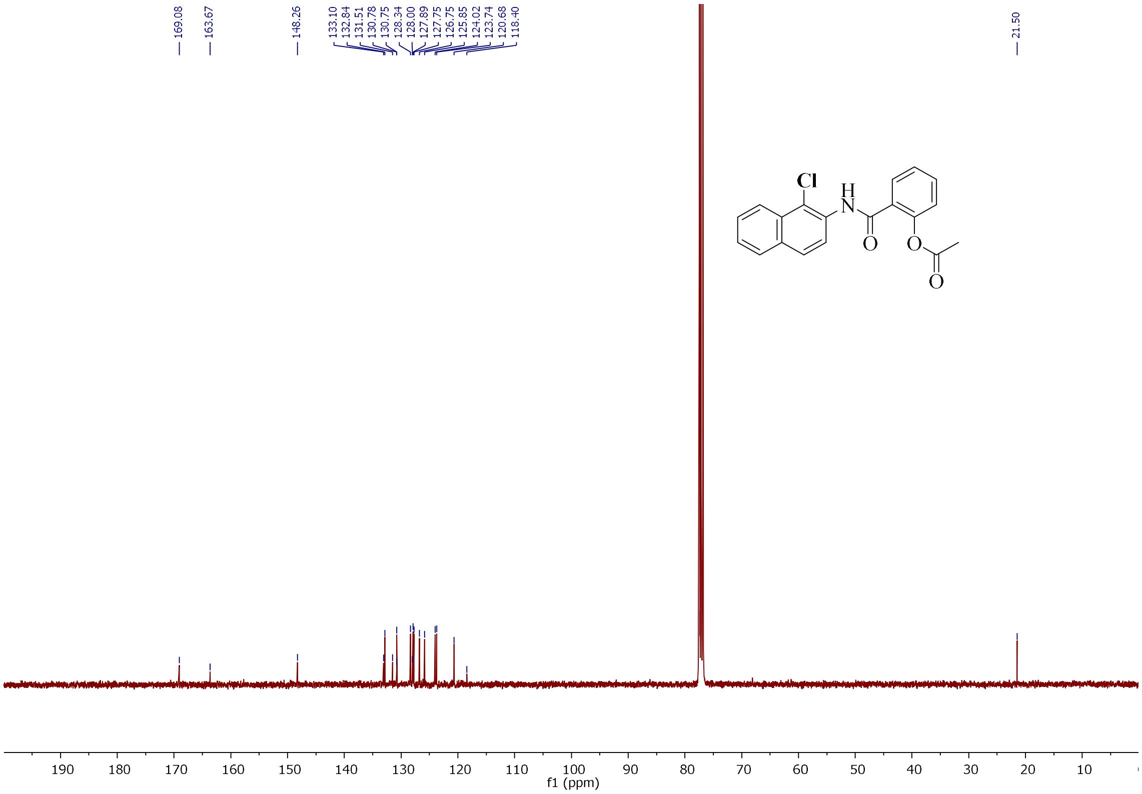

_N_-(2-chloro-4-(3-ethyl-2,6-dioxopiperidin-3-yl)phenyl)cyclohexanecarboxamide: (142)

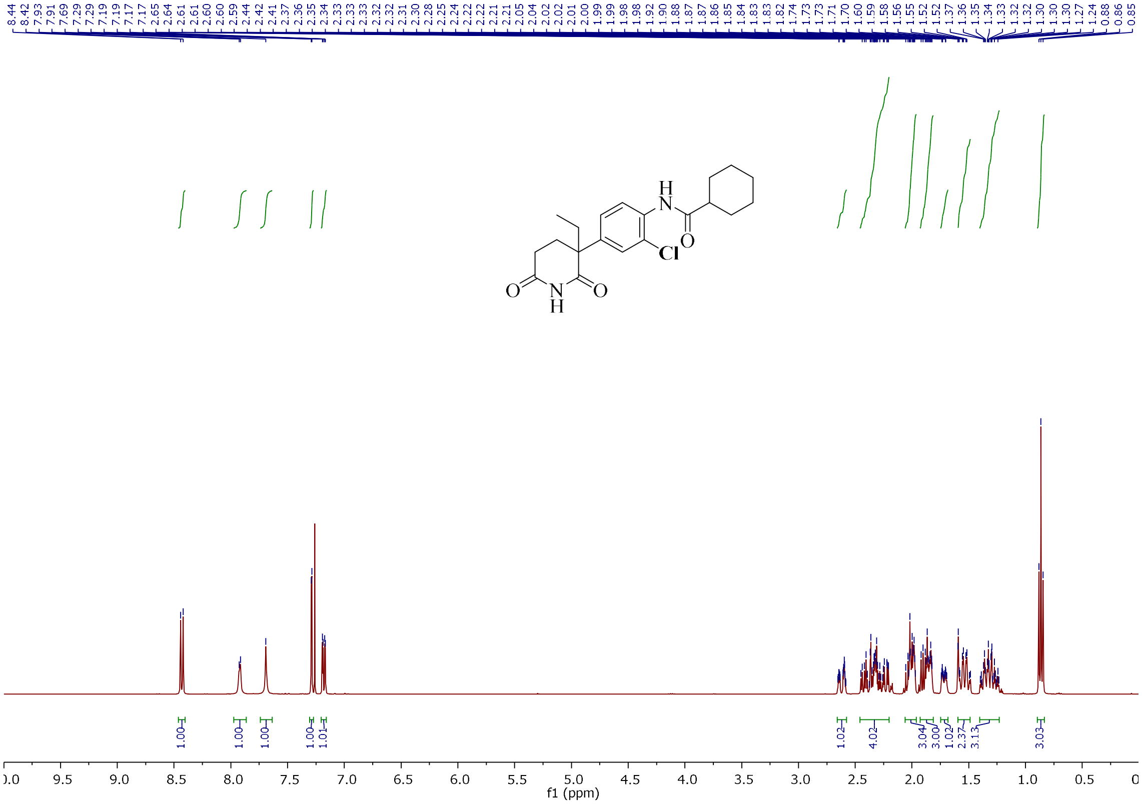

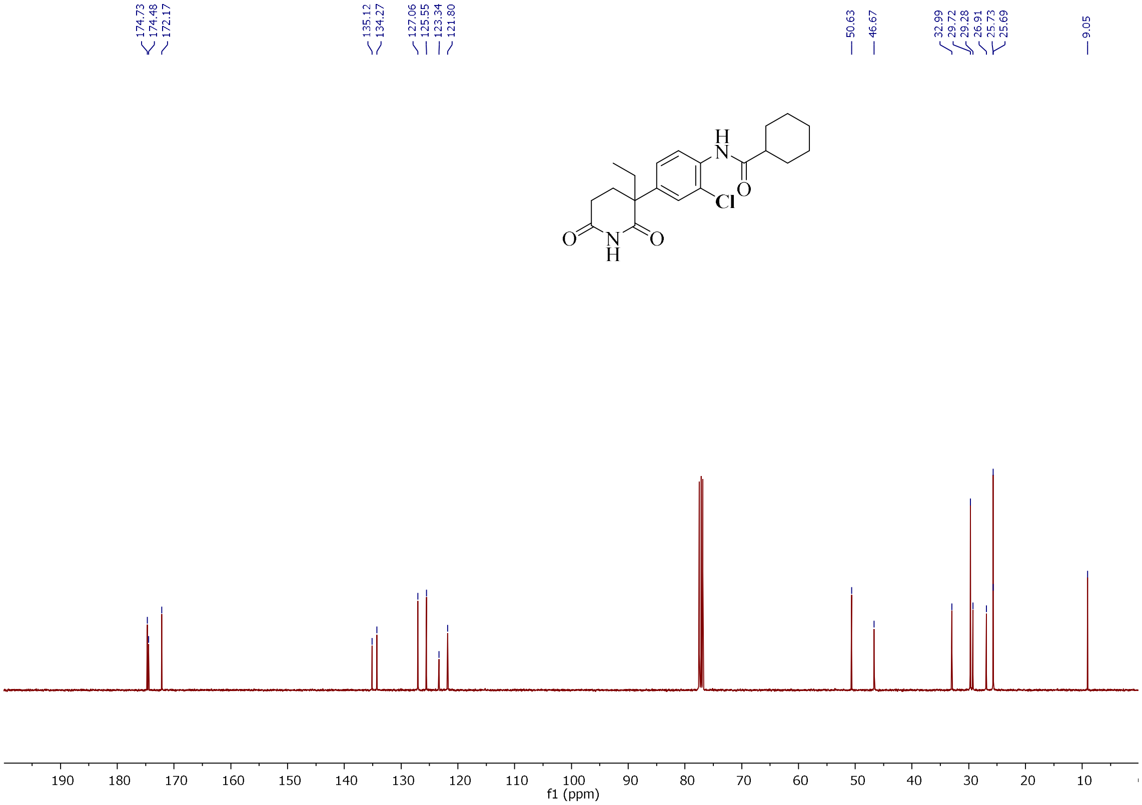

N-(2-chloro-4-(3-ethyl-2,6-dioxopiperidin-3-yl)phenyl)acetamide: (143)

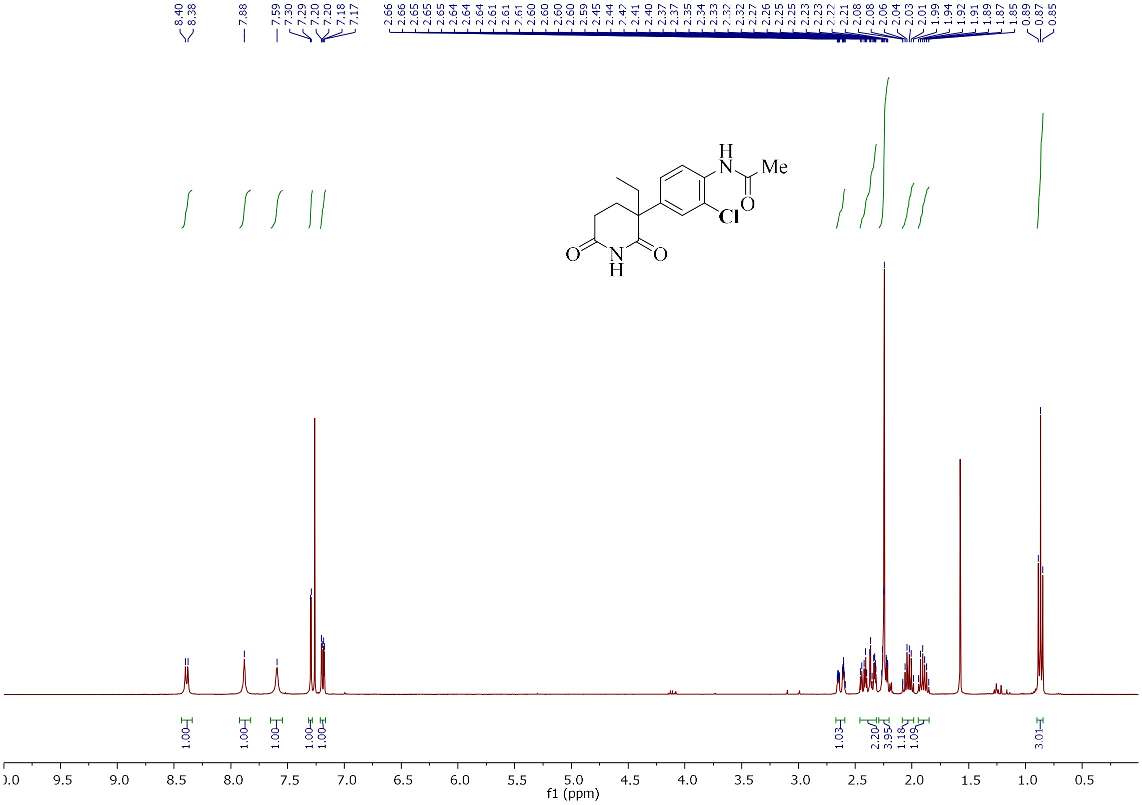

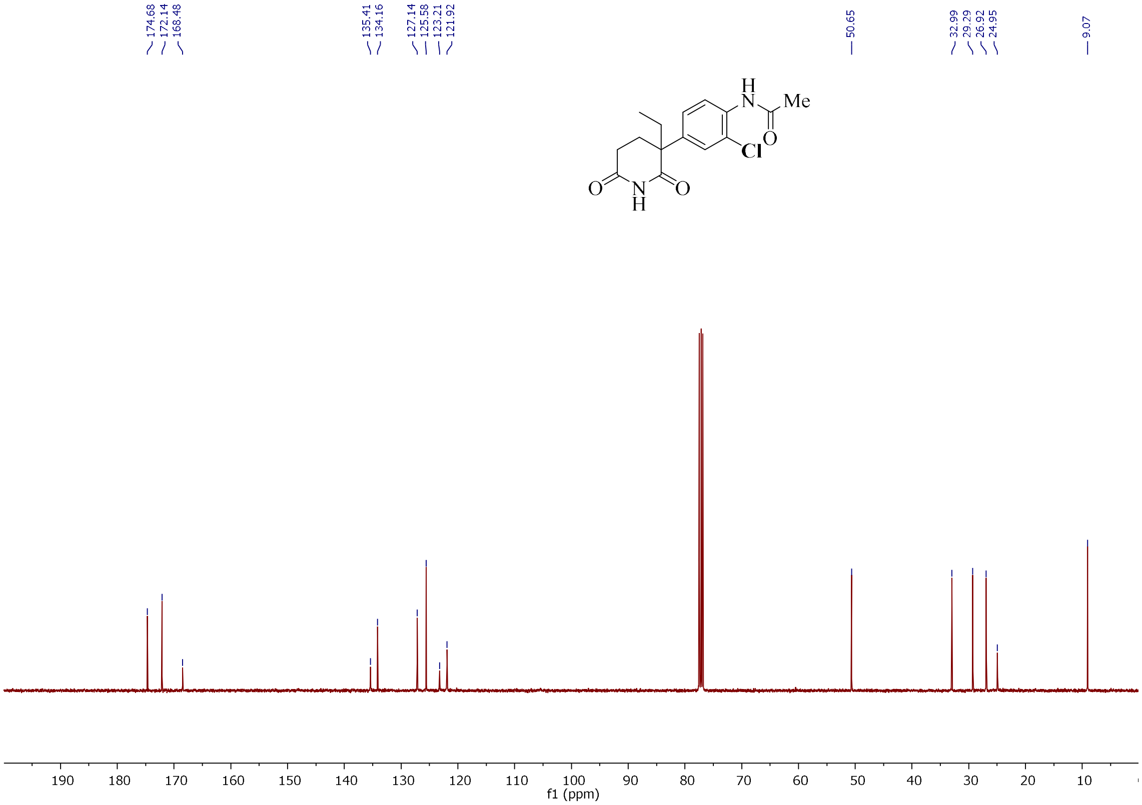

***N*-(2-bromo-4-(3-ethyl-2,6-dioxopiperidin-3-yl)phenyl)propionamide: (144)**

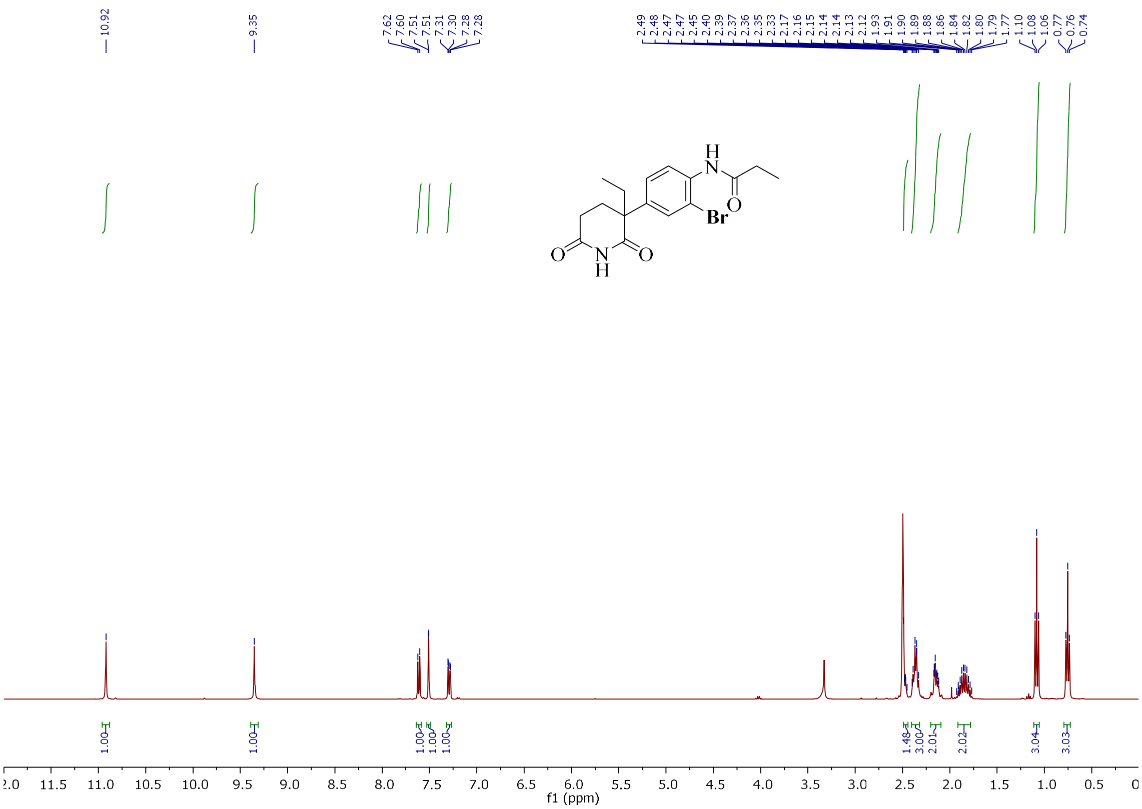

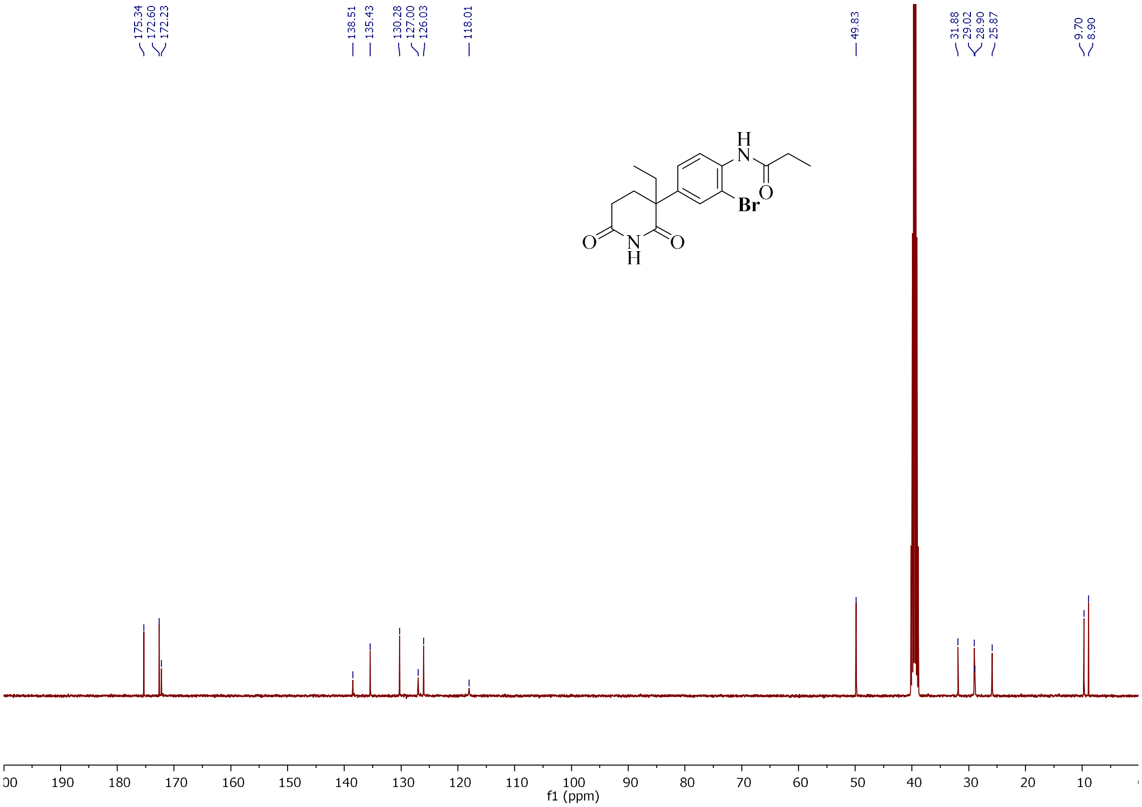

***N*-(3-chloro-4-hydroxyphenyl)acetamide: (145)**

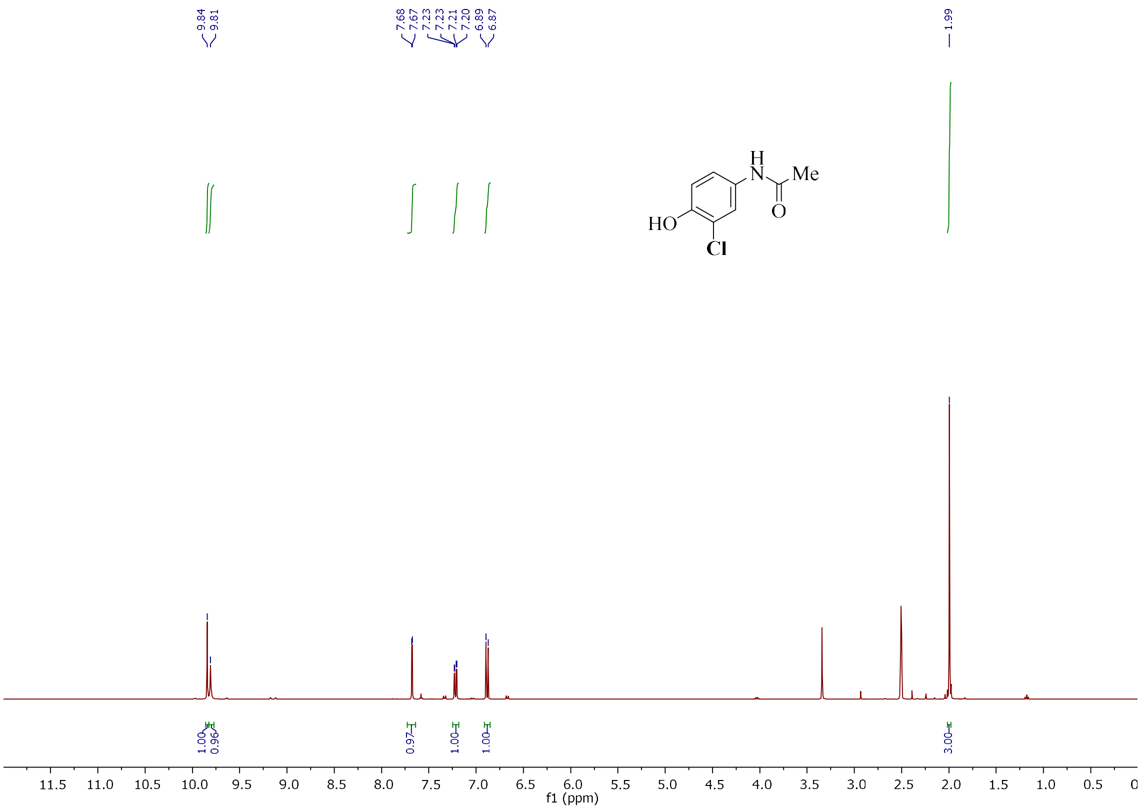

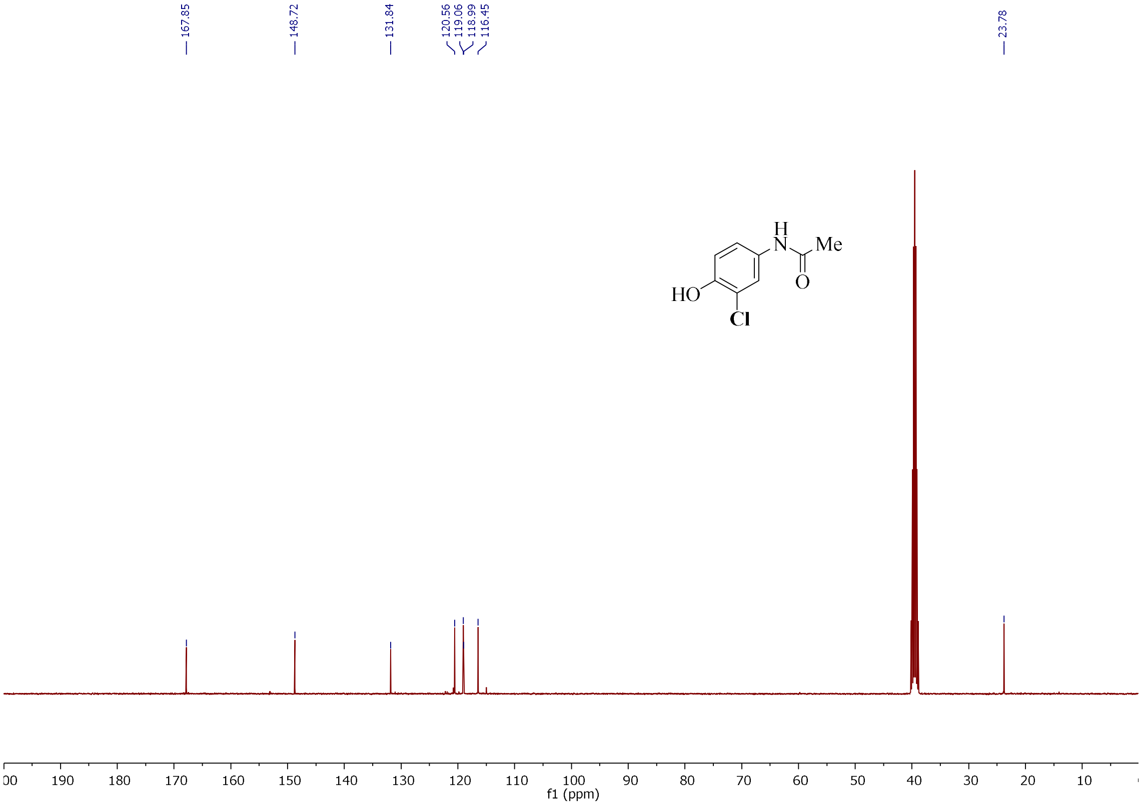

***N*-(3,5-dichloro-4-hydroxyphenyl)acetamide: (146)**

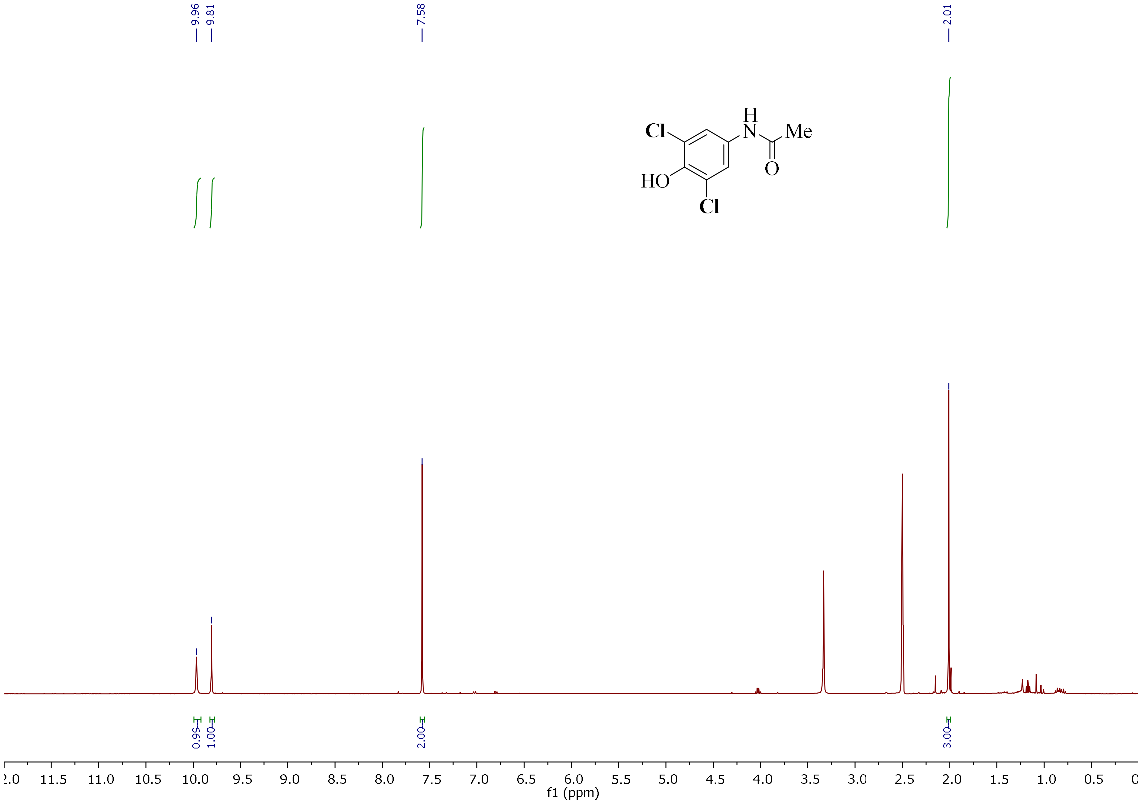

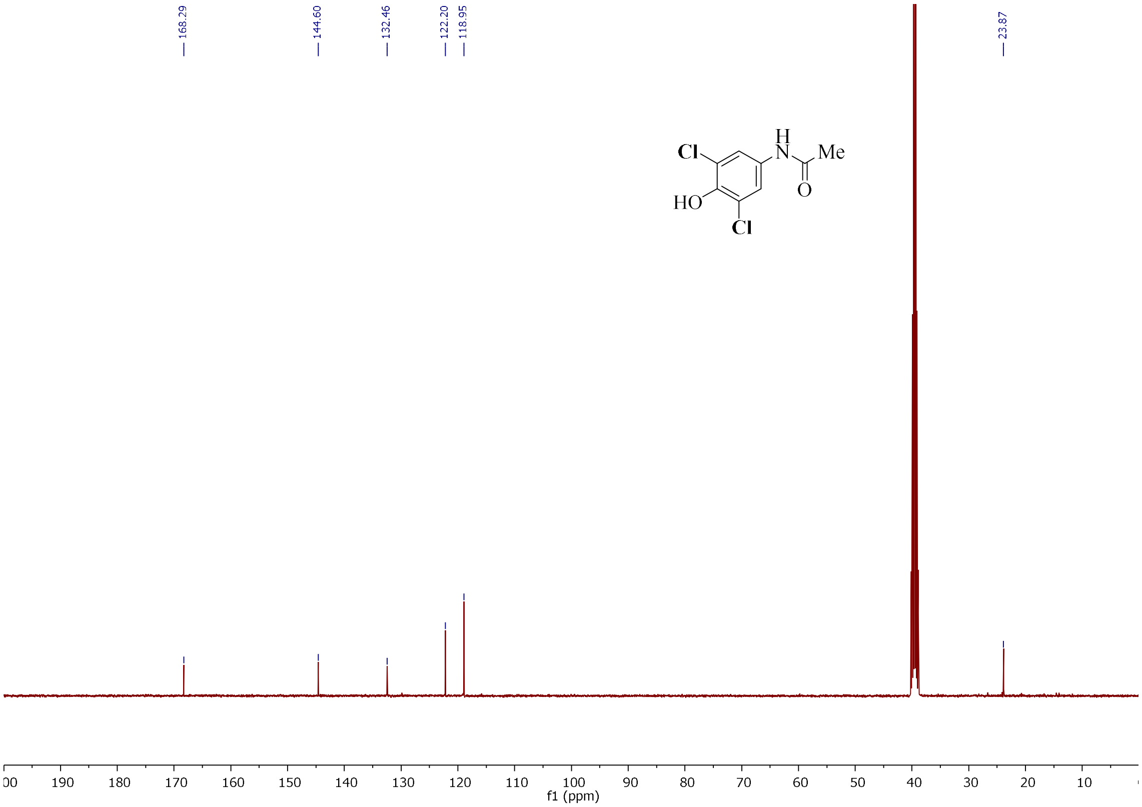

***N*-(8-bromo-3,4-dihydro-2*H*-benzo[*b*][1,4]dioxepin-7-yl)propionamide: (147)**

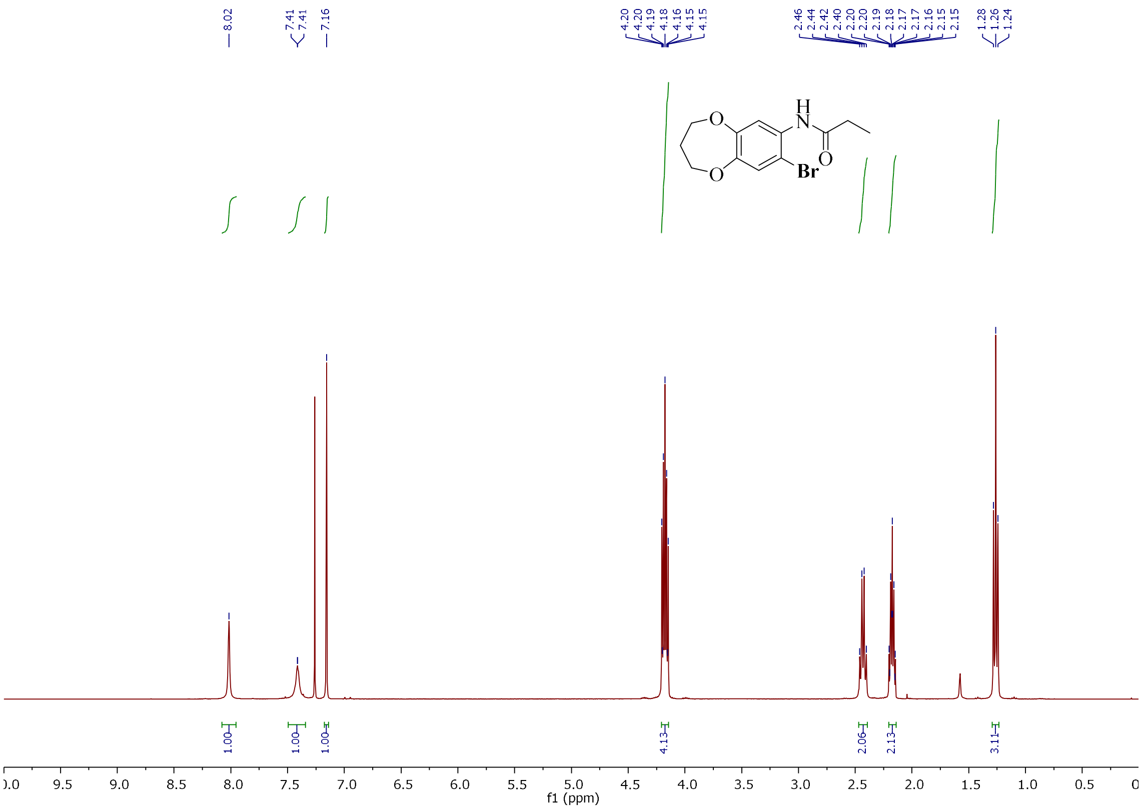

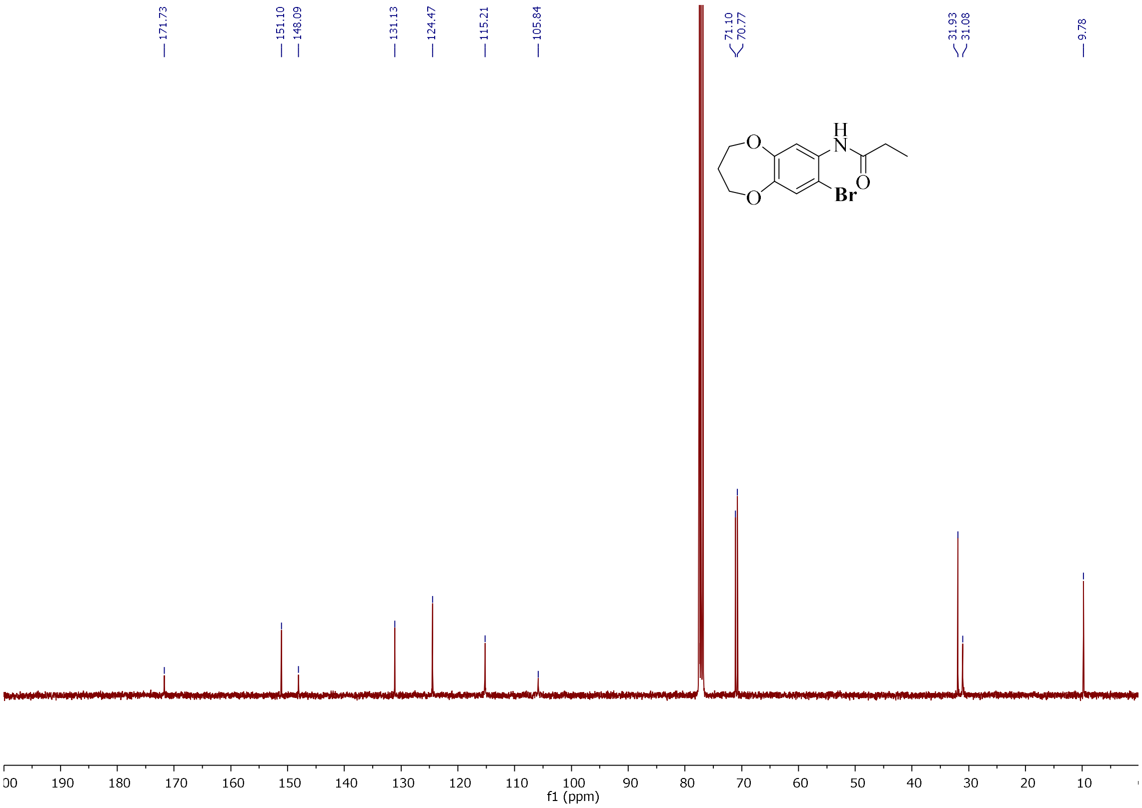

N-(7-bromo-2-(2,6-dioxopiperidin-3-yl)-1-oxoisoindolin-4-yl)propionamide: (148)

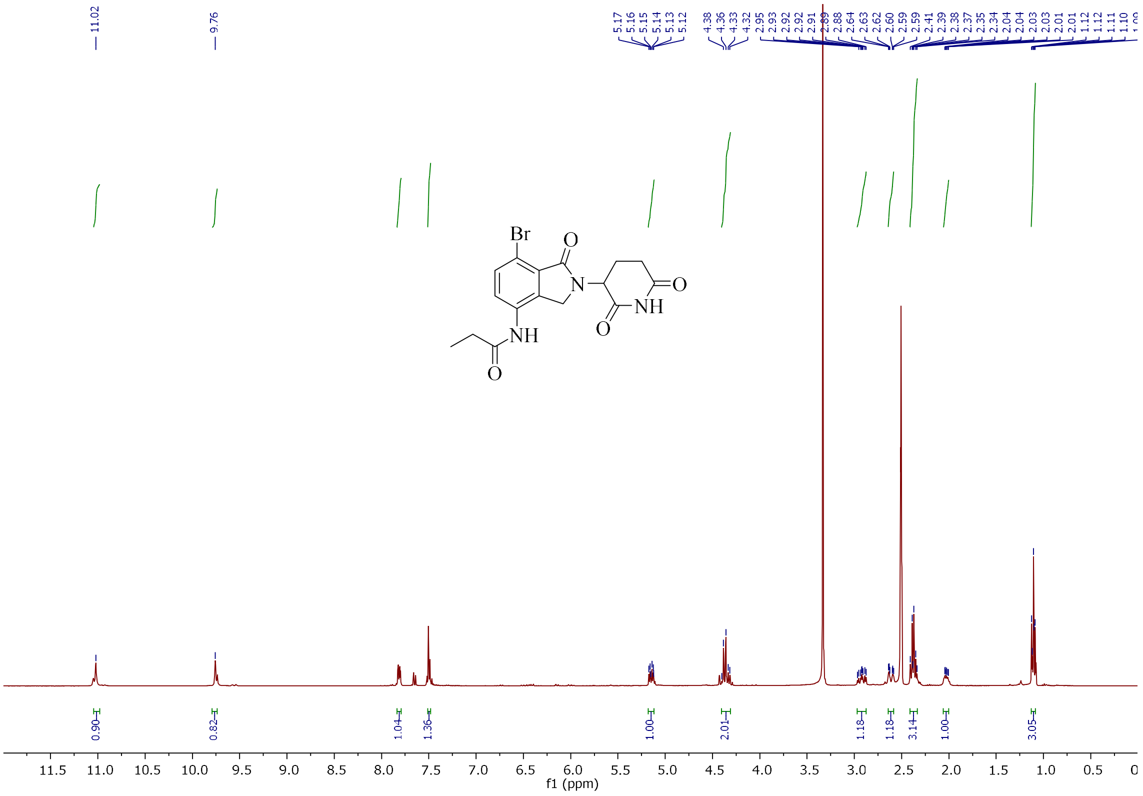

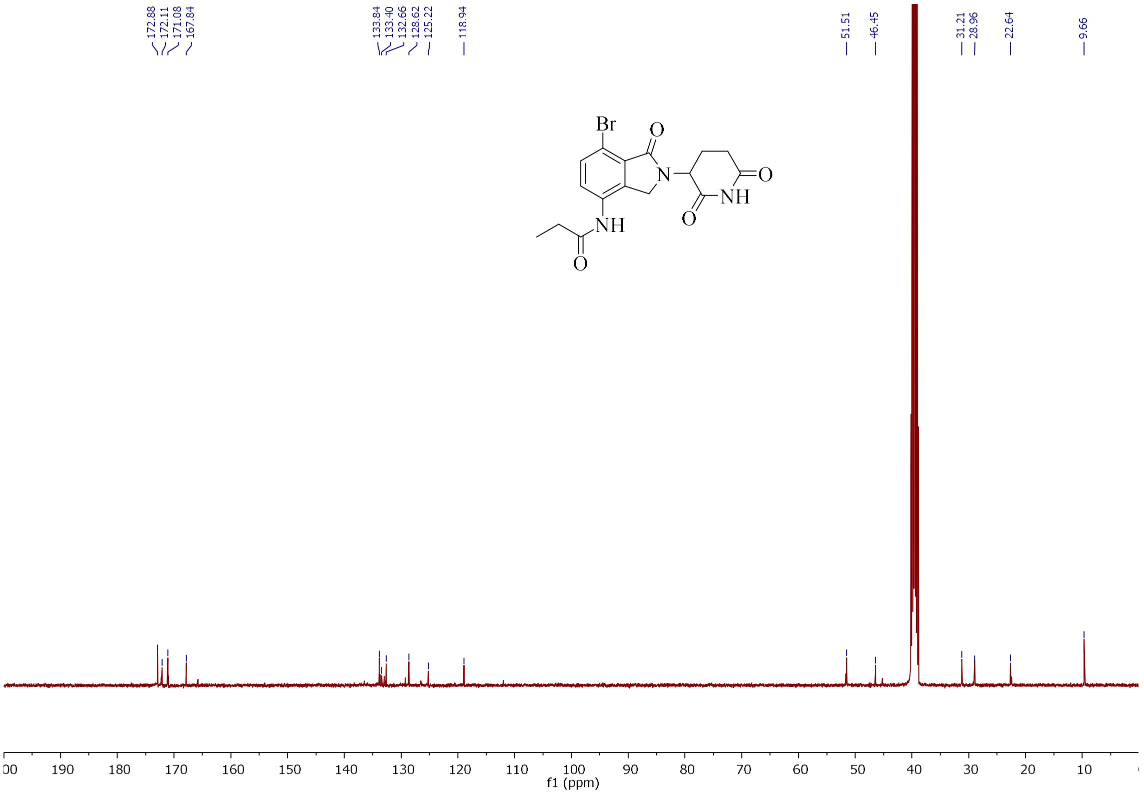

_N_-(6,8-dichloro-3,4-dihydro-2_H_-benzo[_b_][1,4]dioxepin-7-yl)cyclohexanecarboxamide: (149)

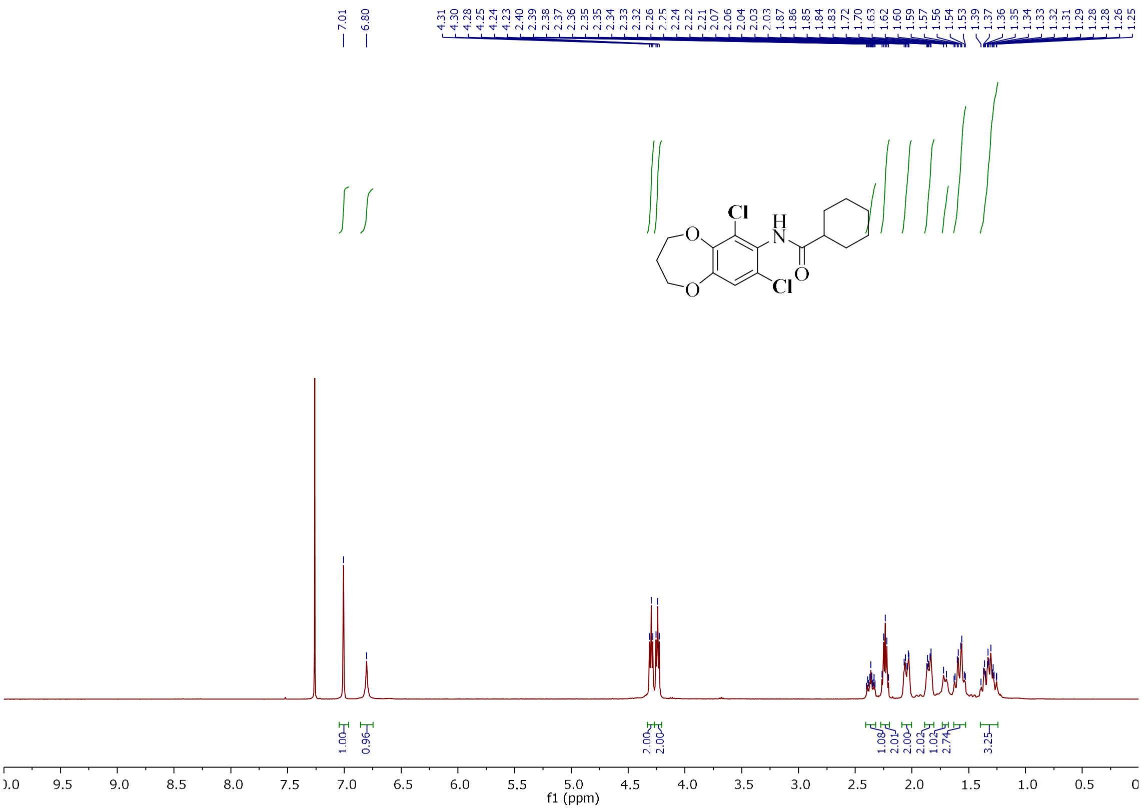

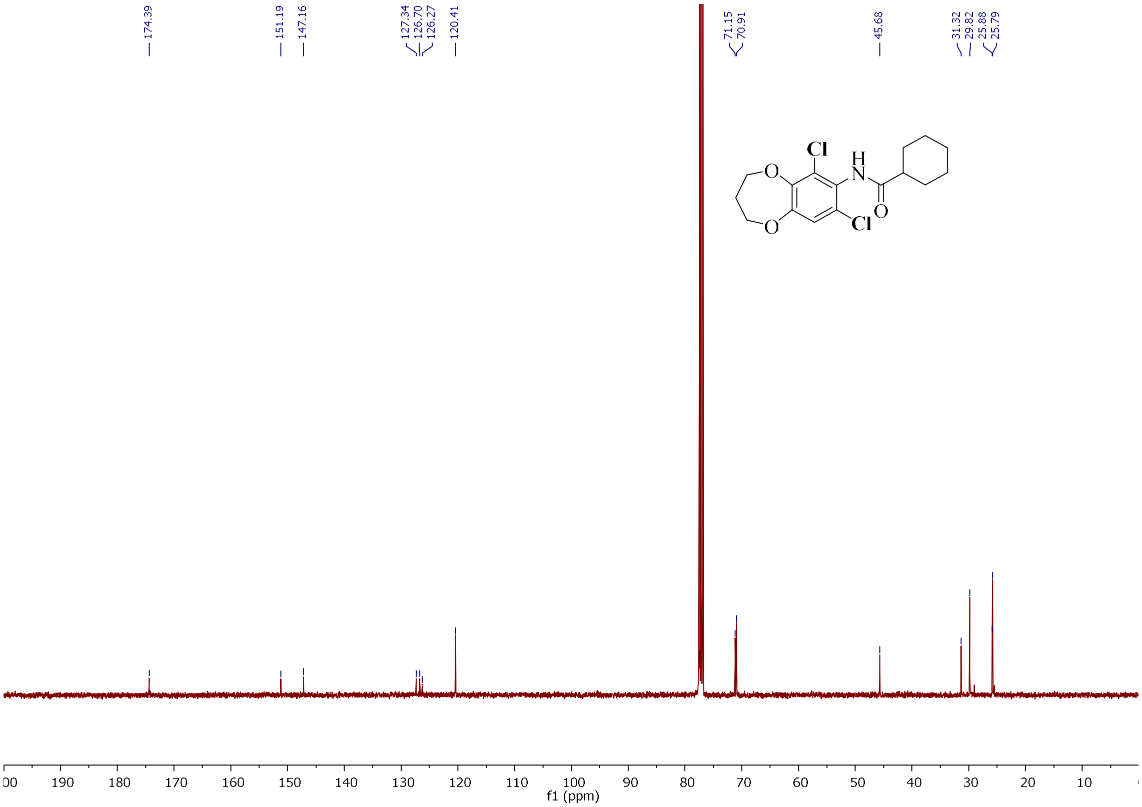

NMR studies of DMA.Br_2 formation:

Direct spectroscopic observation of a DMA·Br_2 intermediate; ^{1}H experiments conducted by mixing DMA with halogen at low temperature (0 ºC) resulted in shifting down field in proton NMR (as shown below).

However, no detectable spectral shift for DMA·Br_2 was observed in our cascade reaction condition, presumably due to its low concentration and rapid consumption of DMA.Br_2 complex under the reaction conditions.

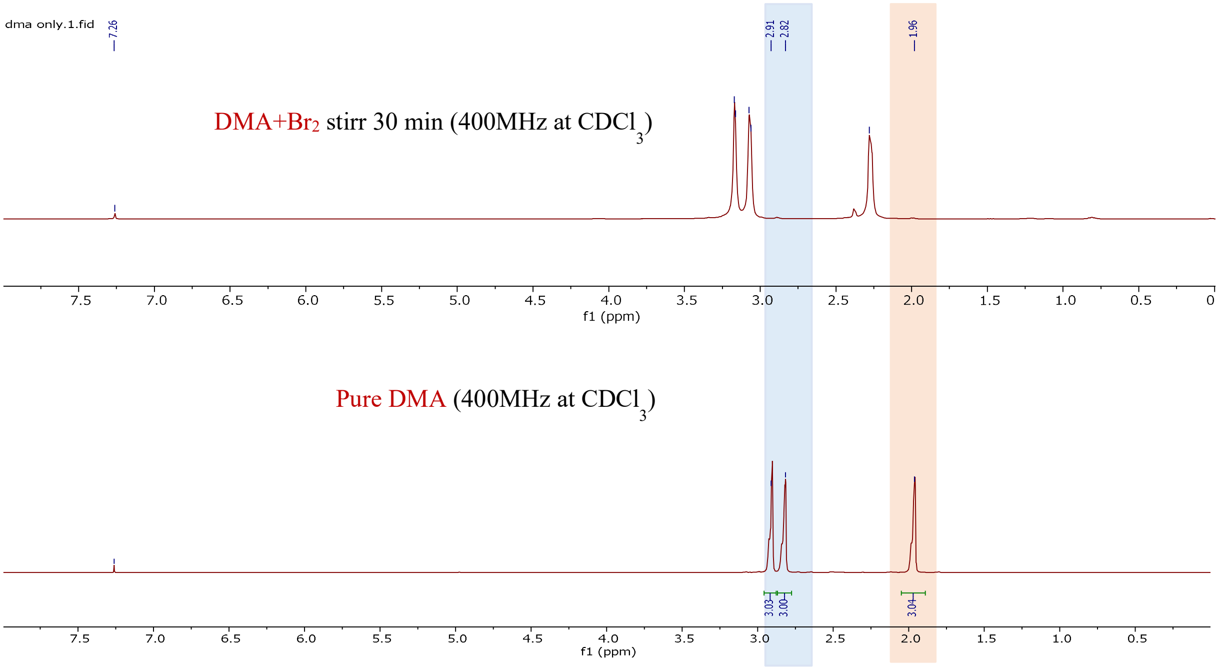

Supplement: Supplementary file 1 — Supporting File 1: anie71887‐sup‐0001‐SuppMat.pdf. [file ANIE-65-e9028210-s001.pdf]
